# Supplementary material for: A third generation of radical fluorinating agents based on N-fluoro-N-arylsulfonamides
Source: Nat Commun. 2018 Nov 20;9:4888. doi: 10.1038/s41467-018-07196-9 (PMC6244228; doi:10.1038/s41467-018-07196-9)
Supplement: Supplementary file 1 — Supplementary Information [file 41467_2018_7196_MOESM1_ESM.pdf]

A third Generation of Radical Fluroinating Agents Based on *N*-Fluoro-*N*-Arylsulfonamides

Meyer et al.

Supplementary Methods **4**
Experimental work 4
General Information 4
Synthesis of Reagents 4
Synthesis of Alkenes 7
Synthesis of Peresters 14
Synthesis of Amides and Sulfonamides 18
Synthesis of *N*-fluoro-*N*-alkyl amides/sulfonamides 25
Crystal structures of NFASs[22,23] 31
Hydrofluorination 34
Kinetic Experiments 46
Decarboxylative Fluorination 47
NMR-Spectra 51
Theoretical calculations **155**
Methodology 155
Structural Comparison (X-Ray vs QM) 156
Bond Strengths (F—NR_2) 157
(U)B3LYP/6-31G(d) 157
(RO)B2PLYP/B2PLYP/G3MP2Large 158
G3(MP2)-RAD 159
Bond Strengths (H—NR_2) 162
QM Data (For Bond Strengths) 163
Free Energy (ΔG) & Enthalpy (ΔH) Surfaces 169
Free Energy & Enthalpy Surfaces in Gas Phase at (U)B3LYP/6-31G(d) 172
Free Energy Surfaces in DMF Solution (Gas Phase Optimized) 174
Charge and Spin Analysis (Gas Phase Optimized) 175
Free Energy & Enthalpy Surfaces at (RO)B2PLYP/G3MP2Large (Gas Phase Optimized) 176
Free Energy & Enthalpy Surfaces in DMF Solution (Solution Phase Optimized) 177

Charge and Spin Analysis (Solution Phase Optimized) 180
(U)B3LYP/6-31G(d) - Energy Surfaces with Conformational Details (Gas) 181
(U)B3LYP/6-31G(d) - Energy Surfaces with Conformational Details (DMF) 182
(U)B3LYP/6-31G(d) - Energy Surfaces with Conformational Details (DMF,opt) 184
QM Data (For Free Energy & Enthalpy Surfaces) 186
References 192

Supplementary Methods

Experimental work

General Information

Starting materials were provided from Sigma Aldrich and Fluorochem. All reactions involving oxygen- or moisture-sensitive compounds were carried out under a dry argon atmosphere. Column chromatography was performed with silica gel (pore size 60 Å, 230-400 mesh particle size) packed in glass columns or using an automated CombiFlash-system. Reactions were monitored by thin layer chromatography (TLC) using Silicycle silica gel 60 F254 plates (0.2 mm thickness). Visualization was performed by ultraviolet light or by PMA- or bromocresol green stain, followed by gentle heating. NMR spectra were recorded at 300 MHz (for ^{1}H-NMR), 75/100 MHz (for ^{13}C-NMR), 282 MHz (for ^{19}F-NMR) and 96 MHz (for ^{11}B-NMR) in $CDCl_3$ or C_6D_6. Chemical shifts are reported as δ (ppm) downfield from tetramethylsilane (δ = 0.00) using residual solvent signal as an internal standard: δ singlet 7.26 (^{1}H), triplet 77.0 (^{13}C). IR spectra were obtained on neat samples (ATR probe). High-resolution mass spectra were recorded on an ESI-Orbitrap MS or a two sector field high-resolution mass analyzer. Gas chromatographic (GC) analyses were carried out with a Macherey-Nagel Optima delta-3-0.25 µm capillary column (20 m or 30 m, 0.25 mm). Gas carrier: He 1.4 mL/min; injector: 220 °C split mode; detector: FID 280 °C, H_2 35 mL/min, air 350 mL/min. Enantiomeric purity was determined by HPLC on a CHIRALPAK IB-3 (250 mm, 4.6 mm, 3 µm).

Synthesis of Reagents

Di-*tert*-butylhyponitrite (DTBHN)

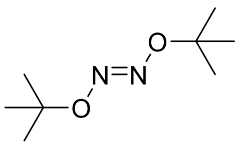

$C_8H_{18}N_2O_2$
174.24 g/mol

According to the literature procedure[1], sodium *trans*-hyponitrite hydrate was dried under high vacuum for 3 days to a constant weight. The dry sodium *trans*-hyponitrite (5.37 g, 50.7 mmol) was added to *tert*-butyl bromide (45.5 mL, 405 mmol) followed by the addition of dry Et_2O (25 mL) and cooled to −5 °C. A suspension of $ZnCl_2$ (2 M in Et_2O, 30.4 mL, 60.8 mmol) was cannulated to the reaction mixture at <0 °C. The suspension was allowed to stir at room temperature for 1.5 h and then filtrated. The filtrate was extracted with water (100 mL) and the aqueous layer was extracted with Et_2O (50 mL). The organic layers were washed with brine (50 mL), dried over Na_2SO_4, and concentrated under reduced pressure at <25 °C. The residue was crystallized from pentane to yield di-*tert*-butylhyponitrite (4.56 g, 52%) as white crystals: ^{1}H-NMR (300 MHz, $CDCl_3$): δ 1.39 (s, 9H); ^{13}C-NMR (75 MHz, $CDCl_3$): δ 81.2, 27.8.

Di-*tert*-butyl peroxyoxalate (DBPO)

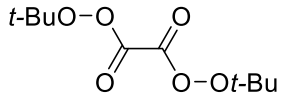

$C_{10}H_{18}O_6$
234.25 g/mol

According to the literature procedure[2], a solution of oxalyl chloride (858 µL, 10.0 mmol) in dry hexane (10 mL) was added to a stirred solution of pyridine (1.61 mL, 20.0 mmol) and *tert*-butyl hydroperoxide (3.64 mL, 5.5 mol/L in decane, 20.0 mmol) in hexane (20 mL) at 0 to −8 °C. The reaction mixture was allowed to warm to 15 °C, filtrated and washed with pentane. The reaction mixture was cooled to −78 °C and the liquid was decantated. The residue was twice diluted with pentane (20 mL), cooled to −78 °C, and decantated. The residue was crystallized from pentane at −25 °C to yield di-*tert*-butyl peroxyoxalate (2.14 g, 91%) as colorless needles: ^{1}H-NMR (300 MHz, $CDCl_3$): δ 1.38 (s, 18H); ^{13}C-NMR (101 MHz, $CDCl_3$): δ 154.2 (very weak signal), 85.9, 26.0.

(+)-Isopinocampheylborane TMEDA complex

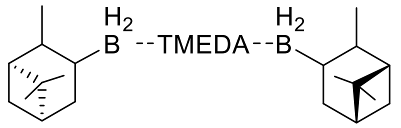

$C_{20}H_{38}B_2$
300.14 g/mol

According to the literature procedure[3], borane dimethyl sulfide complex (15.0 mL, 150 mmol) was dissolved in dry Et_2O (85 mL) and (–)-α-pinene (54.8 mL, 345 mmol) was added dropwise in such a rate that the reaction mixture refluxed gently. The mixture was refluxed for 1 h. Tetramethylethylenediamine (11.25 mL, 75 mmol) was added to the reaction mixture and refluxed for a further 1 h. Seedlings of (+)-$(IpcBH_2)_2$·TMEDA formed by taking one drop with a pipette and let dry on air. These were put in the hot solution whereby the product started to crystallize. The suspension was allowed to cool to room temperature and stored in the fridge at 0 °C overnight. The suspension was filtrated and washed with pentane. The crude product was dried under high vacuum to yield (+)-$(IpcBH_2)_2$·TMEDA (25.40 g, 81%) as a white solid: ^{1}H-NMR (300 MHz, $CDCl_3$): δ 3.34–3.07 (m, 4H), 2.63 (s, 6H), 2.59 (s, 6H), 2.25–2.15 (m, 2H), 2.14–2.03 (m, 2H), 1.88–1.77 (m, 4H), 1.76–1.70 (m, 2H), 1.62–1.51 (m, 2H), 1.16 (s, 6H), 1.09 (s, 6H), 1.00 (d, J = 7.0 Hz, 6H), 0.78 (d, J = 8.9 Hz, 2H), 0.72–0.60 (m, 2H); ^{13}C-NMR (75 MHz, $CDCl_3$): δ 57.2, 50.9, 50.8, 48.7, 43.0, 42.3, 39.0, 38.0, 34.1, 28.4, 25.3, 22.9, 22.7; ^{11}B (96 MHz, $CDCl_3$): δ 1.2 (s); $[\alpha]_D^{23}$ +67.9 (c 9.3, THF).

(+)-Monoisopinocampheylborane ((+)-IpcBH$_2$)

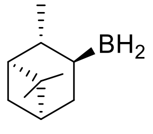

$C_{10}H_{19}B$
150.07 g/mol

According to the literature procedure[4], to a suspension of (+)-(IpcBH$_2$)$_2$·TMEDA (10.41 g, 25.0 mmol) in dry Et$_2$O (34 mL) boron trifluoride diethyl etherate (6.15 mL, 48.5 mmol) was added dropwise at room temperature. The mixture was allowed to stir at room temperature for 3 h. Then the suspension was transferred to a filtration chamber under argon using a double-ended needle. The solid TMEDA-2BF$_3$ was washed with dry Et$_2$O (2 × 17 mL). The combined filtrate was analyzed for (+)-IpcBH$_2$ by hydrolysis[5] with glycerol:water:THF (10/10/10 mL) as the hydrolyzing mixture and found to be 0.76 M: 61 mL; 93% yield; ^{11}B (96 MHz, CDCl$_3$): δ 22.6 (s); $[\alpha]_D^{23}$ +52.9 (c 11.4, Et$_2$O) (Lit. $[\alpha]_D^{23}$ +39.93, c 11.6, Et$_2$O).

4-Methoxy-2,6-dimethylbenzenesulfonyl chloride

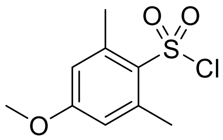

$C_9H_{11}ClO_3S$
234.69 g/mol

According to the literature procedure[6], to a solution of 3,5-dimethylanisol (4.09 g, 30.0 mmol) in dry DCM (40 mL) was added a solution of chlorosulfonic acid (4.59 mL, 69.0 mmol) in dry DCM (20 mL) at 0 °C over 20 min. The reaction mixture was allowed to stir at 0 °C for 10 min and was then poured on ice-water (30 mL). The two layers were separated and the organic layer was washed with ice-water (2 × 30 mL). The water phases were extracted with DCM (30 mL). The organic layers were dried over Na$_2$SO$_4$ and concentrated. The crude product was purified by column chromatography (pentane/Et$_2$O 8:2) to provide 4-methoxy-2,6-dimethylbenzenesulfonyl chloride (4.78 g, 68%) as a colorless and clear liquid: R_f 0.40 (heptane/Et$_2$O 8:2); (300 MHz, CDCl$_3$): δ 6.69 (s, 2H), 3.87 (s, 3H), 2.73 (s, 6H); ^{13}C-NMR (75 MHz, CDCl$_3$): δ 163.1, 142.5, 134.8, 116.4, 55.6, 23.5.

4-(Dimethylamino)benzenesulfonyl chloride

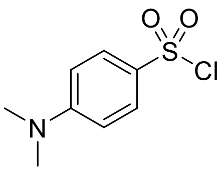

$C_8H_{10}ClNO_2S$
219.68 g/mol

According to the literature procedure[7], bistrimethylsilyl sulfate (7.23 g, 30.0 mmol) and *N*,*N*-dimethylaniline (3.64 g, 30.0 mmol) were heated in a round bottom flask equipped with a distillation bridge to 170 °C for 4 h. The mixture was cooled to room temperature and washed with ether (100 mL). The residue was dissolved in water (45 mL), concentrated and dried under high vacuum. The residue was added to a mixture of phosphorus pentachloride (6.87 g, 33.0 mmol) in DCM (90 mL) at 0 °C and stirred at this temperature for 4 h. The reaction mixture was concentrated under reduced pressure and diluted with water (100 mL). The mixture was extracted with DCM (100/50/50 mL). The organic layers were washed

with water (2 × 50 mL), dried over Na_2SO_4, and concentrated. The crude product was purified by recrystallization from hexane/TBME 7:3 to yield 4-(dimethylamino)benzenesulfonyl chloride (3.66 g, 52%) as an yellow-greenish solid: R_f 0.30 (heptane/EtOAc 7:3); m.p. 111–112 °C; ^{1}H-NMR (300 MHz, $CDCl_3$): δ 7.91–7.75 (m, 2H), 6.76–6.59 (m, 2H), 3.11 (s, 6H); ^{13}C-NMR (75 MHz, $CDCl_3$): δ 154.3, 129.4, 129.3, 110.7, 40.1.

Synthesis of Alkenes

4-(Cyclopent-1-en-1-yl)-1,1'-biphenyl (1b)

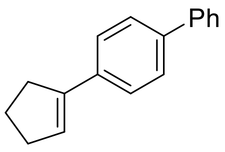

$C_{17}H_{16}$
220.32 g/mol

According to the literature procedure[8], to a mixture of magnesium tunings (0.73 g, 30.0 mmol) and bromobiphenyl (4.38 g, 18.8 mmol) was added a small piece of iodine and stirred under reflux for 2 h. The mixture was cooled to 0 °C, then cyclopentanone (1.26 g, 15.0 mmol) was slowly added and stirred at room temperature for 2 h. The reaction mixture was quenched by the addition of ice cold water (45 mL) and extracted with Et_2O (2 × 40 mL). The organic layers were washed with brine (2 × 40 mL), dried over Na_2SO_4, and concentrated. The crude product was purified by column chromatography (heptane/EtOAc 75:25) to yield the pure alcohol (2.76 g, 77%) as a white solid: ^{1}H-NMR (300 MHz, $CDCl_3$): δ 7.66–7.49 (m, 6H), 7.46–7.38 (m, 2H), 7.37–7.27 (m, 1H), 2.19–1.94 (m, 6H), 1.94–1.77 (m, 2H), 1.62 (s, 1H); ^{13}C-NMR: δ (75 MHz, $CDCl_3$) δ 146.1, 140.9, 139.7, 128.7, 127.2, 127.1, 127.0, 125.6, 83.4, 41.9, 23.9. A 1 M aqueous solution of acetic acid (100 mL) was added to the alcohol and stirred at reflux for 6 h. The reaction mixture was cooled to room temperature and quenched by addition of a 1 M aqueous solution of NaOH until neutral pH. The mixture was extracted with Et_2O (100/50 mL) and the organic layers were washed with brine (2 × 50 mL). The organic layers were dried over Na_2SO_4 and concentrated. The crude product was purified by crystallization from EtOAc/MeOH 6:4 to yield **1b** (2.34 g, 71% over 2 steps) as a white solid: R_f 0.60 (heptane); m.p. 155–156; ^{1}H-NMR (300 MHz, $CDCl_3$): δ 7.69–7.48 (m, 6H), 7.47–7.37 (m, 2H), 7.36–7.26 (m, 1H), 6.29–6.16 (m, 1H), 2.80–2.65 (m, 2H), 2.62–2.45 (m, 2H), 2.12–1.95 (m, 2H); ^{13}C-NMR (75 MHz, $CDCl_3$): δ 142.0, 140.9, 139.5, 135.8, 128.7, 127.1, 126.94, 126.91, 126.3, 126.0, 33.4, 33.2, 23.4.

2-((1*R*,5*S*)-6,6-Dimethylbicyclo[3.1.1]hept-2-en-2-yl)ethyl 4-methylbenzenesulfonate (1d)

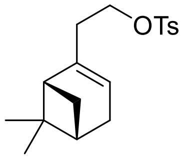

$C_{18}H_{24}O_3S$
320.45 g/mol

To a solution of (−)-Nopol (4.99 g, 30.0 mmol) and triethylamine (4.25 g, 42.0 mmol) in dry DCM (45 mL) was added tosyl chloride (7.44 g, 39.0 mmol) at 0 °C. The reaction mixture was allowed to stir at room temperature overnight. The reaction was quenched by the addition of solution of ammonium hydroxide (25% in H_2O, 3 mL) and stirred at room temperature for 4 h. The mixture was diluted with water (120 mL) and extracted with Et_2O (120/60 mL). The organic layers were washed with a solution of $NaHSO_4$ (10% in H_2O, 60 mL) and water (2 × 60 mL). The organic layers were dried over Na_2SO_4 and concentrated. The crude product was purified by column chromatography (heptane/EtOAc 9:1) to yield **1d** (8.80 g, 91%) as a colorless and clear oil: R_f 0.30 (heptane/EtOAc 9:1); ^{1}H-NMR (300 MHz, $CDCl_3$): *δ* 7.78 (d, *J* = 8.3 Hz, 2H), 7.34 (d, *J* = 8.3 Hz, 2H), 5.34–5.18 (m, 1H), 4.03 (t, *J* = 7.0 Hz, 2H), 2.45 (s, 3H), 2.36–2.26 (m, 3H), 2.26–2.10 (m, 2H), 2.09–2.01 (m, 1H), 1.93 (td, *J* = 5.6, 1.2 Hz, 1H), 1.24 (s, 3H), 1.07 (d, *J* = 8.6 Hz, 1H), 0.77 (s, 3H); ^{13}C-NMR (75 MHz, $CDCl_3$): δ 144.6, 142.6, 133.3, 129.8, 127.9, 119.7, 68.6, 45.6, 40.6, 38.0, 36.1, 31.5, 31.3, 26.2, 21.6, 21.1.

(1*R*,5*S*)-6,6-Dimethyl-2-(4-methylpentyl)bicyclo[3.1.1]hept-2-ene (1c)

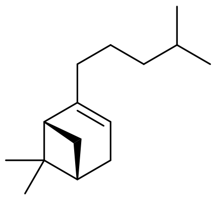

$C_{15}H_{26}$
206.37 g/mol

According to the literature procedure[9], to a stirred suspension of magnesium tunings (0.87 g, 36.0 mmol) in dry THF (15 mL) was added 1-bromo-2-methylpropane (4.73 g, 34.5 mmol) in dry THF (7.5 mL) in such a rate that the mixture was gently refluxing. The mixture was stirred under reflux for 30 min and then cooled to −78 °C. The tosylate **1d** (4.81 g, 15.0 mmol) in dry THF (7.5 mL) and CuI (0.40 g, 2.1 mmol) were added. The mixture was stirred at 0 °C for 3 h and at room temperature overnight. The reaction was quenched by the addition of a saturated solution of NH_4Cl (30 mL) and extracted with pentane (120/60 mL). The organic layers were washed with a saturated solution of $NaHCO_3$ (30 mL) and brine (30 mL). The organic layers were dried over Na_2SO_4 and concentrated. The crude product was purified by column chromatography (pentane) to yield **1c** (3.04 g, 98%) as a colorless and clear liquid: R_f 0.90 (heptane); ^{1}H-NMR (300 MHz, $CDCl_3$): *δ* 5.23–5.13 (m, 1H), 2.41–2.31 (m, 1H), 2.30–2.12 (m, 2H), 2.11–2.04 (m, 1H), 2.00 (td, *J* = 5.7, 1.3 Hz, 1H), 1.94–1.86 (m, 2H), 1.59–1.45 (m, 1H), 1.36–1.24 (m, 2H), 1.27 (s, 3H), 1.19–1.1 (m, 3H), 0.87 (s, 3H), 0.85 (s, 3H), 0.83 (s, 3H); ^{13}C-NMR (75 MHz, $CDCl_3$): δ 148.8, 115.5, 45.9, 40.9, 38.9, 37.9, 37.2, 31.3, 27.9, 26.4, 25.0, 22.70, 22.65, 21.2.

(1*R*,5*S*)-2-((Benzyloxy)methyl)-6,6-dimethylbicyclo[3.1.1]hept-2-ene (1e)

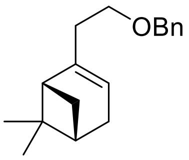

$C_{18}H_{24}O$
256.18 g/mol

According to the literature procedure[1], to a suspension of NaH (0.345 g, 15.0 mmol) in dry THF (30 mL) was added (−)-nopol (1.66 g, 10.0 mmol) at 0 °C. The reaction mixture was stirred at room temperature for 30 min and then benzyl bromide (2.22 g, 13.0 mmol) was added. The reaction mixture was allowed to stir at room temperature for 1 d. The reaction mixture was cooled to 0 °C, diluted with water (30 mL) and extracted with Et_2O (60/30 mL). The organic layers were washed with water (2 × 30 mL), dried over Na_2SO_4, and concentrated. The crude product was purified by column chromatography (pentane/Et_2O 98:2) to yield **1e** (2.47 g, 96%) as a colorless and clear liquid: R_f 0.20 (heptane/Et_2O 98:2); ^{1}H-NMR (300 MHz, $CDCl_3$): *δ* 7.48–7.16 (m, 5H), 5.32–5.20 (m, 1H), 4.49 (s, 2H), 3.49 (t, *J* = 7.1 Hz, 2H), 2.41–2.11 (m, 5H), 2.10–1.99 (m, 2H), 1.26 (s, 3H), 1.16 (d, *J* = 8.5 Hz, 1H), 0.82 (s, 3H); ^{13}C-NMR (75 MHz, $CDCl_3$): δ 145.2, 138.6, 128.3, 127.6, 127.5, 117.9, 72.8, 68.9, 45.8, 40.8, 38.0, 37.2, 31.7, 31.3, 26.3, 21.2.

3,7-Dimethyloct-6-en-1-yl benzoate (1f)

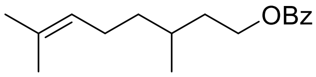

$C_{17}H_{24}O_2$
260.38 g/mol

According to the literature procedure[10], to a solution of beta-citronellol (3.13 g, 20.0 mmol) and triethylamine (2.83 g, 28.0 mmol) in dry Et_2O (30 mL) was added benzoyl chloride (3.65 g, 26.0 mmol) at 0 °C. The reaction mixture was stirred at room temperature overnight. The reaction was quenched by the addition of a solution of NH_4OH (25% in H_2O, 4 mL). The mixture was diluted with water (100 mL) and extracted with Et_2O (100/50 mL). The organic layers were washed with a solution of $NaHSO_4$ (10% in H_2O, 50 mL) and water (50 mL). The organic layers were dried over Na_2SO_4 and concentrated. The crude product was purified by column chromatography (pentane/Et_2O 97:3) to yield **1f** (5.14 g, 99%) as a colorless and clear liquid: R_f 0.50 (heptane/Et_2O 97:3); ^{1}H-NMR (300 MHz, $CDCl_3$): *δ* 8.10–7.95 (m, 2H), 7.60–7.49 (m, 1H), 7.49–7.35 (m, 2H), 5.14–5.01 (m, 1H), 4.44–4.25 (m, 2H), 2.13–1.92 (m, 2H), 1.90–1.76 (m, 1H), 1.73–1.49 (m, 8H), 1.48–1.35 (m, 1H), 1.28–1.17 (m, 1H), 0.97 (d, *J* = 6.4 Hz, 3H); ^{13}C-NMR (75 MHz, $CDCl_3$): δ 166.7, 132.8, 131.4, 130.5, 129.5, 128.3, 124.6, 63.5, 37.0, 35.5, 29.6, 25.7, 25.4, 19.5, 17.7.

1,1'-Bi(cyclohexylidene) (2h)

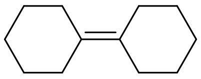

$C_{12}H_{20}$
164.29 g/mol

According to the literature procedure[11], to a suspension of zinc powder (16.4 g, 250 mmol) in dry THF (350 mL) was slowly added $TiCl_4$ (13.7 mL, 125 mmol) at <10 °C. The mixture was allowed to stir at room temperature for 0.5 h and at reflux for 2.5 h. The mixture was cooled to –5 °C, charged with pyridine (5.0 mL, 62.5 mmol) and stirred for 10 min at this temperature. A solution of cyclohexanone (2.45 g, 25.0 mmol) in dry THF (30 mL) was added slowly. After addition, the reaction mixture was stirred at reflux overnight. The reaction was quenched by addition of a solution of K_2CO_3 (10% H_2O, 200 mL) and extracted with pentane (400/200 mL). The organic layers were washed with water (2 × 200mL), dried over Na_2SO_4, and concentrated. The crude product was purified by column chromatography (pentane) to yield **1h** (0.43 g, 21%) as a colorless and clear liquid: R_f 0.90 (heptane); ^{1}H-NMR (300 MHz, $CDCl_3$): δ 2.37–1.98 (m, 8H), 1.63–1.40 (m, 12H); ^{13}C-NMR (75 MHz, $CDCl_3$): δ 129.4, 30.1, 28.7, 27.3.

5-Methyloxepan-2-one

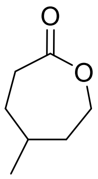

$C_7H_{12}O_2$
128.17 g/mol

According to the literature procedure [12], to a solution 4-methylcyclohexanone (22.43 g, 200 mmol) in DCM (250 mL) was added portion wise 3-chloroperoxybenzoic acid (77% in H_2O, 51.55 g, 230 mmol) at 0 °C. The reaction mixture was slowly warmed to room temperature and stirred overnight. The suspension was filtered. Then a solution of Na_2SO_3 (10% in H2O, 200 mL) was added and stirred at room temperature for 1 h. The phases were separated and the aqueous phase was extracted with DCM (200 mL). The organic layers were washed with a saturated solution of $NaHCO_3$ (200 mL) and water (200 mL). The organic layers were dried over Na_2SO_4 and concentrated. The crude product was purified by chromatographic filtration (pentane/Et_2O 3:7) followed by distillation (b.p. 78–79 °C, 2 mbar) to yield 5-methyloxepan-2-one (23.65 g, 92%) as a colorless and clear liquid: R_f 0.35 (pentane/Et_2O 3:7); ^{1}H-NMR (300 MHz, $CDCl_3$): δ 4.28 (ddd, J = 12.9, 5.6, 2.2 Hz, 1H), 4.18 (ddd, J = 12.9, 10.0, 1.1 Hz, 1H), 2.75–2.55 (m, 2H), 2.00–1.70 (m, 3H), 1.59–1.42 (m, 1H), 1.42–1.26 (m, 1H), 1.00 (d, J = 6.5 Hz, 3H); ^{13}C-NMR (75 MHz, $CDCl_3$): δ 176.1, 68.1, 37.3, 35.3, 33.2, 30.8, 22.2.

6-Methylhept-6-en-1-ol

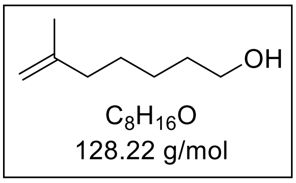

According to the literature procedure[13], to a solution of ε-caprolactone (9.13 g, 80.0 mmol) in dry Et_2O (240 mL) was added dropwise a solution of MeLi (3.0 M in diethoxymethane, 29.3 mL, 88.0 mmol) at −78 °C. The reaction was allowed to stir at −78 °C for 1 h and then quenched by the addition of a saturated solution of NH_4Cl (120 mL). The mixture was extracted with Et_2O (200/100 mL). The organic layers were washed with water (100 mL), dried over Na_2SO_4, and concentrated. The crude product was purified by column chromatography (Et_2O) to yield 7-hydroxyheptan-2-one (2.34 g, ~18.0 mmol, not fully pure). Methyltriphenylphosphonium bromide (19.29 g, 54.0 mmol) and potassium *tert*-butoxide (6.06 g, 54.0 mmol) in dry THF (90 mL) were stirred at reflux for 30 min. 7-Hydroxyheptan-2-one (2.34 g, ~18.0 mmol) was slowly added and stirred at reflux for 3 h. The reaction mixture was cooled to room temperature and quenched by the addition of a saturated solution of NH_4Cl (100 mL). The mixture was extracted with Et_2O (150/75 mL). The organic layers were washed with water (75 mL), brine (75 mL), dried over Na_2SO_4, and concentrated. The crude product was purified by column chromatography (pentane/Et_2O 5:5) to yield 6-methylhept-6-en-1-ol (1.36 g, 13%) as a colorless and clear liquid: R_f 0.35 (heptane/Et_2O 5:5); ^{1}H-NMR (300 MHz, $CDCl_3$): δ 4.68 (d, J = 8.4 Hz, 2H), 3.64 (t, J = 6.6 Hz, 2H), 2.02 (t, J = 7.1 Hz, 2H), 1.71 (s, 3H), 1.66–1.29 (m, 7H); ^{13}C-NMR (75 MHz, $CDCl_3$): δ 146.0, 109.8, 63.0, 37.7, 32.7, 27.4, 25.4, 22.4.

3,6-Dimethylhept-6-en-1-ol

Same procedure as above starting from 5-methyloxepan-2-one (4.49 g, 35.0 mmol), MeLi (3.0 M in diethoxymethane, 12.8 mL, 38.5 mmol) in dry THF (100 mL); column chromatography (Et_2O) yielded 7-hydroxy-5-methylheptan-2-one (3.17 g, ~22 mmol, not fully pure); this alcohol was then converted with methyltriphenylphosphonium bromide (23.58 g, 66.0 mmol) and potassium *tert*-butoxide (7.41 g, 66.0 mmol) in dry THF (90 mL); column chromatography (pentane/Et_2O 5:5) yielded 3,6-dimethylhept-6-en-1-ol (3.02 g, 61%) as a colorless and clear liquid: R_f 0.35 (heptane/Et_2O 5:5); ^{1}H-NMR (300 MHz, $CDCl_3$): δ 4.71–4.64 (d, J = 5.5 Hz, 2H), 3.82–3.56 (m, 2H), 2.14–1.90 (m, 2H), 1.72 (s, 3H), 1.68–1.35 (m, 5H), 1.35–1.19 (m, 1H), 0.92 (d, J = 6.4 Hz, 3H); ^{13}C-NMR (75 MHz, $CDCl_3$): δ 146.3, 109.6, 61.1, 39.9, 35.2, 35.0, 29.2, 22.5, 19.6; IR (neat): 3315, 3073, 2925, 2872, 1649, 1450, 1375, 1057, 883; HRMS (ESI-Orbitrap) *m/z* calcd for $C_9H_{18}ONa$ $[M+Na]^+$: 165.1250; found: 165.1247.

6-Methylhept-6-en-1-yl 4-chlorobenzoate (8a)

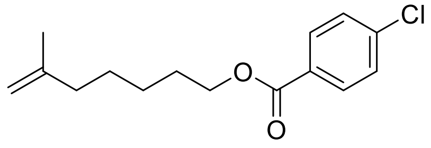

$C_{15}H_{19}ClO_2$
266.77 g/mol

According to the literature procedure[10], to a solution of 6-methylhept-6-en-1-ol (1.14 g, 11.0 mmol) and triethylamine (1.56 g, 15.4 mmol) in dry Et_2O (20 mL) was added 4-chlorobenzoyl chloride (2.50 g, 14.3 mmol) at 0 °C. The suspension was stirred at room temperature overnight. The reaction was quenched by the addition of a solution of NH_4OH (25% in H_2O, 2 mL). The mixture was diluted with water (60 mL) and extracted with Et_2O (60/30 mL). The organic layers were washed with a solution of $NaHSO_4$ (10% in H_2O, 30 mL) and water (30 mL). The organic layers were dried over Na_2SO_4 and concentrated. The crude product was purified by column chromatography (pentane/Et_2O 97:3) to yield **8a** (2.74 g, 93%) as a colorless and clear liquid: R_f 0.35 (heptane/Et_2O 97:3); ^{1}H-NMR (300 MHz, $CDCl_3$): δ 8.06–7.91 (m, 2H), 7.46–7.34 (m, 2H), 4.69 (d, J = 9.0 Hz, 2H), 4.31 (t, J = 6.7 Hz, 2H), 2.04 (t, J = 7.1 Hz, 2H), 1.78 (pent, J = 6.8 Hz, 2H), 1.71 (s, 3H), 1.57–1.37 (m, 4H); ^{13}C-NMR (75 MHz, $CDCl_3$): δ 165.8, 145.7, 139.3, 130.9, 129.0, 128.7, 109.9, 65.3, 37.6, 28.6, 27.2, 25.6, 22.4; IR (neat): 3073, 2933, 2858, 1718, 1649, 1595, 1488, 1401, 1268, 1171, 1091, 1014, 885; HRMS (ESI-Orbitrap) *m/z* calcd for $C_{15}H_{19}O_2ClNa$ $[M+Na]^+$: 289.0966; found: 289.0970.

3,6-Dimethylhept-6-en-1-yl 4-chlorobenzoate 8b

Same procedure as above starting 3,6-dimethylhept-6-en-1-ol (1.41 g, 8.0 mmol), triethylamine (1.13 g, 11.2 mmol) and 4-chlorobenzoyl chloride (1.82 g, 10.4 mmol) in dry Et_2O (15 mL); column chromatography (pentane/Et_2O 97:3) yielded **8b** (2.20 g, 98%) as a colorless and clear liquid: R_f 0.30 (heptane/Et_2O 97:3); ^{1}H-NMR (300 MHz, $CDCl_3$): δ 8.04–7.88 (m, 2H), 7.53–7.33 (m, 2H), 4.75–4.62 (m, 2H), 4.48–4.29 (m, 2H), 2.14–1.94 (m, 2H), 1.93–1.76 (m, 1H), 1.76–1.45 (m, 6H), 1.41–1.26 (m, 1H), 0.98 (d, J = 6.3 Hz, 3H); ^{13}C-NMR (75 MHz, $CDCl_3$): δ 165.8, 146.0, 139.3, 130.9, 128.9, 128.7, 109.8, 63.7, 35.5, 35.1, 34.8, 29.6, 22.5, 19.5; IR (neat): 3073, 2960, 2926, 1718, 1649, 1595, 1488, 1401, 1268, 1171, 1091, 1014, 884; HRMS (ESI-Orbitrap) *m/z* calcd for $C_{16}H_{21}O_2ClNa$ $[M+Na]^+$: 303.1122; found: 303.1121.

2-Ethylbutanenitrile

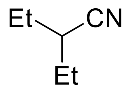

$C_6H_{11}N$
97.16 g/mol

According to the literature procedure[14], a solution of *n*-BuLi (2.5 M in hexane, 29.4 mL, 73.5 mmol) was added to a solution of diisopropylamine (10.9 mL, 77.0 mmol) in dry THF (70 mL) at <−30 °C. After stirring at −30 °C for 30 min. Butyronitrile (4.84 g, 70.0 mmol) was added dropwise at −78 °C and stirred at this temperature for 2 h. Then bromoethane (7.64 g, 70.0 mmol) was added and the mixture was allowed to stir at −78 °C for 3 h and at room temperature overnight. The reaction was quenched by the addition of water (100 mL) and the mixture was extracted with pentane (200/100 mL). The organic layers were washed with water (100 mL) and brine (100 mL), dried over Na_2SO_4, and concentrated. The crude product was purified with the CombiFlash (eluent: 0–1 min pentane/Et_2O 98:2, 1–11 min to pentane/Et_2O 93:7, 11–16 min pentane/Et_2O 93:7) to yield 2-ethylbutanenitrile (1.47 g, 22%) as a colorless and clear liquid: R_f 0.35 (heptane/Et_2O 95:5); ^{1}H-NMR (300 MHz, $CDCl_3$): δ 2.42 (p, J = 7.1 Hz, 1H), 1.64 (p, J = 7.3 Hz, 4H), 1.08 (t, J = 7.4 Hz, 6H); ^{13}C-NMR (75 MHz, $CDCl_3$): δ 122.2, 35.0, 25.2, 11.6.

2-Cyclopentyl-2-ethylbutanenitrile

Same procedure as above starting from a solution of *n*-BuLi (2.5 M in hexane, 7.0 mL, 17.4 mmol), diisoproplyamine (2.66 mL, 18.9 mmol), 2-ethylbutanenitrile (1.41 g, 14.5 mmol) and bromocyclopentane (2.81 g, 18.9 mmol) in dry THF (15 mL); column chromatography (pentane/Et_2O 95:5) yielded 2-cyclopentyl-2-ethylbutanenitrile (2.27 g, 95%) as a colorless and clear liquid: R_f 0.45 (heptane/Et_2O 95:5); ^{1}H-NMR (300 MHz, $CDCl_3$): δ 2.03–1.88 (m, 1H), 1.85–1.40 (m, 12H), 0.96 (t, J = 7.5 Hz, 6H); ^{13}C-NMR (75 MHz, $CDCl_3$): δ 123.6, 45.6, 44.3, 28.4, 26.5, 25.2, 8.3; IR (neat): 2952, 2870, 2229, 1454, 1385, 1304, 919; HRMS (ESI-Orbitrap) *m/z* calcd for $C_{11}H_{19}NNa$ $[M+Na]^+$: 188.1410; found: 188.1409.

(3-Ethylpent-1-en-3-yl)cyclopentane 11

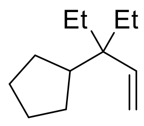

$C_{12}H_{22}$
166.31 g/mol

According to the literature procedures[15], DIBAL-H (1.0 M in hexane, 14.8 mL, 14.8 mmol) was slowly added to a mixture of 2-cyclopentyl-2-ethylbutanenitrile (2.23 g, 13.5 mmol) in dry DCM (70 mL) at −78 °C. The mixture was slowly warmed to room temperature and stirred overnight. The reaction mixture was quenched by the addition of a saturated solution of Rochelle salt (30 mL) and stirred at room temperature for 1 h. The mixture was diluted with water (100 mL) and extracted with DCM (3 × 50 mL). The organic layers were washed with water (100 mL), dried over Na_2SO_4, and concentrated. The crude product was purified by

column chromatography (pentane/Et_2O 97:3) to yield 2-cyclopentyl-2-ethylbutanal (contains ~18% of 2-cyclopentyl-2-ethylbutanenitrile according GC-FID, 1.56 g, 7.6 mmol). Methyltriphenylphosphonium bromide (4.07 g, 11.4 mmol) and potassium *tert*-butoxide (1.28 g, 11.4 mmol) in dry Et_2O (8 mL) were stirred at reflux for 30 min. 2-Cyclopentyl-2-ethylbutanal (82% purity, 1.56 g, 7.6 mmol) were slowly added and stirred at reflux for 30 min. The reaction mixture was cooled to room temperature and quenched by the addition of a saturated solution of NH_4Cl (30 mL). The mixture was extracted with pentane (60/30 mL). The organic layers were washed with water (30 mL), brine (30 mL), dried over Na_2SO_4, and concentrated. The crude product was purified by column chromatography (pentane) to yield **11** (1.23 g, 56%) as a colorless and clear liquid: R_f 0.35 (heptane/Et_2O 5:5); ^{1}H-NMR (300 MHz, $CDCl_3$): δ 5.69 (dd, J = 17.7, 11.1 Hz, 1H), 5.10 (dd, J = 11.1, 1.8 Hz, 1H), 4.89 (dd, J = 17.7, 1.8 Hz, 1H), 1.97–1.81 (m, 1H), 1.60–1.31 (m, 10H), 1.28–1.14 (m, 2H), 0.75 (t, J = 7.5 Hz, 6H); ^{13}C-NMR (75 MHz, $CDCl_3$): δ 143.4, 113.9, 45.8, 43.6, 26.5, 26.0, 25.5, 7.8; IR (neat): 3081, 2952, 2868, 1633, 1453, 1415, 1378, 1006, 910; HRMS (EI) *m/z* calcd for $C_{12}H_{22}$ $[M]^+$: 166.1716; found: 166.1712.

Synthesis of Peresters

(*R*)-4-((3*R*,5*S*,7*R*,8*R*,9*S*,10*S*,12*S*,13*R*,14*S*,17*R*)-3,7,12-Trimethoxy-10,13-dimethyl-hexadecahydro-1*H*-cyclopenta[*a*]phenanthren-17-yl)pentanoic acid

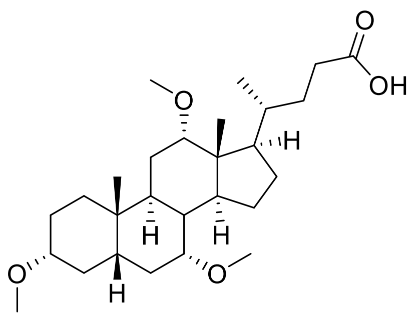

$C_{27}H_{46}O_5$
450.66 g/mol

According to the literature procedure[16], to a suspension of sodium hydride (5.00 g, 60% suspension in mineral oil, 125 mmol) in dry THF (250 mL) was added cholic acid (10.21 g, 25.0 mmol) at 0 °C. The mixture was allowed to stir at 0 °C for 10 min and then methyl iodide (8.52 g, 60 mmol) was added. The reaction mixture was stirred at room temperature for 1 d. More sodium hydride (4.00 g, 60% suspension in mineral oil, 100 mmol) followed by methyl iodide (7.10 g, 50 mmol) was added and stirred at room temperature for 1 d. These addition was repeated totally 4 times. After the last addition the reaction mixture was allowed to stir for 4 d. The reaction mixture was cooled to 0 °C and methanol (40 mL) was added dropwise. The reaction mixture was concentrated and the residue diluted with EtOAc (150 mL). The suspension was poured on ice-water (100 mL) and acidified with conc. HCl. The layers were separated and the water phase was extracted with EtOAc (2 × 75mL). The organic layers were washed with brine (75 mL), a solution of $Na_2S_2O_3$ (5% in H_2O, 75 mL), and brine (2 × 75mL). The organic layers were dried over Na_2SO_4 and concentrated. The crude product was purified by column chromatography with the CombiFlash

(0–5 min heptane/EtOAc 7:3, 5–15 min to heptane/EtOAc 4:6, 15–20 min heptane/EtOAc 4:6) to yield trimethoxy cholic acid (7.61 g, 68%) as a white solid: R_f 0.30 (heptane/EtOAc 4:6); m.p. 70–76 °C; ^{1}H-NMR (300 MHz, $CDCl_3$): *δ* 3.38–3.34 (m, 1H), 3.33 (s, 3H), 3.26 (s, 3H), 3.21 (s, 3H), 3.14 (q, *J* = 2.7 Hz, 1H), 3.07–2.94 (m, 1H), 2.47–2.34 (m, 1H), 2.33–2.16 (m, 2H), 2.14–2.00 (m, 2H), 2.00–1.60 (m, 9H), 1.60–1.11 (m, 9H), 1.09–0.86 (m, 8H), 0.66 (s, 3H); ^{13}C-NMR (75 MHz, $CDCl_3$): δ 180.3, 82.0, 80.8, 76.9, 55.8, 55.7, 55.3, 46.3, 46.1, 42.7, 42.0, 39.6, 35.3, 35.1, 34.9, 34.4, 31.0, 30.7, 28.0, 27.8, 27.3, 26.7, 23.1, 22.9, 22.0 17.4, 12.5.

2-Ethyltetradecanoic acid

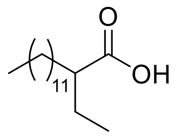

$C_{16}H_{32}O_2$
256.43 g/mol

According to the literature procedure[17], a solution of *n*-BuLi (2.5 M in hexane, 64.8 mL, 162 mmol) was added to a solution of diisoproplyamine (23.4 mL, 168 mmol) in dry THF (70 mL) at <−20 °C and stirred for 30 min at this temperature. Myristic acid (16.44 g, 72.0 mmol) in dry THF (70 mL) was added dropwise at <−20°C and stirred at 0 °C for 15 min. Then DMPU (8.71 mL, 72.0 mmol) was added and stirred at 0 °C for 30 min. Iodoethane (13.0 mL, 162 mmol) was added at <10 °C and the mixture was allowed to stir at room temperature overnight. The reaction mixture was diluted with pentane (400 mL) and acidified with a 2 M aqueous solution of HCl. The phases were separated and the aqueous layer was extracted with pentane (100 mL). The organic layers were washed with water (200 mL), brine (200 mL), dried over Na_2SO_4, and concentrated. The crude product was purified by column chromatography (heptane/EtOAc/AcOH 92:7:1) to yield 2-ethyltetradecanoic acid (12.31 g, 67%) as a colorless and clear oil: R_f 0.25 (heptane/EtOAc/AcOH 92:7:1); ^{1}H-NMR 300 MHz, $CDCl_3$): *δ* 2.28 (tt, *J* = 8.5, 5.5 Hz, 1H), 1.78–1.40 (m, 5H), 1.39–1.18 (m, 19H), 0.94 (t, *J* = 7.4 Hz, 3H), 0.88 (t, *J* = 6.7 Hz, 3H); ^{13}C-NMR (75 MHz, $CDCl_3$): δ 183.1, 47.2, 32.0, 31.8, 29.70, 29.68 (2C), 29.63, 29.61, 29.5, 29.4, 27.4, 25.2, 22.7, 14.1, 11.8.

2,2-Dimethyltetradecanoic acid

Same procedure as above starting from *n*-BuLi (2.5 M in hexane, 32.4 mL, 81.0 mmol), diisopropylamine (11.7 mL, 82.8 mmol), isobutyric acid (3.17 g, 36 mmol), DMPU (4.35 mL, 36 mmol) and 1-iodododecane (23.99 g, 81.0 mmol); column chromatography (heptane/AcOH 99:1 to heptane/EtOAc/AcOH 94:5:1) yielded 2,2-dimethyltetradeconoic acid (8.10 g, 88%) as a white solid: R_f 0.20 (heptane/EtOAc/AcOH 94:5:1); m.p. 41–42 °C; ^{1}H-NMR (300 MHz, $CDCl_3$): *δ* 1.59–1.47 (m, 2H), 1.35–1.21 (m, 20H), 1.19 (s, 6H), 0.88 (t, *J* = 6.6 Hz, 3H); ^{13}C-NMR (75 MHz, $CDCl_3$): *δ* 185.1, 42.2, 40.6, 31.9, 30.1, 29.7 (4C), 29.5, 29.4, 24.9 (2C), 24.9, 22.7, 14.1.

(4*R*)-2-Methyl-4-((3*R*,5*S*,7*R*,8*R*,9*S*,10*S*,12*S*,13*R*,14*S*,17*R*)-3,7,12-trimethoxy-10,13-dimethylhexadecahydro-1*H*-cyclopenta[*a*]phenanthren-17-yl)pentanoic acid

Same procedure as above starting from *n*-BuLi (2.5 M in hexane, 7.20 mL, 18.0 mmol), diisopropylamine (2.60 mL, 18.4 mmol), trimethoxy cholic acid (3.72 g, 8.0 mmol), DMPU (0.97 mL, 8.0 mmol) and iodomethane (1.12 mL, 18.0 mmol); column chromatography with the CombiFlash (0–3 min heptane/EtOAc 9:1, 3–13 min to heptane/EtOAc 6:4, 13–20 min heptane/EtOAc 6:4) yielded two diastereomers of α-methylated trimethoxy cholic acid.

Diastereomer A: white solid; yield 1.22 g (33%); R_f 0.35 (heptane/EtOAc 6:4); m.p. 87–92 °C; ^{1}H-NMR (300 MHz, $CDCl_3$): δ 3.40–3.35 (m, 1H), 3.33 (s, 3H), 3.25 (s, 3H), 3.21 (s, 3H), 3.14 (q, J = 2.1 Hz, 1H), 3.08–2.93 (m, 1H), 2.67–2.53 (m, 1 H), 2.19 (dd, J = 24.9, 12.8 Hz, 1H), 2.12–1.97 (m, 2H), 1.94–1.40 (m, 11H), 1.40–1.11 (m, 8H), 1.09–0.82 (m, 9H), 0.64 (s, 3H); ^{13}C-NMR (75 MHz, $CDCl_3$): δ 183.2, 82.0, 80.8, 77.0, 55.8, 55.7, 55.3, 47.2, 46.2, 42.7, 42.0, 40.7, 39.6, 37.2, 35.3, 34.9, 34.42, 34.37, 28.0, 27.8, 27.4, 26.7, 23.2, 22.9, 22.0, 18.9, 17.7, 12.5.

Diastereomer B: white solid; yield 1.69 g (45%); R_f 0.25 (heptane/EtOAc 6:4); m.p. 87–93 °C; ^{1}H-NMR (300 MHz, $CDCl_3$): δ 3.41–3.36 (m, 1H), 3.34 (s, 3H), 3.26 (s, 3H), 3.21 (s, 3H), 3.14 (q, J = 2.6 Hz, 1H), 3.08–2.95 (m, 1H), 2.58–2.39 (m, 1H), 2.20 (dd, J = 24.8, 12.9 Hz, 1H), 2.13–2.00 (m, 2H), 1.99–1.60 (m, 8H), 1.58–1.10 (m, 12H), 1.08–0.86 (m, 8H), 0.67 (s, 3H); ^{13}C-NMR (75 MHz, $CDCl_3$): δ 183.5, 82.0, 80.8, 76.9, 55.8, 55.7, 55.3, 47.2, 46.3, 42.7, 42.0, 39.6, 39.4, 36.8, 35.3, 34.9, 34.3, 33.5, 28.0, 27.8, 27.7, 26.7, 23.2, 22.9, 22.0, 17.3, 15.9, 12.5; IR (neat): 2929, 2867, 2818, 1732, 1703, 1454, 1370, 1233, 1183, 1099, 1087, 942; HRMS (ESI-Orbitrap) *m/z* calcd for $C_{28}H_{49}O_5$ $[M+H]^+$: 465.3575; found: 465.3563.

***tert*-Butyl 2-ethyltetradecaneperoxoate (13a)**

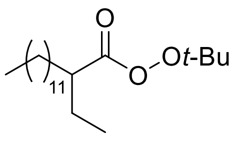

$C_{20}H_{40}O_3$
328.54 g/mol

According to the literature procedure[16], to a solution of the 2-ethyltetradecanoic acid (3.08 g, 12.0 mmol) in DCM (120 mL) was added DMAP (0.15 g, 1.2 mmol) followed by a solution of *tert*-butyl hydroperoxide (70% in H_2O, 1.73 mL, 12.6 mmol) at room temperature. The reaction mixture was cooled to 0 °C and a solution DCC (2.72 g, 13.2 mmol) in DCM (60 mL) was added. The mixture was stirred at 0 °C for 30 min and at room temperature overnight. The reaction mixture was filtered through a small plug of silica to remove solids and polar impurities. The crude product was purified by column chromatography (pentane/Et_2O 95:5) to yield **13a** (2.95 g, 75%) as a colorless and clear oil: R_f 0.25 (heptane/Et_2O 95:5); ^{1}H-NMR (300 MHz, $CDCl_3$): δ 2.34–2.17 (m, 1H), 1.77–1.39 (m, 5H),

1.37–1.20 (m, 28H), 1.02–0.78 (m, 6H); ^{13}C-NMR (75 MHz, $CDCl_3$): δ 173.6, 83.0, 45.1, 32.2, 31.9, 29.67, 29.65 (2C), 129.58, 129.5 (2C), 29.4, 27.4, 26.3 (3C), 25.7, 22.7, 14.1, 11.9.

tert-Butyl (4_R_)-2-methyl-4-((3_R_,5_S_,7_R_,9_S_,10_S_,12_S_,13_R_,14_S_,17_R_)-3,7,12-trimethoxy-10,13-dimethylhexadecahydro-1H-cyclopenta[a]phenanthren-17-yl)pentaneperoxoate (13c)

Same procedure as above starting from α-methylated trimethoxy cholic acid diastereomer A (1.39 g, 3.0 mmol), DMAP (37 mg, 0.3 mmol), *tert*-butyl hydroperoxide (70% in H_2O, 0.43 mL, 3.15 mmol) and DCC (0.68 g, 3.3 mmol); column chromatography (pentane/Et_2O 7:3) **13c** (1.21 g, 75%) as a white solid: R_f 0.30 (heptane/Et_2O 7:3); 106–108 °C (decomposition); ^{1}H-NMR (300 MHz, C_6H_6): *δ* 3.26 (s, 3H), 3.29–3.22 (m, 1H), 3.08 (s, 3H), 3.00 (s, 3H), 3.03–2.90 (m. 2H), 2.59–2.52 (m, 1H), 2.51–2.35 (m, 2H), 2.30 (td, *J* = 12.5, 3.9 Hz, 1H), 2.16–1.96 (m, 2H), 1.93–1.35 (m, 12H), 1.22 (s, 9H), 1.25–1.12 (m, 1H), 1.10–0.97 (m, 8H), 0.97–0.86 (m, 2H), 0.83 (s, 3H), 0.63 (s, 3H); ^{13}C-NMR (75 MHz, C_6H_6): *δ* 173.6, 82.5, 82.0, 80.9, 77.3, 55.7, 55.32, 55.29, 47.5, 46.6, 42.9, 42.4, 41.1, 40.1, 35.7, 35.2 (2C), 35.0, 34.7, 28.3, 28.1, 27.7, 27.5, 26.3 (3C), 23.6, 23.2, 22.3, 19.3, 17.8, 12.7.

tert-Butyl 2,2-dimethyltetradecaneperoxoate (13b)

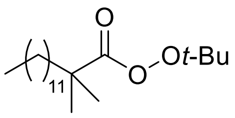

$C_{20}H_{40}O_3$
328.54 g/mol

According to the literature procedures[18,19], 2,2-Dimethyltetradecanoic acid (2.05 g, 8.0 mmol) was dissolved in thionylchloride (8 mL) and heated to reflux for 1 h. The mixture was cooled to room temperature and dried under high vacuum. The residue was dissolved in pentane (5 mL) and *tert*-butyl hydroperoxide (5.5 mol/L in decane, 2.47 mL, 13.6 mmol) was added. The mixture was cooled to 0 °C and pyridine (0.77 mL, 9.6 mmol) was added slowly. The reaction mixture was allowed to stir at room temperature for 6 h. The mixture was quenched by the addition of a solution of ice cold aqueous 1 M HCl (20 mL) and extracted with pentane (100/50 mL). The organic layers were washed with brine (3 × 100 mL), dried over Na_2SO_4, and concentrated. The crude product was purified by column chromatography (pentane/Et_2O 96:4) to yield **13b** (1.74 g, 66%) as a colorless and clear oil: R_f 0.30 (heptane/Et_2O 96:4); ^{1}H-NMR (300 MHz, $CDCl_3$): *δ* 1.60–1.50 (m, 2H), 1.33 (s, 9H), 1.32–1.22 (m, 20H), 1.22 (s, 6H), 0.88 (t, *J* = 6.8 Hz, 3H); ^{13}C-NMR (75 MHz, $CDCl_3$): δ 174.6, 83.2, 42.6, 41.0, 31.9, 30.1, 29.7, 29.65 (2C), 29.59, 29.5, 29.4, 26.2 (3C), 25.2 (2C), 24.9, 22.7, 14.1; IR (neat): 2924, 2853, 1767, 1468, 1389, 1365, 1245, 1193, 1085, 857; HRMS (ESI-Orbitrap) *m/z* calcd for $C_{20}H_{40}O_3Na$ [M+Na]$^+$: 351.2870; found: 351.2861.

Synthesis of Amides and Sulfonamides

General Procedure 1 (GP1): Synthesis of amides and sulfonamides (S1 and S2)

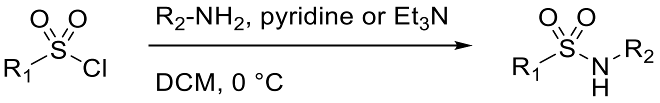

Lit.: B. Niu, P. Xie, Z. Bian, W. Zhao, M. Zhang, Y. Zhou, L. Feng, C. U. Pittman Jr, A. Zhou, *Synlett,* 2015*, 26,* 635*.*

The amine (1.0–1.2 equiv) was added to a mixture of pyridine or Et_3N (3.16 g, 40.0 mmol) in dry DCM (16 mL) and cooled to 0 °C. The sulfonyl chloride (20.0 mmol) dissolved in dry DCM (12 mL) was added and the mixture was allowed to stir at 0 °C or room temperature until full conversion was monitored by TLC. The mixture was diluted with Et_2O/pentane 1:1 (80 mL) and extracted with a 1 M HCl solution (40 mL). The layers were separated and the aqueous phase was extracted with Et_2O/pentane 1:1 (40 mL). The organic layers were washed with a 1 M aqueous solution of HCl (2 × 40 mL) and water (3 × 40 mL), dried over Na_2SO_4, and concentrated. The crude product was purified by column chromatography or recrystallization.

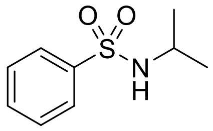

$C_9H_{13}NO_2S$
199.27 g/mol

***N*-Isopropylbenzenesulfonamide (S1-a).** Prepared according GP1 from isopropylamine (3.55 g, 60.0 mmol), triethylamine (10.12 g, 100 mmol) and benzenesulfonyl chloride (8.83 g, 50.0 mmol) at 0 °C for 1 h; column chromatography (pentane/Et_2O 4:6) yielded **S1-a** (9.90 g, 99%) as a slightly yellow and clear liquid: R_f 0.45 (heptane/Et_2O 4:6); ^{1}H-NMR (300 MHz, $CDCl_3$): δ 7.95–7.87 (m, 2H), 7.61–7.47 (m, 3H), 4.69 (brd, J = 6.1 Hz, 1H), 3.47 (oct, J = 6.5 Hz, 1H), 1.08 (d, J = 6.5 Hz, 6H); ^{13}C-NMR (75 MHz, $CDCl_3$): δ 141.1, 132.5, 129.1, 127.0, 46.1, 23.7.

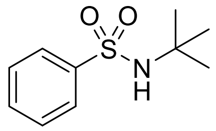

$C_{10}H_{15}NO_2S$
213.30 g/mol

***N*-(*tert*-Butyl)benzenesulfonamide (S1-b).** Prepared according GP1 from *tert*-butylamine (2.63 g, 36.0 mmol), triethylamine (6.07 g, 60.0 mmol) and benzenesulfonyl chloride (5.30 g, 30.0 mmol) at 0 °C for 2 h and at room temperature for 2 h; column chromatography (pentane/Et_2O 6:4) yielded **S1-b** (6.24 g, 98%) as a white solid: R_f 0.25 (heptane/Et_2O 6:4); m.p. 78–79 °C; ^{1}H-NMR (300 MHz, $CDCl_3$): δ 8.01–7.84 (m, 2H), 7.64–7.39 (m, 3H), 5.07 (s, 1H), 1.22 (s, 9H); ^{13}C-NMR (75 MHz, $CDCl_3$): δ 143.5, 132.1, 128.9, 126.9, 54.6, 30.1.

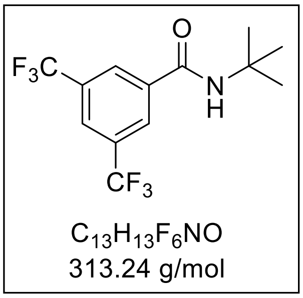

***N*-(*tert*-Butyl)-3,5-bis(trifluoromethyl)benzamide (S1-c).** Prepared according GP1 from *tert*-butylamine (2.63 g, 36.0 mmol), triethylamine (6.07 g, 60.0 mmol) and 3,5-bis(trifluoromethyl)benzoyl chloride (8.30 g, 30.0 mmol) at 0 °C for 1 h; recrystallization from toluene yielded **S1-c** (8.53 g, 91%) as a white solid: R_f 0.45 (heptane/Et_2O 8:2); m.p. 144–145 °C; ^{1}H-NMR (300 MHz, $CDCl_3$): δ 8.14 (s, 2H), 7.95 (s, 1H), 6.15 (s, 1H), 1.50 (s, 9H); ^{13}C-NMR (75 MHz, $CDCl_3$): δ 164.0, 138.0, 132.1 (q, J = 33.8 Hz), 127.2 (q, J = 2.8 Hz), 124.6 (spt, J = 3.8 Hz), 123.0 (q, J = 272.9 Hz), 52.5, 28.7.

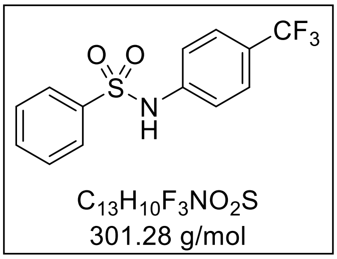

***N*-(4-(Trifluoromethyl)phenyl)benzenesulfonamide (S2-a).** Prepared according GP1 from 4-(trifluoromethyl)aniline (13.97 g, 86.7 mmol), pyridine (13.45 g, 170 mmol) and benzenesulfonyl chloride (15.01 g, 85.0 mmol) at 0 °C for 1 h; recrystallization from heptane yielded **S2-a** (24.62 g, 96%) as a white solid: R_f 0.30 (heptane/EtOAc 7:3); m.p. 100–101 °C; ^{1}H-NMR (300 MHz, $CDCl_3$): δ 7.92–7.85 (m, 2H), 7.80 (s, 1H), 7.61–7.53 (m, 1H), 7.52–7.42 (m, 4H), 7.22 (d, J = 8.5 Hz, 2H); ^{13}C-NMR (75 MHz, $CDCl_3$): δ 139.8 (q, J = 1.2 Hz), 138.6, 133.6, 129.4, 127.2, 126.9 (q, J = 32.9 Hz), 126.7(q, J = 3.8 Hz), 123.9 (q, J = 271.7 Hz), 119.8.

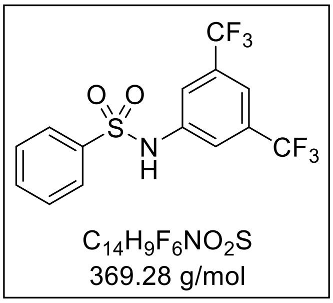

***N*-(3,5-Bis(trifluoromethyl)phenyl)benzenesulfonamide (S2-b).** Prepared according GP1 from 3,5-bis(trifluoromethyl)aniline (7.01 g, 30.6 mmol), pyridine (4.75 g, 60.0 mmol) and benzenesulfonyl chloride (5.30 g, 30.0 mmol) at 0 °C for 1 h; column chromatography (heptane/EtOAc 8:2) yielded **S2-b** (10.31 g, 93%) as a white solid: R_f 0.35 (heptane/EtOAc 8:2); m.p. 108 °C; ^{1}H-NMR (300 MHz, $CDCl_3$): δ 8.14 (s, 1H), 7.90 (d, J = 7.4 Hz, 2H), 7.71–7.44 (m, 6H); ^{13}C-NMR (75 MHz, $CDCl_3$): δ 138.4, 138.0, 134.1, 132.9 (q, J = 33.8 Hz), 129.6, 127.3, 122.8 (q, J = 272.9 Hz), 120.1 (q, J = 3.1 Hz), 118.4 (spt, J = 3.8 Hz); IR (neat): 3259, 1622, 1509, 1470, 1422, 1376, 1334, 1275, 1125, 1089, 975, 878; HRMS (ESI-Orbitrap) *m/z* calcd for $C_{14}H_{10}O_2NF_6S$ $[M+H]^+$: 370.0331; found: 370.0324.

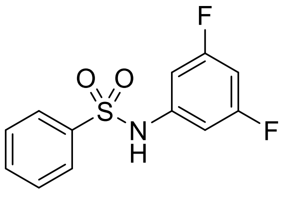

$C_{12}H_9F_2NO_2S$
269.27 g/mol

***N*-(3,5-Difluorophenyl)benzenesulfonamide (S2-c).** Prepared according GP1 from 3,5-bis(trifluoromethyl)aniline (2.63 g, 20.4 mmol), pyridine (3.16 g, 40.0 mmol) and benzenesulfonyl chloride (3.53 g, 20.0 mmol) at 0 °C for 1 h; column chromatography (heptane/EtOAc 7:3) yielded **S2-c** (5.35 g, 99%) as a white solid: R_f 0.45 (heptane/EtOAc 7:3); m.p. 131 °C; ^{1}H-NMR (300 MHz, $CDCl_3$): δ 7.93–7.84 (m, 2H), 7.66 (s, 1H), 7.63–7.57 (m, 1H), 7.56–7.45 (m, 2H), 6.72–6.62 (m, 2H), 6.51 (tt, J = 8.9, 2.2 Hz, 1H); ^{13}C-NMR (75 MHz, $CDCl_3$): δ 163.3 (dd, J = 248.4, 14.5 Hz), 139.0 (t, J = 12.9 Hz), 138.4, 133.7, 129.4, 127.2, 103.6–103.1 (m), 100.3 (t, J = 25.5 Hz); IR (neat): 3291, 1626, 1609 1496, 1477, 1449, 1409, 1330, 1307, 1146, 1117, 1091, 1025, 994; HRMS (ESI-Orbitrap) *m/z* calcd for $C_{12}H_{10}O_2NF_2S$ [M+H]$^+$: 270.0395; found: 270.0393.

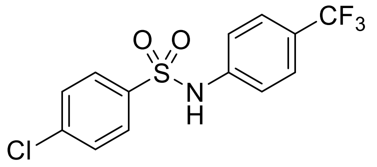

$C_{13}H_9ClF_3NO_2S$
335.73 g/mol

4-Chloro-*N*-(4-(trifluoromethyl)phenyl)benzenesulfonamide (S2-d). Prepared according GP1 from 4-(trifluoromethyl)aniline (2.47 g, 15.3 mmol), pyridine (2.37 g, 30.0 mmol) and 4-chlorobenzenesulfonyl chloride (3.17 g, 15.0 mmol) at 0 °C for 1 h; column chromatography (heptane/EtOAc 7:3) yielded **S2-d** (4.98 g, 99%) as a white solid: R_f 0.30 (heptane/EtOAc 7:3); m.p. 94–95 °C; ^{1}H-NMR (300 MHz, $CDCl_3$): δ 7.81 (s, 1H), 7.81 (d, J = 8.5 Hz, 2H), 7.50 (d, J = 8.5 Hz, 2H), 7.44 (d, J = 8.5 Hz, 2H), 7.22 (d, J = 8.5 Hz, 2H); ^{13}C-NMR (75 MHz, $CDCl_3$): δ 140.3, 139.5 (q, J = 1.2 Hz), 137.0, 129.8, 128.7, 127.2 (q, J = 30.0 Hz), 126.8 (q, J = 3.7 Hz), 123.8 (q, J = 271.9 Hz), 120.0; IR (neat): 3267, 1616, 1589, 1573, 1517, 1477, 1443, 1399, 1322, 1227, 1156, 1103, 1095, 1066, 1020, 1013, 904; HRMS (ESI-Orbitrap) *m/z* calcd for $C_{13}H_8O_2NClF_3S$ [M−H]$^-$: 333.9922; found: 333.9920.

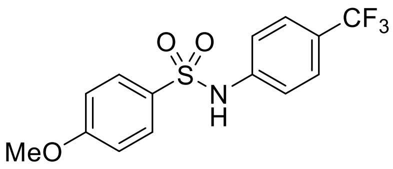

$C_{14}H_{12}F_3NO_3S$
331.31 g/mol

4-Methoxy-*N*-(4-(trifluoromethyl)phenyl)benzenesulfonamide (S2-e). Prepared according GP1 from 4-(trifluoromethyl)aniline (3.29 g, 20.4 mmol), pyridine (3.16 g, 40.0 mmol) and 4-methoxybenzenesulfonyl chloride (4.13 g, 20.0 mmol) at 0 °C for 1 h; column chromatography (heptane/EtOAc 5:5) yielded **S2-e** (6.55 g, 99%) as a white solid: R_f 0.50 (heptane/EtOAc 5:5); m.p. 116–117 °C; ^{1}H-NMR (300 MHz, $CDCl_3$): δ 7.85 (s, 1H), 7.84–7.75 (m, 2H), 7.47 (d, J = 8.5 Hz, 2H), 7.21 (d, J = 8.5 Hz, 2H), 7.00–6.84 (m, 2H), 3.82 (s, 3H); ^{13}C-NMR (75 MHz, $CDCl_3$): δ 163.6, 140.2 (q, J = 1.2 Hz), 130.0, 129.5, 126.6 (q,

J = 3.8 Hz), 126.5 (q, *J* = 32.8 Hz), 124.0 (q, *J* = 271.6 Hz), 119.5, 114.5, 55.6; IR (neat): 3298, 3239, 1616, 1593, 1575, 1519, 1497, 1465, 1320, 1267, 1148, 1114, 1068, 1015, 913; HRMS (ESI-Orbitrap) *m/z* calcd for $C_{14}H_{11}O_3NF_3S$ $[M{-}H]^-$: 330.0417; found: 330.0407.

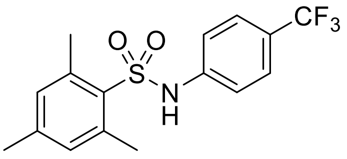

$C_{16}H_{16}F_3NO_2S$
343.36 g/mol

2,4,6-Trimethyl-*N*-(4-(trifluoromethyl)phenyl)benzene-sulfonamide (S2-f). Prepared according GP1 from 4-(trifluoromethyl)aniline (17.26 g, 107 mmol), pyridine (16.61 g, 210 mmol) and 2-mesitylenesulfonyl chloride (22.96 g, 105 mmol) at room temperature for 6 h; recrystallization from heptane yielded **S2-f** (35.07 g, 97%) as a white solid: R_f 0.50 (heptane/EtOAc 7:3); m.p. 106–107 °C; ^{1}H-NMR (300 MHz, $CDCl_3$): *δ* 7.50 (s, 1H), 7.45 (d, *J* = 8.5 Hz, 2H), 7.03 (d, *J* = 8.5 Hz, 2H), 6.95 (s, 2H), 2.67 (s, 6H), 2.28 (s, 3H); ^{13}C-NMR (75 MHz, $CDCl_3$): *δ* 143.4, 140.2 (q, *J* = 1.1 Hz), 139.4, 133.0, 132.4, 126.6 (q, *J* = 3.7 Hz), 126.0 (q, *J* = 32.9 Hz), 124.0 (q, *J* = 271.7 Hz), 118.6, 23.0, 21.0; IR (neat): 3274, 1619, 1523, 1466, 1399, 1325, 1299, 1234, 1145, 1114, 1073, 1054, 1015, 911; HRMS (ESI-Orbitrap) *m/z* calcd for $C_{16}H_{17}O_2NF_3S$ $[M{+}H]^+$: 344.0927; found: 344.0918.

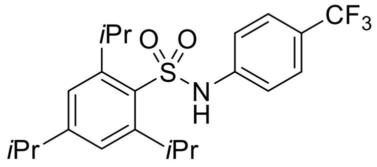

$C_{22}H_{28}F_3NO_2S$
427.53 g/mol

2,4,6-Triisopropyl-*N*-(4-(trifluoromethyl)phenyl)benzene-sulfonamide (S2-g). Prepared according GP1 from 4-(trifluoromethyl)aniline (3.29 g, 20.4 mmol), pyridine (3.16 g, 40.0 mmol) and 2,4,6-triisopropylbenzenesulfonylchloride (6.06 g, 20.0 mmol) at reflux for 3 h; column chromatography (heptane/EtOAc 8:2) yielded **S2-g** (6.18 g, 72%) as a white solid: R_f 0.30 (heptane/EtOAc 8:2); m.p. 175–176 °C; ^{1}H-NMR (300 MHz, $CDCl_3$): *δ* 7.62 (s, 1H), 7.46 (d, *J* = 8.5 Hz, 2H), 7.17 (s, 2H), 7.08 (d, *J* = 8.5 Hz, 2H), 4.20 (sept, *J* = 6.8 Hz, 2H), 2.89 (sept, *J* = 6.8 Hz, 1H), 1.24 (d, *J* = 6.8 Hz, 18H); ^{13}C-NMR (75 MHz, $CDCl_3$): *δ* 153.8, 150.7, 140.4 (q, *J* = 1.1 Hz), 131.8, 126.5 (q, *J* = 3.7 Hz), 126.3 (q, *J* = 32.9 Hz), 124.2, 124.0 (q, *J* = 271.5 Hz), 119.7, 34.1, 29.9, 24.7, 23.5; IR (neat): 3245, 2965, 2931, 2874, 1617, 1598, 1523, 1476, 1320, 1304, 1244, 1159, 1147, 1112, 1070, 912; HRMS (ESI-Orbitrap) *m/z* calcd for $C_{22}H_{27}O_2NF_3S$ $[M{-}H]^-$: 426,1720; found: 426.1704.

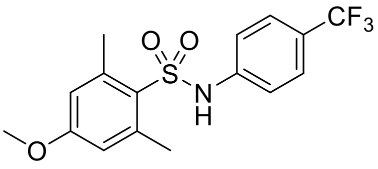

$C_{16}H_{16}F_3NO_3S$
359.36 g/mol

4-Methoxy-2,6-dimethyl-N-(4-(trifluoromethyl)phenyl)-benzenesulfonamide (S2-h). Prepared according GP1 from 4-(trifluoromethyl)aniline (2.96 g, 18.4 mmol), pyridine (2.85 g, 36.0 mmol) and 4-methoxy-2,6-dimethylbenzenesulfonyl chloride (4.22 g, 18.0 mmol) at room temperature for 4 h; column chromatography (heptane/EtOAc 6:4) yielded **S2-h** (6.13 g, 95%) as a white solid: R_f 0.35 (heptane/EtOAc 6:4); m.p. 135–136 °C; ^{1}H-NMR (300 MHz, $CDCl_3$): *δ* 7.70 (s, 1H), 7.45 (d, *J* = 8.5 Hz, 2H), 7.03 (d, *J* = 8.5 Hz, 2H), 6.64 (s, 2H), 3.80 (s, 3H), 2.69 (s, 6H); ^{13}C-NMR (75 MHz, $CDCl_3$): *δ* 161.9, 142.2, 140.4 (q, *J* = 1.1 Hz), 127.7, 126.6 (q, *J* = 3.7 Hz), 125.9 (q, *J* = 32.8 Hz), 124.0 (q, *J* = 271.6 Hz), 118.5, 116.6, 55.3, 23.5; IR (neat): 3258, 2947, 1615, 1595, 1520, 1470, 1401, 1316, 1298, 1234, 1148, 1109, 1088, 1070, 1012, 912; HRMS (ESI-Orbitrap) *m/z* calcd for $C_{16}H_{16}O_3NF_3SNa$ $[M+Na]^+$: 382.0695; found: 382.0698.

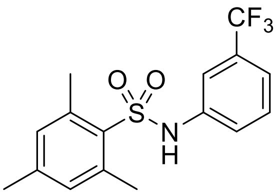

$C_{16}H_{16}F_3NO_2S$
343.36 g/mol

2,4,6-Trimethyl-*N*-(3-(trifluoromethyl)phenyl)benzenesulfon-amide (S2-i). Prepared according GP1 from 3-(trifluoromethyl)aniline (2.47 g, 15.3 mmol), pyridine (2.37 g, 30.0 mmol) and 2-mesitylene sulfonyl chloride (3.28 g, 15.0 mmol) at room temperature for 6 h; crystallization from heptane/EtOAc (7:3) yielded **S2-i** (4.82 g, 94%) as a white solid: R_f 0.40 (heptane/EtOAc 7:3); m.p. 114–115 °C; ^{1}H-NMR (300 MHz, $CDCl_3$): *δ* 7.53 (s, 1H), 7.37–7.26 (m, 2H), 7.24–7.17 (m, 1H); 7.17–7.10 (m, 1H), 6.94 (s, 2H), 2.65 (s, 6H), 2.27 (s, 3H); ^{13}C-NMR (75 MHz, $CDCl_3$): *δ* 143.3, 139.5, 137.5, 132.8, 132.2, 131.7 (q, *J* = 32.7 Hz), 129.9, 123.6 (q, *J* = 272.5 Hz), 123.2 (q, *J* = 0.8 Hz), 121.2 (q, *J* = 3.7 Hz), 116.6 (q, *J* = 3.9 Hz), 22.9, 21.0; IR (neat): 3247, 2983, 1601, 1469, 1404, 1324, 1147, 1115, 1099, 1072, 1052, 932, 891; HRMS (ESI-Orbitrap) *m/z* calcd for $C_{16}H_{15}O_2NF_3S$ $[M-H]^-$: 342.0781; found: 342.0775.

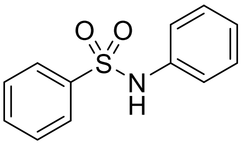

$C_{12}H_{11}NO_2S$
233.29 g/mol

***N*-Phenylbenzenesulfonamide (S2-j).** Prepared according GP1 from aniline (3.35 g, 36.0 mmol), triethylamine (6.07 g, 60.0 mmol) and benzenesulfonyl chloride (5.30 g, 30.0 mmol) at 0 °C for 1 h; column chromatography (pentane/Et_2O 7:3 to 5:5) yielded **S2-j** (5.84 g, 83%) as a white solid: R_f 0.30 (heptane/Et_2O 5:5); m.p. 109 °C; ^{1}H-NMR (300 MHz, $CDCl_3$): *δ* 7.88–7.76 (m, 2H), 7.57–7.47 (m, 1H), 7.46–7.34 (m, 3H), 7.28–7.14 (m, 2H), 7.13–7.00 (m, 3H); ^{13}C-NMR (75 MHz, $CDCl_3$): *δ* 138.9, 136.4, 133.0, 129.3, 129.0, 127.2, 125.4, 121.6.

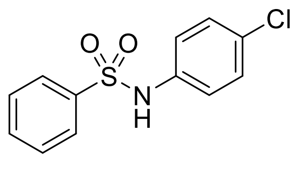

$C_{12}H_{10}ClNO_2S$
267.73 g/mol

***N*-(4-Chlorophenyl)benzenesulfonamide (S2-k).** Prepared according GP1 from 4-chloroaniline (6.51 g, 51.0 mmol), pyridine (7.91 g, 100 mmol) and benzenesulfonyl chloride (8.83 g, 50.0 mmol) at 0 °C for 1 h; column chromatography (heptane/EtOAc 8:2) yielded **S2-k** (12.98 g, 97%) as a white solid: R_f 0.25 (heptane/EtOAc 8:2); m.p. 122–123 °C; ^{1}H-NMR (300 MHz, $CDCl_3$): δ 7.85–7.77 (m, 2H), 7.59–7.49 (m, 1H), 7.51 (s, 1H), 7.49–7.39 (m, 2H), 7.21–7.14 (m, 2H), 7.08–7.01 (m, 2H); ^{13}C-NMR (75 MHz, $CDCl_3$): δ 138.5, 135.0, 133.3, 131.0, 129.4, 129.2, 127.2, 123.0.

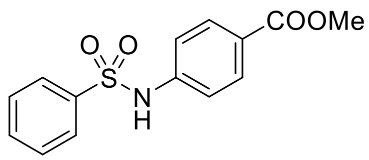

$C_{14}H_{13}NO_4S$
291.32 g/mol

Methyl 4-(phenylsulfonamido)benzoate (S2-l). Prepared according GP1 from methyl 4-aminobenzoate (4.31 g, 30.0 mmol), pyridine (4.75 g, 60.0 mmol) and benzenesulfonyl chloride (5.30 g, 30.0 mmol) at 0 °C for 1 h; quenching by addition of a NH_4OH solution (25% in H_2O, 3 mL), dilution with H_2O (75 mL) and extracted with diethylether/pentane 1:1(150/75 mL); column chromatography (heptane/EtOAc 7:3) yielded **S2-l** (2.61 g, 31%) as a slightly red solid: R_f 0.25 (heptane/EtOAc 7:3); m.p. 156–157 °C; ^{1}H-NMR (300 MHz, $CDCl_3$): δ 7.95–7.83 (m, 4H), 7.77 (s, 1H), 7.58–7.51 (m, 1H), 7.45 (t, J = 7.5 Hz, 2H), 7.17 (d, J = 8.7 Hz, 2H), 3.87 (s, 3H); ^{13}C-NMR (75 MHz, $CDCl_3$): δ 166.5, 141.0, 138.7, 133.4, 131.1, 129.3, 127.2, 126.2, 119.1, 52.2.

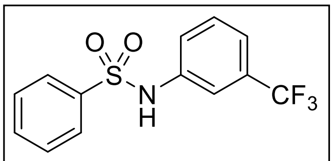

$C_{13}H_{10}F_3NO_2S$
301.28 g/mol

***N*-(3-(Trifluoromethyl)phenyl)benzenesulfonamide (S2-m).** Prepared according GP1 from 3-(trifluoromethyl)aniline (3.29 g, 20.4 mmol), pyridine (3.16 g, 40.0 mmol) and benzenesulfonyl chloride (3.53 g, 20.0 mmol) at 0 °C for 1 h; column chromatography (heptane/EtOAc 7:4) yielded **S2-m** (5.99 g, 99%) as a white solid: R_f 0.40 (heptane/EtOAc 7:3); m.p. 91 °C; ^{1}H-NMR (300 MHz, $CDCl_3$): δ 7.90–7.78 (m, 2H), 7.65 (s, 1H), 7.60–7.52 (m, 1H), 7.52–7.41 (m, 2H), 7.39–7.27 (m, 4H); ^{13}C-NMR (75 MHz, $CDCl_3$): δ 138.5, 137.2, 133.5, 131.8 (q, J = 32.8 Hz), 130.0, 129.3, 127.2, 124.2 (q, J = 1.0 Hz), 123.5 (q, J = 272.6 Hz), 121.9 (q, J = 3.8 Hz), 117.8 (q, J = 3.9 Hz); IR (neat): 3252, 1599, 1500, 1448, 1408, 1326, 1266, 1159, 1090, 1069, 924; HRMS (ESI-Orbitrap) *m/z* calcd for $C_{13}H_{11}O_2NF_3S$ $[M+H]^+$: 302.0457; found: 302.0467.

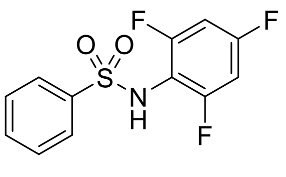

$C_{12}H_8F_3NO_2S$
287.26 g/mol

***N*-(2,4,6-Trifluorophenyl)benzenesulfonamide (S2-n).** Prepared according GP1 from 2,4,6-trifluoroaniline (3.00 g, 20.4 mmol), pyridine (3.16 g, 40.0 mmol) and benzenesulfonyl chloride (3.53 g, 20.0 mmol) at 0 °C for 1 h; column chromatography (heptane/EtOAc 7:3) yielded **S2-n** (4.77 g, 83%) as a white solid: R_f 0.40 (heptane/EtOAc 7:3); m.p. 124 °C; ^{1}H-NMR (300 MHz, $CDCl_3$): δ 7.94–7.76 (m, 2H), 7.61 (t, J = 7.4 Hz, 1H), 7.49 (t, J = 7.7 Hz, 2H), 6.75–6.55 (m, 2H), 6.45 (s, 1H); ^{13}C-NMR (75 MHz, $CDCl_3$): δ 161.4 (dt, J = 251.7, 14.7 Hz), 159.5 (ddd, J = 254.3, 15.2, 6.3 Hz), 139.2, 133.4, 129.0, 127.3, 109.7 (td, J = 16.7, 5.2 Hz), 100.9 (tdd, J = 25.8, 3.5, 0.9 Hz); IR (neat): 3250, 1635, 1605, 1510, 1450, 1340, 1231, 1168, 1163, 1127, 1037, 1001, 895; HRMS (ESI-Orbitrap) *m/z* calcd for $C_{12}H_9O_2NF_3S$ $[M+H]^+$: 288.0301; found: 288.0300.

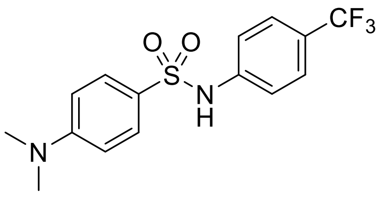

$C_{15}H_{15}F_3N_2O_2S$
344.3522

4-(Dimethylamino)-*N*-(4-(trifluoromethyl)phenyl)-benzenesulfonamide (S2-o). Prepared according GP1 from 4-(trifluoromethyl)aniline (2.38 g, 14.8 mmol), pyridine (2.29 g, 29.0 mmol) and 4-(dimethylamino)benzenesulfonyl chloride (3.19 g, 14.5 mmol) at 0 °C for 1 h; crystallization from heptane/EtOAc 7:3 yielded **S2-o** (4.81 g, 96%) as a white solid: R_f 0.25 (heptane/EtOAc 7:3); m.p. 169–171 °C; ^{1}H-NMR (300 MHz, $CDCl_3$): δ 7.92 (s, 1H), 7.80–7.65 (m, 2H), 7.43 (d, J = 8.5 Hz, 2H), 7.20 (d, J = 8.4 Hz, 2H), 6.67–6.53 (m, 2H), 2.98 (s, 6H); ^{13}C-NMR (75 MHz, $CDCl_3$): δ 153.2, 140.7 (q, J = 1.1 Hz), 129.2, 126.5 (q, J = 3.7 Hz), 125.8 (q, J = 32.8 Hz), 124.1 (q, J = 271.6 Hz), 123.3, 119.0, 110.9, 40.0; IR (neat): 3244, 1618, 1595, 1523, 1481, 1321, 1142, 1109, 1068, 1015, 960, 944; HRMS (ESI-Orbitrap) *m/z* calcd for $C_{15}H_{15}O_2N_2F_3SNa$ $[M+Na]^+$: 367.0699; found: 367.0696.

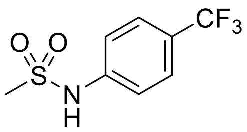

$C_8H_8F_3NO_2S$
239.21 g/mol

***N*-(4-(Trifluoromethyl)phenyl)methanesulfonamide (S2-p).** Prepared according GP1 from 4-(trifluoromethyl)aniline (2.47 g, 15.3 mmol), pyridine (2.37 g, 30.0 mmol) and methanesulfonyl chloride (1.72 g, 15.0 mmol) at 0 °C for 1 h; column chromatography (heptane/EtOAc 5:5) yielded **S2-p** (3.65 g, 100%) as a white solid: R_f 0.45 (heptane/EtOAc 5:5); m.p. 123 °C; ^{1}H-NMR (300 MHz, $CDCl_3$): δ 7.61 (d, J = 8.5 Hz, 2H), 7.47 (s, 1H), 7.34 (d, J = 8.5 Hz, 2H), 3.10 (s, 3H); ^{13}C-NMR (75 MHz, $CDCl_3$): δ 140.1 (q, J = 1.1 Hz), 127.04 (q, J = 3.7 Hz), 126.96 (q, J = 33.1 Hz), 123.9 (q, J = 271.7 Hz), 119.2, 39.8; IR (neat): 3280, 1619, 1520, 1480, 1400, 1319, 1310, 1297, 1237, 1149, 1102, 1071, 976, 962; HRMS (ESI-Orbitrap) *m/z* calcd for $C_8H_7O_2NF_3S$ $[M-H]^-$: 238.0155; found: 238.0156.

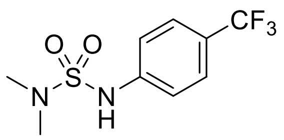

$C_9H_{11}F_3N_2O_2S$
268.25 g/mol

1-(Dimethylsulfamoylamino)-4-(trifluoromethyl)benzene (S2-q). Prepared according GP1 from 4-(trifluoromethyl)aniline (3.29 g, 20.4 mmol), pyridine (3.16 g, 40.0 mmol) and *N*,*N*-dimethylsulfamoyl chlorid (2.87 g, 20.0 mmol) at room temperature for 2 d; crystallization from heptane/EtOAc 6:4 yielded **S2-q** (2.59 g, 48%) as a white solid: R_f 0.40 (heptane/EtOAc 6:4); m.p. 115 °C; ^{1}H-NMR (300 MHz, $CDCl_3$): δ 7.59 (s, 1H), 7.56 (d, J = 8.5 Hz, 2H), 7.26 (d, J = 8.5 Hz, 2H), 2.88 (s, 6H); ^{13}C-NMR (75 MHz, $CDCl_3$): δ 140.7 (q, J = 1.0 Hz), 126.7 (q, J = 3.8 Hz), 125.9 (q, J = 32.9 Hz), 124.1 (q, J = 271.6 Hz), 118.4, 38.1; IR (neat): 3241, 1617, 1523, 1481, 1403, 1321, 1310, 1298, 1244, 1141, 1106, 1067, 1015, 939; HRMS (ESI-Orbitrap) *m/z* calcd for $C_9H_{10}O_2N_2F_3S$ $[M-H]^-$: 267.0421; found: 267.0419.

Synthesis of *N*-fluoro-*N*-alkyl amides/sulfonamides

General Procedure 1 (GP1): Synthesis of amides and sulfonamides (S1 and S2)

According to the literature procedure[20], to a suspension of potassium hydride (3.52 g, 90.0 mmol) in DCM (60 mL) was added *N*-alky lamide/sulfonamide (15.0 mmol) and stirred at room temperature for 30 min. Then NFSI (14.19 g, 45.0 mmol) in DCM (60 mL) was added and the reaction mixture was allowed to stir at room temperature for 4–16 h. The reaction mixture was cooled to 0 °C and quenched by the dropwise addition of water. The mixture was diluted with a NaOH-NH_4OH solution (300 mL, 20 g NH_4OH and 65 g NaOH in 1000 mL H_2O) and extracted with Et_2O (300/100 mL). The organic layers were washed with a NaOH-NH_4OH solution (200 mL), a 2 M aqueous solution of HCl (2 × 200 mL), and brine (200 mL). The organic layers were dried over Na_2SO_4, and concentrated. The crude product was purified by column chromatography.

General Procedure 3 (GP3): *N*-Fluoro-*N*-arylsulfonamides (4)

According to the literature procedure[21], to a solution of *N*-arylsulfonamides (10.0 mmol) in DCM (100 mL) was added cesium carbonate (6.52 g, 20.0 mmol) and stirred at room temperature for 1 h. Then NFSI (6.31, 20.0 mmol) was added and the mixture was allowed to stir at room temperature for 1–6 h. The mixture was diluted with pentane (100) mL and filtrated. The filtrate was concentrated and the residue was suspended in pentane/Et_2O 9:1, 8:2 or 7:3 (100 mL) at room temperature. The suspension was filtrated and concentrated. The crude product was

purified by FAST column chromatography (nearly all products are unstable on silicagel) and/or recrystallization.

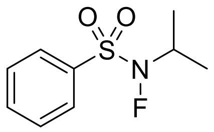
$C_9H_{12}FNO_2S$
217.26 g/mol

***N*-Fluoro-*N*-isopropylbenzenesulfonamide (3a).** Prepared according GP2 from potassium hydride (3.52 g, 90.0 mmol), **S1-a** (2.99 g, 15.0 mmol) and NFSI (14.19 g, 45.0 mmol) at room temperature for 4 h; column chromatography (pentane/DCM 6:4) yielded **3a** (1.48 g, 45%) as a slightly yellow and clear liquid: R_f 0.30 (heptane/DCM 6:4); ^{1}H-NMR (300 MHz, $CDCl_3$): δ 7.97 (d, J = 8.0 Hz, 2H), 7.80–7.66 (m, 1H), 7.58 (t, J = 7.8 Hz, 2H), 4.26–3.98 (m, 1H), 1.36–1.27 (m, 6H); ^{13}C-NMR (75 MHz, $CDCl_3$): δ 135.3, 134.5, 129.3 (d, J = 0.5 Hz), 129.2, 55.4 (d, J = 13.3 Hz), 19.3 (d, J = 6.8 Hz); ^{19}F-NMR (282 MHz, $CDCl_3$): δ −75.8 (d, J = 34.5 Hz).

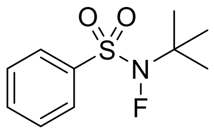
$C_{10}H_{14}FNO_2S$
231.29 g/mol

***N*-(*tert*-Butyl)-*N*-fluorobenzenesulfonamide (3b).** Prepared according GP2 from potassium hydride (3.52 g, 90.0 mmol), **S1-b** (3.20 g, 15.0 mmol) and NFSI (14.19 g, 45.0 mmol) at room temperature for 6 h; column chromatography (pentane/DCM 6:4) yielded **3b** (2.78 g, 80%) as a slightly yellow and clear liquid: R_f 0.50 (heptane/DCM 6:4); ^{1}H-NMR (300 MHz, $CDCl_3$): δ 8.03–7.95 (m, 2H), 7.72–7.63 (m, 1H), 7.61–7.51 (m, 2H), 1.47 (d, J = 1.8 Hz, 9H); ^{13}C-NMR (75 MHz, $CDCl_3$): δ 137.4, 134.2, 129.1 (d, J = 1.3 Hz), 129.0, 66.6 (d, J = 12.1 Hz), 27.2 (d, J = 6.1 Hz); ^{19}F-NMR (282 MHz, $CDCl_3$): δ −62.4 (s).

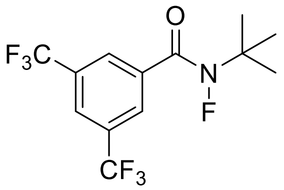
$C_{13}H_{12}F_7NO$
331.23 g/mol

***N*-(*tert*-Butyl)-*N*-fluoro-3,5-bis(trifluoromethyl)benzamide (3c).** Prepared according GP2 from potassium hydride (3.52 g, 90.0 mmol), **S1-c** (4.70 g, 15.0 mmol) and NFSI (14.19 g, 45.0 mmol) at room temperature for 16 h; column chromatography (pentane/DCM 9:1) yielded **3c** (1.17 g, 24%) as a yellow and clear liquid: R_f 0.40 (heptane/DCM 9:1); ^{1}H-NMR (300 MHz, $CDCl_3$): δ 8.16 (s, 2H), 8.00 (s, 1H), 1.57 (d, J = 2.0 Hz, 9H); ^{13}C-NMR (75 MHz, $CDCl_3$): δ 171.1 (d, J = 8.0 Hz), 136.0 (d, J = 1.2 Hz), 131.9 (q, J = 34.1 Hz), 129.5–129.1 (m), 125.3 (spt, J = 3.7 Hz), 123.0 (q, J = 273.0 Hz), 64.9 (d, J = 10.6 Hz), 26.7 (d, J = 5.9 Hz); ^{19}F-NMR (282 MHz, $CDCl_3$): δ −63.0 (s), −65.3 (s); IR (neat): 2985, 1691, 1621, 1461, 1369, 1275, 1175, 1128, 1110, 986, 907; HRMS (ESI-Orbitrap) *m/z* calcd for $C_{13}H_{13}ONF_7$ $[M+H]^+$: 332.0880; found: 332.0881.

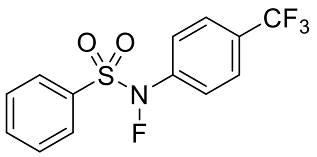

$C_{13}H_9F_4NO_2S$
319.27 g/mol

***N*-Fluoro-*N*-(4-(Trifluoromethyl)phenyl)benzenesulfonamide (4a).** Prepared according GP3 from cesium carbonate (11.08 g, 34.0 mmol), **S2-a** (5.12 g, 17.0 mmol) and NFSI (10.72 g, 34.0 mmol) at room temperature for 6 h; suspension in pentane/Et_2O 9:1; column chromatography (heptane/TBME 9:1) and crystallization from heptane yielded **4a** (4.17 g, 77%) as a slightly yellow solid: R_f 0.40 (heptane/TBME 9:1); m.p. 74–75 °C; ^{1}H-NMR (300 MHz, $CDCl_3$): δ 7.77–7.71 (m, 1H), 7.70–7.63 (m, 2H), 7.59 (d, J = 8.4 Hz, 2H), 7.56–7.49 (m, 2H), 7.24 (d, J = 8.6 Hz, 2H); ^{13}C-NMR (75 MHz, $CDCl_3$): δ 142.8 (dq, J = 9.3, 1.3 Hz), 135.5, 131.1 (qd, J = 33.1, 1.3 Hz), 130.9, 130.2 (d, J = 0.5 Hz), 129.2, 125.9 (qd, J = 3.7, 2.0 Hz), 123.5 (q, J = 272.4 Hz), 122.3 (d, J = 10.1 Hz); ^{19}F-NMR (282 MHz, $CDCl_3$): δ −39.7 (s), −62.7 (s); IR (neat): 1615, 1583, 1454, 1416, 1360, 1323, 1162, 1133, 1109, 1086, 1065, 1019, 910; HRMS (ESI-Orbitrap) *m/z* calcd for $C_{13}H_{10}O_2NF_4S$ $[M+H]^+$: 320.0363; found: 320.0366.

Scale-up procedure:

To a solution of **S2-a** (12.05 g, 40.0 mmol) in DCM (400 mL) was added Cs_2CO_3 (16.90 g, 52.0 mmol) and stirred at room temperature for 60 min. Then NFSI (16.40 g, 52.0 mmol) was added and the mixture was allowed to stir at room temperature for 5 h. The mixture was diluted with pentane (400 mL), filtrated, and concentrated. The product was purified by FAST column chromatography (heptane/TBME 85:15). The fractions were evaporated under reduced pressure to a volume of 100-150 mL whereby the product crystallized out. To complete the crystallization the flask was stored in the fridge overnight to yield **4a** (10.15 g, 80%) as a slightly yellow solid.

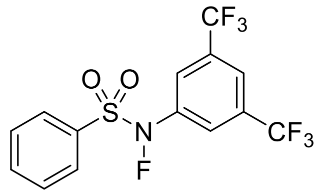

$C_{14}H_8F_7NO_2S$
387.27 g/mol

***N*-(3,5-Bis(Trifluoromethyl)phenyl)-*N*-fluorobenzenesulfonamide (4b).** Prepared according GP3 from cesium carbonate (9.81 g, 30.0 mmol), **S2-b** (5.54 g, 15.0 mmol) and NFSI (9.46 g, 30.0 mmol) at room temperature for 4 h; suspension in pentane/Et_2O 9:1; column chromatography (heptane/TBME 9:1) yielded **4b** (5.40 g, 93%) as a slightly yellow liquid: R_f 0.50 (heptane/TBME 9:1); ^{1}H-NMR (300 MHz, $CDCl_3$): δ 7.86 (s, 1H), 7.81–7.64 (m, 1H), 7.71–7.64 (m, 2H), 7.62–7.48 (m, 4H); ^{13}C-NMR (75 MHz, $CDCl_3$): δ 141.6 (d, J = 9.8 Hz), 135.9, 132.5 (qd, J = 34.3, 1.6 Hz), 130.3, 130.2, 129.4, 122.9–122.4 (m), 122.5 (q, J = 273.1 Hz); ^{19}F-NMR (282 MHz, $CDCl_3$): δ −37.6 (s), −63.2 (s); IR (neat): 1450, 1393, 1366, 1276, 1173, 1129, 1111, 1087, 967, 927, 892; Elem. anal. calcd for $C_{14}H_8F_7NO_2S$: C, 43.42; H, 2.08; F, 34.34; N, 3.62; O, 8.26; S, 8.28; found: C, 43.82; H, 1.81.

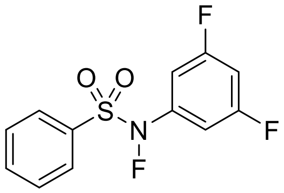

$C_{12}H_8F_3NO_2S$
287.26 g/mol

***N*-(3,5-Difluorophenyl)-*N*-fluorobenzenesulfonamide (4c).** Prepared according GP3 from cesium carbonate (6.52 g, 20.0 mmol), **S2-c** (2.69 g, 10.0 mmol) and NFSI (6.31 g, 20.0 mmol) at room temperature for 4 h; suspension in pentane/Et_2O 9:1; column chromatography (heptane/TBME 9:1) and crystallization from heptane yielded **4c** (0.58 g, 20%) as a slightly yellow solid: R_f 0.55 (heptane/TBME 9:1); m.p. 64–65 °C; ^{1}H-NMR (300 MHz, $CDCl_3$): δ 7.80–7.69 (m, 3H), 7.60–7.52 (m, 2H), 6.80 (tt, J = 8.6, 2.3 Hz, 1H), 6.74–6.63 (m, 2H); ^{13}C-NMR (75 MHz, $CDCl_3$): δ 162.4 (ddd, J = 250.7, 13.5, 2.4 Hz), 142.1 (td, J = 11.7, 10.4 Hz), 135.6, 130.8, 130.1 (d, J = 0.5 Hz), 129.2, 106.0–105.4 (m), 104.5 (t, J = 25.4 Hz); ^{19}F-NMR (282 MHz, $CDCl_3$): δ −37.5 (s), −107.2 (t, J = 7.2 Hz); IR (neat): 1603, 1464, 1449, 1392, 1362, 1312, 1214, 1171, 1126, 1086; HRMS (ESI-Orbitrap) *m/z* calcd for $C_{12}H_9O_2NF_3S$ $[M+H]^+$: 288.0294; found: 288.0297.

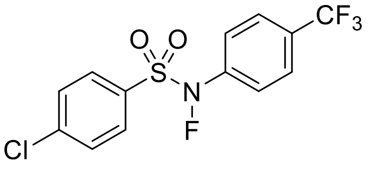

$C_{13}H_8ClF_4NO_2S$
353.72 g/mol

4-Chloro-*N*-fluoro-*N*-(4-(trifluoromethyl)phenyl)benzene-sulfonamide (4d). Prepared according GP3 from cesium carbonate (6.52 g, 20.0 mmol), **S2-d** (3.36 g, 10.0 mmol) and NFSI (6.31 g, 20.0 mmol) at room temperature for 2 h; suspension in pentane/Et_2O 8:2; column chromatography (heptane/TBME 7:3) and crystallization from heptane yielded **4d** (1.48 g, 42%) as a slightly yellow solid: R_f 0.45 (heptane/TBME 7:3); m.p. 100–101 °C; ^{1}H-NMR (300 MHz, $CDCl_3$): δ 7.67–7.58 (m, 4H), 7.55–7.47 (m, 2H), 7.28 (d, J = 8.7 Hz, 2H); ^{13}C-NMR (75 MHz, $CDCl_3$): δ 142.6, 142.4 (dq, J = 9.5, 1.3 Hz), 131.5 (d, 0.5 Hz), 131.3 (qd, J = 33.1, 1.3 Hz), 129.6, 129.4, 125.8 (qd, J = 3.7, 2.0 Hz), 123.5 (q, J = 272.4 Hz), 122.3 (d, J = 9.9 Hz); ^{19}F-NMR (282 MHz, $CDCl_3$): δ −39.3 (s), −62.7 (s); IR (neat): 1614, 1572, 1471, 1416, 1385, 1324, 1176, 1164, 1109, 1066, 1012, 946, 877; HRMS (ESI-Orbitrap) *m/z* calcd for $C_{13}H_7O_2NClF_4S$ $[M-H]^-$: 351.9828; found: 351.9820.

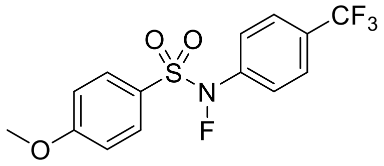

$C_{14}H_{11}F_4NO_3S$
349.30 g/mol

***N*-Fluoro-4-methoxy-*N*-(4-(trifluoromethyl)phenyl)-benzenesulfonamide (4e).** Prepared according GP3 from cesium carbonate (13.03 g, 40.0 mmol), **S2-e** (6.63 g, 20.0 mmol) and NFSI (12.61 g, 40.0 mmol) at room temperature for 6 h; suspension in pentane/Et_2O 8:2; column chromatography (heptane/TBME 7:3) and crystallization

from heptane yielded **4e** (6.07 g, 87%) as a slightly yellow solid: R_f 0.35 (heptane/TBME 7:3); m.p. 74°C; ^{1}H-NMR (300 MHz, $CDCl_3$): *δ* 7.65–7.51 (m, 4H), 7.25 (d, *J* = 8.7 Hz, 2H), 7.01–6.91 (m, 2H), 3.90 (s, 3H); ^{13}C-NMR (75 MHz, $CDCl_3$): *δ* 165.2, 143.1 (dq, *J* = 9.2, 1.2 Hz), 132.5, 130.9 (qd, *J* = 32.8, 1.2 Hz), 125.8 (qd, *J* = 3.7, 2.1 Hz), 123.6 (q, *J* = 272.3 Hz), 122.3 (d, *J* = 10.3 Hz), 121.9, 114.4, 55.9; ^{19}F-NMR (282 MHz, $CDCl_3$): *δ* −39.9 (s), −62.6 (s); IR (neat): 1614, 1593, 1576, 1498, 1415, 1362, 1321, 1268, 1161, 1107, 1089, 1062, 1029, 1015; HRMS (ESI-Orbitrap) *m/z* calcd for $C_{14}H_{10}O_3NF_4S$ $[M{-}H]^-$: 348.0323; found: 348.0315.

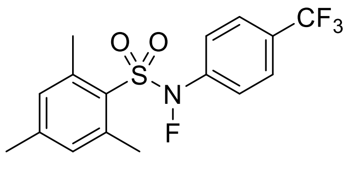

$C_{16}H_{15}F_4NO_2S$
361.35 g/mol

***N*-Fluoro-2,4,6-trimethyl-*N*-(4-(trifluoromethyl)phenyl)-benzenesulfonamide (4f).** Prepared according GP3 from cesium carbonate (6.52 g, 20.0 mmol), **S2-f** (3.43 g, 10.0 mmol) and NFSI (6.31 g, 20.0 mmol) at room temperature for 4 h; suspension in pentane/Et_2O 9:1; column chromatography (heptane/TBME 9:1) and crystallization from heptane yielded **4f** (2.61 g, 72%) as a slightly yellow solid: R_f 0.55 (heptane/TBME 9:1); m.p. 116–117 °C; ^{1}H-NMR (300 MHz, $CDCl_3$): *δ* 7.69 (d, *J* = 8.4 Hz, 2H), 7.55 (d, *J* = 8.4 Hz, 2H), 7.04 (s, 2H), 2.63 (s, 6H), 2.35 (s, 3H); ^{13}C-NMR (75 MHz, $CDCl_3$): *δ* 145.6, 142.9, 141.7 (dq, *J* = 9.6, 1.2 Hz), 132.4, 131.5 (qd, *J* = 32.9, 1.6 Hz), 127.4, 125.9 (qd, *J* = 3.5, 2.2 Hz), 124.1 (d, *J* = 10.1 Hz), 123.6 (q, *J* = 272.6 Hz), 23.1 (d, *J* = 0.8 Hz), 21.2; ^{19}F-NMR (282 MHz, $CDCl_3$): *δ* −36.4 (s), −62.7 (s); IR (neat): 1614, 1600, 1354, 1322, 1194, 1166, 1132, 1108, 1065, 1039, 1016, 908; HRMS (ESI-Orbitrap) *m/z* calcd for $C_{16}H_{16}O_2NF_4S$ $[M{+}H]^+$: 362.0832; found: 362.0830.

Scale-up procedure:

To a solution of **S2-f** (17.17 g, 50.0 mmol) in DCM (400 mL) was added Cs_2CO_3 (21.18 g, 65.0 mmol) and stirred at room temperature for 60 min. Then NFSI (20.50 g, 65.0 mmol) was added and the mixture was allowed to stir at room temperature for 3 h. The mixture was diluted with pentane (400 mL), filtrated, and concentrated. The product was purified by FAST column chromatography (heptane/TBME 85:15). The fractions were evaporated under reduced pressure to a volume of 100-150 mL whereby the product crystallized out. To complete the crystallization the flask was stored in the fridge overnight to yield **4f** (16.16 g, 89%) as a slightly yellow solid.

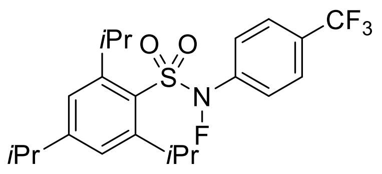

$C_{22}H_{27}F_4NO_2S$
445.52 g/mol

***N*-Fluoro-2,4,6-triisopropyl-*N*-(4-(trifluoromethyl)-phenyl)benzenesulfonamide (4g).** Prepared according GP3 from cesium carbonate (9.12 g, 28.0 mmol), **S2-g** (5.99 g, 14.0 mmol) and NFSI (8.83 g, 28.0 mmol) at room temperature for 4 h; suspension in pentane/Et_2O 9:1; column chromatography (heptane/TBME 9:1) and crystallization from pentane at −78 °C yielded **4g** (4.46 g, 72%) as a slightly yellow and viscous oil: R_f 0.75 (heptane/TBME 9:1); ^{1}H-NMR (300 MHz, $CDCl_3$): δ 7.71 (d, J = 8.4 Hz, 2H), 7.59 (d, J = 8.5 Hz, 2H), 7.26 (s, 2H), 4.12 (hept, J = 6.7 Hz, 2H), 2.95 (hept, J = 6.9 Hz, 1H), 1.40–1.13 (m, 18H); ^{13}C-NMR (75 MHz, $CDCl_3$): δ 155.8, 153.8, 142.0 (dq, J = 9.5, 1.2 Hz), 131.5 (qd, J = 32.9, 1.5 Hz), 126.5, 126.0 (qd, J = 3.7, 1.1 Hz), 124.6, 124.2 (d, J = 10.1 Hz), 123.6 (q, J = 272.4 Hz), 34.4, 30.3, 24.8, 23.4; ^{19}F-NMR (282 MHz, $CDCl_3$): δ −36.5 (s), −62.8 (s); IR (neat): 2961, 2931, 1613, 1598, 1463, 1427, 1321, 1168, 1129, 1110, 1066, 1017, 938; HRMS (ESI-Orbitrap) *m/z* calcd for $C_{22}H_{28}O_2NF_4S$ $[M+H]^+$: 446.1771; found: 446.1771.

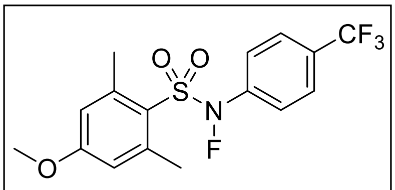

$C_{16}H_{15}F_4NO_3S$
377.35 g/mol

***N*-Fluoro-4-methoxy-2,6-dimethyl-*N*-(4-(trifluoromethyl)-phenyl)benzenesulfonamide (4h).** Prepared according GP3 from cesium carbonate (7.82 g, 24.0 mmol), **S2-h** (4.31 g, 12.0 mmol) and NFSI (3.78 g, 12.0 mmol) at room temperature for 1 h; suspension in pentane/Et_2O 7:3; crystallization from heptane yielded **4h** (2.40 g, 53%) as a slightly yellow solid: R_f 0.45 (heptane/TBME 7:3); m.p. 104–105 °C; ^{1}H-NMR (300 MHz, $CDCl_3$): δ 7.68 (d, J = 8.6 Hz, 2H), 7.54 (d, J = 8.6 Hz, 2H), 6.71 (s, 2H), 3.86 (s, 3H), 2.62 (s, 6H); ^{13}C-NMR (75 MHz, $CDCl_3$): δ 163.4, 145.8, 142.0 (dq, J = 9.5, 1.4 Hz), 131.4 (qd, J = 33.0, 1.9 Hz), 125.9 (qd, J = 3.7, 1.6 Hz), 124.1 (d, J = 10.0 Hz), 123.6 (q, J = 272.3 Hz), 121.7, 116.6, 55.5, 23.7 (d, J = 0.9 Hz); ^{19}F-NMR (282 MHz, $CDCl_3$): δ −36.6 (s), −62.7 (s); IR (neat): 2949, 1591, 1573, 1474, 1443, 1414, 1352, 1310, 1161, 1119, 1110, 1089, 1064, 1033, 1016, 1009; HRMS (ESI-Orbitrap) *m/z* calcd for $C_{16}H_{16}O_3NF_4S$ $[M+H]^+$: 378.0782; found: 378.0778.

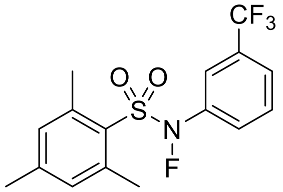

$C_{16}H_{15}F_4NO_2S$
361.35 g/mol

***N*-Fluoro-2,4,6-trimethyl-*N*-(3-(trifluoromethyl)phenyl)-benzenesulfonamide (4i).** Prepared according GP3 from cesium carbonate (3.91 g, 12.0 mmol), **S2-i** (2.06 g, 6.0 mmol) and NFSI (1.89 g, 6.0 mmol) at room temperature for 2 h; suspension in pentane/Et_2O 9:1; crystallization from heptane yielded **4i** (0.86 g, 40%) as a slightly yellow solid: R_f 0.55 (heptane/TBME 8.5:1.5); m.p.

81–82 °C; ^{1}H-NMR (300 MHz, $CDCl_3$): δ 7.72–7.63 (m, 2H), 7.61–7.51 (m, 2H), 7.03 (s, 2H), 2.60 (s, 6H), 2.35 (s, 3H); ^{13}C-NMR (75 MHz, $CDCl_3$): δ 145.6, 142.9, 139.4 (d, J = 10.0 Hz), 132.4, 131.5 (qd, J = 33.2, 1.1 Hz), 129.4 (d, J = 0.5 Hz), 127.8 (dq, J = 9.0, 0.7 Hz), 127.0, 126.5 (qd, J = 3.5, 1.7 Hz), 123.4 (q, J = 272.7 Hz), 121.3–120.9 (m), 23.1 (d, J = 0.9 Hz), 21.2; ^{19}F-NMR (282 MHz, $CDCl_3$): δ −33.5 (s), −62.8 (s); IR (neat): 1603, 1448, 1355, 1321, 1287, 1161, 1122, 1093, 1070, 916; HRMS (ESI-Orbitrap) *m/z* calcd for $C_{16}H_{14}O_2NF_4S$ $[M-H]^-$: 360.0687; found: 360.0683.

Other *N*-fluoro-*N*-arylsulfonamides

Attempts to prepare *N*-arylsulfonamides **3j**-**3q** from amide **2j**-**2q** were unsuccessful.

Crystal structures of NFASs[22,23]

Crystal-Structure Determination of *N*-Fluoro-*N*-(4-(trifluoromethyl)phenyl)-benzenesulfonamide (4a)

CCDC 1828679 contains the supplementary crystallographic data for **4a**. These data can be obtained free of charge from The Cambridge Crystallographic Data Centre via http://www.ccdc.cam.ac.uk/data_request/cif.

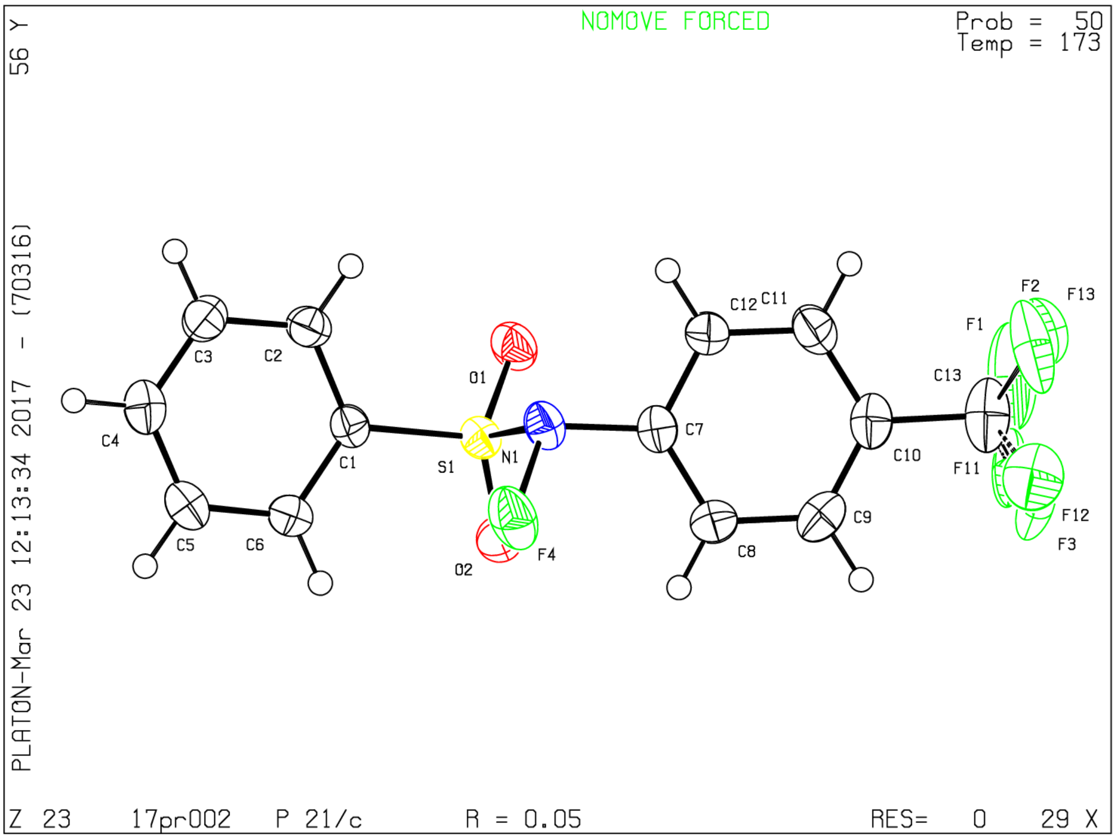

Supplementary Figure 1. ORTEP drawing of **4a**. Thermal ellipsoids are drawn at 50% probability level.

A crystal of $C_{13}H_7F_4NO_2S$ was mounted in air at ambient conditions. All measurements were made on a *Oxford Diffraction SuperNova* area-detector diffractometer[1] using mirror optics monochromated Mo *K*α radiation (λ = 0.71073 Å) and Al filtered.[2] The unit cell constants and an orientation matrix for data collection were obtained from a least-squares refinement of the setting angles of reflections in the range 2.96 < θ < 26.74°. A total of 584 frames were collected using ω scans, with 5+5 seconds exposure time, a rotation angle of 1.0° per frame, a crystal-detector distance of 65.0 mm, at T = 173(2) K.

Data reduction was performed using the *CrysAlisPro*[1] program. The intensities were corrected for Lorentz and polarization effects, and an absorption correction based on the multi-scan method using SCALE3 ABSPACK in *CrysAlisPro*[1] was applied. Data collection and refinement parameters are given in *Table 1.*

The structure was solved by direct methods using *SHELXT*[3], which revealed the positions of all non-hydrogen atoms of the title compound. The non-hydrogen atoms were refined anisotropically. All H-atoms were placed in geometrically calculated positions and refined using

a riding model where each H-atom was assigned a fixed isotropic displacement parameter with a value equal to 1.2Ueq of its parent atom.

Refinement of the structure was carried out on F^2 using full-matrix least-squares procedures, which minimized the function $\Sigma w(F_o^2 - F_c^2)^2$. The weighting scheme was based on counting statistics and included a factor to downweight the intense reflections. All calculations were performed using the *SHELXL-2014/7*[4] program.

The CF3 group was found to be disordered over two conformation, with unequal distribution (ca. 75%:25%), despite the low temperature measurement.

Crystal-Structure Determination of *N*-Fluoro-2,4,6-trimethyl-*N*-(4-(trifluoromethyl)phenyl)benzenesulfonamide (4f)

CCDC 1828684 contains the supplementary crystallographic data for **4f**. These data can be obtained free of charge from The Cambridge Crystallographic Data Centre via http://www.ccdc.cam.ac.uk/data_request/cif.

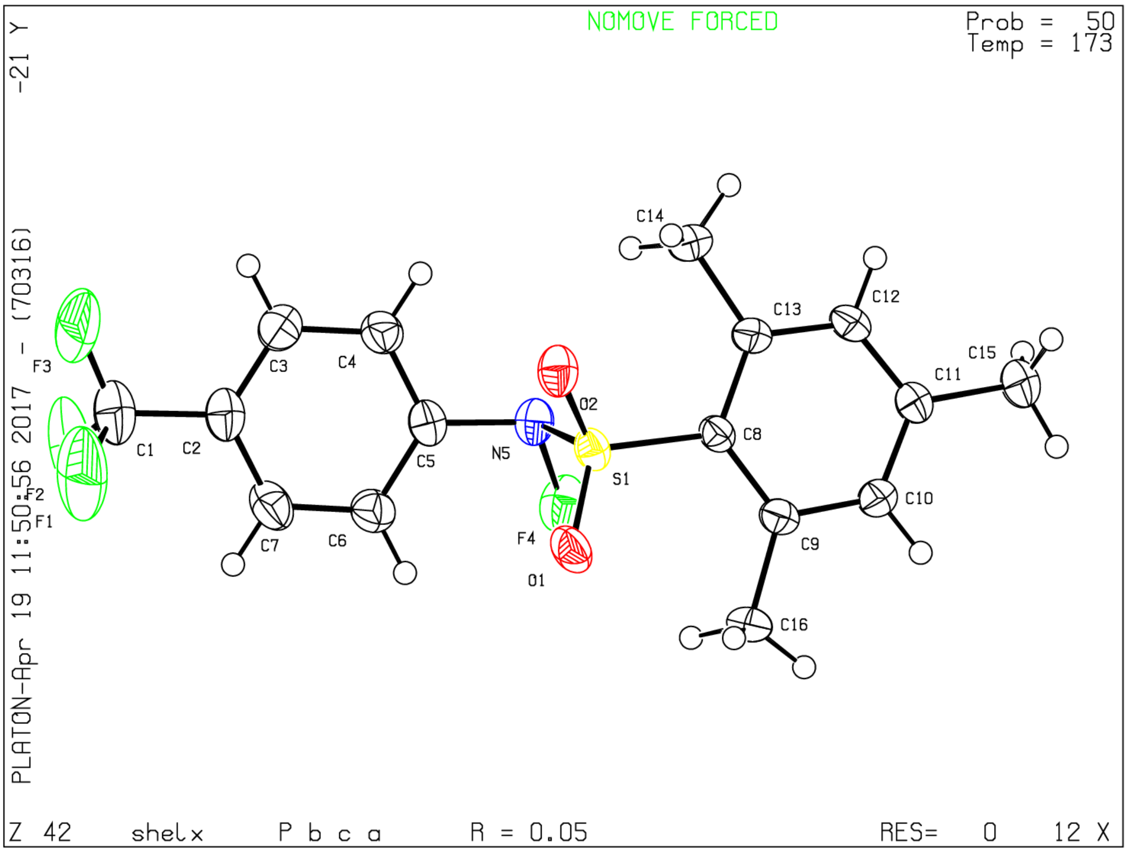

Supplementary Figure 2. ORTEP drawing of **4f**. Thermal ellipsoids are drawn at 50% probability level.

A crystal of C16H15F4NO2S was mounted in air at ambient conditions. All measurements were made on a Oxford Diffraction SuperNova area-detector diffractometer1 using mirror optics monochromated Mo Kα radiation (λ = 0.71073 Å) and Al filtered.2 The unit cell constants and an orientation matrix for data collection were obtained from a least-squares refinement of the setting angles of reflections in the range 2.0 < θ < 27.5°. A total of 317 frames were collected using ω scans, with 20+20 seconds exposure time, a rotation angle of 1.0° per frame, a crystal-detector distance of 65.0 mm, at T = 173(2) K.

Data reduction was performed using the CrysAlisPro1 program.[24] The intensities were corrected for Lorentz and polarization effects, and an absorption correction based on the multi-scan method using SCALE3 ABSPACK in CrysAlisPro1 was applied.

The structure was solved by direct methods using SHELXT3, which revealed the positions of all non-hydrogen atoms of the title compound. The non-hydrogen atoms were refined anisotropically. All H-atoms were placed in geometrically calculated positions and refined using a riding model where each H-atom was assigned a fixed isotropic displacement parameter with a value equal to 1.2Ueq of its parent atom.

Refinement of the structure was carried out on F2 using full-matrix least-squares procedures, which minimized the function Σw(Fo2 – Fc2)2. The weighting scheme was based on counting statistics and included a factor to downweight the intense reflections. All calculations were performed using the SHELXL-2014/74 program.

Hydrofluorination

Hydrofluorination of 1-phenyl-1-cyclohexene with Selectfluor, NFSI and 3a–3c and 4a–4i.

To a solution of 1-phenylcyclohex-1-ene **1a** (158 mg, 1.0 mmol) and *N*,*N*-dimethylacetamide (14 µL, 0.15 mmol) in dry DCM (1 mL) was added dropwise catecholborane (0.23 mL, 2.2 mmol) at 0 °C. The reaction was allowed to stir at 30 °C for 16 h. The mixture was cooled to 0 °C and was quenched by the addition of *t*-BuOH (0.124 mL, 1.3 mmol) and stirred at room temperature for 15 min. The mixture was concentrated under high vacuum and the residue was dissolved in dry DMF (2mL). *n*-Undecane (84 µL, 0.4 mmol), **3a**–**3c** or **4a**–**4i** (3.0 mmol) and DTBHN or DTBPO (0.1–0.5 mmol) were added. The mixture was heated to 80 °C (initiation with DTBHN) or 60 °C (initiation with DTBPO) (preheated oil bath) and stirred at this temperature for 30 min. The yield of **2a** was determined by GC-FID using *n*-undecane as

internal standard (filtration of an aliquot over neutral ALOX using Et_2O as eluent). In most cases the reaction was complete within 10 min. Results are summarized in Scheme 2 and Table 1.

General Procedure 4 (GP4): Hydrofluorination of alkenes

To a solution of alkene **1** (1.0 mmol) and *N*,*N*-dimethylacetamide (14 µL, 0.15 mmol) in dry DCM (1 mL) was added dropwise catecholborane (0.23 mL, 2.2 mmol) at 0 °C. The reaction was allowed to stir at 30 °C for 16 h. The mixture was cooled to 0 °C and was quenched by the addition of *t*-BuOH (0.124 mL, 1.3 mmol) and stirred at room temperature for 15 min. The mixture was concentrated under high vacuum and the residue was dissolved in dry DMF (2 mL). **4a** or **4f** (3.0 mmol) and DTBPO (117 mg, 0.5 mmol) were added. The mixture was heated to 60 °C (preheated oil bath) and stirred at this temperature for 30–45 min. The crude product was purified by column chromatography.

(2-Fluorocyclohexyl)benzene 2a. Prepared according GP4 from **1a** (158 mg, 1.0 mmol) and **4f** (1084 mg, 3.0 mmol); column chromatography (pentane/DCM 8.5:1.5) yielded **2a** (85 mg, 48%, *trans*/*cis* 49:51) as a colorless and clear liquid. For analytical purposed, the two diastereomers were separated by column chromatography (pentane/DCM 9:1).

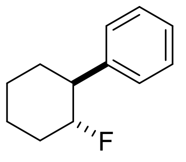

$C_{12}H_{15}F$
178.25 g/mol

***trans*-2a:** white solid, R_f 0.30 (heptane/DCM 9:1); m.p. 43–44 °C; ^{1}H-NMR (300 MHz, $CDCl_3$): δ 7.42–7.13 (m, 5H), 4.55 (dtd, J = 48.9, 10.4, 4.7 Hz, 1H), 2.84–2.55 (m, 1H), 2.33–2.17 (m, 1H), 2.02–1.84 (m, 2H), 1.80–1.68 (m, 1H), 1.66–1.23 (m, 4H); ^{13}C-NMR (75 MHz, $CDCl_3$): δ 142.8 (d, J = 0.7 Hz), 128.4, 127.5, 126.6, 95.1 (d, J = 175.8 Hz), 50.5 (d, J = 17.4 Hz), 33.1 (d, J = 7.9 Hz), 32.9 (d, J = 18.4 Hz), 25.6 (d, J = 2.1 Hz), 24.4 (d, J = 11.3 Hz); ^{19}F-NMR (282 MHz, $CDCl_3$): δ −171.1 (m); IR (neat): 3018, 2926, 2857, 1602, 1495, 1447, 1256, 1210, 1024, 955; HRMS (EI) *m/z* calcd for $C_{12}H_{15}F$ $[M]^+$: 178.1152; found: 178.1153.

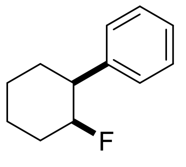

$C_{12}H_{15}F$
178.25 g/mol

***cis*-2a:** colorless and clear liquid; R_f 0.40 (heptane/DCM 9:1); ^{1}H-NMR (300 MHz, $CDCl_3$): δ 7.41–7.13 (m, 5H), 5.01–4.71 (m, 1H), 2.66 (dddd, J = 36.6, 12.9, 3.9, 1.4 Hz, 1H), 2.22–1.95 (m, 2H), 1.95–1.84 (m, 1H), 1.80–1.34 (m, 5H); ^{13}C-NMR (75 MHz, $CDCl_3$): δ 143.5, 128.3, 128.0 (d, J = 1.7 Hz), 126.5, 92.1 (d, J = 174.0 Hz), 47.6 (d, J = 19.5 Hz), 31.6 (d, J = 21.8 Hz), 26.5 (d, J = 2.9 Hz), 25.9, 19.7 (d, J = 1.3 Hz); ^{19}F-NMR (282 MHz, $CDCl_3$): δ −197.2 (m); IR (neat): 3028, 2932, 2864, 1603, 1497, 1446, 1148, 1103, 1052, 950; HRMS (EI) *m/z* calcd for $C_{12}H_{15}F$ $[M]^+$: 178.1152; found: 178.1153.

Reaction with **4a** (958 mg, 3.0 mmol) afforded **2a** (80 mg, 45%, *trans*/*cis* 56:44).

Reaction with NFSI (946 mg, 3.0 mmol) afforded **2a** (29% yield, trans/cis 58:42).

Gas chromatography analysis of the side products formed with the different fluorinating agents

The crude products of the reactions run with **4a**, **4f** and NFSI using 0.1 equiv DTBHN were analyzed by GC (50 °C (1 min) to 180 °C; 8 °C/min). The observed ratio of product **2a**/**1a**/phenylcyclohexane are given in the table and the chromatogram are depicted below. The amount of phenylcyclohexane resulting from a hydrogen transfer remain similar in all three cases but the reaction with NFSI produce significantly more of the alkene **1a**. Under the applied hydroboration conditions, the conversion of the alkene **1a** is complete. Therefore, **1a** detected after the fluorination step arises from side reactions.

Supplementary Table 1. Ratio of fluorides **2a**, alkene **1a** and phenylcyclohexane.

Fluorinating agent	*Cis- and trans-***2a**	**1a**	Phenylcyclohexane
**4a**	63%	20%	17%
**4f**	63%	13%	24%
NFSI	46%	36%	18%

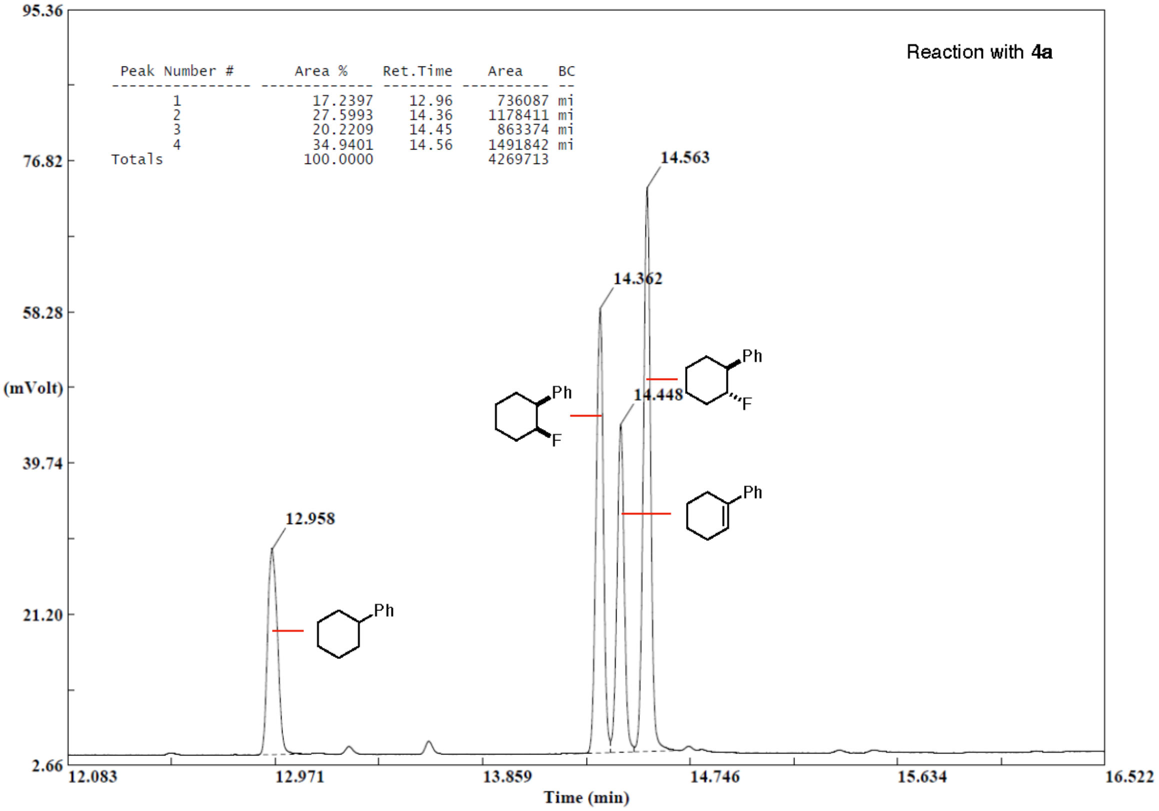

Supplementary Figure 3. GC trace for the reaction of **1a** with **4a**.

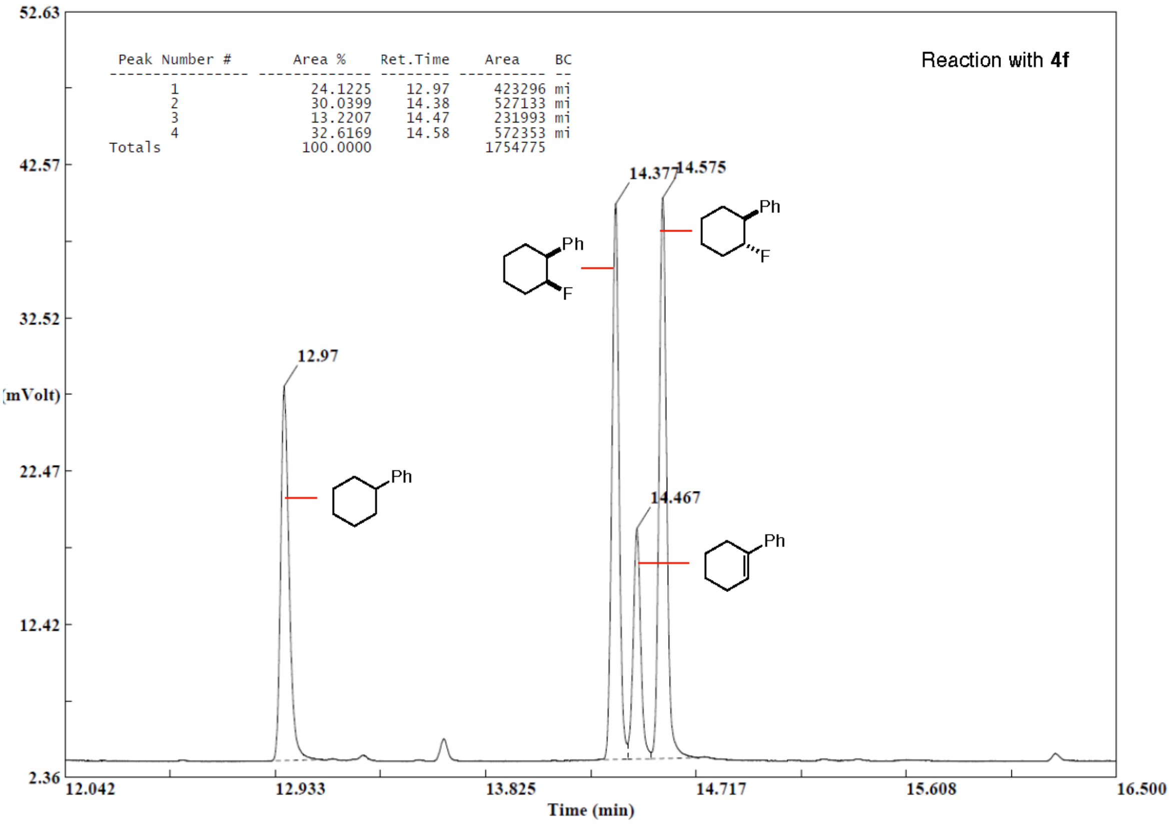

Supplementary Figure 4. GC trace for the reaction of **1a** with **4f**.

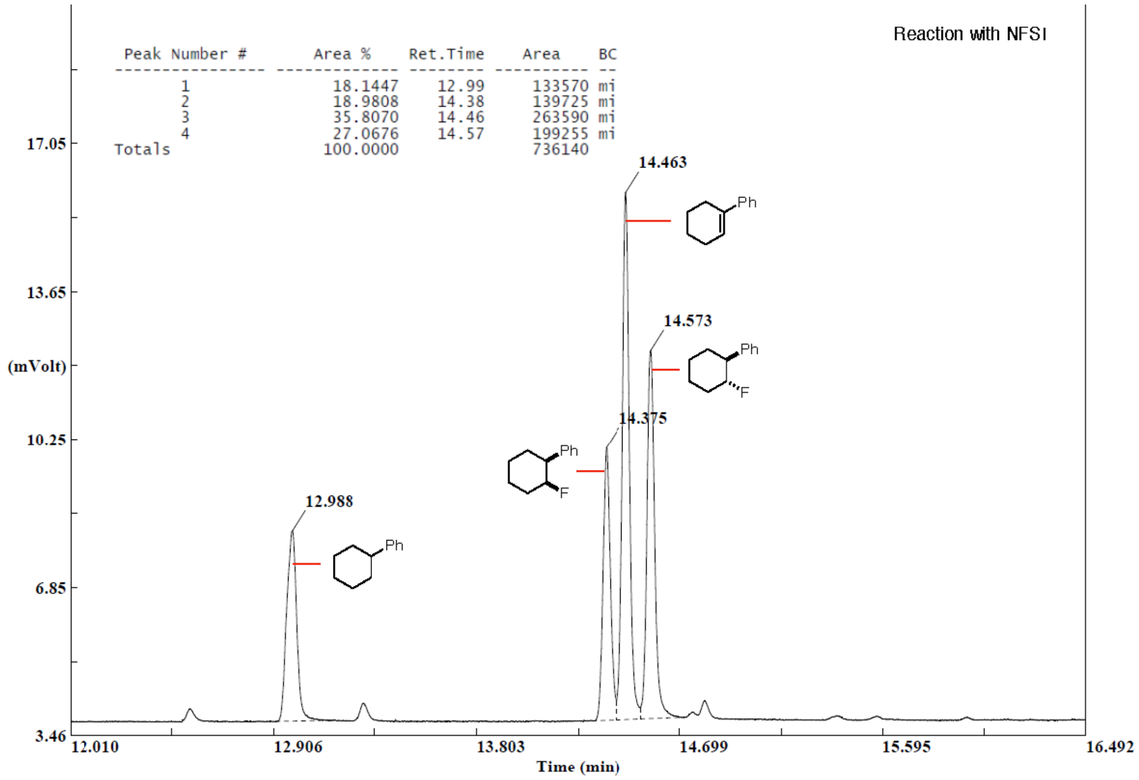

Supplementary Figure 5. GC trace for the reaction of **1a** with NFSI.

4-(2-Fluorocyclopentyl)-1,1'-biphenyl 2b. Prepared according GP4 from **1b** (220 mg, 1.0 mmol) and **4f** (1084 mg, 3.0 mmol); column chromatography (pentane/DCM 8.5:1.5) yielded **2b** (127 mg, 53%, *trans*/*cis* 88:12) as a white solid. The two diastereomers were separated by column chromatography (pentane/DCM 9:1).

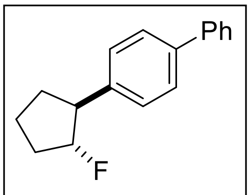

***trans*-2b:** white solid; R_f 0.40 (heptane/DCM 9:1); m.p. 49–50 °C; ^{1}H-NMR (300 MHz, $CDCl_3$): δ 7.62–7.50 (m, 4H), 7.46–7.37 (m, 2H), 7.35–7.25 (m, 3H), 5.06 (dd, J = 54.0, 4.4 Hz, 1H), 3.33 (dtd, J = 27.3, 8.3, 4.2 Hz, 1H), 2.38–2.13 (m, 1H), 2.10–2.02 (m, 1H), 2.02–1.66 (m, 4H); ^{13}C-NMR (75 MHz, $CDCl_3$): δ 141.7 (d, J = 5.0 Hz), 140.9, 139.4, 128.7, 127.6, 127.3, 127.2, 127.0, 101.7 (d, J = 179.1 Hz), 51.8 (d, J = 20.8 Hz), 32.7 (d, J = 21.8 Hz), 31.7 (d, J = 3.7 Hz), 22.9 (d, J = 2.2 Hz); ^{19}F-NMR (282 MHz, $CDCl_3$): δ −169.1 (m); IR (neat): 2970, 2934, 2866, 1597, 1484, 1449, 1432, 1113, 1072, 1008, 954; HRMS (ESI-Orbitrap) *m/z* calcd for $C_{17}H_{17}FNa$ [M+Na]$^+$: 263.1207; found: 263.1207.

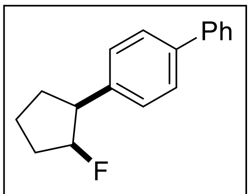

***cis*-2b:** white solid; R_f 0.35 (heptane/DCM 9:1); m.p. 106–107 °C; ^{1}H-NMR (300 MHz, $CDCl_3$): δ 7.68–7.50 (m, 4H), 7.45–7.36 (m, 4H), 7.37–7.27 (m, 1H), 5.14 (dtd, J = 54.0, 3.3, 1.3 Hz, 1H), 3.16–2.86 (m, 1H), 2.29–1.89 (m, 5H), 1.90–1.63 (m, 1H); ^{13}C-NMR (75 MHz, $CDCl_3$): δ 141.1, 139.5, 138.4 (d, J = 1.7 Hz), 129.0 (d, J = 1.6 Hz), 128.7, 127.1, 126.9, 97.7 (d, J = 177.9 Hz), 51.1 (d, J = 19.6 Hz), 33.0 (d, J = 21.5 Hz), 29.0, 22.2; ^{19}F-NMR (282 MHz, $CDCl_3$): δ −184.0 (m); IR (neat): 2974, 2876, 1597, 1486, 1447, 1407, 1252, 1155, 1076, 1006, 974; HRMS (ESI-Orbitrap) *m/z* calcd for $C_{17}H_{17}FNa$ [M+Na]$^+$: 263.1207; found: 263.1205.

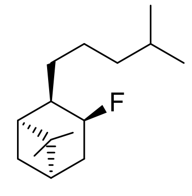

(1*S*,2*R*,3*S*,5*R*)-3-Fluoro-6,6-dimethyl-2-(4-methylpentyl)bicyclo[3.1.1]-heptane (2c). Prepared according GP4 from **1c** (206 mg, 1.0 mmol) and **4a** (958 mg, 3.0 mmol); column chromatography (pentane) yielded **2c** (140 mg, 62%, dr 98:2) as a colorless and clear liquid: R_f 0.60 (heptane); ^{1}H-NMR (300 MHz, $CDCl_3$): δ 4.87 (ddt, J = 51.9, 9.0, 3.4 Hz, 1H), 2.61–2.31 (m, 2H), 2.18–1.87 (m, 4H), 1.61–1.46 (m, 2H), 1.44–1.29 (m, 3H), 1.22 (s, 3H), 1.21–1.12 (m, 3H), 0.87 (d, J = 6.6 Hz, 6H), 0.83 (s, 3H); ^{13}C-NMR (75 MHz, $CDCl_3$): δ 93.2 (d, J = 170.3 Hz), 50.3 (d, J = 20.2 Hz), 45.4 (d, J = 5.6 Hz), 41.0 (d, J = 3.0 Hz), 39.1, 37.9, 35.9 (d, J = 22.5 Hz), 35.6 (d, J = 2.4 Hz), 32.7, 27.9, 27.2 (d, J = 1.6 Hz), 25.9, 24.0, 22.7, 22.6; ^{19}F-NMR (282 MHz, $CDCl_3$): δ −145.3 (dtd, J = 51.2, 38.2, 12.9 Hz); IR (neat): 2926, 2870, 1468,

1385, 1367, 1152, 1050, 1011, 985, 929, 849; HRMS (EI) *m/z* calcd for $C_{15}H_{27}F$ $[M]^+$: 226.2091; found: 226.2087; $[\alpha]_D^{23}$ +4.1 (c 2.0, $CHCl_3$).

Reaction with **4f** (1084 mg, 3.0 mmol): yield 113 mg (50%); dr 97:3.

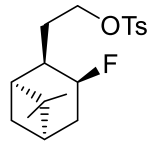

$C_{18}H_{25}FO_3S$
340.45 g/mol

2-((1*S*,2*R*,3*S*,5*R*)-3-Fluoro-6,6-dimethylbicyclo[3.1.1]heptan-2-yl)ethyl 4-methylbenzenesulfonate (2d). Prepared according GP4 from **1d** (320 mg, 1.0 mmol) and **4a** (958 mg, 3.0 mmol); column chromatography (pentane/Et_2O 8.5:1.5) yielded **2d** (190 mg, 56%, dr 95:5) as a colorless and clear oil: R_f 0.25 (heptane/Et_2O 8.5:1.5); ^{1}H-NMR (300 MHz, $CDCl_3$): δ 7.79 (d, J = 8.2 Hz, 2H), 7.35 (d, J = 8.2 Hz, 2H), 4.81 (ddt, J = 51.4, 8.7, 3.4 Hz, 1H), 4.20–3.99 (m, 2H), 2.45 (s, 3H), 2.55–2.30 (m, 2H), 2.20–1.70 (m, 6H), 1.19 (s, 3H), 1.10 (dd, J = 10.2, 1.0 Hz, 1H), 0.78 (s, 3H); ^{13}C-NMR (75 MHz, $CDCl_3$): δ 144.7, 133.1, 129.8, 127.9, 92.2 (d, J = 171.7 Hz), 69.0, 46.2 (d, J = 21.5 Hz), 45.2 (d, J = 5.3 Hz), 40.8 (d, J = 3.1 Hz), 37.8, 35.7 (d, J = 22.3 Hz), 34.4 (d, J = 2.2 Hz), 32.7, 27.0 (d, J = 1.6 Hz), 23.8, 21.6; ^{19}F-NMR (282 MHz, $CDCl_3$): δ −146.6 (dtd, J = 50.3, 38.1, 12.1 Hz); IR (neat): 2922, 1598, 1470, 1358, 1188, 1174, 1096, 973, 954, 913; HRMS (ESI-Orbitrap) *m/z* calcd for $C_{18}H_{25}O_3FSNa$ $[M+Na]^+$: 363.1401; found: 363.1399; $[\alpha]_D^{23}$ +1.1 (c 2.0, $CHCl_3$).

Reaction with **4f** (1084 mg, 3.0 mmol): yield 180 mg (53%); dr 95:5.

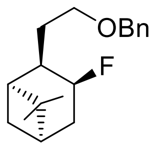

$C_{18}H_{25}FO$
276.40 g/mol

(1*S*,2*R*,3*S*,5*R*)-2-(2-(Benzyloxy)ethyl)-3-fluoro-6,6-dimethylbicyclo-[3.1.1]heptane (2e). Prepared according GP4 from **1e** (256 mg, 1.0 mmol) and **4f** (1084 mg, 3.0 mmol); column chromatography (pentane/DCM 6:4) yielded **2e** (142 mg, 51%, dr 95:5) as a colorless and clear liquid: R_f 0.25 (heptane/DCM 6:4); ^{1}H-NMR (300 MHz, $CDCl_3$): δ 7.44–7.21 (m, 5H), 4.92 (ddt, J = 51.7, 9.0, 3.4 Hz, 1H), 4.59–4.41 (m, 2H), 3.69–3.43 (m, 2H), 2.60–2.45 (m, 1H), 2.43–2.33 (m, 1H), 2.32–2.12 (m, 1H), 2.10–1.83 (m, 4H), 1.80–1.64 (m, 1H), 1.21 (s, 3H), 1.17 (dd, J = 10.1, 1.8 Hz, 1H), 0.84 (s, 3H); ^{13}C-NMR (75 MHz, $CDCl_3$): δ 138.5, 128.3, 127.7, 127.5, 92.9 (d, J = 170.7 Hz), 73.0, 69.1, 46.9 (d, J = 21.1 Hz), 45.7 (d, J = 5.6 Hz), 40.9 (d, J = 2.8 Hz), 37.9, 35.9 (d, J = 22.4 Hz), 34.4 (d, J = 2.5 Hz), 32.6, 27.1 (d, J = 1.6 Hz), 23.9; ^{19}F-NMR (282 MHz, $CDCl_3$): δ −145.3 (dtd, J = 50.9, 37.7, 13.0 Hz); IR (neat): 2917, 2869, 1496, 1472, 1453, 1362, 1204, 1098, 1028, 986, 930; HRMS (ESI-Orbitrap) *m/z* calcd for $C_{18}H_{25}OFNa$ $[M+Na]^+$: 299.1782; found: 299.1381; $[\alpha]_D^{23}$ +3.4 (c 2.0, $CHCl_3$).

Reaction with **4a** (958 mg, 3.0 mmol): yield 106 mg (38%); dr 96:4.

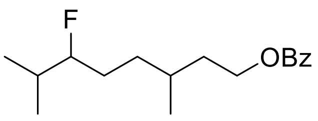

$C_{17}H_{25}FO_2$
280.38 g/mol

6-Fluoro-3,7-dimethyloctyl benzoate (2f). Prepared according GP4 from **1f** (260 mg, 1.0 mmol) and **4f** (1084 mg, 3.0 mmol); column chromatography (heptane/EtOAc 97:3) yielded **2f** (148 mg, 53%) as a colorless and clear oil: R_f 0.25 (heptane/EtOAc 97:3); ^{1}H-NMR (300 MHz, $CDCl_3$): *δ* 8.12–7.97 (m, 2H), 7.62–7.50 (m, 1H), 7.48–7.37 (m, 2H), 4.48–4.32 (m, 2H), 4.30–4.05 (m, 1H), 1.93–1.73 (m, 2H), 1.72–1.52 (m, 4H), 1.51–1.16 (m, 2H), 1.02–0.90 (m, 9H); ^{13}C-NMR (75 MHz, $CDCl_3$): *δ* 166.7, 132.8, 130.5, 129.5, 128.3, 99.9 (d, *J* = 170.8 Hz), 98.8 (d, *J* = 170.7 Hz), 63.4, 35.6, 35.4, 32.6 (d, *J* = 3.6 Hz), 32.5 (d, *J* = 3.4 Hz), 32.3 (d, *J* = 20.2 Hz), 32.2 (d, *J* = 20.3 Hz), 30.0, 29.9, 29.58 (d, *J* = 21.5 Hz), 29.56 (d, *J* = 21.5 Hz), 19.5, 19.4, 18.5 (d, *J* = 5.5 Hz), 18.4 (d, *J* = 5.6 Hz); 17.2 (d, *J* = 6.3 Hz), 17.1 (d, *J* = 6.2 Hz); ^{19}F-NMR (282 MHz, $CDCl_3$): *δ* −187.2 (m); IR (neat): 2960, 2875, 1716, 1603, 1584, 1452, 1389, 1314, 1269, 1175, 1110, 1069, 1026; HRMS (ESI-Orbitrap) *m/z* calcd for $C_{17}H_{25}O_2FNa$ $[M+Na]^+$: 303.1719; found: 303.1730.

Reaction with **4a** (958 mg, 3.0 mmol): yield 128 mg (46%)

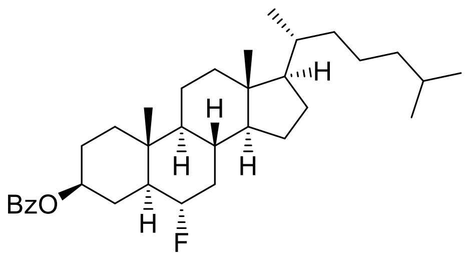

$C_{34}H_{51}FO_2$
510.78 g/mol

(3*S*,5*S*,6*S*,8*S*,9*S*,10*R*,13*R*,14*S*,17*R*)-6-Fluoro-10,13-dimethyl-17-((R)-6-methylheptan-2-yl)hexadeca-hydro-1H-cyclopenta[a]phenanthren-3-yl benzoate (2g). Prepared according GP4 from cholesteryl benzoate **1g** (491 mg, 1.0 mmol) and **4f** (1084 mg, 3.0 mmol); column chromatography (pentane/DCM 6.5:3.5) yielded **2g** (244 mg, 48%) as a white solid: R_f 0.25 (heptane/DCM 6.5:3.5); m.p. 166–168°C; ^{1}H-NMR (300 MHz, $CDCl_3$): *δ* 8.04 (d, *J* = 7.3 Hz, 2H), 7.54 (t, *J* = 7.3 Hz, 1H), 7.42 (t, *J* = 7.3 Hz, 2H), 5.07–4.81 (m, 1H), 4.30 (dtd, *J* = 50.1, 10.7, 4.9 Hz, 1H), 2.38–2.25 (m, 1H), 2.20–2.06 (m, 1H), 2.05–1.91 (m, 2H), 1.90–1.72 (m, 2H), 1.67–0.98 (m, 22H), 0.94–0.80 (m, 12H), 0.79–0.68 (m, 1H), 0.66 (s, 3H); ^{13}C-NMR (75 MHz, $CDCl_3$): *δ* 166.0, 132.7, 130.8, 129.5, 128.2, 91.7 (d, *J* = 171.3 Hz), 73.6, 56.2, 56.0, 53.5 (d, *J* = 1.6 Hz), 49.7 (d, *J* = 15.1 Hz), 42.6, 39.6, 39.5, 38.2 (d, *J* = 17.7 Hz), 37.0, 36.7 (d, *J* = 8.0 Hz), 36.1, 35.8, 34.1 (d, *J* = 11.0 Hz), 28.12, 28.10 (d, *J* = 4.6 Hz), 28.0, 27.2, 24.2, 23.8, 22.8, 22.6, 21.1, 18.7, 13.3, 12.0; ^{19}F-NMR (282 MHz, $CDCl_3$): *δ* −180.4 (d, *J* = 49.9 Hz); IR (neat): 2935, 2864, 1708, 1601, 1470, 1451, 1377, 1314, 1271, 1250, 1161, 1113, 989; HRMS (EI) *m/z* calcd for $C_{34}H_{52}O_2F$ $[M+H]^+$: 511.3946; found: 511.3936; $[\alpha]_D^{23}$ +39.2 (c 2.0, $CHCl_3$).

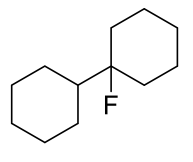

1-Fluoro-1,1'-bi(cyclohexane) (2h). Prepared according GP4 from **1h** (164 mg, 1.0 mmol) and **4f** (1084 mg, 3.0 mmol); reaction time was 20 min (product eliminate in the reaction mixture); column chromatography (pentane) yielded **2h** (126 mg, 68%) as a colorless and clear liquid: R_f 0.50 (heptane); ^{1}H-NMR (300 MHz, $CDCl_3$): δ 1.85–0.95 (m, 21H); ^{13}C-NMR (101 MHz, $CDCl_3$): δ 97.9 (d, J = 170.7 Hz), 47.3 (d, J = 21.6 Hz), 32.2 (d, J = 23.2 Hz), 26.9 (d, J = 6.0 Hz), 26.6, 26.5, 25.7, 21.8 (d, J = 2.5 Hz); ^{19}F-NMR (282 MHz, $CDCl_3$): δ −162.27 (t, J = 35.5 Hz).

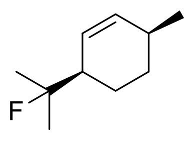

(*3R*,*6S*)-3-(2-Fluoropropan-2-yl)-6-methylcyclohex-1-ene (2i). Prepared according GP4 from (+)-2-carene **1i** (136 mg, 1.0 mmol) and **4f** (1084 mg, 3.0 mmol); column chromatography (pentane) yielded **2i** (104 mg, 67%) as a colorless and clear liquid: R_f 0.30 (heptane); ^{1}H-NMR (300 MHz, $CDCl_3$): δ 5.80–5.70 (m, 1H), 5.67–5.58 (m, 1H), 2.44–2.28 (m, 1H), 2.26–2.11 (m, 1H), 1.80–1.56 (m, 2H), 1.55–1.37 (m, 2H), 1.35 (d, J = 12.4 Hz, 3H), 1.27 (d, J = 12.7 Hz, 3H), 0.99 (d, J = 7.1 Hz, 3H); ^{13}C-NMR (75 MHz, $CDCl_3$): δ 135.0, 125.7 (d, J = 6.9 Hz), 97.8 (d, J = 167.2 Hz), 45.2 (d, J = 21.1 Hz), 28.7, 25.1 (d, J = 24.5 Hz), 23.8 (d, J = 24.9 Hz), 20.9, 20.2 (d, J = 7.1 Hz); ^{19}F-NMR (282 MHz, $CDCl_3$): δ −138.6 (m); IR (neat): 3023, 2982, 2957, 2935, 2870, 1457, 1384, 1372, 1259, 1232, 1190, 1147, 1120, 890; HRMS (EI) *m/z* calcd for $C_{10}H_{17}F$ $[M]^+$: 156.1301; found: 156.1312; $[\alpha]_D^{23}$ −62.3 (c 2.0, $CHCl_3$).

Reaction with **4a** (958 mg, 3.0 mmol): yield 75 mg (48%)

Hydrofluorination of (8a). Prepared according GP4 from **8a** (267 mg, 1.0 mmol) and **4f** (1084 mg, 3.0 mmol); column chromatography (pentane/Et_2O 97:3).

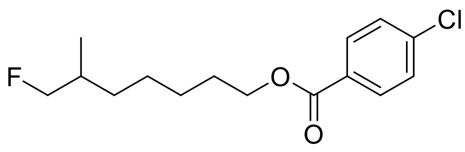

7-Fluoro-6-methylheptyl 4-chlorobenzoate 9a: colorless and clear oil; yield 20 mg (7%); R_f 0.15 (heptane/Et_2O 97:3); ^{1}H-NMR (300 MHz, $CDCl_3$): δ 8.07–7.87 (m, 2H), 7.53–7.31 (m, 2H), 4.37–4.27 (m, 3H), 4.21–4.11 (m, 1H), 1.92–1.71 (m, 3H), 1.58–1.28 (m, 5H), 1.27–1.13 (m, 1H), 0.94 (d, J = 6.8 Hz, 3H); ^{13}C-NMR (75 MHz, $CDCl_3$): δ 165.8, 139.3, 130.9, 128.9, 128.7, 88.3 (d, J = 168.6 Hz), 65.3, 34.0 (d, J = 18.0 Hz), 32.3 (d, J = 5.3 Hz), 28.6, 26.5, 26.3, 15.8 (d, J = 6.5 Hz); ^{19}F-NMR (282 MHz, $CDCl_3$): δ −222.3 (td, J = 48.6, 19.1 Hz); IR (neat): 2933, 2858, 1717, 1595, 1488, 1465, 1402, 1268, 1172, 1114, 1103, 1091, 1015; HRMS (ESI-Orbitrap) *m/z* calcd for $C_{15}H_{20}O_2ClFNa$ $[M+Na]^+$: 309.1028; found: 309.1022.

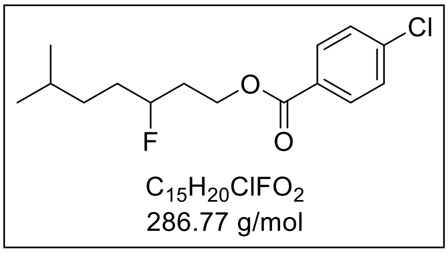

3-Fluoro-6-methylheptyl 4-chlorobenzoate 9a': colorless and clear oil; yield 20 mg (7%); R_f 0.20 (heptane/Et_2O 97:3); ^{1}H-NMR (300 MHz, $CDCl_3$): δ 8.11–7.87 (m, 2H), 7.52–7.33 (m, 2H), 4.82–4.53 (m, 1H), 4.53–4.36 (m, 2H), 2.16–1.92 (m, 2H), 1.83–1.19 (m, 5H), 0.93–0.85 (m, 6H); ^{13}C-NMR (75 MHz, $CDCl_3$): δ 165.6, 139.5, 131.0, 128.75, 128.66, 91.6 (d, J = 168.4 Hz), 61.5 (d, J = 4.7 Hz), 34.4 (d, J = 21.3 Hz), 34.0 (d, J = 4.3 Hz), 33.1 (d, J = 20.5 Hz), 27.9, 22.5, 22.4; ^{19}F-NMR (282 MHz, $CDCl_3$): δ −182.6 (m); IR (neat): 2955, 2870, 1720, 1595, 1489, 1468, 1402, 1268, 1172, 1116, 1102, 1091, 1015; HRMS (ESI-Orbitrap) *m/z* calcd for $C_{15}H_{20}O_2ClFNa$ $[M+Na]^+$: 309.1028; found: 309.1027.

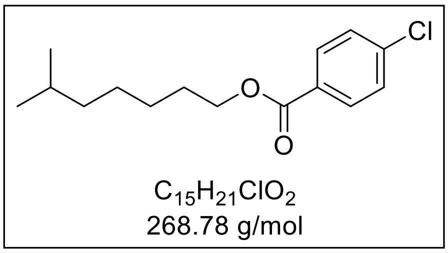

6-Methylheptyl 4-chlorobenzoate 10a: colorless and clear oil; yield 134 mg (50%); R_f 0.35 (heptane/Et_2O 97:3); ^{1}H-NMR (300 MHz, $CDCl_3$): δ 8.06–7.91 (m, 2H), 7.54–7.36 (m, 2H), 4.31 (t, J = 6.7 Hz, 2H), 1.83–1.68 (m, 2H), 1.61–1.48 (m, 1H), 1.46–1.29 (m, 4H), 1.24–1.14 (m, 2H), 0.87 (d, J = 6.6 Hz, 6H); ^{13}C-NMR (75 MHz, $CDCl_3$): δ 165.8, 139.2, 130.9, 129.0, 128.7, 65.4, 38.9, 28.7, 27.9, 27.1, 26.3, 22.6; IR (neat): 2953, 2927, 2868, 1719, 1595, 1488, 1467, 1402, 1267, 1171, 1113, 1103, 1091, 1015; HRMS (ESI-Orbitrap) *m/z* calcd for $C_{15}H_{21}O_2ClNa$ $[M+Na]^+$: 291.1122; found: 291.1120.

Reaction with **4a** (958 mg, 3.0 mmol): yield: **9a** 20 mg (7%), **9a'** 18 mg (6%) and **10a** 91 mg (34%).

Reaction with **4f** (1084 mg, 3.0 mmol) in DMF-d7 (2 mL): yield: **9a** 34 mg (12%), **9a'** 31 mg (11%) and **10a** 78 mg (29%).

Hydrofluorination of (8b). Prepared according GP4 from **8b** (281 mg, 1.0 mmol) and **4f** (1084 mg, 3.0 mmol); purification with the CombiFlash (Eluent: 0–1 min pentane/Et_2O 99:1, 1–16 min to pentane/Et_2O 96:4, 16–21 min pentane/Et_2O 96:4).

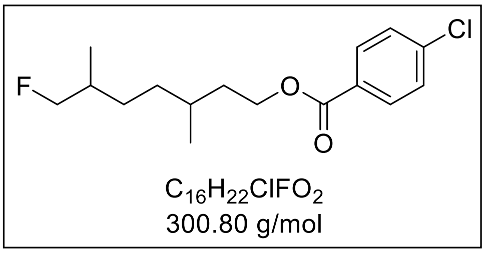

7-Fluoro-3,6-dimethylheptyl 4-chlorobenzoate 9b: colorless and clear oil; yield 10 mg (4%); R_f 0.30 (heptane/Et_2O 96:4); ^{1}H-NMR (300 MHz, $CDCl_3$): δ 8.08–7.84 (m, 2H), 7.50–7.33 (m, 2H), 4.40–4.33 (m, 2H), 4.33–4.12 (m, 2H), 1.91–1.68 (m, 2H), 1.66–1.54 (m, 2H), 1.52–1.12 (m, 4H), 1.04–0.89 (m, 6H); ^{13}C-NMR (75 MHz, $CDCl_3$): δ 165.8, 139.3, 130.9, 128.9, 128.7, 88.4 (d, J = 168.8 Hz), 88.3 (d, J

= 168.7 Hz), 63.72, 63.71, 35.6, 35.4, 34.33 (d, *J* = 18.0 Hz), 34.27 (d, *J* = 18.0 Hz), 34.1, 34.0, 30.3, 30.2, 29.7 (d, *J* = 5.6 Hz), 29.6 (d, *J* = 5.6 Hz), 19.6, 19.4, 15.9 (d, *J* = 6.6 Hz), 15.7 (d, *J* = 6.6 Hz); ^{19}F-NMR (282 MHz, $CDCl_3$): δ −222.3 (m); IR (neat): 2958, 2827, 1717, 1595, 1488, 1460, 1401, 1268, 1172, 1114, 1103, 1091, 1015; HRMS (ESI-Orbitrap) *m/z* calcd for $C_{16}H_{22}O_2ClFNa$ [M+Na]$^+$: 323.1185; found: 323.1175.

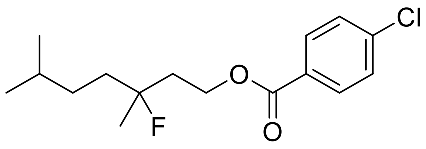

$C_{16}H_{22}ClFO_2$
300.80 g/mol

3-Fluoro-3,6-dimethylheptyl 4-chlorobenzoate 9b': colorless and clear oil; yield 90 mg (30%); R_f 0.35 (heptane/Et_2O 96:4); ^{1}H-NMR (300 MHz, $CDCl_3$): δ 8.11–7.84 (m, 2H), 7.53–7.32 (m, 2H), 4.47 (t, *J* = 7.0 Hz, 2H), 2.23–1.96 (m, 2H), 1.74–1.61 (m, 2H), 1.53 (sept, *J* = 6.6 Hz, 1H), 1.39 (d, *J* = 21.8 Hz, 3H), 1.32–1.23 (m, 2H), 0.90 (d, *J* = 6.6 Hz, 3H), 0.89 (d, *J* = 6.6 Hz, 3H); ^{13}C-NMR (75 MHz, $CDCl_3$): δ 165.7, 139.4, 131.0, 128.73, 128.70, 96.2 (d, *J* = 168.3 Hz), 61.1 (d, *J* = 6.3 Hz), 38.0 (d, *J* = 22.8 Hz, 2C), 32.6 (d, *J* = 5.6 Hz), 28.3, 24.6 (d, *J* = 24.9 Hz), 22.54, 22.50; ^{19}F-NMR (282 MHz, $CDCl_3$): δ −144.3 (m); IR (neat): 2955, 2869, 1719, 1595, 1489, 1468, 1401, 1269, 1172, 1115, 1103, 1091, 1014; HRMS (ESI-Orbitrap) *m/z* calcd for $C_{16}H_{22}O_2ClFNa$ [M+Na]$^+$: 323.1185; found: 323.1178.

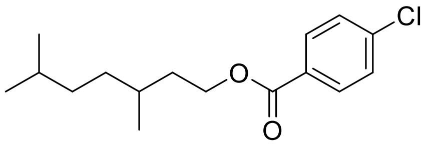

$C_{16}H_{23}ClO_2$
282.81 g/mol

3,6-Dimethylheptyl 4-chlorobenzoate 10b: colorless and clear oil; yield 95 mg (34%); R_f 0.50 (heptane/Et_2O 96:4); ^{1}H-NMR (300 MHz, $CDCl_3$): δ 8.14–7.84 (m, 2H), 7.54–7.29 (m, 2H), 4.45–4.28 (m, 2H), 1.87–1.74 (m, 1H), 1.65–1.43 (m, 3H), 1.41–1.14 (m, 4H), 0.95 (d, *J* = 6.3 Hz, 3H), 0.88 (dd, *J* = 6.6, 1.4 Hz, 6H); ^{13}C-NMR (75 MHz, $CDCl_3$): δ 165.8, 139.2, 130.9, 129.0, 128.7, 63.9, 36.2, 35.6, 34.7, 30.3, 28.3, 22.8, 22.6, 19.7; IR (neat): 2954, 2925, 2869, 1719, 1595, 1488, 1465, 1401, 1268, 1171, 1114, 1102, 1091, 1015; HRMS (ESI-Orbitrap) *m/z* calcd for $C_{16}H_{23}O_2ClNa$ [M+Na]$^+$: 305.1279; found: 305.1276.

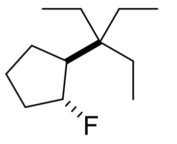

$C_{12}H_{23}F$
186.31 g/mol

***trans*-1-(3-Ethylpentan-3-yl)-2-fluorocyclopentane 12**. Prepared according GP4 from **11** (166 mg, 1.0 mmol) and **4f** (1084 mg, 3.0 mmol); column chromatography (pentane) yielded **12** (126 mg, 68%, *trans*/*cis* 98:2) as a colorless and clear liquid: R_f 0.40 (heptane); ^{1}H-NMR (300 MHz, $CDCl_3$): δ 5.24–4.95 (m, 1H), 2.22–1.85 (m, 2H), 1.81–1.50 (m, 4H), 1.33 (q, *J* = 7.5 Hz, 7H), 0.82 (t, *J* = 7.5 Hz, 9H); ^{13}C-NMR (75 MHz, $CDCl_3$): δ 97.5 (d, *J* = 170.5 Hz), 54.3 (d, *J* = 19.1 Hz), 38.2 (d, *J* = 3.6 Hz), 34.6 (d, *J* = 23.5 Hz), 27.7, 27.2 (d, *J* = 4.2 Hz), 23.7, 8.4; ^{19}F-NMR (282 MHz, $CDCl_3$): δ −160.1 (m); IR (neat): 2961, 2880, 1465, 1433, 1381, 1169, 1019, 991, 957, 903; $C_{12}H_{22}$[M−HF]$^+$: 166.1716; found: 166.1712.

4-((1S,2R)-2-Fluorocyclopentyl)-1,1'-biphenyl (2b)

According to the literature procedure[10], to a suspension of **1b** (220 mg, 1.0 mmol) in THF (1.6 mL) was added (+)-IpcBH_2 (0.768 M in diethylether, 1.58 mL, 1.2 mmol) at −78 °C. The mixture was stirred at −25 °C for 4 h and stored in the freezer at −25 °C for 3 d. Acetaldehyde (0.34 mL, 6.0 mmol) was added at −40 °C to the reaction mixture and stirred at 0 °C for 10 min and at room temperature for 7 h. Catechol (143 mg, 1.3 mmol) was added at 0 °C and stirred at room temperature overnight. The reaction mixture was evaporated and dried under high vacuum. The residue was dissolved in dry DMF (2 mL) and **4f** (1084 mg, 3.0 mmol) and DTBPO (117 mg, 0.50 mmol) were added. The reaction mixture was stirred at 60 °C for 45 min (preheated oil bath). The crude product was purified by column chromatography (pentane/DCM 8.5:1.5) to yield **2b** (126 mg, 52%, *trans*/*cis* 88:12) as a white solid. The two diastereomers were separated by column chromatography (pentane/DCM 9:1).

(1S,2R)-**2b** (*trans*, major): $[\alpha]_D^{23}$ −21.0 (c 2.0, $CHCl_3$); ^{1}H– and ^{13}C–NMR-spectra correspond to **2b** obtained by racemic hydrofluorination; *er* 91:9 (CHIRALPAK IB-3; hexane/*i*PrOH 99.2:0.8; 1 mL min^{-1}, λ = 250 nm.

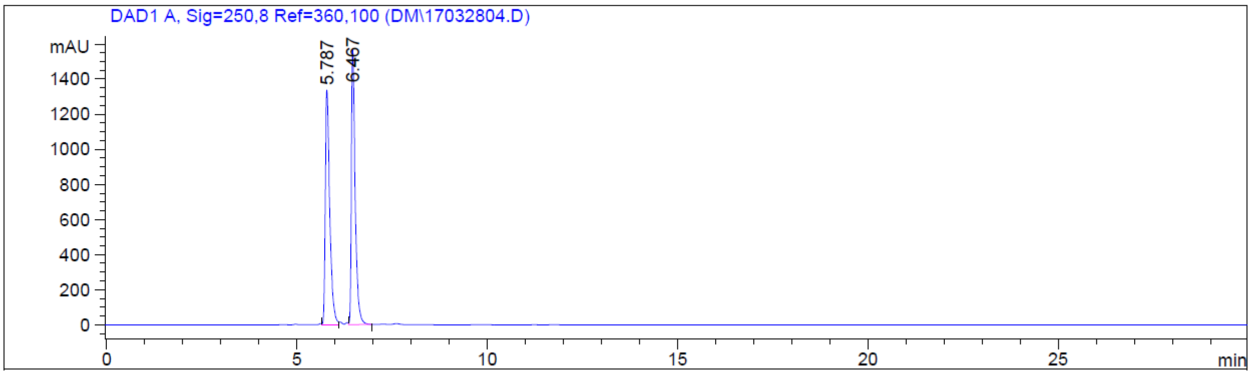

Signal 1: DAD1 A, Sig=250,8 Ref=360,100

Peak #	RetTime [min]	Type	Width [min]	Area [mAU*s]	Height [mAU]	Area %
1	5.787	VV	0.1213	1.09177e4	1336.94934	50.2234
2	6.467	VB	0.1027	1.08206e4	1564.63245	49.7766

Totals : 2.17383e4 2901.58179

Supplementary Figure 6. Determination of the enantiomeric ratio of *trans*-**2b** (racemic sample).

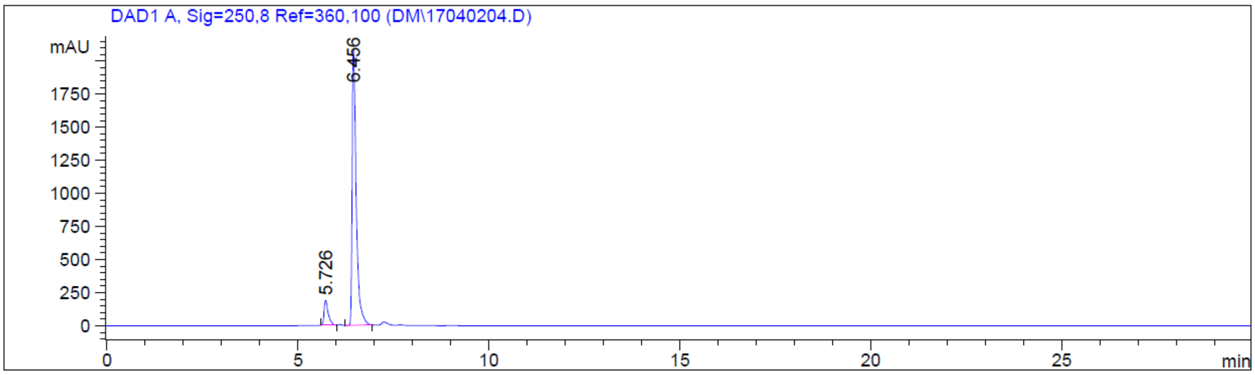

Signal 1: DAD1 A, Sig=250,8 Ref=360,100

Peak #	RetTime [min]	Type	Width [min]	Area [mAU*s]	Height [mAU]	Area %
1	5.726	BB	0.1136	1460.68518	190.18463	8.6327
2	6.456	VB	0.1105	1.54597e4	2084.68799	91.3673
Totals :				1.69204e4	2274.87262	

Supplementary Figure 7. Determination of the enantiomeric ratio of *trans*-**2b** (enantiomerically enriched sample).

(1*S*,2*S*)-2b (*cis*, minor): $[\alpha]_D^{23}$ +60.6 (c 1.0, $CHCl_3$). ^{1}H– and ^{13}C–NMR-spectra correspond to **2b** obtained by racemic hydrofluorination; *er* 91:9 (CHIRALPAK IB-3; hexane/*i*PrOH 99.2:0.8; 1 mL min^{-1}, λ = 250 nm.

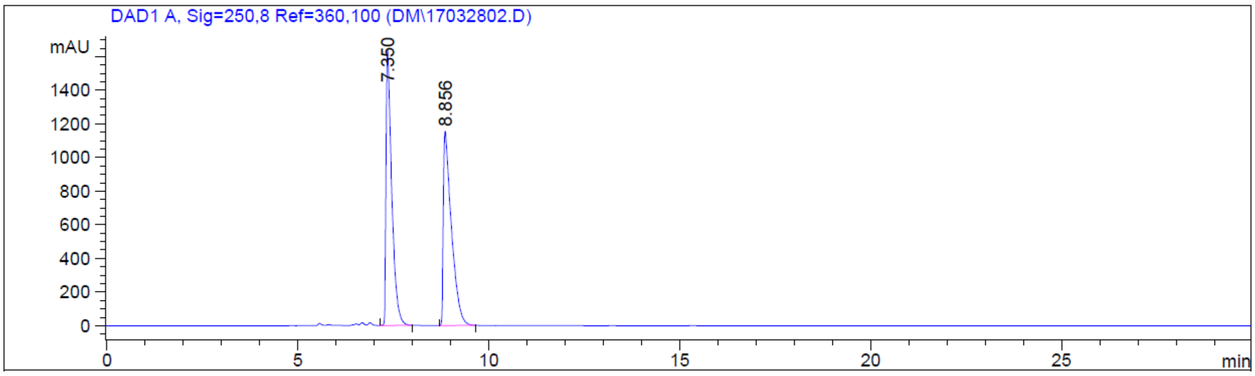

Signal 1: DAD1 A, Sig=250,8 Ref=360,100

Peak #	RetTime [min]	Type	Width [min]	Area [mAU*s]	Height [mAU]	Area %
1	7.350	BB	0.1513	1.70330e4	1638.11682	50.0722
2	8.856	BB	0.2075	1.69838e4	1155.55859	49.9278
Totals :				3.40168e4	2793.67542	

Supplementary Figure 8. Determination of the enantiomeric ratio of *cis*-**2b** (racemic sample).

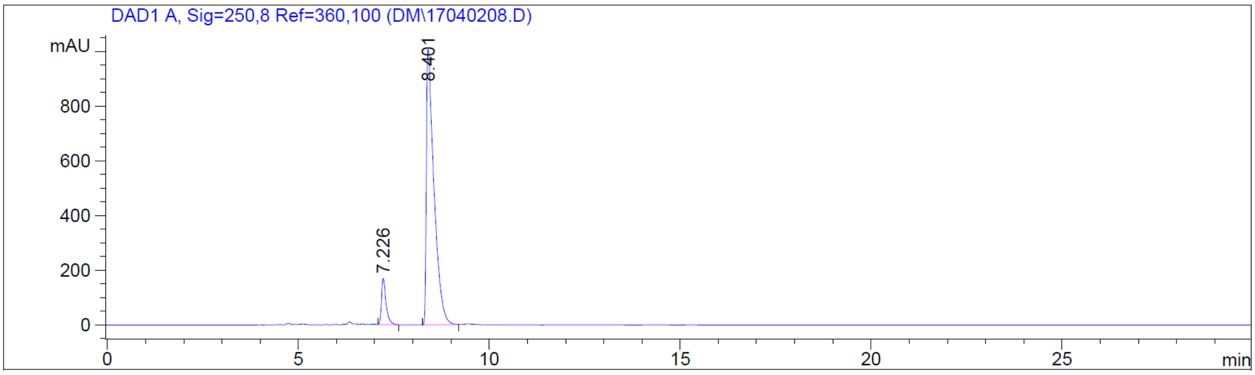

Signal 1: DAD1 A, Sig=250,8 Ref=360,100

Peak #	RetTime [min]	Type	Width [min]	Area [mAU*s]	Height [mAU]	Area %
1	7.226	BB	0.1220	1389.59277	168.89984	9.0550
2	8.401	BB	0.1955	1.39565e4	1008.30200	90.9450

Totals : 1.53461e4 1177.20184

Supplementary Figure 9. Determination of the enantiomeric ratio of *cis*-**2b** (enantiomerically enriched sample).

Kinetic Experiments

(Z)-2-(cyclooct-4-en-1-yl)benzo[d][1,3,2]dioxaborole (5)

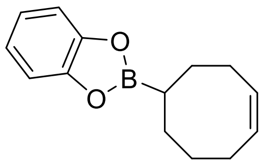

$C_{14}H_{17}BO_2$
228.10 g/mol

According to the literature procedure[25], catecholborane (2.64 g, 22 mmol) was added to 1,5-cycloocatdiene (16.66 g, 154 mmol). The neat solution was heated at 100 °C for 20 h. The excess of 1,5-cyclooctadiene was removed under high vacuum and the residue was quickly distilled under reduced pressure (2 × 10^{-2} mbar) to yield **5** (2.68 g, corrected yield: 43%) along with 20% of an undetermined byproduct: ^{1}H-NMR (300 MHz, C_6H_6): *δ* 7.08–6.97 (m, 2H), 6.84–6.75 (m, 2H), 5.75–5.56 (m, 2H), 2.30–2.18 (m, 1H), 2.08–1.97 (m. 3H), 1.88–1.25 (m, 7H); ^{13}C-NMR (75 MHz, C_6H_6): *δ* 148.6, 130.5, 129.8, 122.3, 112.1, 30.2, 29.2, 27.6, 25.9, 25.7, 20.7 (broad).

Competitive cyclization/fluorination of 5 with 4a, 4f and NFSI

F-Reagent (1.5 mmol) and DTBHN (44 mg, 0.25 mmol) were dissolved in dry DMF (1 mL). Alkylcatechlborane **5** (80% purity, 143 mg, 0.5 mmol) was added and the mixture was heated to 80 °C (preheated oil bath) and stirred at this temperature for 30 min. The ratio of **6**/**7** was determined by GC-FID.

Preparative fluorination of 5

Reagent **4f** (4.34 g, 12.0 mmol) and DTBHN (348 mg, 2.0 mmol) were dissolved in dry DMF (8 mL). Alkylcatechlborane **5** (80% purity, 1140 mg, 4.0 mmol) was added and the mixture was heated to 80 °C (preheated oil bath) and stirred at this temperature for 30 min. The crude product was purified by column chromatography (pentane) to yield **6** and **7** (dr 80:20).

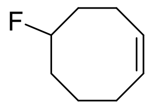

$C_8H_{13}F$
128.19 g/mol

6: colorless and clear liquid; yield 160 mg (31%); R_f 0.40 (heptane); ^{1}H-NMR (300 MHz, $CDCl_3$): δ 5.78–5.50 (m, 2H), 4.79–4.44 (m, 1H), 2.44–2.24 (m, 1H), 2.21–1.83 (m, 6H), 1.80–1.60 (m, 2H), 1.58–1.42 (m, 1H); ^{13}C-NMR (75 MHz, $CDCl_3$): δ 129.7, 129.4, 95.8 (d, J = 163.1 Hz), 34.6 (d, J = 23.8 Hz), 34.0 (d, J = 22.3 Hz), 25.4, 24.0 (d, J = 11.7 Hz), 21.6 (d, J = 13.5 Hz); ^{19}F-NMR (282 MHz, $CDCl_3$): δ −160.7 (m); IR (neat): 3018, 2933, 2859, 1468, 1453, 1370, 1103, 972, 938; HRMS (EI) m/z calcd for $C_8H_{13}F$ $[M]^+$: 128.0996; found: 128.0995.

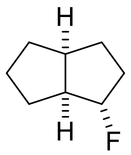

$C_8H_{13}F$
128.19 g/mol

7: colorless and clear liquid, yield 113 mg (22%); R_f major 0.50 and minor 0.55 (heptane); ^{1}H-NMR (300 MHz, $CDCl_3$): δ 5.04–4.85 (m, 0.20H), 4.85–4.64 (m, 0.80H), 2.76–2.42 (m, 2H), 1.99–1.03 (m, 11H); ^{13}C-NMR (101 MHz, $CDCl_3$): δ 101.7 (d, J = 172.2 Hz), (97.4 (d, J = 178.1 Hz)), 50.4 (d, J = 19.7 Hz), (47.7 (d, J = 18.3 Hz)), (43.2), 41.7, (34.7 (d, J = 21.8 Hz)), 34.4 (d, J = 0.9 Hz), (33.6), 31.6 (d, J = 21.5 Hz), 30.7 (d, J = 9.8 Hz), 29.9 (d, J = 1.2 Hz), (29.7), (27.5 (d, J = 1.5 Hz)), 27.0 (d, J = 1.5 Hz), (26.1 (d, J = 11.3 Hz)); ^{19}F-NMR (282 MHz, $CDCl_3$): δ −170.3 (m), (−190.0 (m)); IR (neat): 2944, 2862, 1451, 1353, 1190, 1107, 1040, 963, 931; HRMS (EI) m/z calcd for $C_8H_{13}F$ $[M]^+$: 128.0996; found: 128.0996.

Decarboxylative Fluorination

Optimization of decarboxylative Fluorination

According to the literature procedures[16,26], to a solution of perester **13a** (99 mg, 0.3 mmol) and *n*-undecane (63 µL, 0.3 mmol) in chlorobenzene (0.2–0.3 M) was added **4a** (1.5–5 equiv). The reaction mixture was heated to 100–110 °C (preheated oil bath) and stirred at this temperature for 1–2 h.

Supplementary Table 2. Optimization of the decarboxylative fluorination

Entry	**4a** [equiv]	Temp. [°C]	Time [h]	M [mmol/mL]	Yield [%]
1	3	100	2	0.2	47
2	5	100	2	0.2	45
3	3	110	1	0.2	47

4	5	110	1	0.2	46
5	2	110	1	0.2	47
6	1.5	110	1	0.2	42
7	2	110	1	0.3	45

General Procedure 5 (GP5): Decarboxylative Fluorination

To a solution of perester **13** (1.0 mmol) in chlorobenzene (5 mL) was added F-reagent (2.0 mmol). The reaction mixture was heated to 110 °C (preheated oil bath) and stirred at this temperature for 0.25–1 h. The crude product was purified by column chromatography. Fluorides **14a** and **14b** were contaminated with chlorobenzene which could be removed by azeotropic distillation with 2-propanol on the rotavap (10 ml and 2 × 5 mL were added and evaporated).

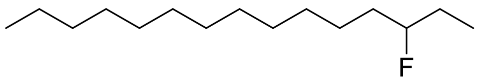

$C_{15}H_{31}F$
230.41 g/mol

3-Fluoropentadecane 14a. Prepared according GP5 from **13a** (329 mg, 1.0 mmol) and **4a** (639 mg, 2.0 mmol); reaction at 110 °C for 1 h; column chromatography (pentane) yielded **14a** (110 mg, 48%) as a colorless and clear liquid: R_f 0.55 (heptane); ^{1}H-NMR (300 MHz, $CDCl_3$): δ 4.56–4.25 (m, 1H), 1.73–1.42 (m, 5H), 1.40–1.21 (m, 19H), 0.96 (t, J = 7.4 Hz, 3H), 0.88 (t, J = 6.7 Hz, 3H); ^{13}C-NMR (75 MHz, $CDCl_3$): δ 95.8 (d, J = 166.9 Hz), 34.7 (d, J = 20.9 Hz), 32.0, 29.71, 29.68 (2C), 29.62, 29.58, 29.56, 29.4, 28.1 (d, J = 21.5 Hz), 25.2 (d, J = 4.5 Hz), 22.7, 14.1, 9.4 (d, J = 5.8 Hz); ^{19}F-NMR (282 MHz, $CDCl_3$): δ −181.2 (m).

Reaction with **4f** (723 mg, 2.0 mmol): yield 108 mg (47%).

Reaction with **NFSI** (631 mg, 2.0 mmol): yield 17 mg (7%).

Reaction with **Selectfluor®** (709 mg, 2.0 mmol) in chlorobenzene/DMPU 1:1: yield <2% according GC-FID.

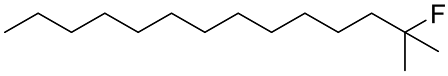

$C_{15}H_{31}F$
230.41 g/mol

2-Fluoro-2-methyltetradecane 14b. Prepared according GP5 from **13b** (329 mg, 1.0 mmol) and **4a** (639 mg, 2.0 mmol); reaction at 110 °C for 0.25 h; GC analysis of the crude product shows the presence of **14b** (46%), 2-methyltetradec-2-ene (6%) and 2-methyltetradec-1-ene (29%); column chromatography (pentane) yielded **14b** (105 mg, 46%) as a colorless and clear liquid: R_f 0.50

(heptane); ^{1}H-NMR (300 MHz, $CDCl_3$): δ 1.66–1.53 (m, 2H), 1.49–1.10 (m, 26H), 0.88 (t, J = 6.3 Hz, 3H); ^{13}C-NMR (75 MHz, $CDCl_3$): δ 95.9 (d, J = 163.9 Hz), 41.5 (d, J = 22.7 Hz), 31.9, 30.0, 29.7 (2C), 29.61, 29.60, 29.4, 26.6 (d, J = 24.9 Hz, 2C), 24.0 (d, J = 5.3 Hz), 22.7, 14.1; ^{19}F-NMR (282 MHz, $CDCl_3$): δ −136.9 (m); IR (neat): 2979, 2923, 2853, 1467, 1384, 1372, 1256, 1146, 876; HRMS (EI) *m/z* calcd for $C_{15}H_{30}$ $[M-HF]^+$: 210.2342; found: 210.2341.

Reaction with **4f** (723 mg, 2.0 mmol) afforded **14b** (109 mg, 47%).

Reaction with NFSI (631 mg, 2.0 mmol) afforded only traces of **14b** (3%) according to GC analysis. Isolation of **14b** was not attempted.

Gas chromatography analysis of the crude reaction products obtained with ***4a*** *and NFSI*

The crude reaction mixtures were analyzed by GC (50 °C (1 min) to 280 °C (15 min); 30 °C/min) using *n*-undecane as an internal standard to determine the yields.

Reaction with ***4a***. The major product was the fluoride **14b** (46%) accompanied by 2-methyltetradec-2-ene (6%) and 2-methyltetradec-1-ene (29%).

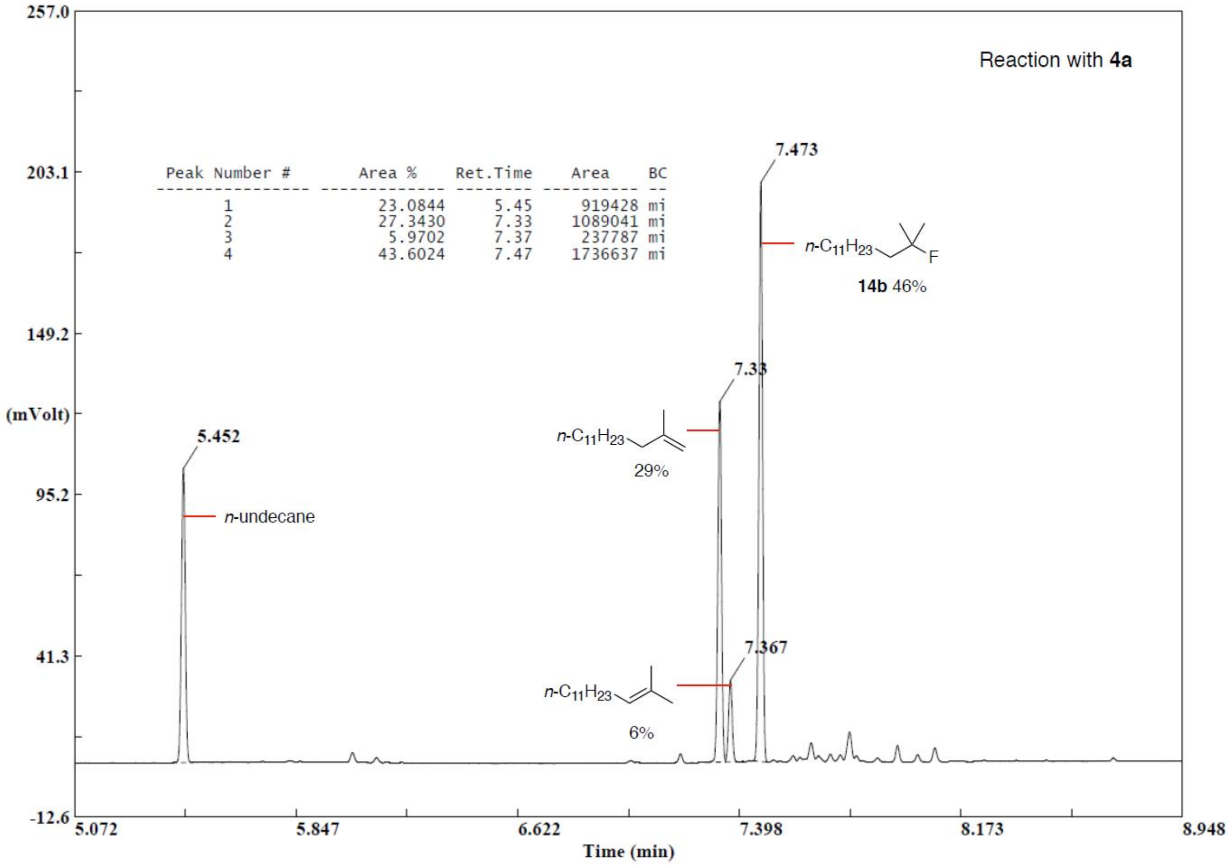

Supplementary Figure 10. GC trace for the reaction of **13b** with **4a**.

Reaction with NFSI. The presence of **14b** (<3%) could be detected together with 2-methyltetradec-2-ene (55%) and 2-methyltetradec-1-ene (9%). Other impurities could not be identified.

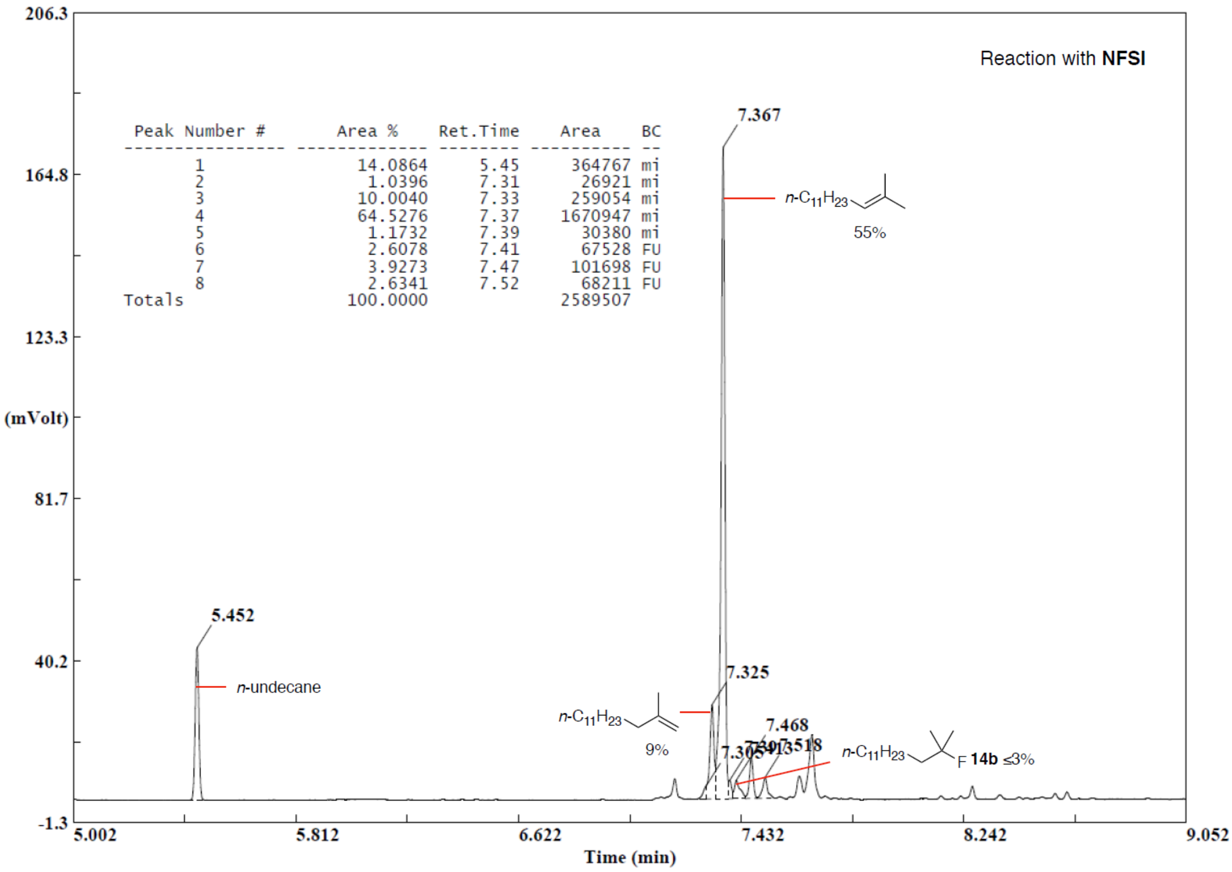

Supplementary Figure 11. GC trace for the reaction of **13b** with NFSI.

Discussion. For the reaction with **4a**, the prevalence of the less substituted terminal alkene over the non-terminal alkene suggests that a radical cross-disproportionation between the alkyl radical and the amidyl radical may be occurring. For NFSI, the prevalence of the more substituted alkene supports a carbocationic or an acid catalyzed elimination process. Since the defluorinated sulfonamides were identified as by-product during the decarboxylation reaction, catalysis of HF elimination by the acidic bis(benzenesulfonyl)imide was envisaged. This process was ruled out since treatment of the tertiary fluoride **14b** with bis(benzenesulfonyl)imide in chlorobenzene at 110 °C for 15 minutes did not led to any formation of the alkene. However, it was shown that under these reaction conditions, aqueous HF was promoting a clean elimination leading to a 85:15 mixture of 2-methyltetradec-2-ene and 2-methyltetradec-1-ene.

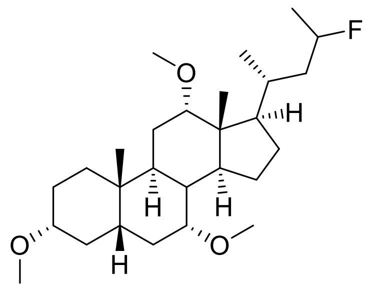

$C_{27}H_{47}FO_3$
438.69 g/mol

(3*R*,5*S*,7*R*,9*S*,10*S*,12*S*,13*R*,14*S*,17*R*)-17-((2*R*)-4-Fluoropentan-2-yl)-3,7,12-trimethoxy-10,13-dimethylhexadecahydro-1H-cyclopenta[a]phenanthrene 14c. Prepared according GP5 from **13c** (215 mg, 0.4 mmol) and **4a** (255 mg, 0.8 mmol); reaction at 110 °C for 1 h in dry ACN (2 mL) in a sealed microwave-tube; column chromatography with the CombiFlash (pentane/Et_2O 8:2) yielded **14c** (68 mg, 39%, dr NMR (300 MHz, $CDCl_3$): δ 4.94–4.57 (m, 1H), 3.43–3.34 (m, 1H), 3.33 (s, 3H), 3.26 (s, 3H), 3.21 (s, 3H), 3.17–3.10 (m, 1H), 3.06–2.92 (m, 1H), 2.20 (dd, *J* = 22.9, 11.2 Hz, 1H), 2.13–2.00 (m, 2H), 1.98–1.43 (m, 12H), 1.39–1.11 (m, 8H), 1.09–0.87 (m, 8H), 0.67 (d, *J* = 4.9 Hz, 3H); ^{13}C-NMR (75 MHz, $CDCl_3$): δ 90.8 (d, *J* = 162.6 Hz), 88.5 (d, *J* = 163.6 Hz), 82.05, 81.98, 80.7, 77.0, 76.9, 55.8, 55.7, 55.4, 47.2, 47.0, 46.2, 46.1, 43.5 (d, *J* = 20.8 Hz), 43.2 (d, *J* = 19.0 Hz), 42.73, 42.68, 42.0, 39.6, 35.3, 34.9, 34.4, 33.9 (d, *J* = 6.5 Hz), 32.0 (d, *J* = 1.6 Hz), 28.0, 27.79, 27.77, 27.5, 26.7, 23.1, 22.9, 21.98 (d, *J* = 22.7 Hz), 21.97, 21.3 (d, *J* = 23.0 Hz), 18.4, 17.7, 12.5, 12.4; ^{19}F-NMR (282 MHz, $CDCl_3$): δ −168.3 (m), −174.3 (m).

Reaction with **4f** (289 mg, 0.8 mmol): yield 58 mg (33%); dr 1:1.

Reaction with **NFSI** (252 mg, 0.8 mmol): yield 40 mg (23%); dr 1:1.

NMR-Spectra

See following pages

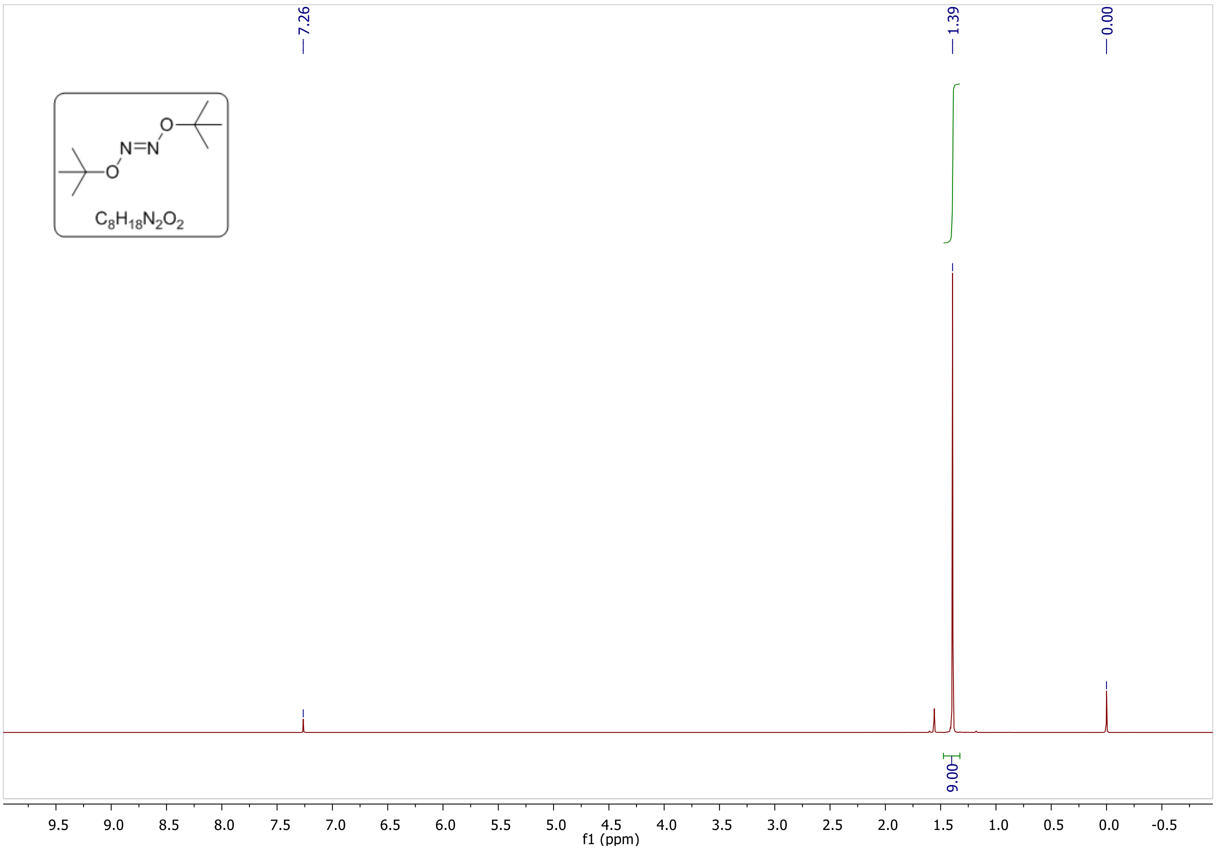

Supplementary Figure 12. ^{1}H-NMR (300 MHz) of di-*tert*-butylhyponitrite.

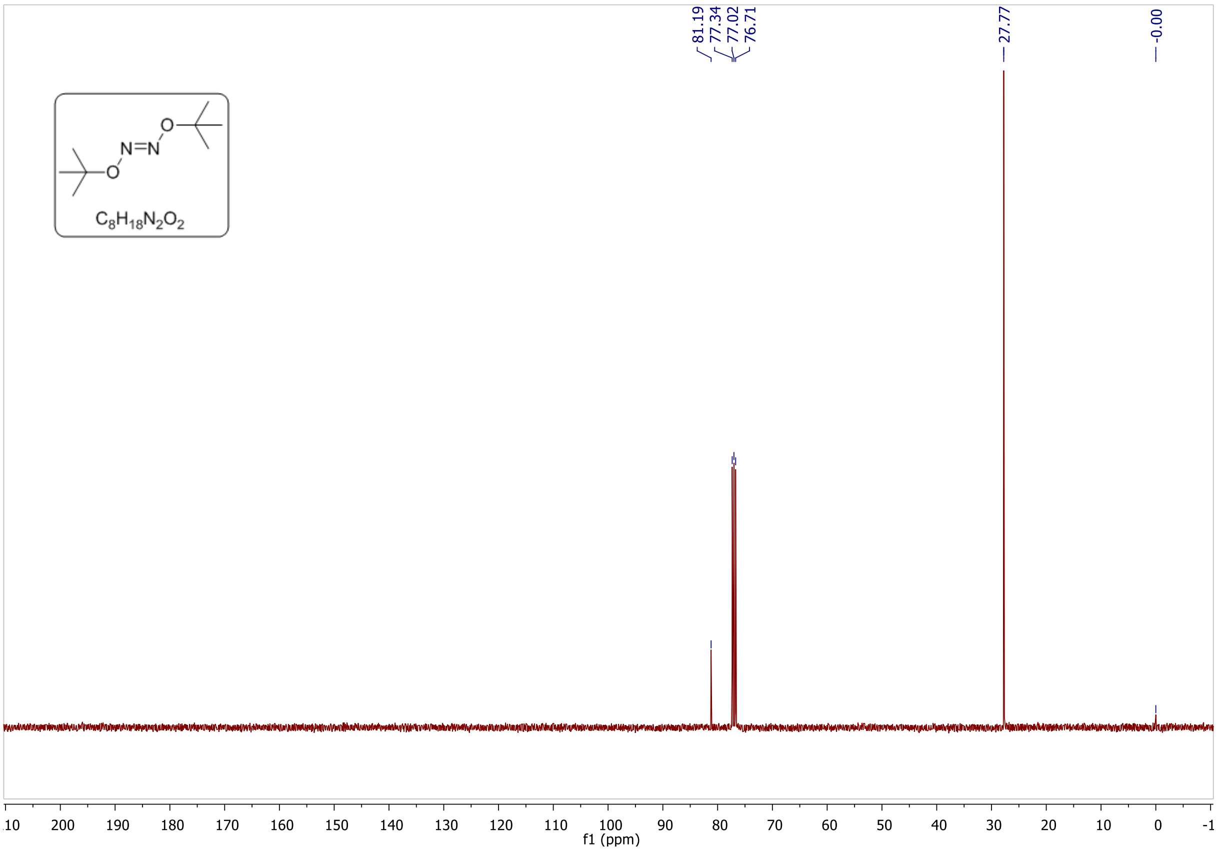

Supplementary Figure 13. ^{13}C-NMR (75 MHz) of di-*tert*-butylhyponitrite.

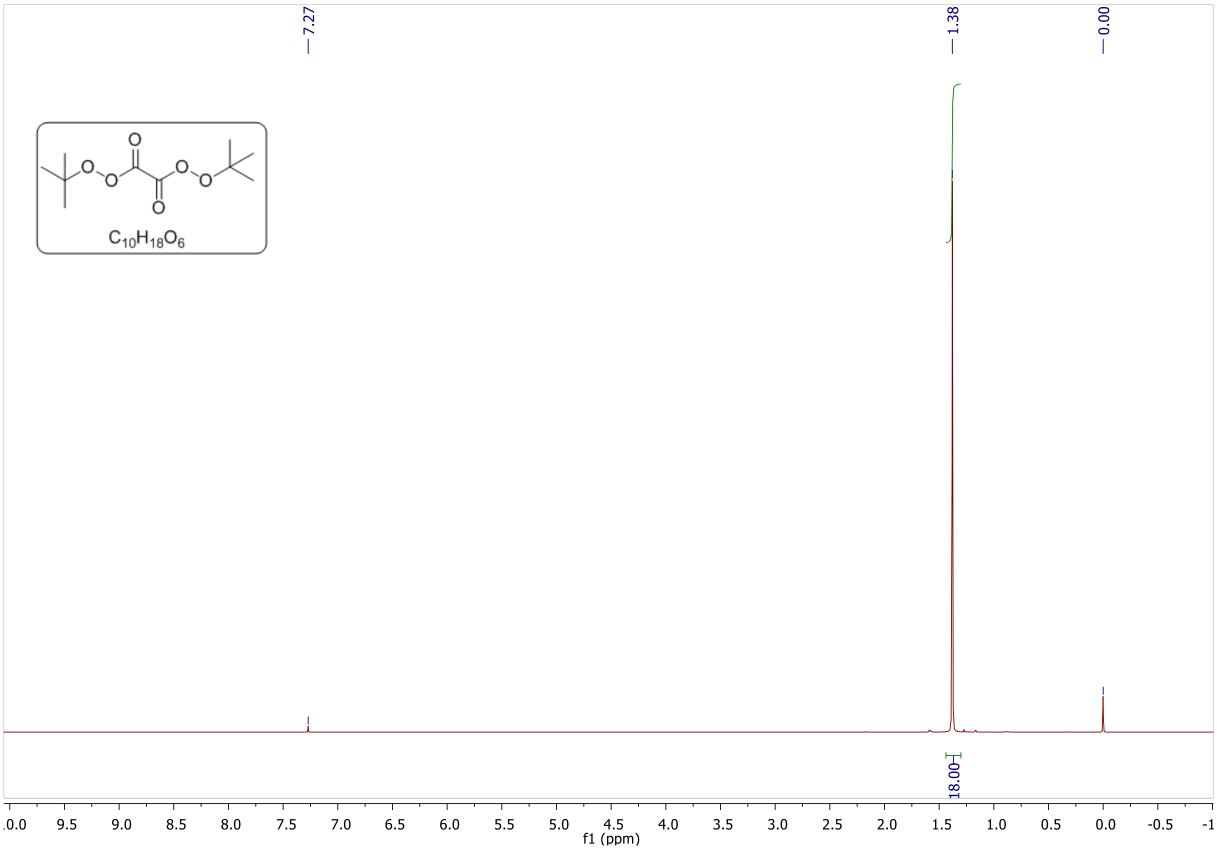

Supplementary Figure 14. ^{1}H-NMR (300 MHz) of di-*tert*-butyl peroxyoxalate.

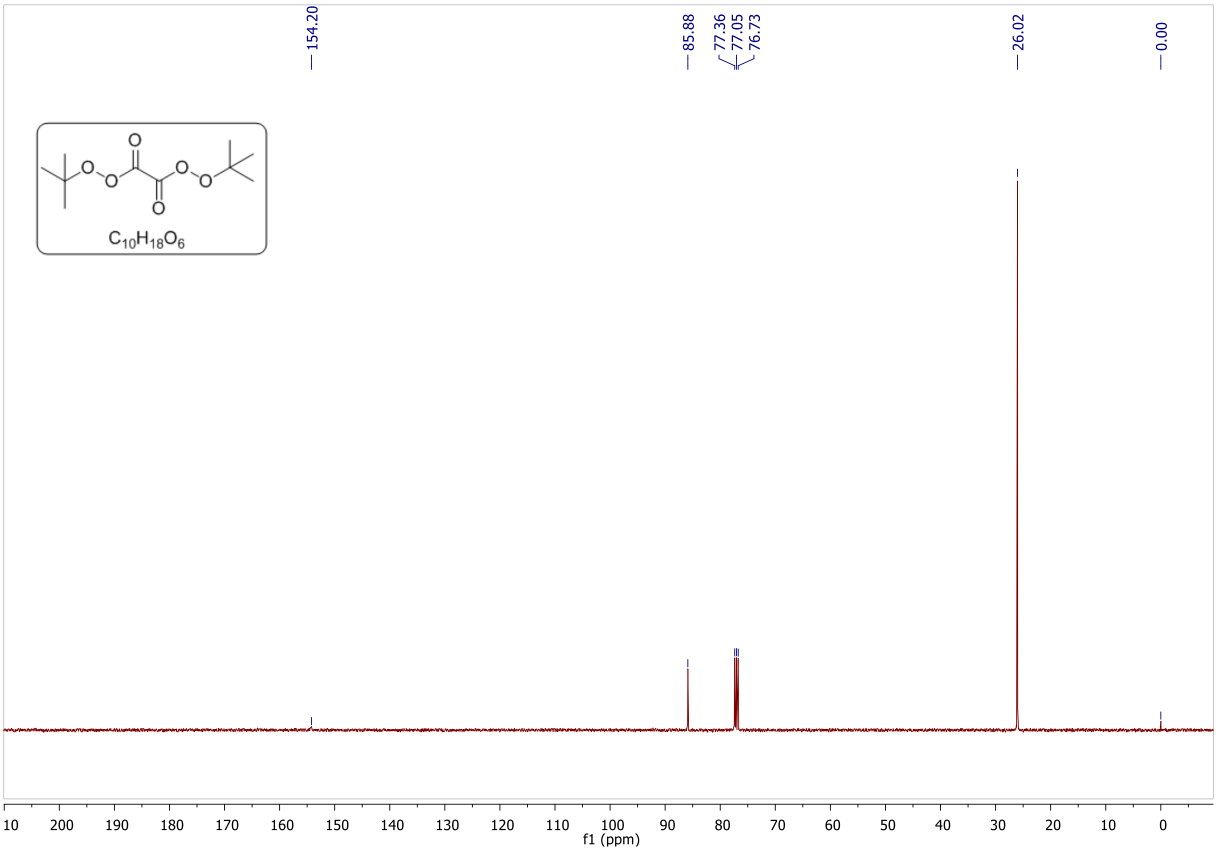

Supplementary Figure 15. ^{13}C-NMR (101 MHz) of di-*tert*-butyl peroxyoxalate.

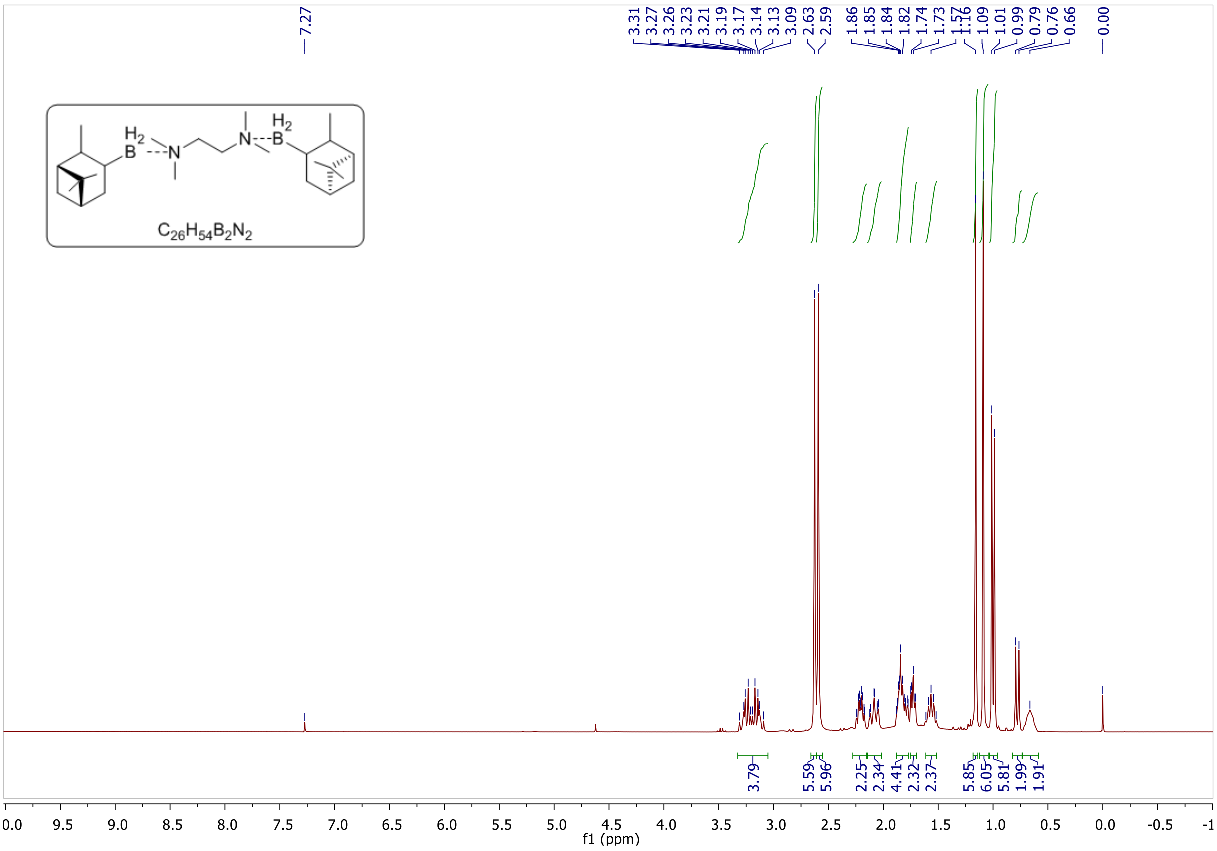

Supplementary Figure 16. ^{1}H-NMR (300 MHz) of (+)-(IpcBH$_2$)$_2$·TMEDA.

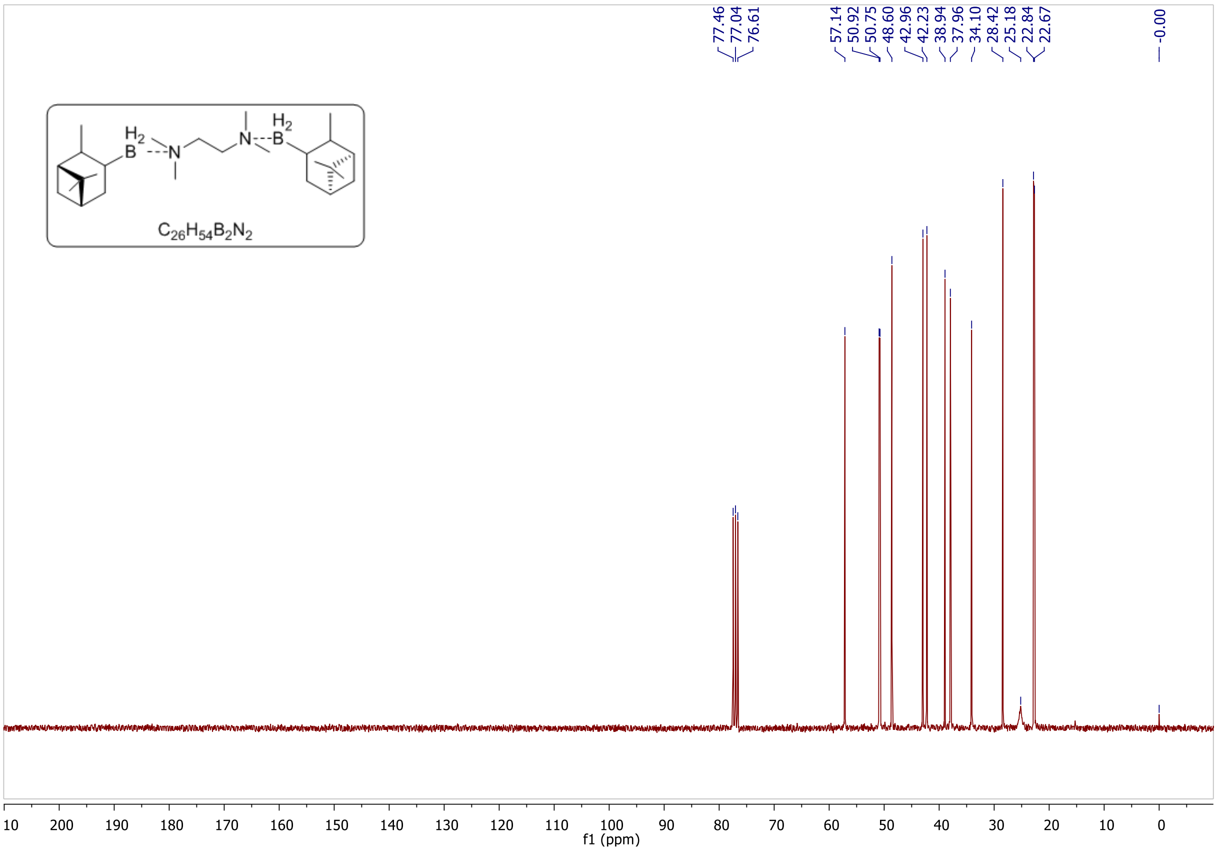

Supplementary Figure 17. ^{13}C-NMR (75 MHz) of (+)-(IpcBH$_2$)$_2$·TMEDA.

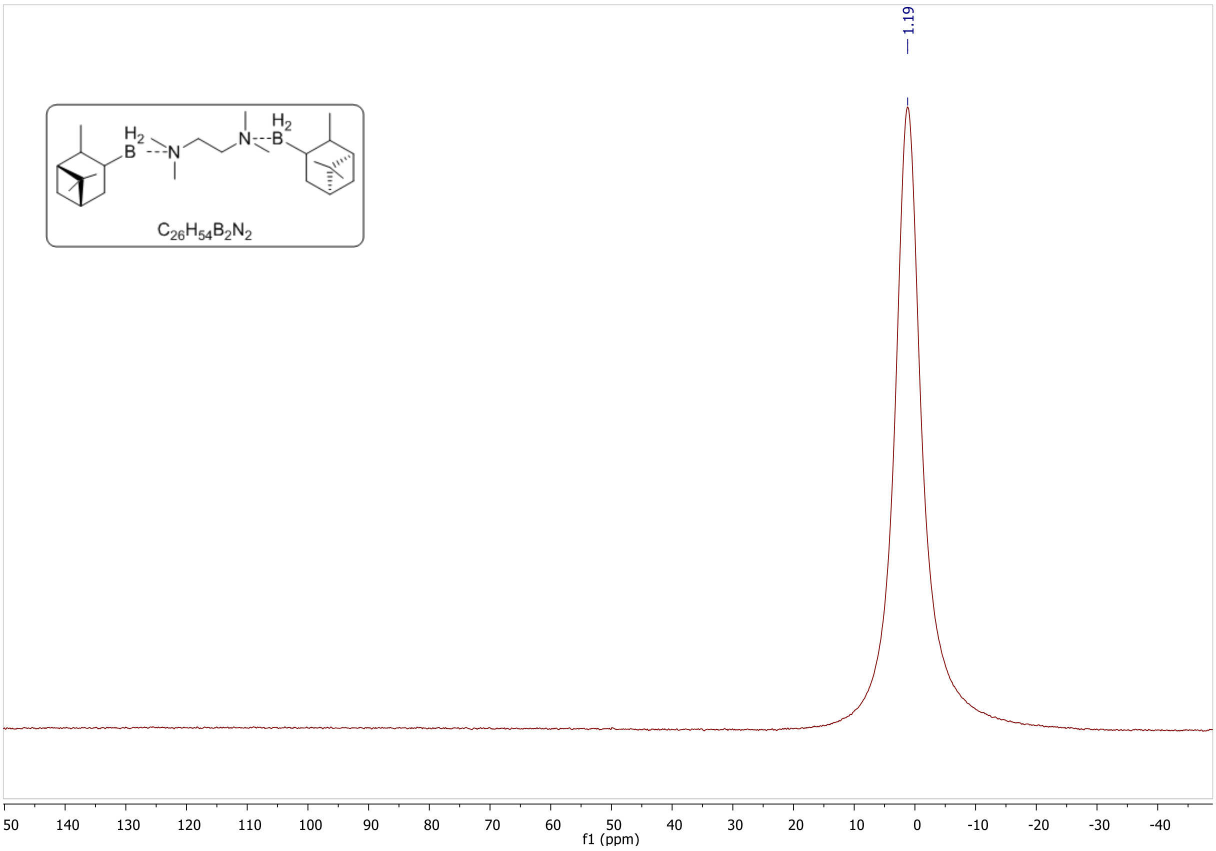

Supplementary Figure 18. ^{11}B-NMR (96 MHz) of (+)-$(IpcBH_2)_2$·TMEDA.

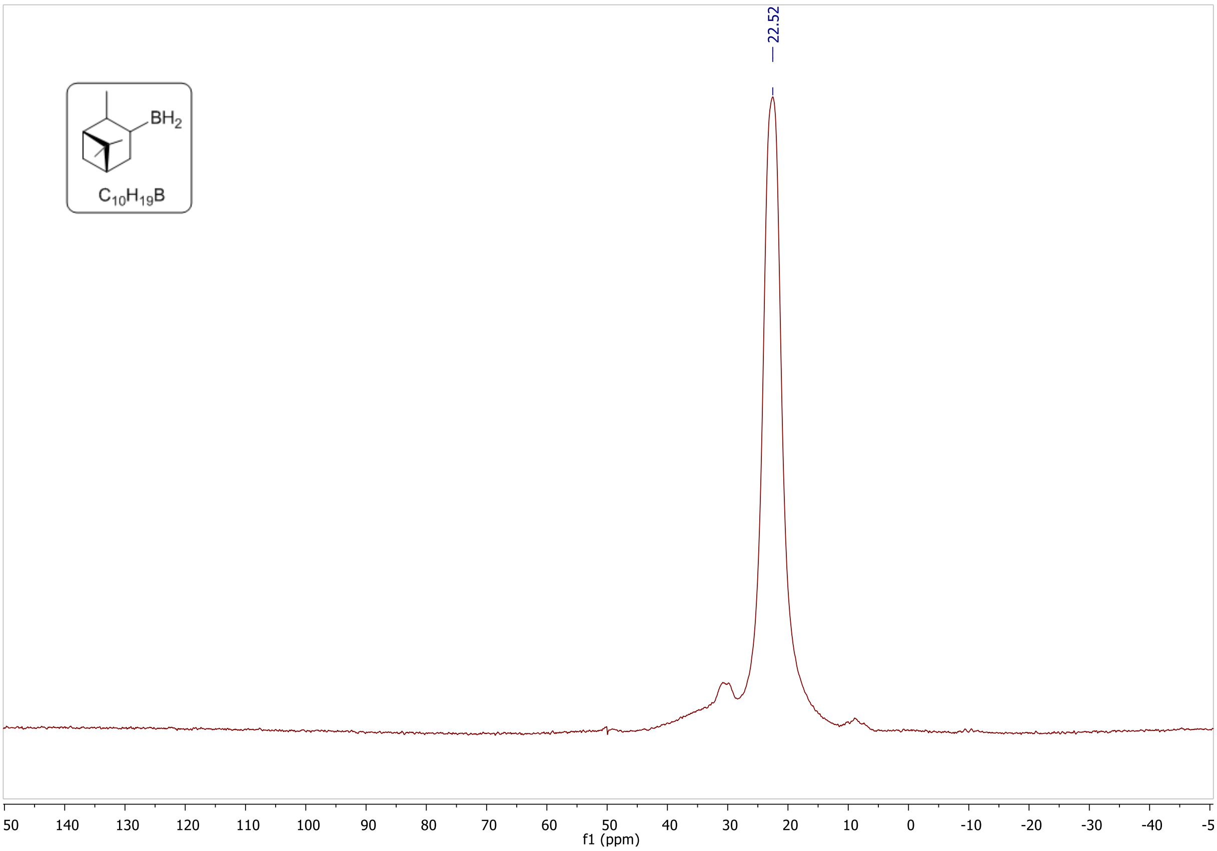

Supplementary Figure 19. ^{11}B-NMR (96 MHz) of (+)-$IpcBH_2$.

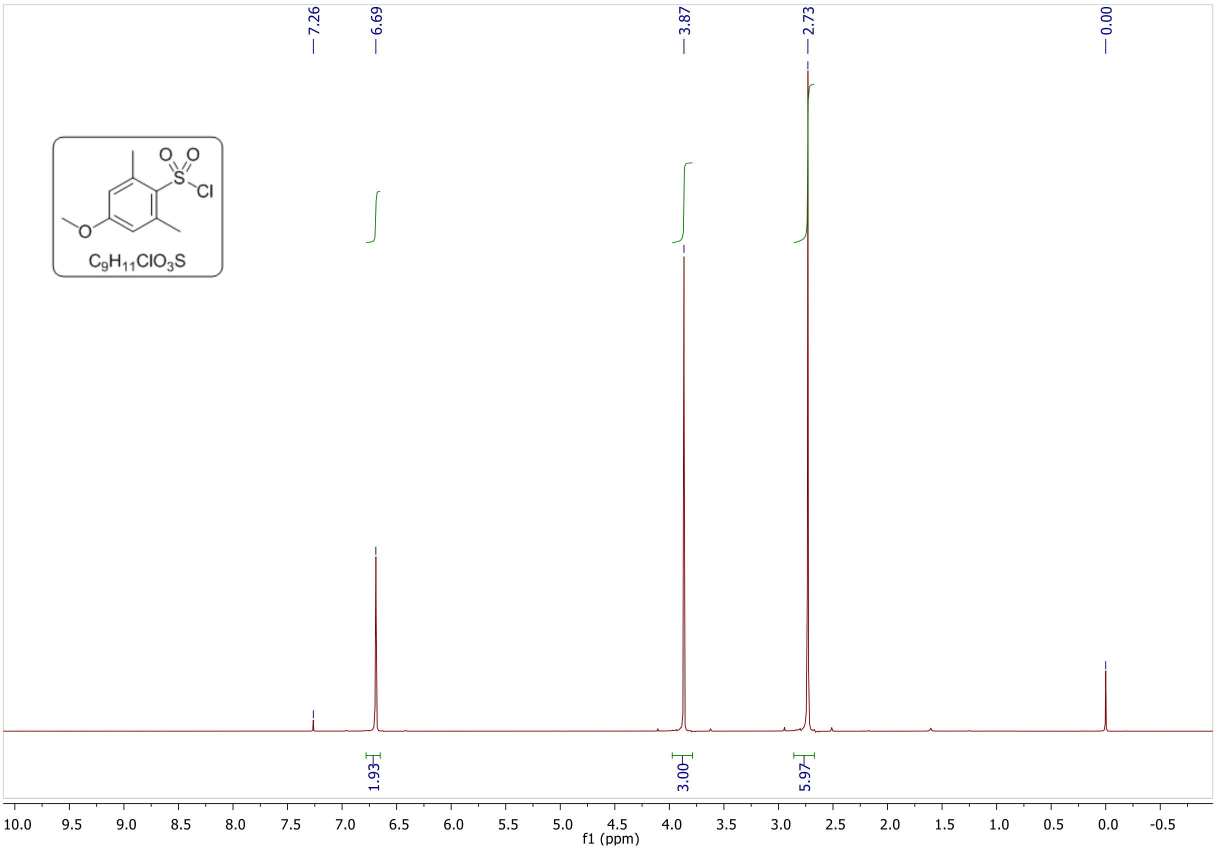

Supplementary Figure 20. ^{1}H-NMR (300 MHz) of 4-methoxy-2,6-dimethylbenzenesulfonyl chloride.

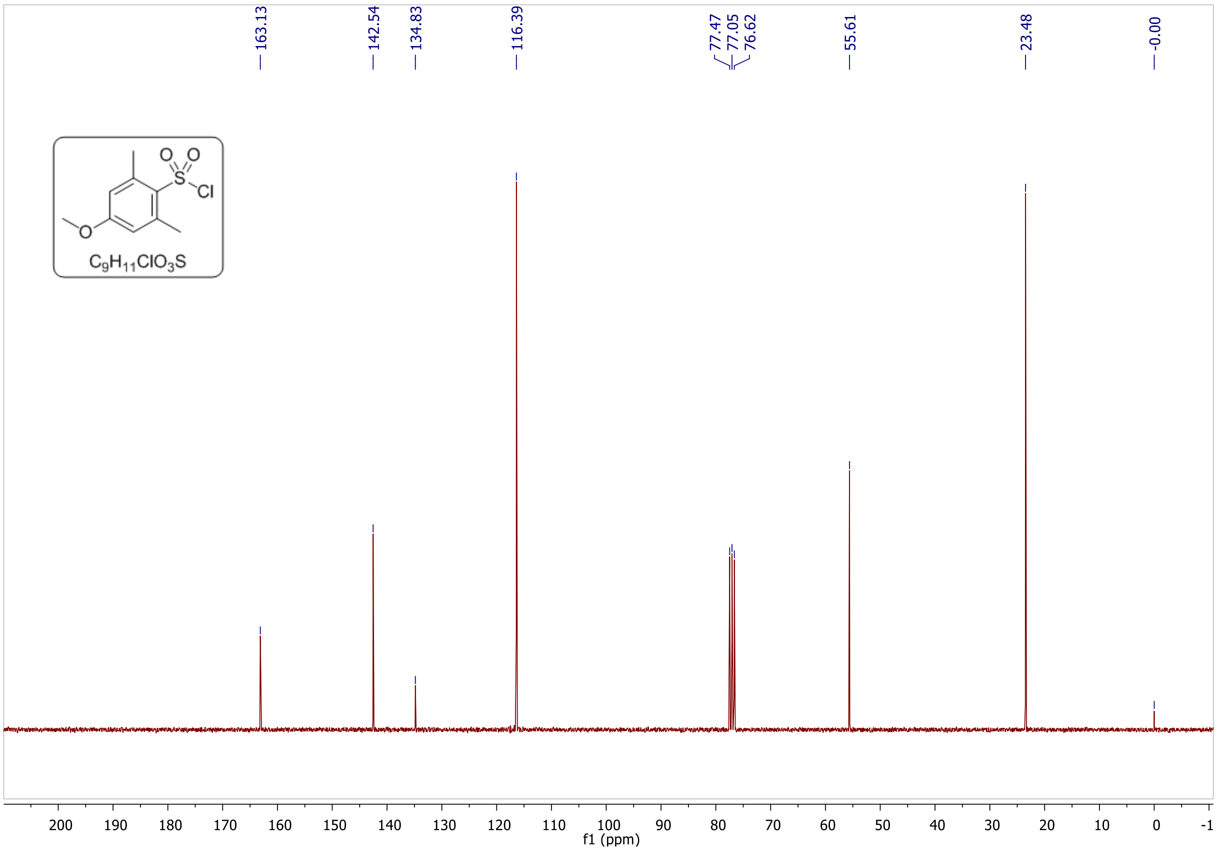

Supplementary Figure 21. ^{13}C-NMR (75 MHz) of 4-methoxy-2,6-dimethylbenzenesulfonyl chloride.

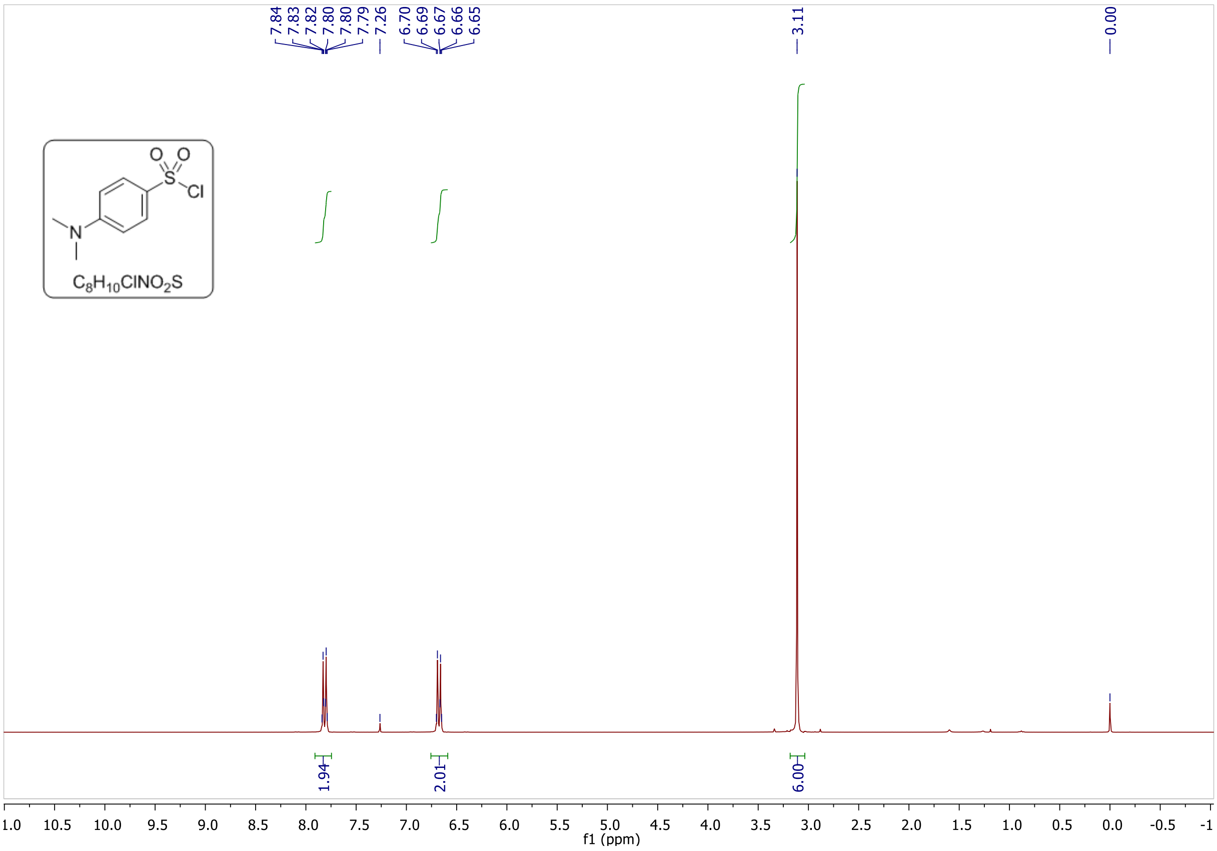

Supplementary Figure 22. ^{1}H-NMR (300 MHz) of 4-(dimethylamino)benzenesulfonyl chloride.

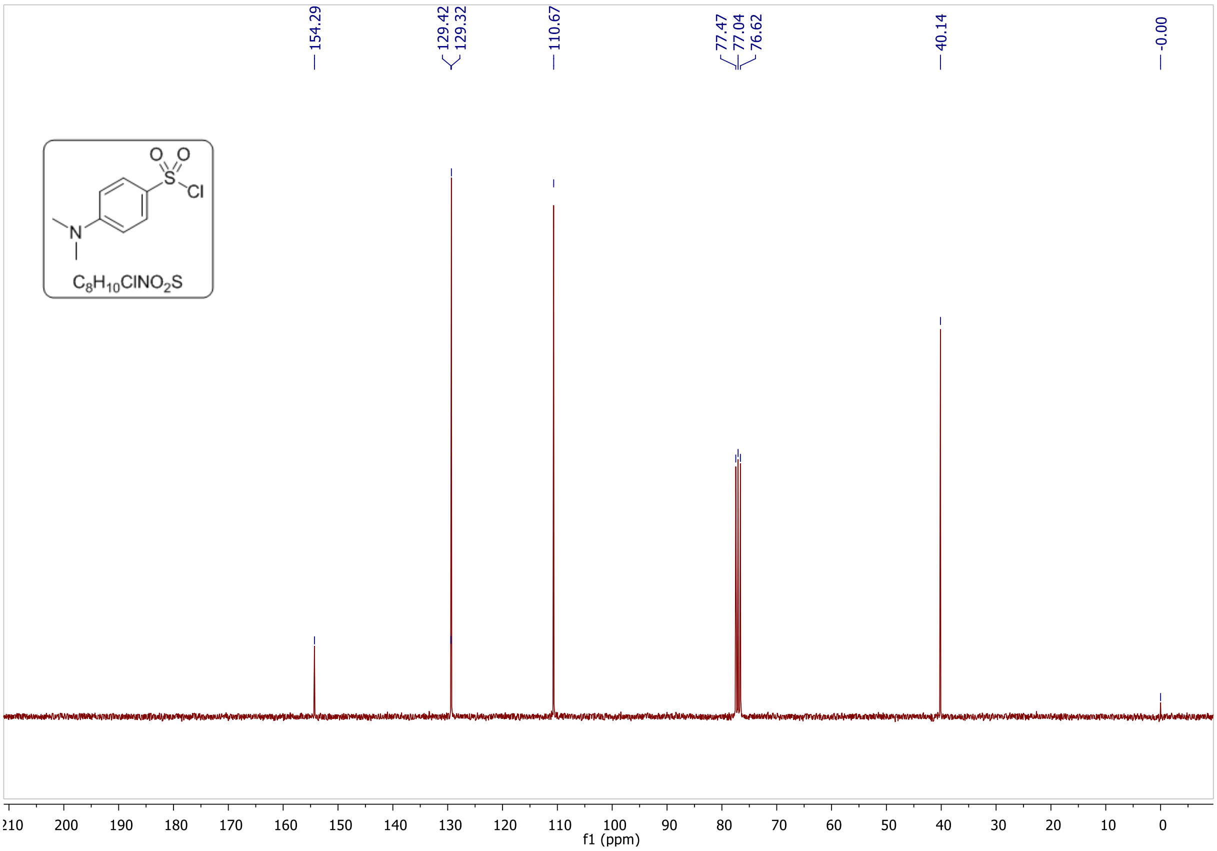

Supplementary Figure 23. ^{13}C-NMR (75 MHz) of 4-(dimethylamino)benzenesulfonyl chloride.

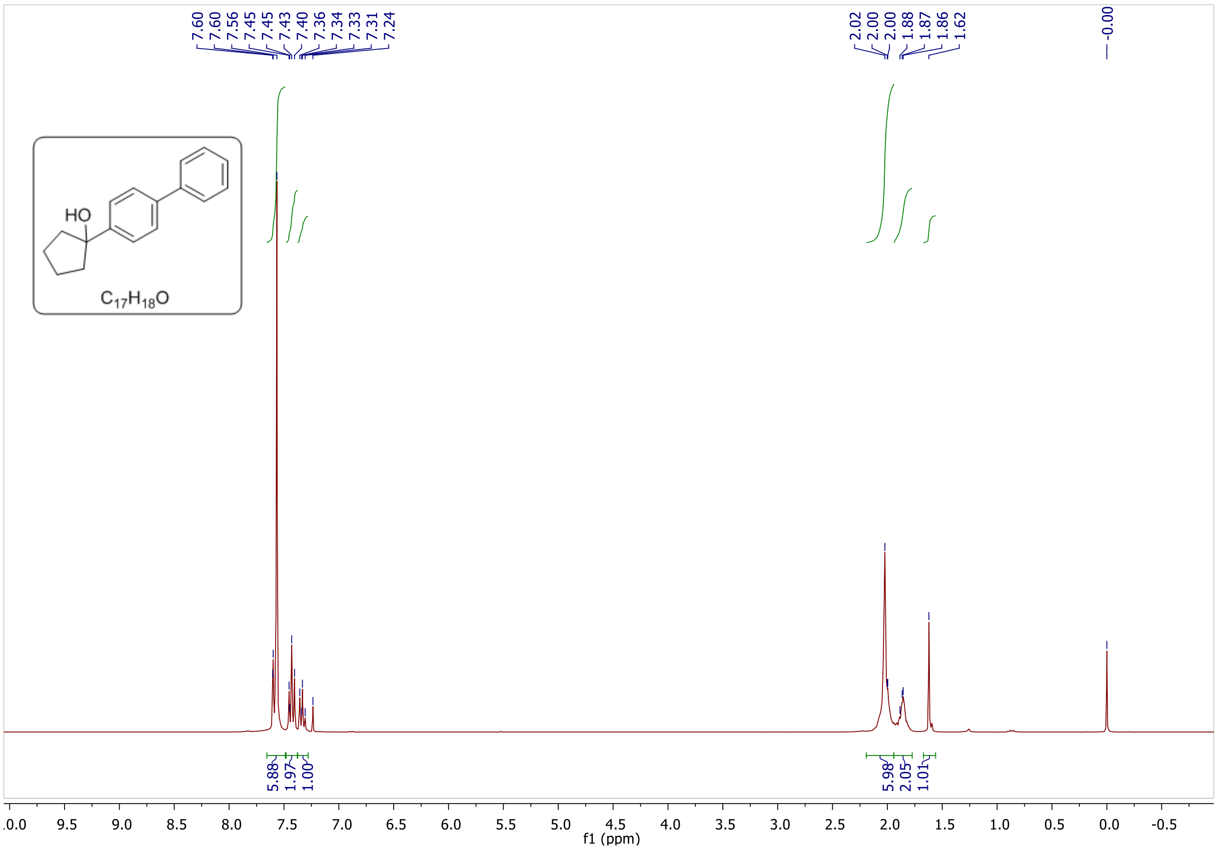

Supplementary Figure 24. ^{1}H-NMR (300 MHz) of 1-([1,1'-biphenyl]-4-yl)cyclopentan-1-ol.

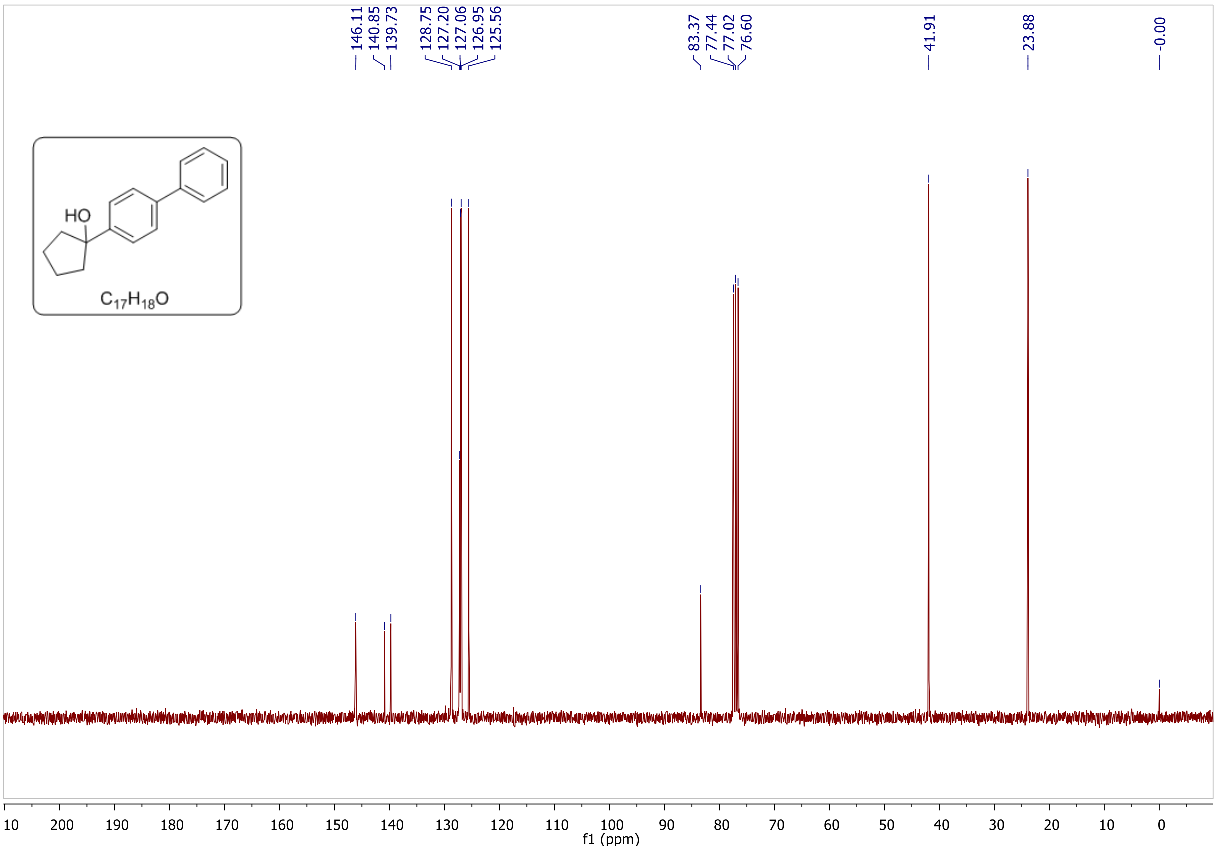

Supplementary Figure 25. ^{13}C-NMR (75 MHz) of 1-([1,1'-biphenyl]-4-yl)cyclopentan-1-ol.

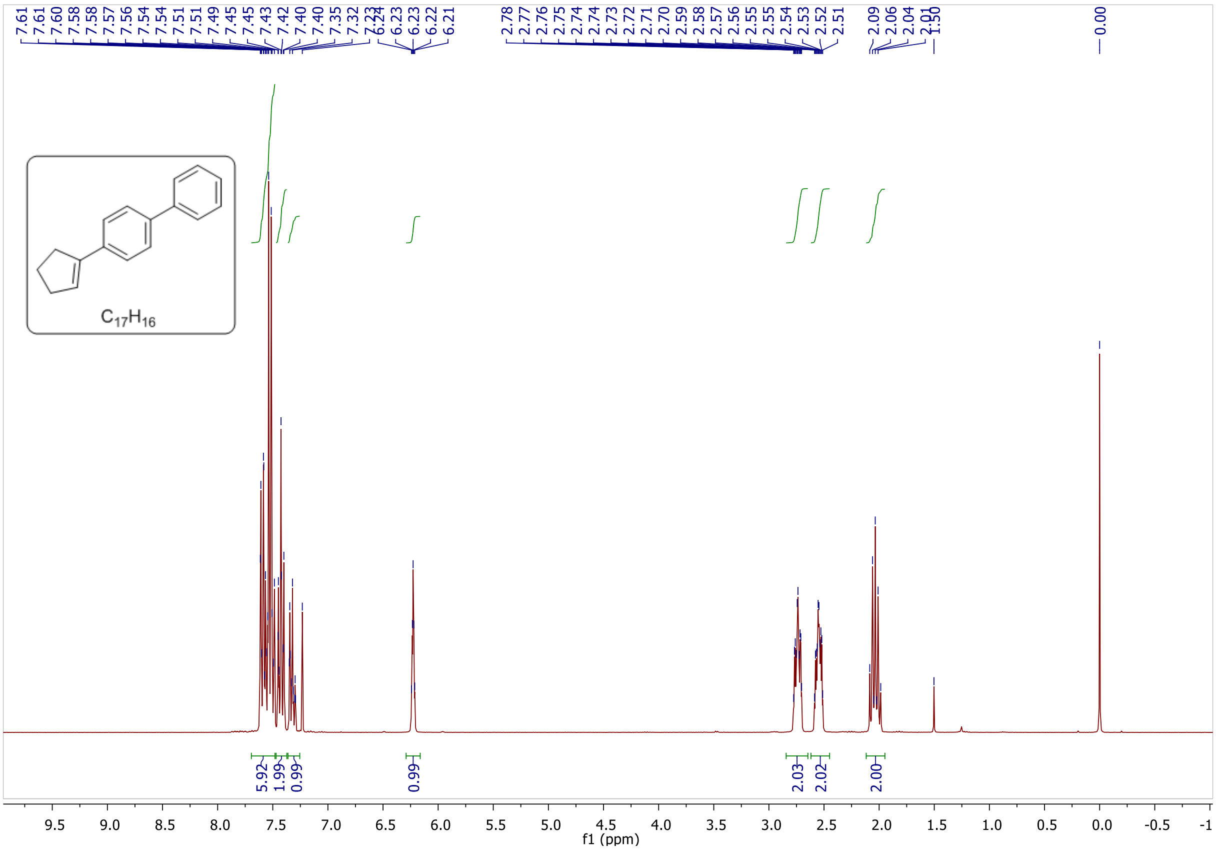

Supplementary Figure 26. ^{1}H-NMR (300 MHz) of **2b**.

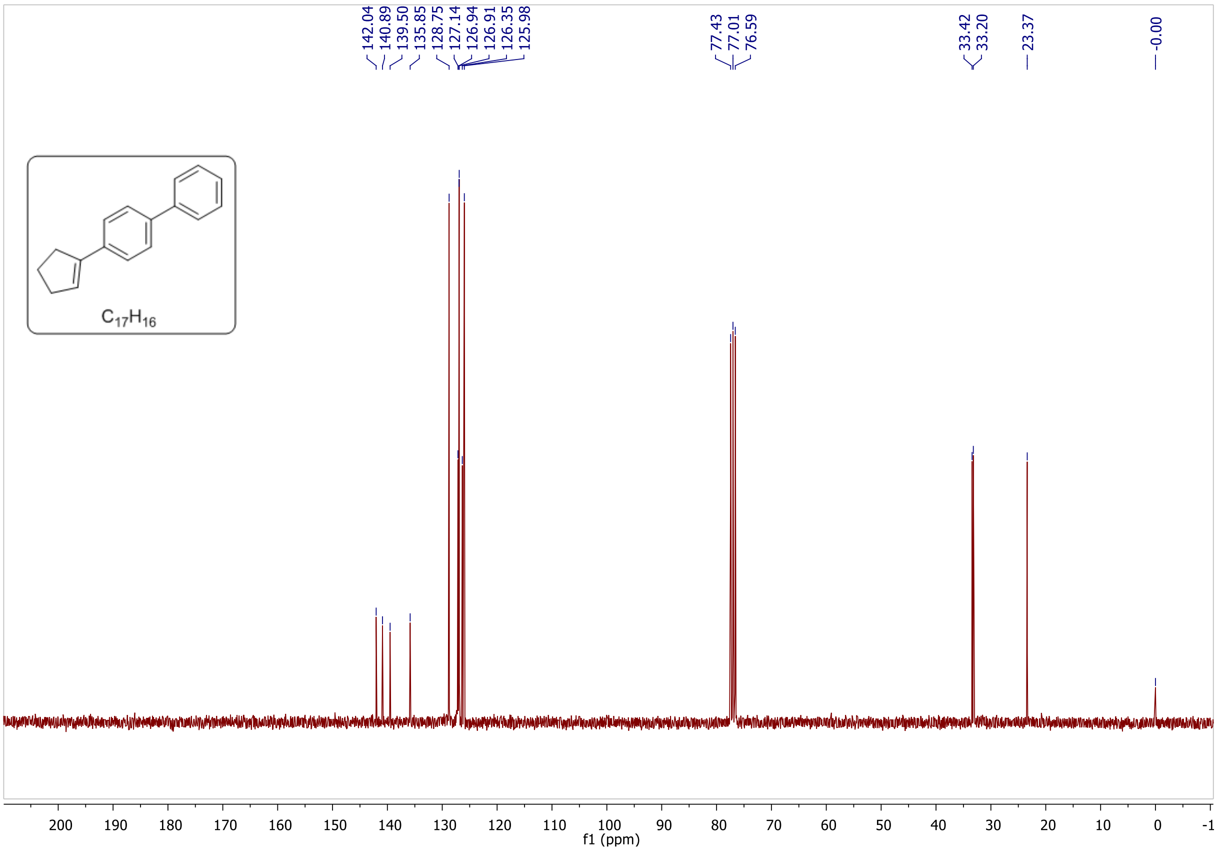

Supplementary Figure 27. ^{13}C-NMR (75 MHz) of **2b**.

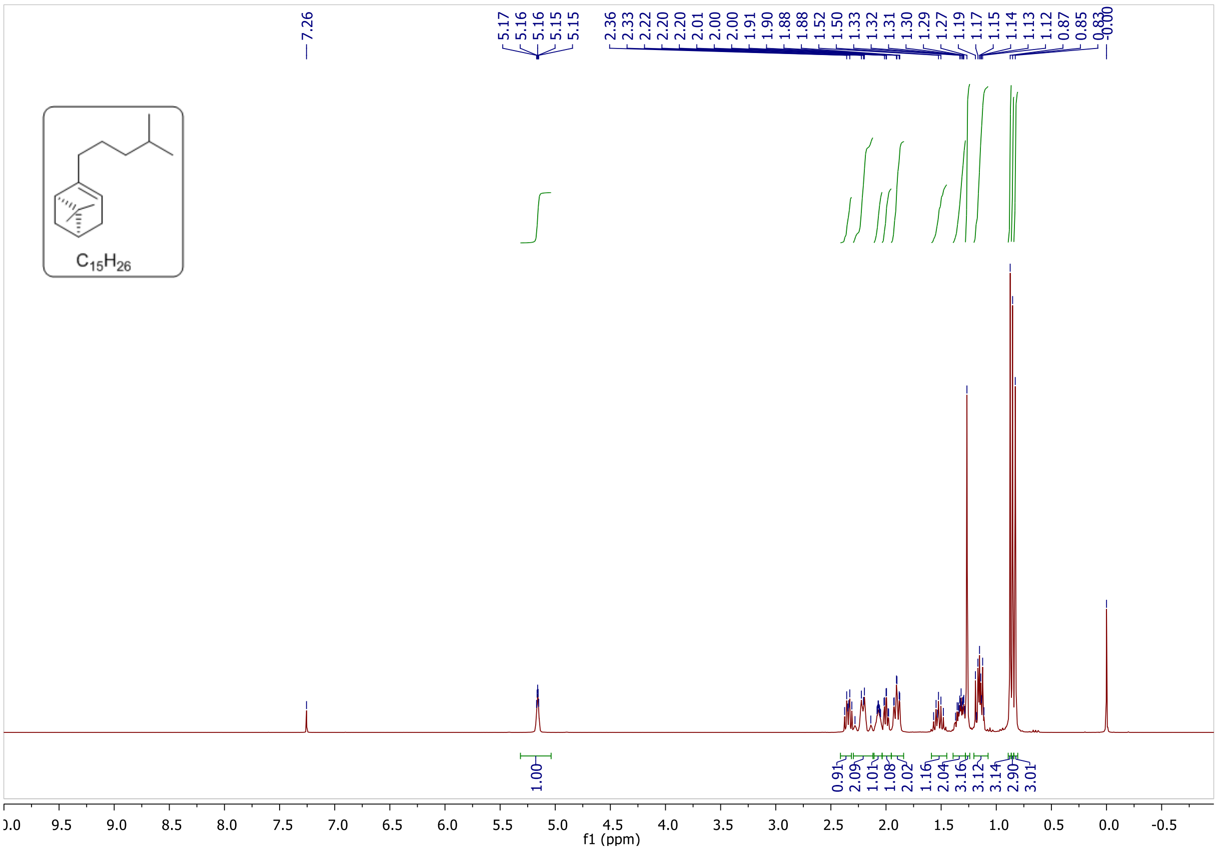

Supplementary Figure 28. ^{1}H-NMR (300 MHz) of **2c**.

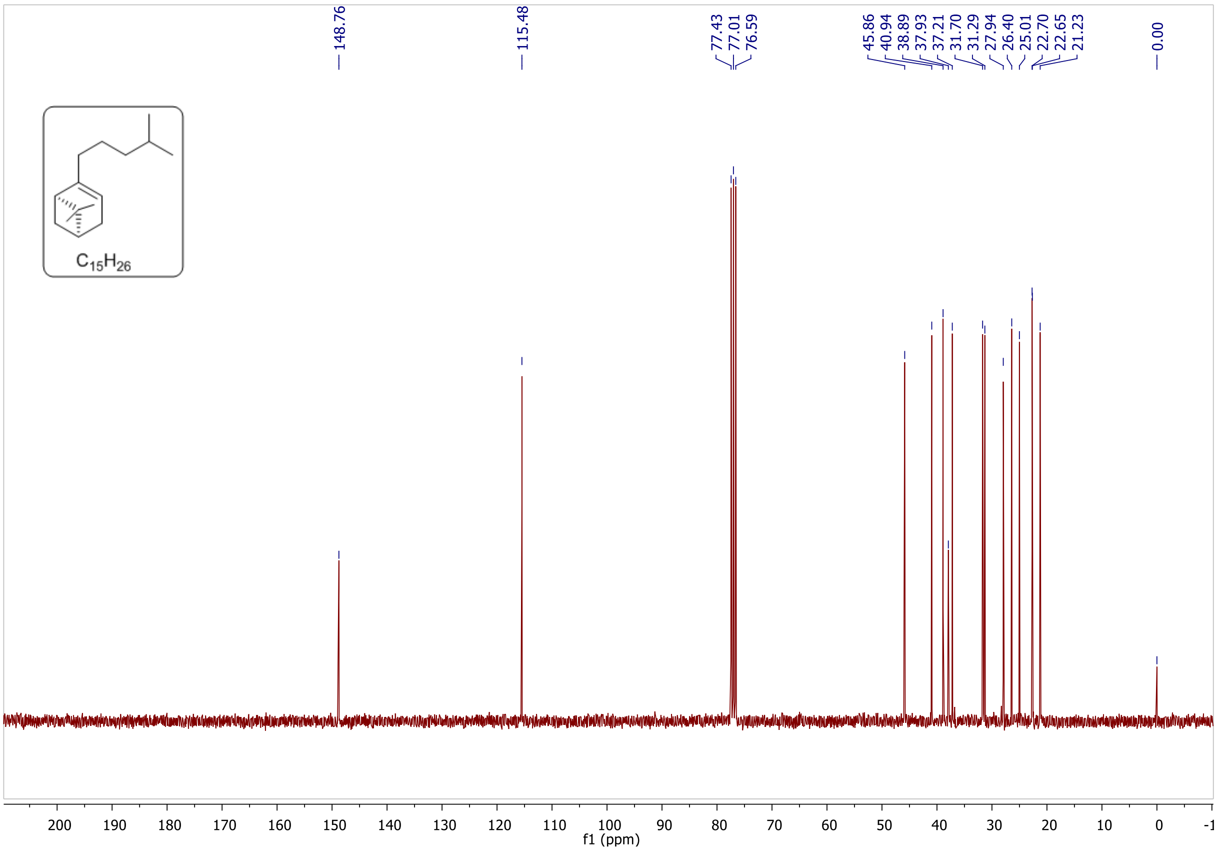

Supplementary Figure 29. ^{13}C-NMR (75 MHz) of **2c**.

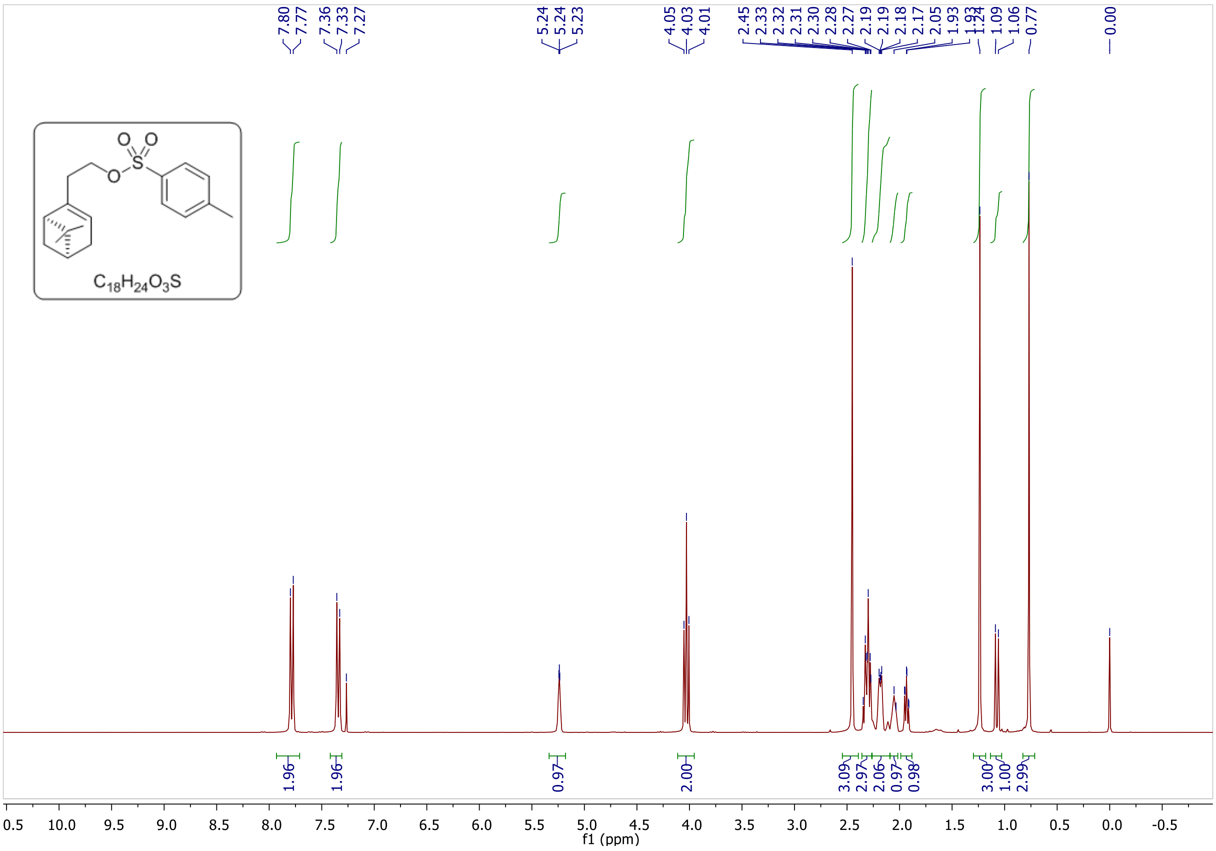

Supplementary Figure 30. ^{1}H-NMR (300 MHz) of **2d**.

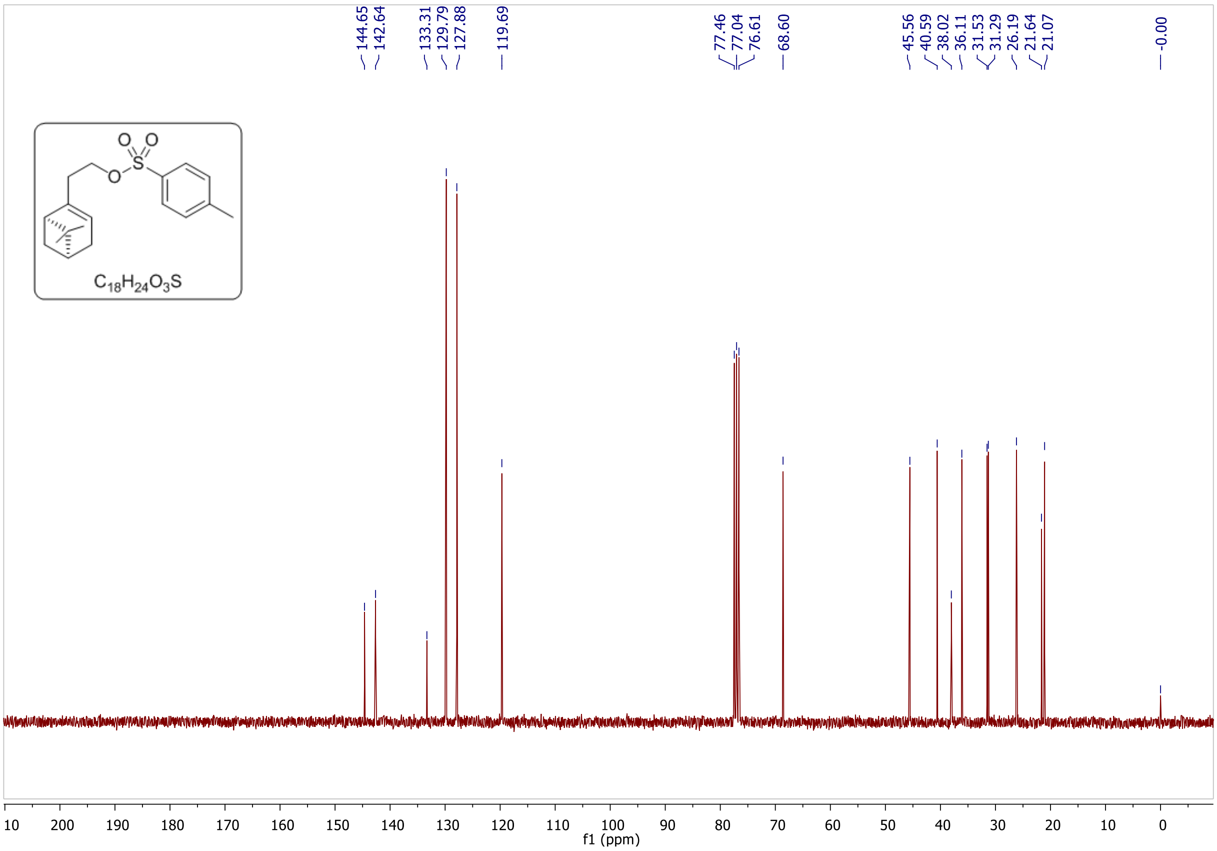

Supplementary Figure 31. ^{13}C-NMR (75 MHz) of **2d**.

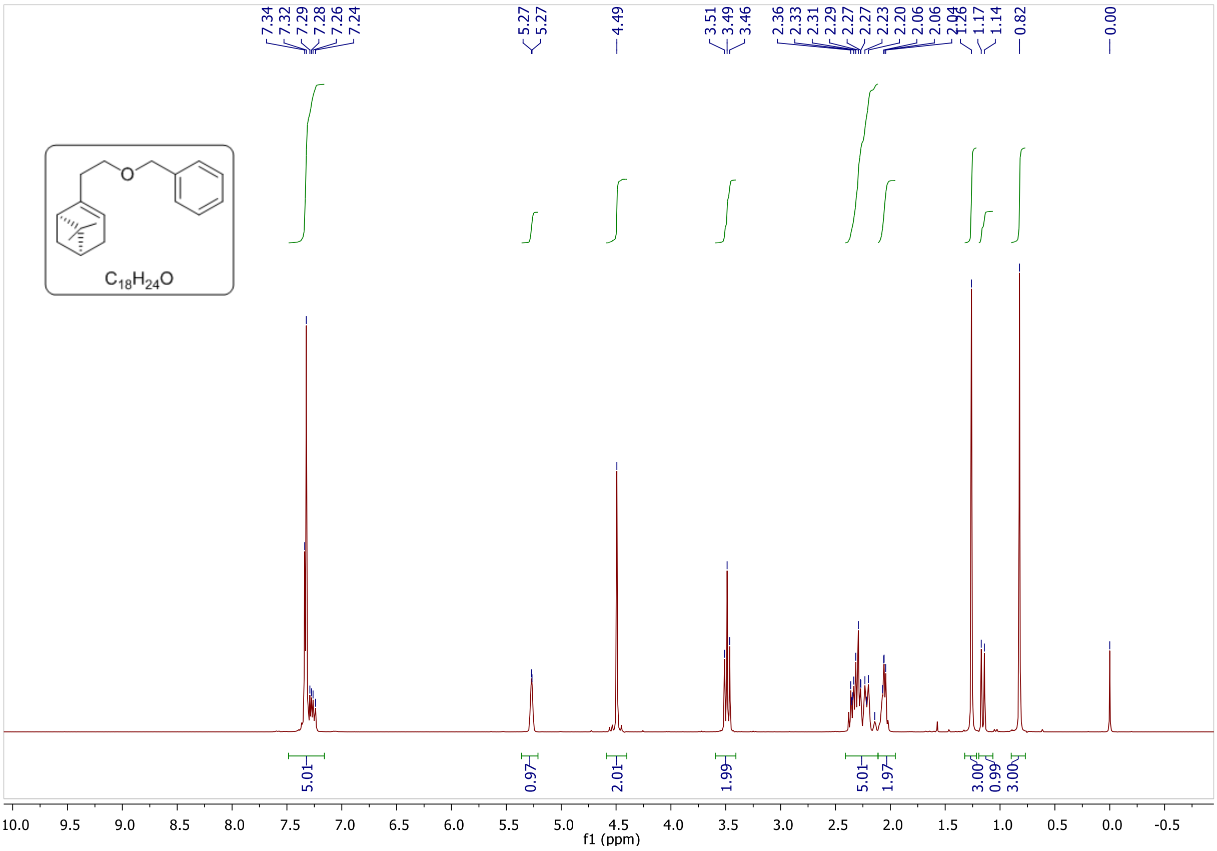

Supplementary Figure 32. ^{1}H-NMR (300 MHz) of **2e**.

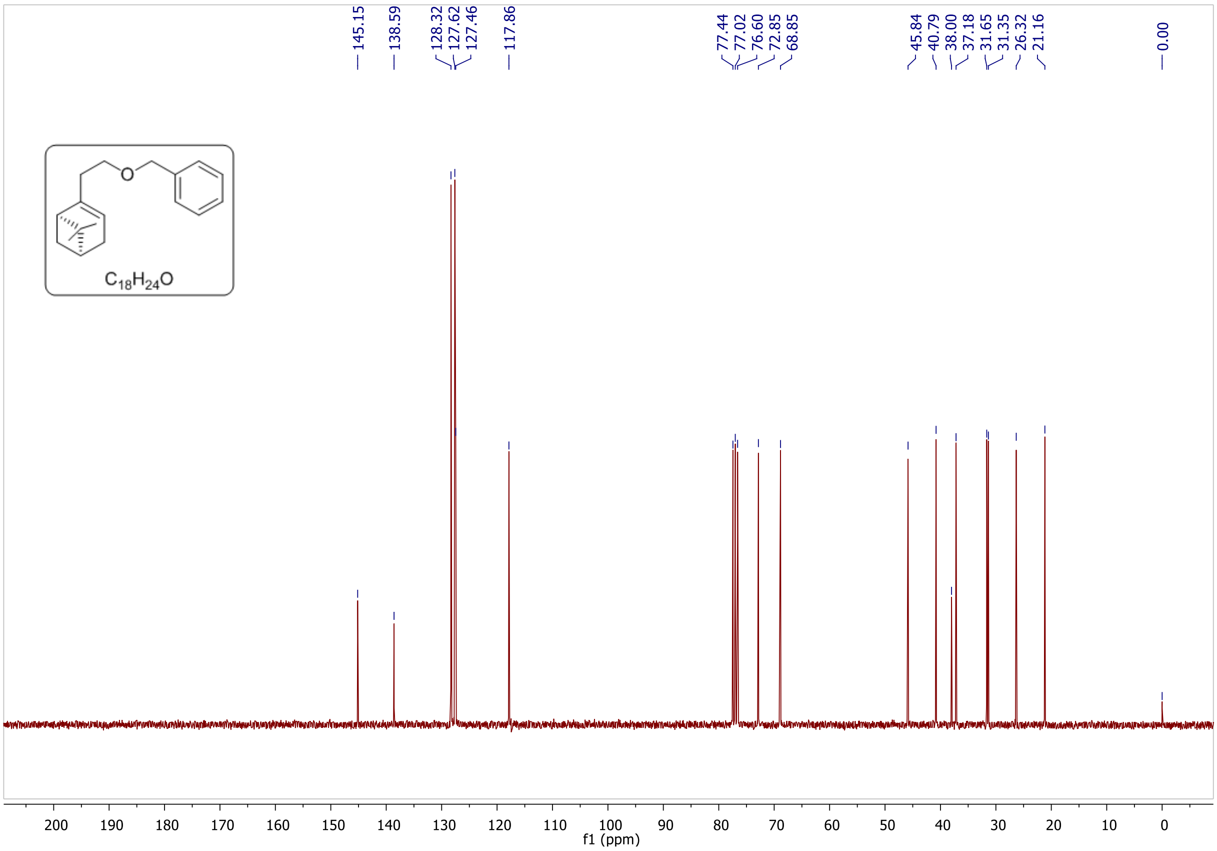

Supplementary Figure 33. ^{13}C-NMR (75 MHz) of **2e**.

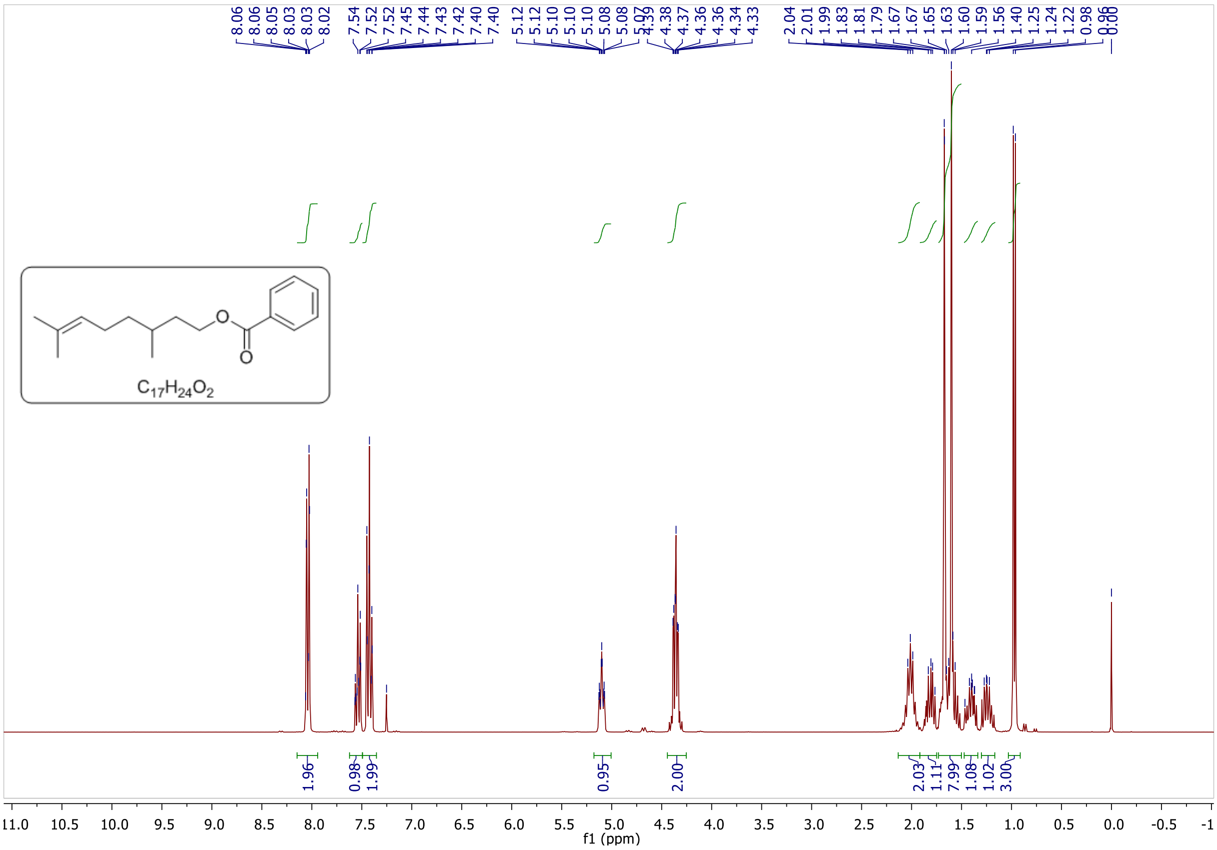

Supplementary Figure 34. ^{1}H-NMR (300 MHz) of **2f**.

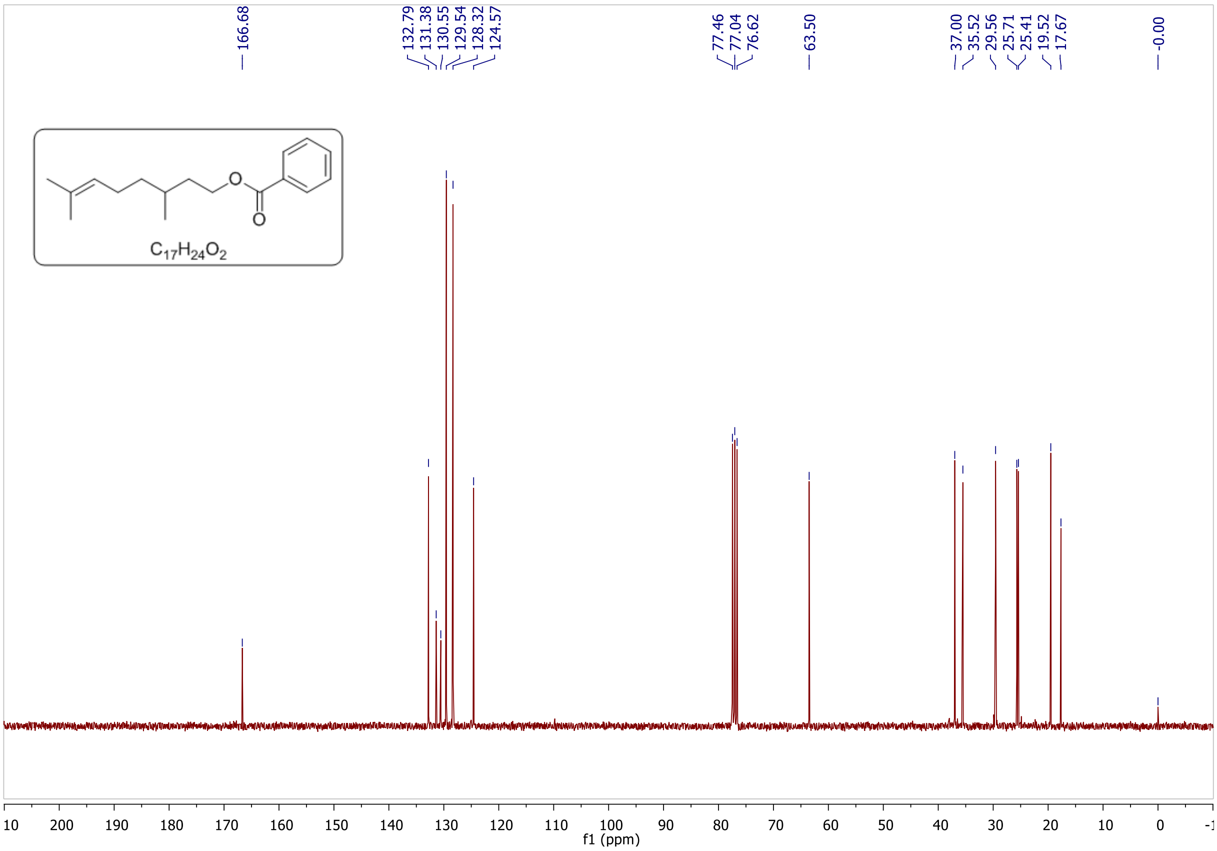

Supplementary Figure 35. ^{13}C-NMR (75 MHz) of **2f**.

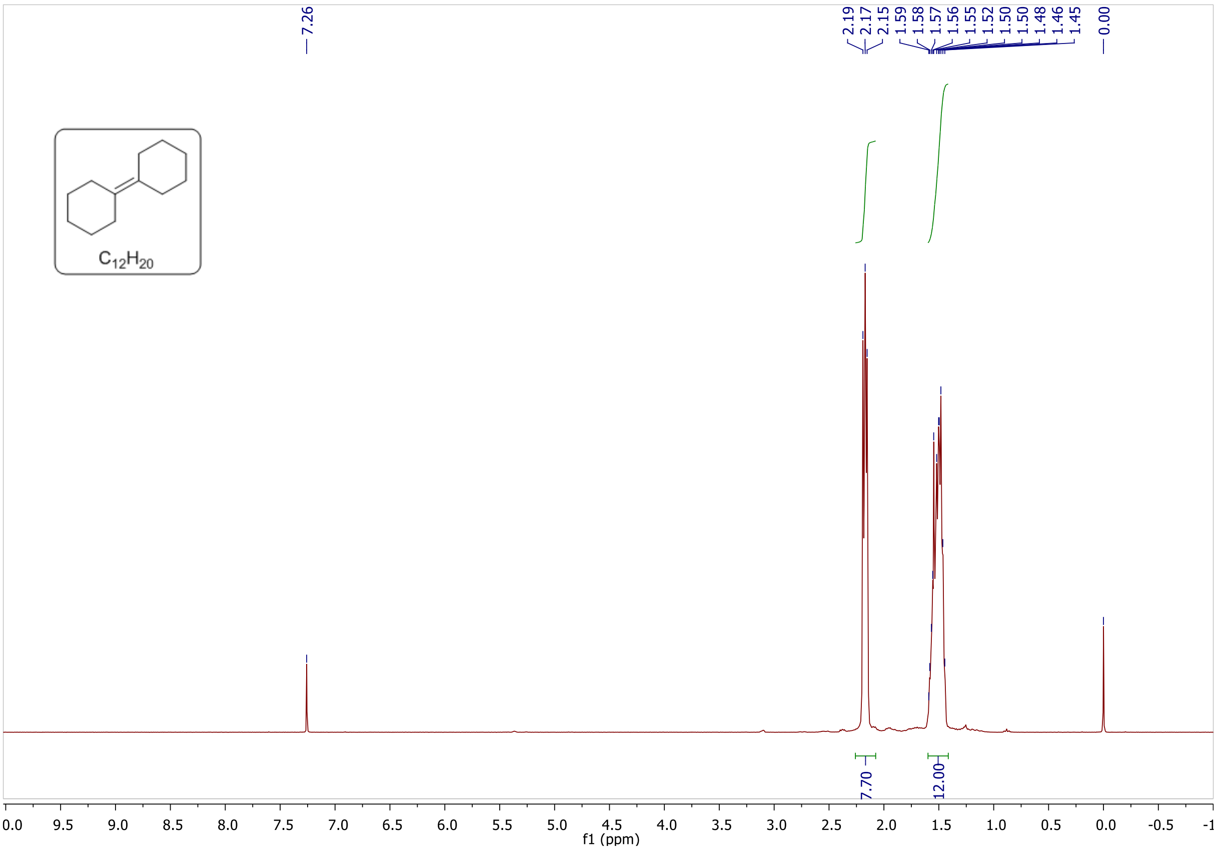

Supplementary Figure 36. ^{1}H-NMR (300 MHz) of **2h**.

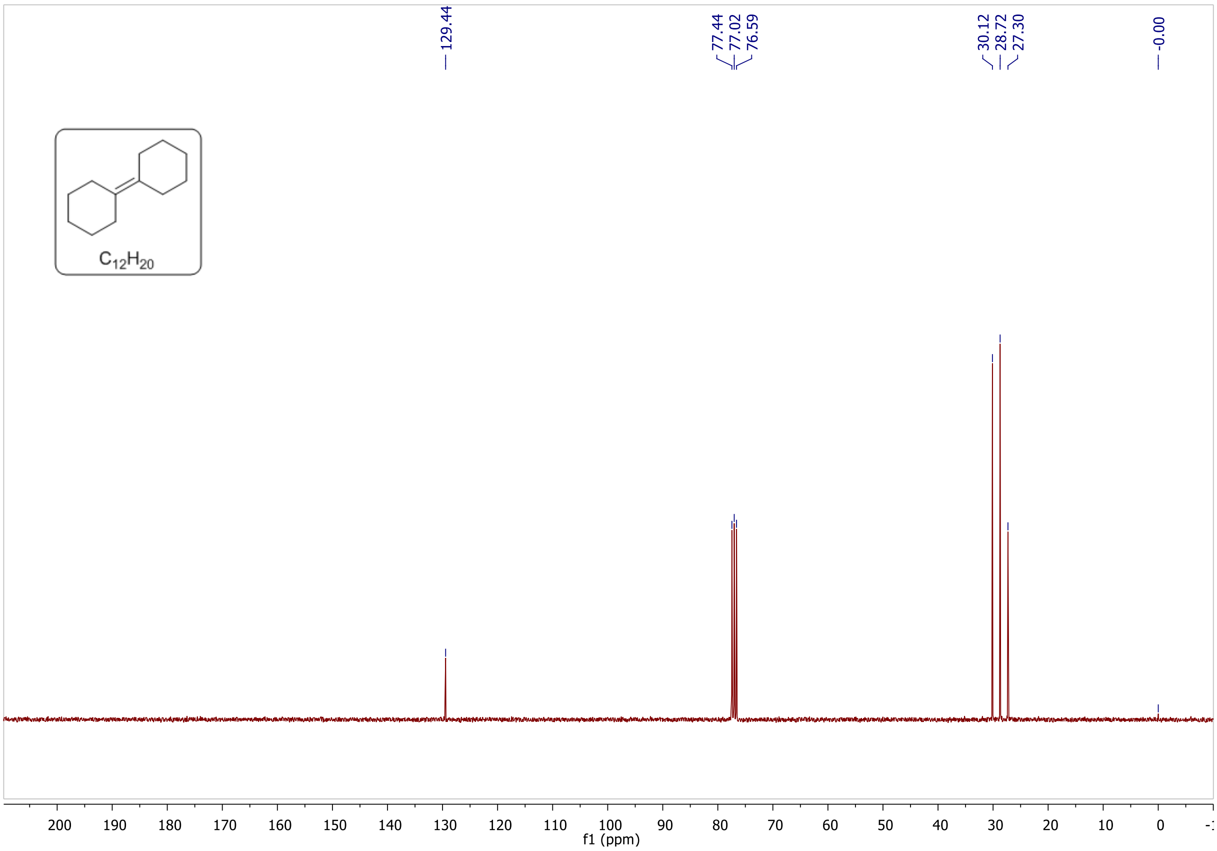

Supplementary Figure 37. ^{13}C-NMR (75 MHz) of **2h**.

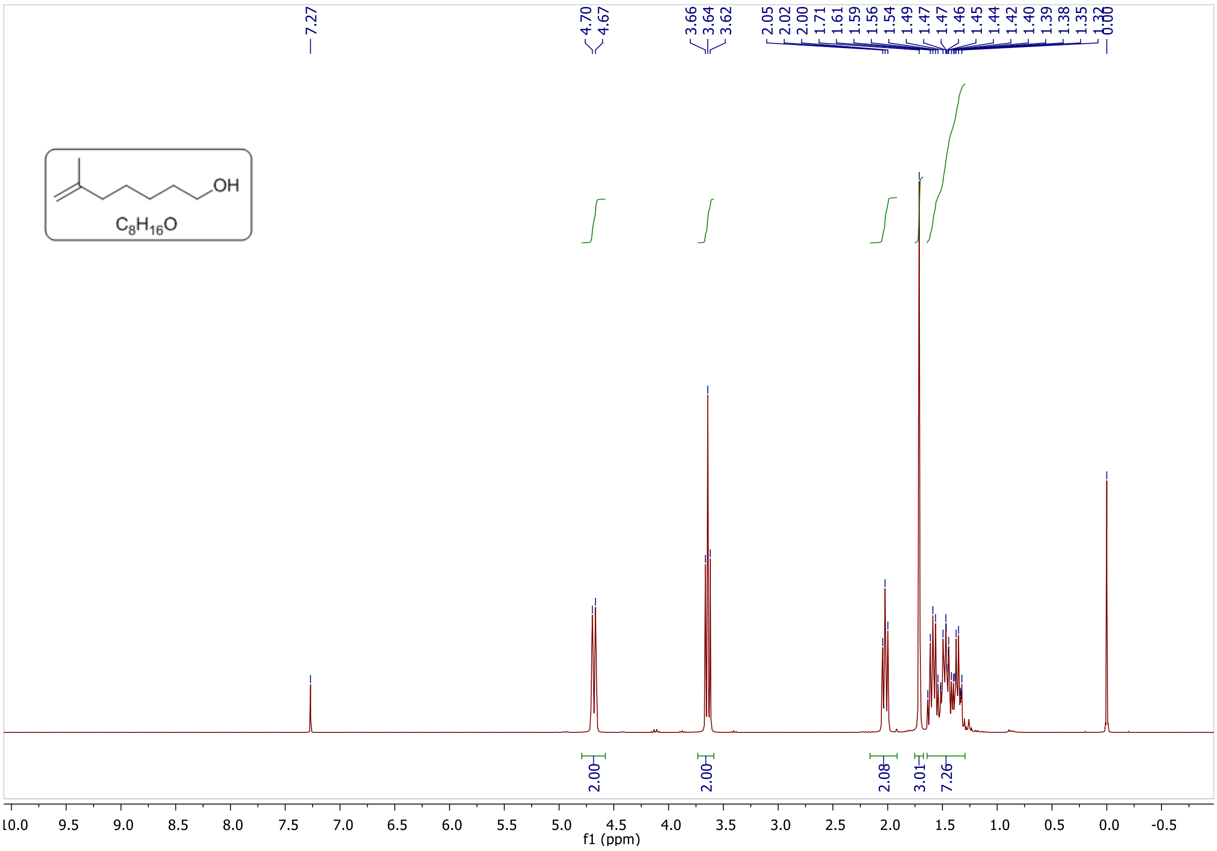

Supplementary Figure 38. ^{1}H-NMR (300 MHz) of 6-methylhept-6-en-1-ol.

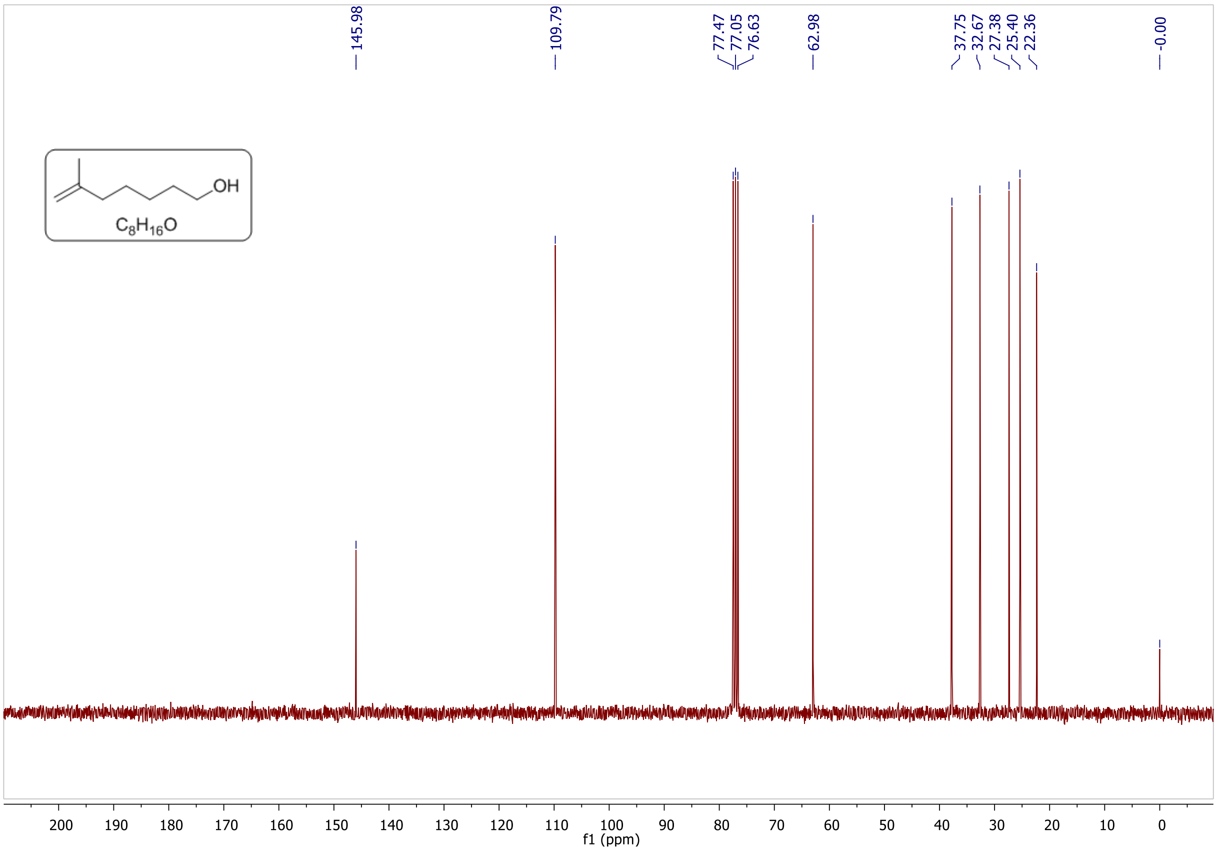

Supplementary Figure 39. ^{13}C-NMR (75 MHz) of 6-methylhept-6-en-1-ol.

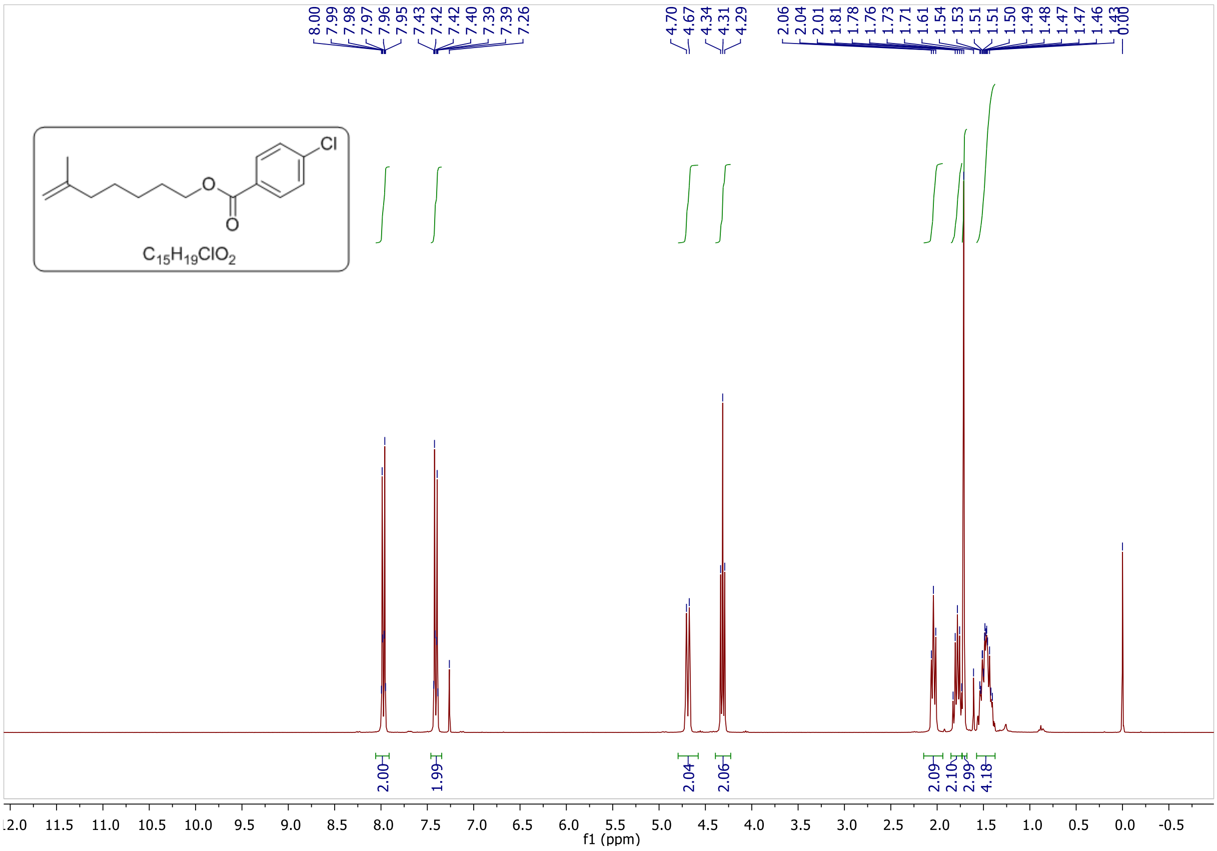

Supplementary Figure 40. ^{1}H-NMR (300 MHz) of **8a**.

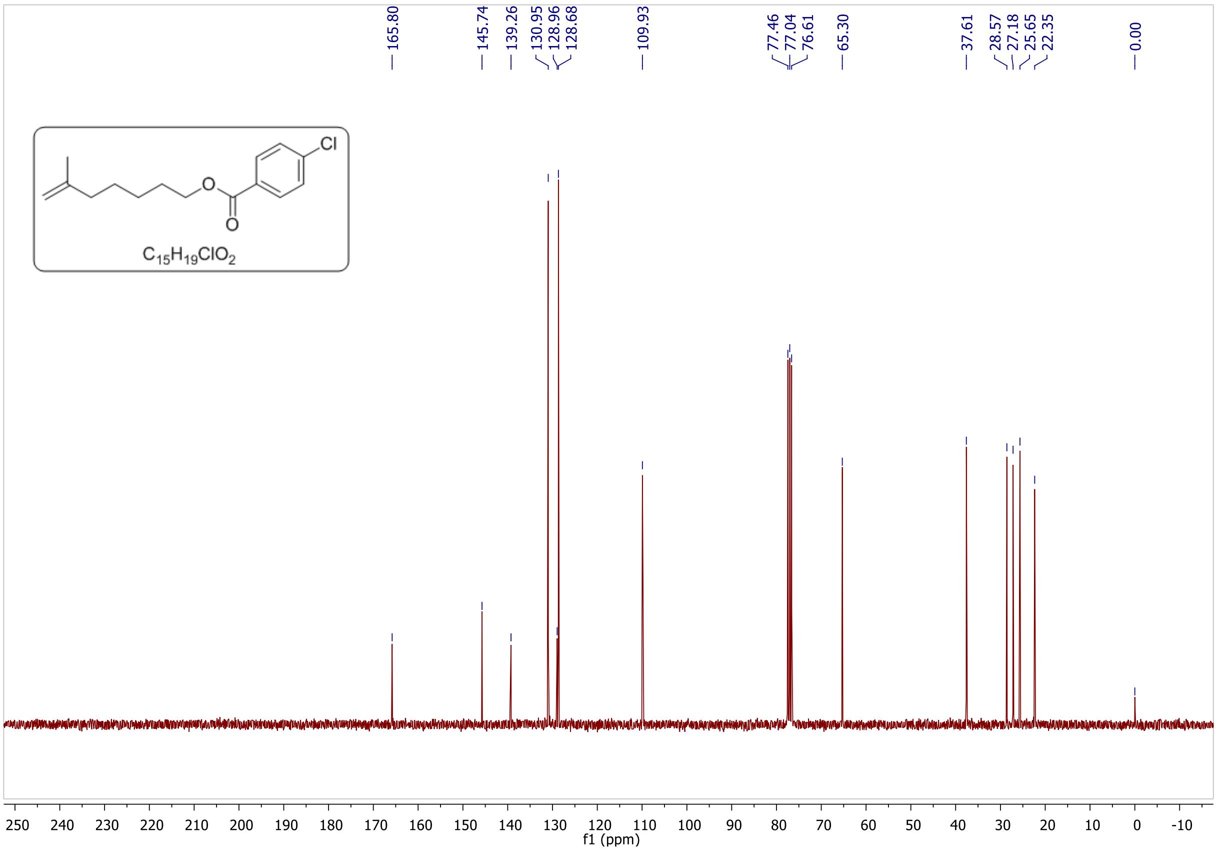

Supplementary Figure 41. ^{13}C-NMR (75 MHz) of **8a**.

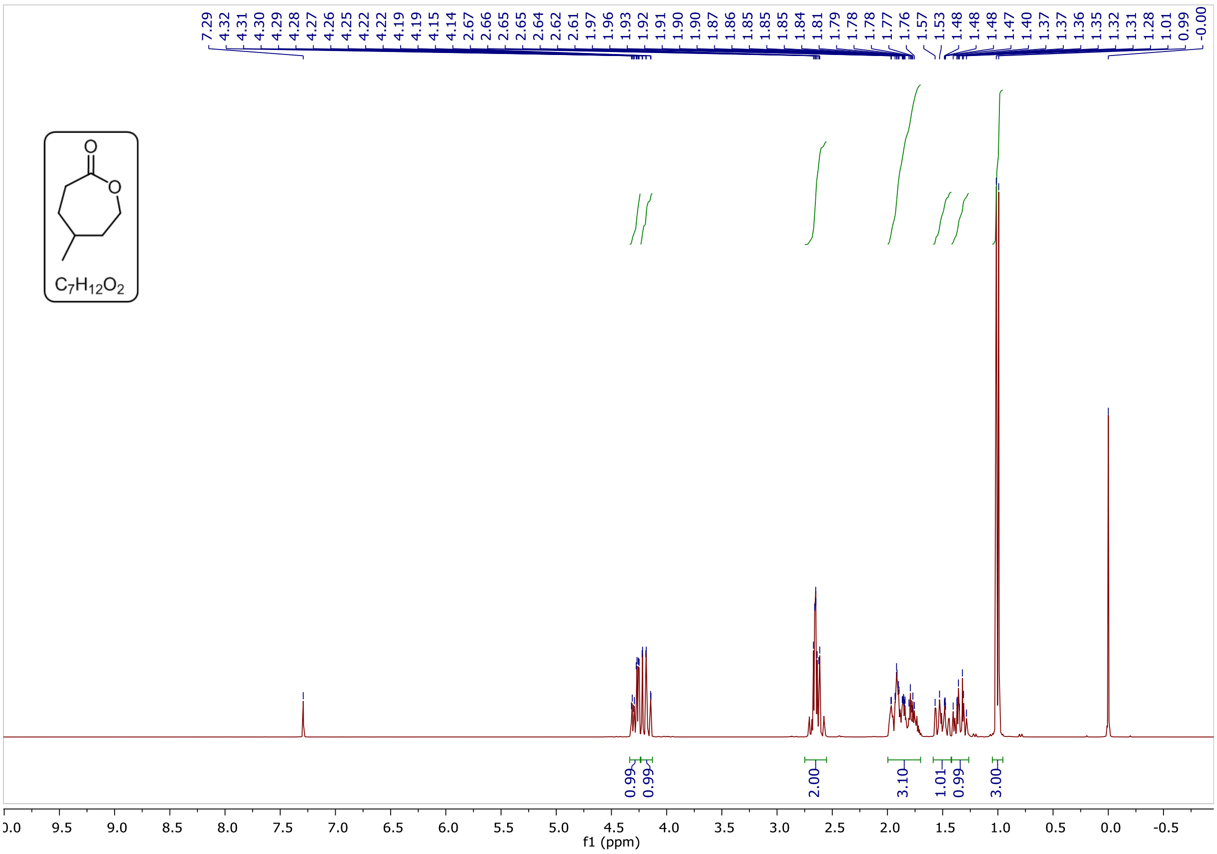

Supplementary Figure 42. ^{1}H-NMR (300 MHz) of 6-methylhept-6-en-1-ol.

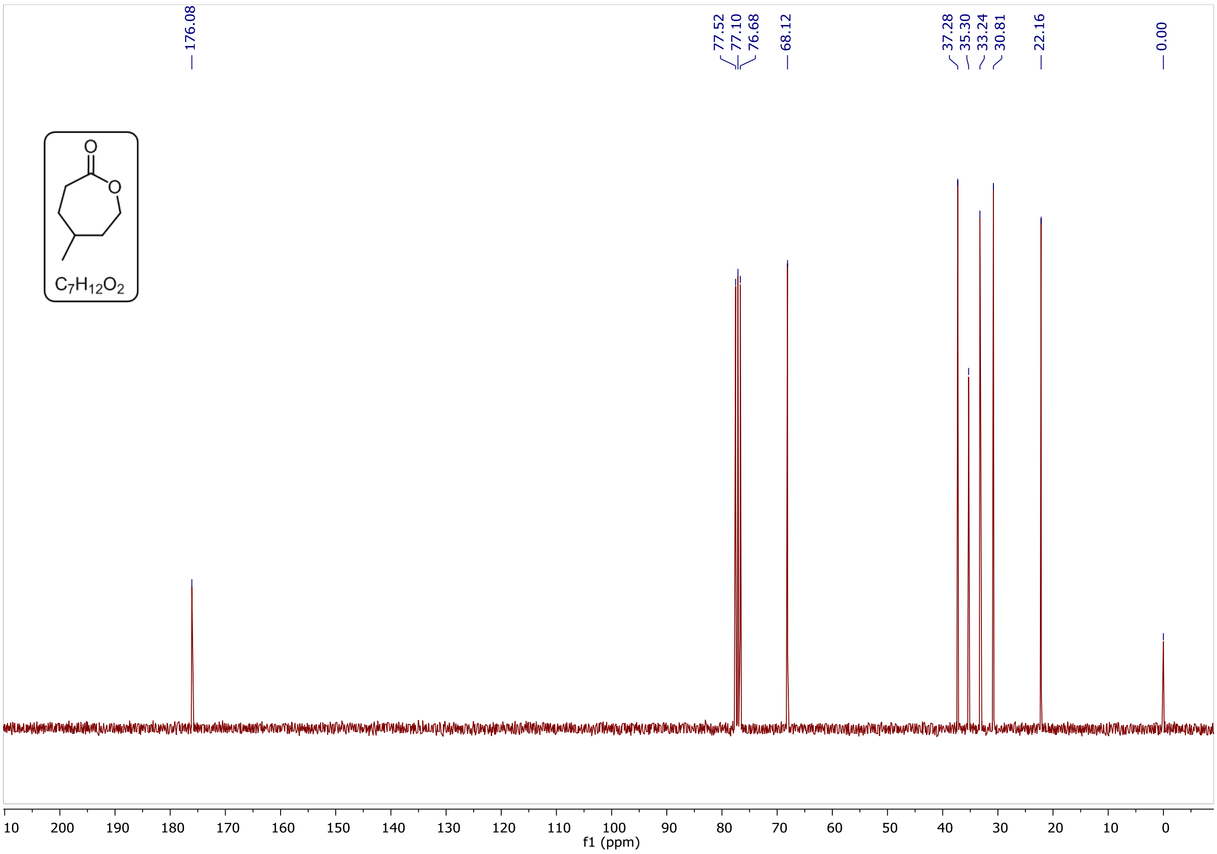

Supplementary Figure 43. ^{13}C-NMR (75 MHz) of 6-methylhept-6-en-1-ol.

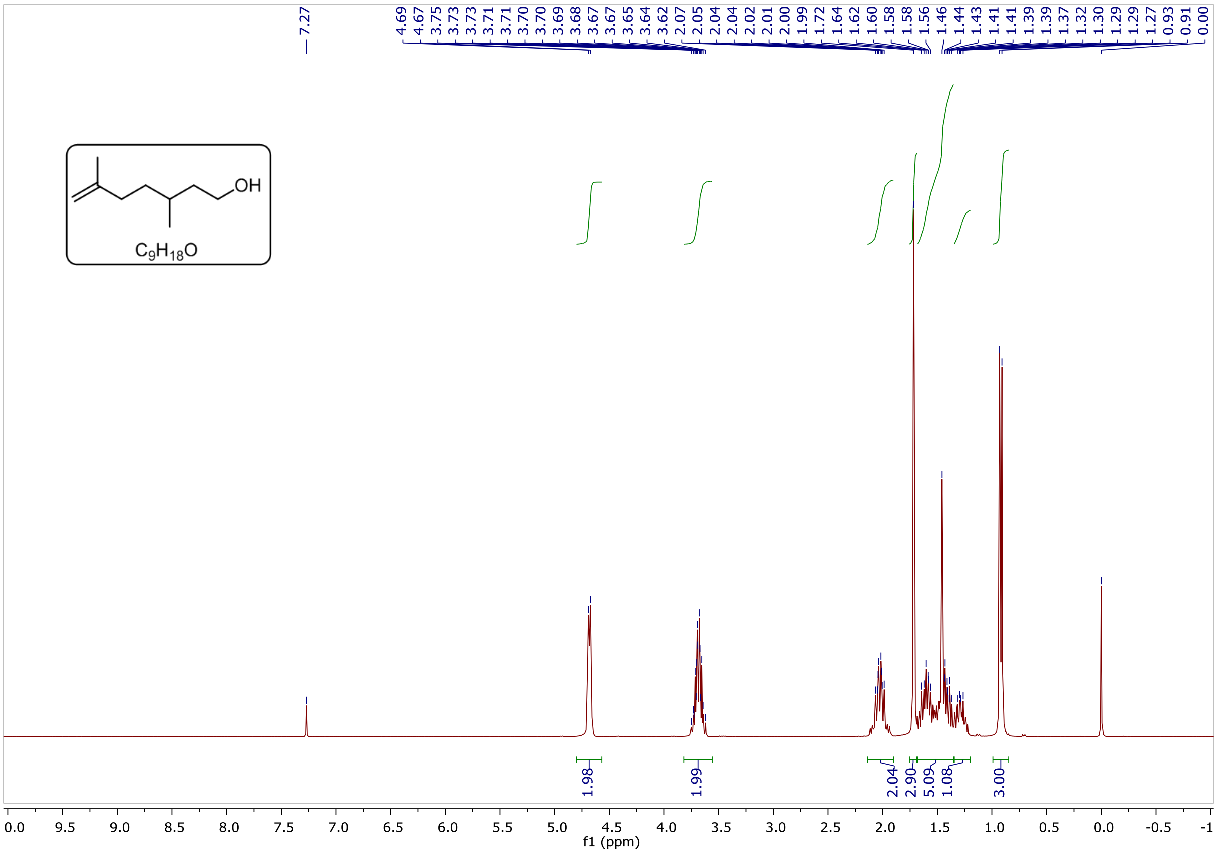

Supplementary Figure 44. ^{1}H-NMR (300 MHz) of 3,6-dimethylhept-6-en-1-ol.

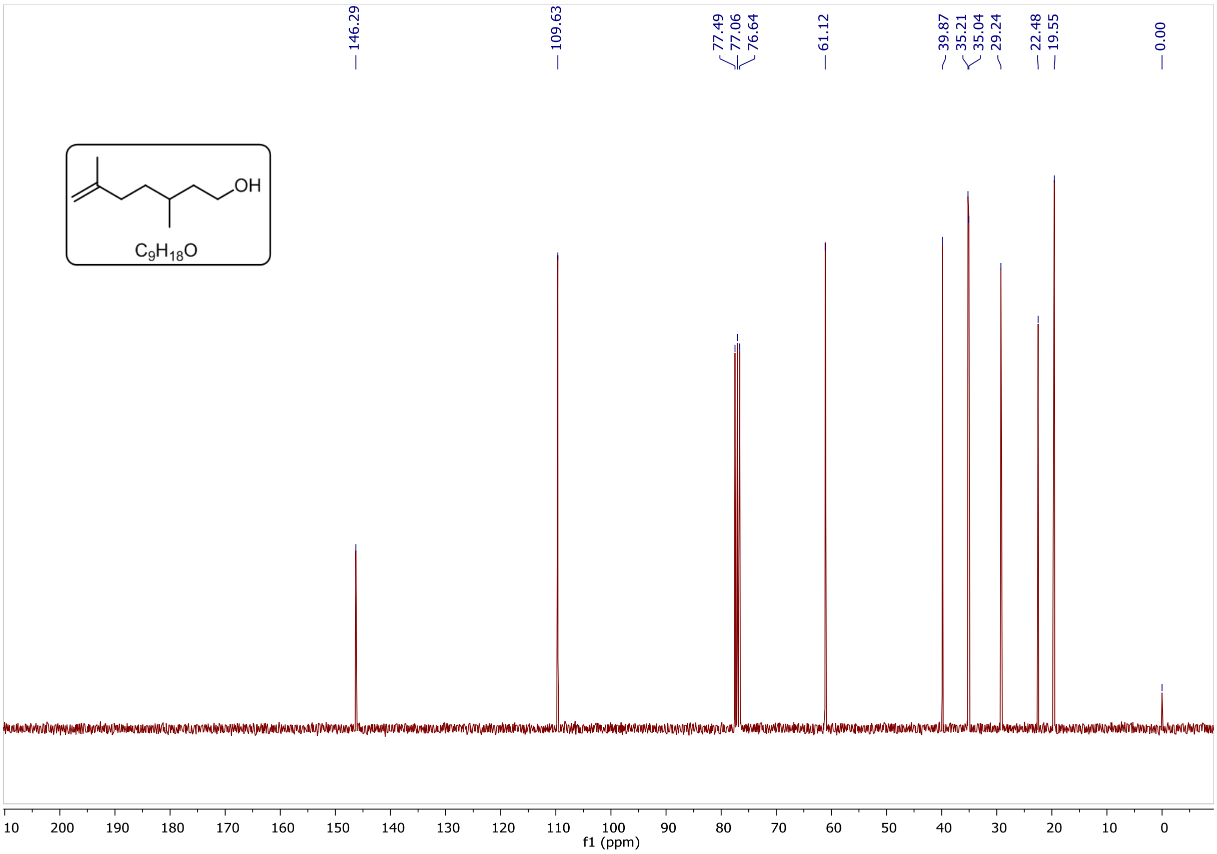

Supplementary Figure 45. ^{13}C-NMR (75 MHz) of 3,6-dimethylhept-6-en-1-ol.

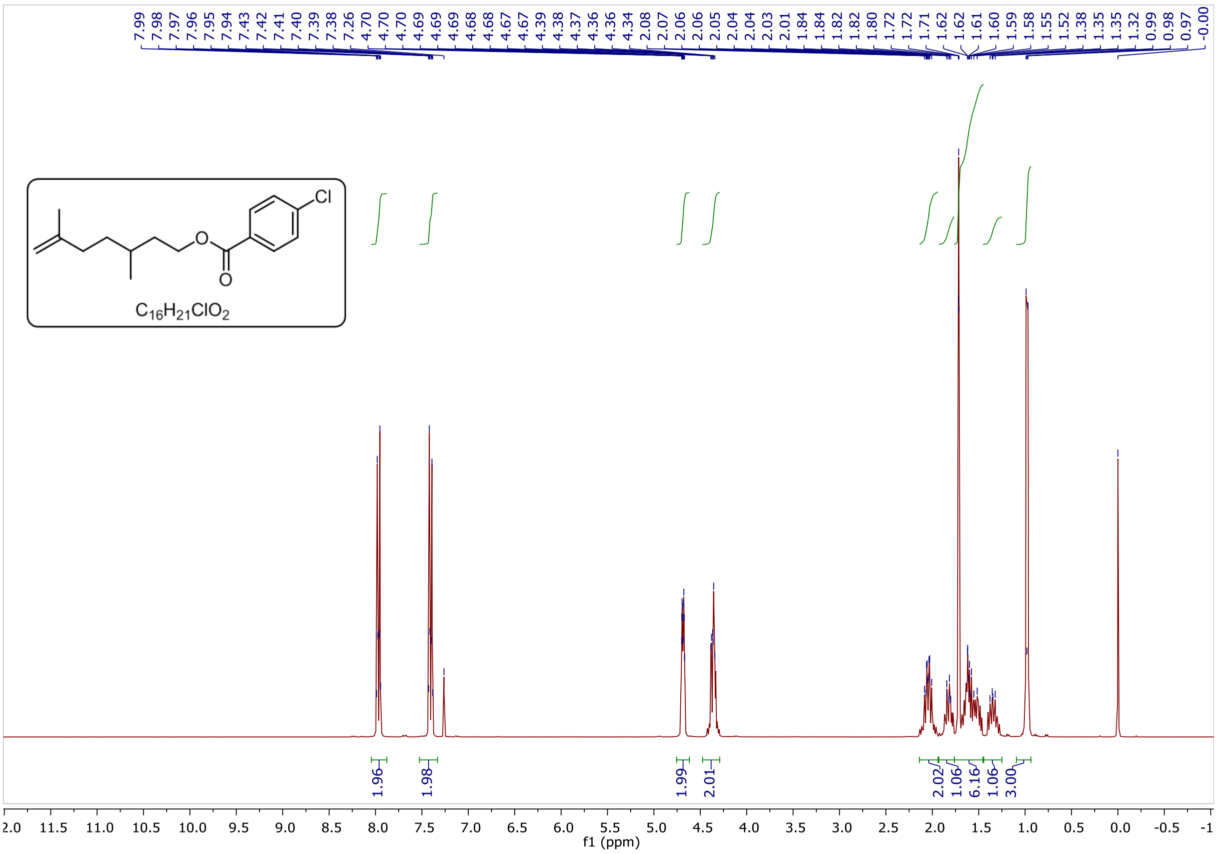

Supplementary Figure 46. ^{1}H-NMR (300 MHz) of **8b**.

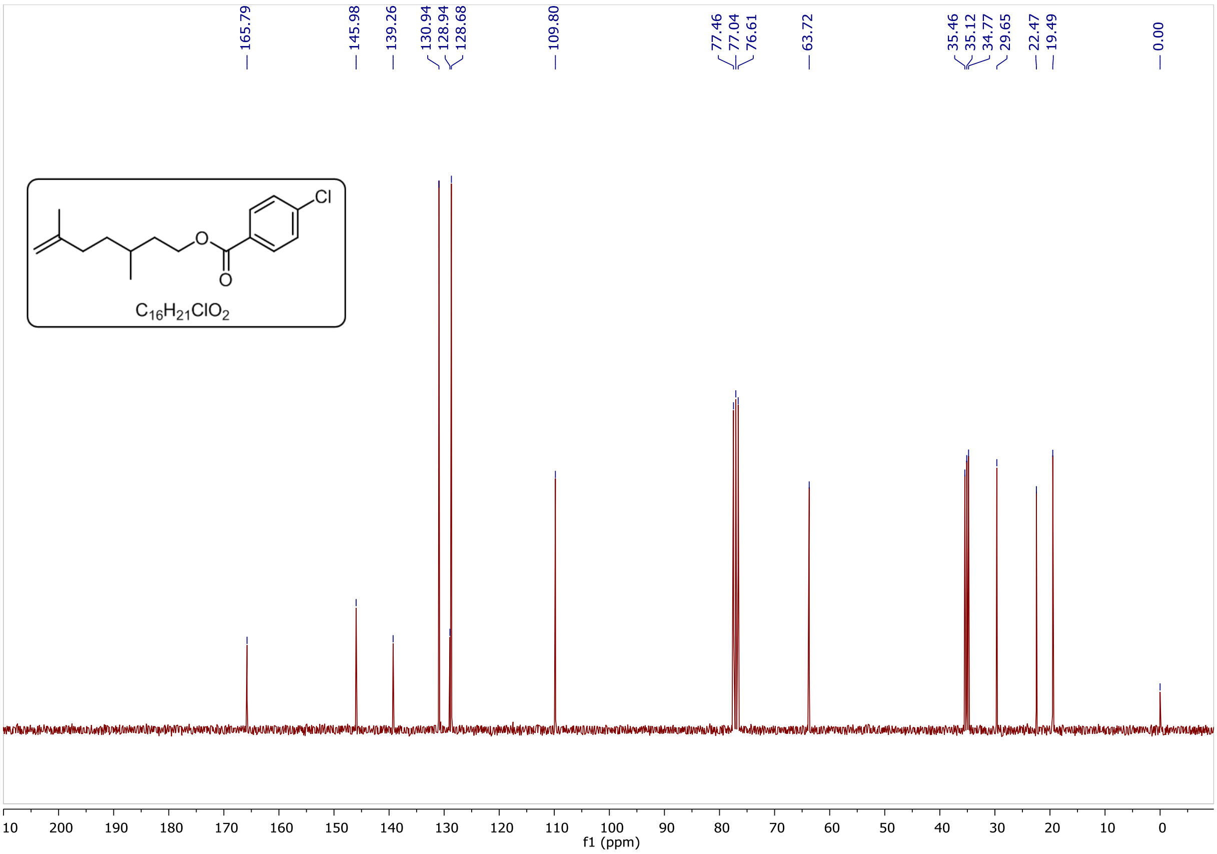

Supplementary Figure 47. ^{13}C-NMR (75 MHz) of **8b**.

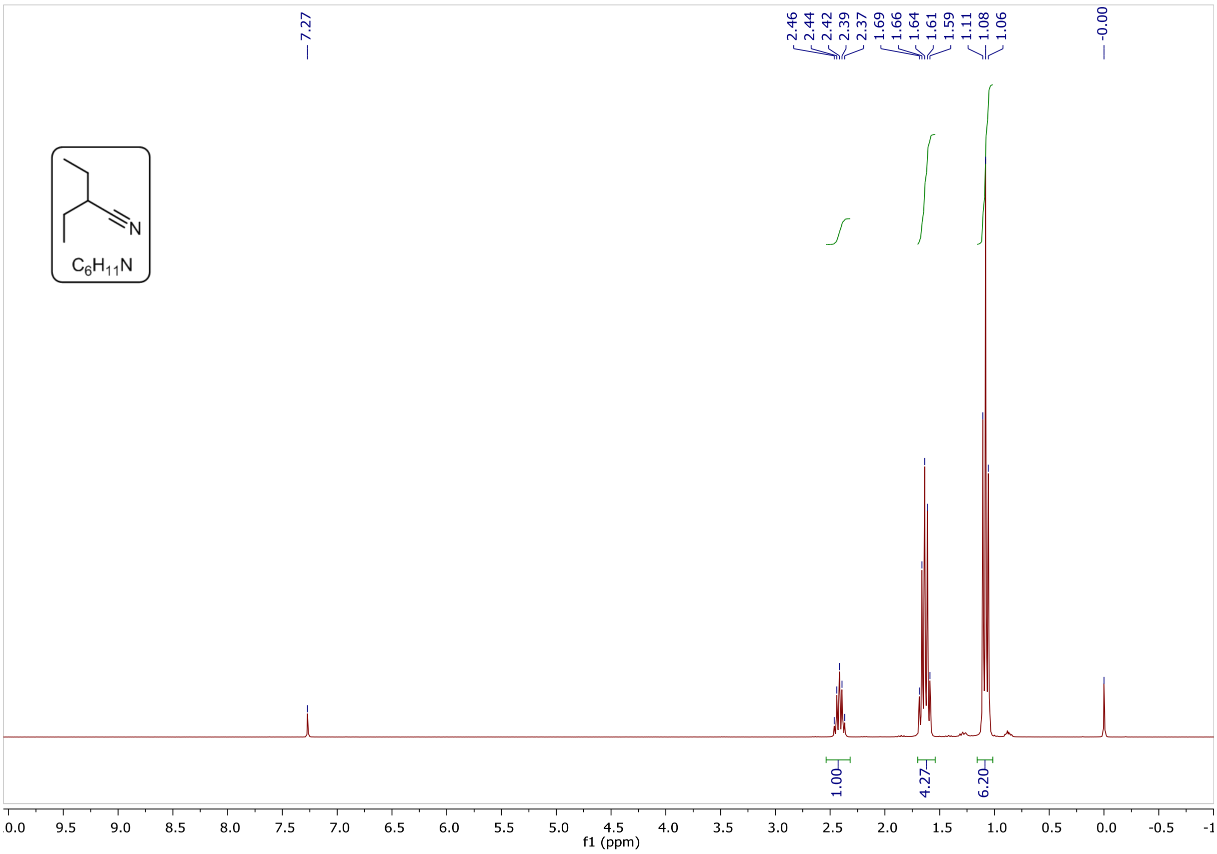

Supplementary Figure 48. ^{1}H-NMR (300 MHz) of 2-ethylbutanenitrile.

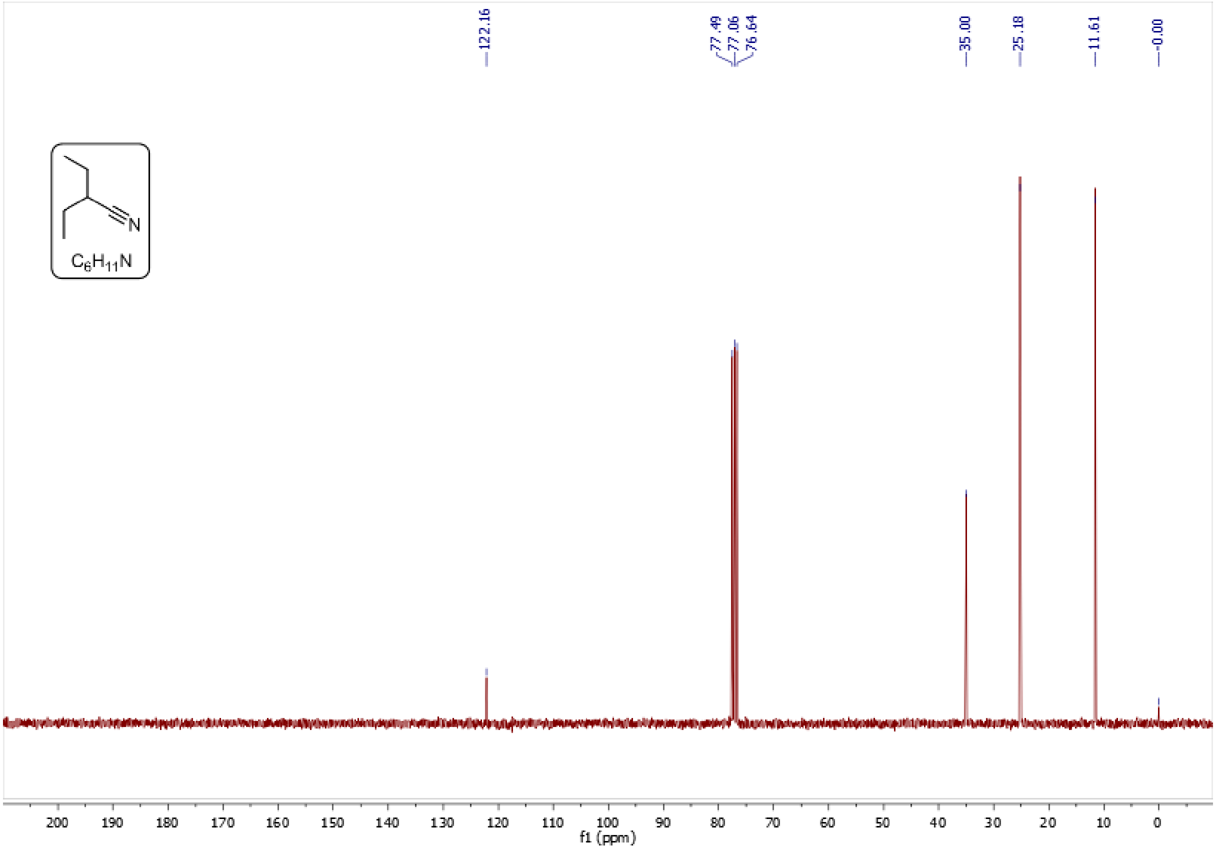

Supplementary Figure 49. ^{13}C-NMR (75 MHz) of 2-ethylbutanenitrile.

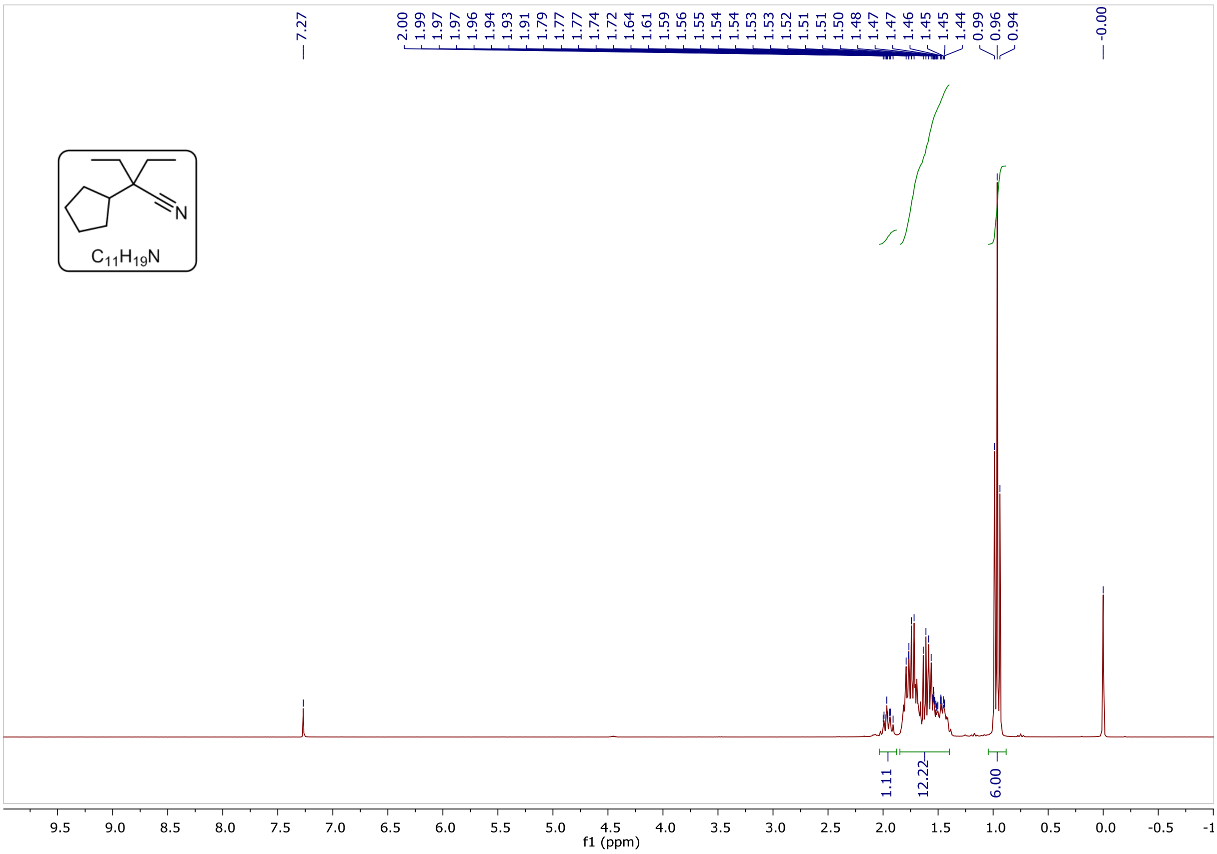

Supplementary Figure 50. ^{1}H-NMR (300 MHz) of 2-cyclopentyl-2-ethylbutanenitrile.

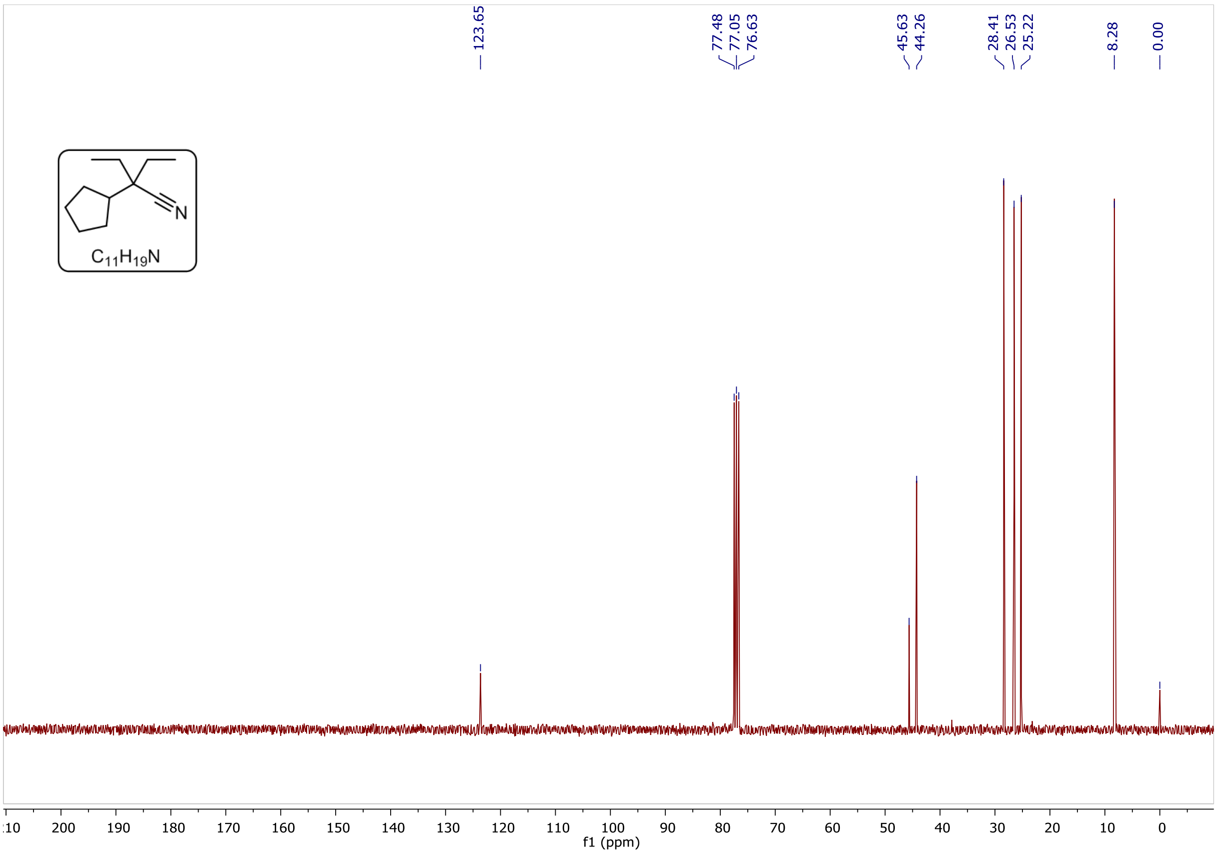

Supplementary Figure 51. ^{13}C-NMR (75 MHz) of 2-cyclopentyl-2-ethylbutanenitrile.

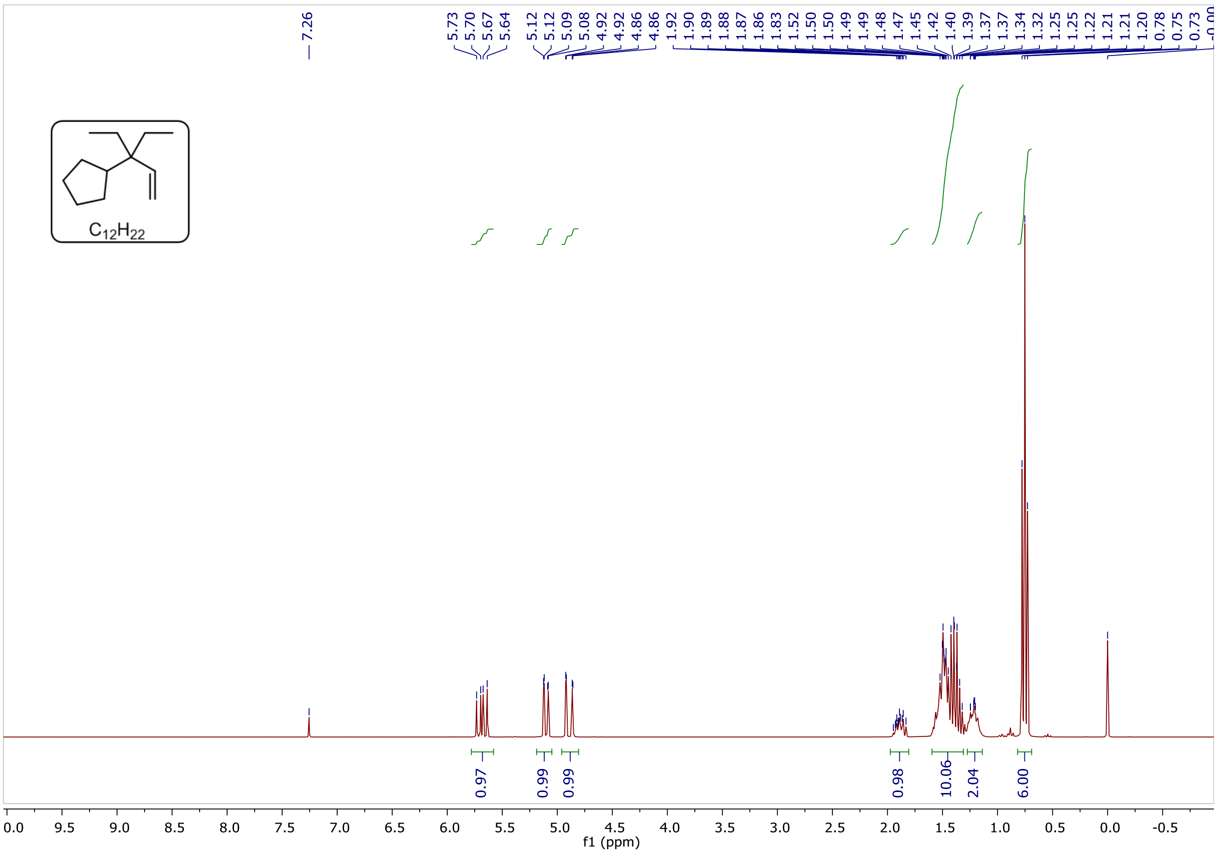

Supplementary Figure 52. ^{1}H-NMR (300 MHz) of **11**.

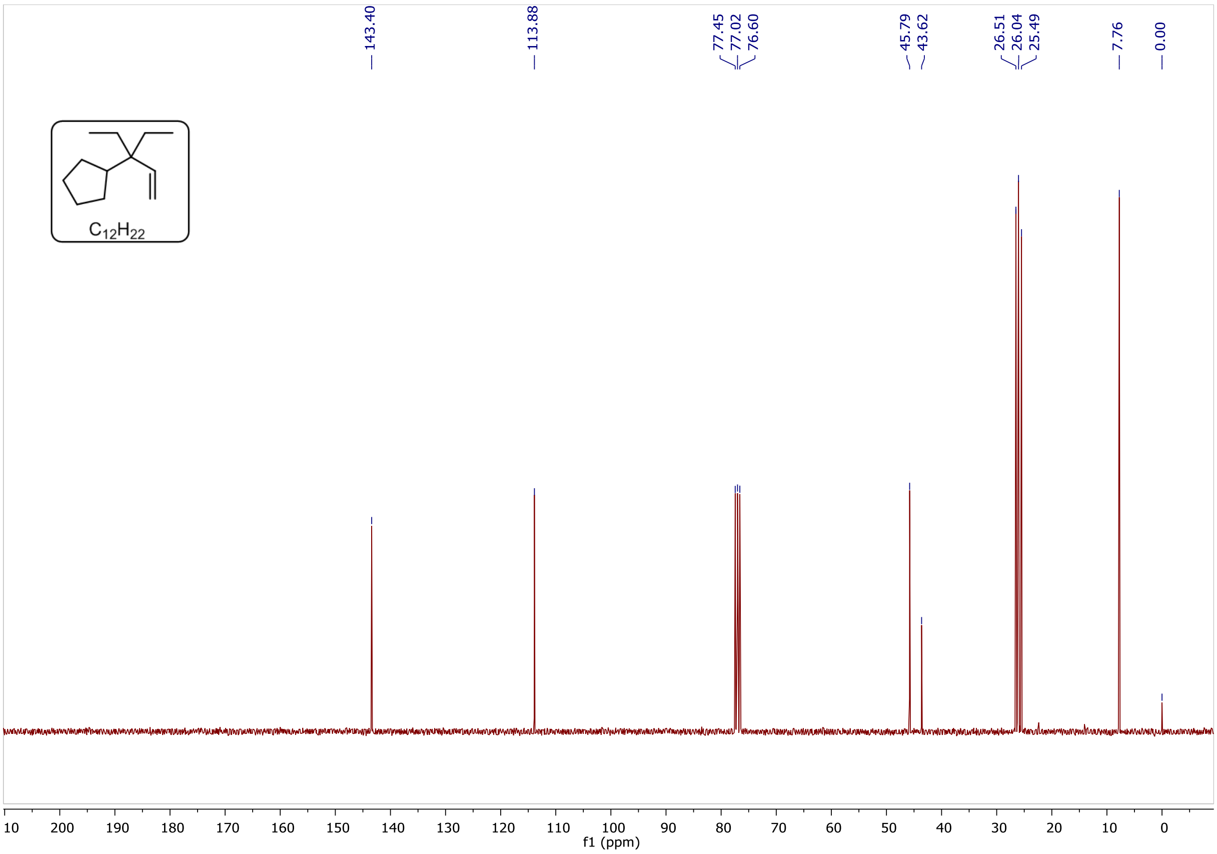

Supplementary Figure 53. ^{13}C-NMR (75 MHz) of **11**.

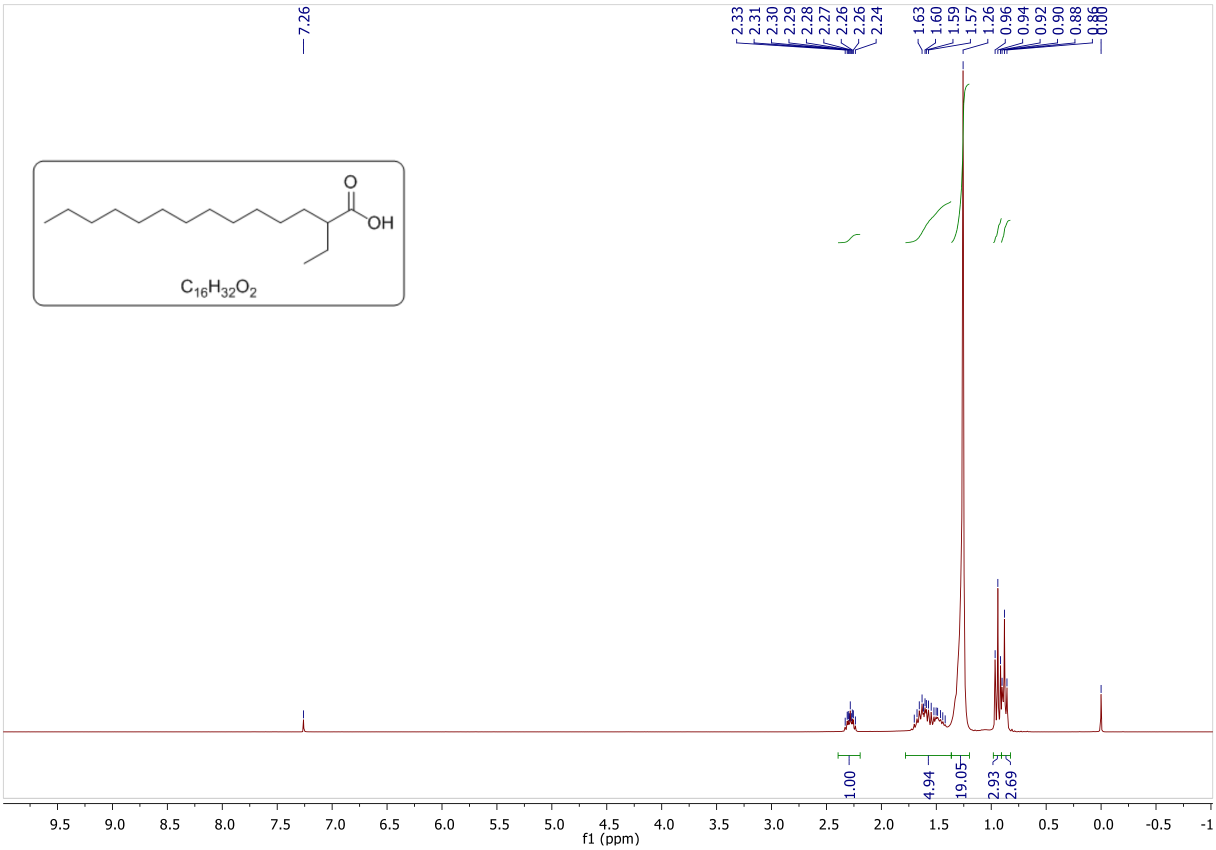

Supplementary Figure 54. ^{1}H-NMR (300 MHz) of 2-ethyltetradecanoic acid.

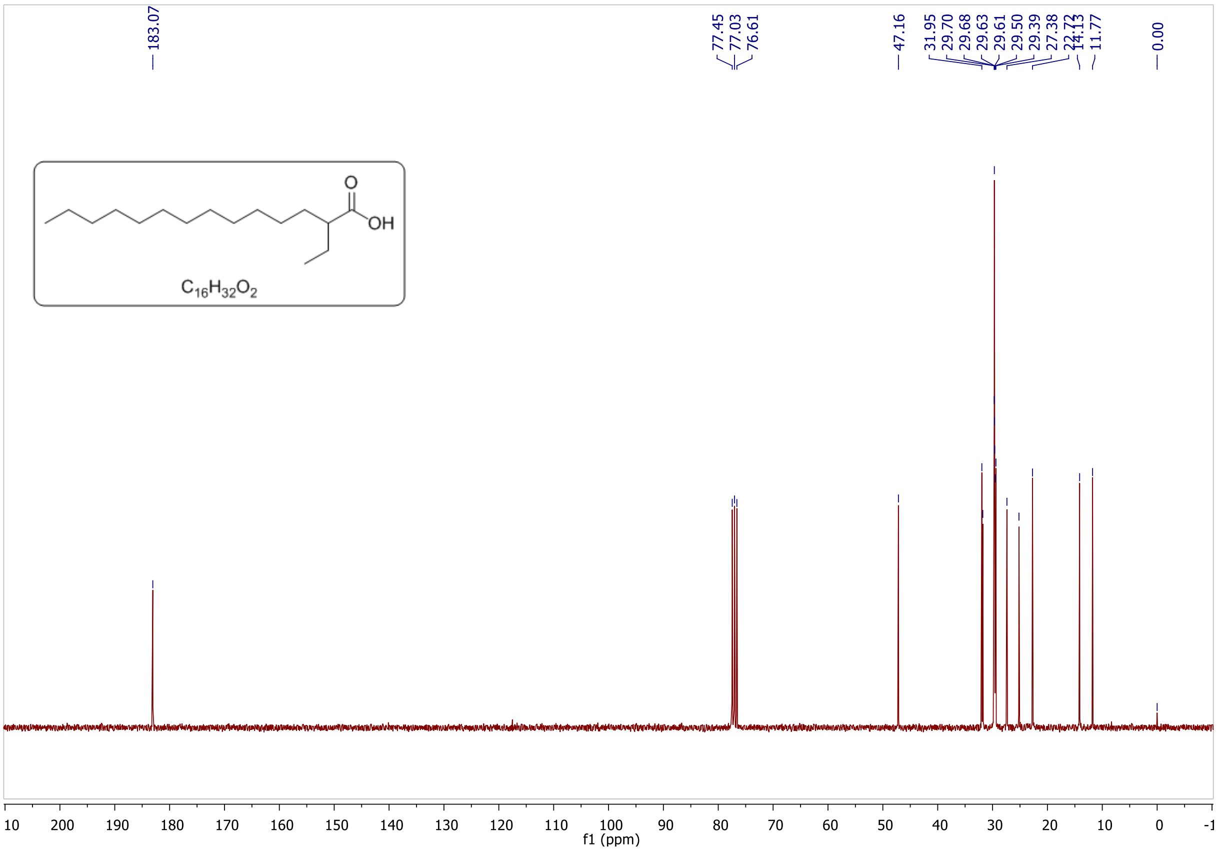

Supplementary Figure 55. ^{13}C-NMR (75 MHz) of 2-ethyltetradecanoic acid.

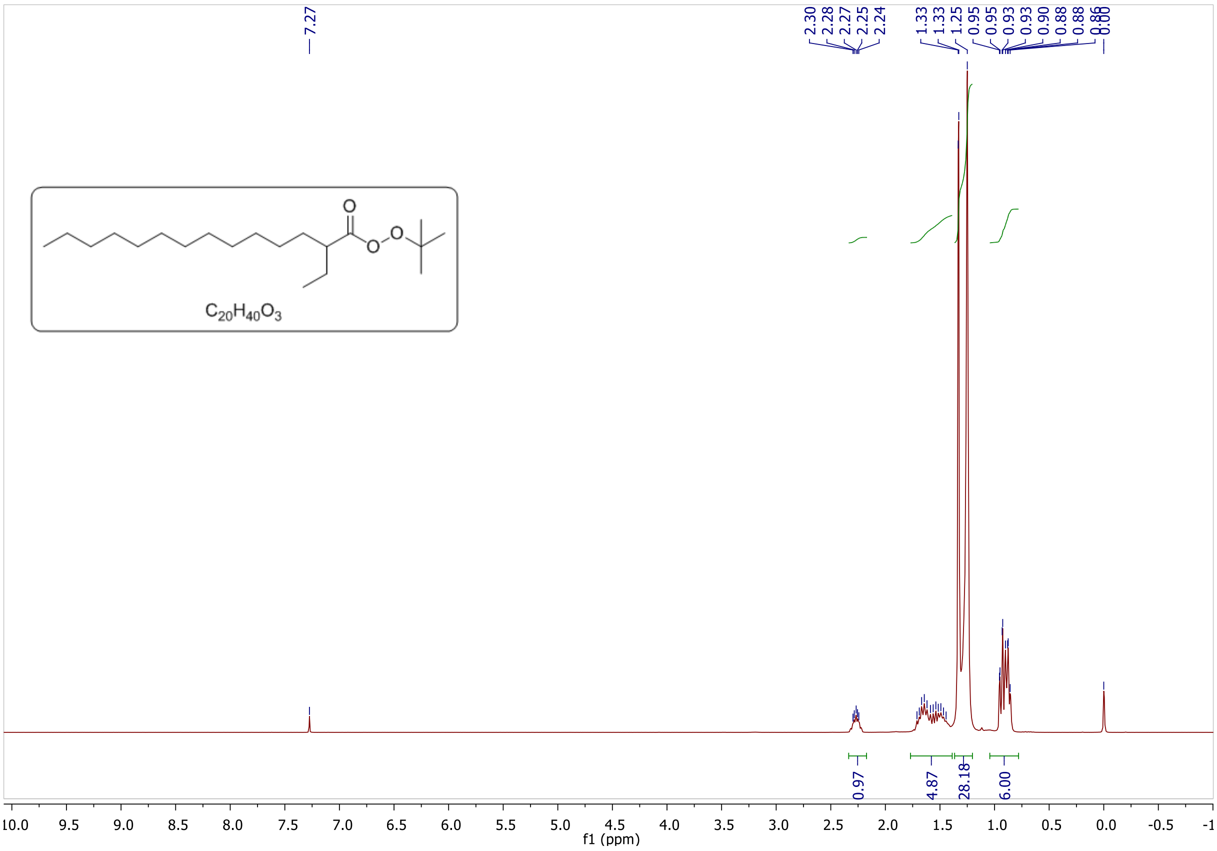

Supplementary Figure 56. ^{1}H-NMR (300 MHz) of **13a**.

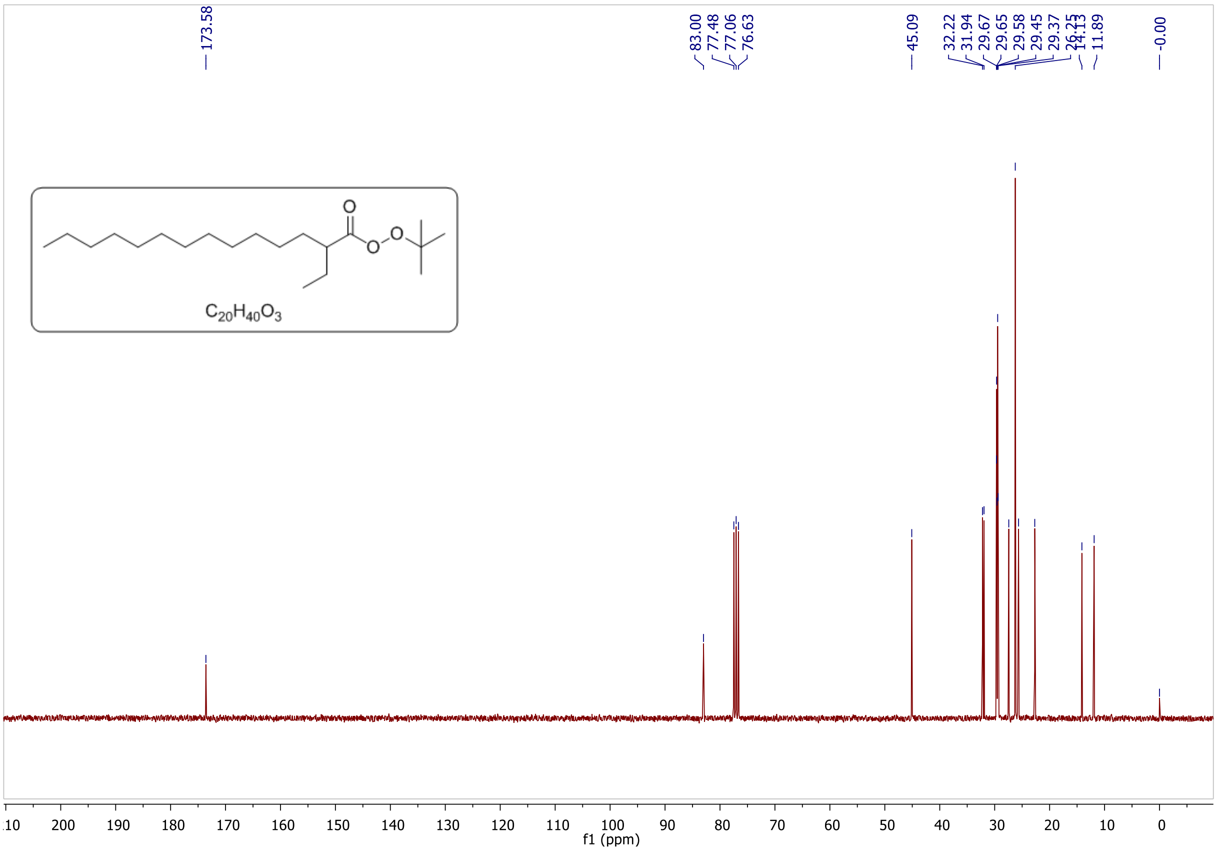

Supplementary Figure 57. ^{13}C-NMR (75 MHz) of **13a**.

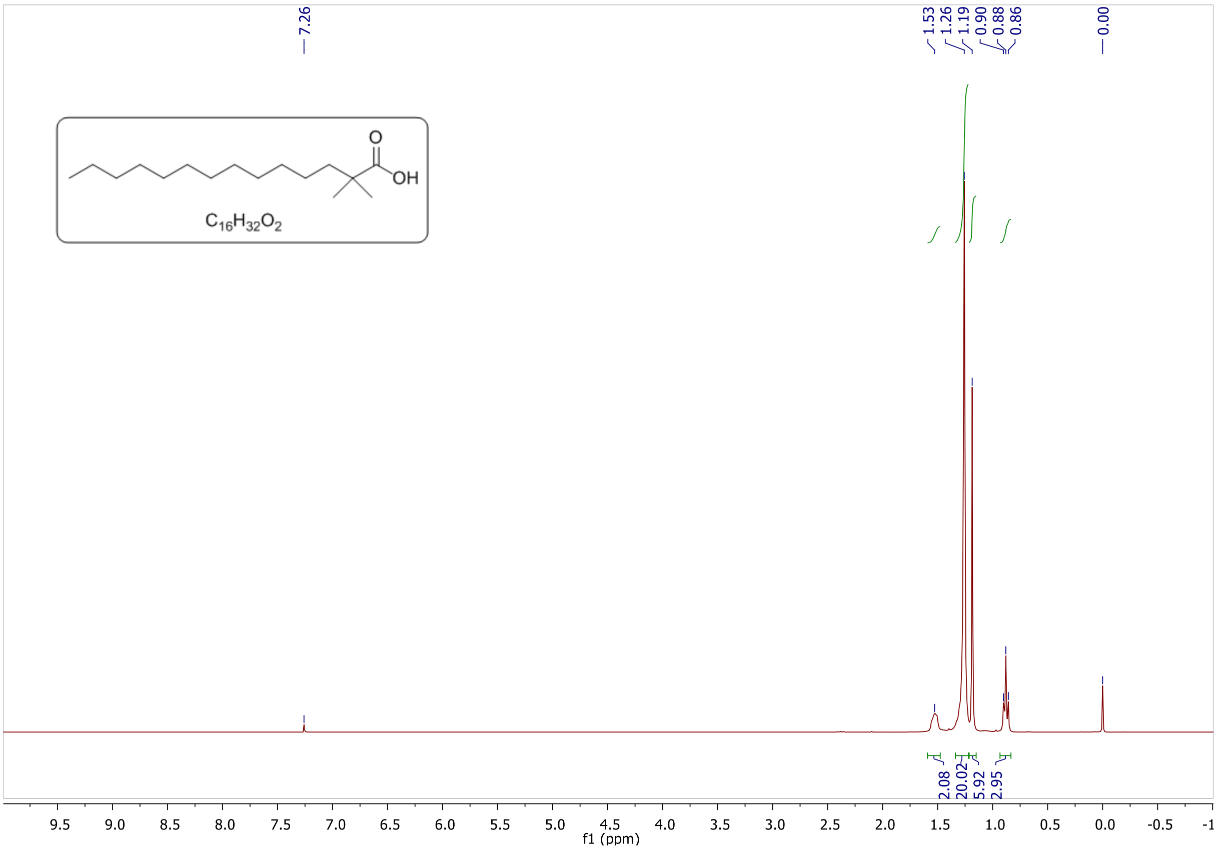

Supplementary Figure 58. ^{1}H-NMR (300 MHz) of 2,2-dimethyltetradecanoic acid.

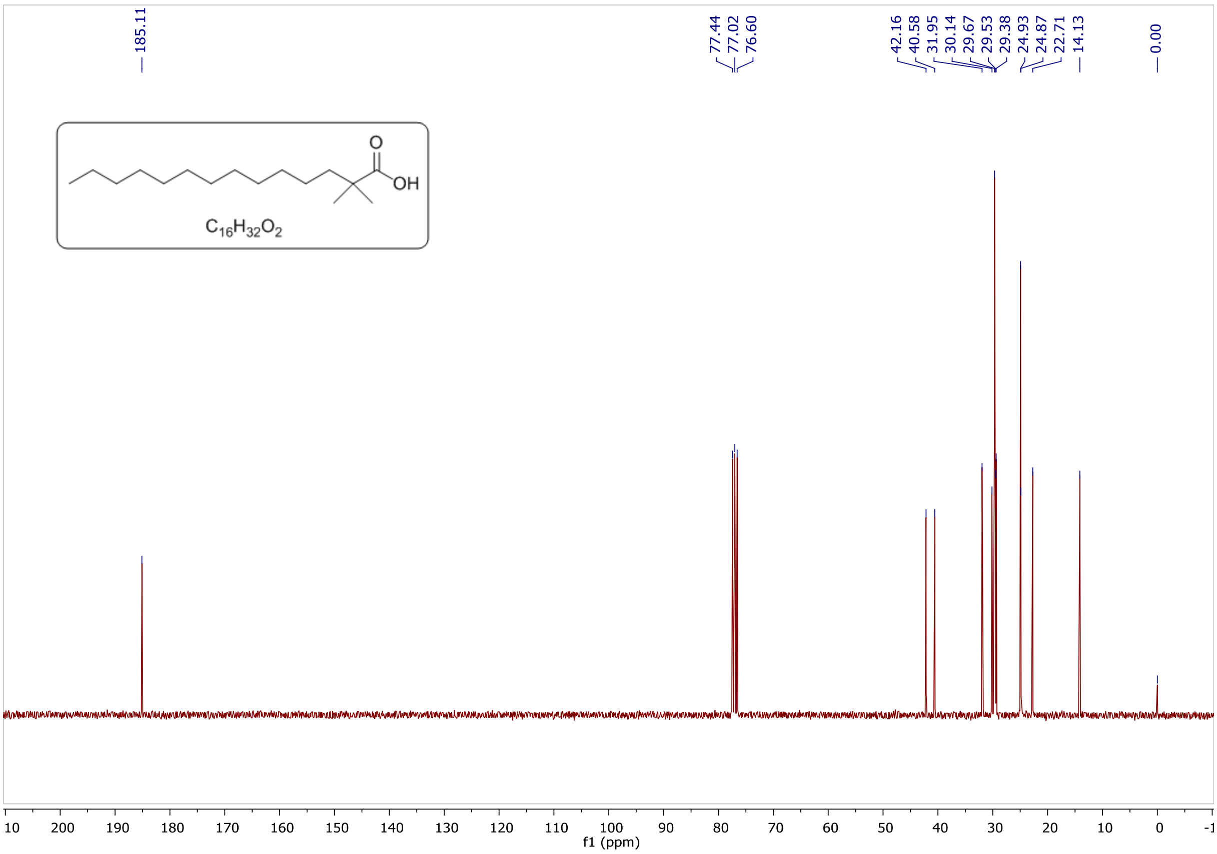

Supplementary Figure 59. ^{13}C-NMR (75 MHz) of 2,2-dimethyltetradecanoic acid.

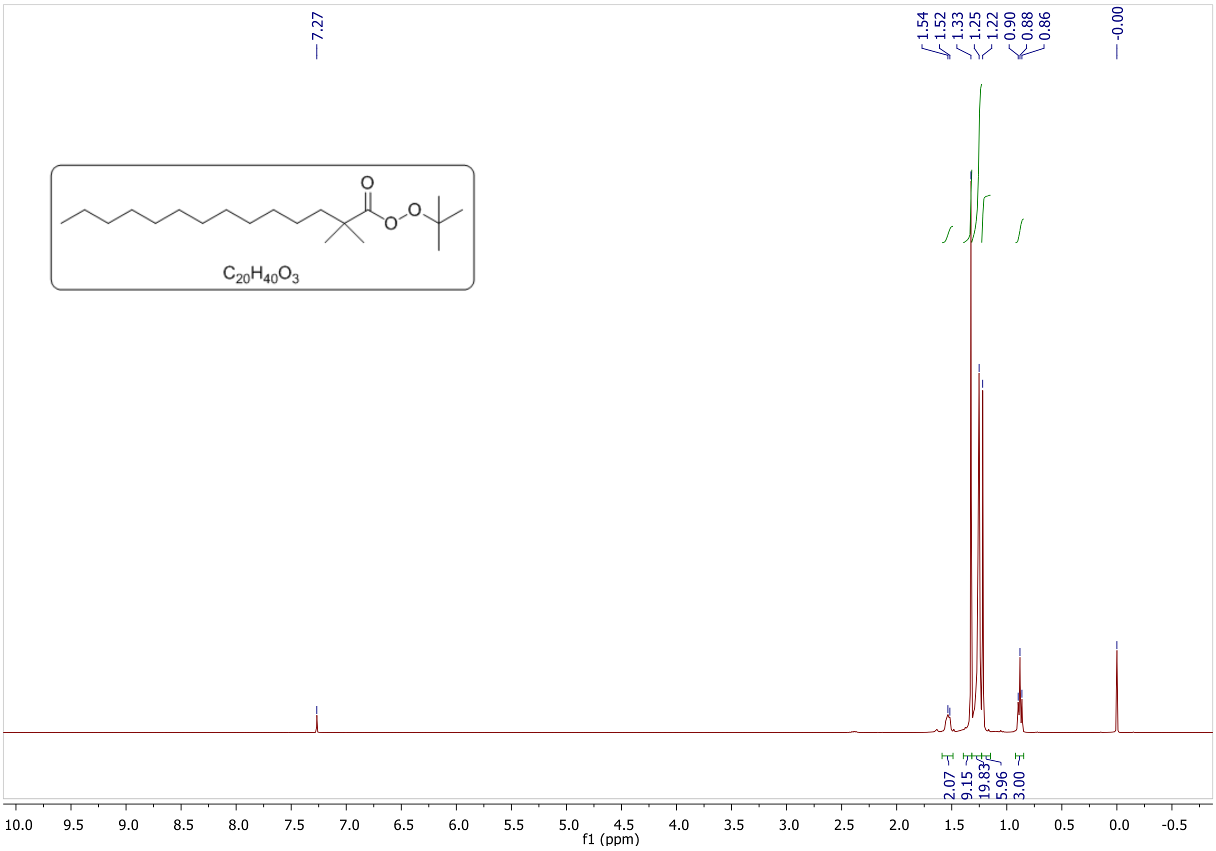

Supplementary Figure 60. ^{1}H-NMR (300 MHz) of **13b**.

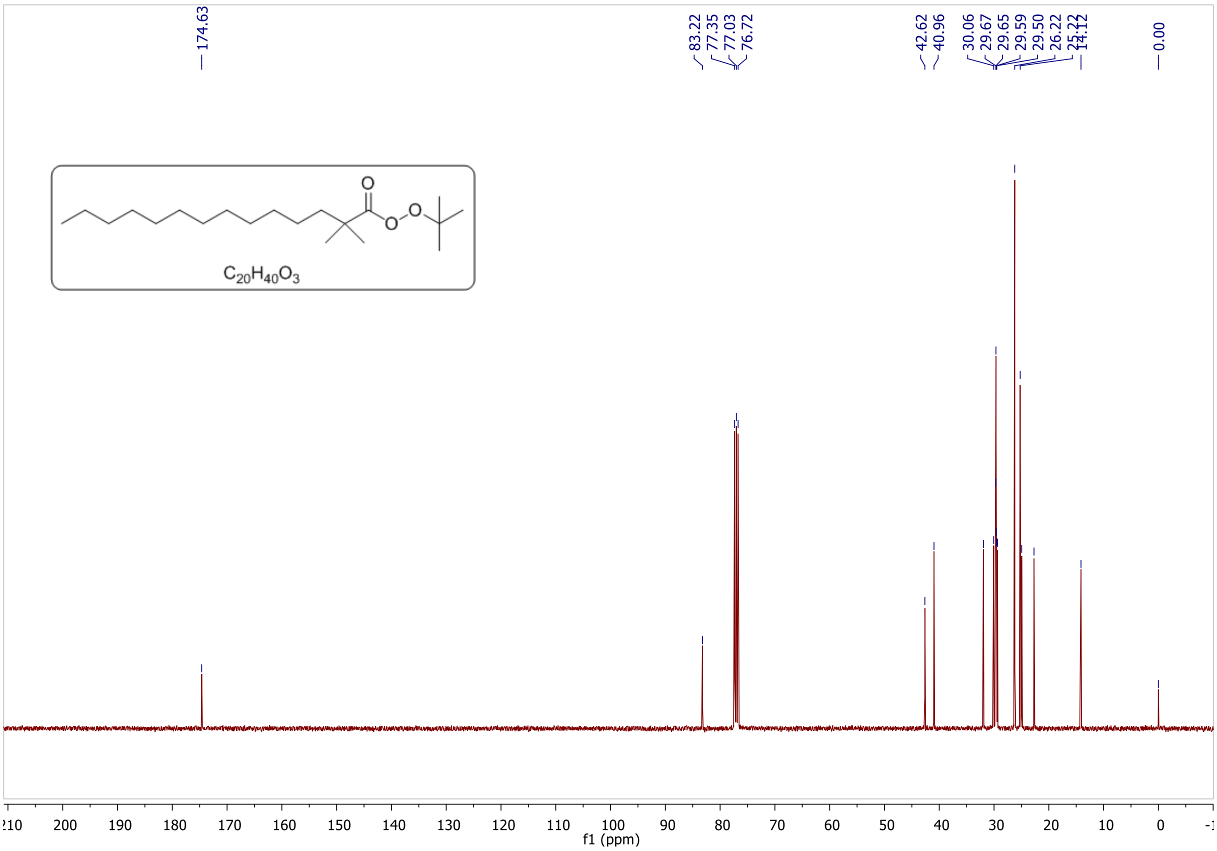

Supplementary Figure 61. ^{13}C-NMR (75 MHz) of **13b**.

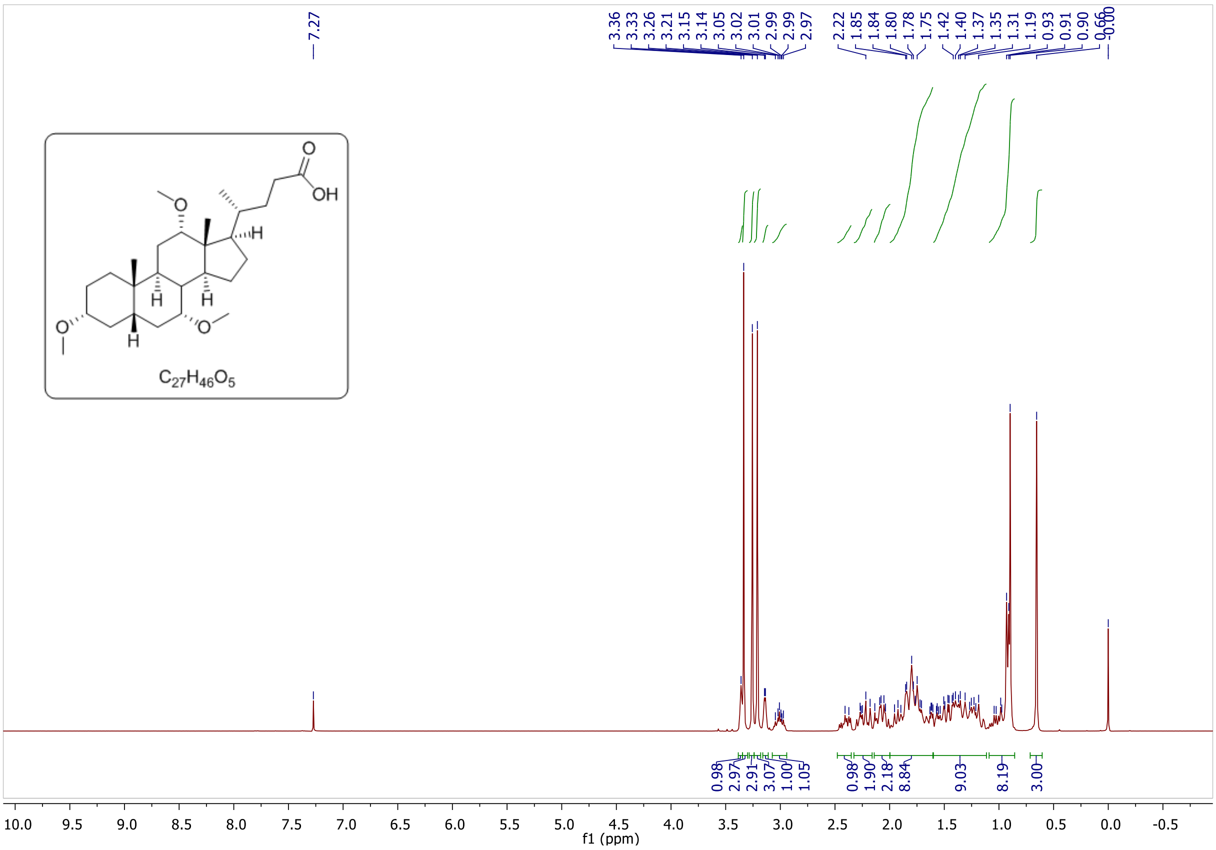

Supplementary Figure 62. ^{1}H-NMR (300 MHz) of trimethoxy cholic acid.

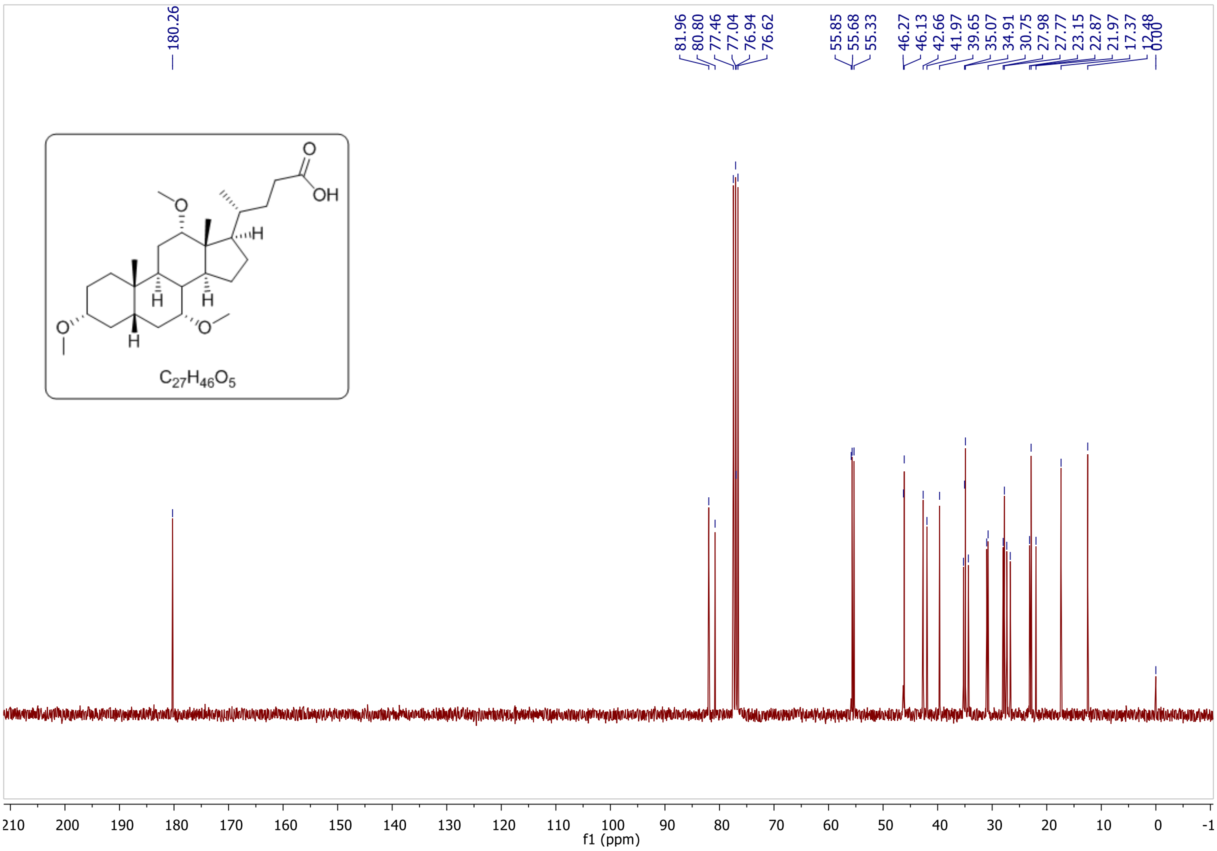

Supplementary Figure 63. ^{13}C-NMR (75 MHz) of trimethoxy cholic acid.

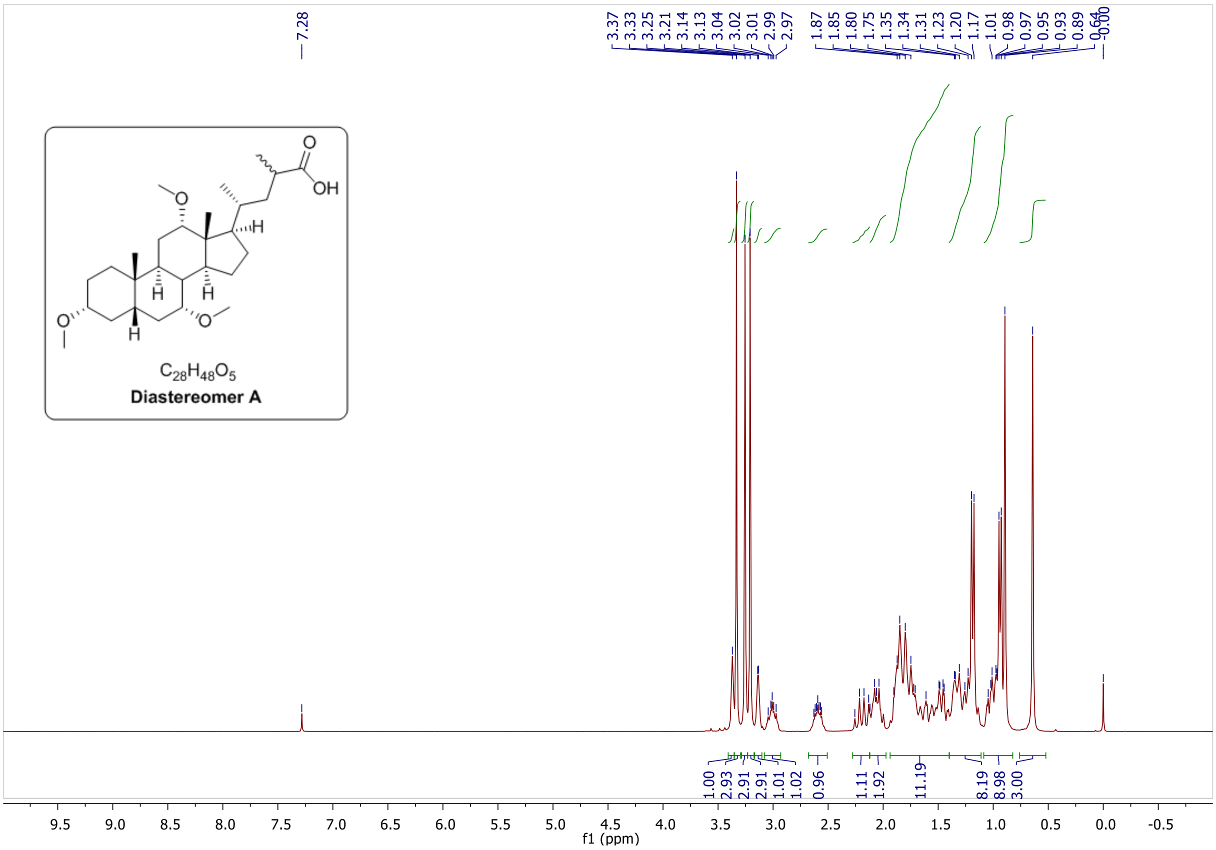

Supplementary Figure 64. ^{1}H-NMR (300 MHz) of α-methylated trimethoxy cholic acid (Diastereom. A).

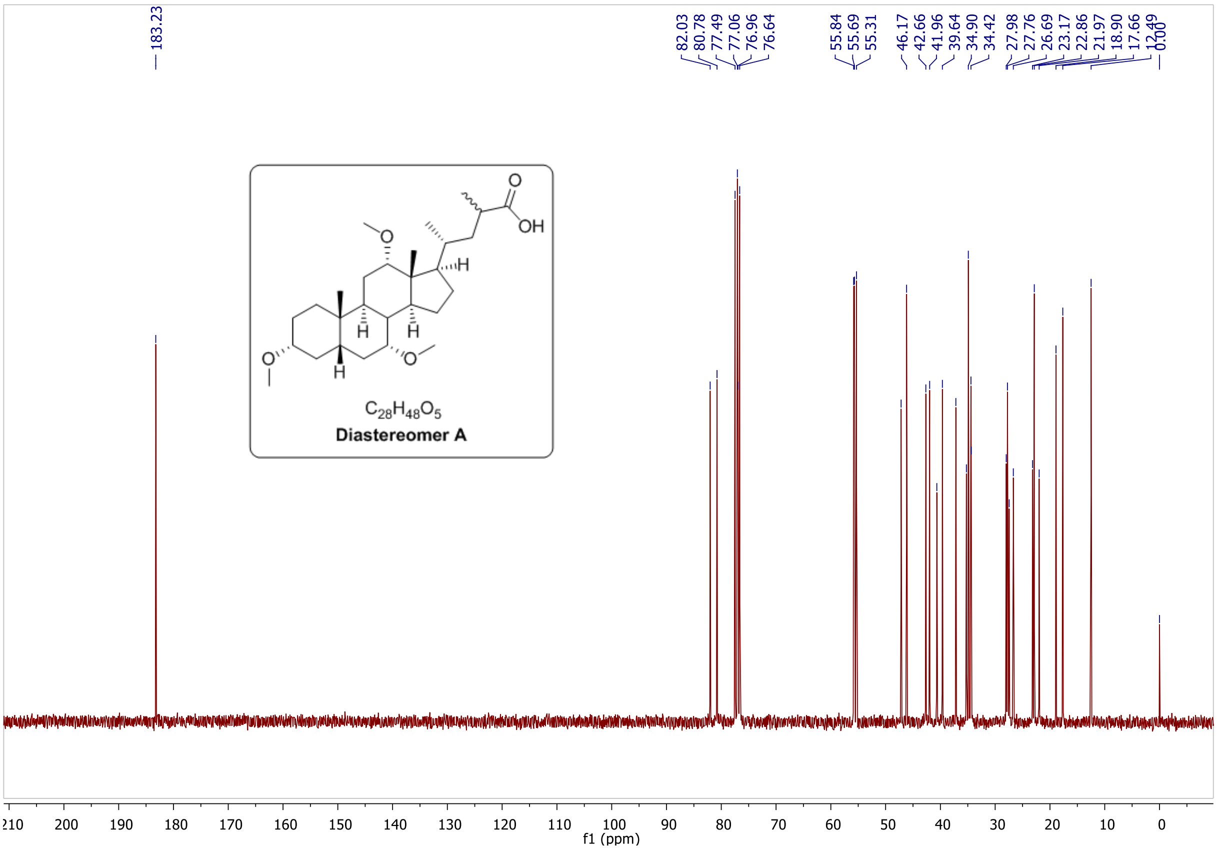

Supplementary Figure 65. ^{13}C-NMR (75 MHz) of α-methylated trimethoxy cholic acid (Diastereom. A).

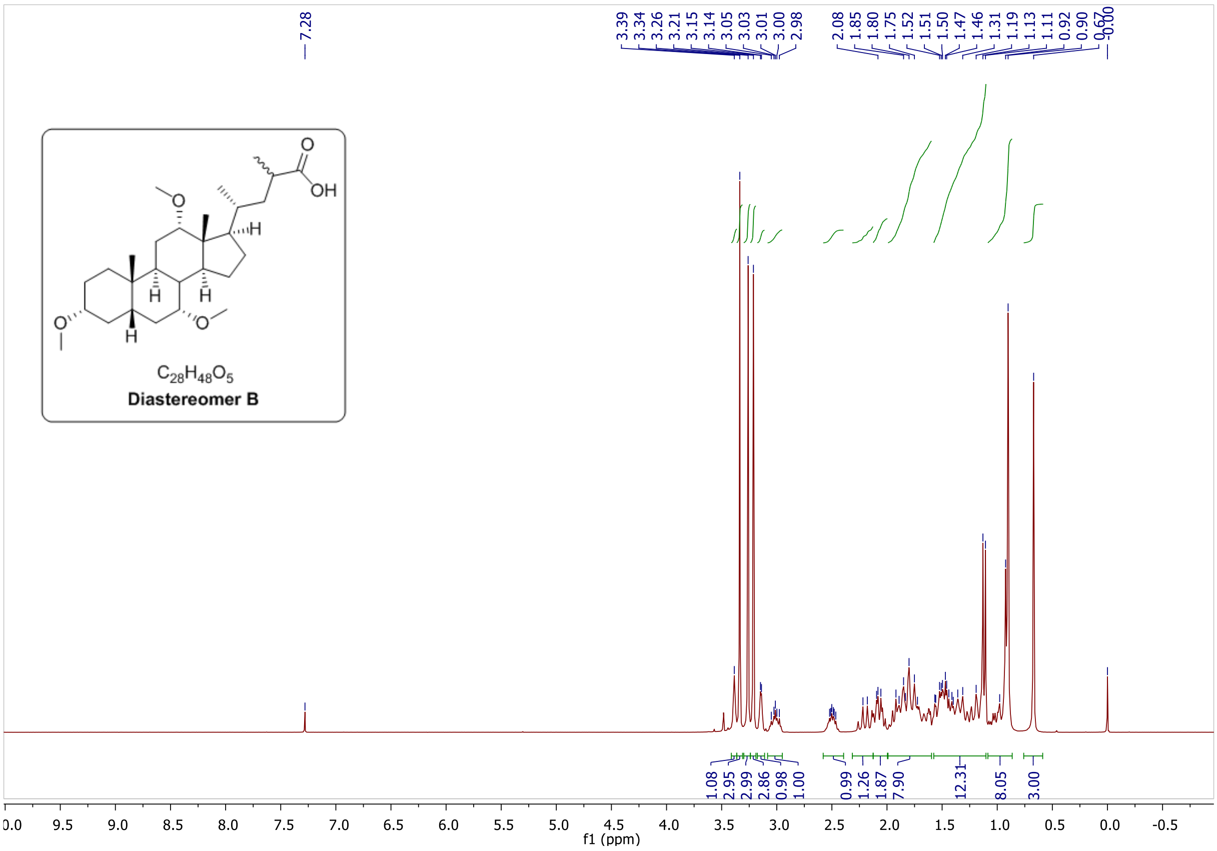

Supplementary Figure 66. ^{1}H-NMR (300 MHz) of α-methylated trimethoxy cholic acid (Diastereom. B).

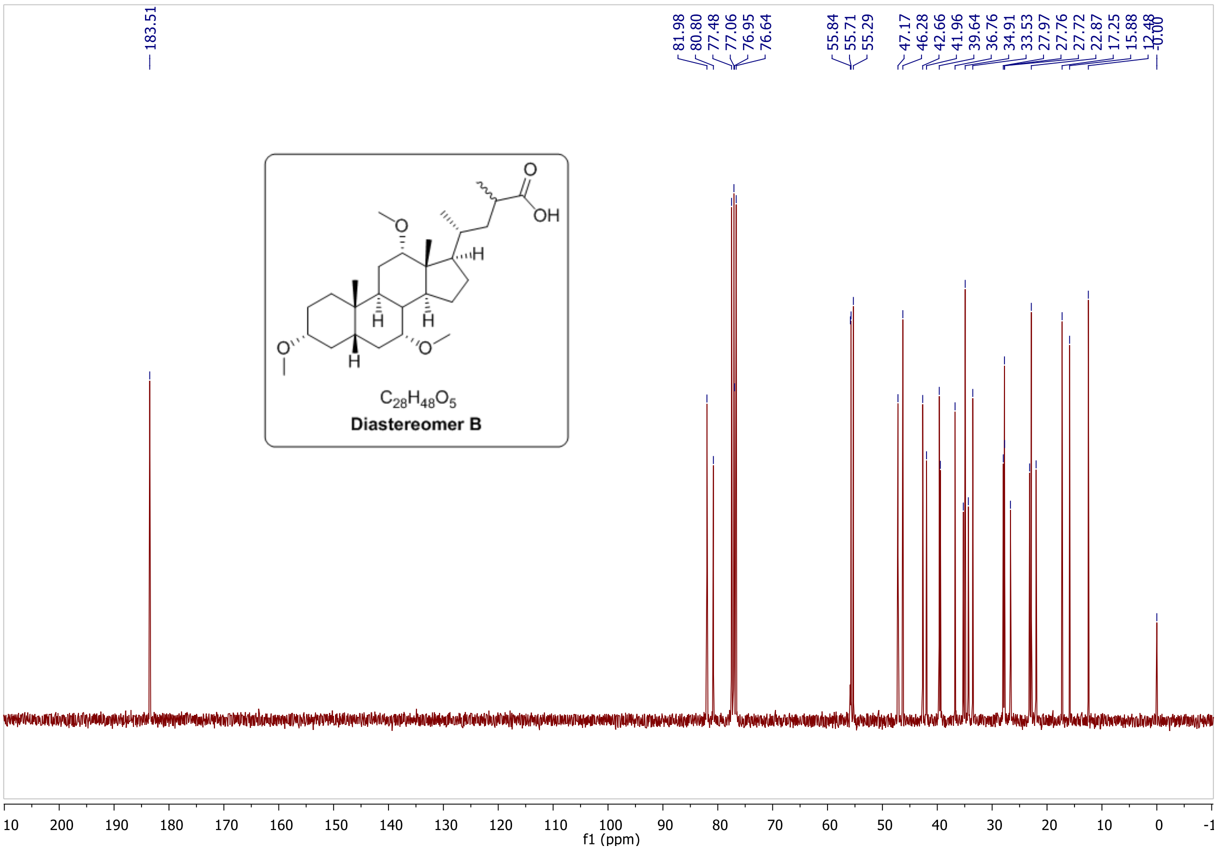

Supplementary Figure 67. ^{13}C-NMR (75 MHz) of α-methylated trimethoxy cholic acid (Diastereom. B).

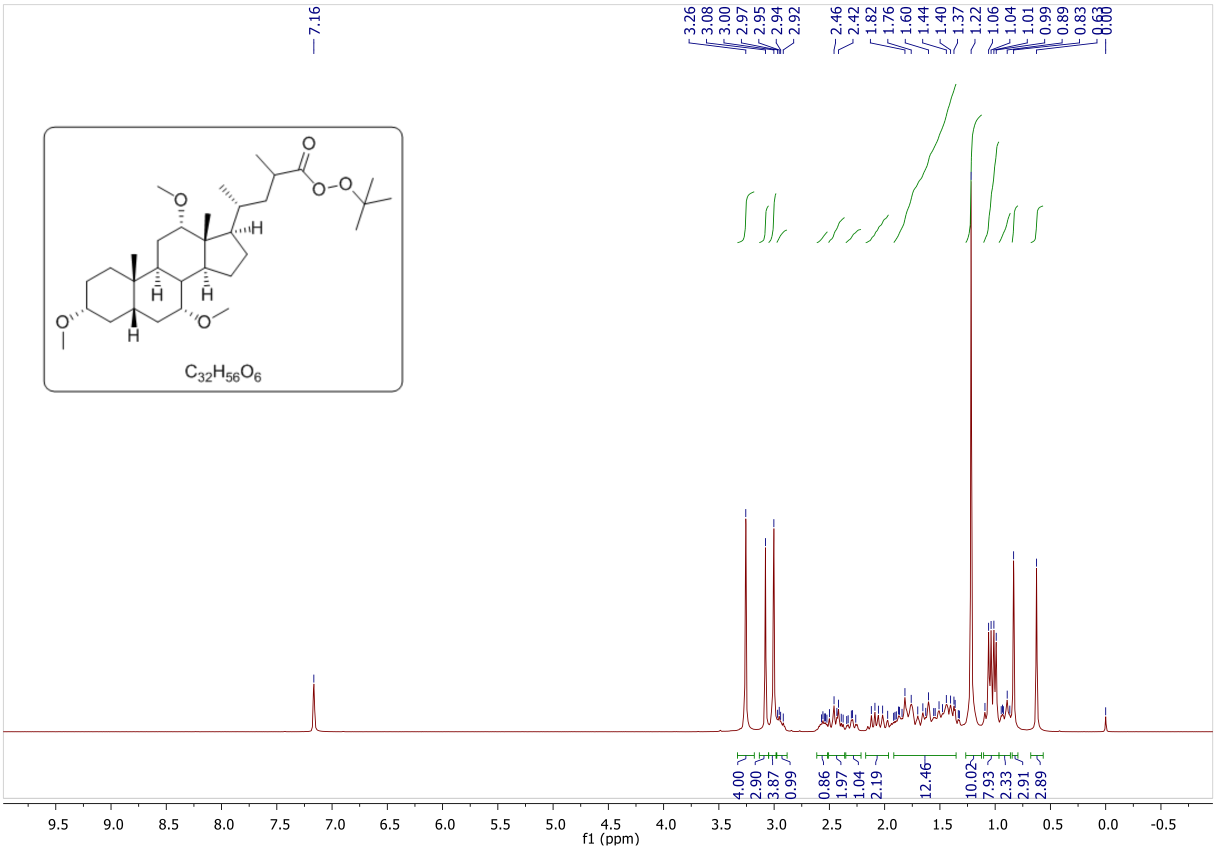

Supplementary Figure 68. ^{1}H-NMR (300 MHz) **13c**.

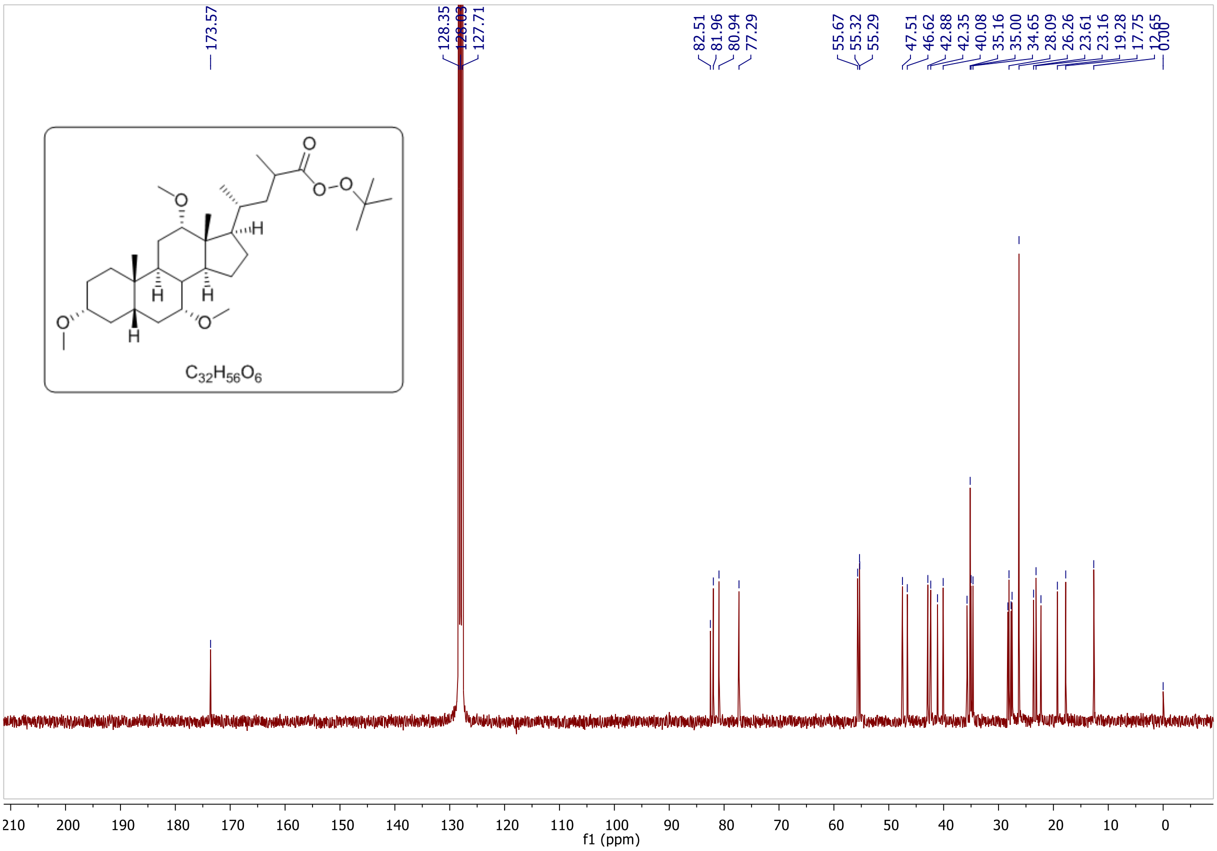

Supplementary Figure 69. ^{13}C-NMR (75 MHz) of **13c**.

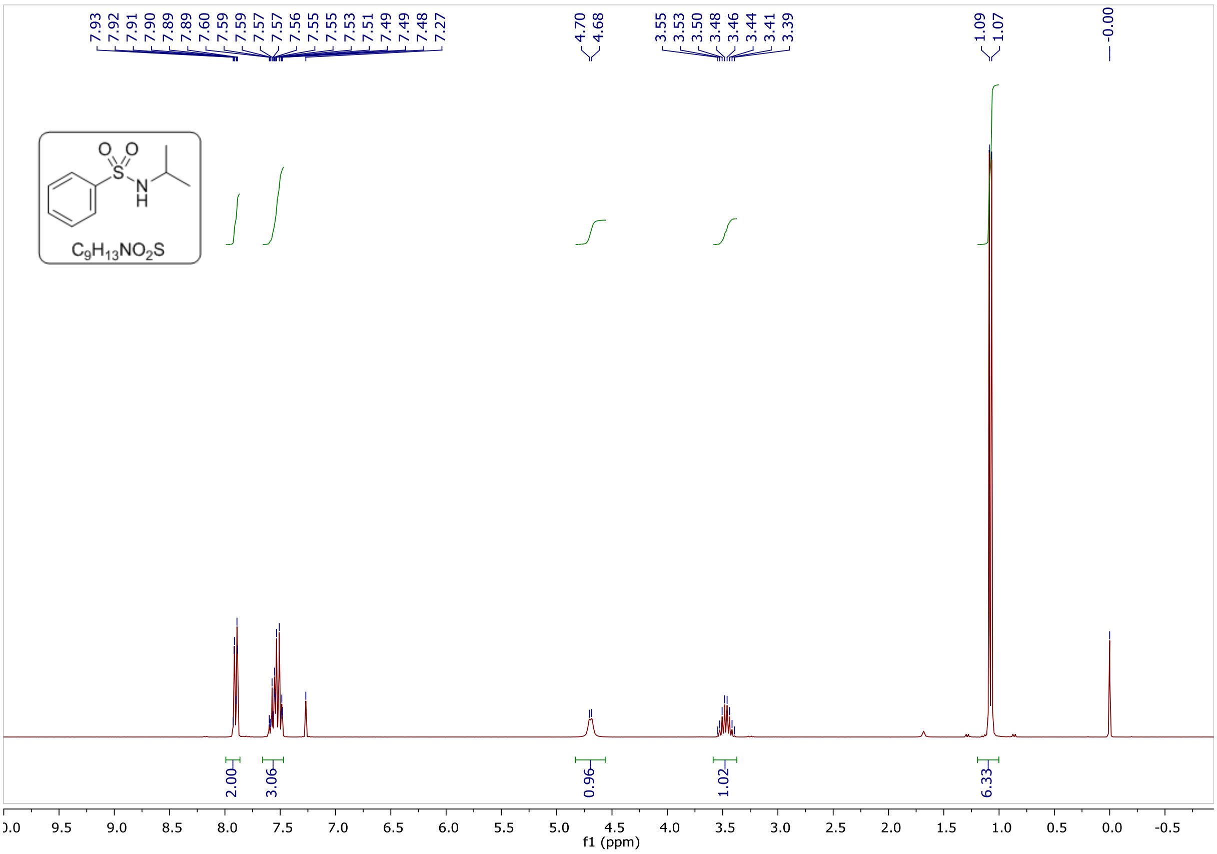

Supplementary Figure 70. ^{1}H-NMR (300 MHz) **S1-a**.

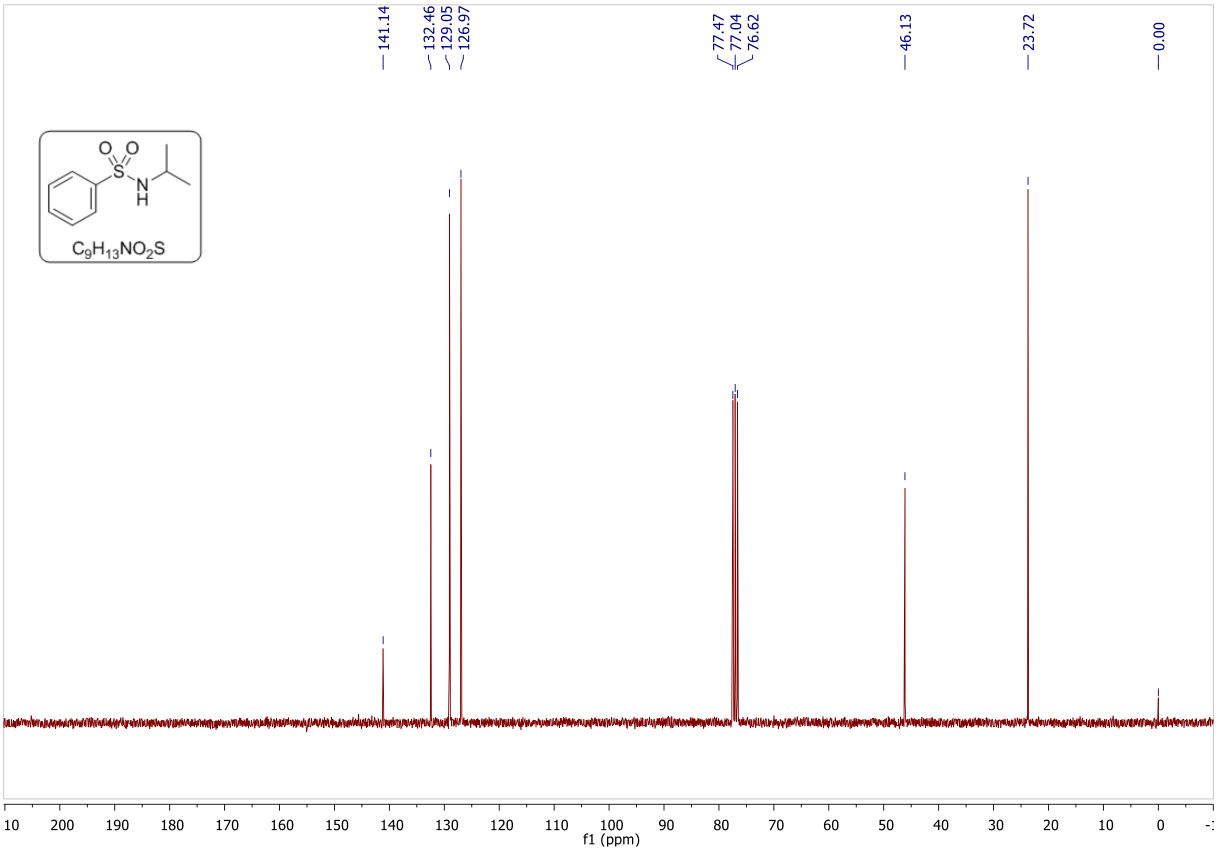

Supplementary Figure 71. ^{13}C-NMR (75 MHz) of **S1-a**.

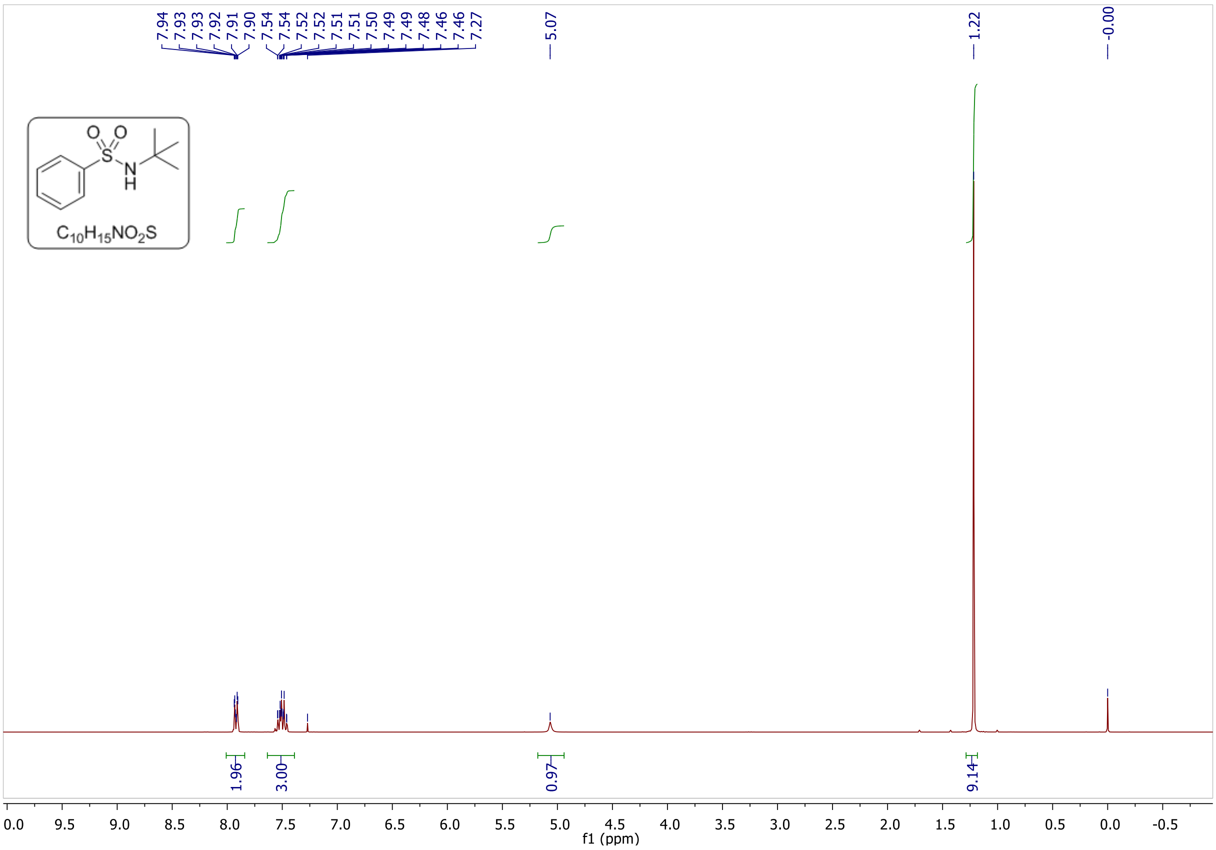

Supplementary Figure 72. ^{1}H-NMR (300 MHz) **S1-b**.

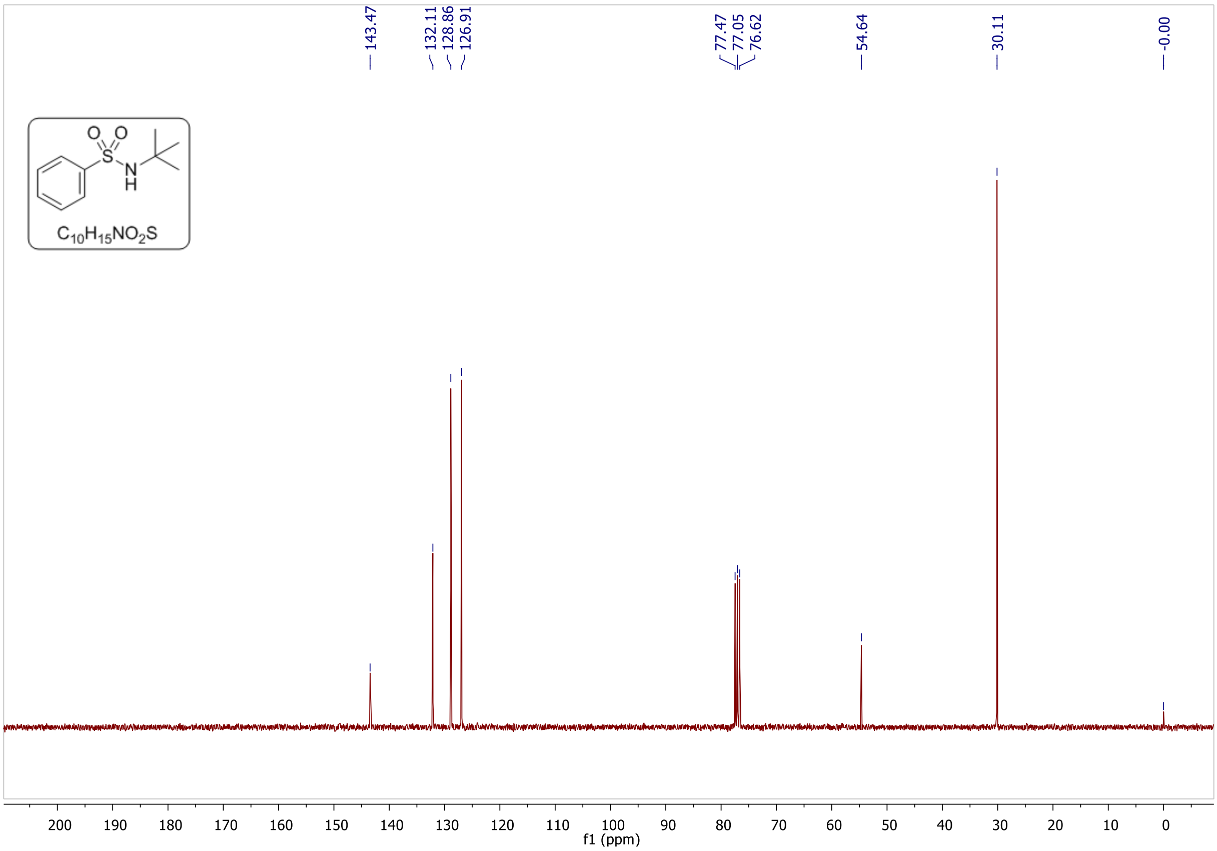

Supplementary Figure 73. ^{13}C-NMR (75 MHz) of **S1-b**.

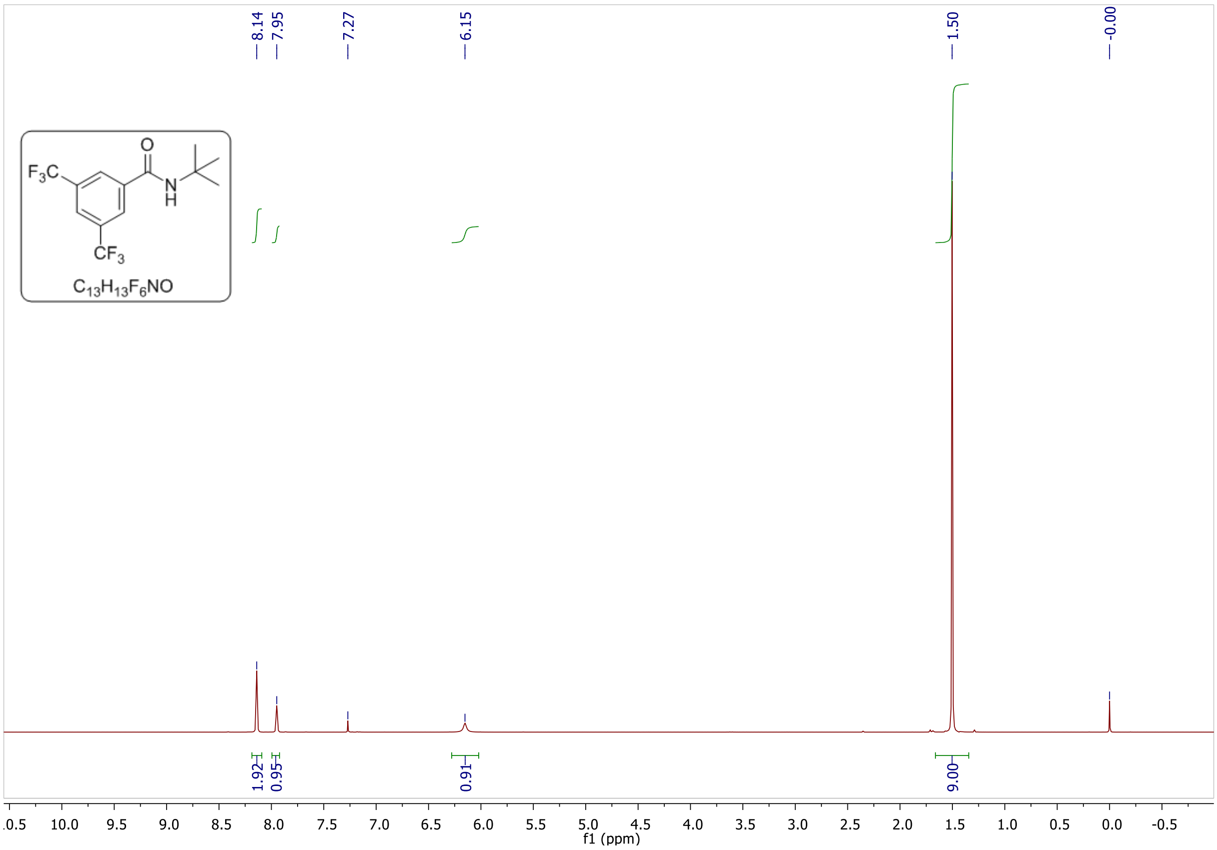

Supplementary Figure 74. ^{1}H-NMR (300 MHz) **S1-c**.

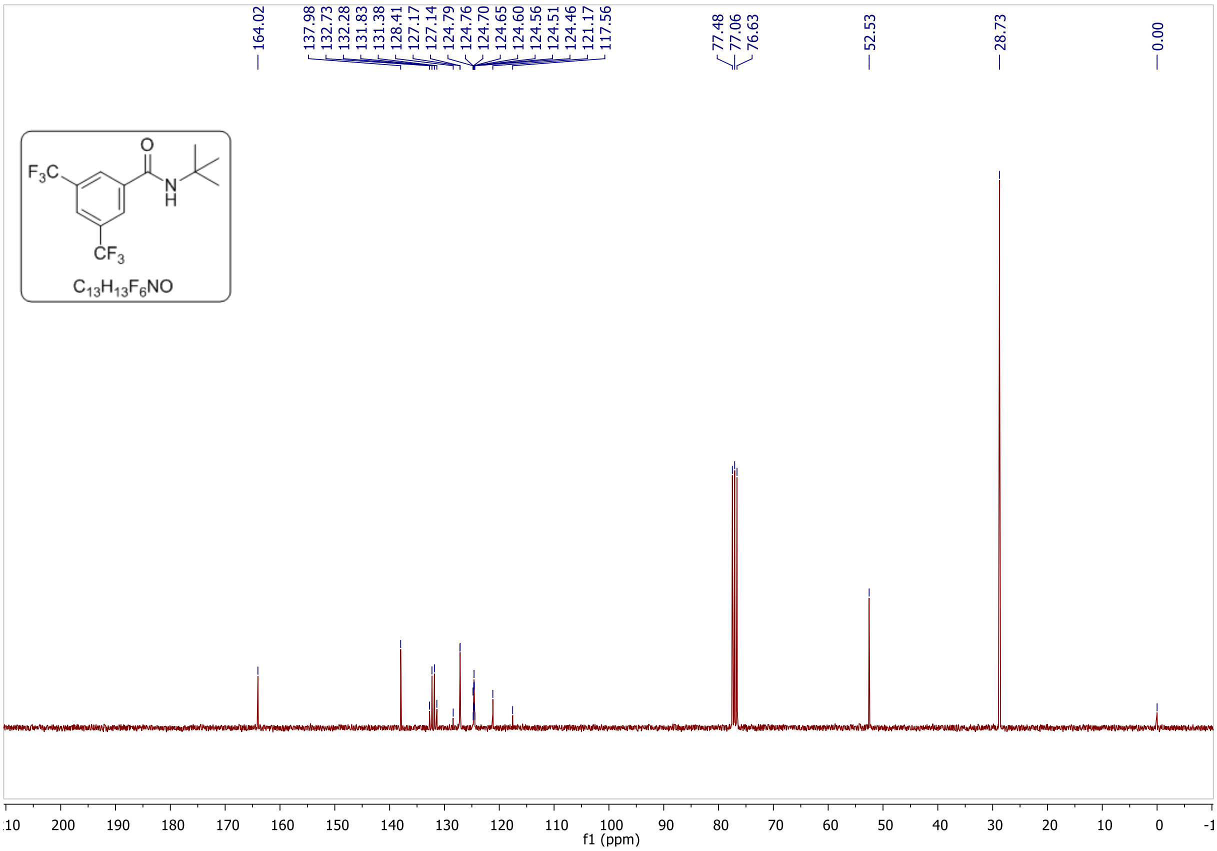

Supplementary Figure 75. ^{13}C-NMR (75 MHz) of **S1-c**.

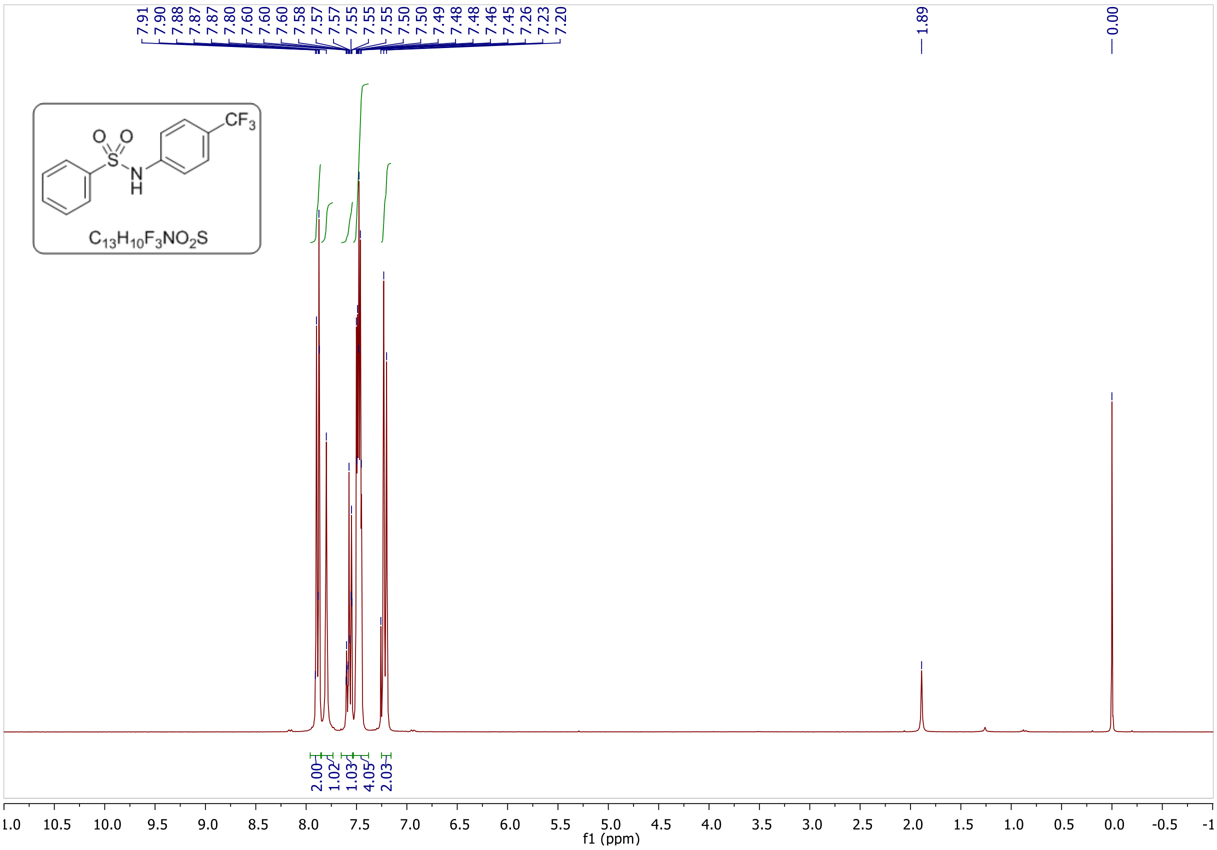

Supplementary Figure 76. ^{1}H-NMR (300 MHz) **S2-a**.

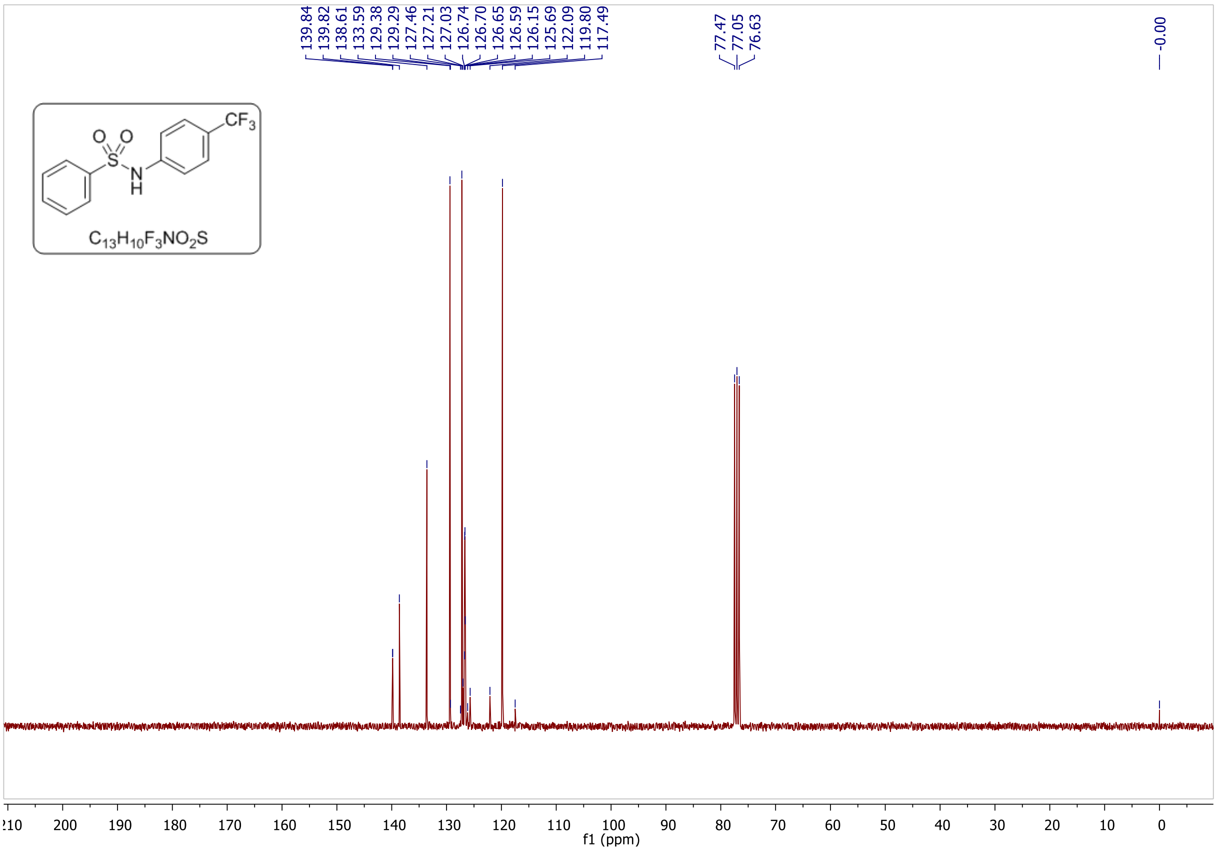

Supplementary Figure 77. ^{13}C-NMR (75 MHz) of **S2-a**.

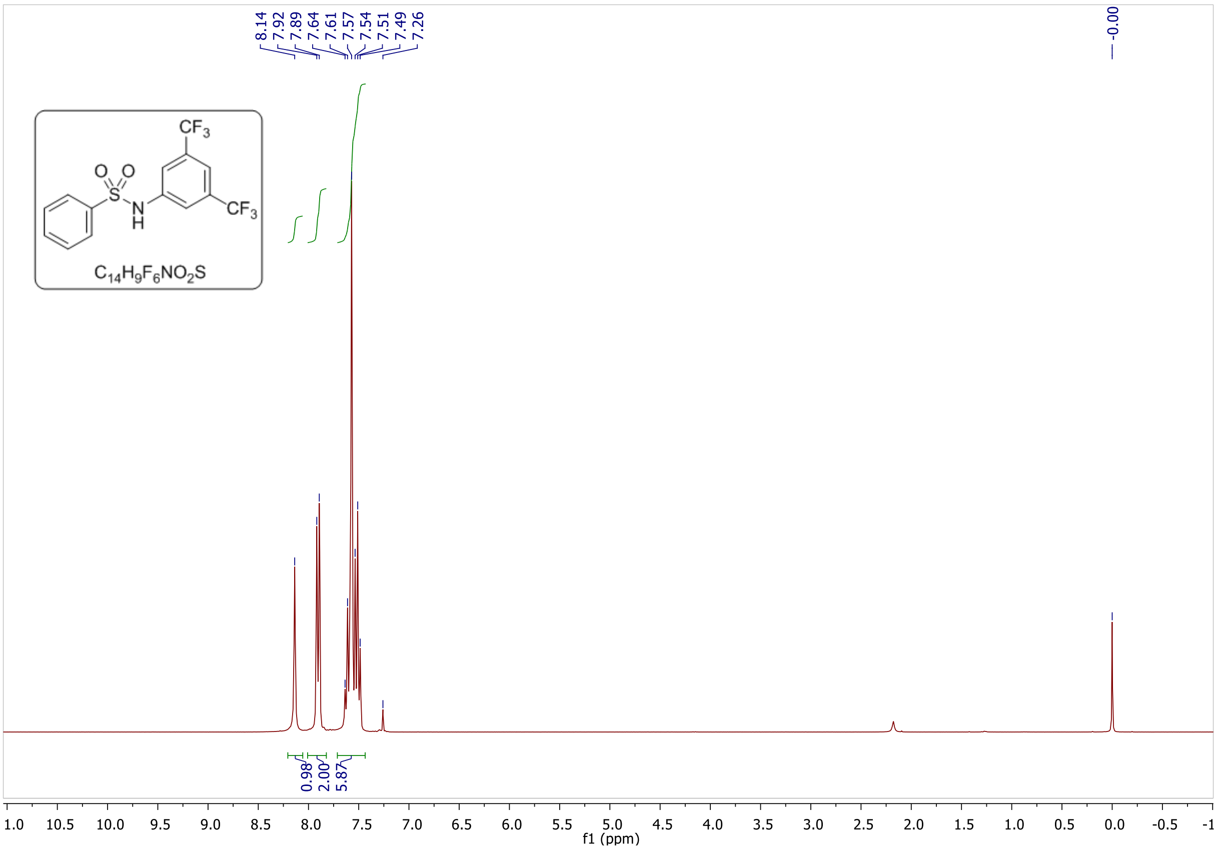

Supplementary Figure 78. ^{1}H-NMR (300 MHz) **S2-b**.

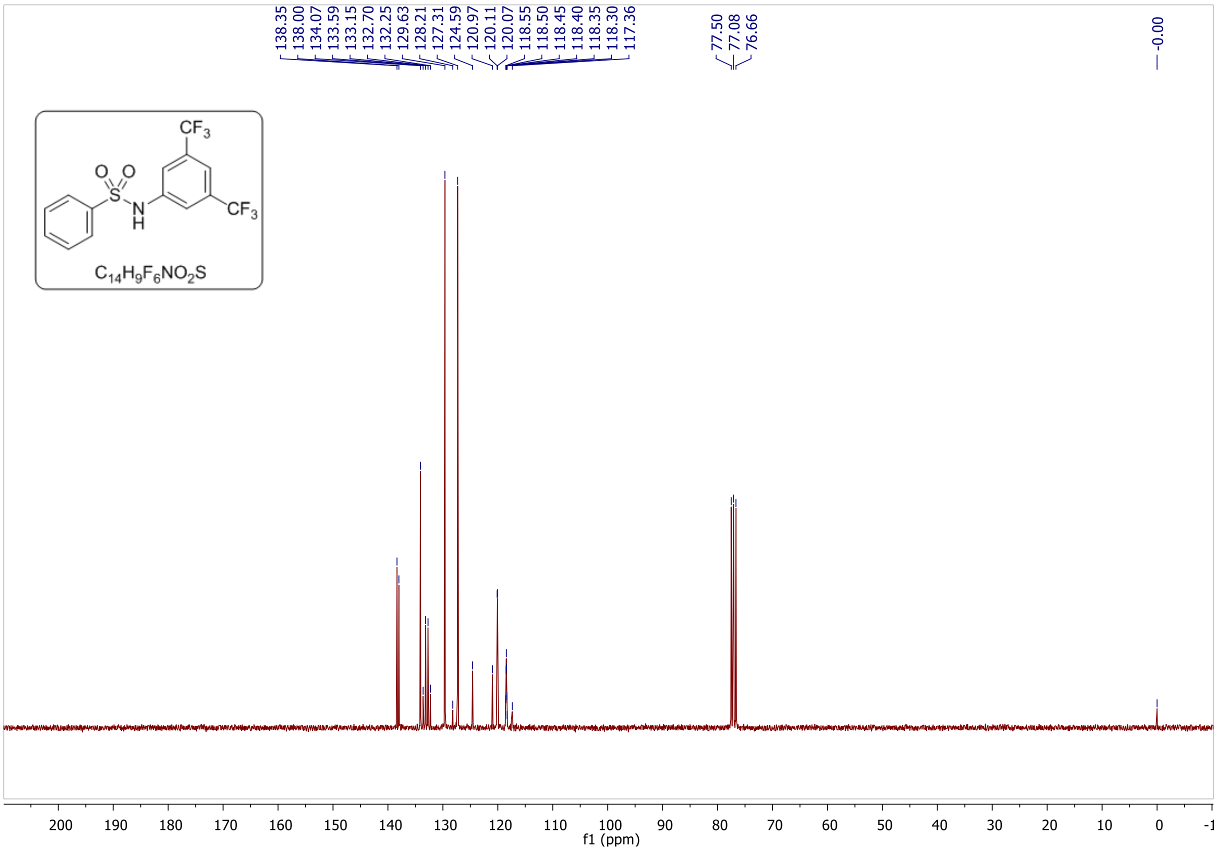

Supplementary Figure 79. ^{13}C-NMR (75 MHz) of **S2-b**.

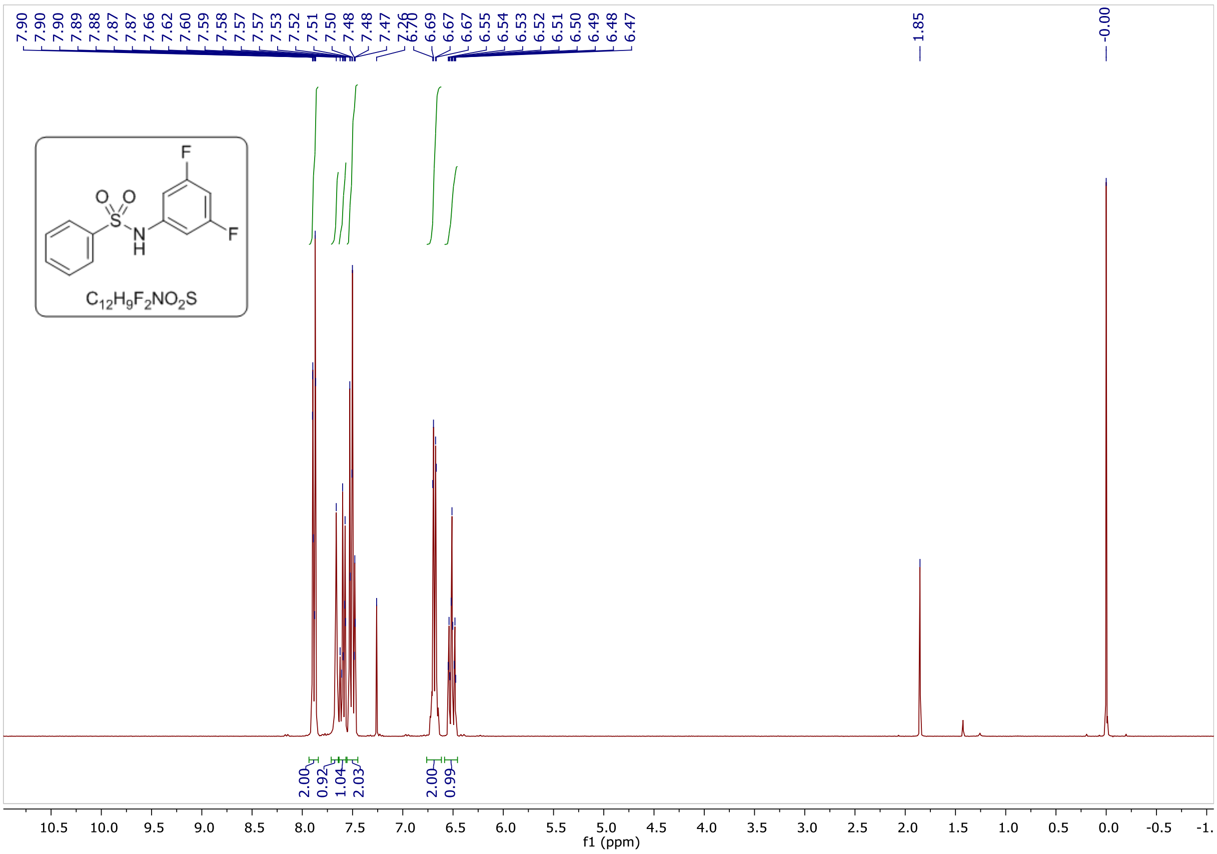

Supplementary Figure 80. ^{1}H-NMR (300 MHz) **S2-c**.

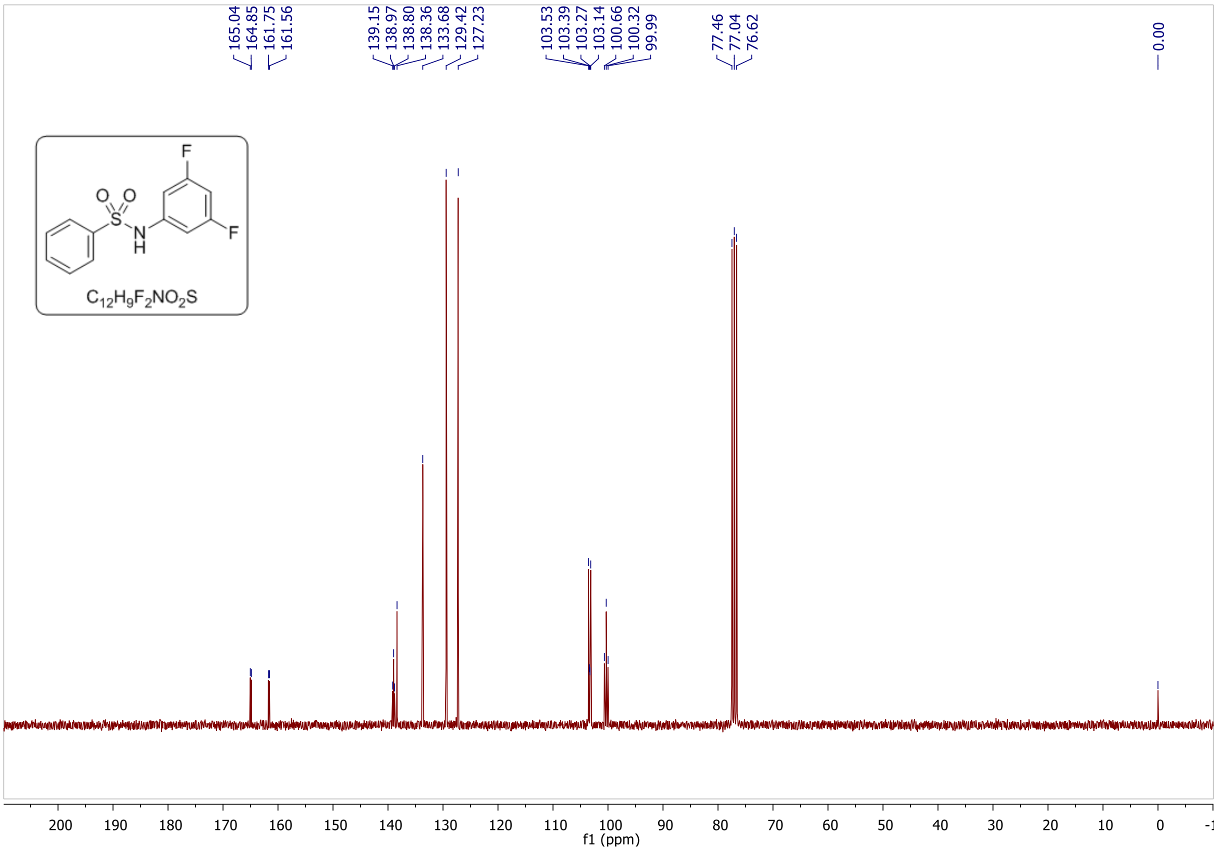

Supplementary Figure 81. ^{13}C-NMR (75 MHz) of **S2-c**.

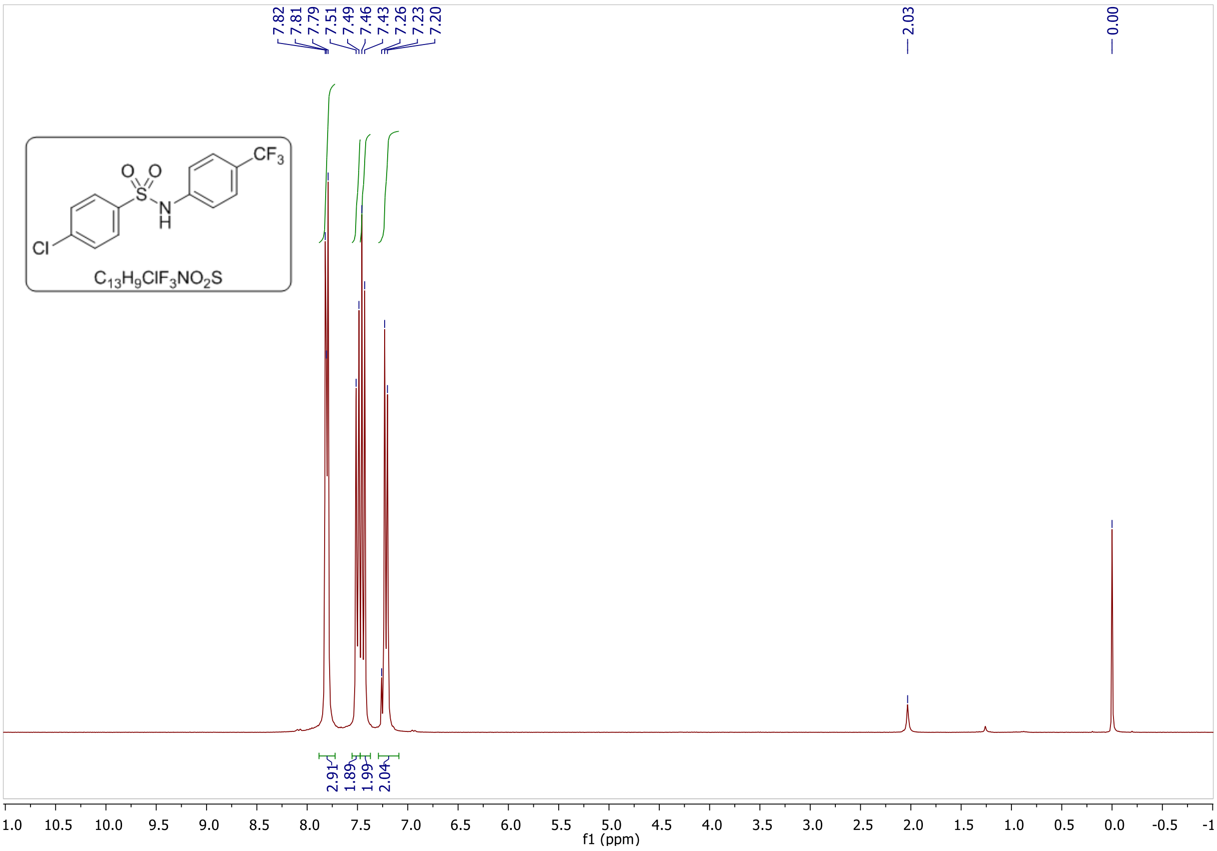

Supplementary Figure 82. ^{1}H-NMR (300 MHz) of **S2-d**.

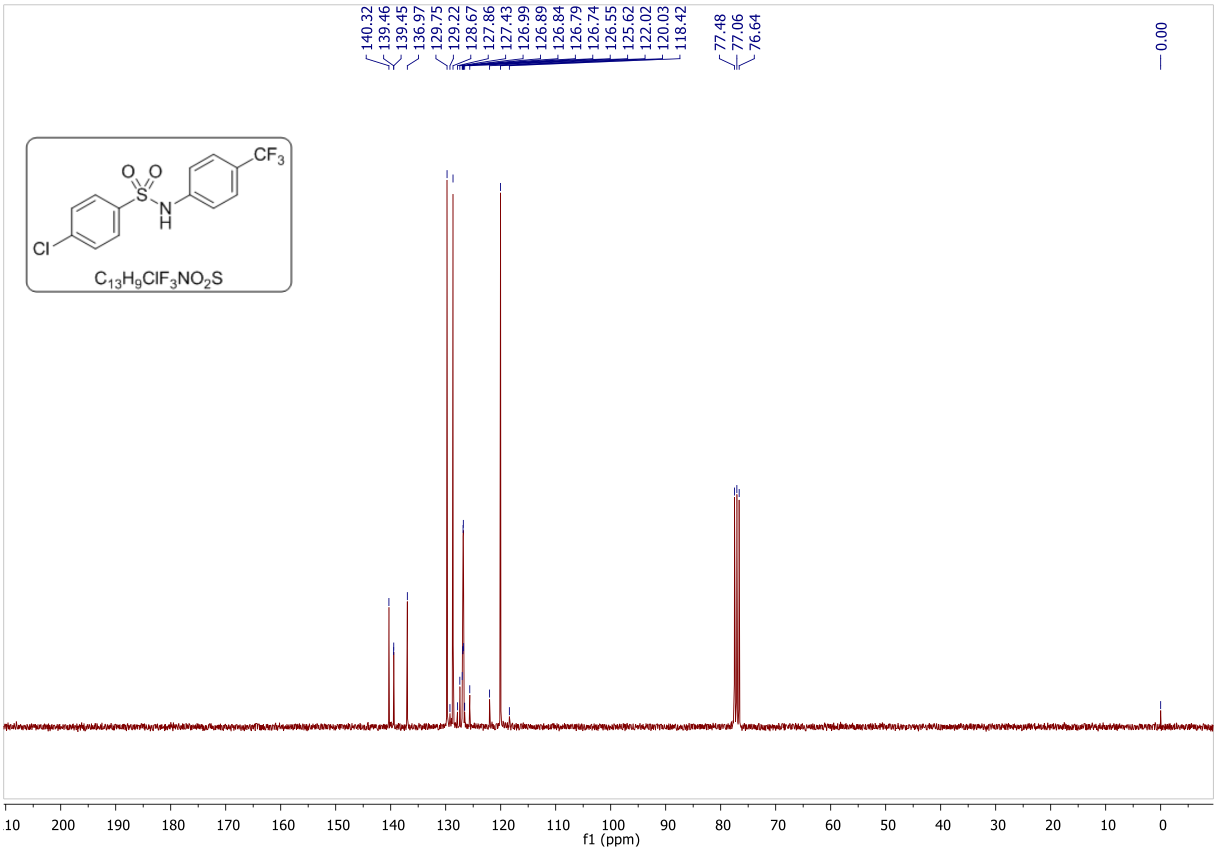

Supplementary Figure 83. ^{13}C-NMR (75 MHz) of **S2-d**.

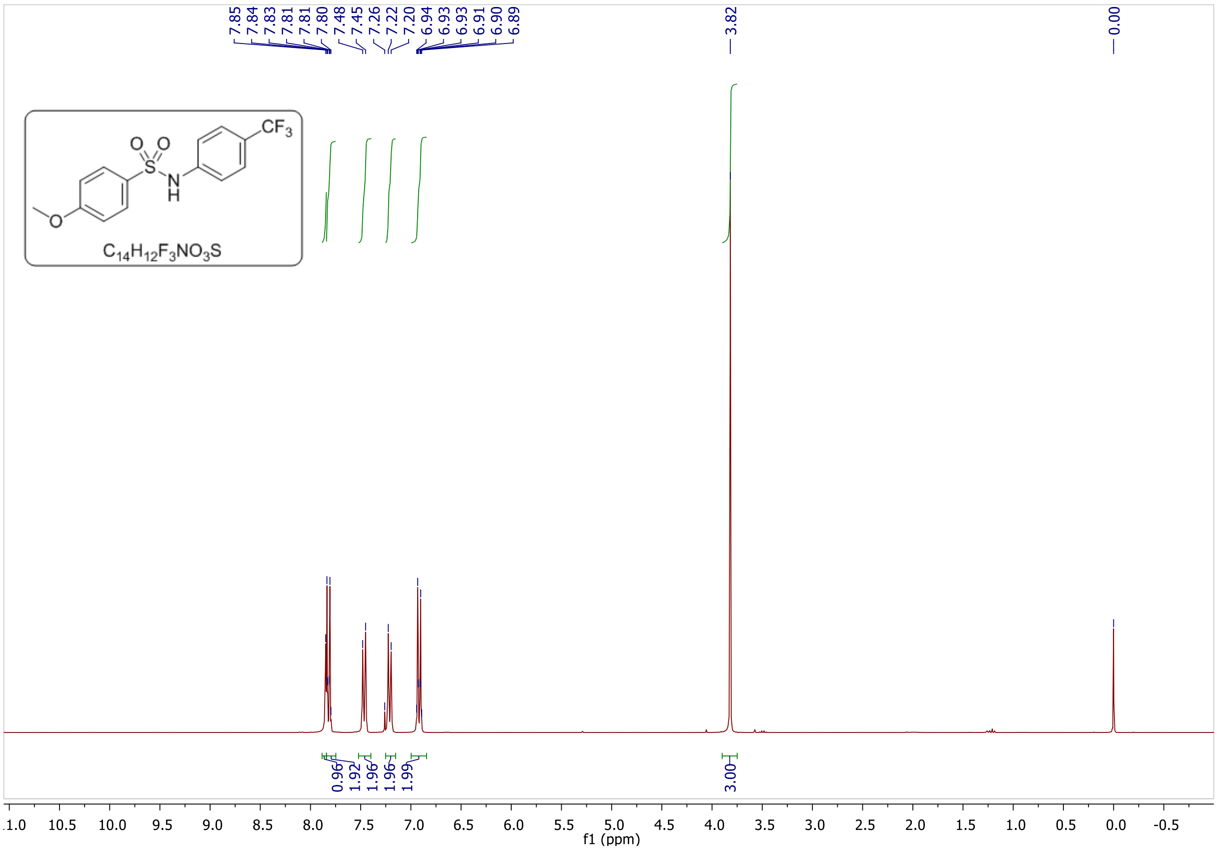

Supplementary Figure 84. ^{1}H-NMR (300 MHz) of **S2-e**.

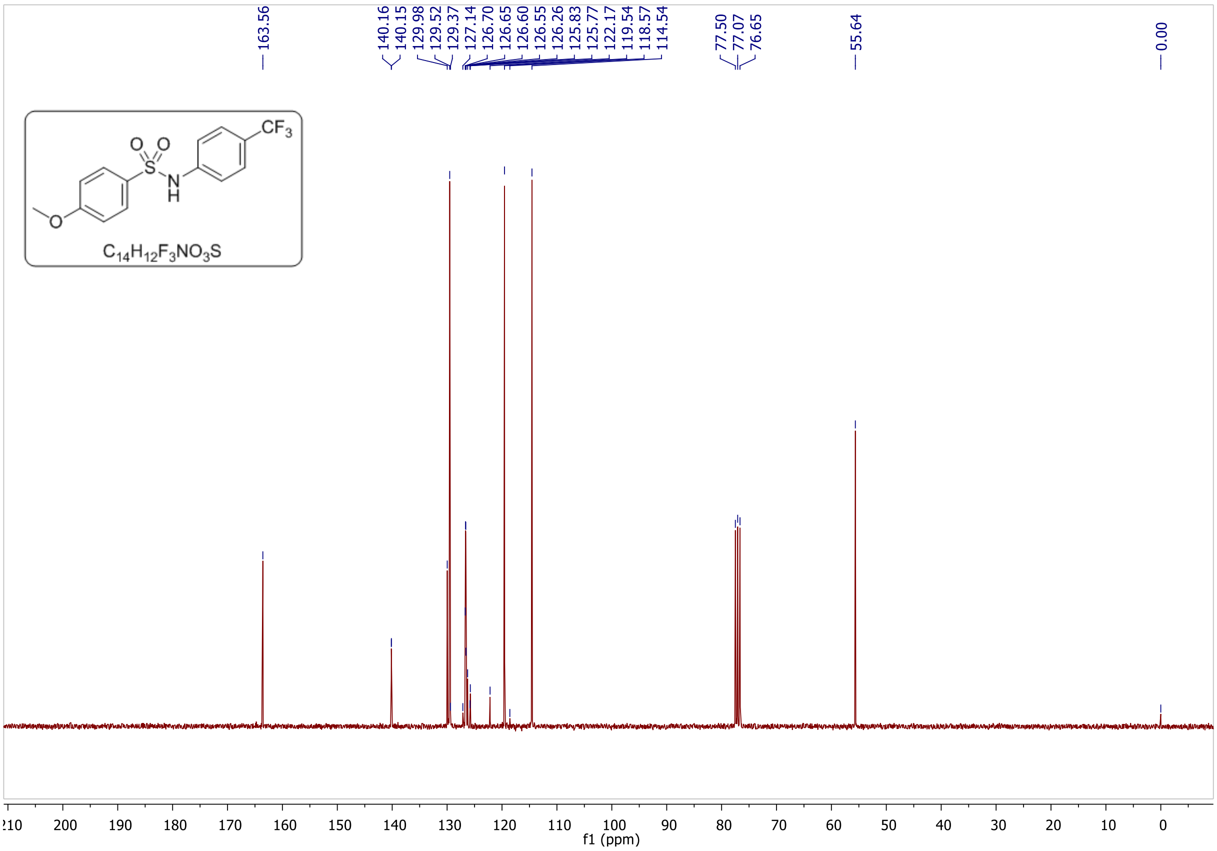

Supplementary Figure 85. ^{13}C-NMR (75 MHz) of **S2-e**.

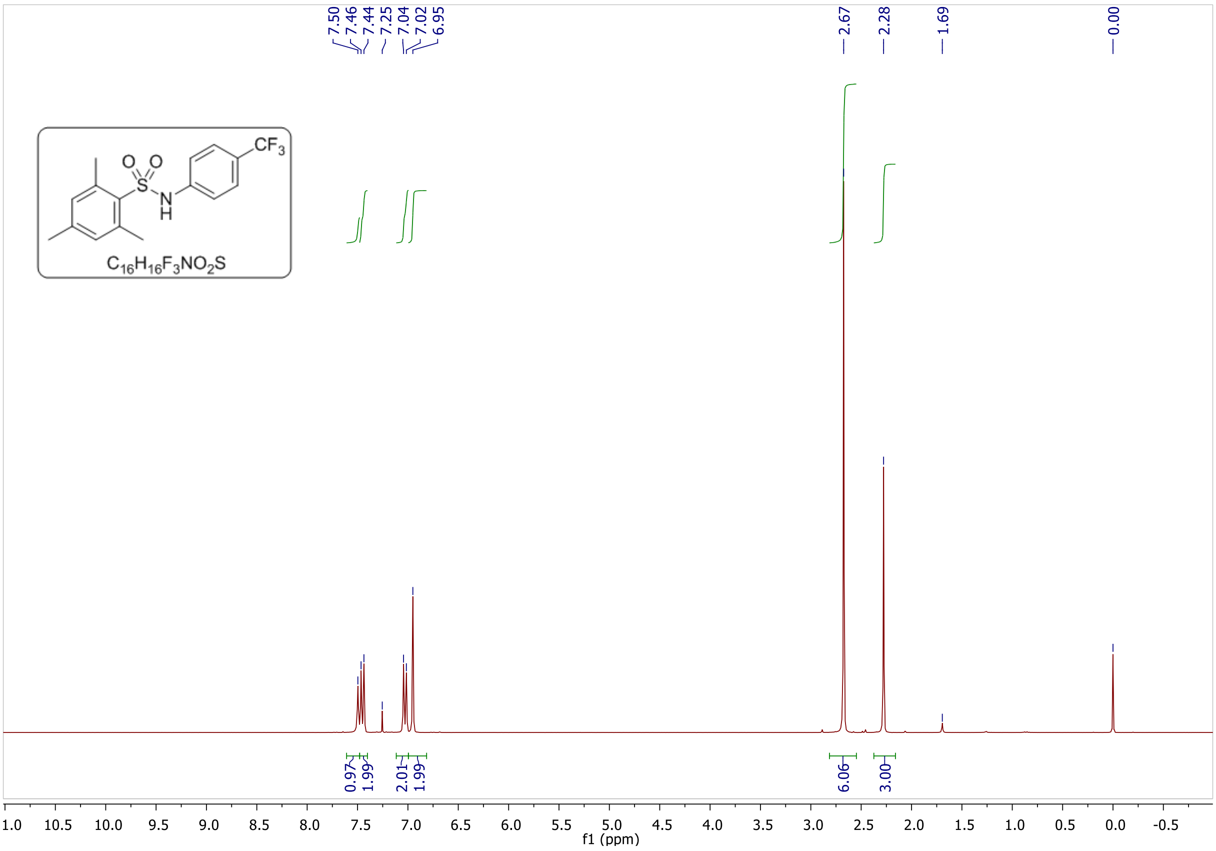

Supplementary Figure 86. ^{1}H-NMR (300 MHz) of **S2-f**.

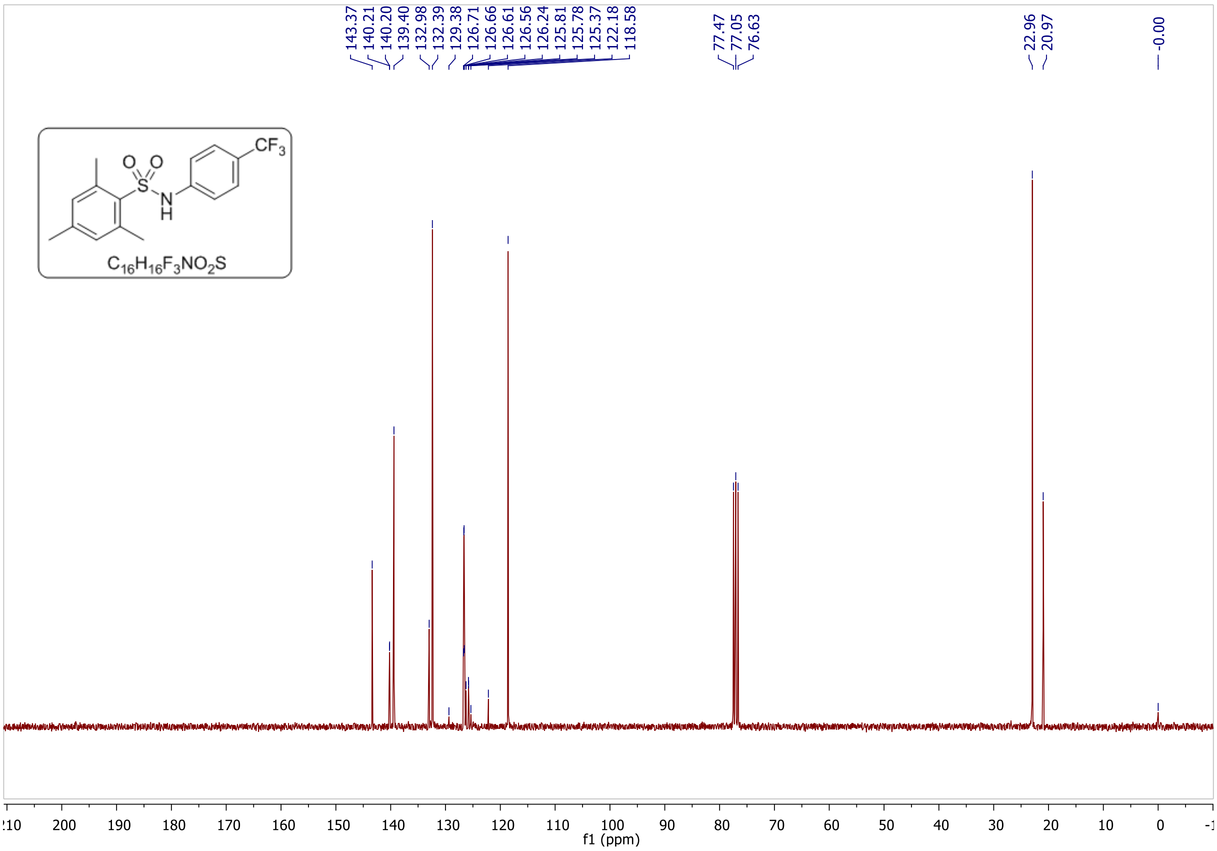

Supplementary Figure 87. ^{13}C-NMR (75 MHz) of **S2-f**.

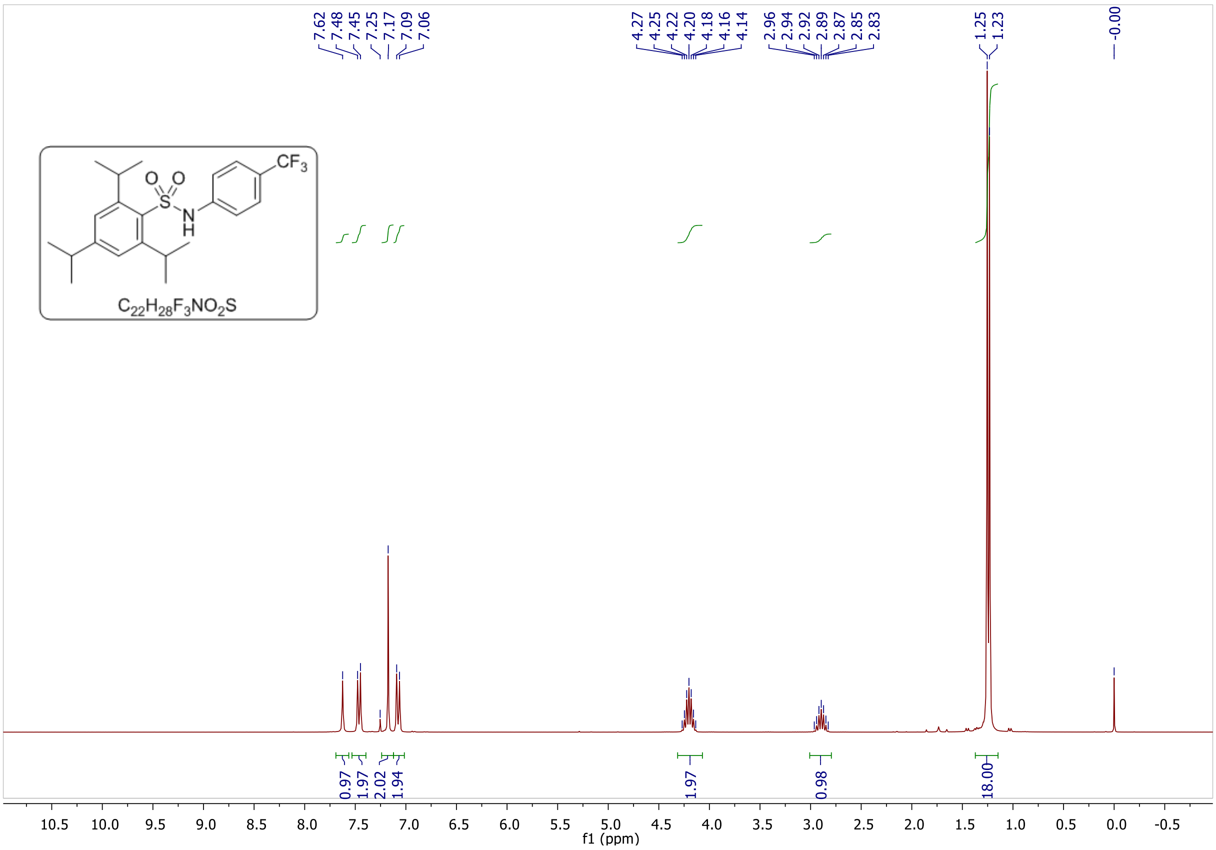

Supplementary Figure 88. ^{1}H-NMR (300 MHz) of **S2-g**.

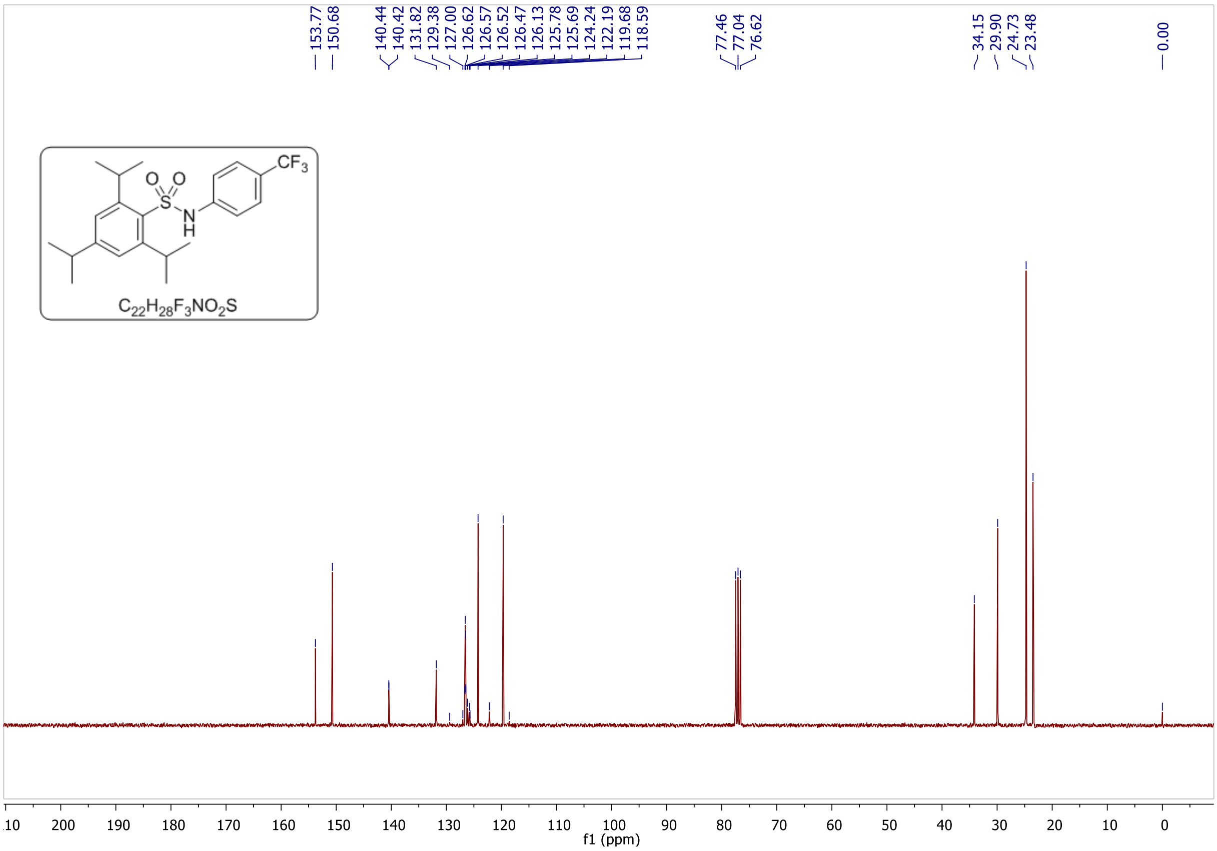

Supplementary Figure 89. ^{13}C-NMR (75 MHz) of **S2-g**.

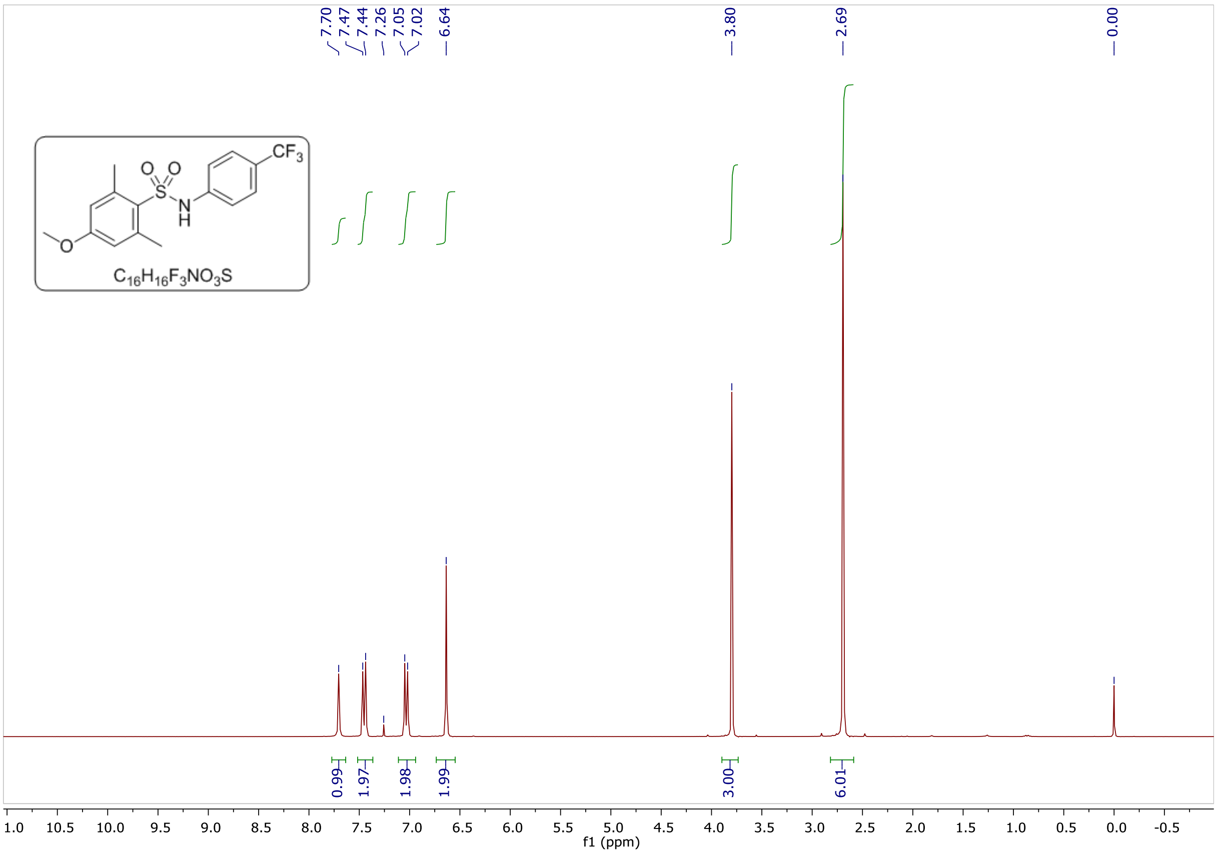

Supplementary Figure 90. ^{1}H-NMR (300 MHz) of **S2-h**.

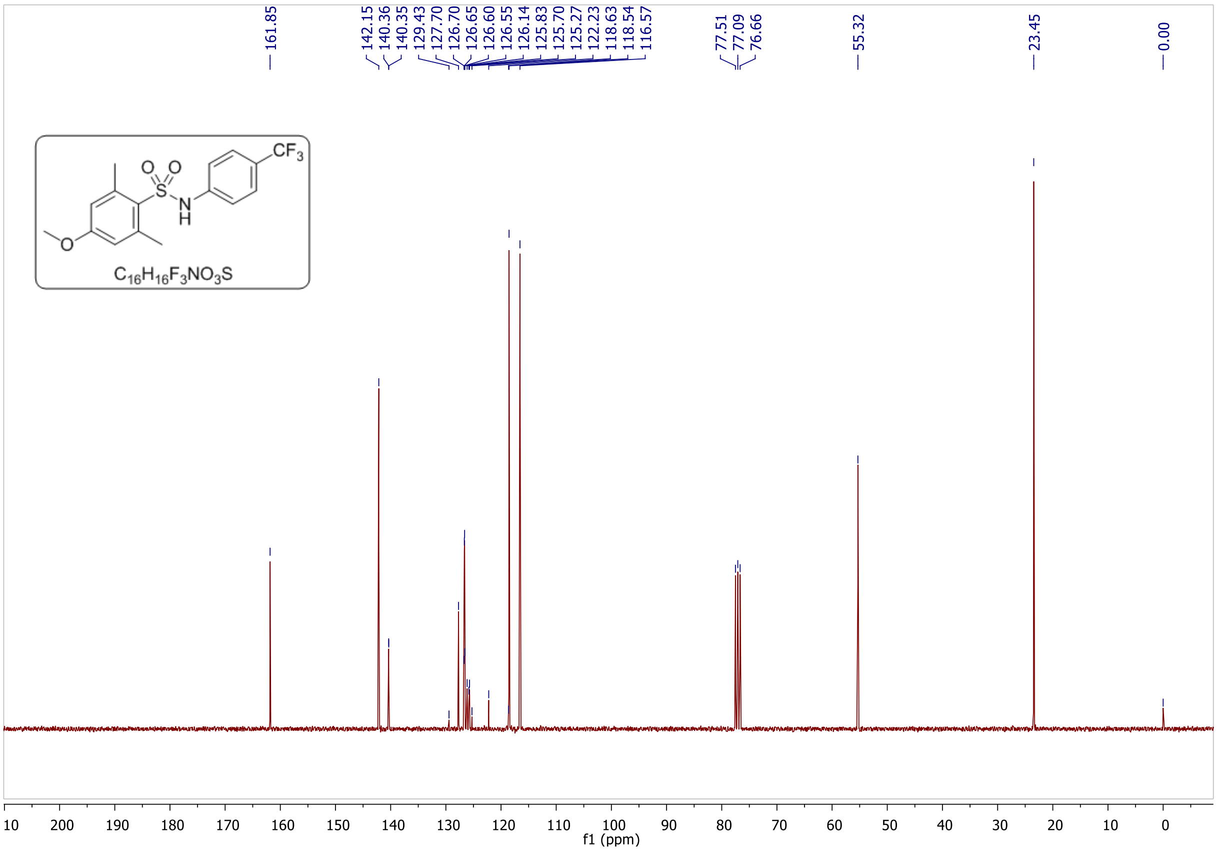

Supplementary Figure 91. ^{13}C-NMR (75 MHz) of **S2-h**.

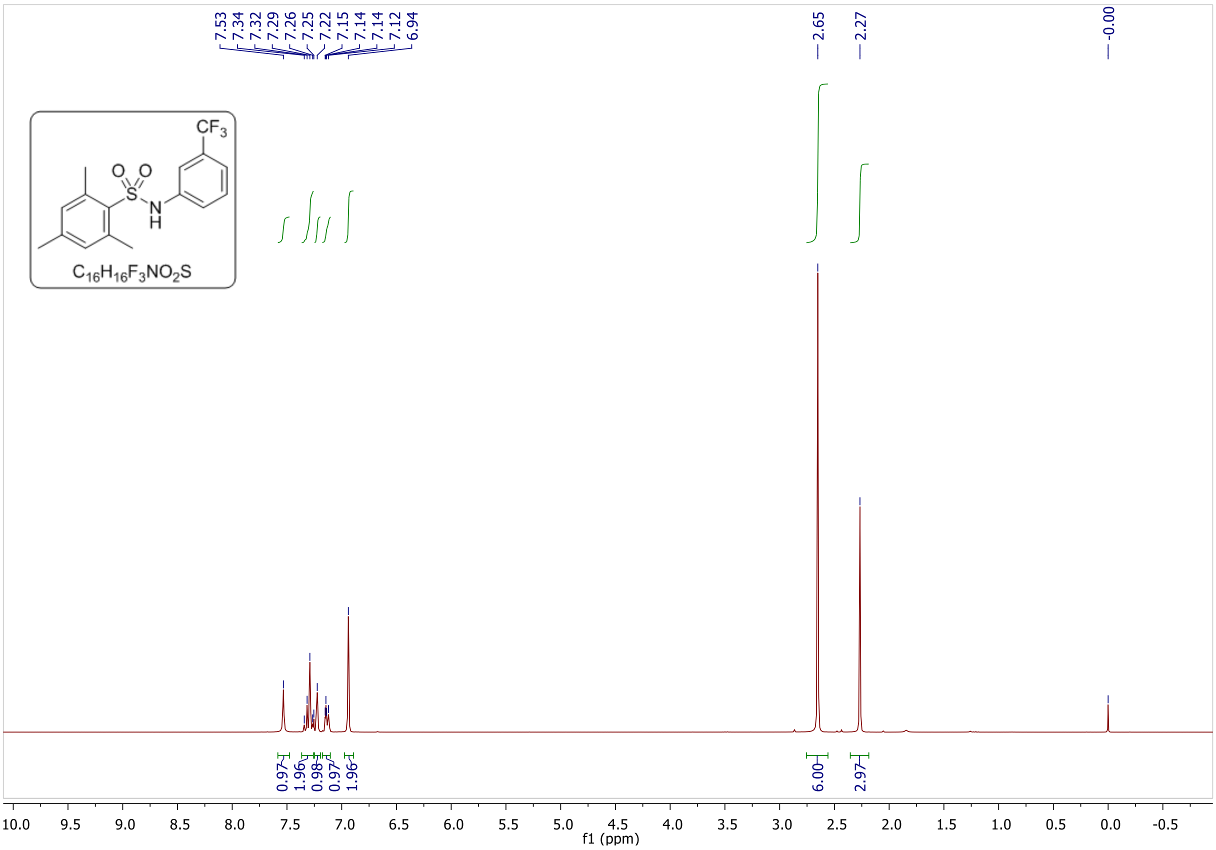

Supplementary Figure 92. ^{1}H-NMR (300 MHz) of **S2-i**.

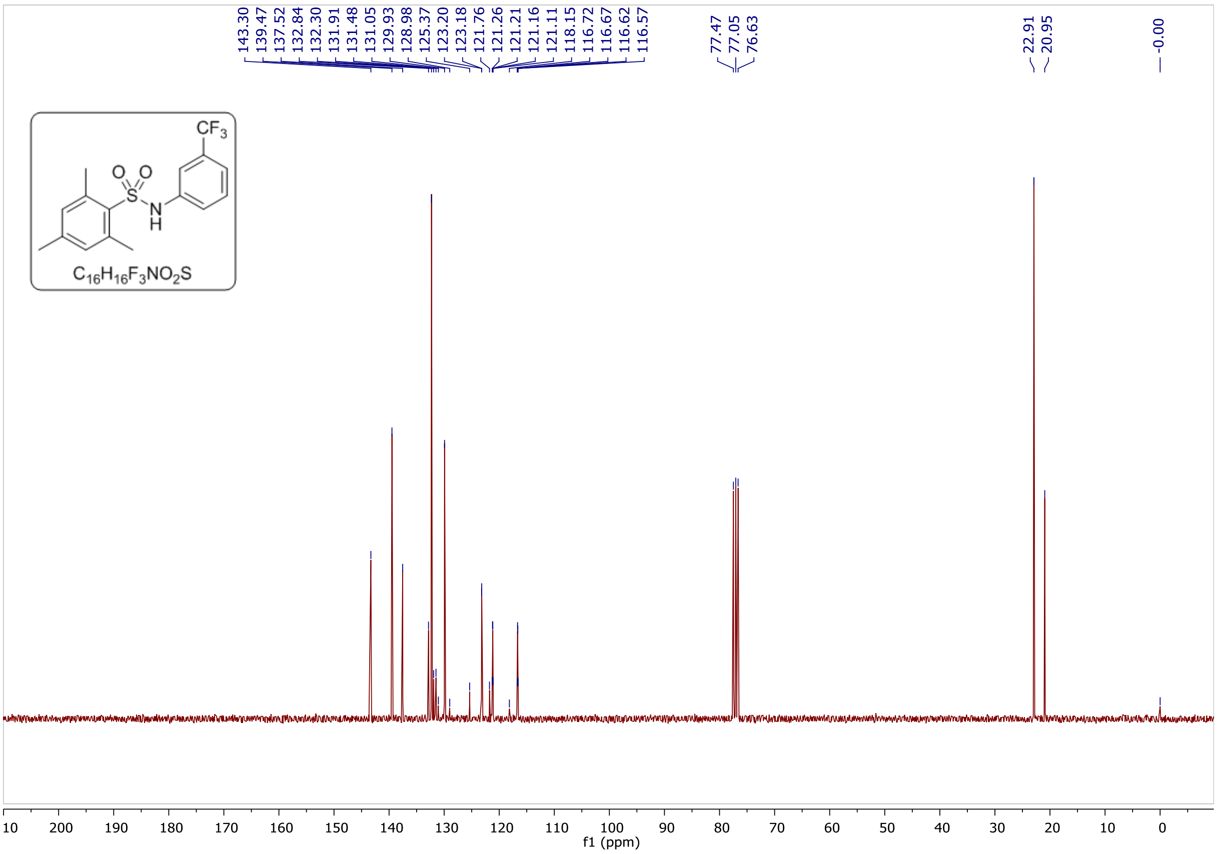

Supplementary Figure 93. ^{13}C-NMR (75 MHz) of **S2-i**.

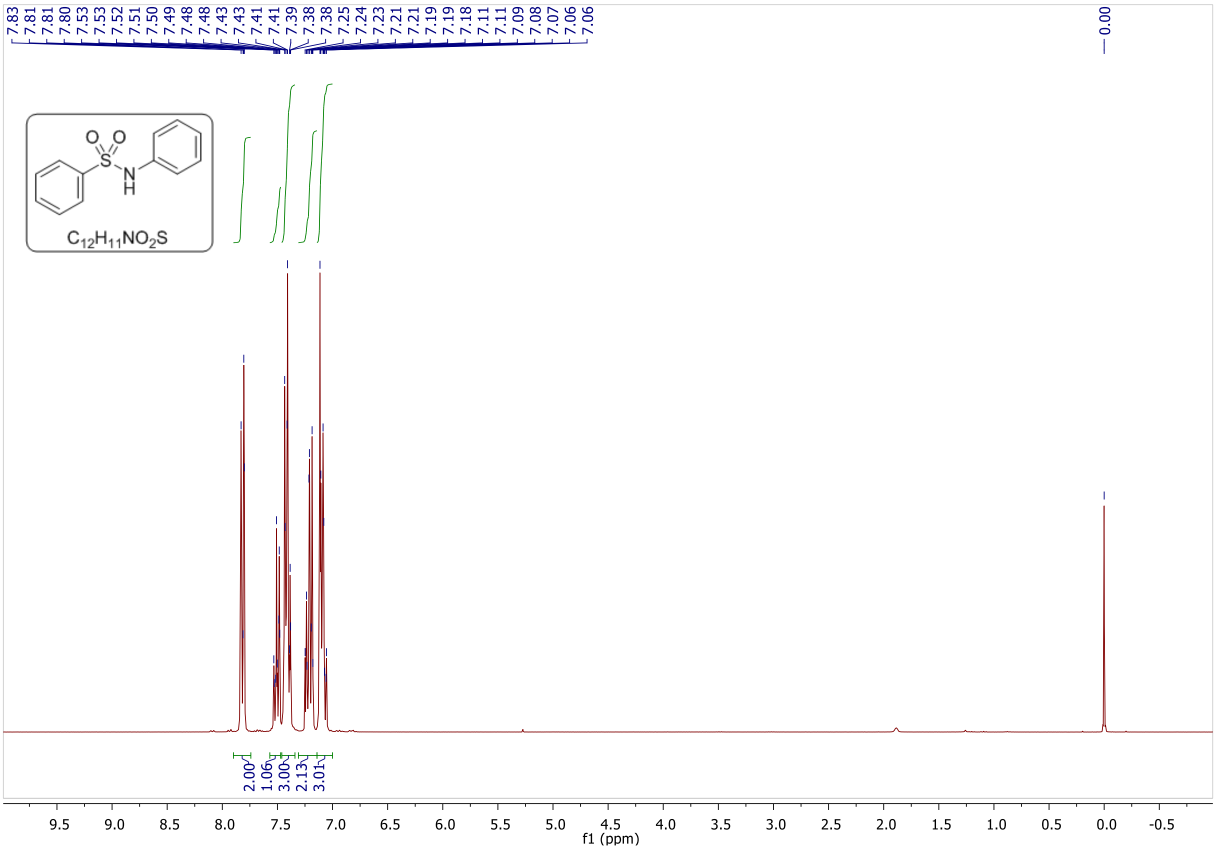

Supplementary Figure 94. ^{1}H-NMR (300 MHz) of **S2-j**.

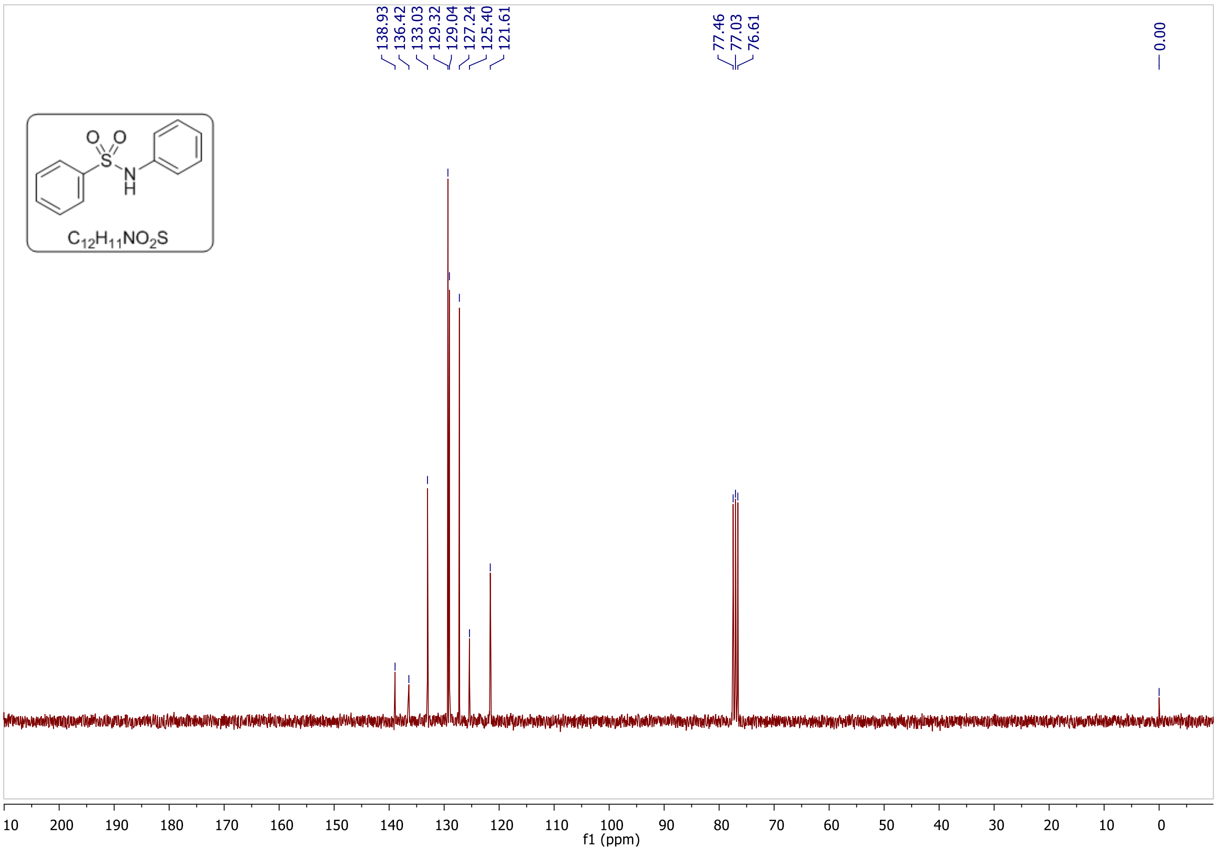

Supplementary Figure 95. ^{13}C-NMR (75 MHz) of **S2-j**.

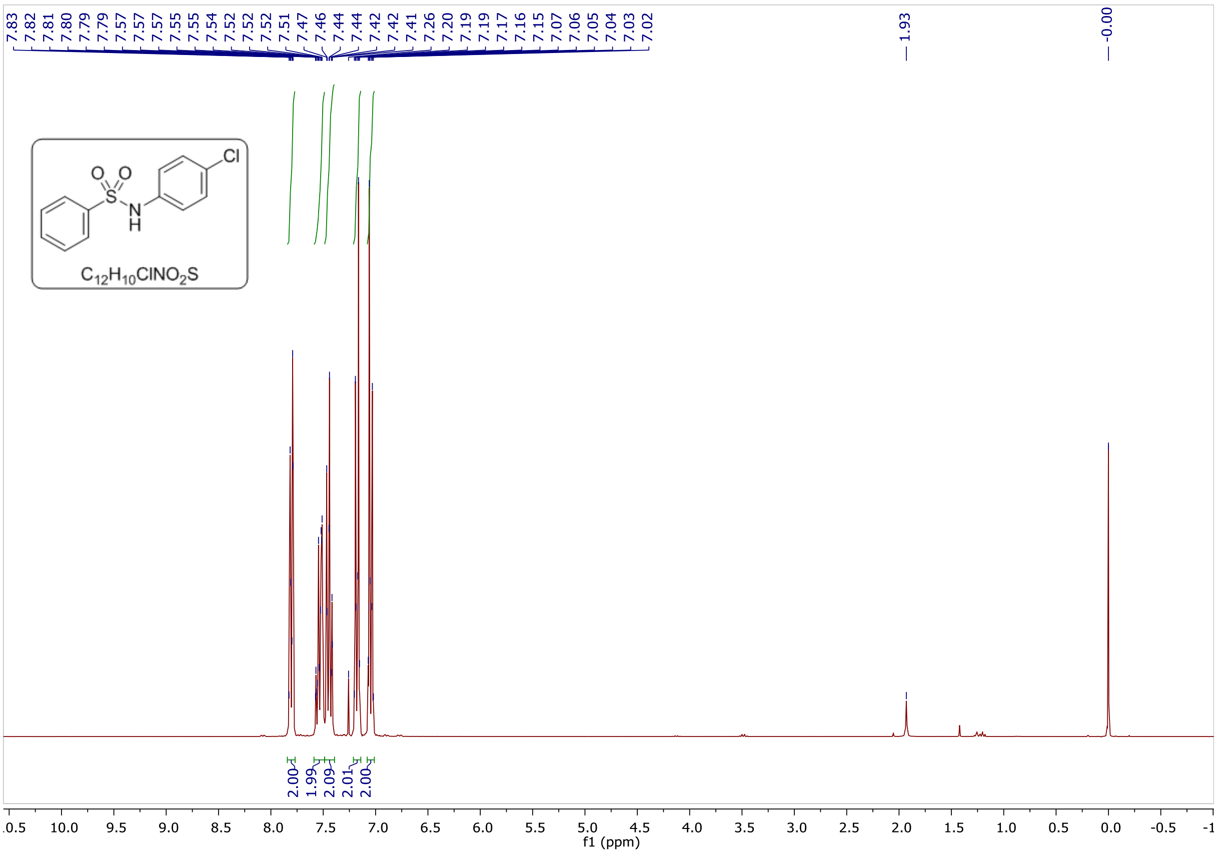

Supplementary Figure 96. ^{1}H-NMR (300 MHz) of **S2-k**.

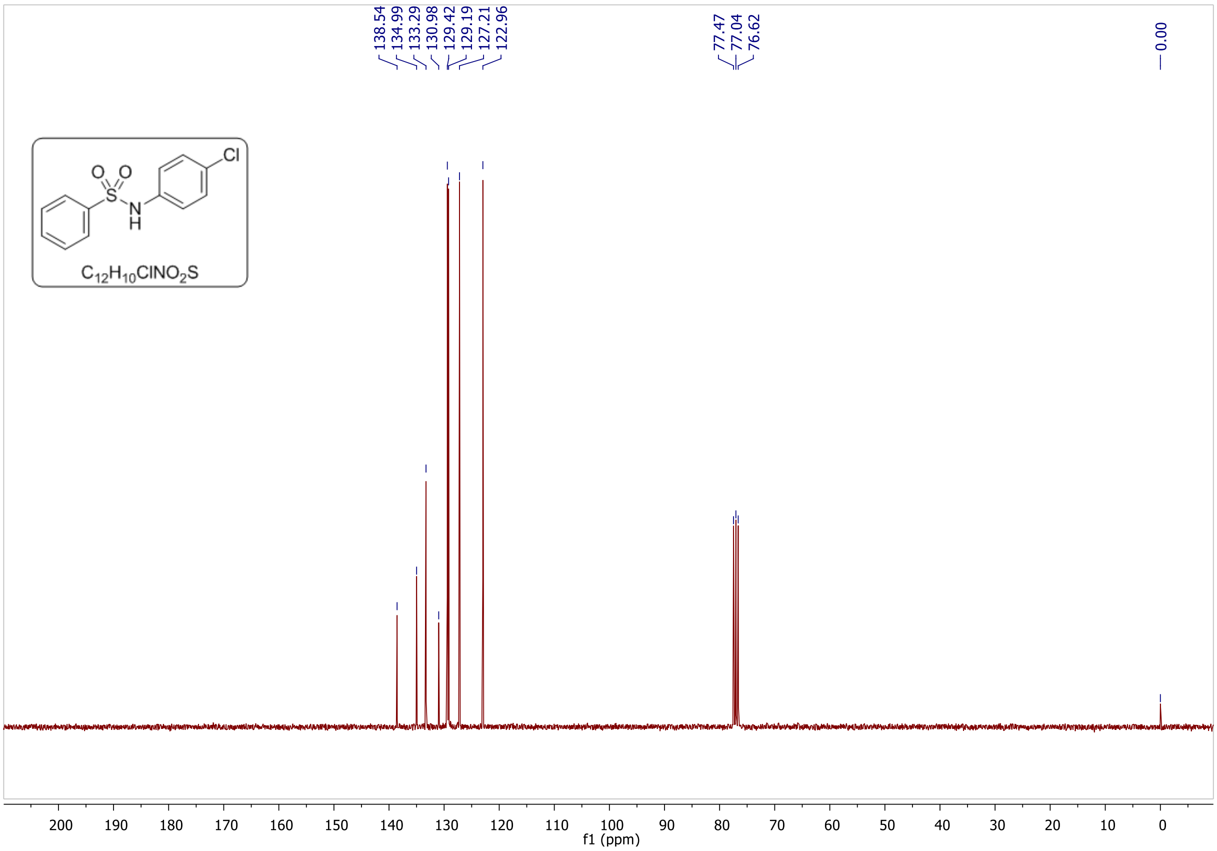

Supplementary Figure 97. ^{13}C-NMR (75 MHz) of **S2-k**.

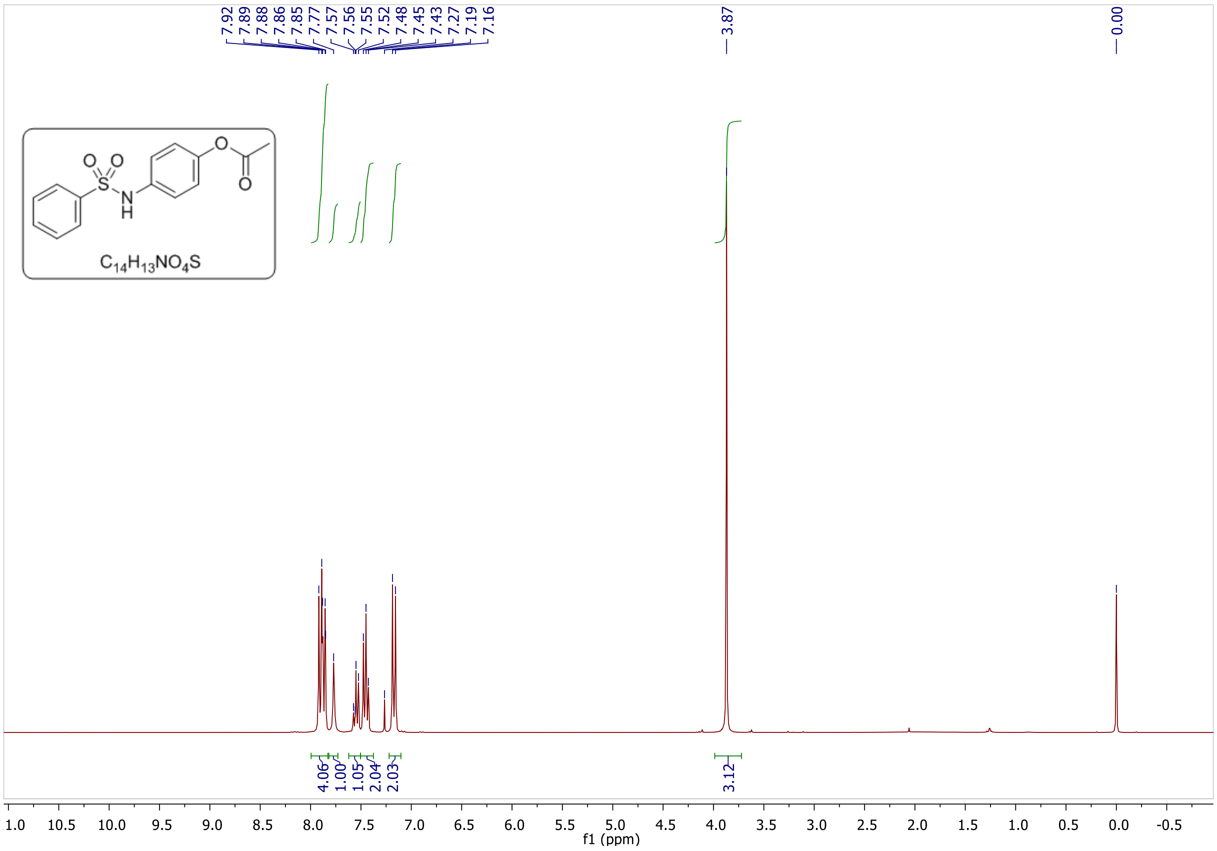

Supplementary Figure 98. ^{1}H-NMR (300 MHz) of **S2-l**.

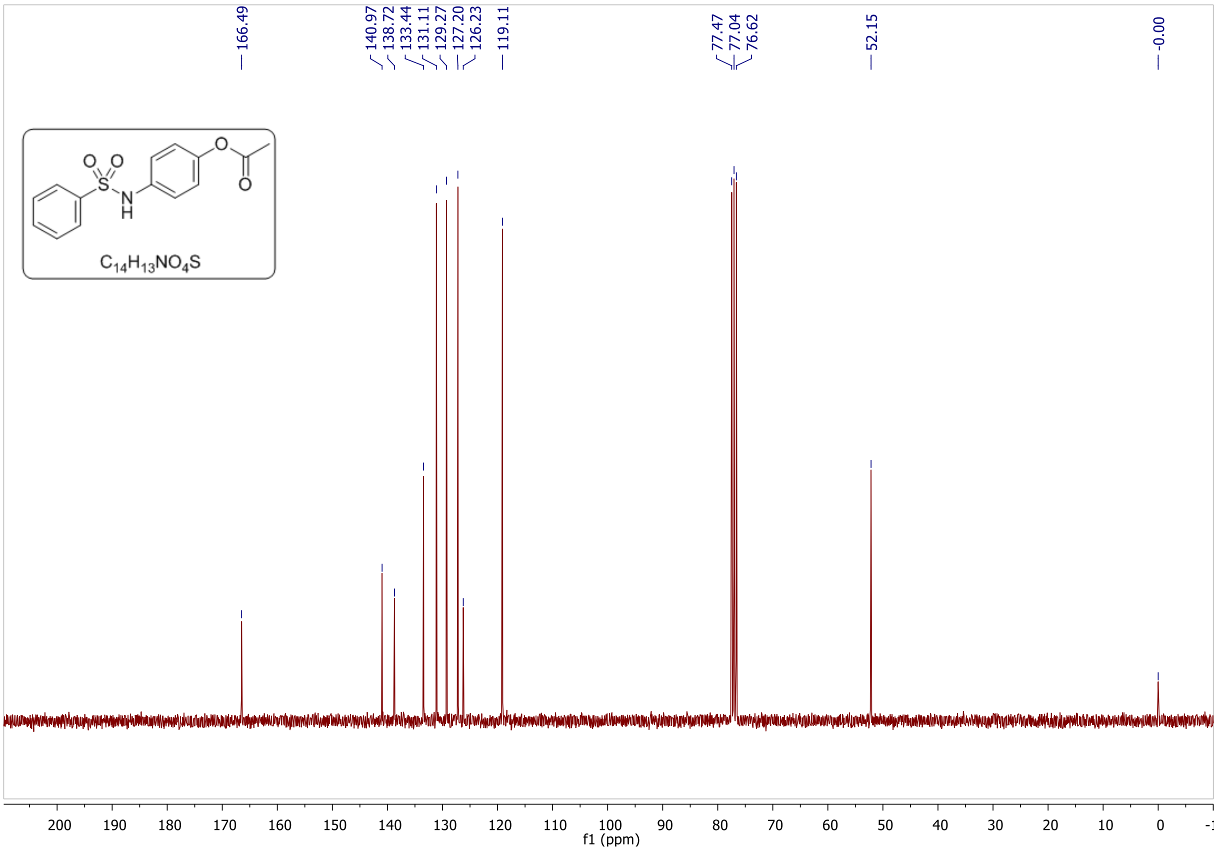

Supplementary Figure 99. ^{13}C-NMR (75 MHz) of **S2-l**.

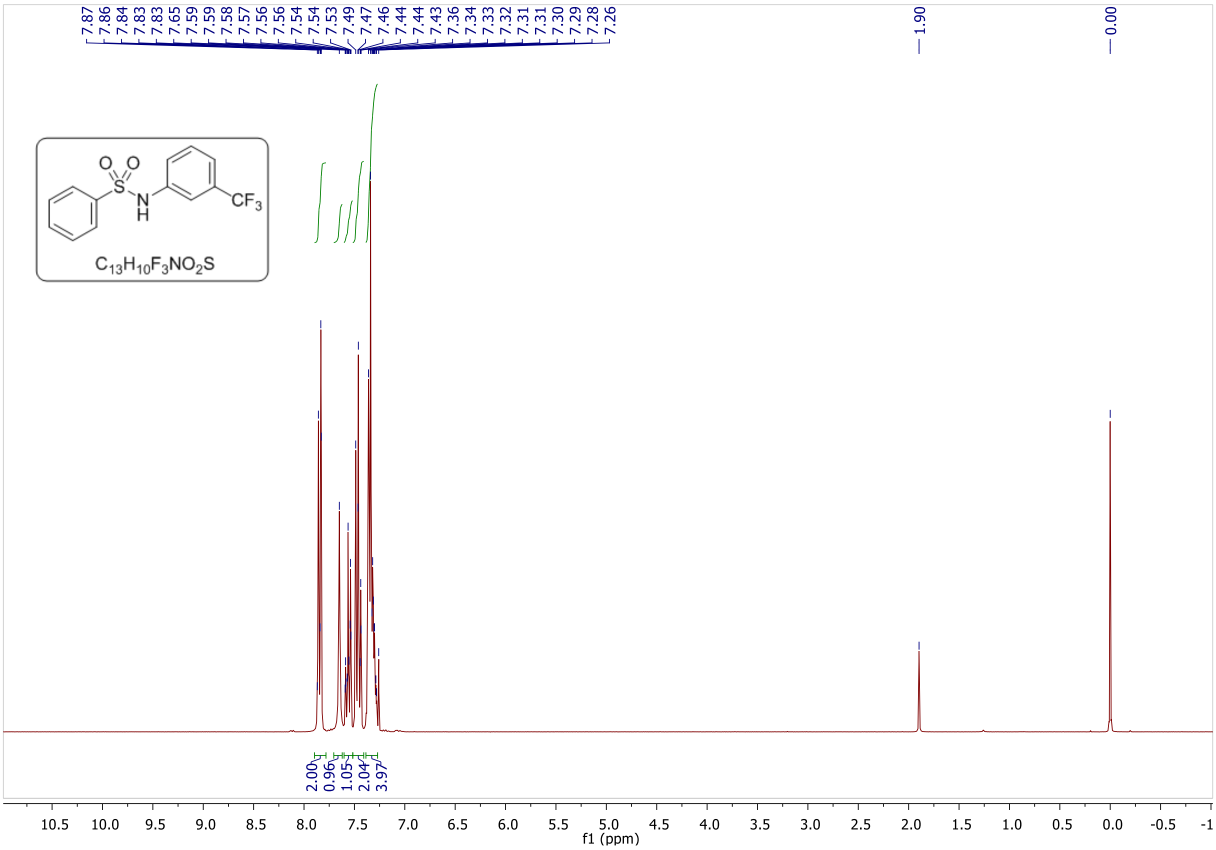

Supplementary Figure 100. ^{1}H-NMR (300 MHz) of **S2-m**.

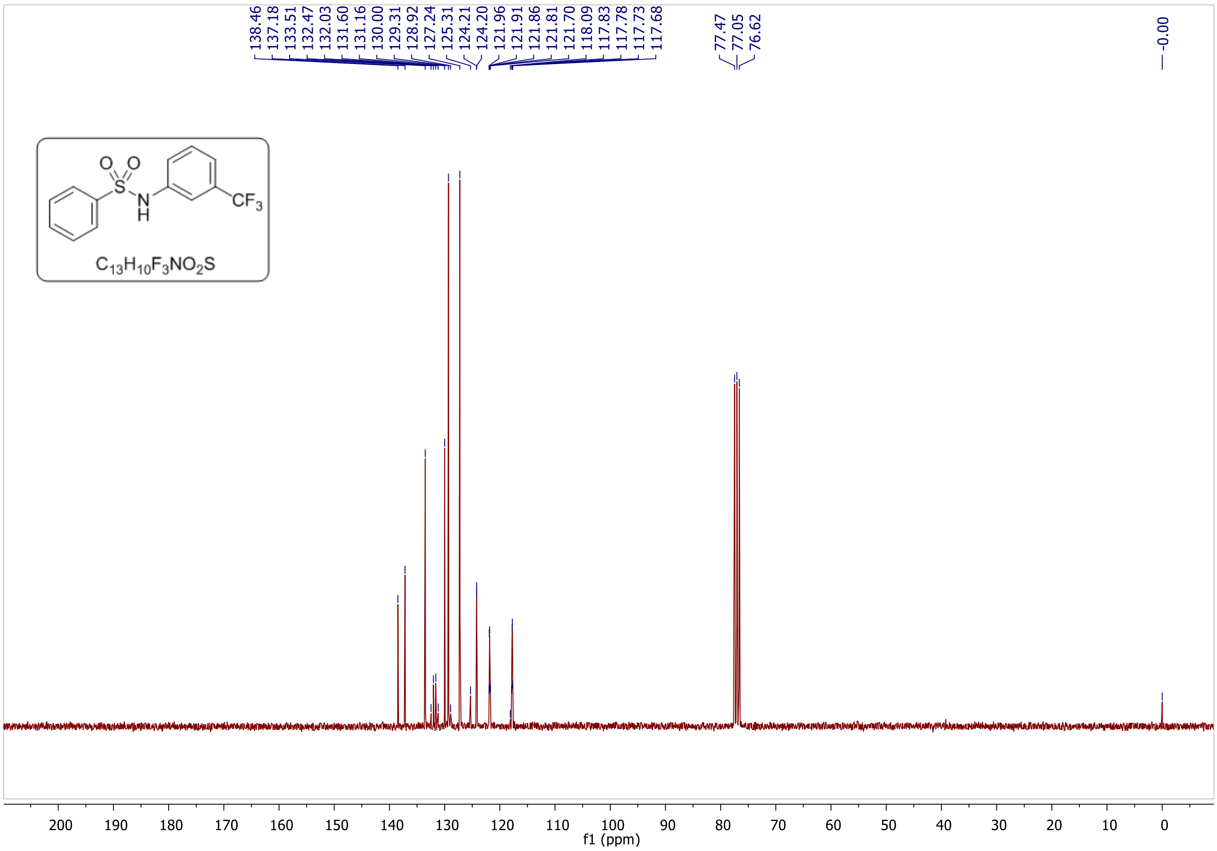

Supplementary Figure 101. ^{13}C-NMR (75 MHz) of **S2-m**.

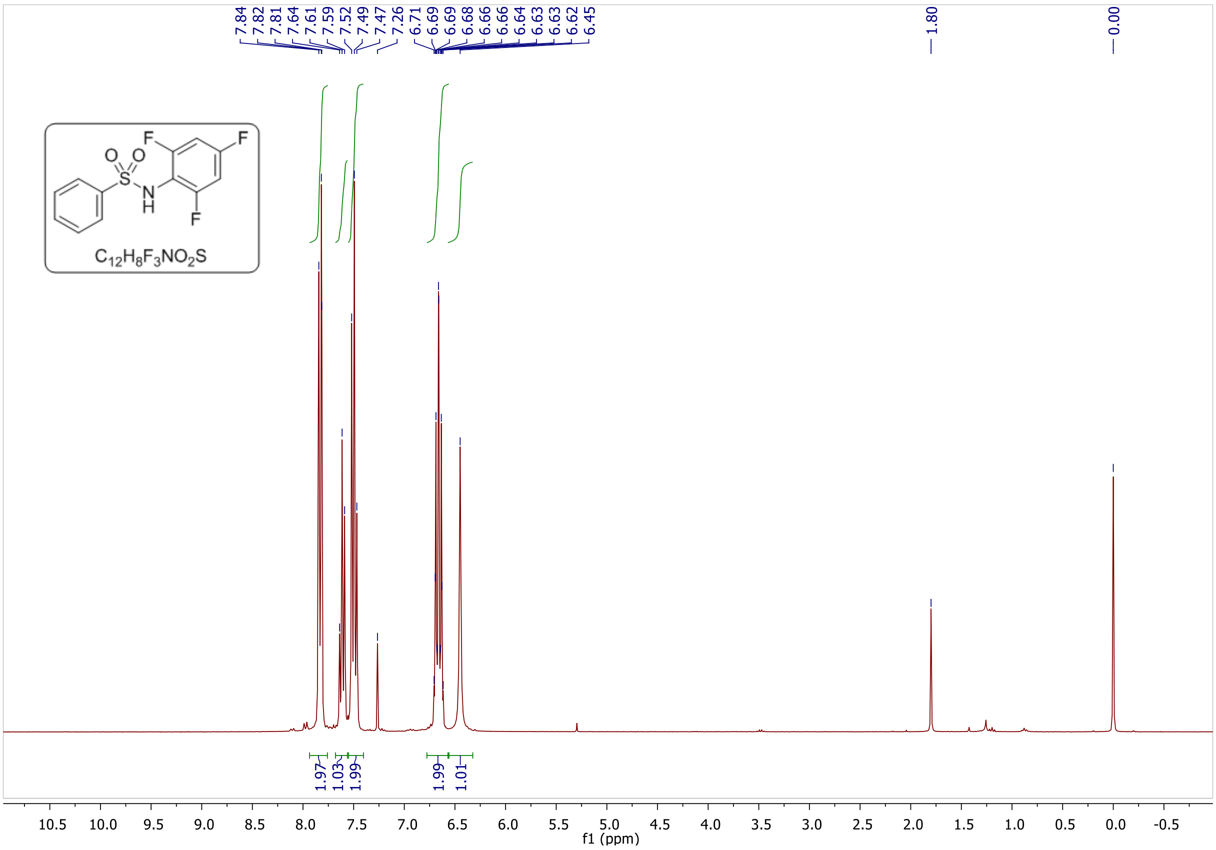

Supplementary Figure 102. ^{1}H-NMR (300 MHz) of **S2-n**.

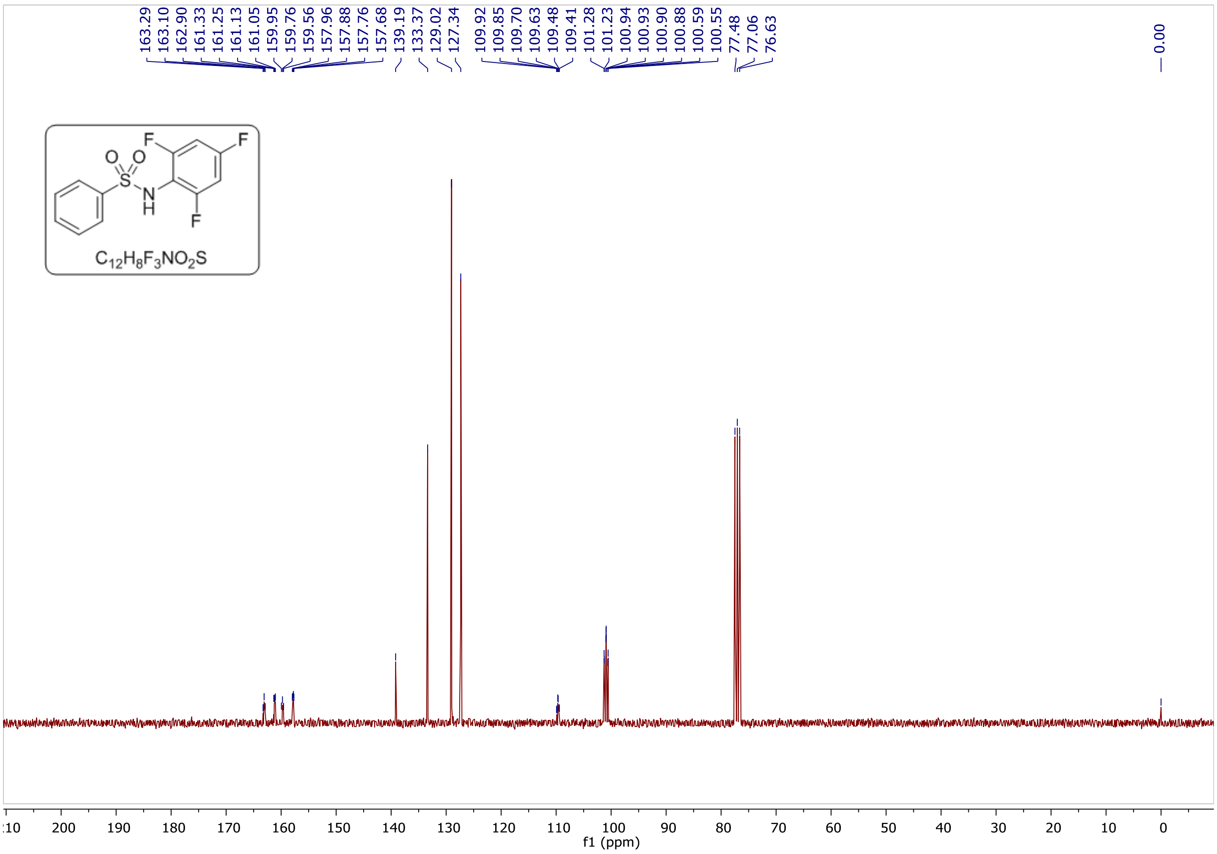

Supplementary Figure 103. ^{13}C-NMR (75 MHz) of **S2-n**.

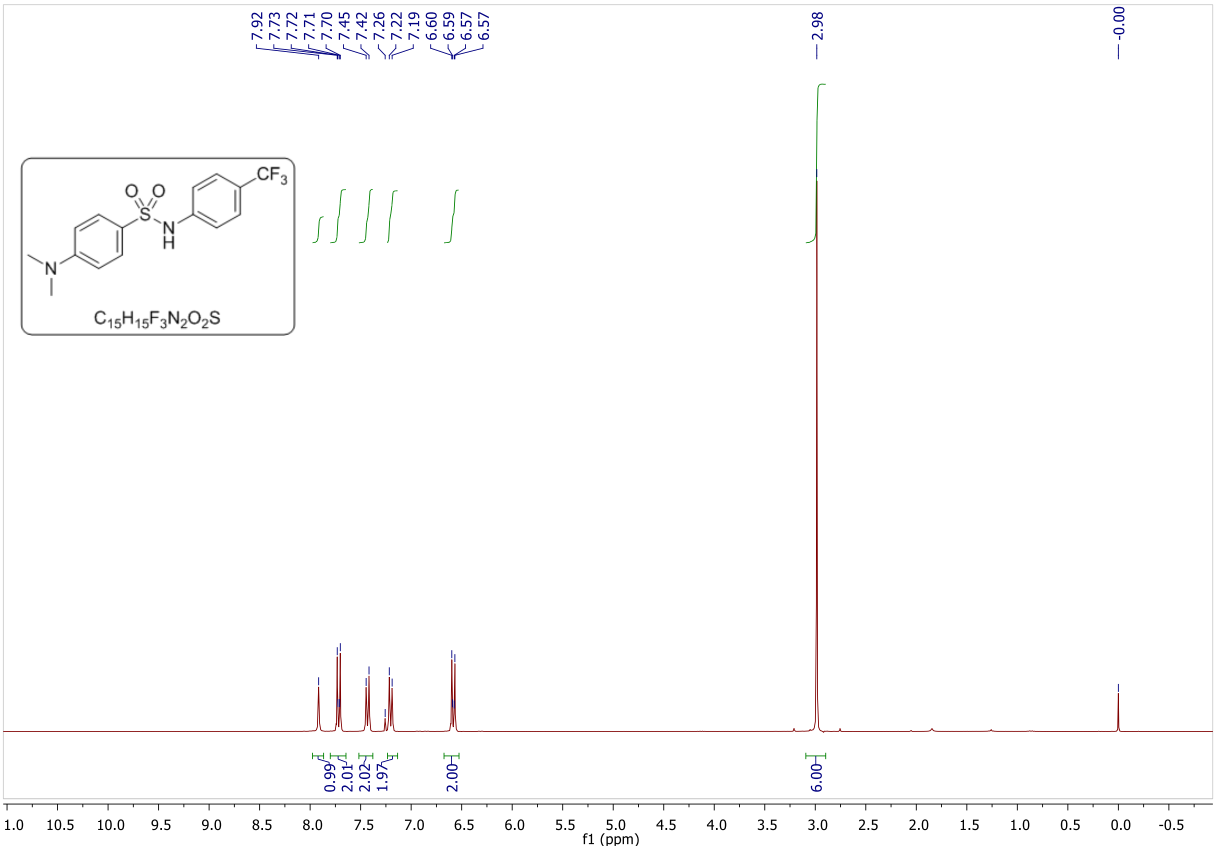

Supplementary Figure 104. ^{1}H-NMR (300 MHz) of **S2-o**.

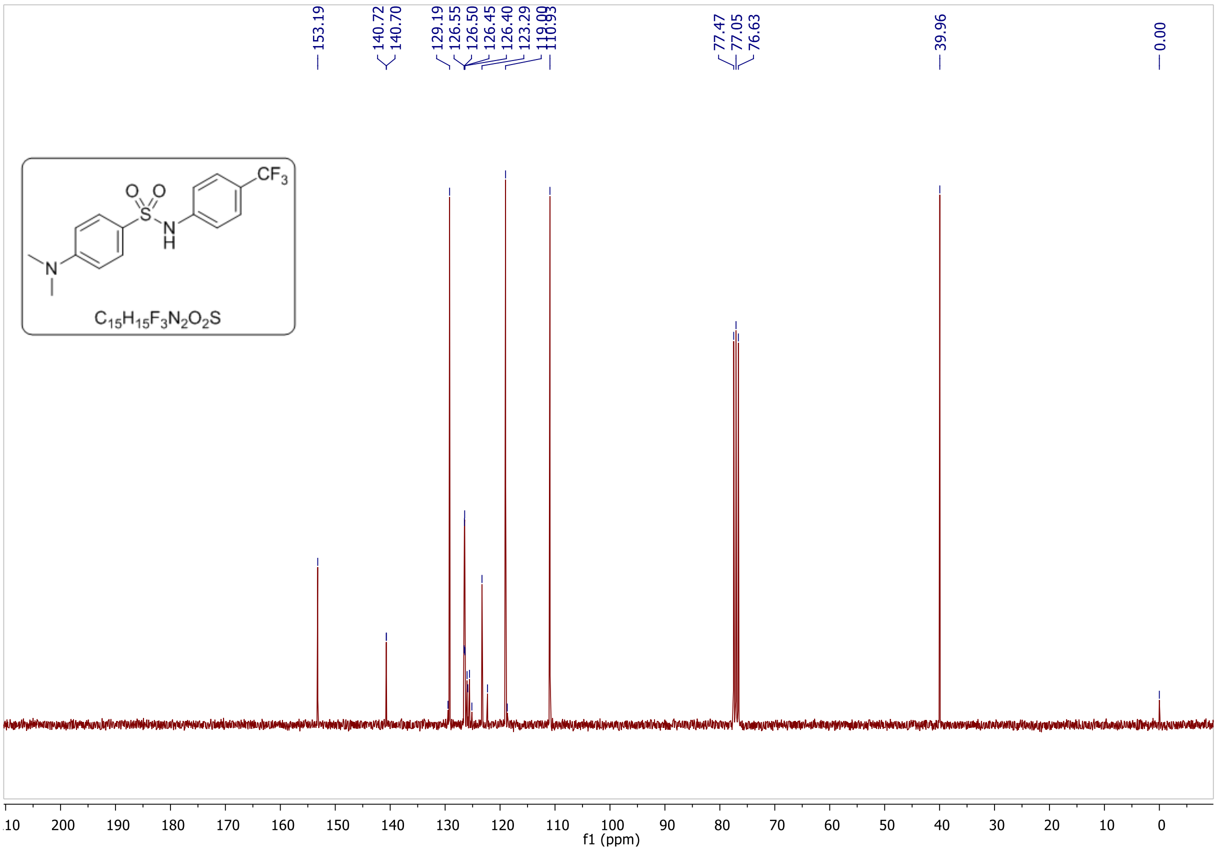

Supplementary Figure 105. ^{13}C-NMR (75 MHz) of **S2-o**.

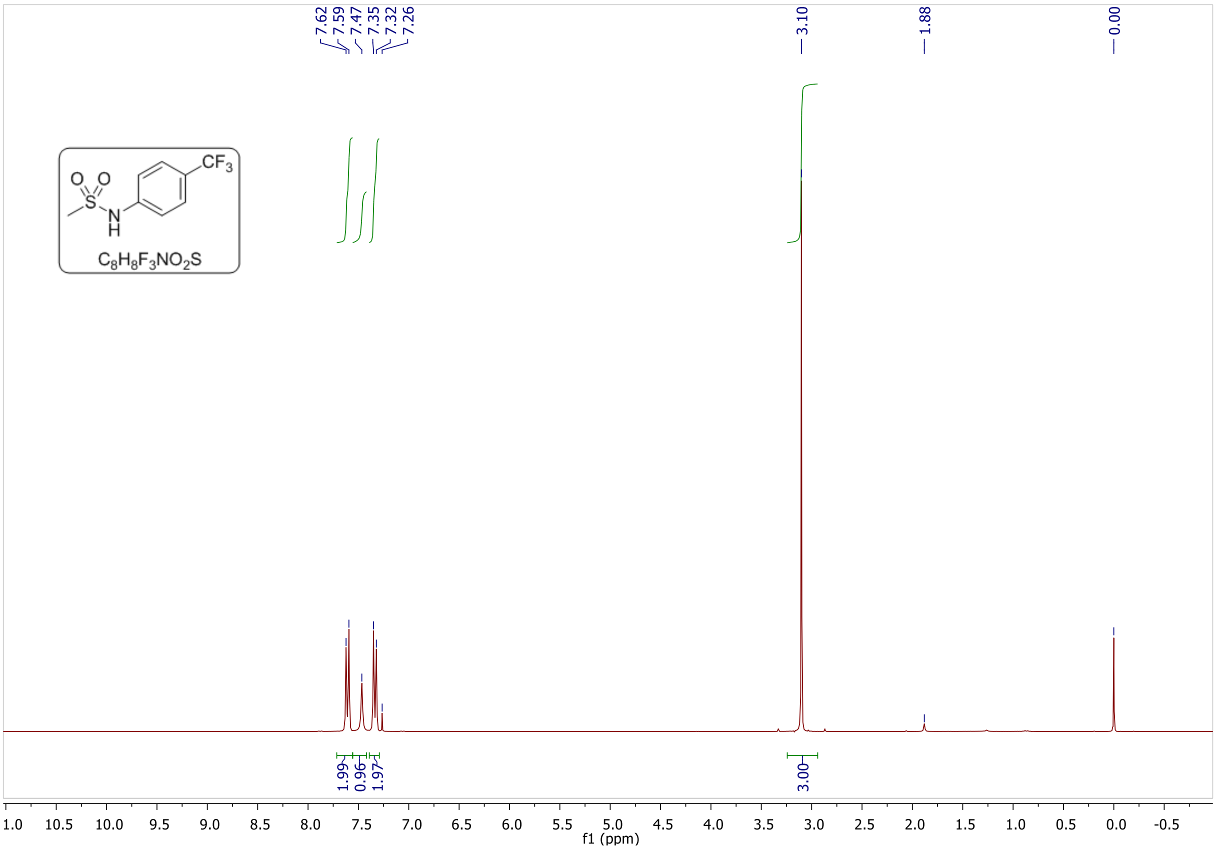

Supplementary Figure 106. ^{1}H-NMR (300 MHz) of **S2-p**.

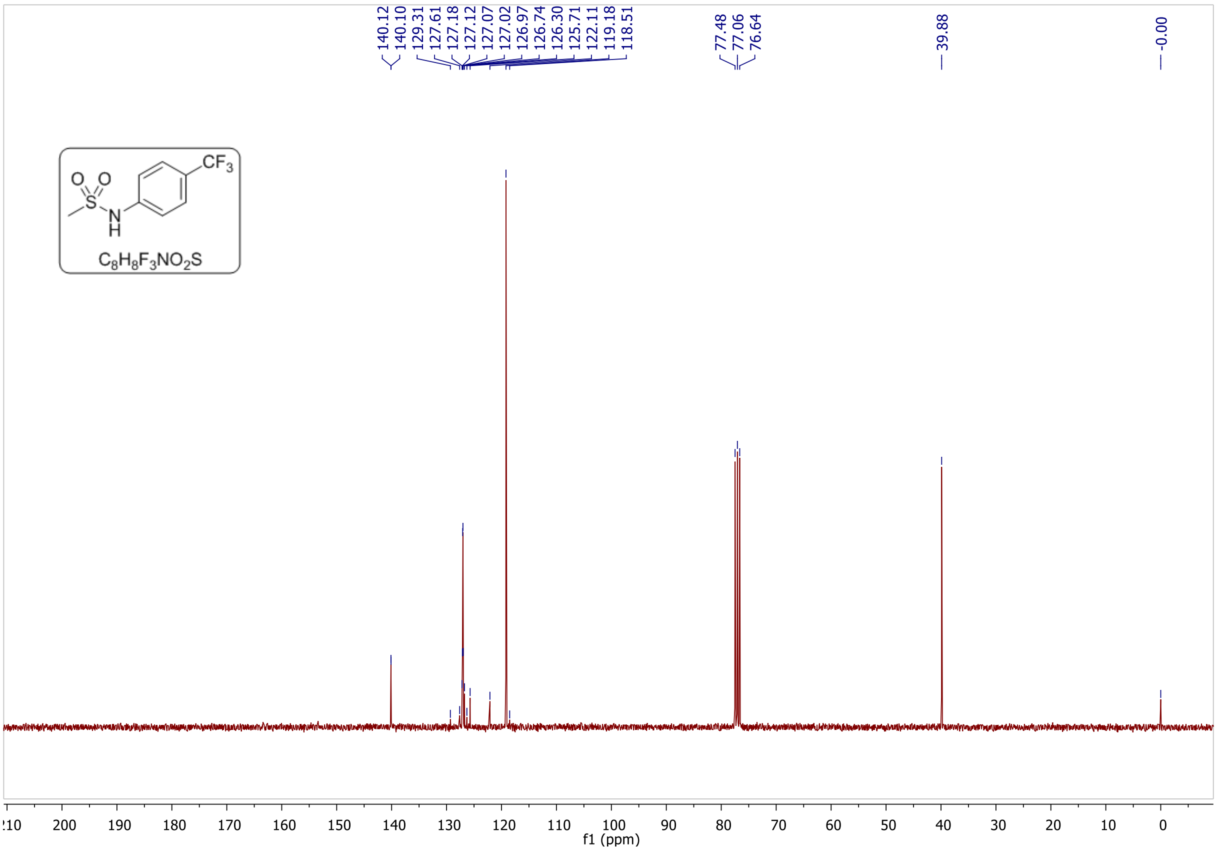

Supplementary Figure 107. ^{13}C-NMR (75 MHz) of **S2-p**.

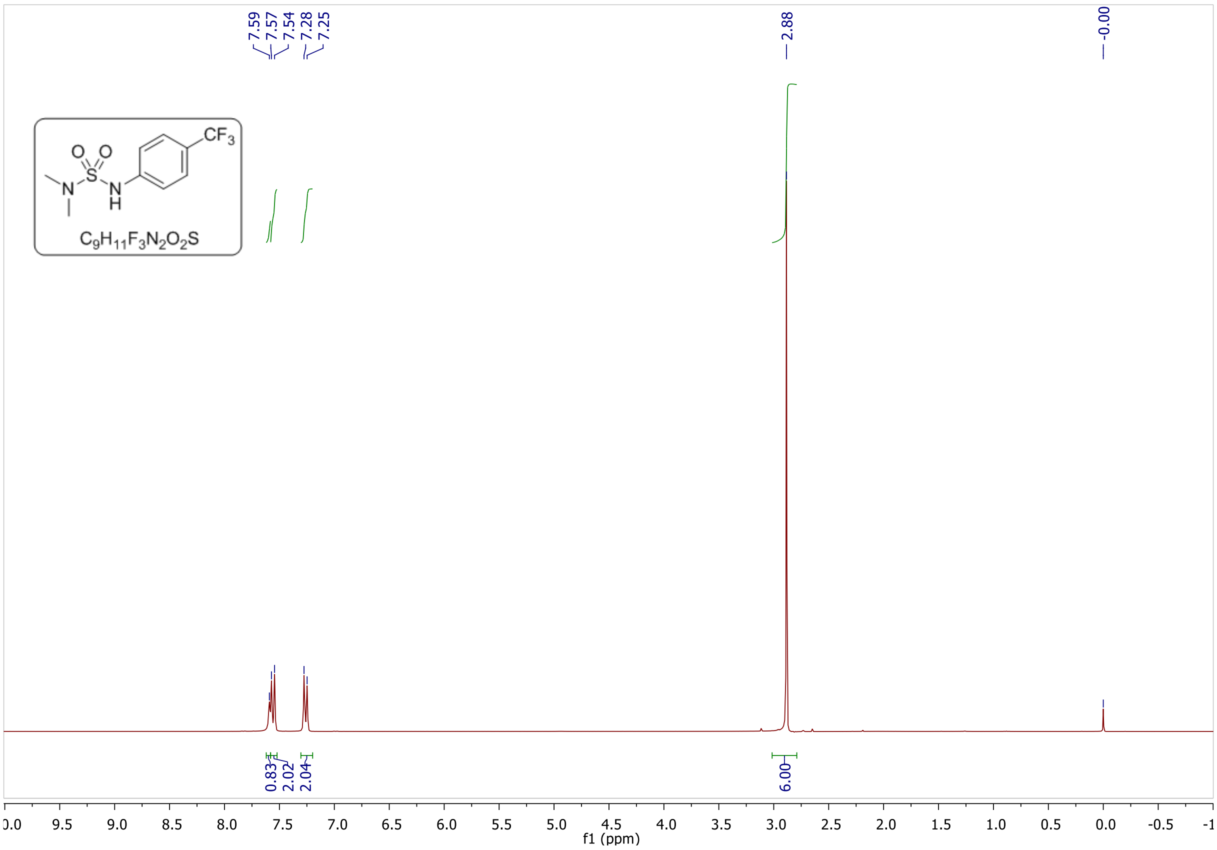

Supplementary Figure 108. ^{1}H-NMR (300 MHz) of **S2-q**.

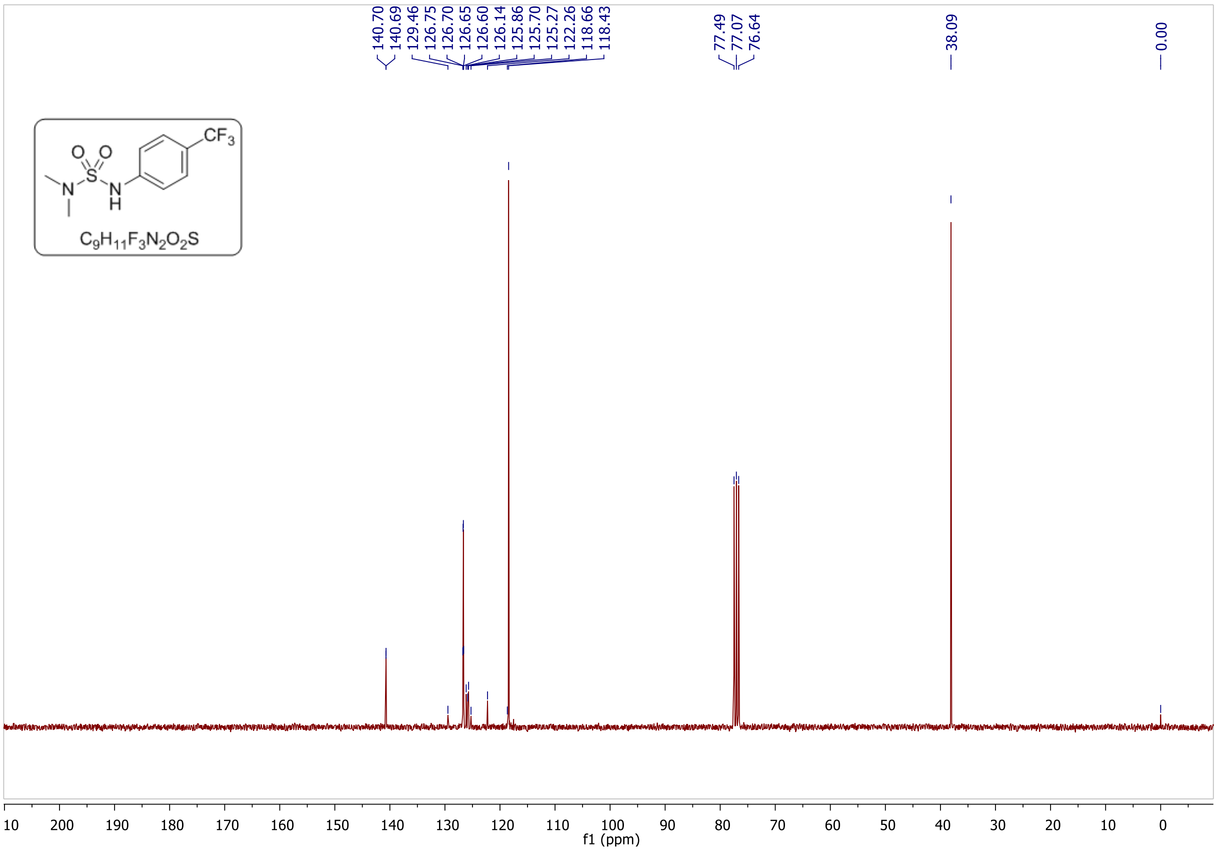

Supplementary Figure 109. ^{13}C-NMR (75 MHz) of **S2-q**.

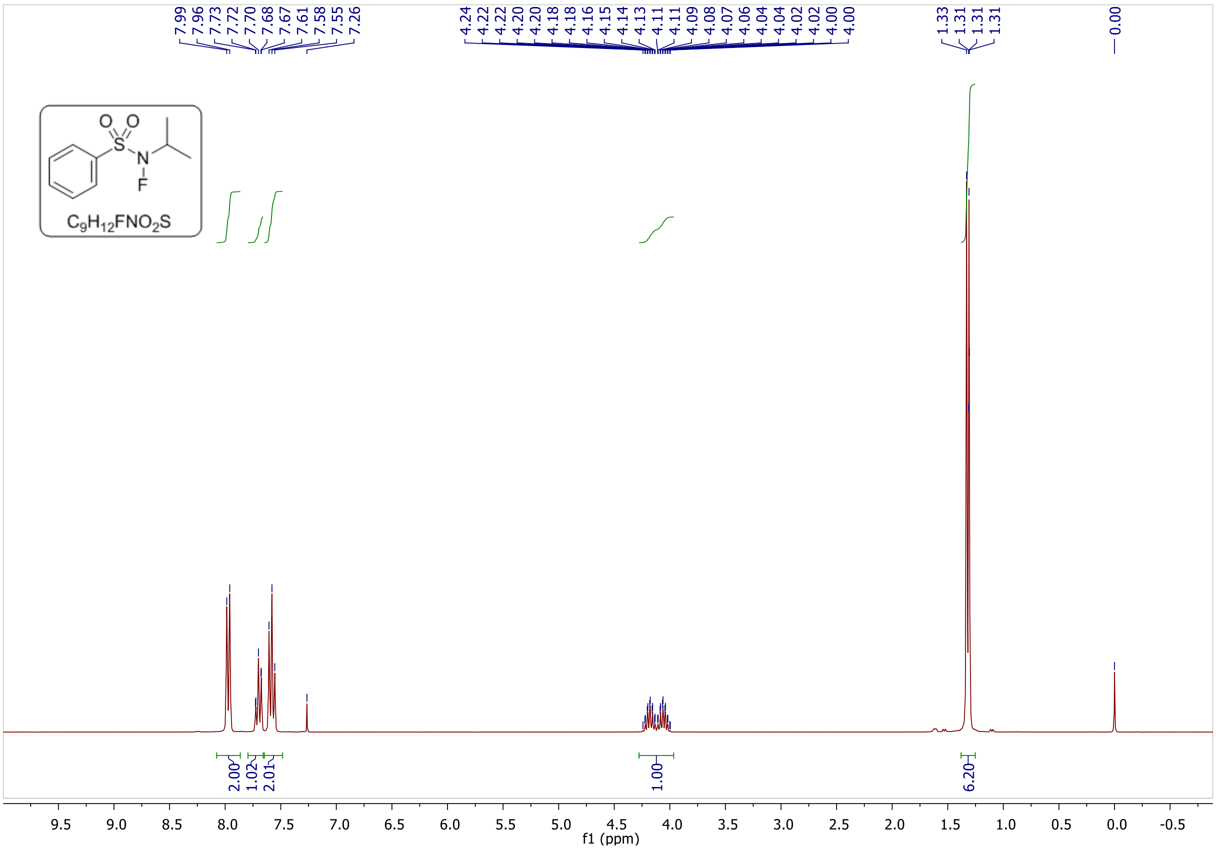

Supplementary Figure 110. ^{1}H-NMR (300 MHz) of **3a**.

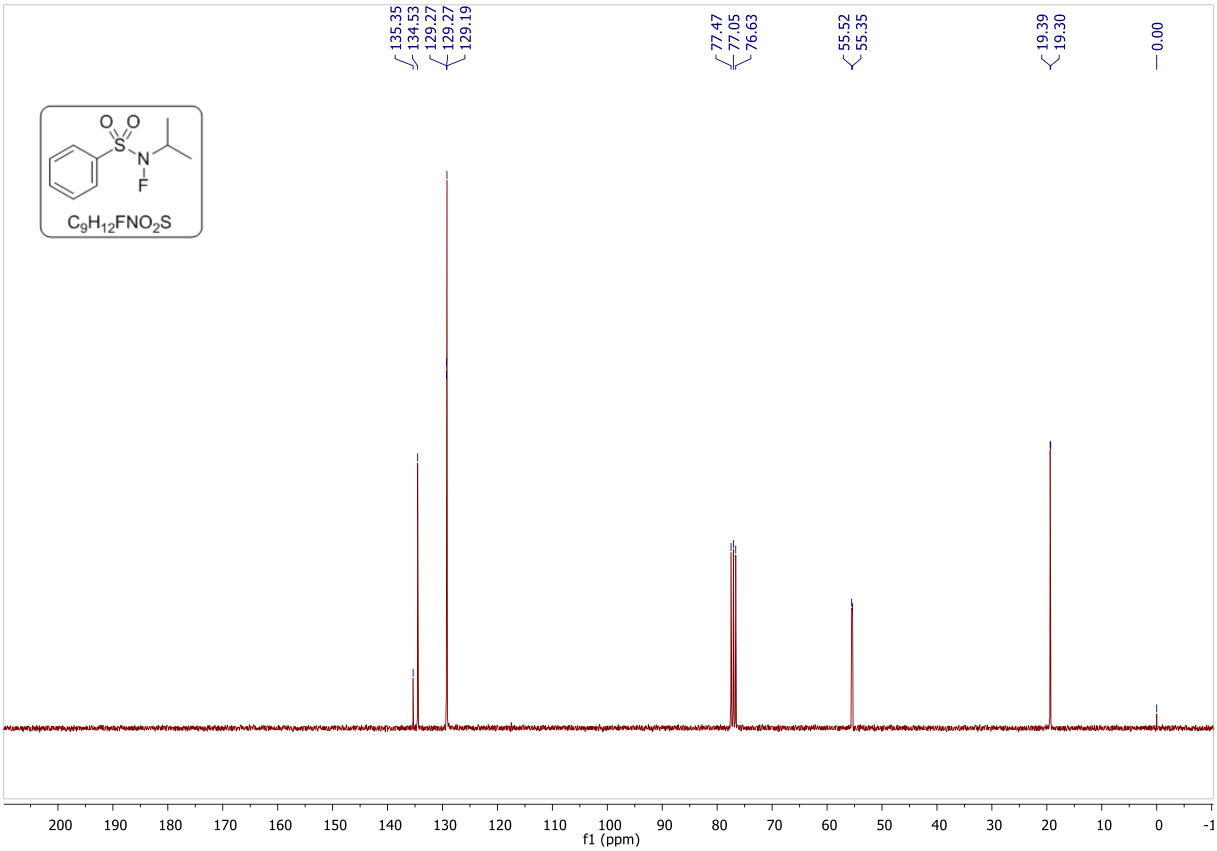

Supplementary Figure 111. ^{13}C-NMR (75 MHz) of **3a**.

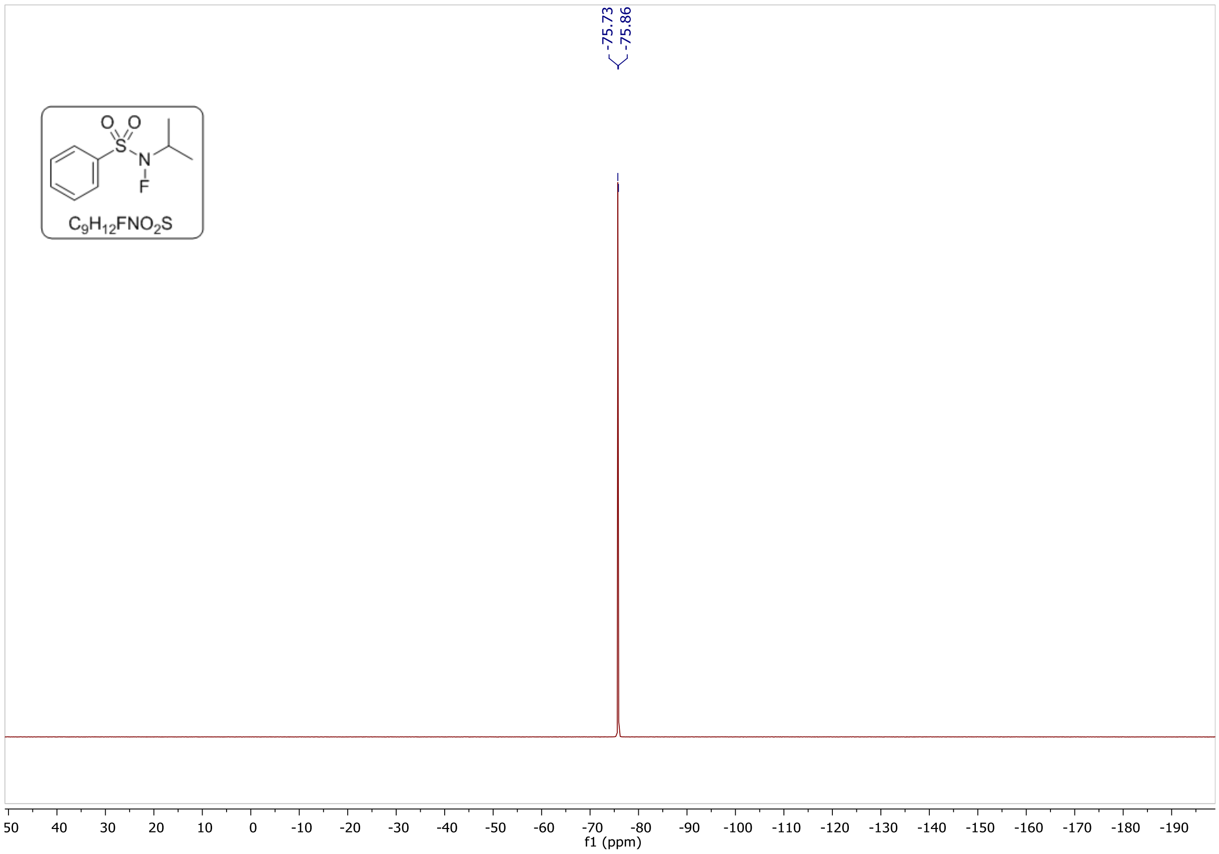

Supplementary Figure 112. ^{19}F-NMR (282 MHz) of **3a**.

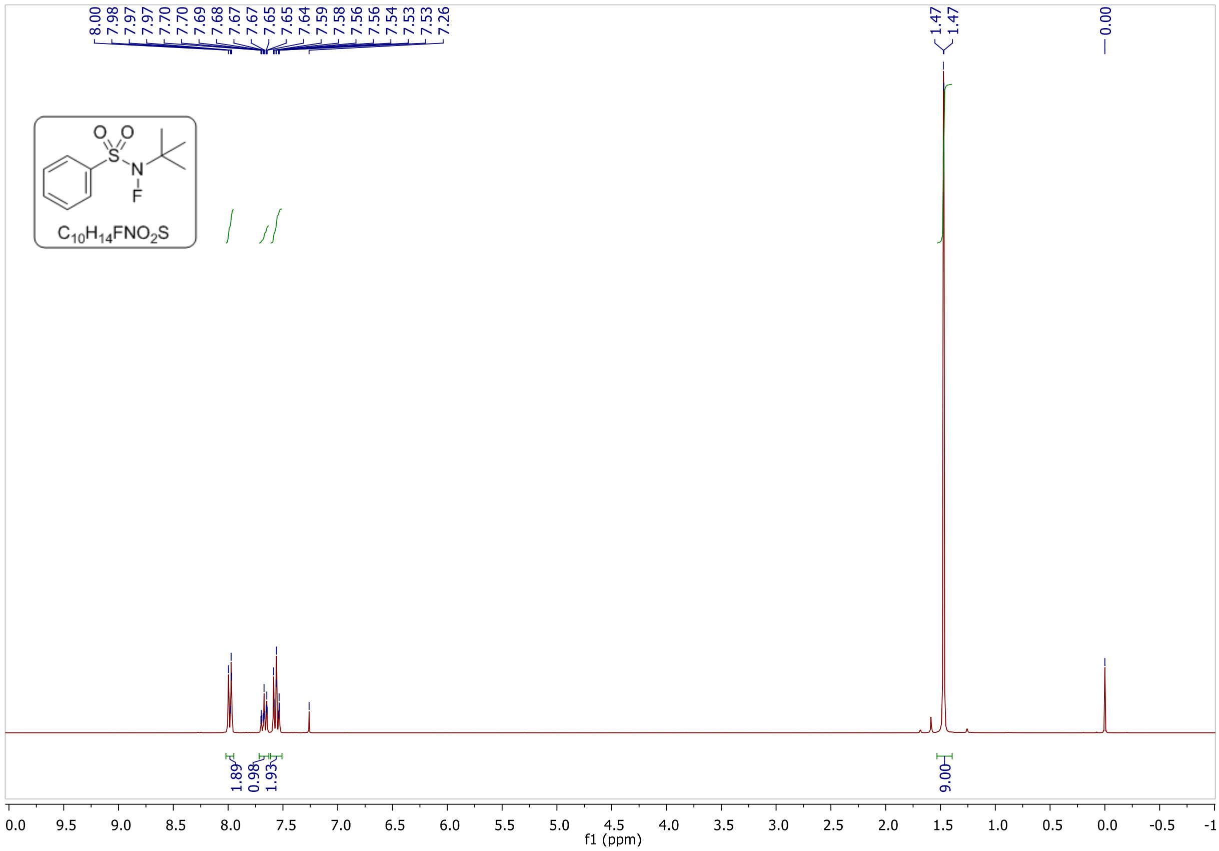

Supplementary Figure 113. ^{1}H-NMR (300 MHz) of **3b**.

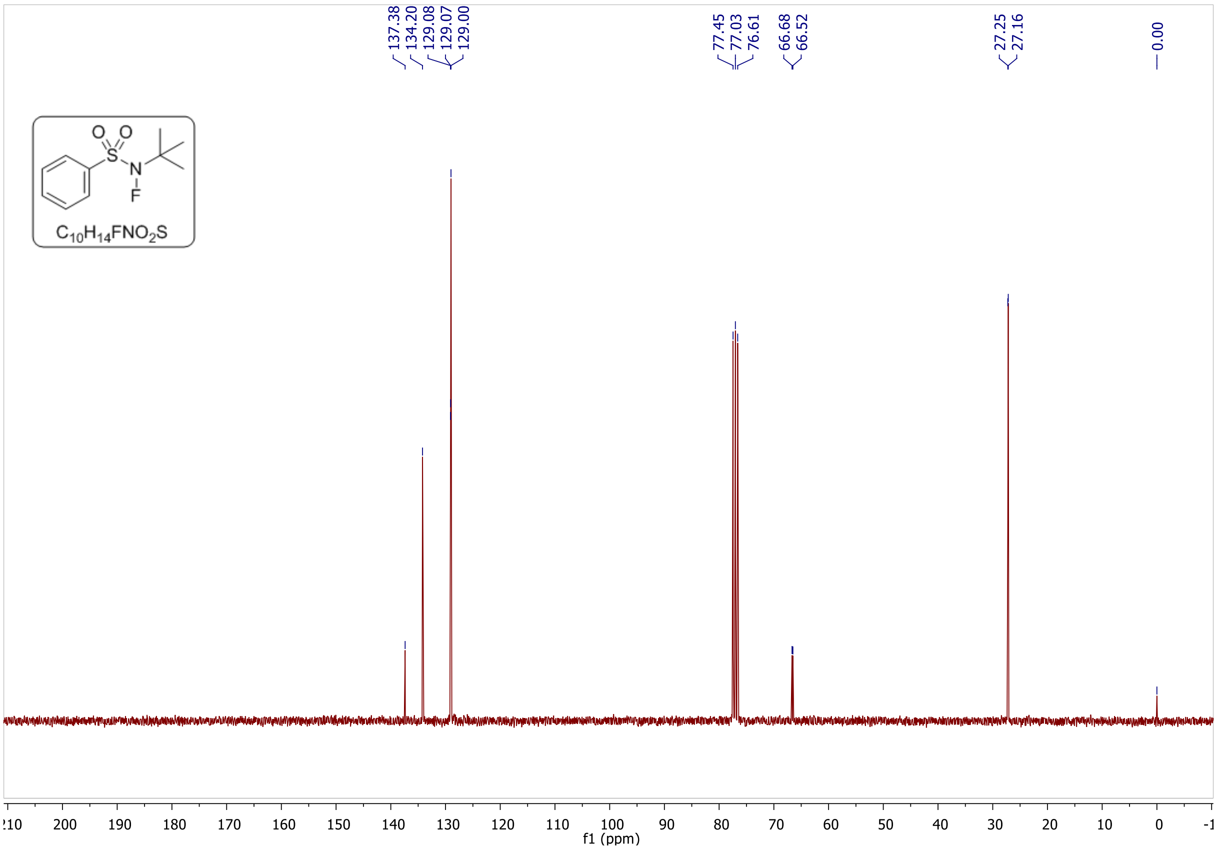

Supplementary Figure 114. ^{13}C-NMR (75 MHz) of **3b**.

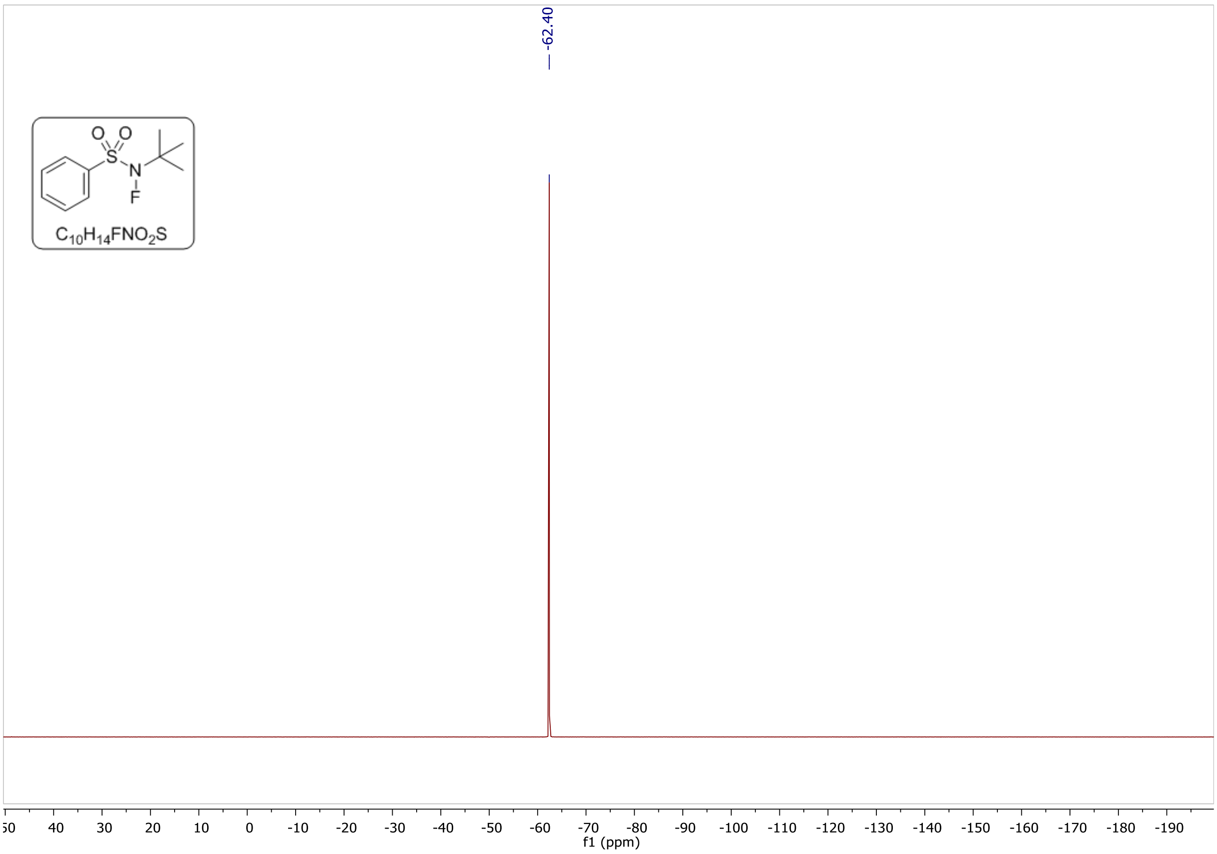

Supplementary Figure 115. ^{19}F-NMR (282 MHz) of **3b**.

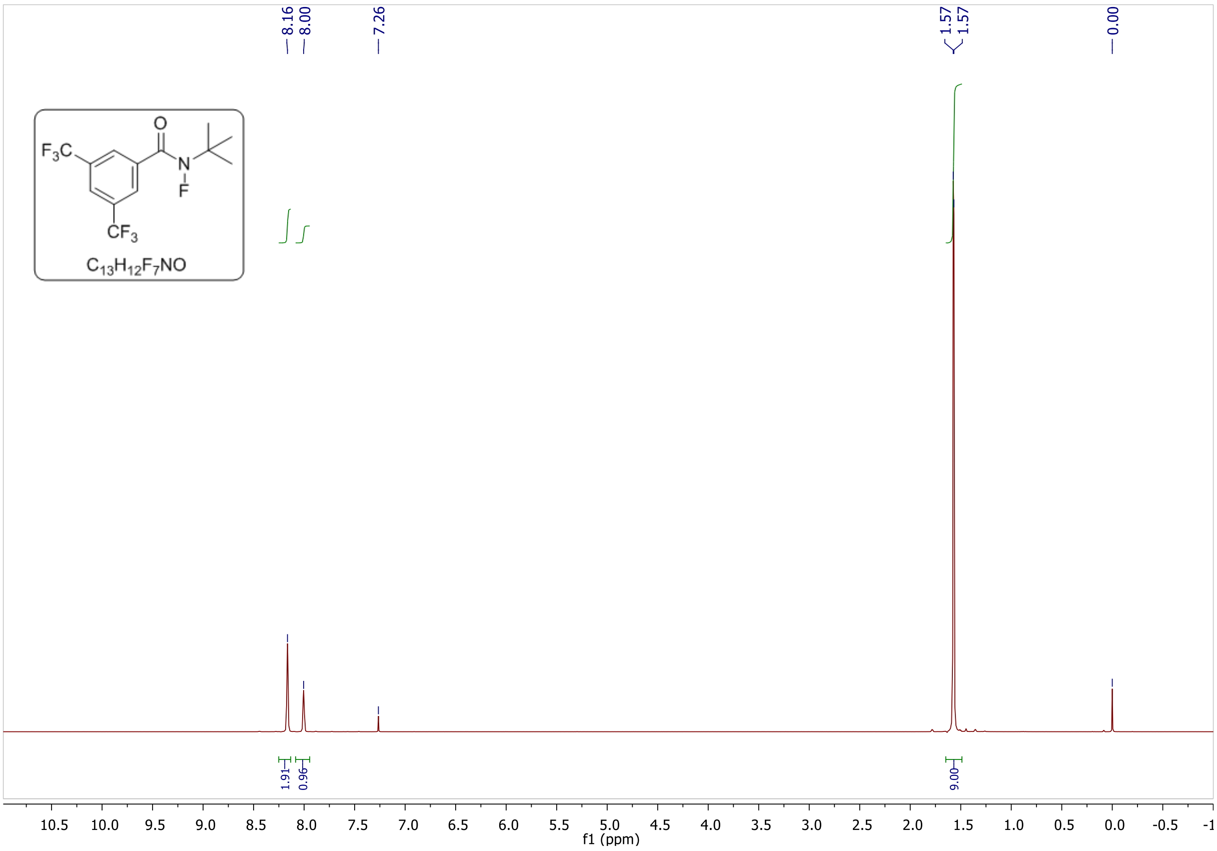

Supplementary Figure 116. ^{1}H-NMR (300 MHz) of **3c**.

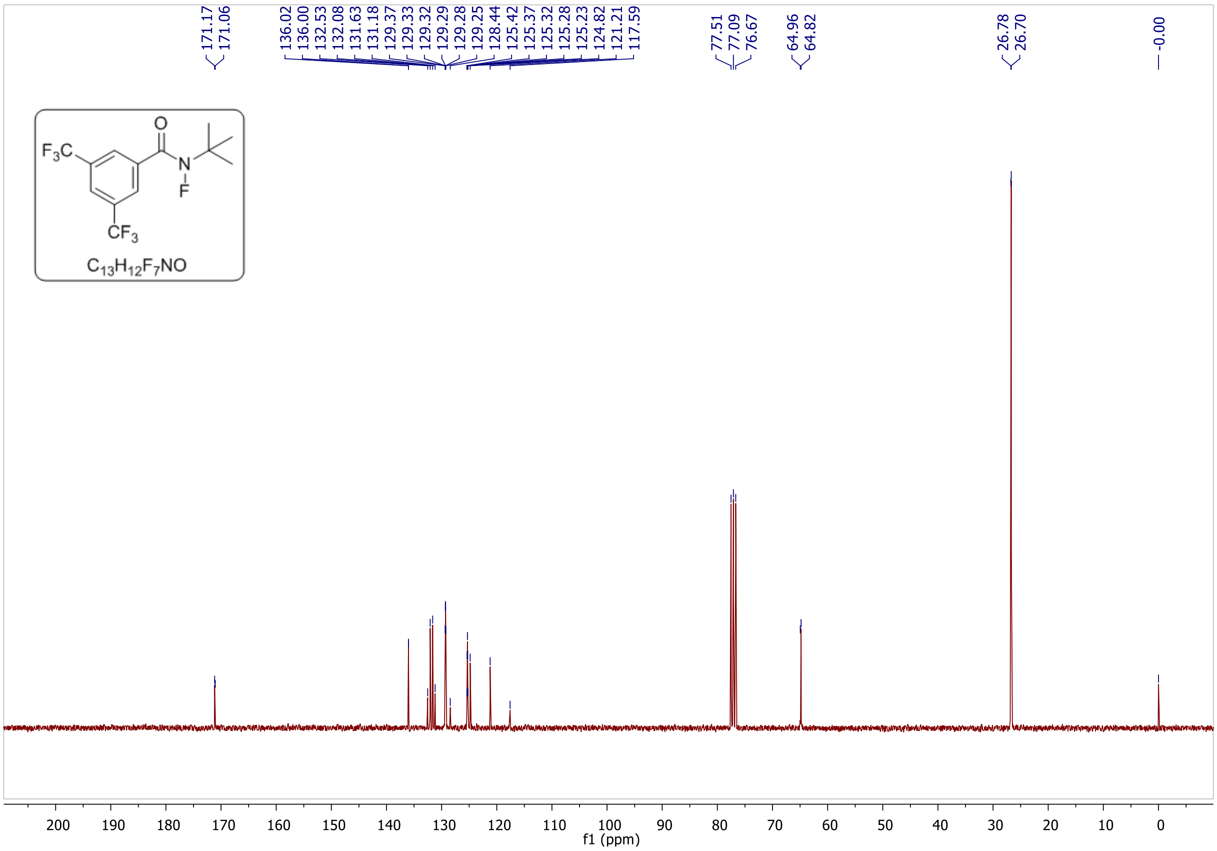

Supplementary Figure 117. ^{13}C-NMR (75 MHz) of **3c**.

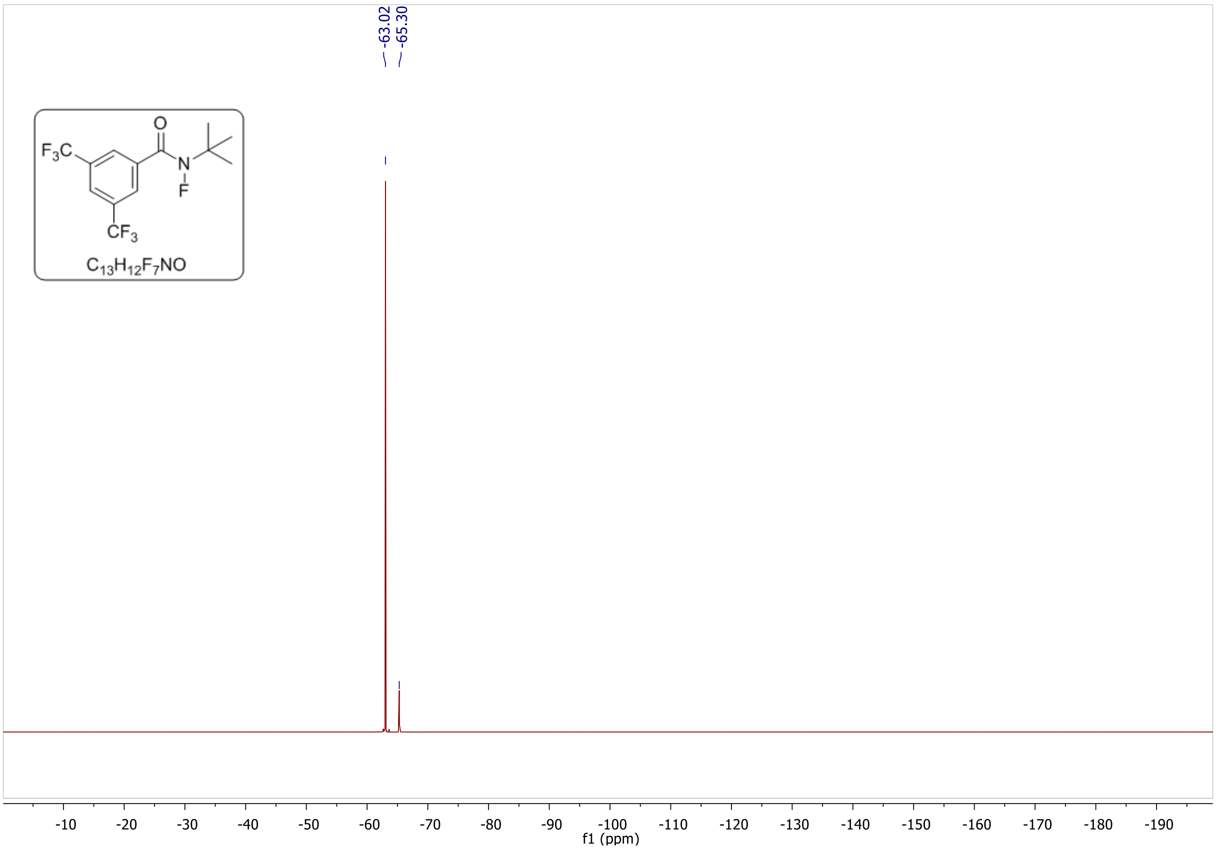

Supplementary Figure 118. ^{19}F-NMR (282 MHz) of **3c**.

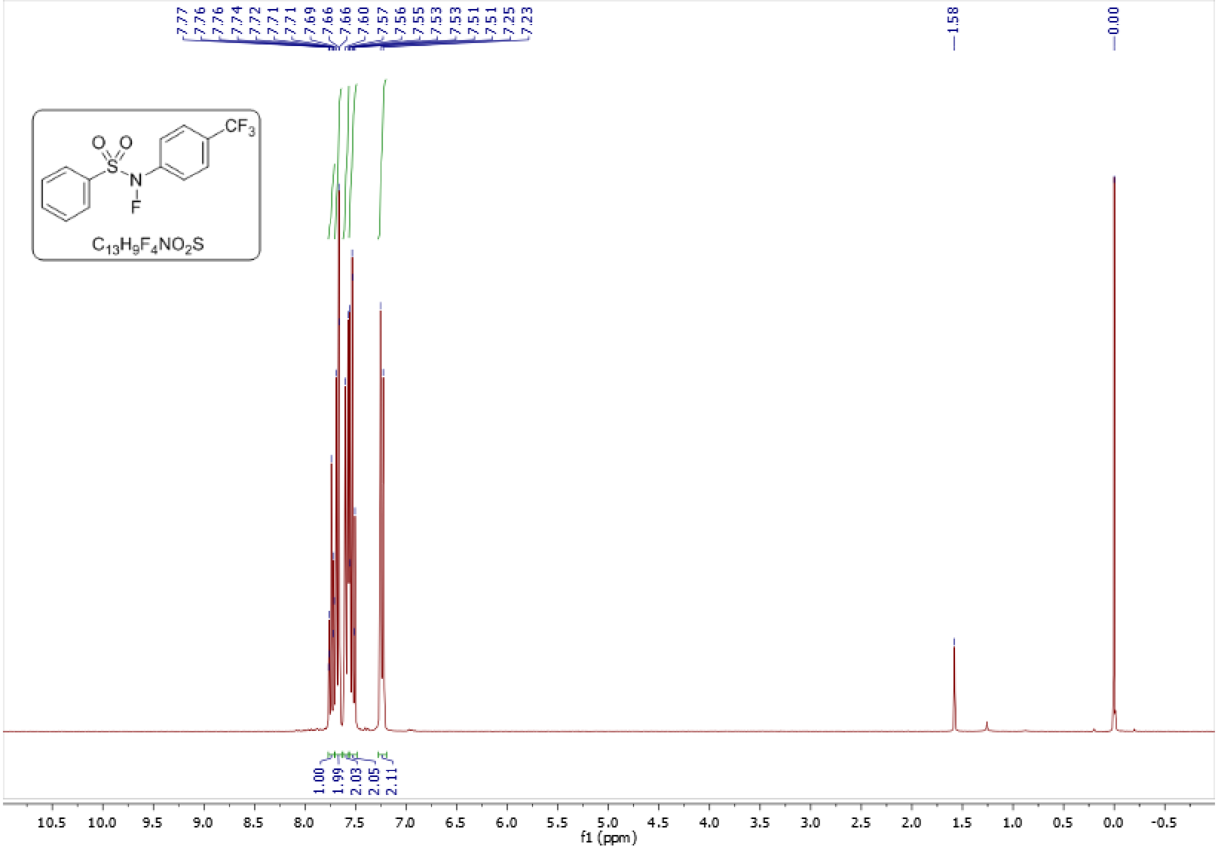

Supplementary Figure 119. ^{1}H-NMR (300 MHz) of **4a**.

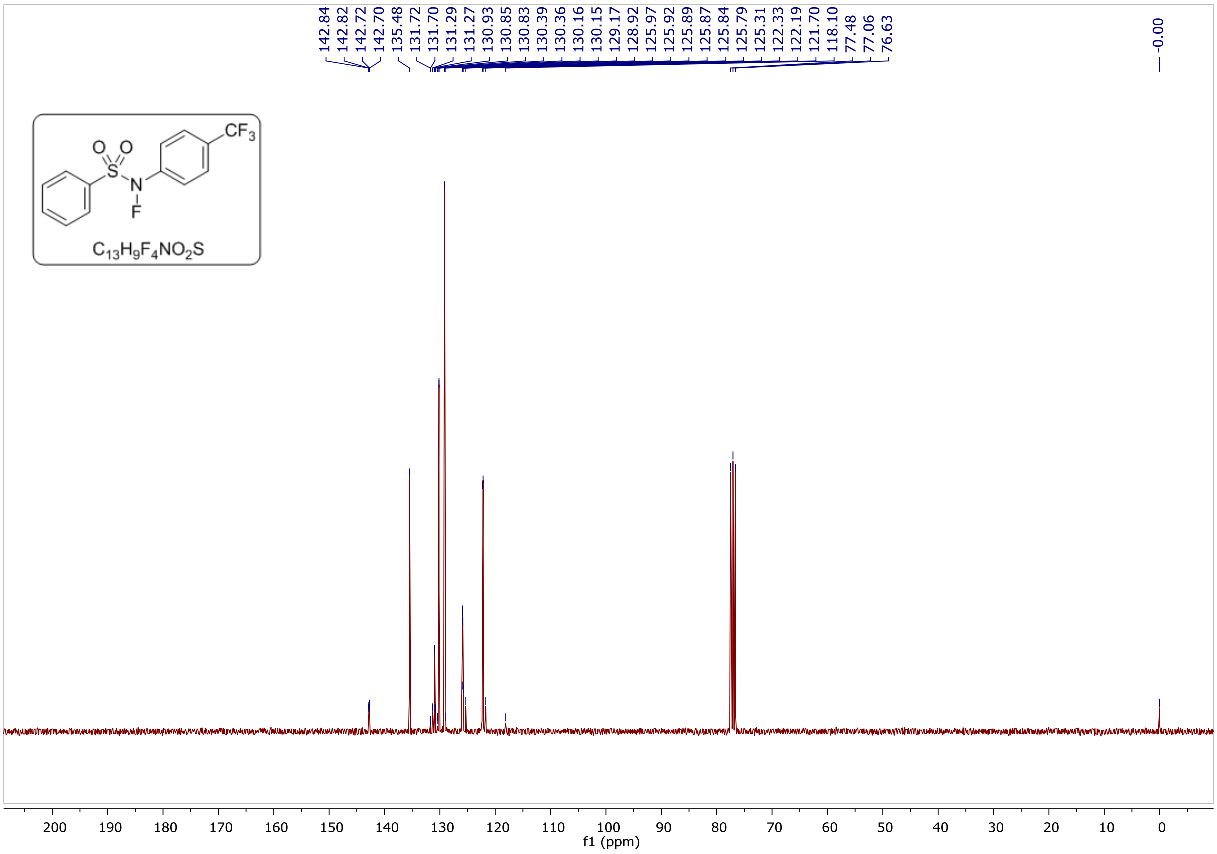

Supplementary Figure 120. ^{13}C-NMR (75 MHz) of **4a**.

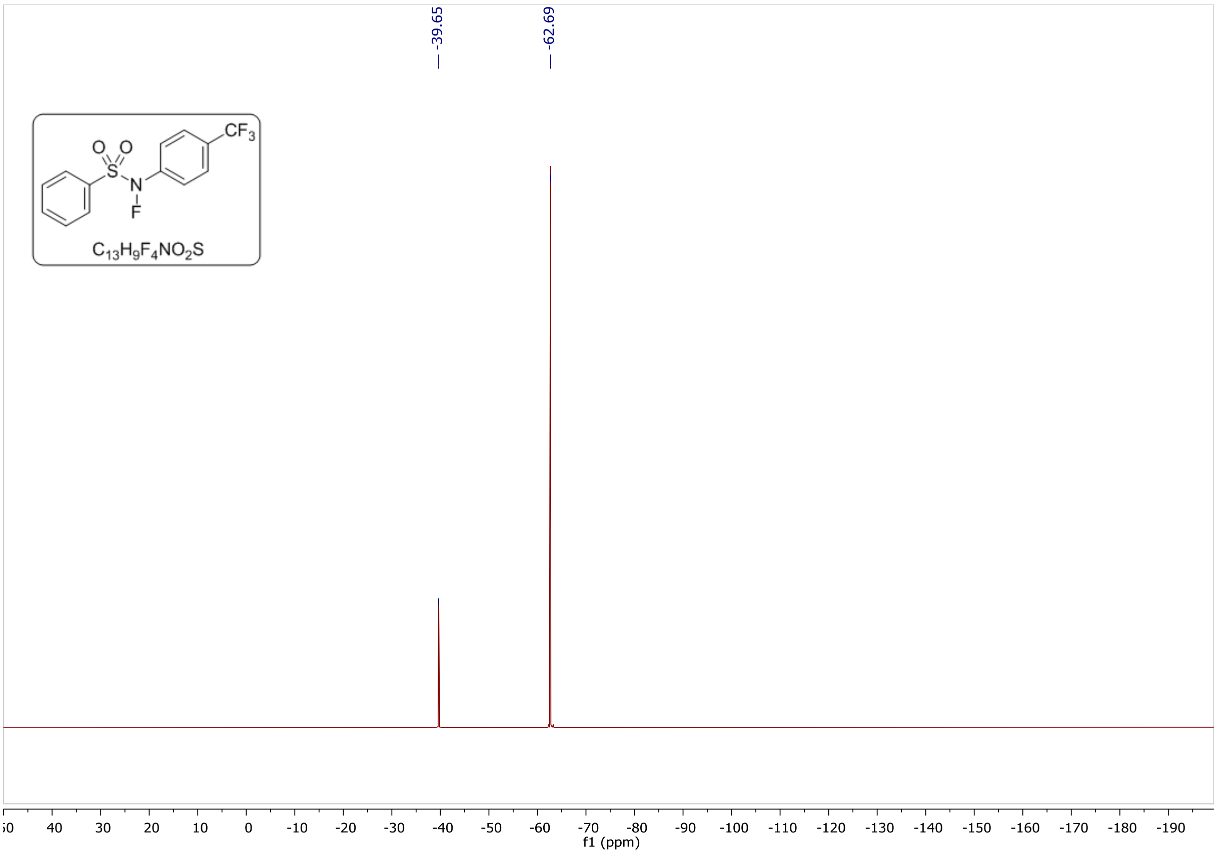

Supplementary Figure 121. ^{19}F-NMR (282 MHz) of **4a**.

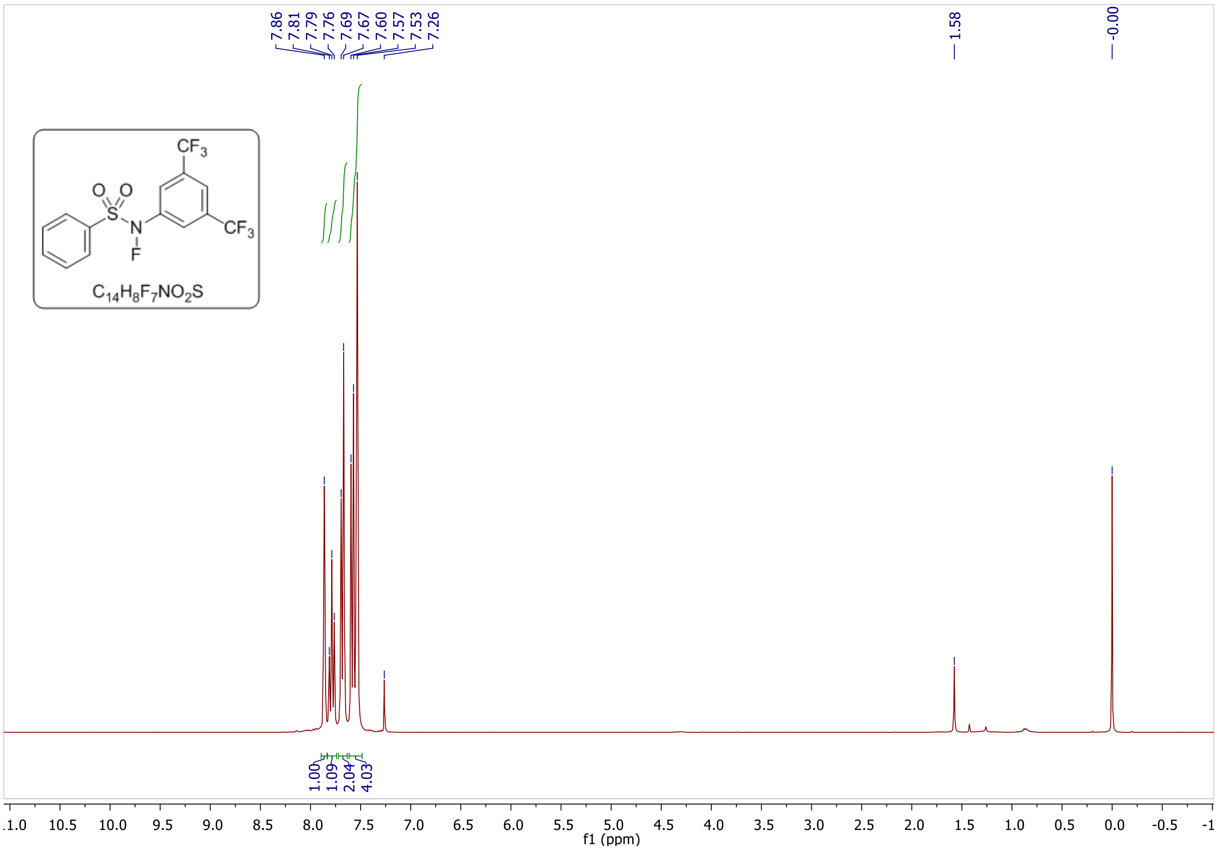

Supplementary Figure 122. ^{1}H-NMR (300 MHz) of **4b**.

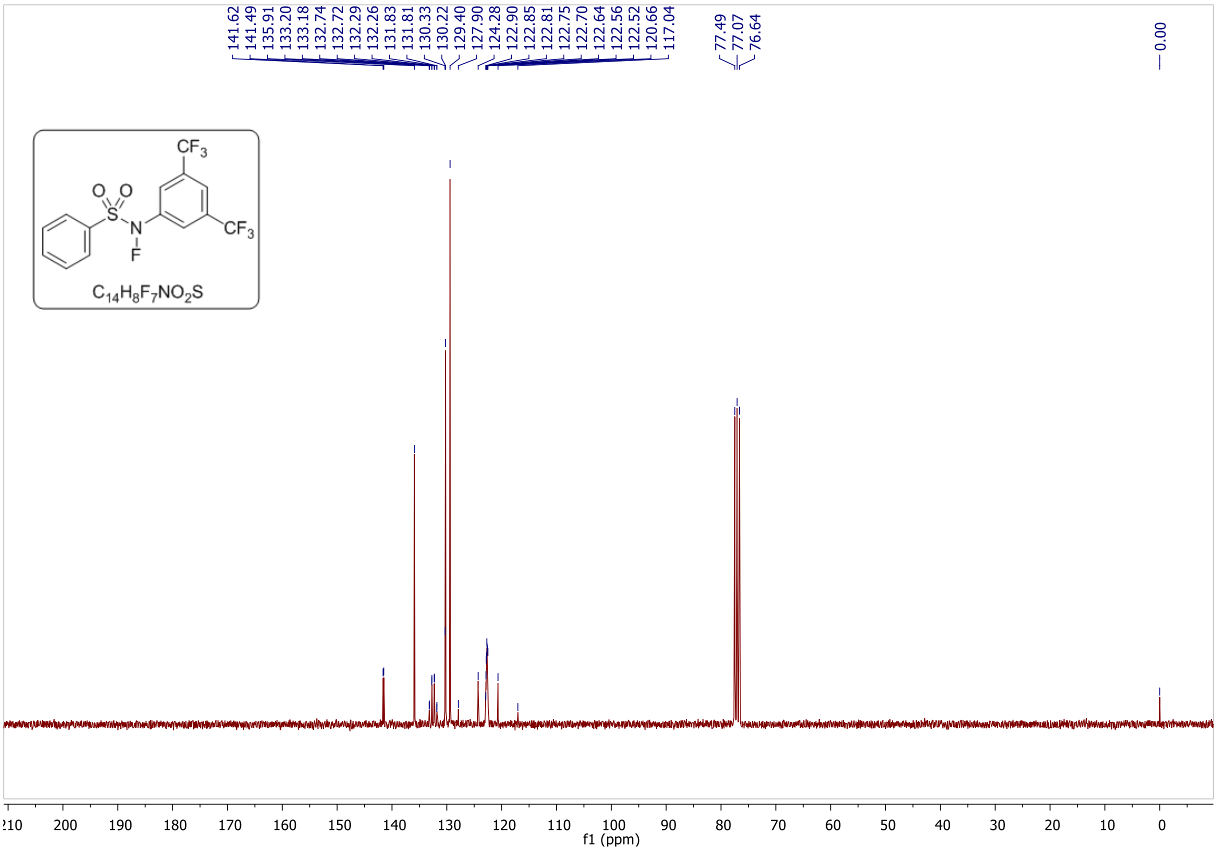

Supplementary Figure 123. ^{13}C-NMR (75 MHz) of **4b**.

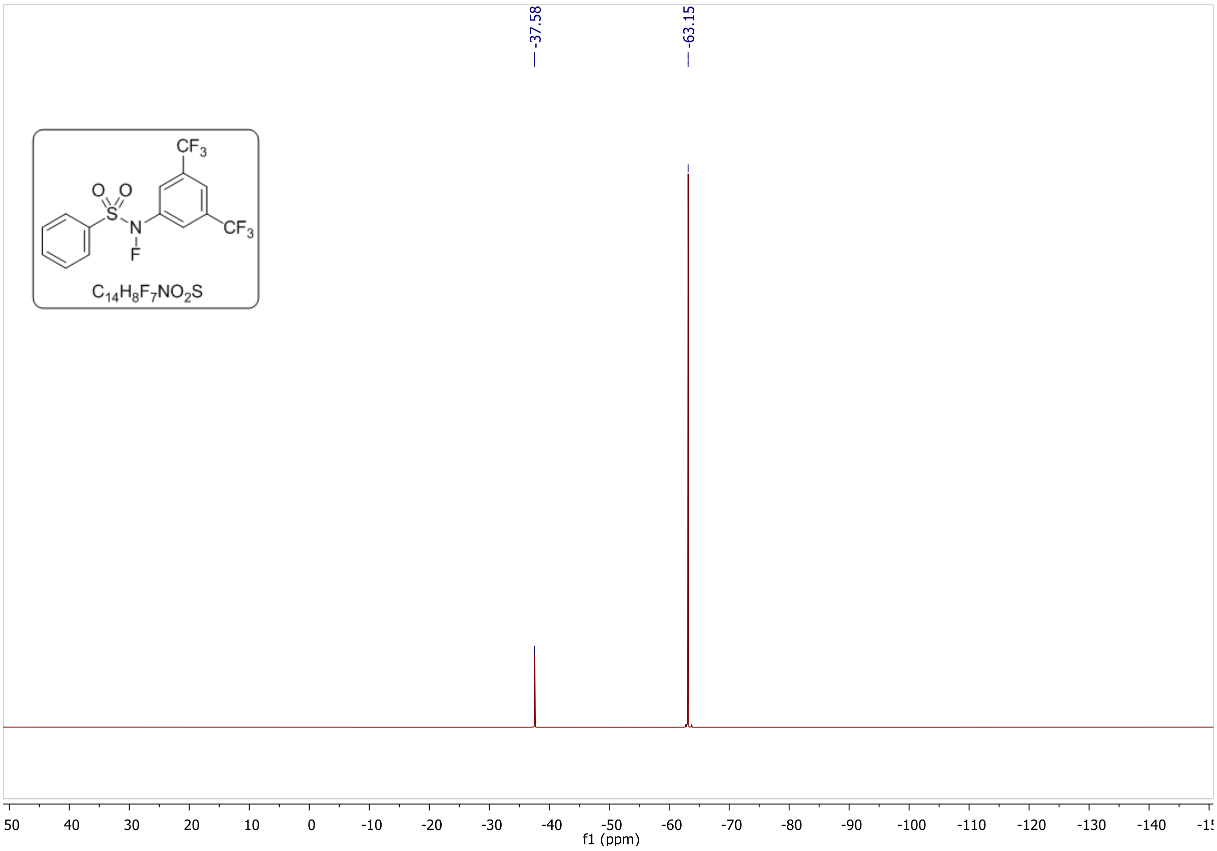

Supplementary Figure 124. ^{19}F-NMR (282 MHz) of **4b**.

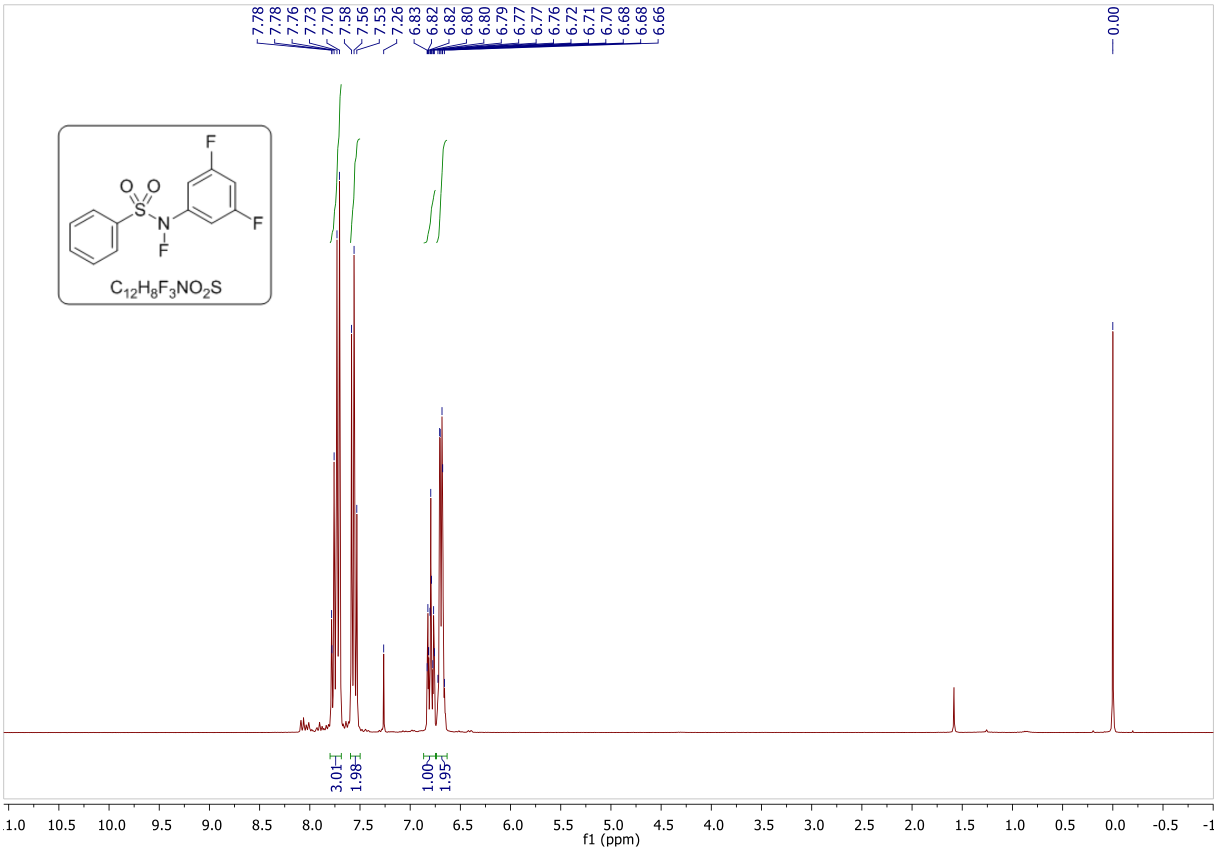

Supplementary Figure 125. ^{1}H-NMR (300 MHz) of **4c**.

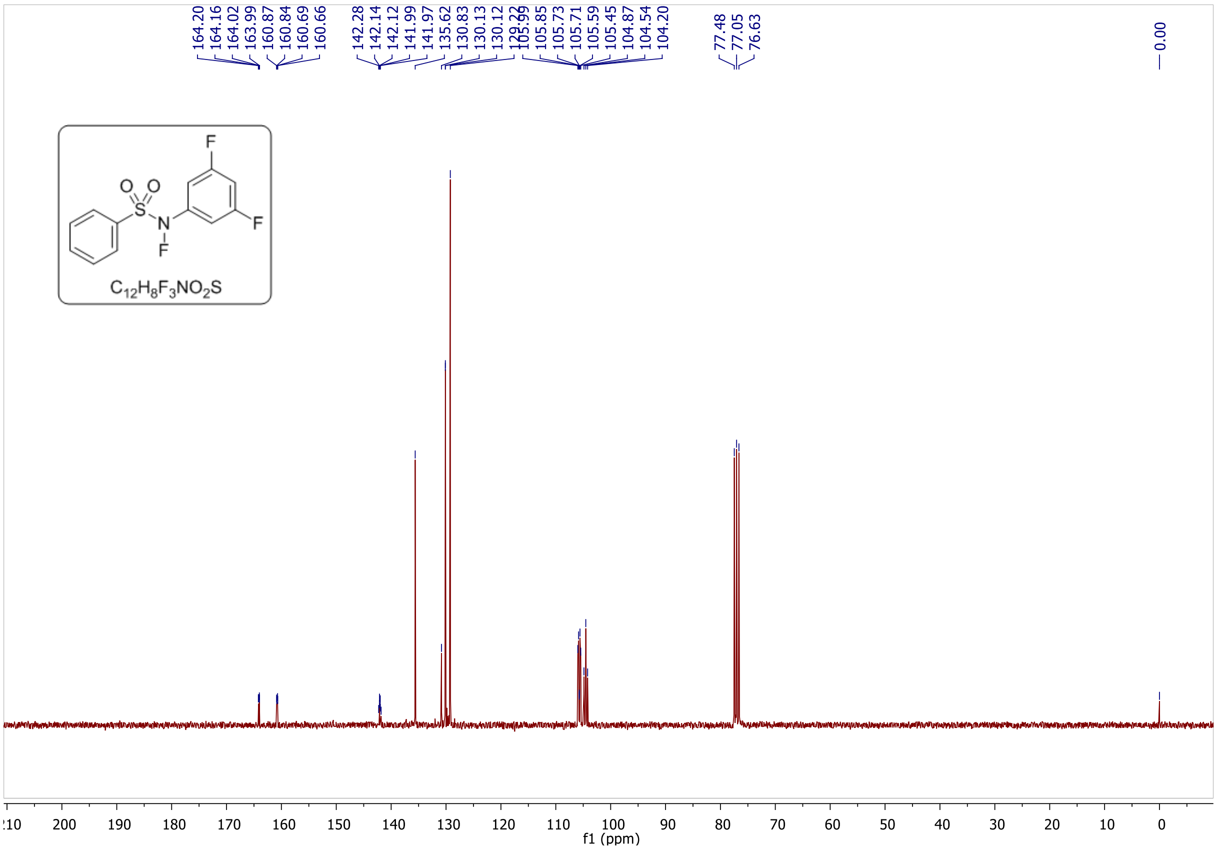

Supplementary Figure 126. ^{13}C-NMR (75 MHz) of **4c**.

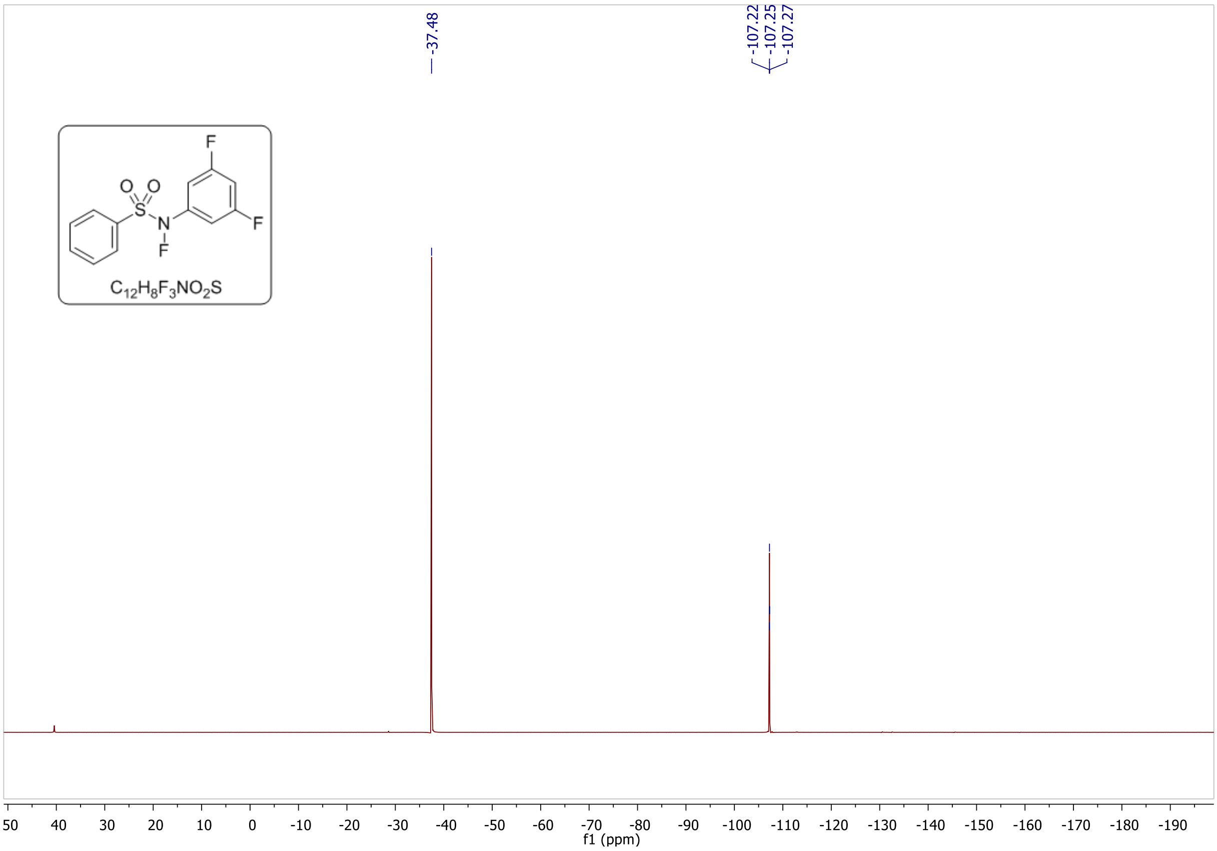

Supplementary Figure 127. ^{19}F-NMR (282 MHz) of **4c**.

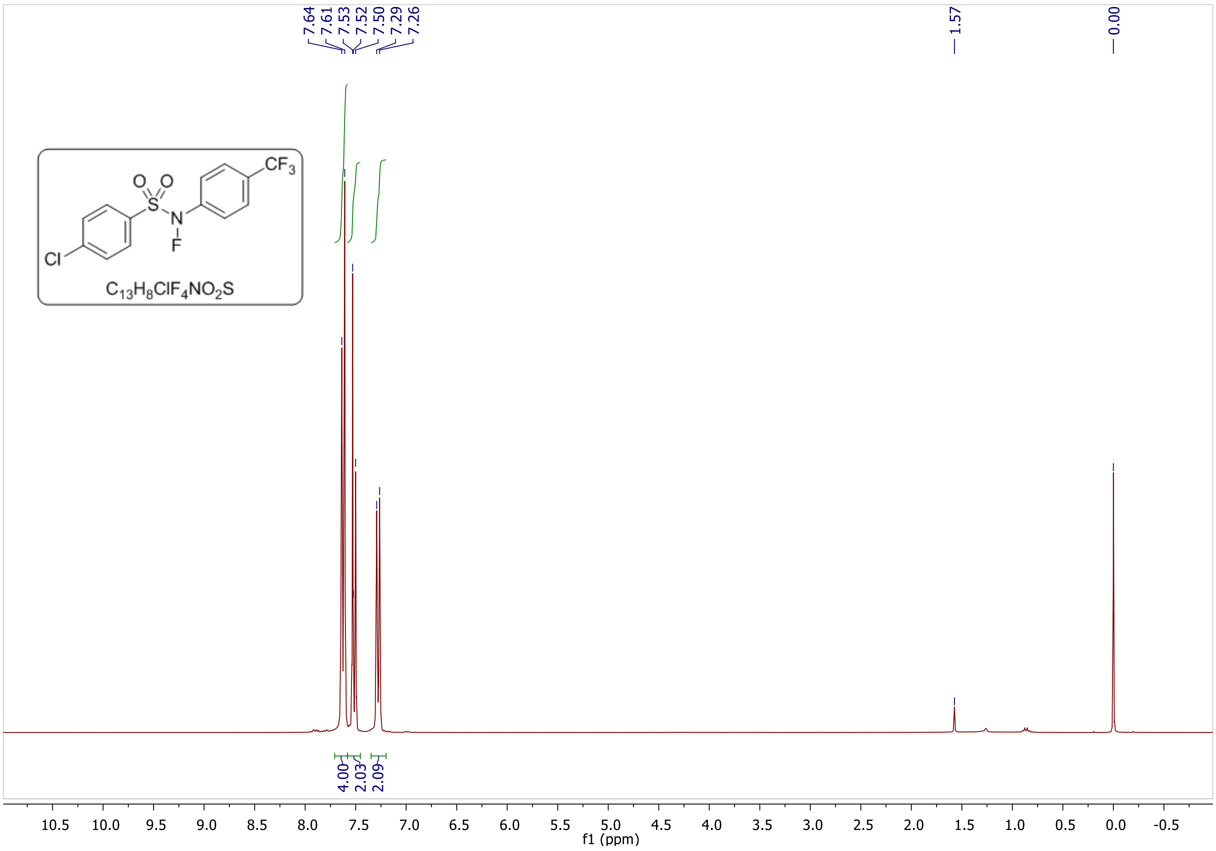

Supplementary Figure 128. ^{1}H-NMR (300 MHz) of **4d**.

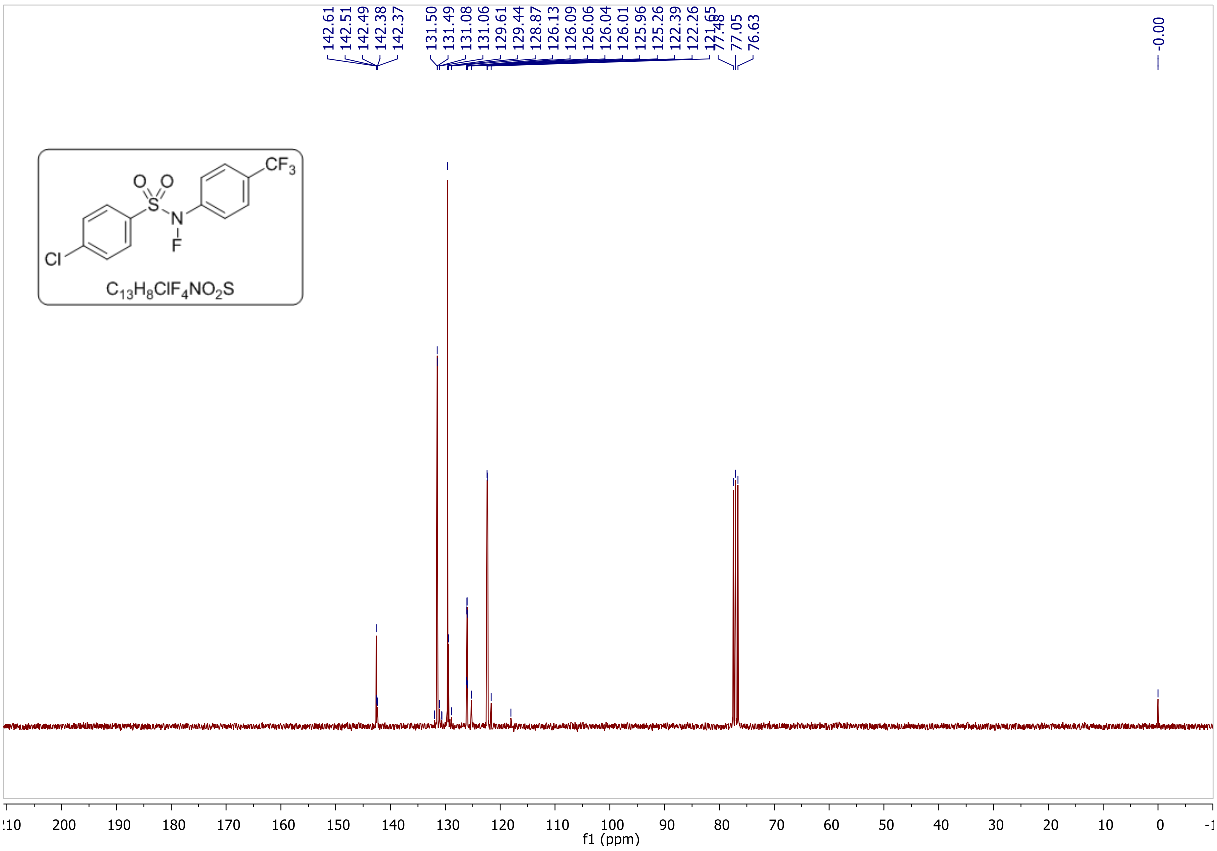

Supplementary Figure 129. ^{13}C-NMR (75 MHz) of **4d**.

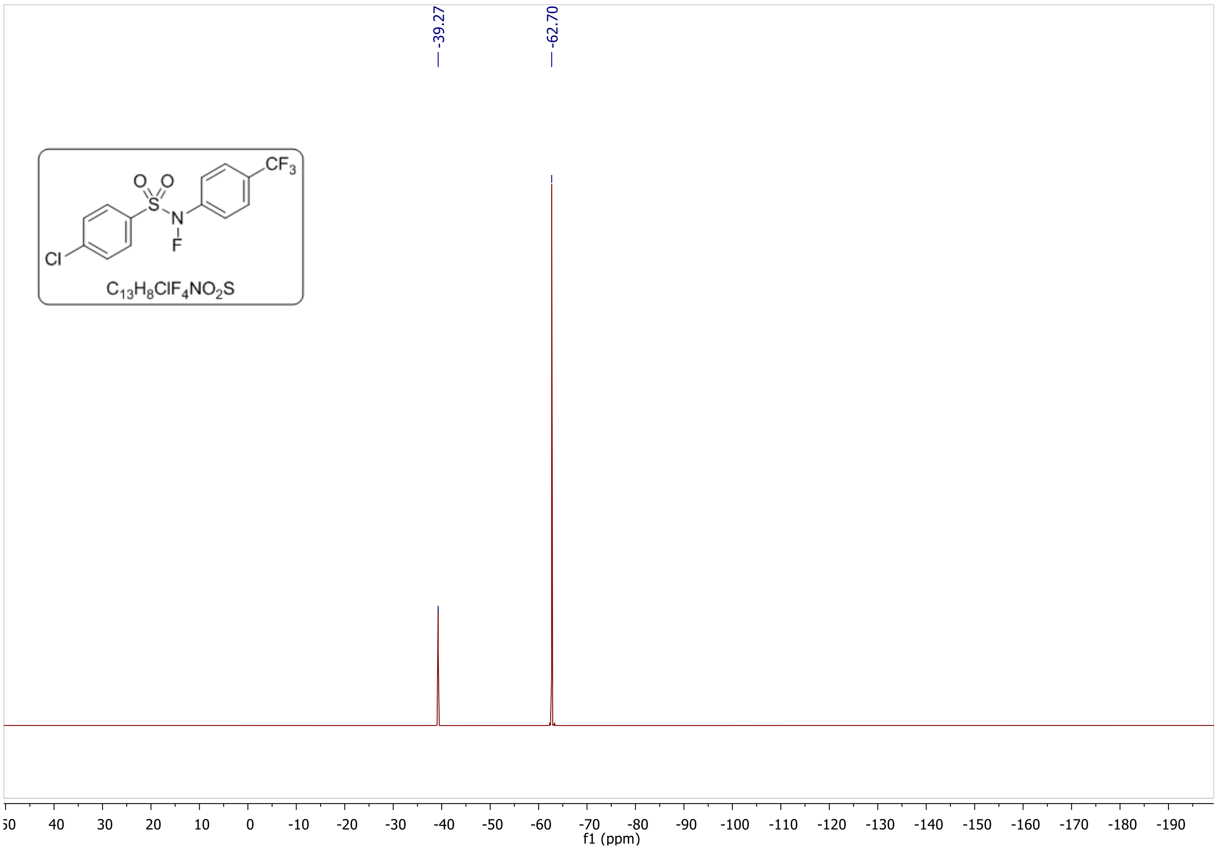

Supplementary Figure 130. ^{19}F-NMR (282 MHz) of **4d**.

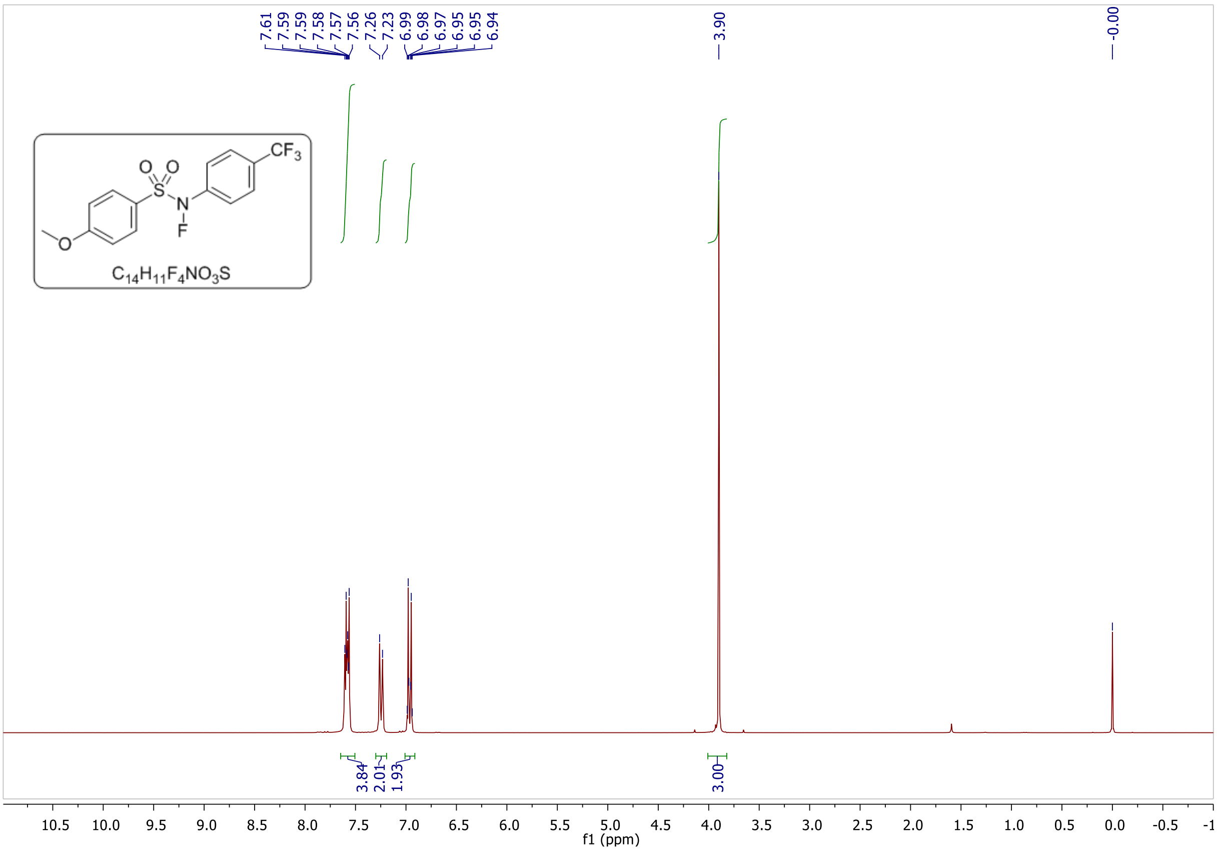

Supplementary Figure 131. ^{1}H-NMR (300 MHz) of **4e**.

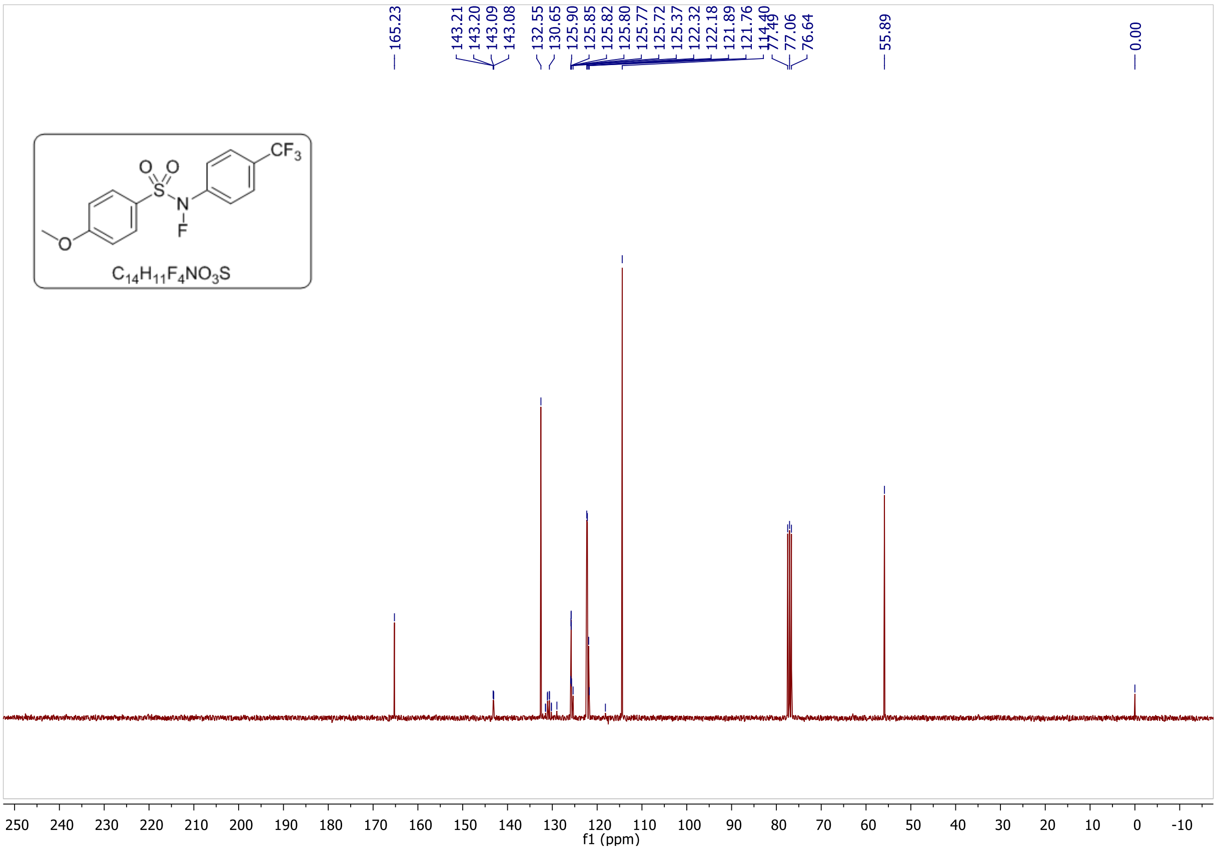

Supplementary Figure 132. ^{13}C-NMR (75 MHz) of **4e**.

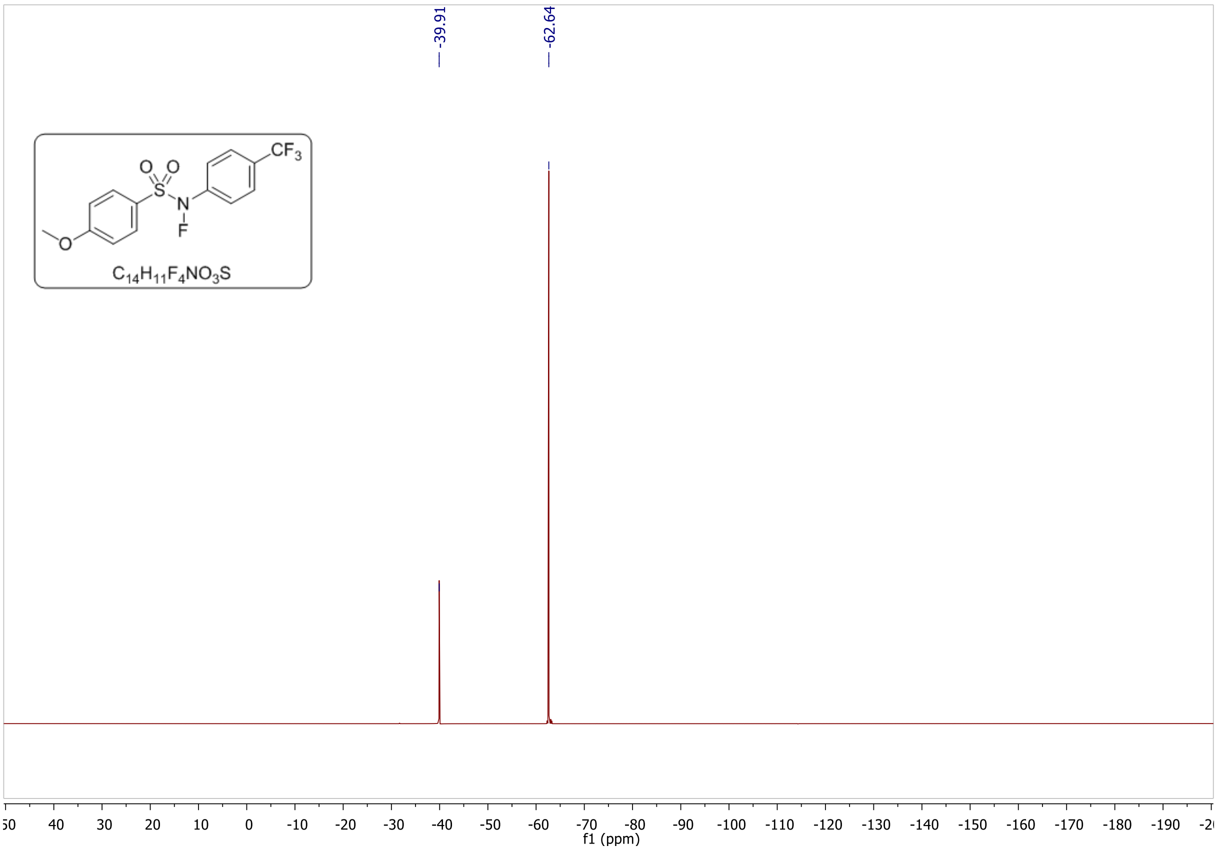

Supplementary Figure 133. ^{19}F-NMR (282 MHz) of **4e**.

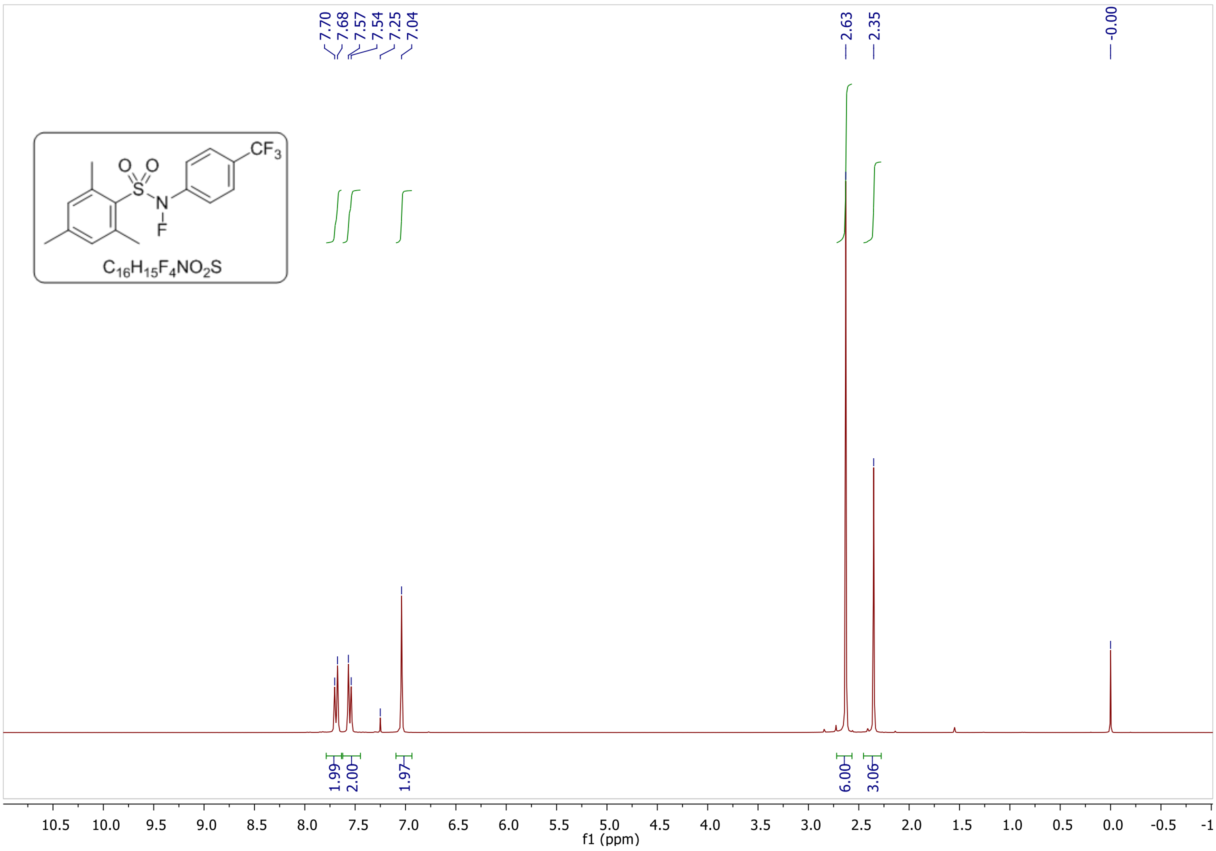

Supplementary Figure 134. ^{1}H-NMR (300 MHz) of **4f**.

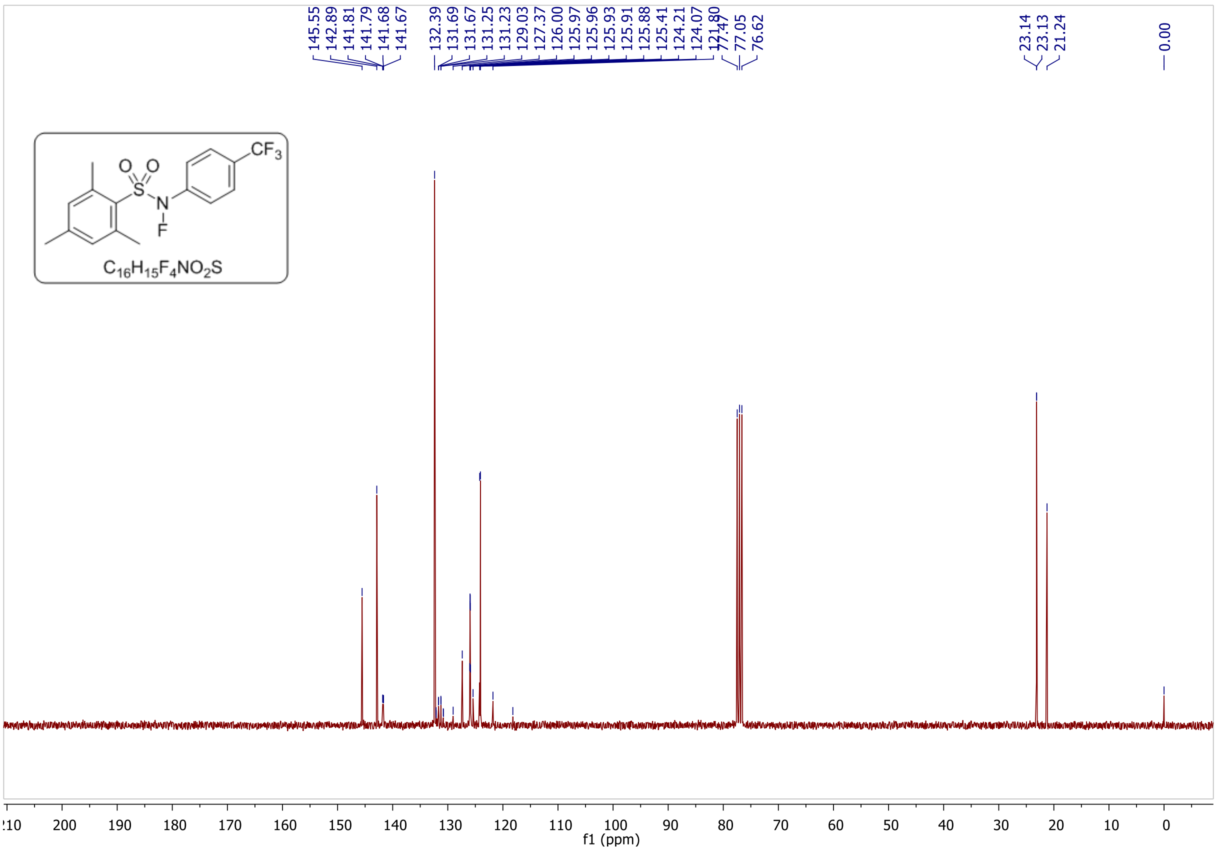

Supplementary Figure 135. ^{13}C-NMR (75 MHz) of **4f**.

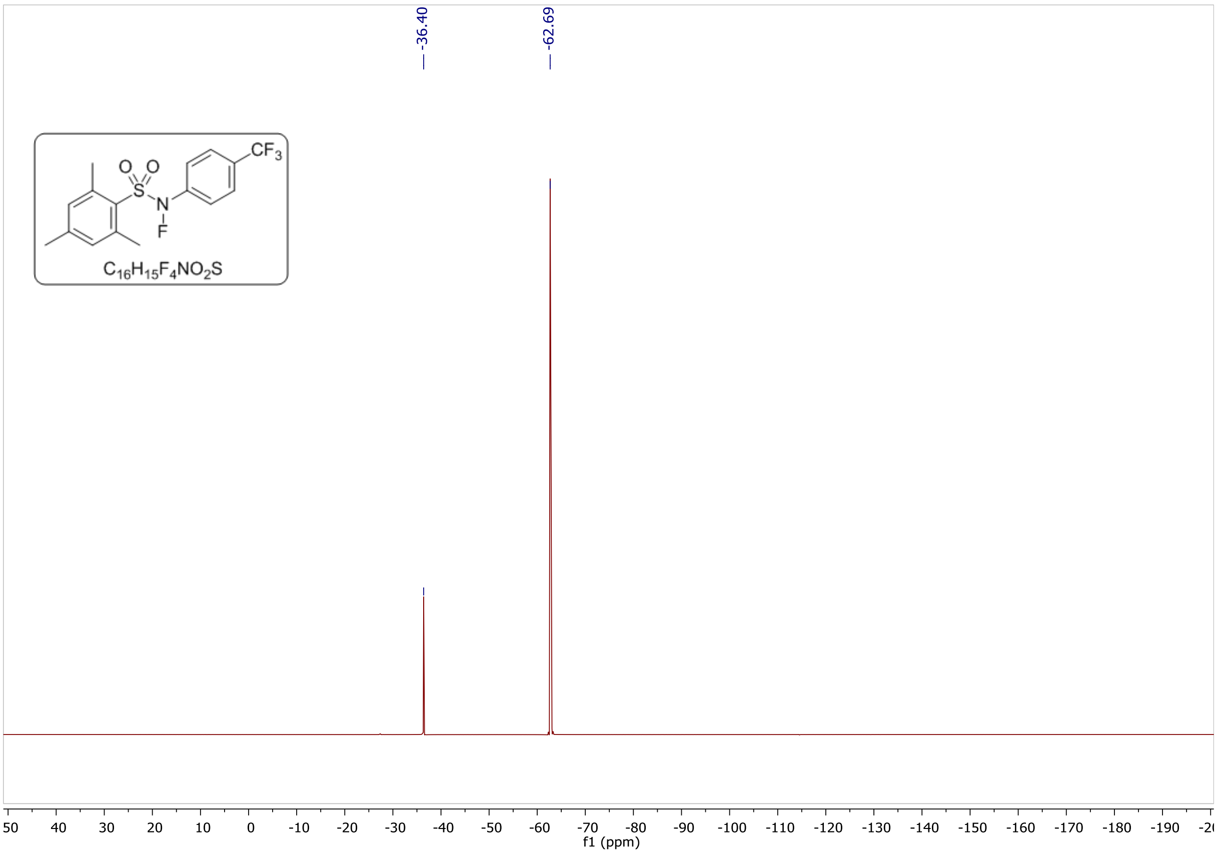

Supplementary Figure 136. ^{19}F-NMR (282 MHz) of **4f**.

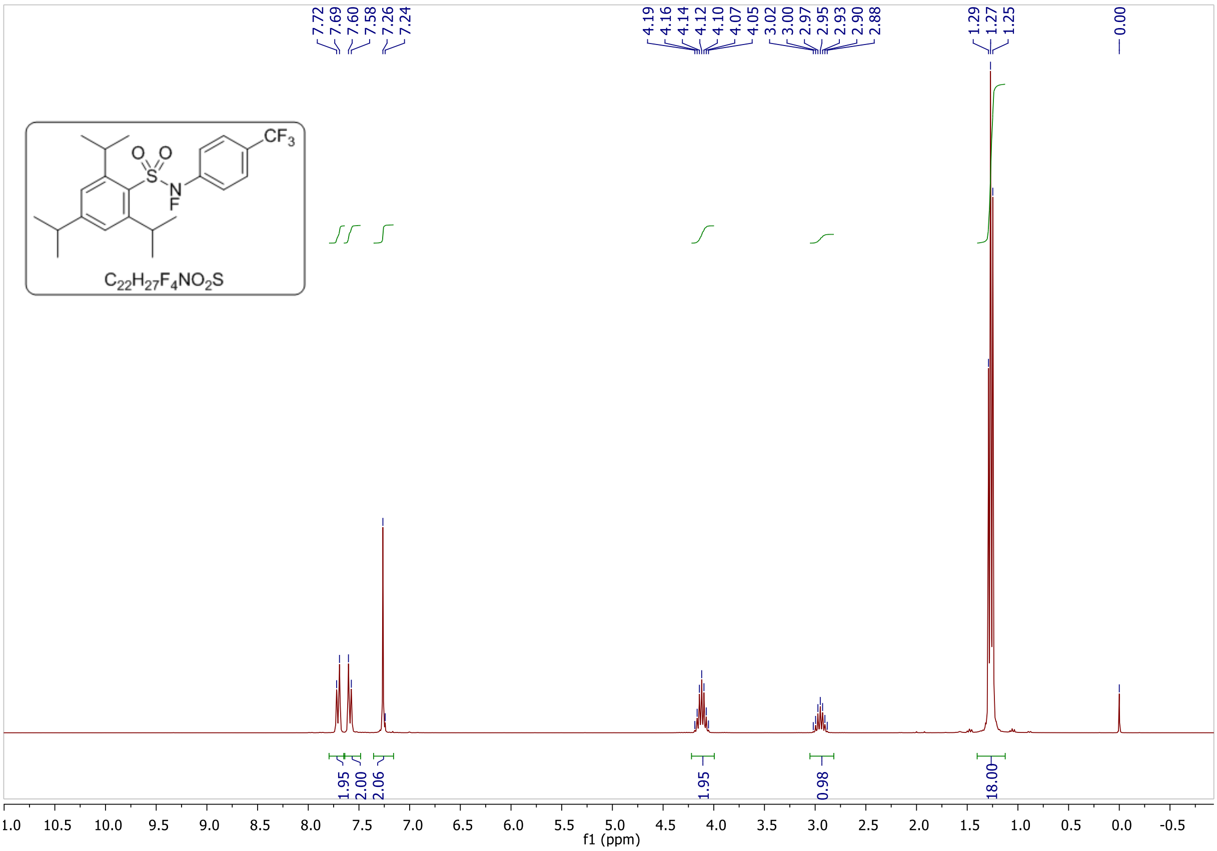

Supplementary Figure 137. ^{1}H-NMR (300 MHz) of **4g**.

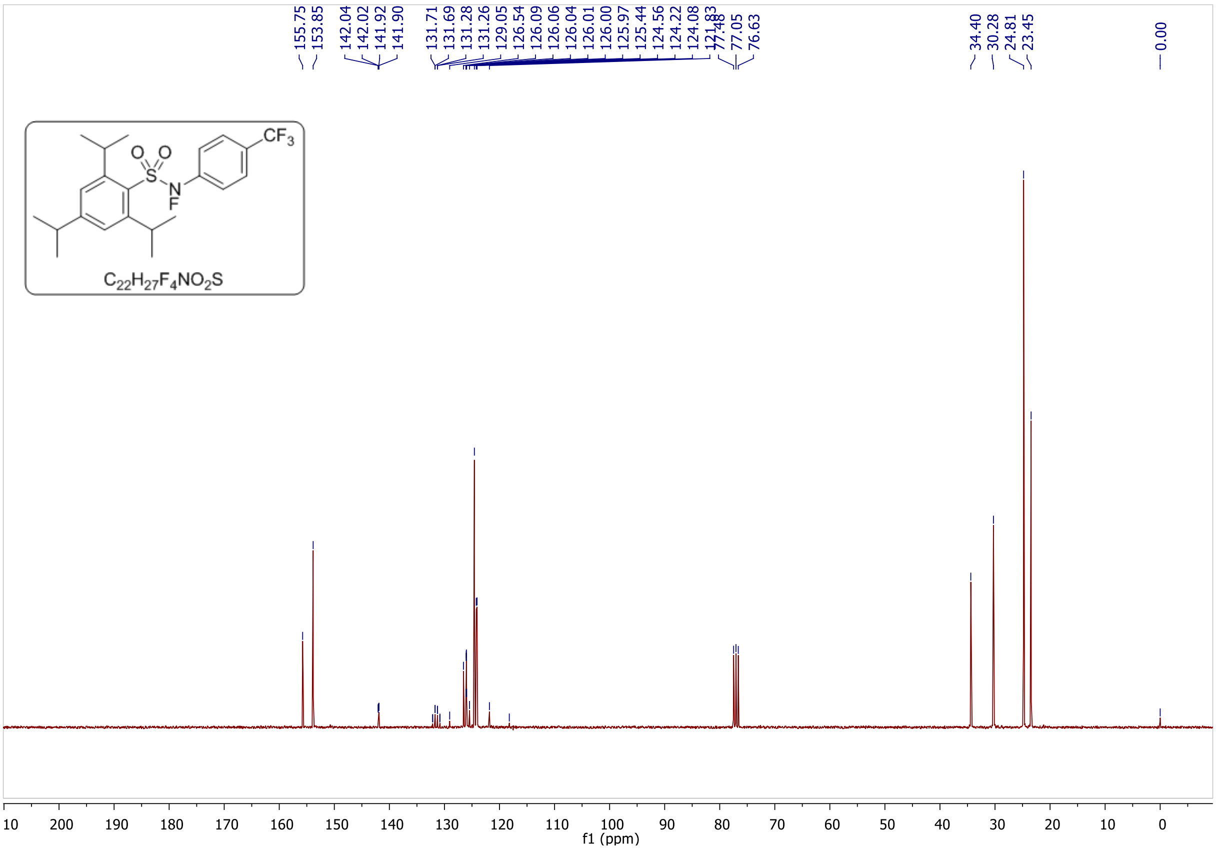

Supplementary Figure 138. ^{13}C-NMR (75 MHz) of **4g**.

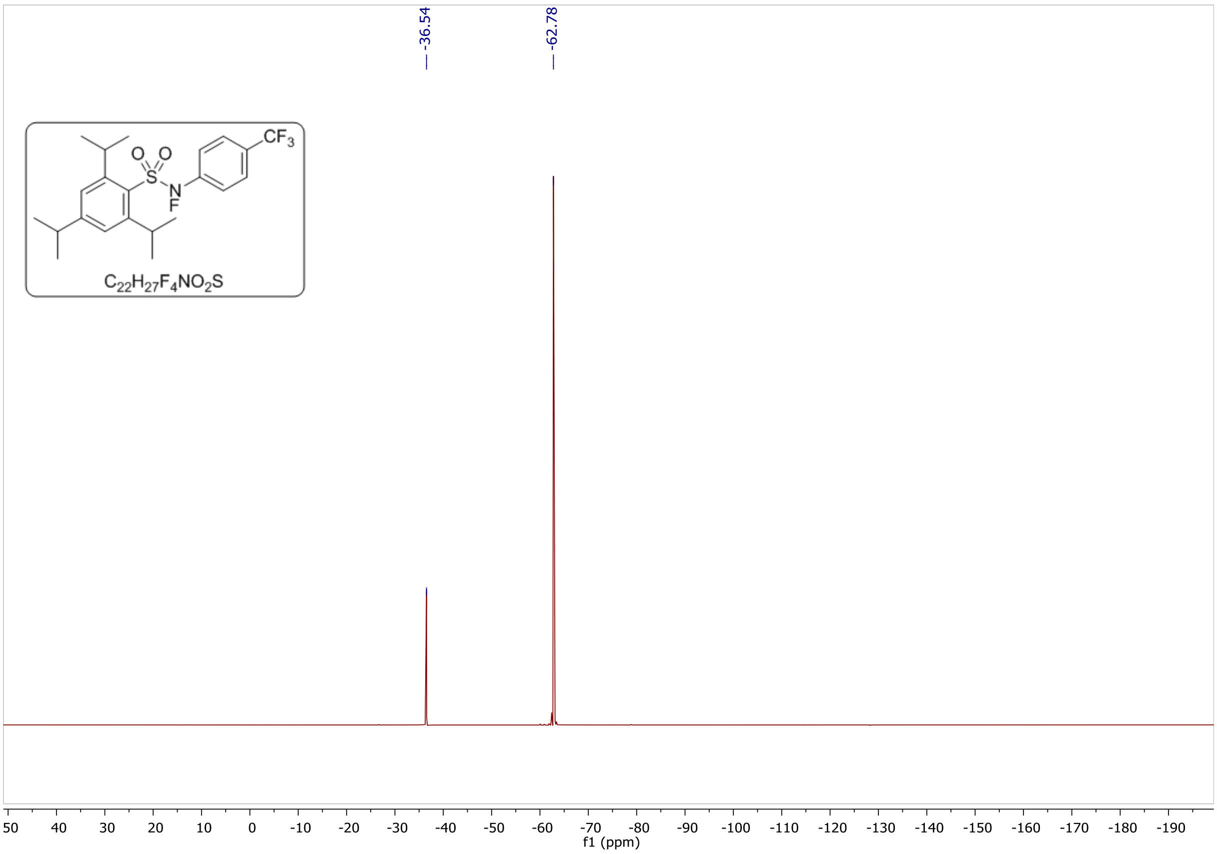

Supplementary Figure 139. ^{19}F-NMR (282 MHz) of **4g**.

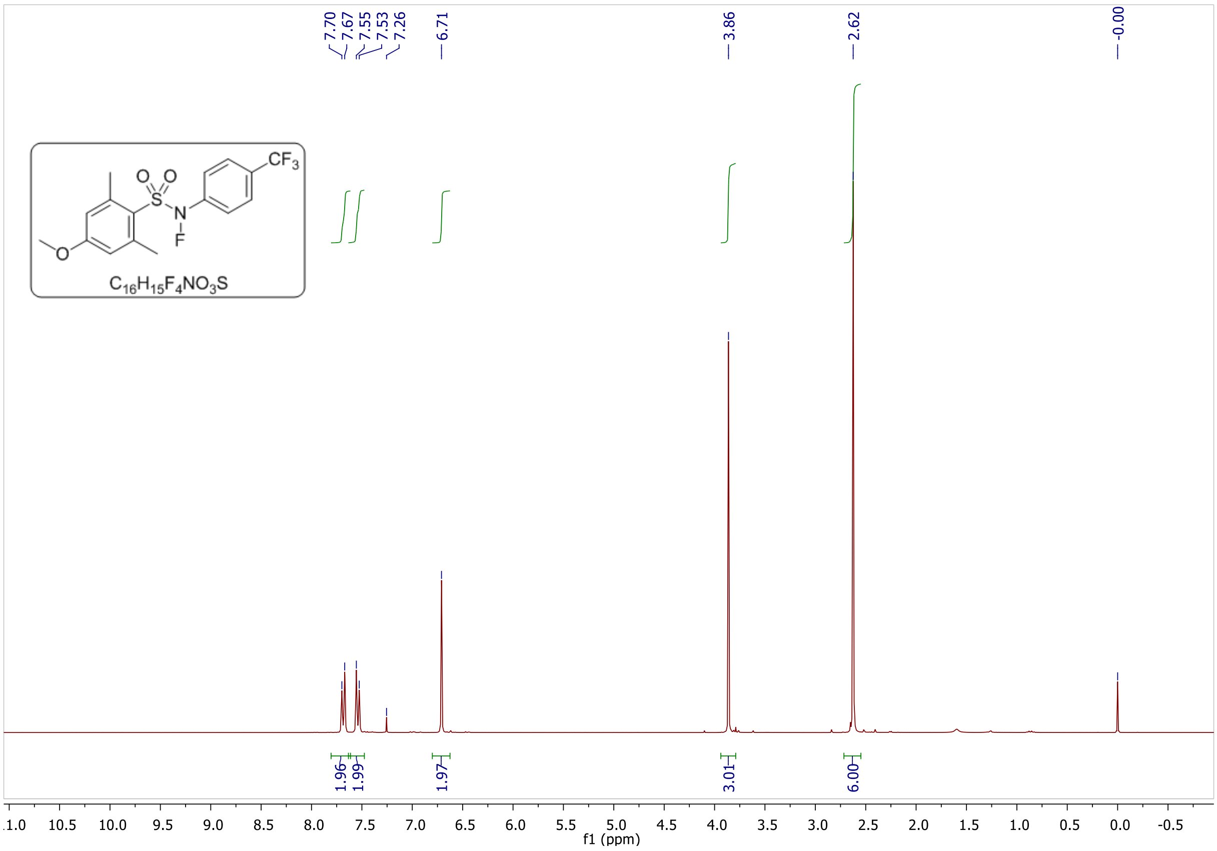

Supplementary Figure 140. ^{1}H-NMR (300 MHz) of **4h**.

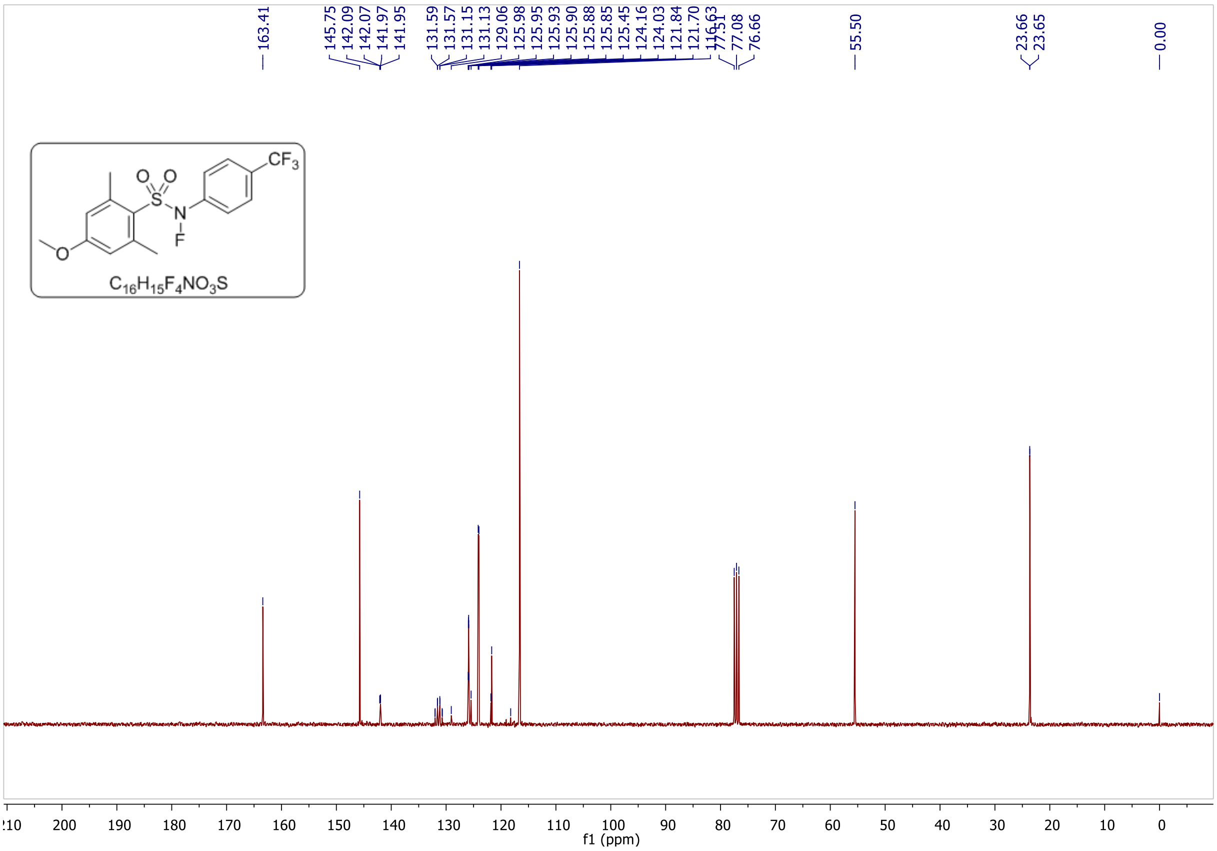

Supplementary Figure 141. ^{13}C-NMR (75 MHz) of **4h**.

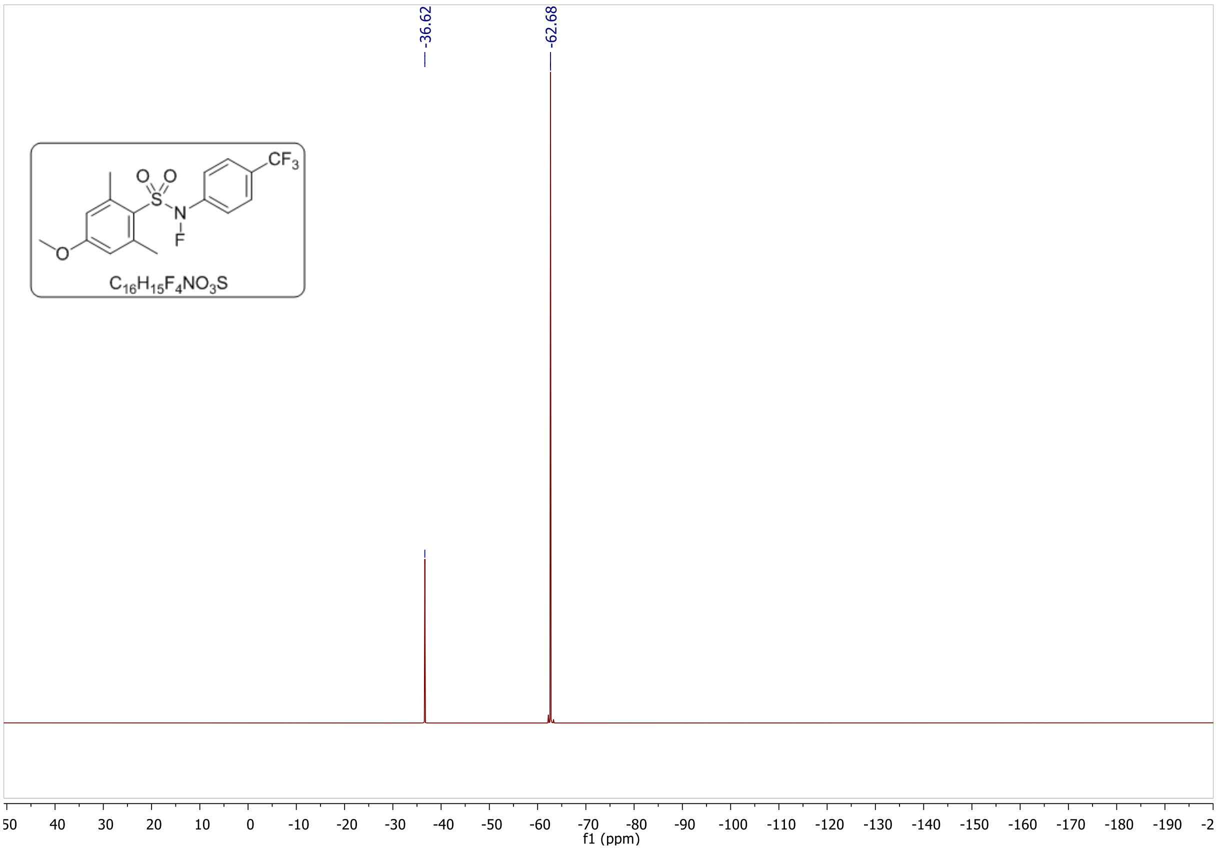

Supplementary Figure 142. ^{19}F-NMR (282 MHz) of **4h**.

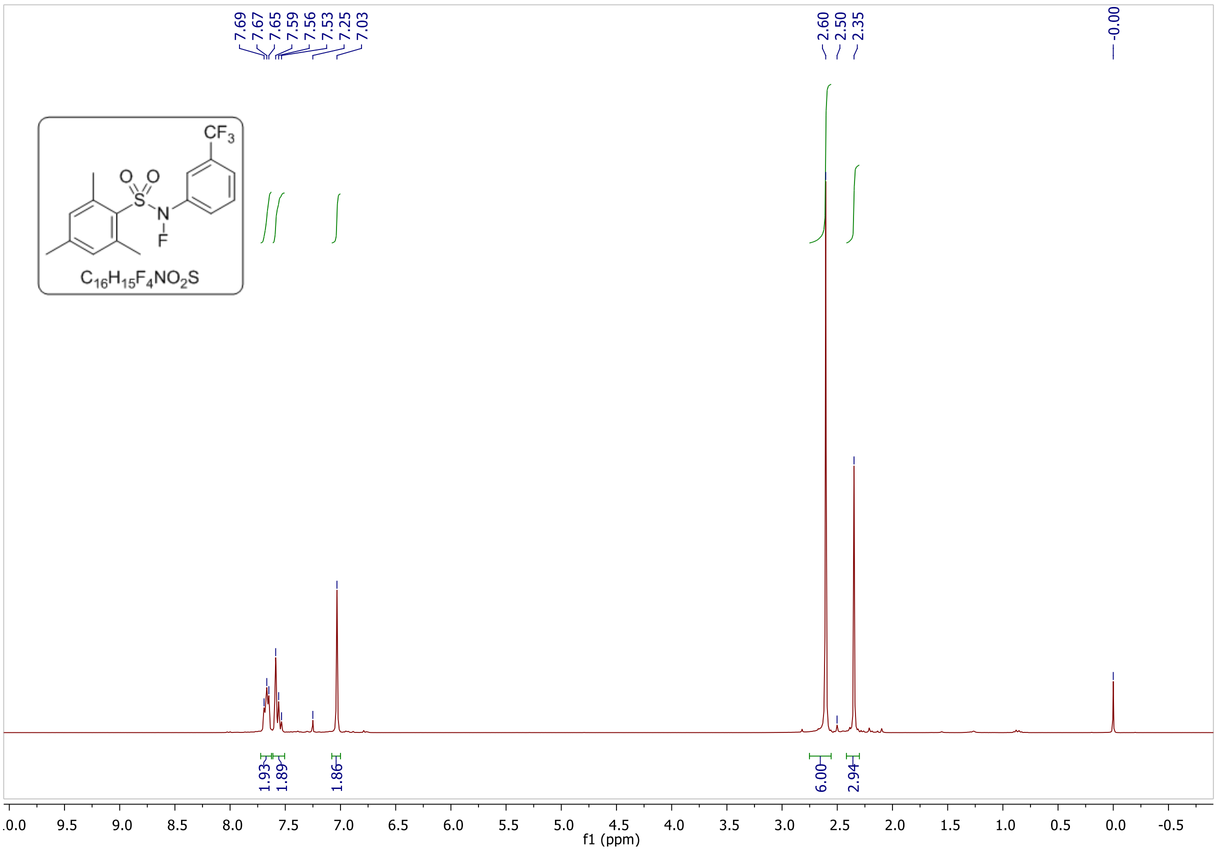

Supplementary Figure 143. ^{1}H-NMR (300 MHz) of **4i**.

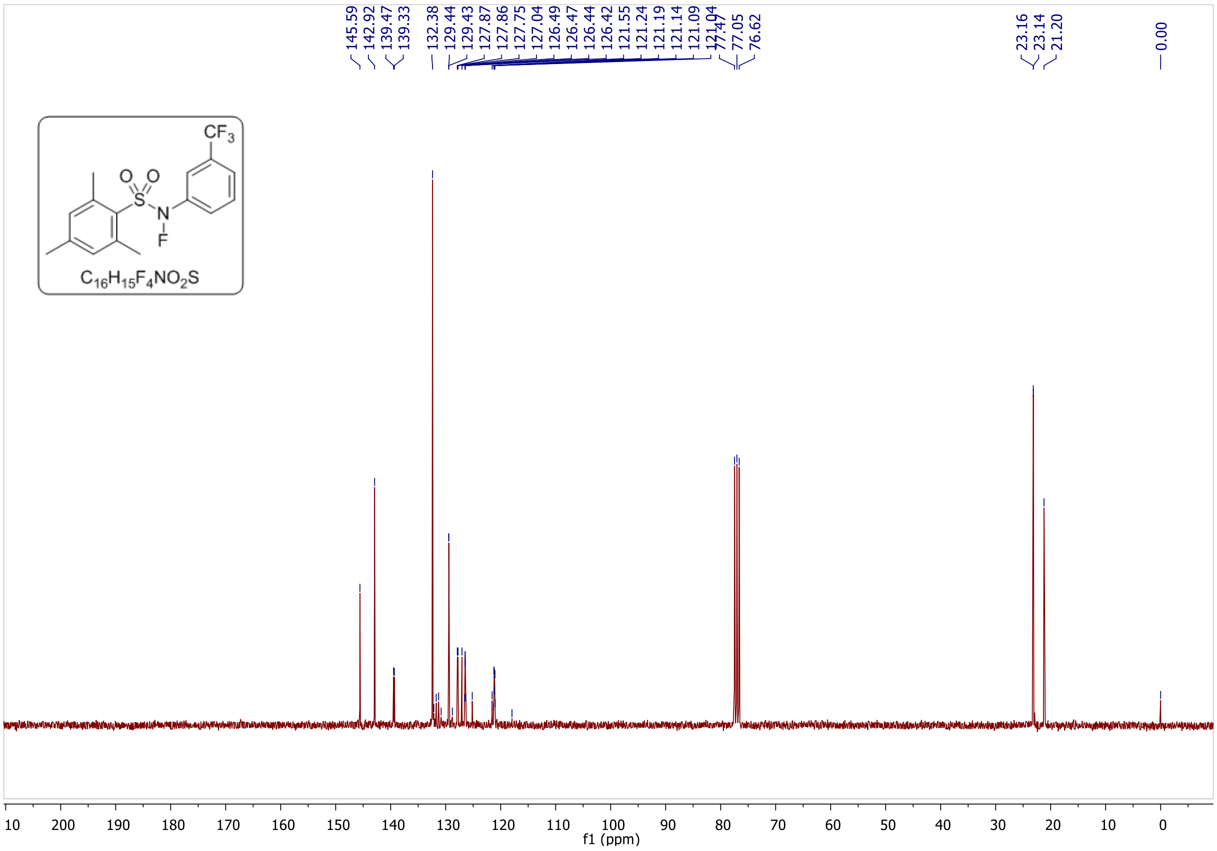

Supplementary Figure 144. ^{13}C-NMR (75 MHz) of **4i**.

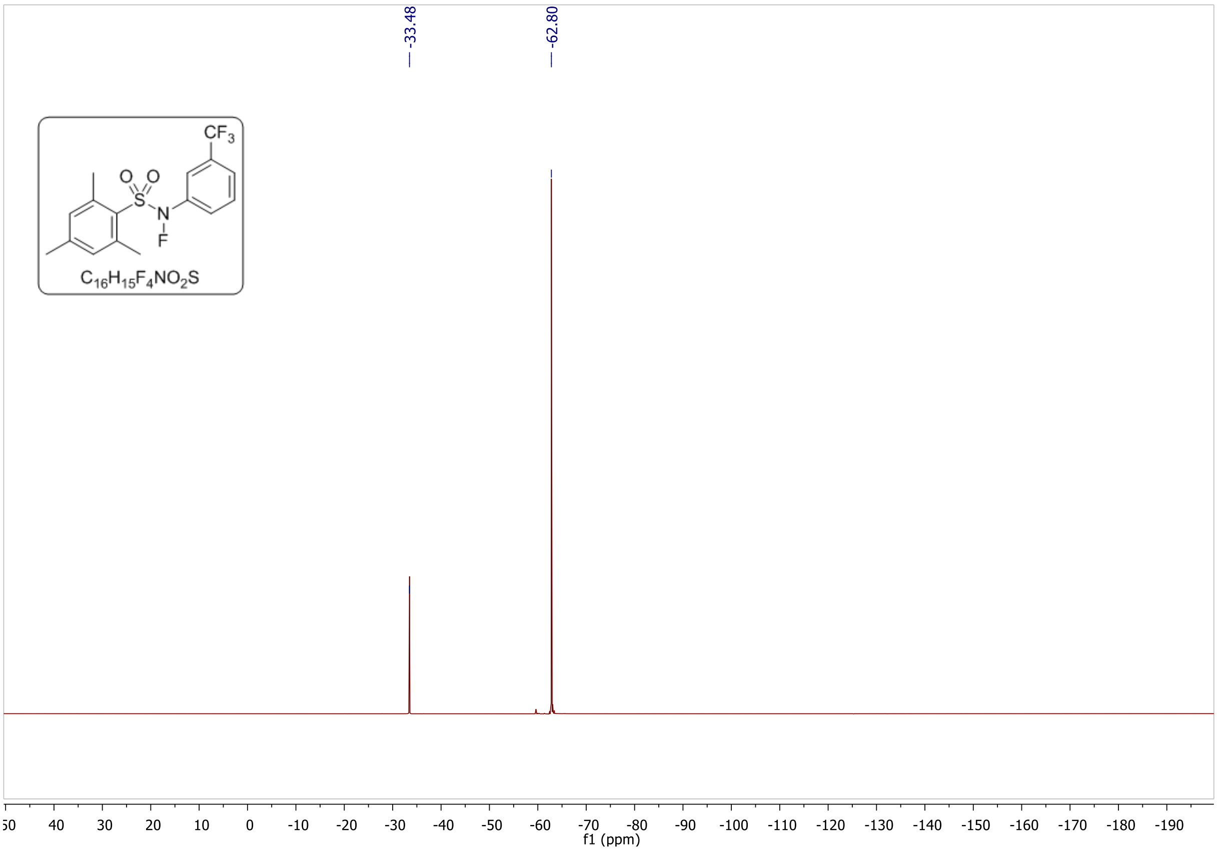

Supplementary Figure 145. ^{19}F-NMR (282 MHz) of **4i**.

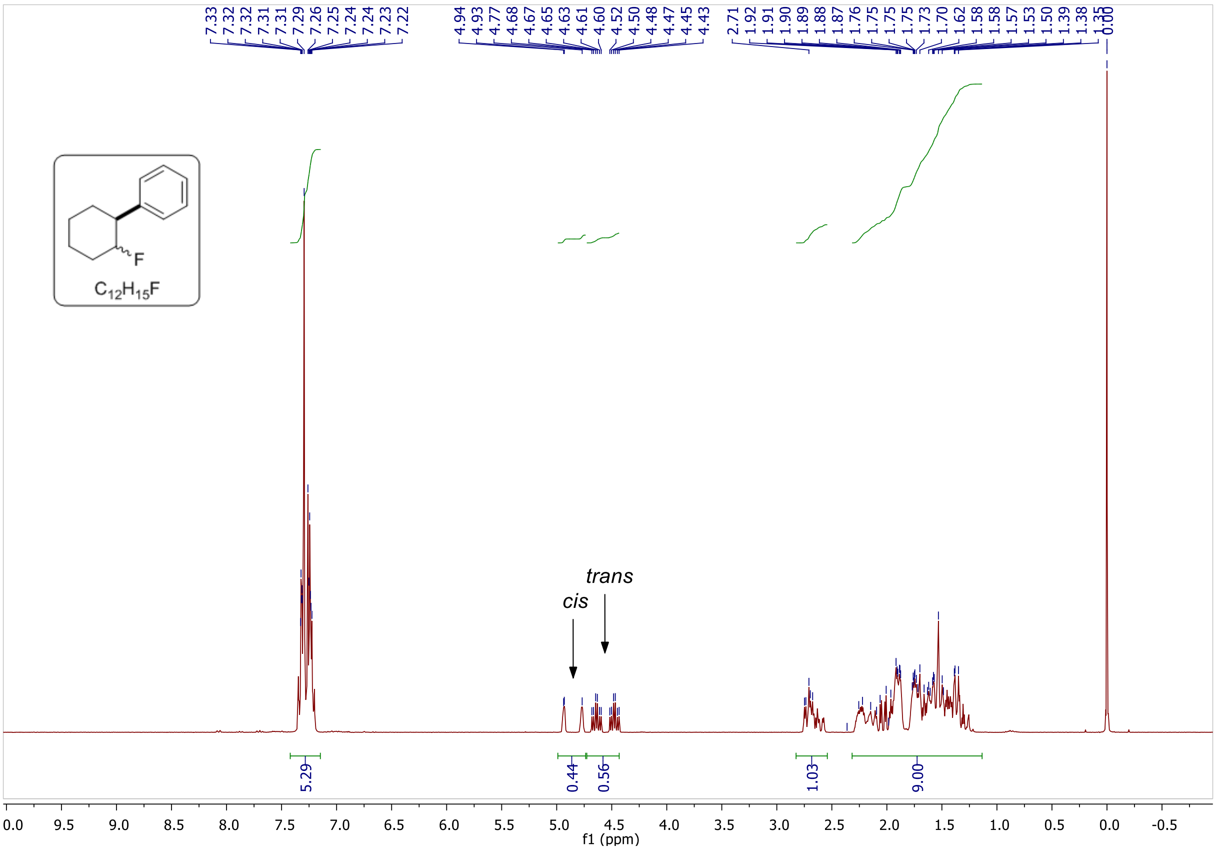

Supplementary Figure 146. ^{1}H-NMR (300 MHz) of **2a**.

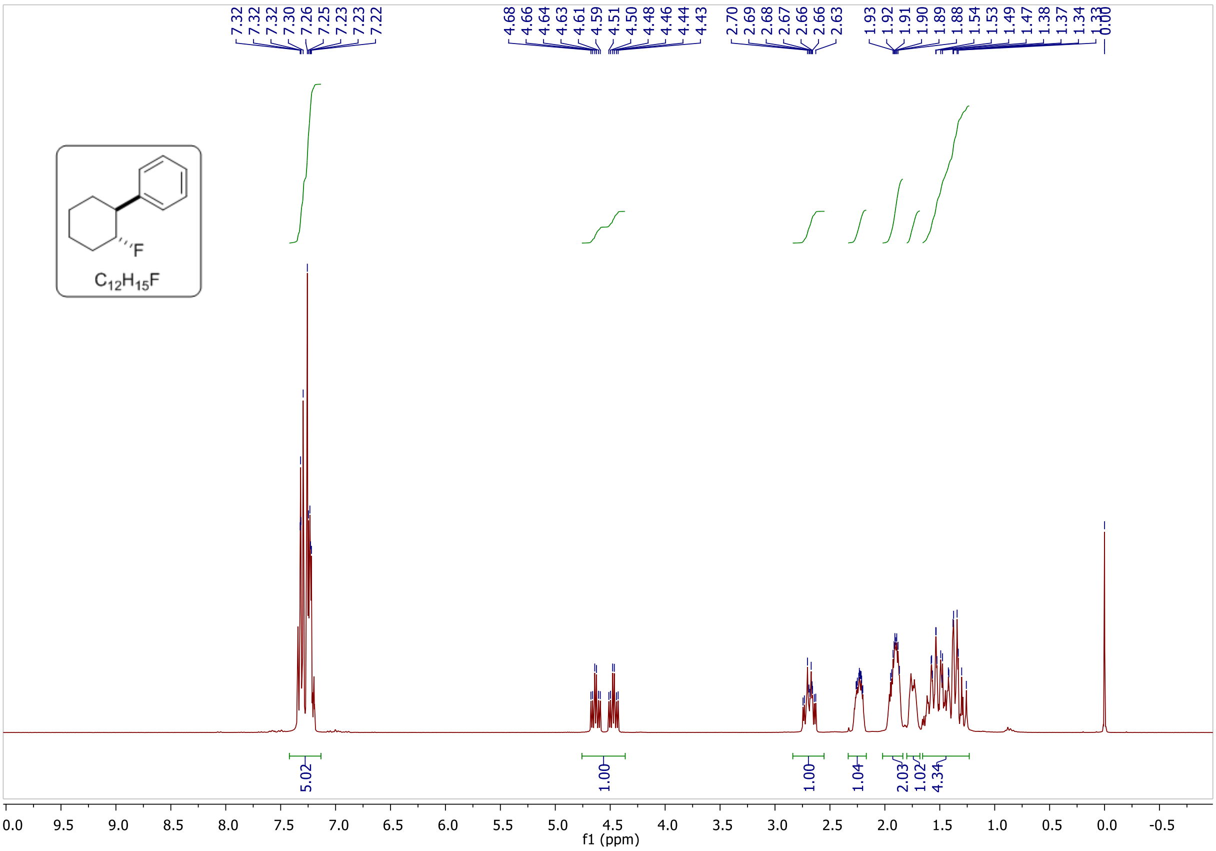

Supplementary Figure 147. ^{1}H-NMR (300 MHz) of *trans*-**2a**.

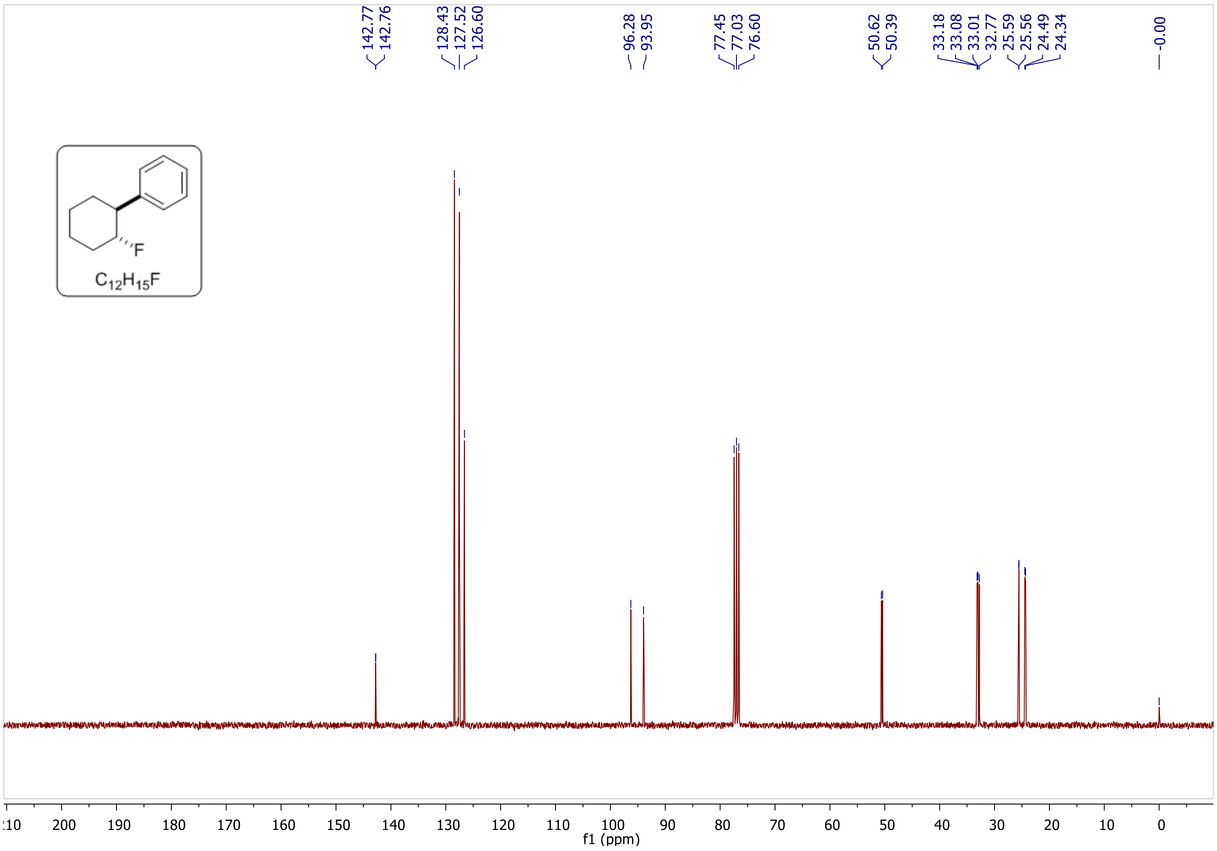

Supplementary Figure 148. ^{13}C-NMR (75 MHz) of *trans*-**2a**.

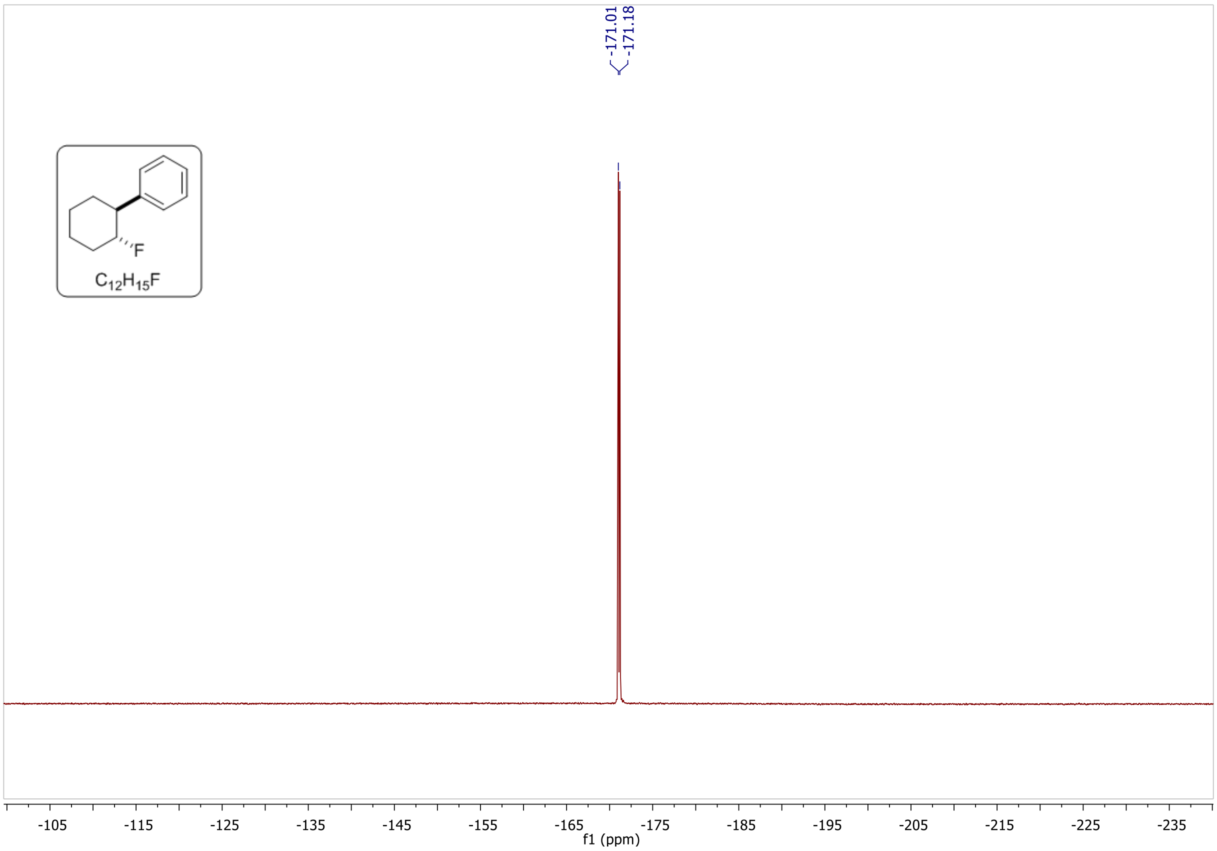

Supplementary Figure 149. ^{19}F-NMR (282 MHz) of *trans*-**2a**.

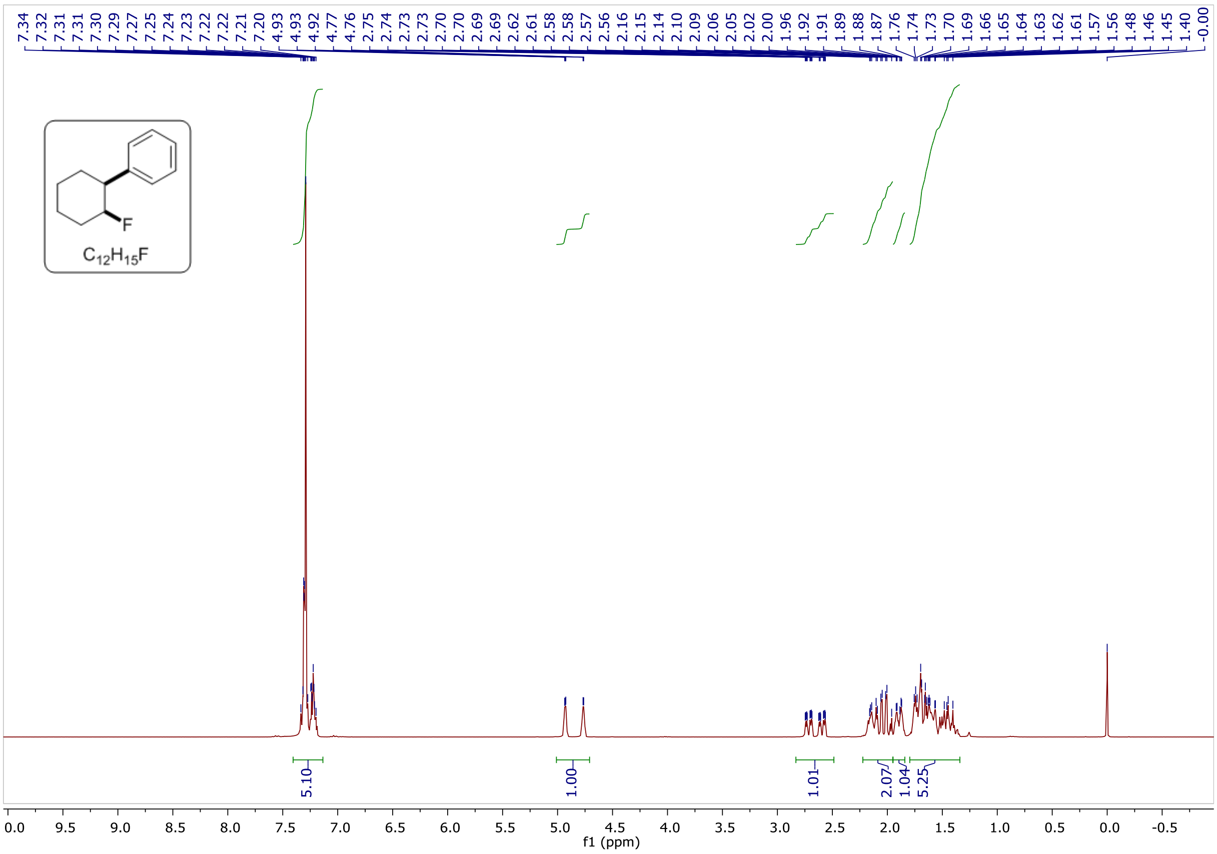

Supplementary Figure 150. ^{1}H-NMR (300 MHz) of *cis*-**2a**.

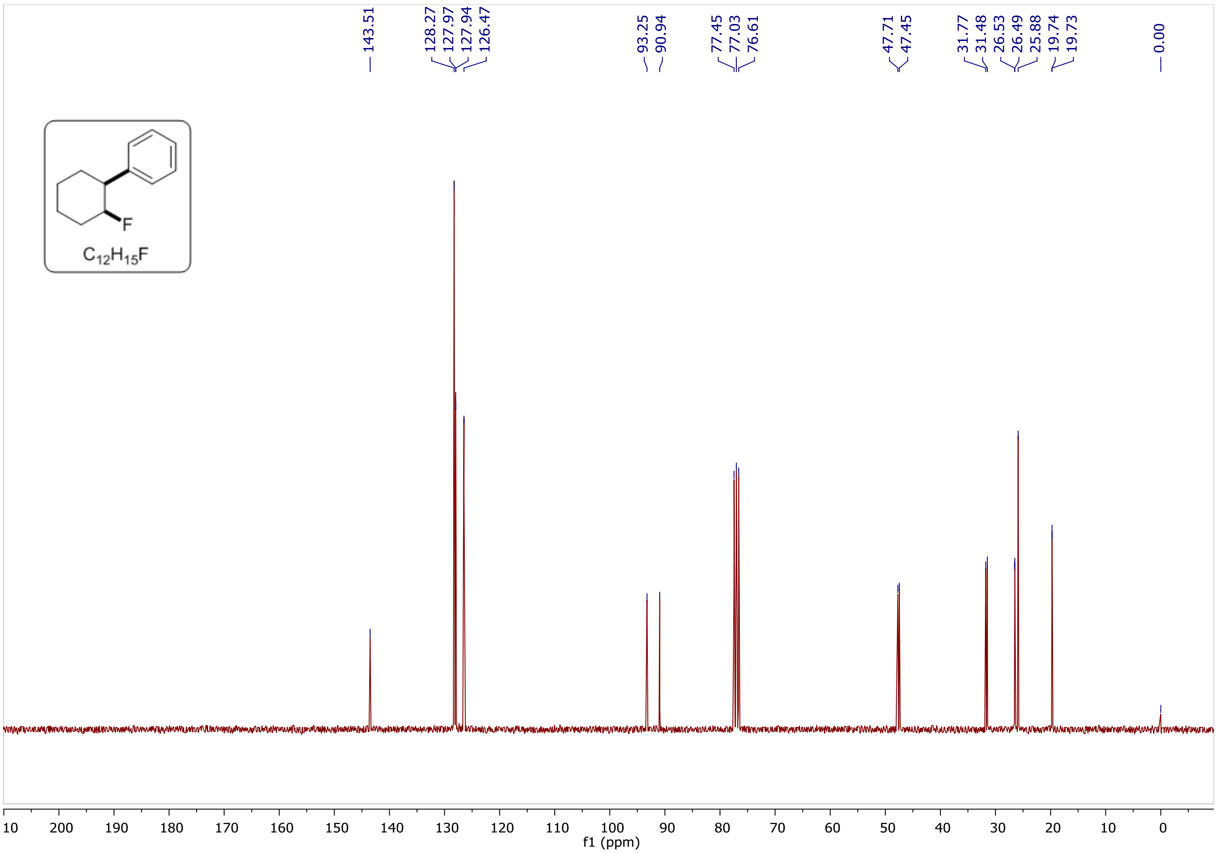

Supplementary Figure 151. ^{13}C-NMR (75 MHz) of *cis*-**2a**.

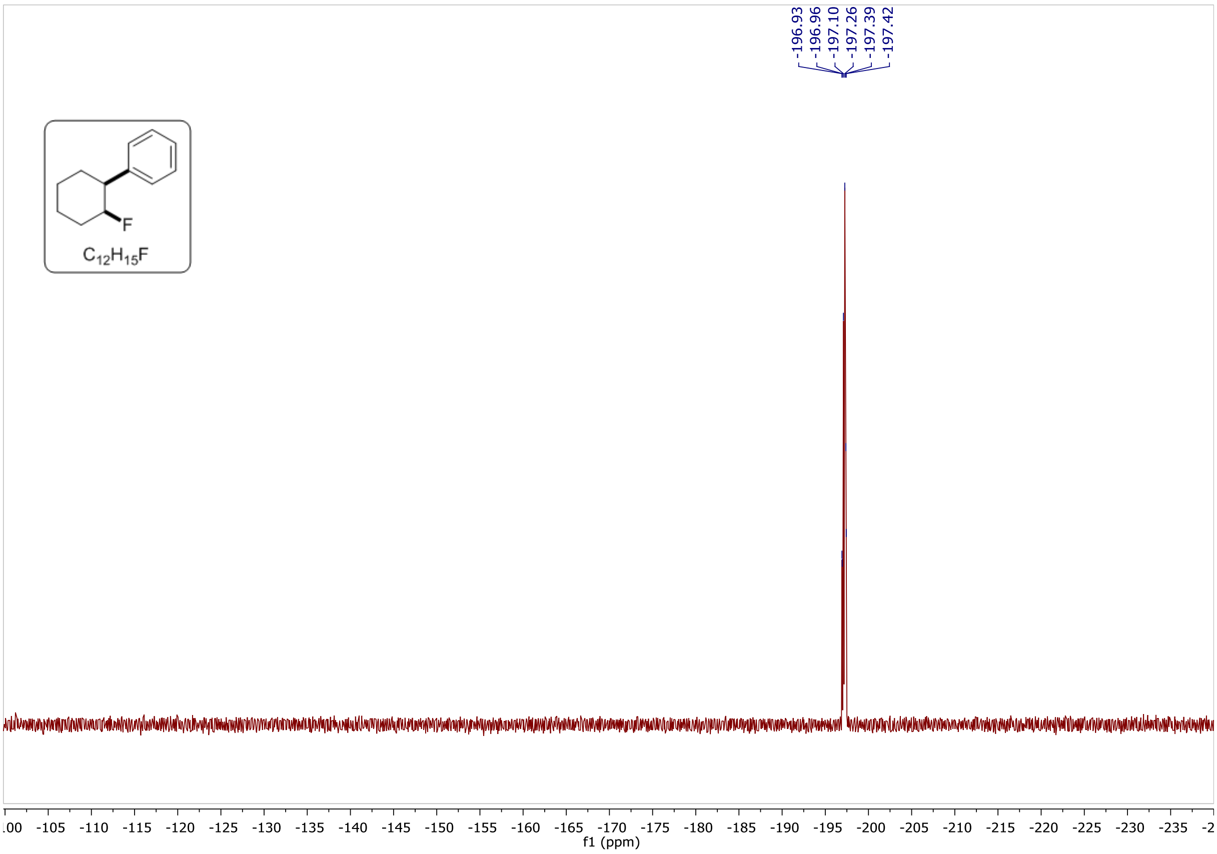

Supplementary Figure 152. ^{19}F-NMR (282 MHz) of *cis*-**2a**.

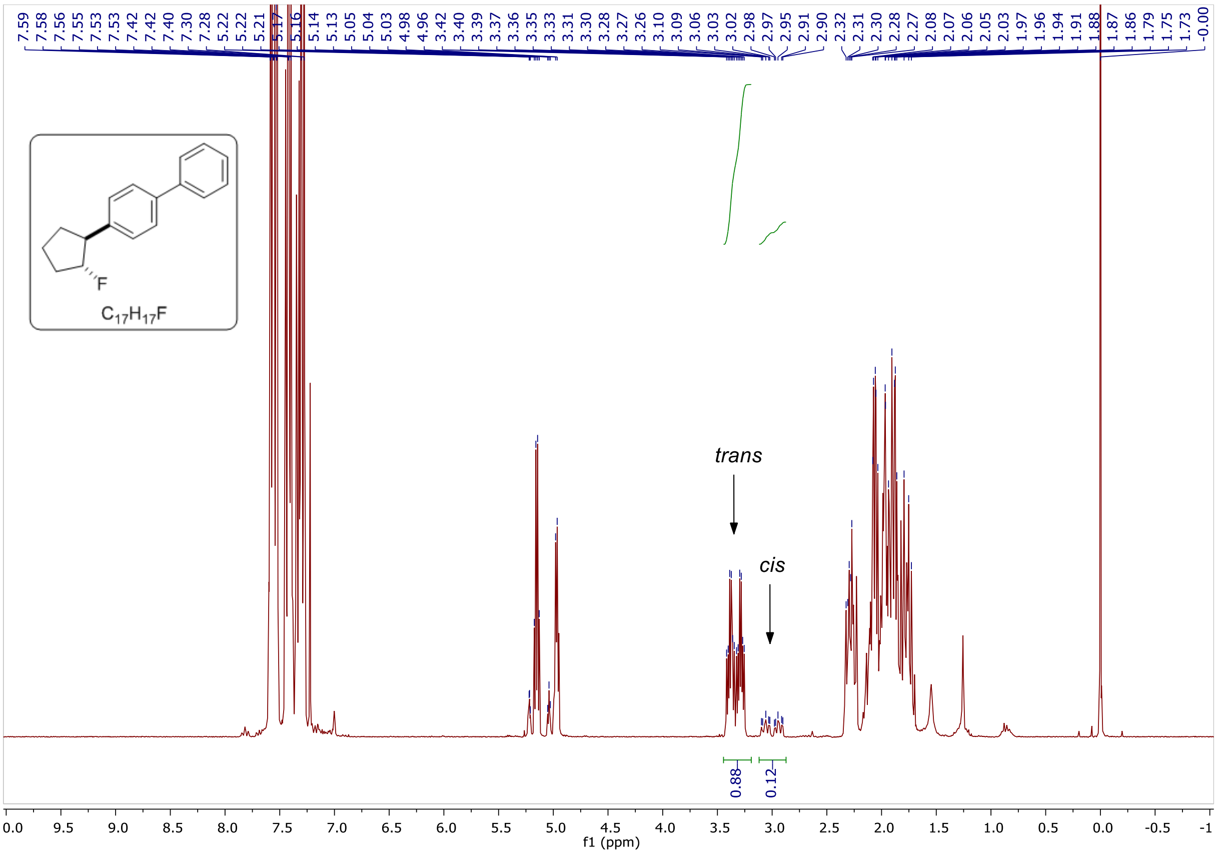

Supplementary Figure 153. ^{1}H-NMR (300 MHz) of **2b**.

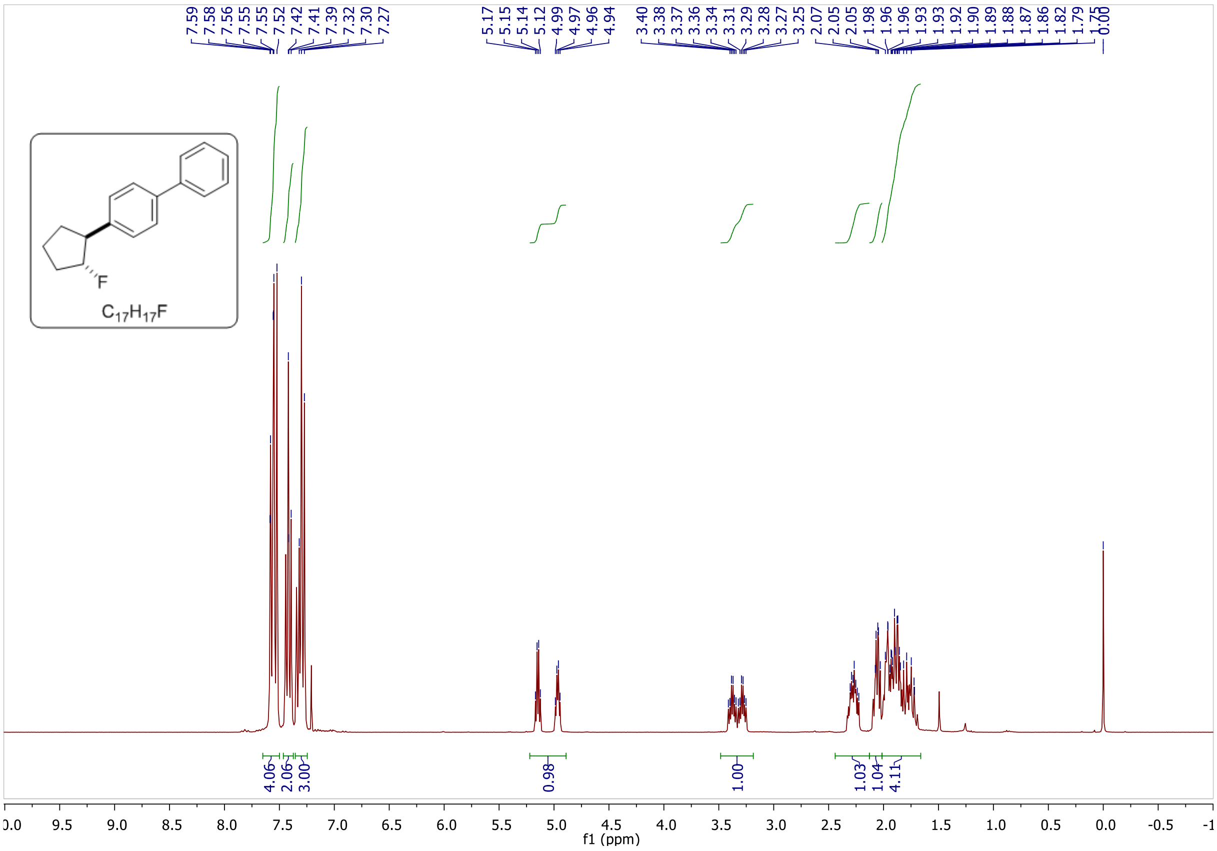

Supplementary Figure 154. ^{1}H-NMR (300 MHz) of *trans*-**2b**.

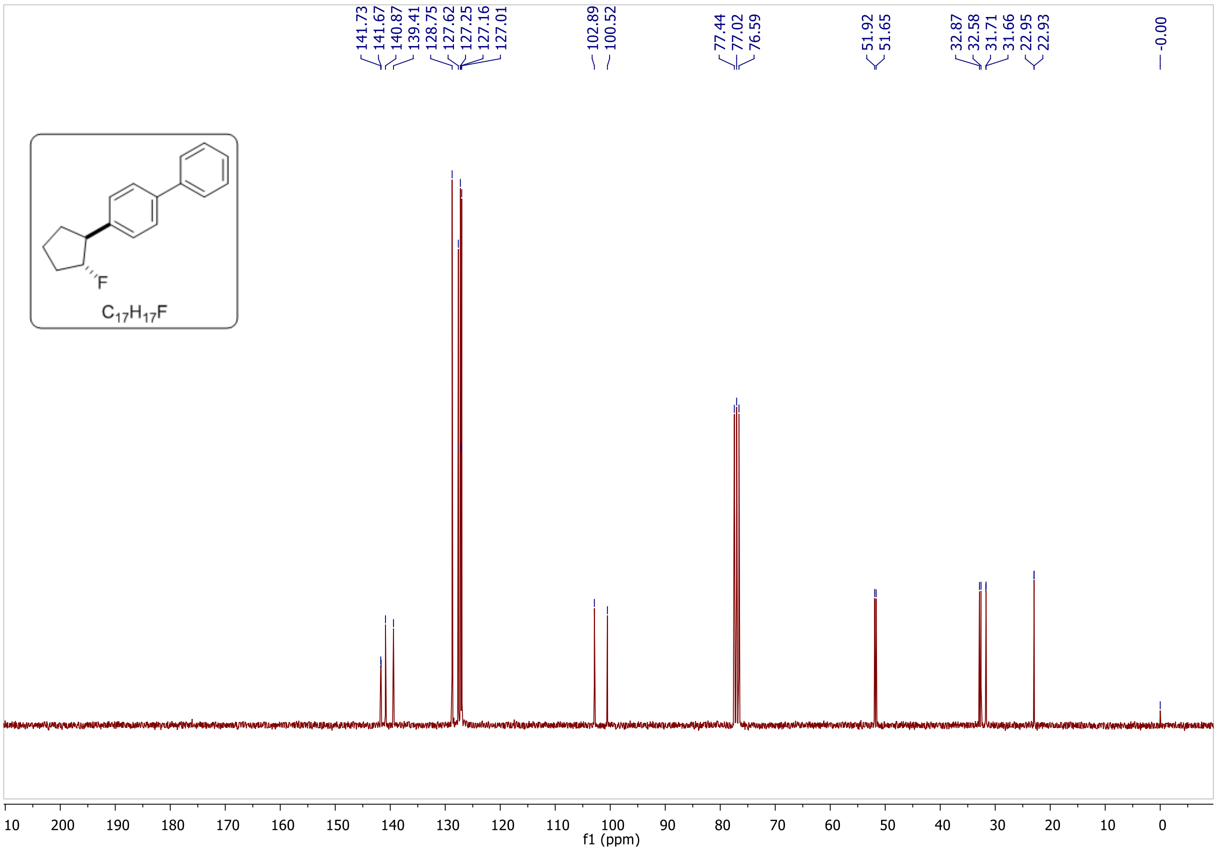

Supplementary Figure 155. ^{13}C-NMR (75 MHz) of *trans*-**2b**.

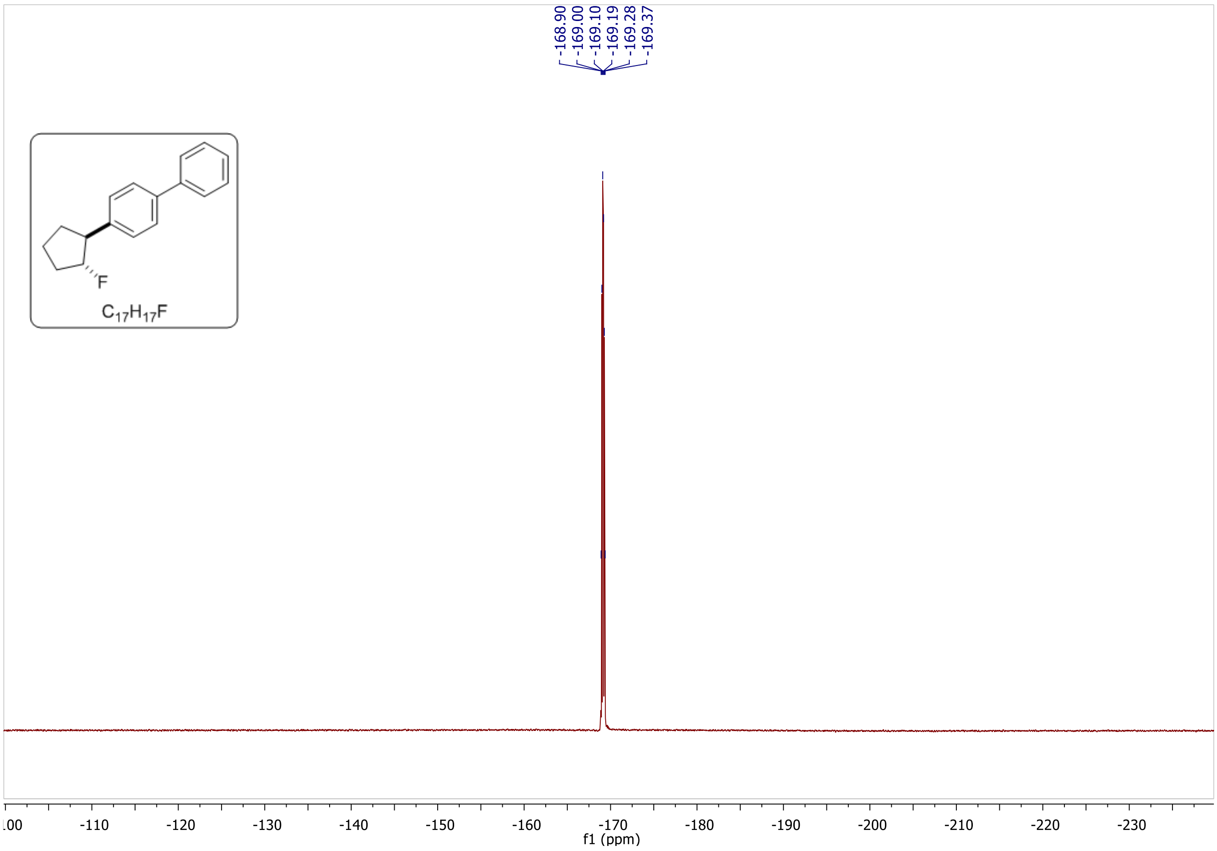

Supplementary Figure 156. ^{19}F-NMR (282 MHz) of *trans*-**2b**.

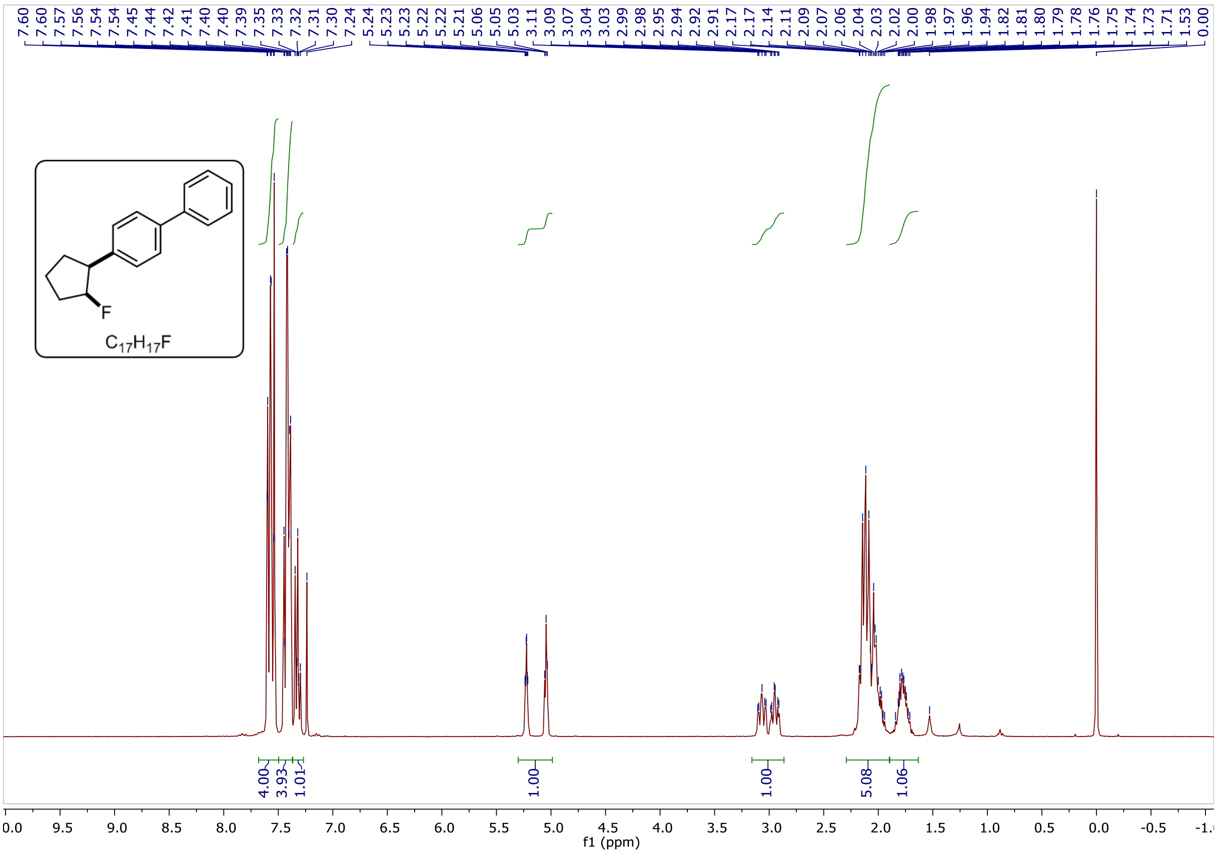

Supplementary Figure 157. ^{1}H-NMR (300 MHz) of *cis*-**2b**.

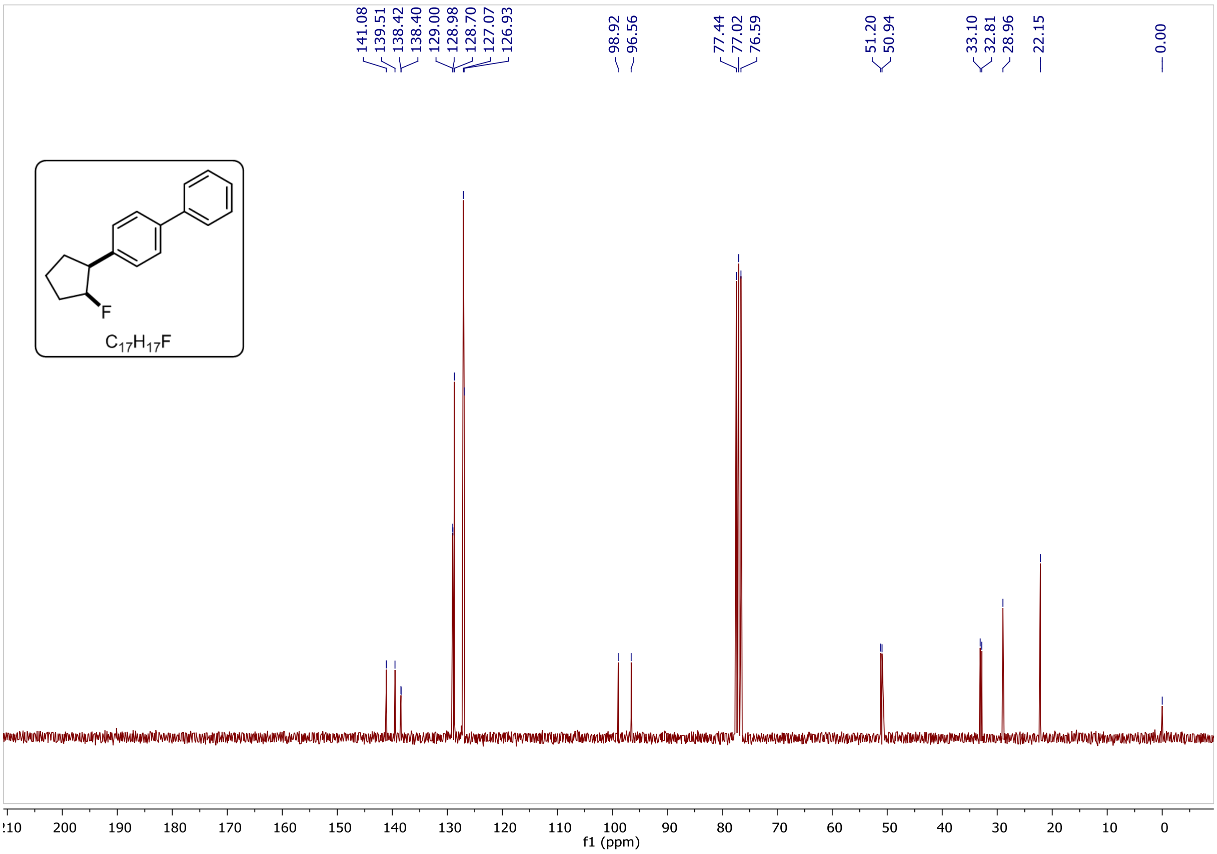

Supplementary Figure 158. ^{13}C-NMR (75 MHz) of *cis*-**2b**.

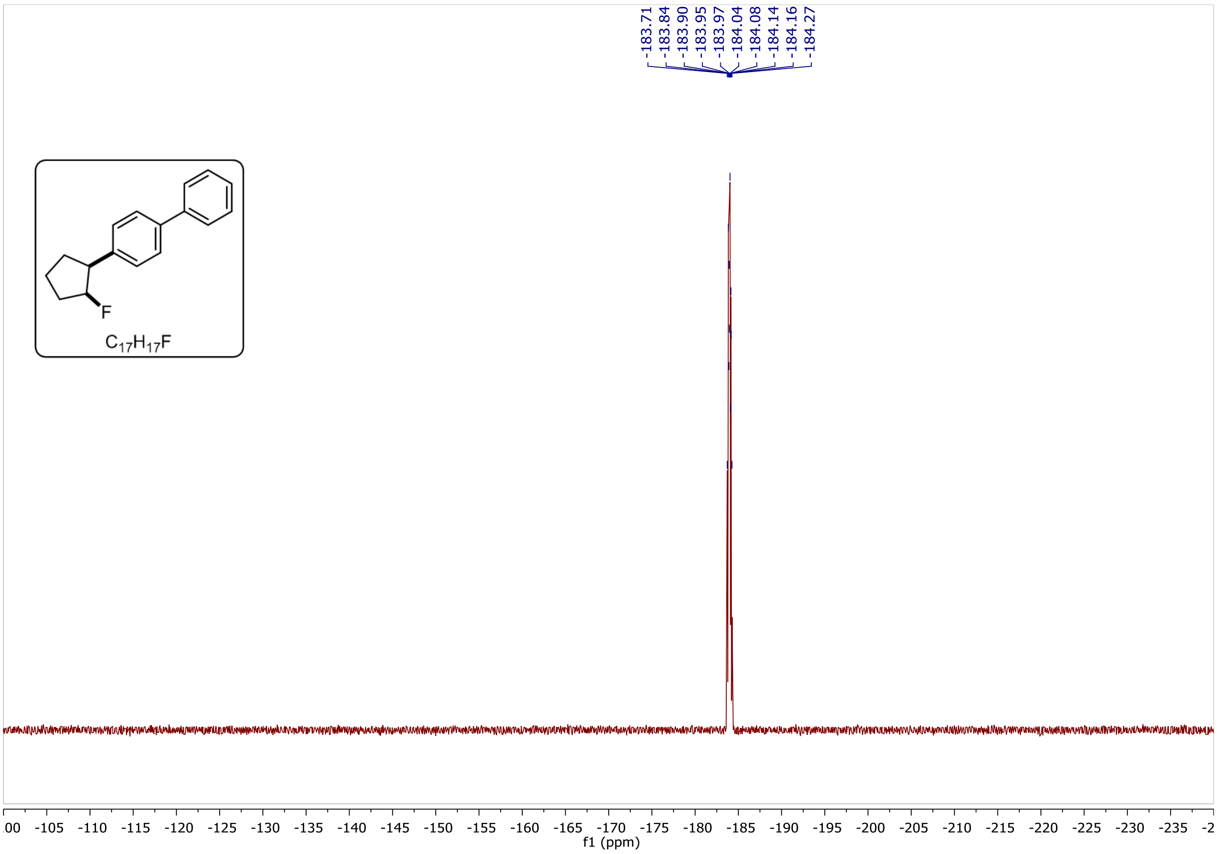

Supplementary Figure 159. ^{19}F-NMR (282 MHz) of *cis*-**2b**.

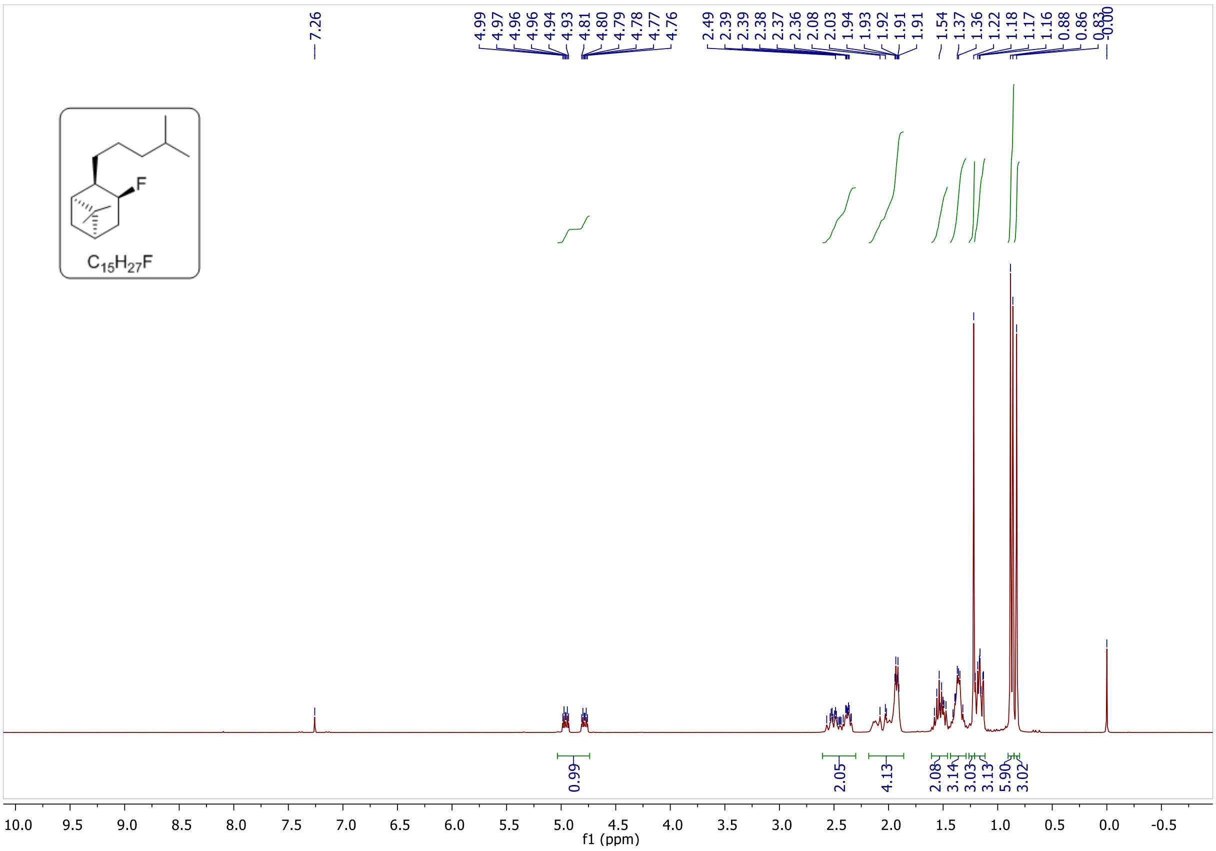

Supplementary Figure 160. ^{1}H-NMR (300 MHz) of **2c**.

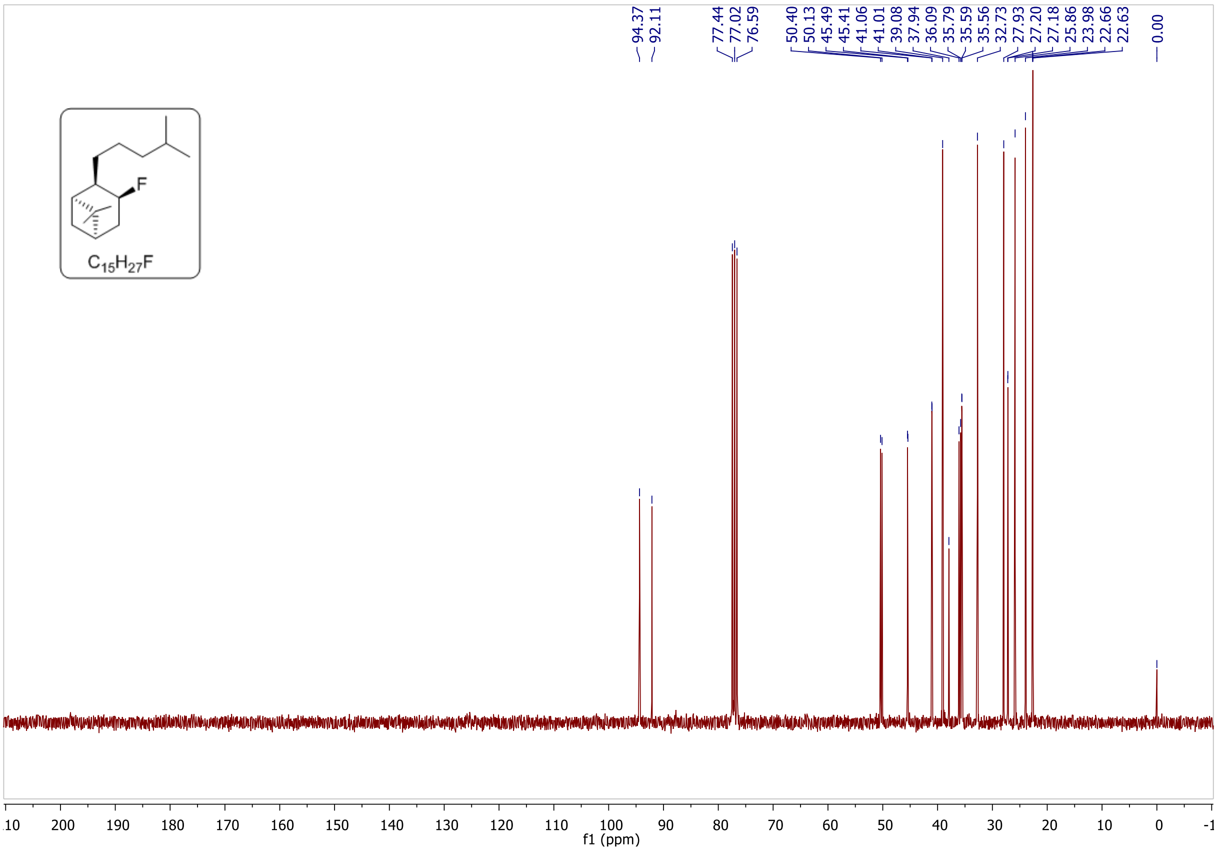

Supplementary Figure 161. ^{13}C-NMR (75 MHz) of **2c**.

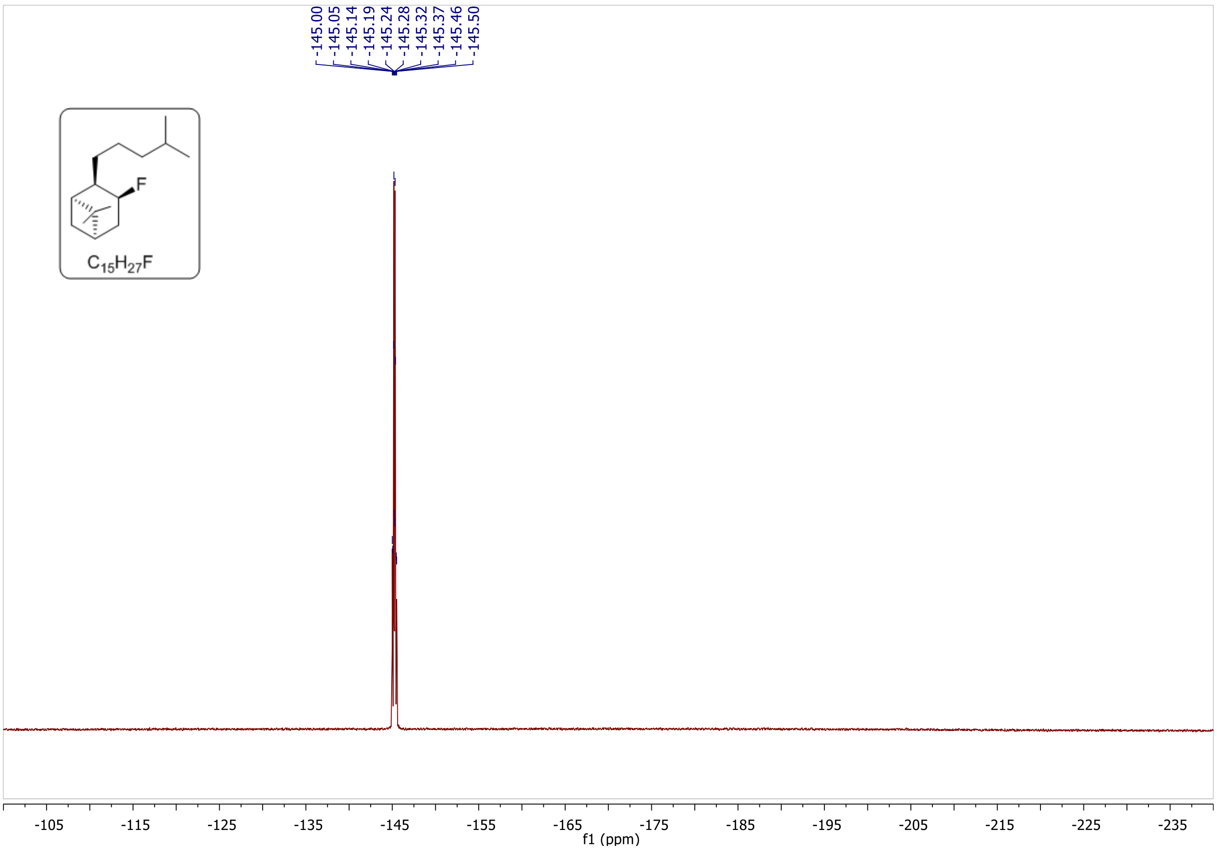

Supplementary Figure 162. ^{19}F-NMR (282 MHz) of **2c**.

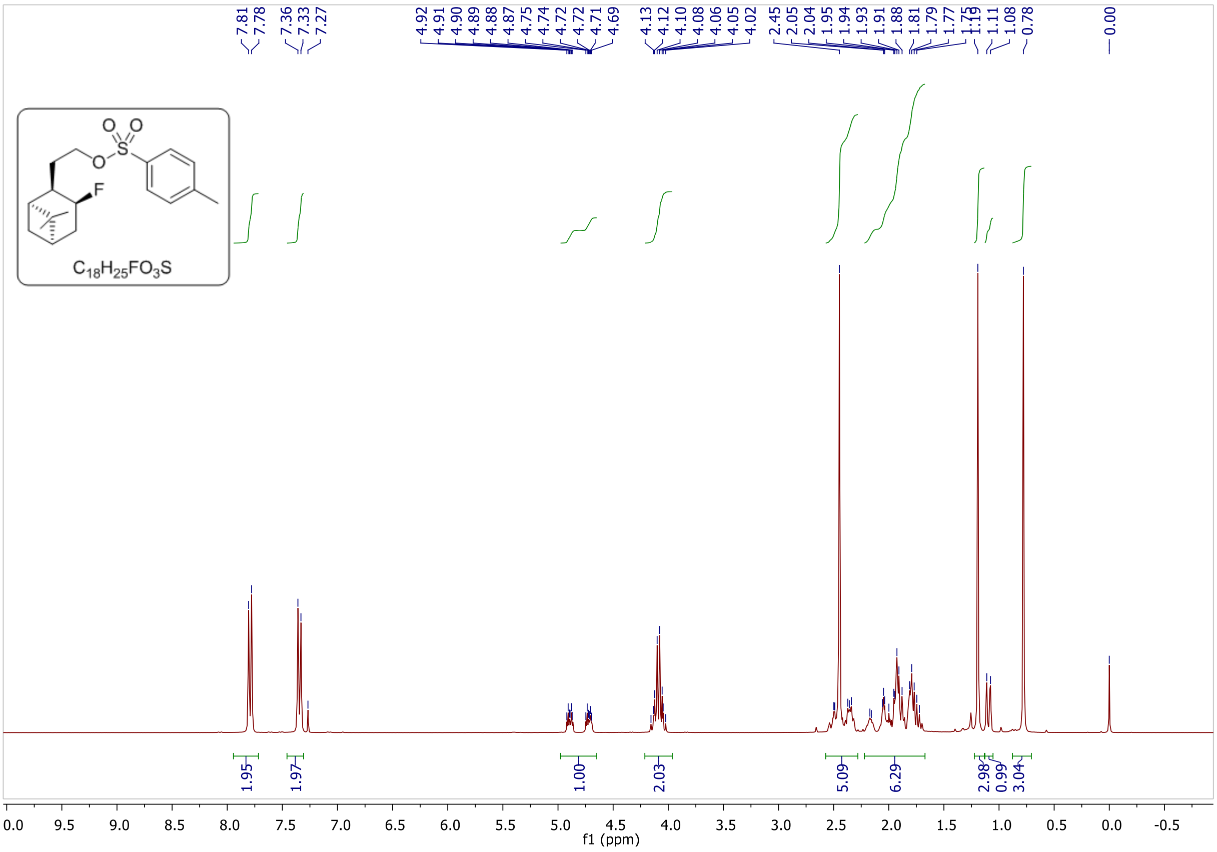

Supplementary Figure 163. ^{1}H-NMR (300 MHz) of **2d**.

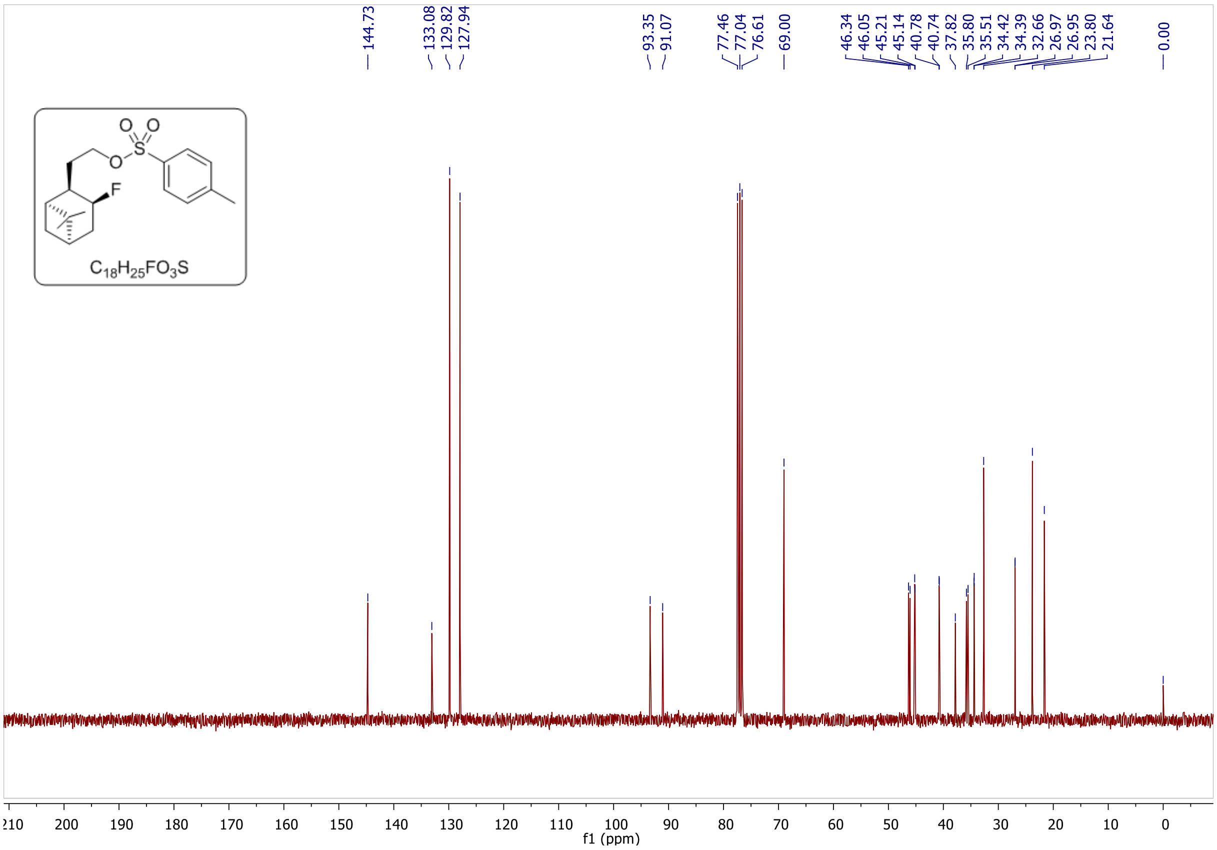

Supplementary Figure 164. ^{13}C-NMR (75 MHz) of **2d**.

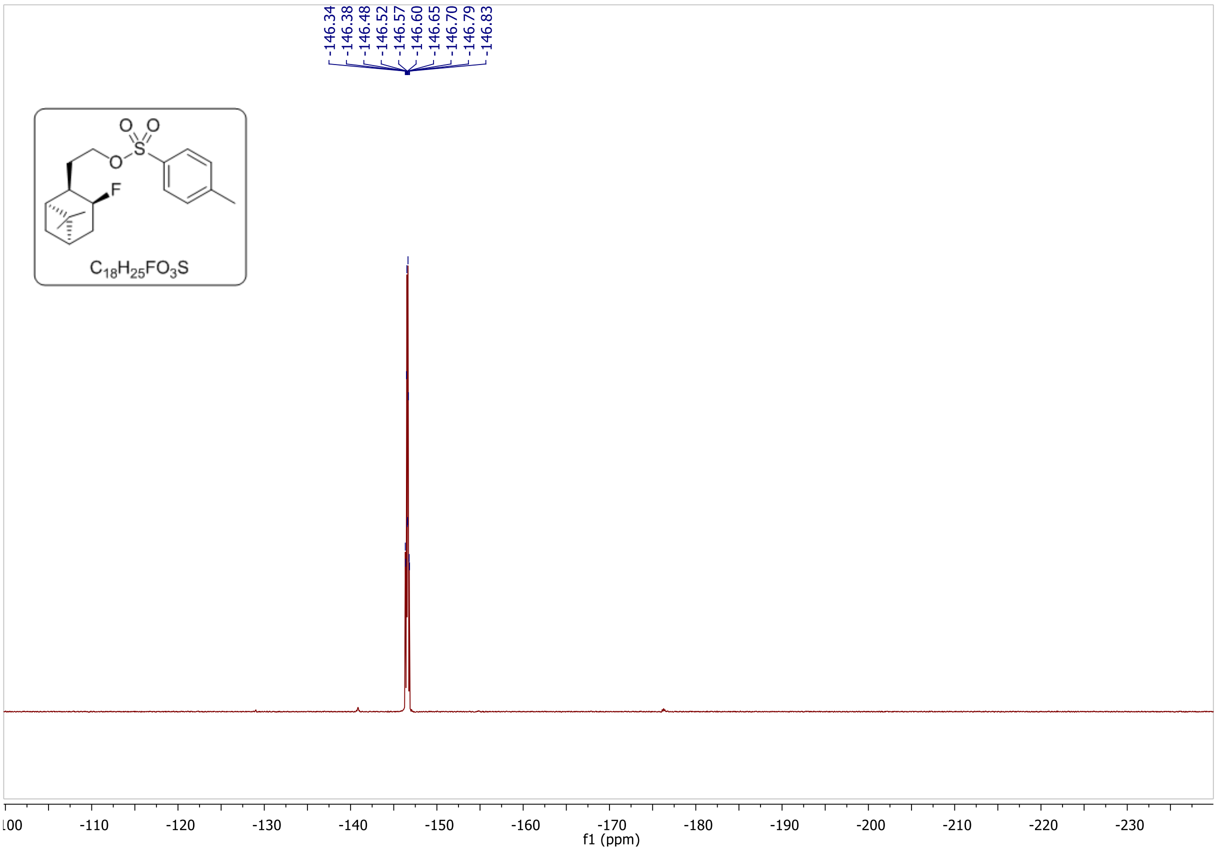

Supplementary Figure 165. ^{19}F-NMR (282 MHz) of **2d**.

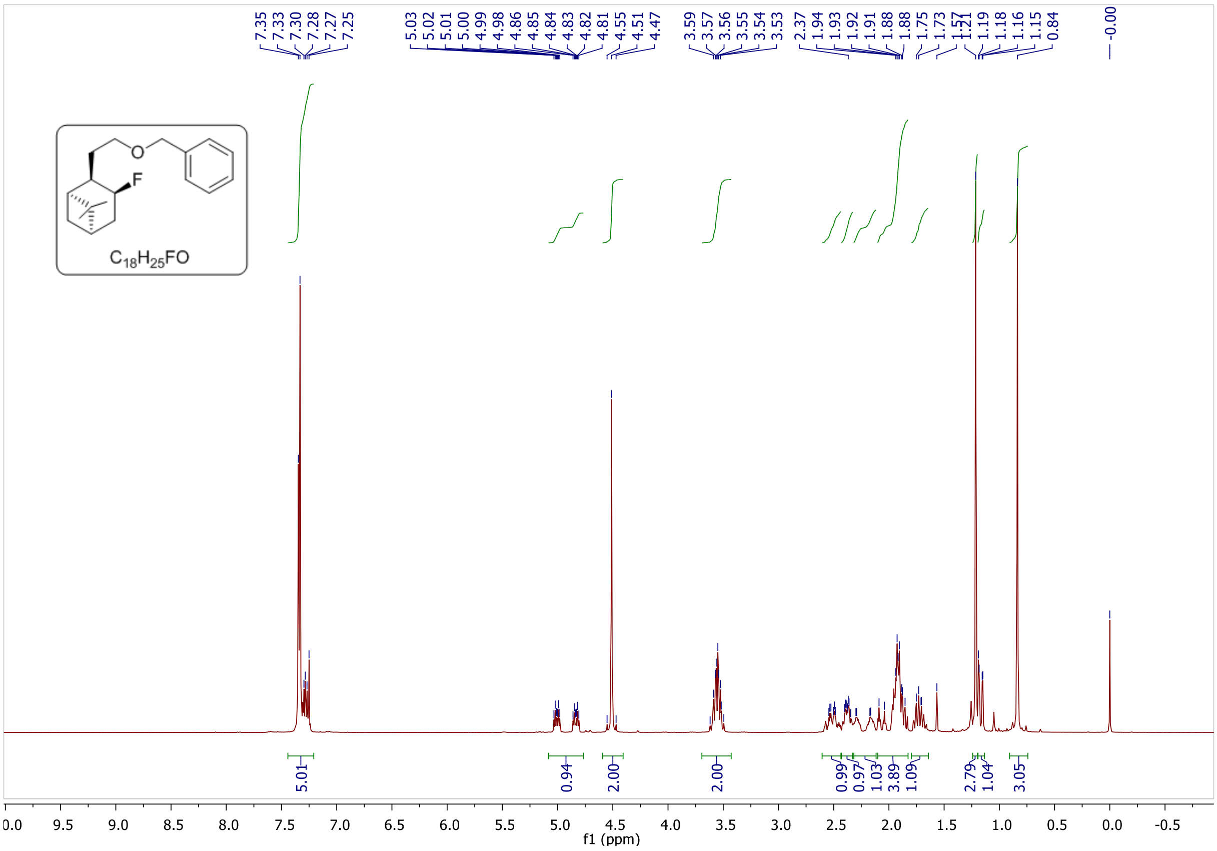

Supplementary Figure 166. ^{1}H-NMR (300 MHz) of **2e**.

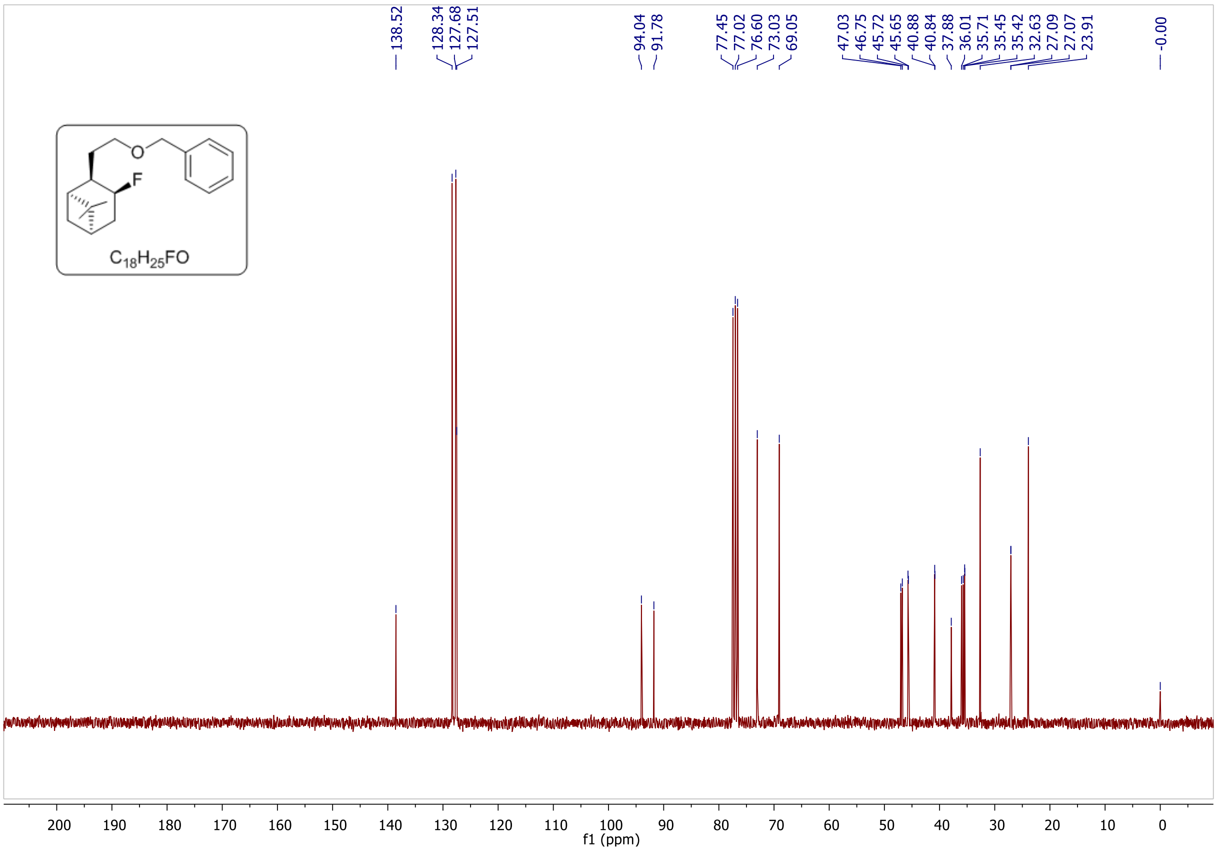

Supplementary Figure 167. ^{13}C-NMR (75 MHz) of **2e**.

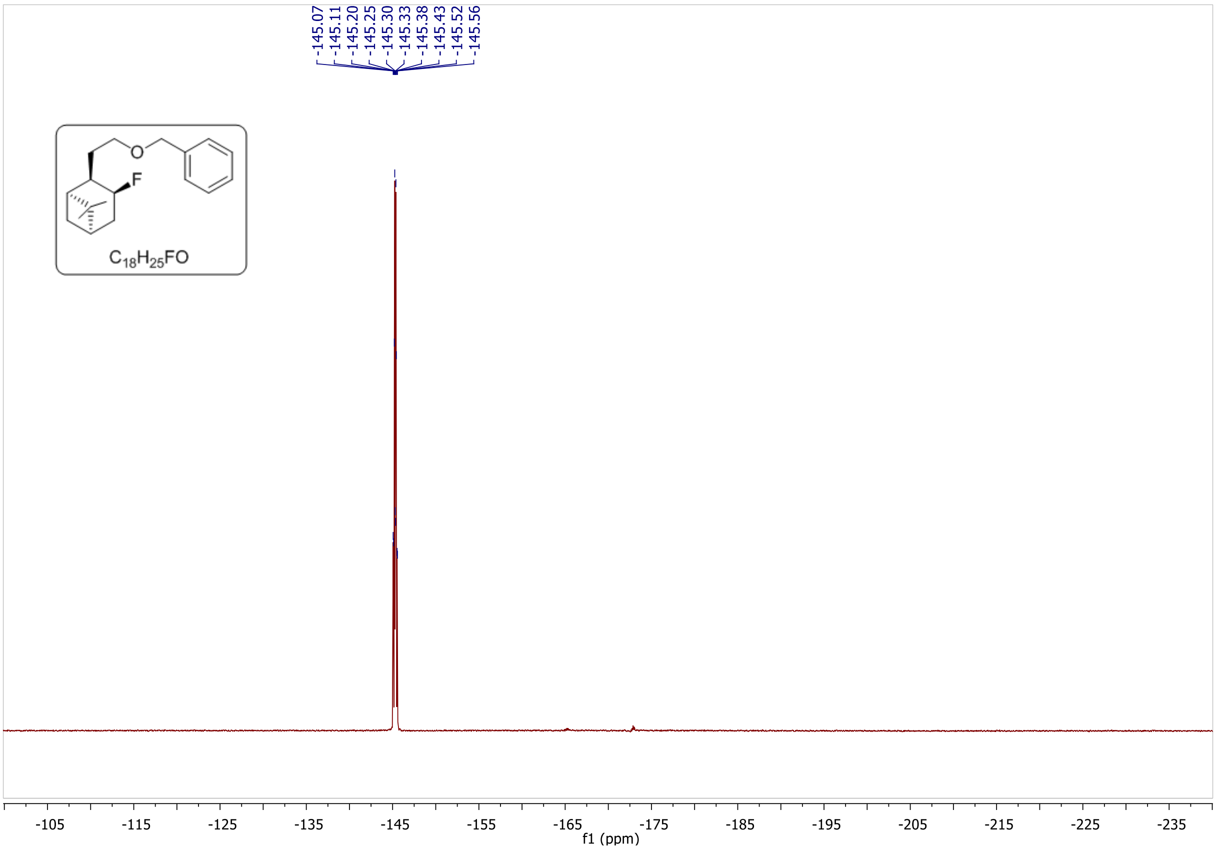

Supplementary Figure 168. ^{19}F-NMR (282 MHz) of **2e**.

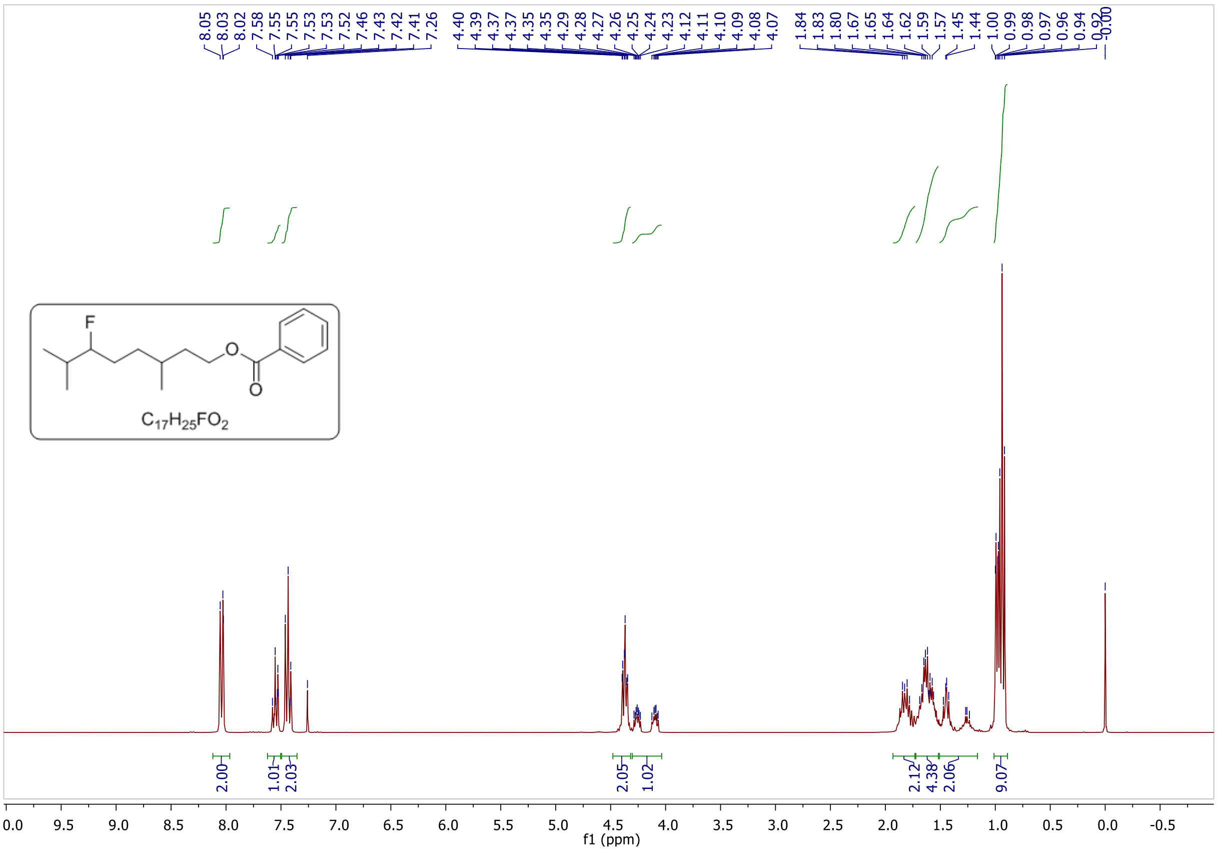

Supplementary Figure 169. ^{1}H-NMR (300 MHz) of **2f**.

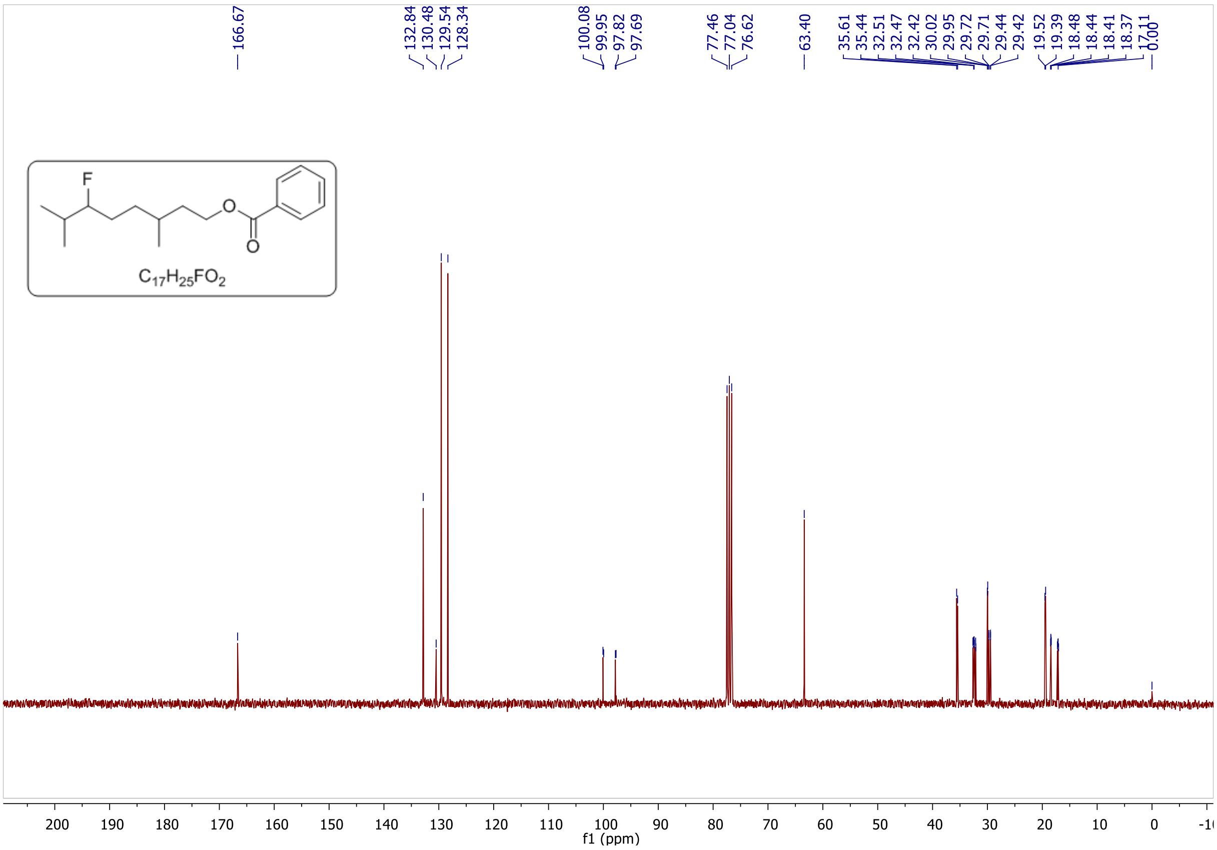

Supplementary Figure 170. ^{13}C-NMR (75 MHz) of **2f**.

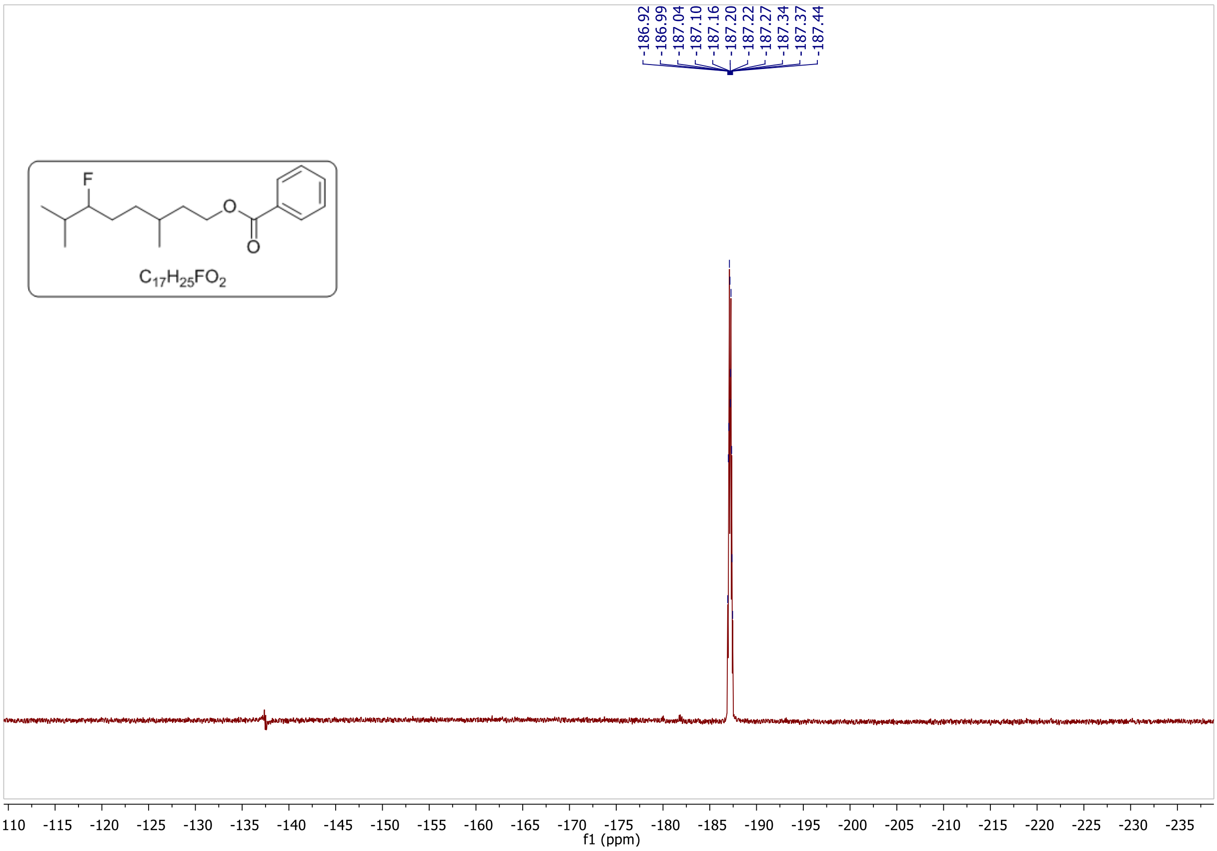

Supplementary Figure 171. ^{19}F-NMR (282 MHz) of **2f**.

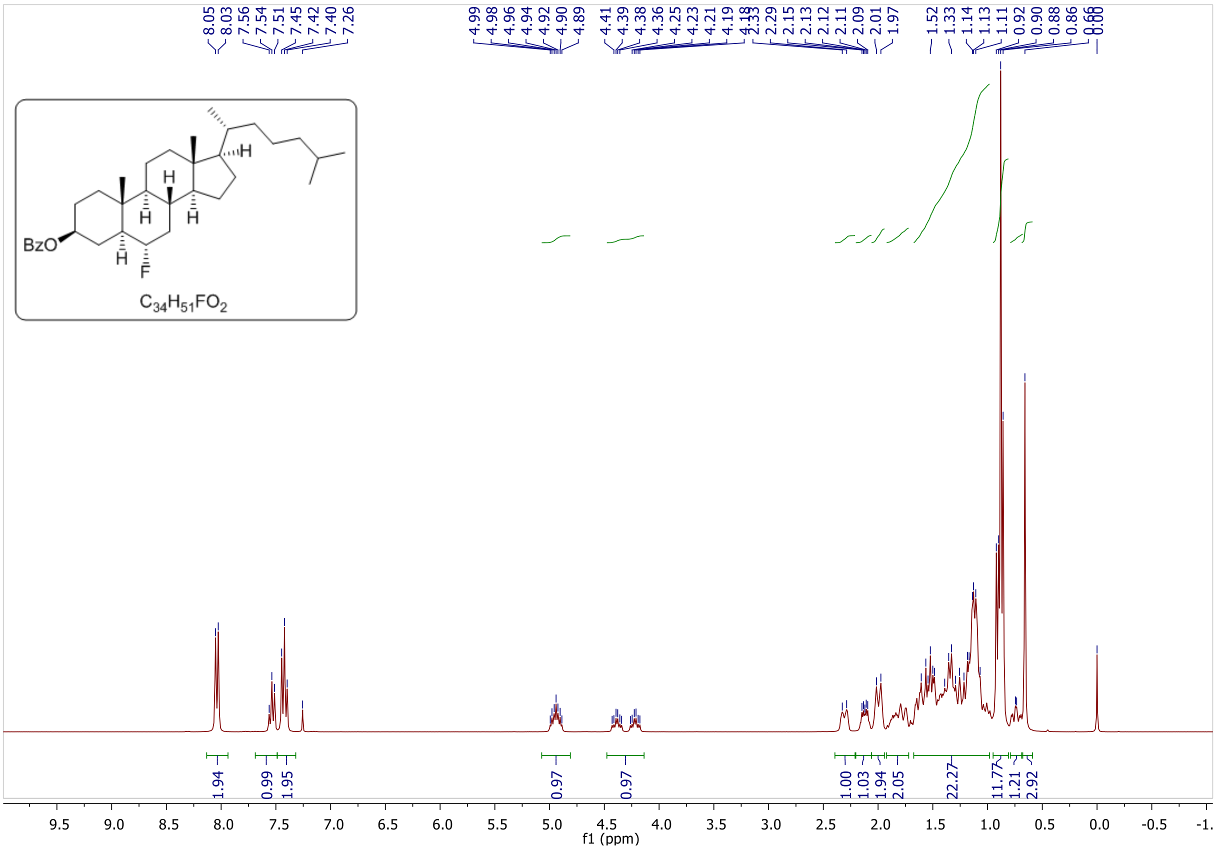

Supplementary Figure 172. ^{1}H-NMR (300 MHz) of **2g**.

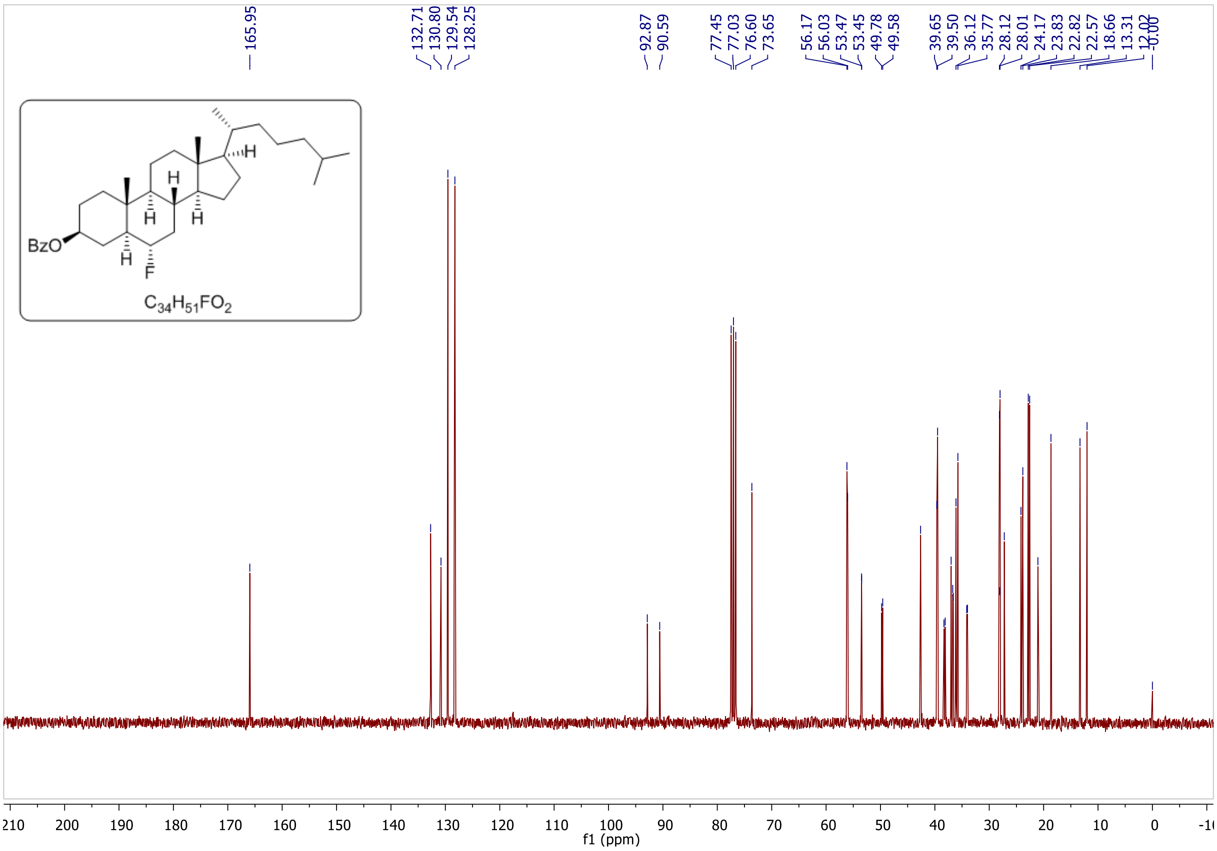

Supplementary Figure 173. ^{13}C-NMR (75 MHz) of **2g**.

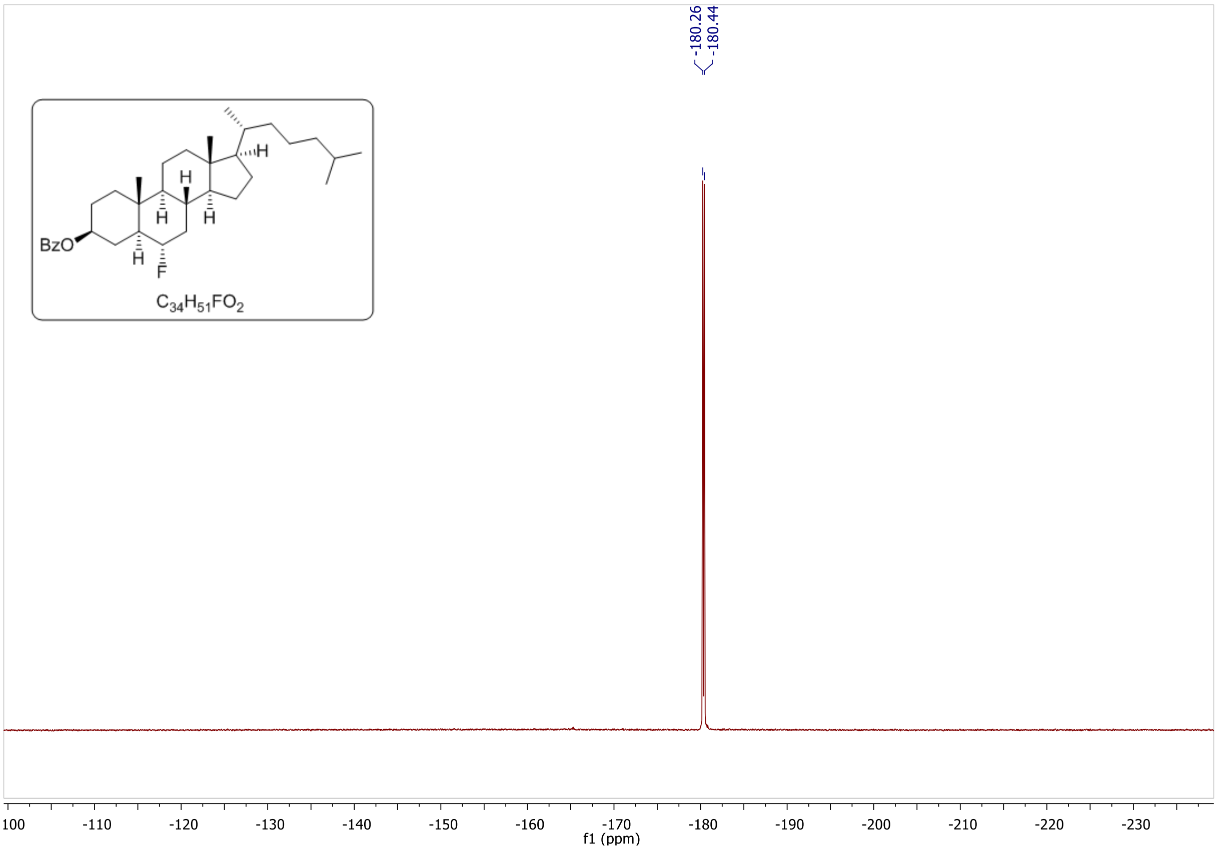

Supplementary Figure 174. ^{19}F-NMR (282 MHz) of **2g**.

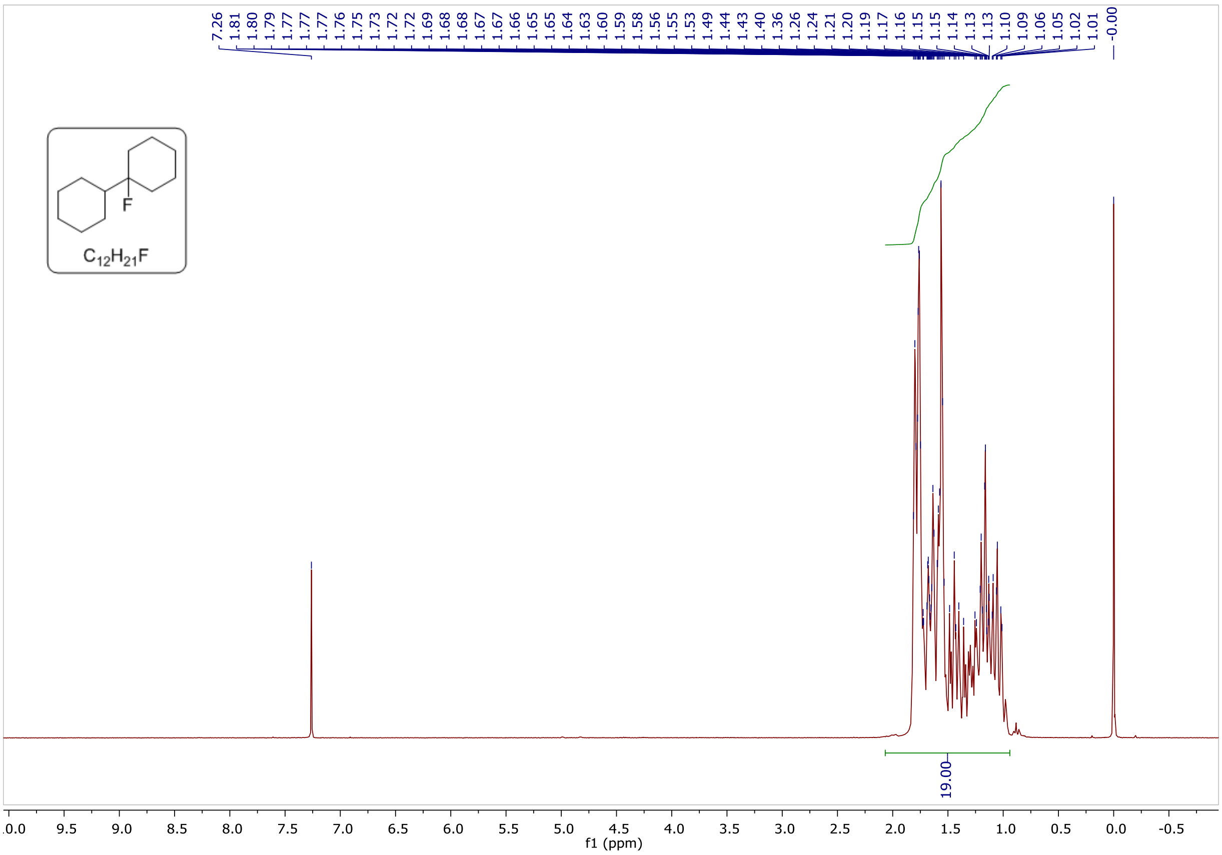

Supplementary Figure 175. ^{1}H-NMR (300 MHz) of **2h**.

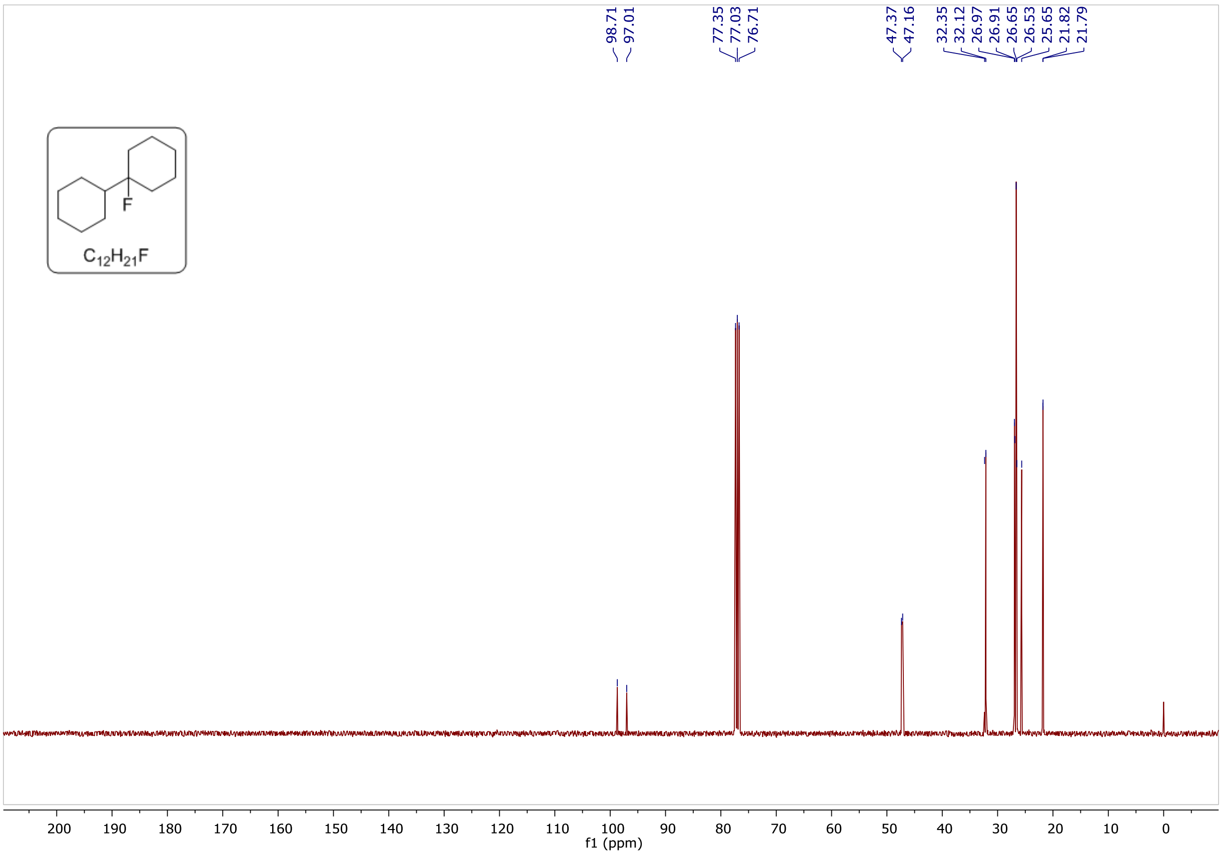

Supplementary Figure 176. ^{13}C-NMR (101 MHz) of **2h**.

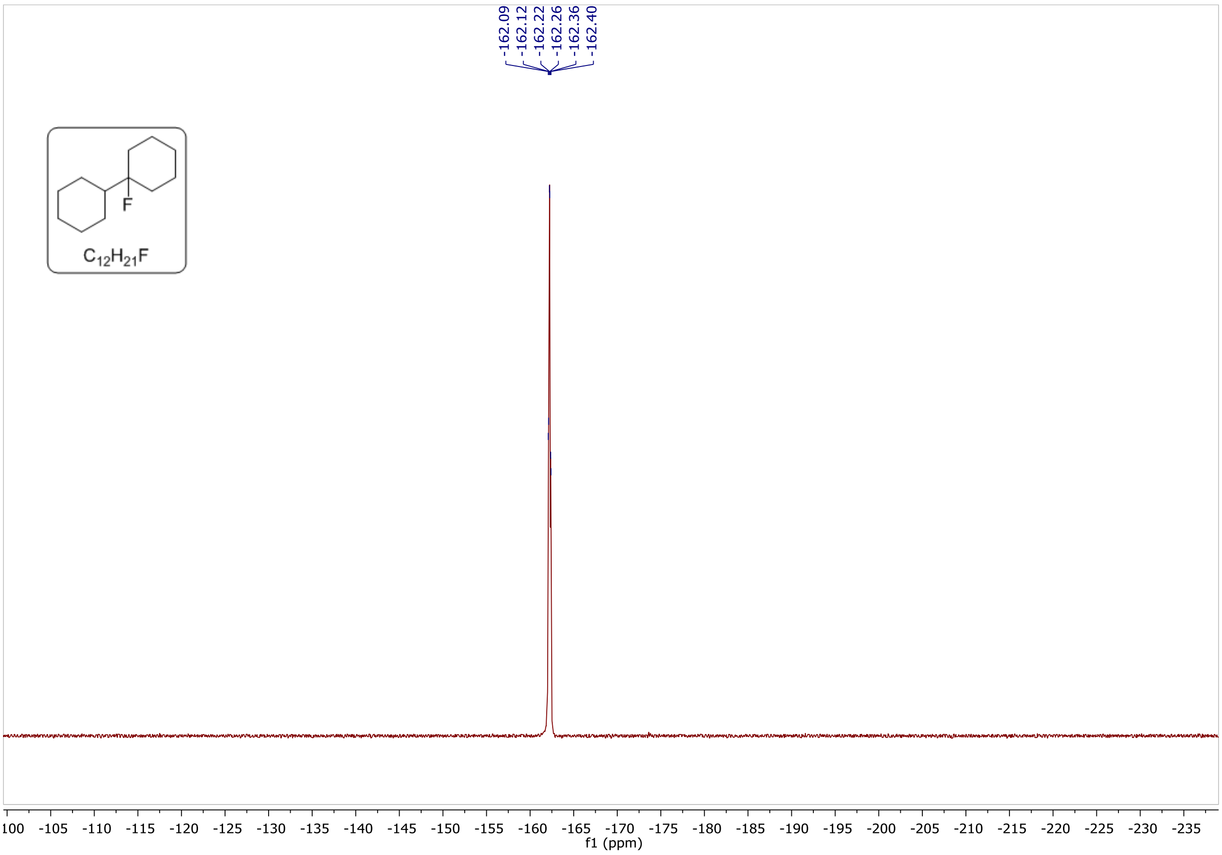

Supplementary Figure 177. ^{19}F-NMR (282 MHz) of **2h**.

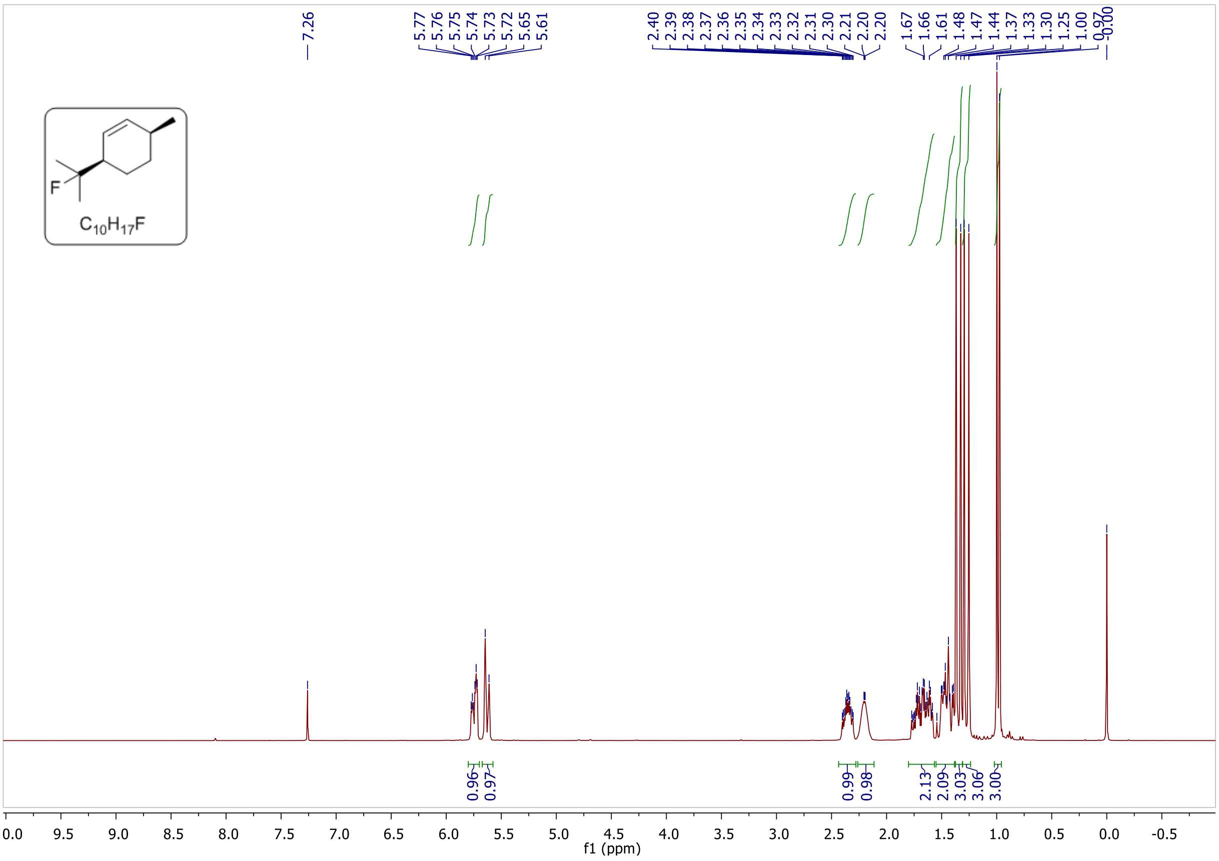

Supplementary Figure 178. ^{1}H-NMR (300 MHz) of **2i**.

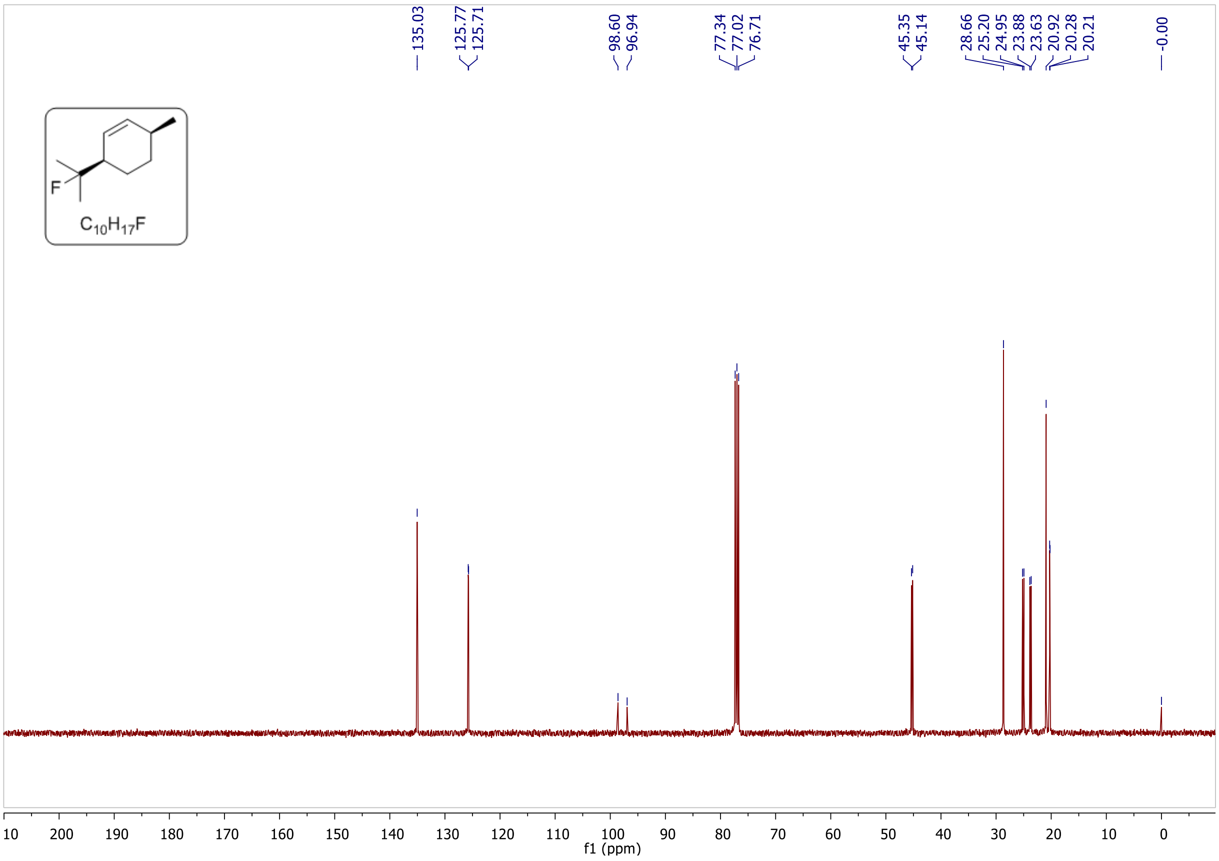

Supplementary Figure 179. ^{13}C-NMR (75 MHz) of **2i**.

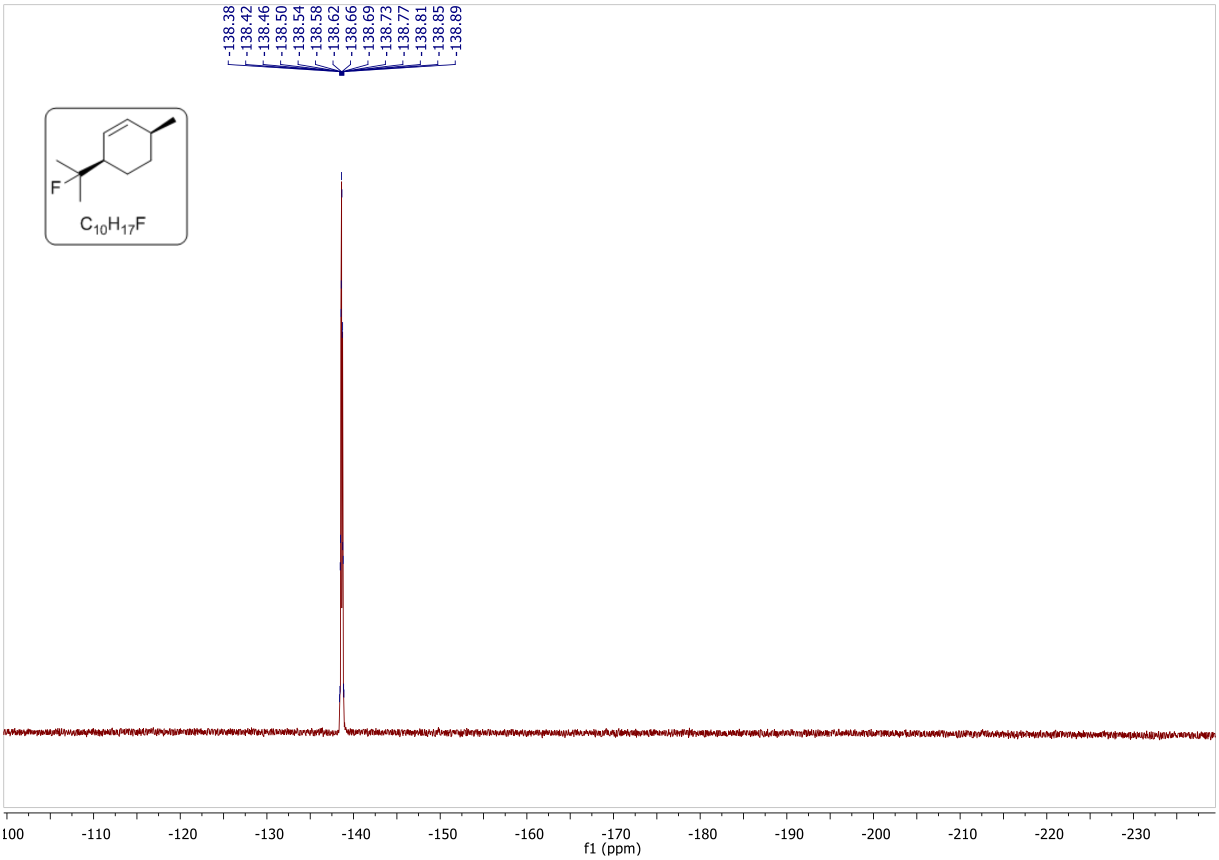

Supplementary Figure 180. ^{19}F-NMR (282 MHz) of **2i**.

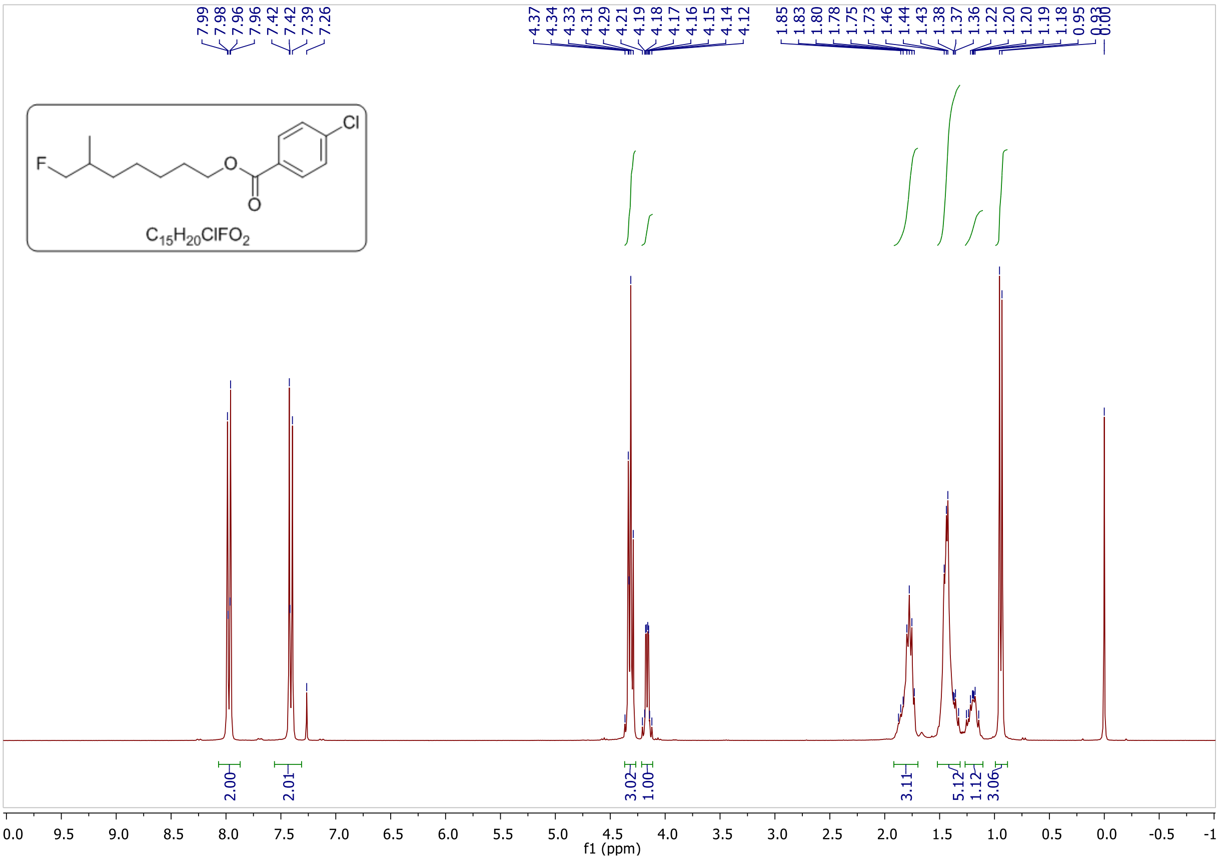

Supplementary Figure 181. ^{1}H-NMR (300 MHz) of **9a**.

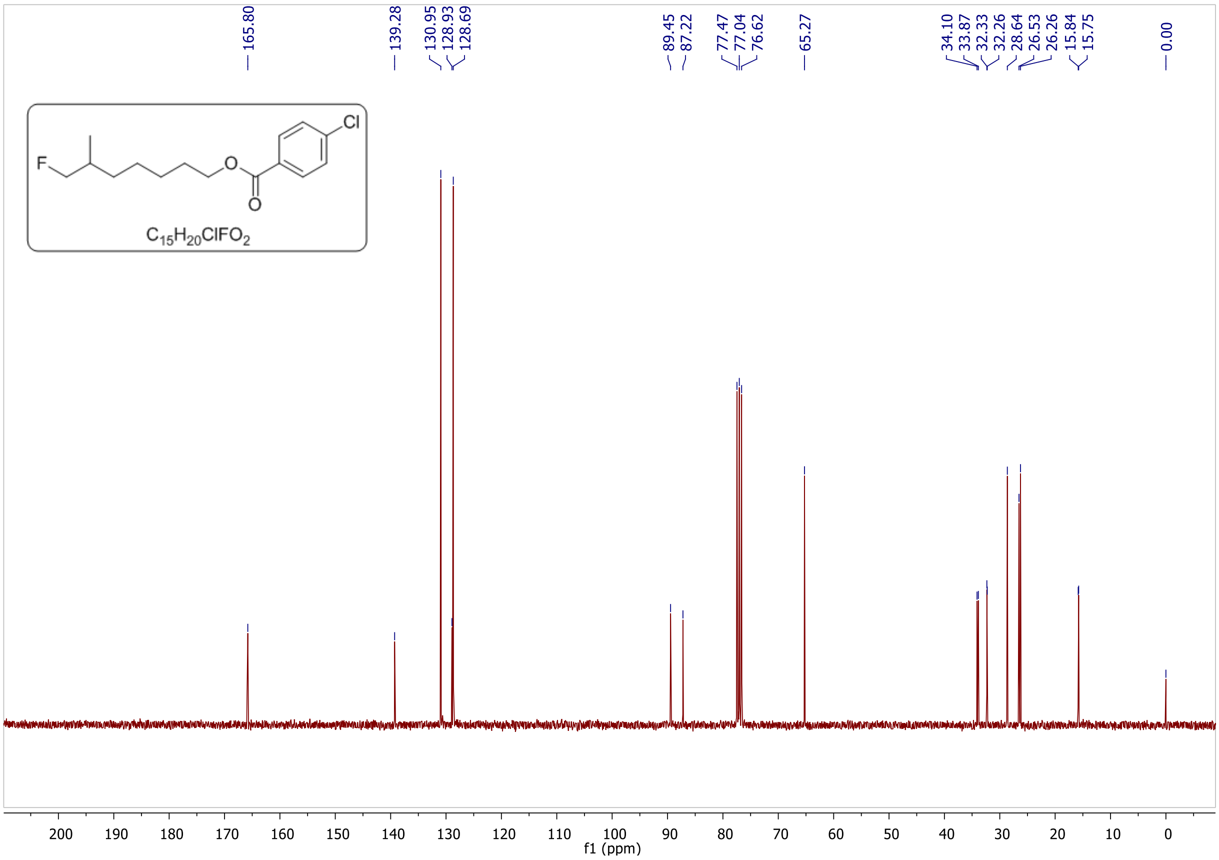

Supplementary Figure 182. ^{13}C-NMR (75 MHz) of **9a**.

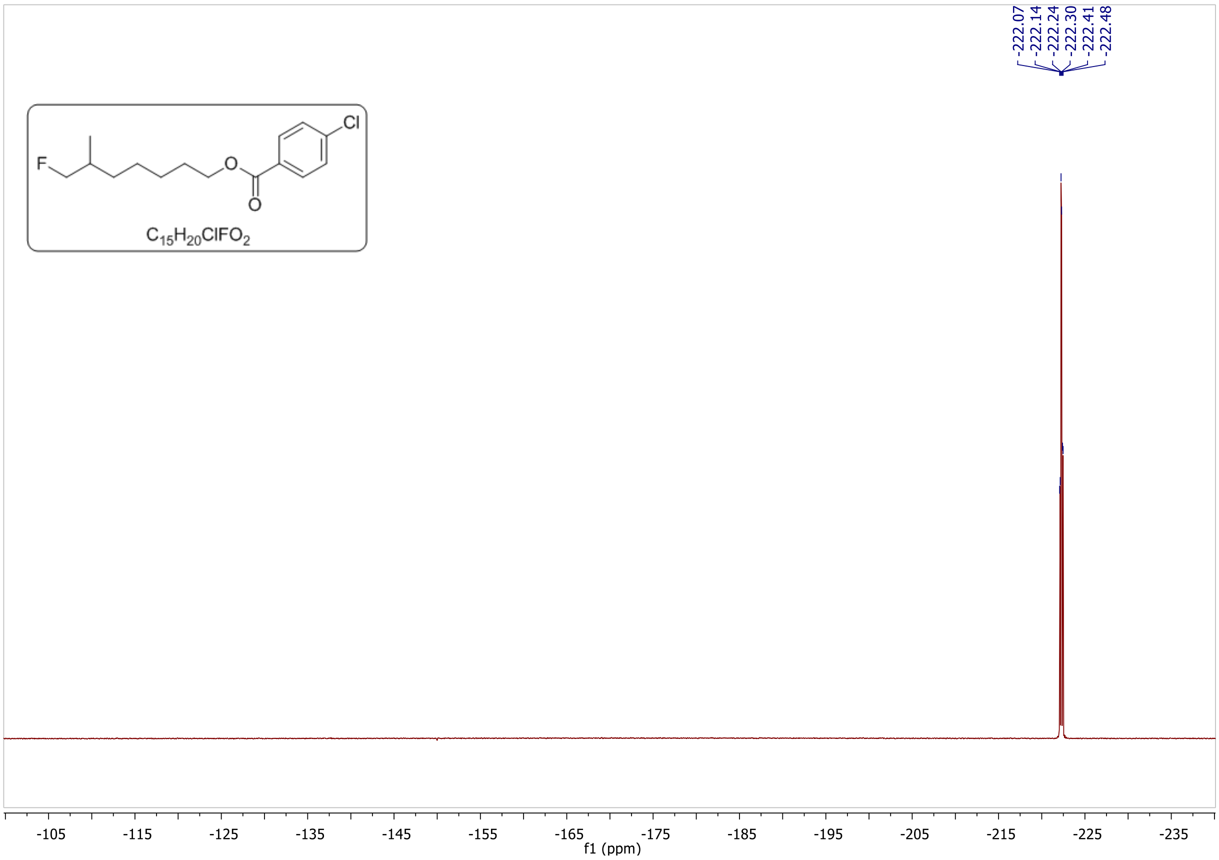

Supplementary Figure 183. ^{19}F-NMR (282 MHz) of **9a**.

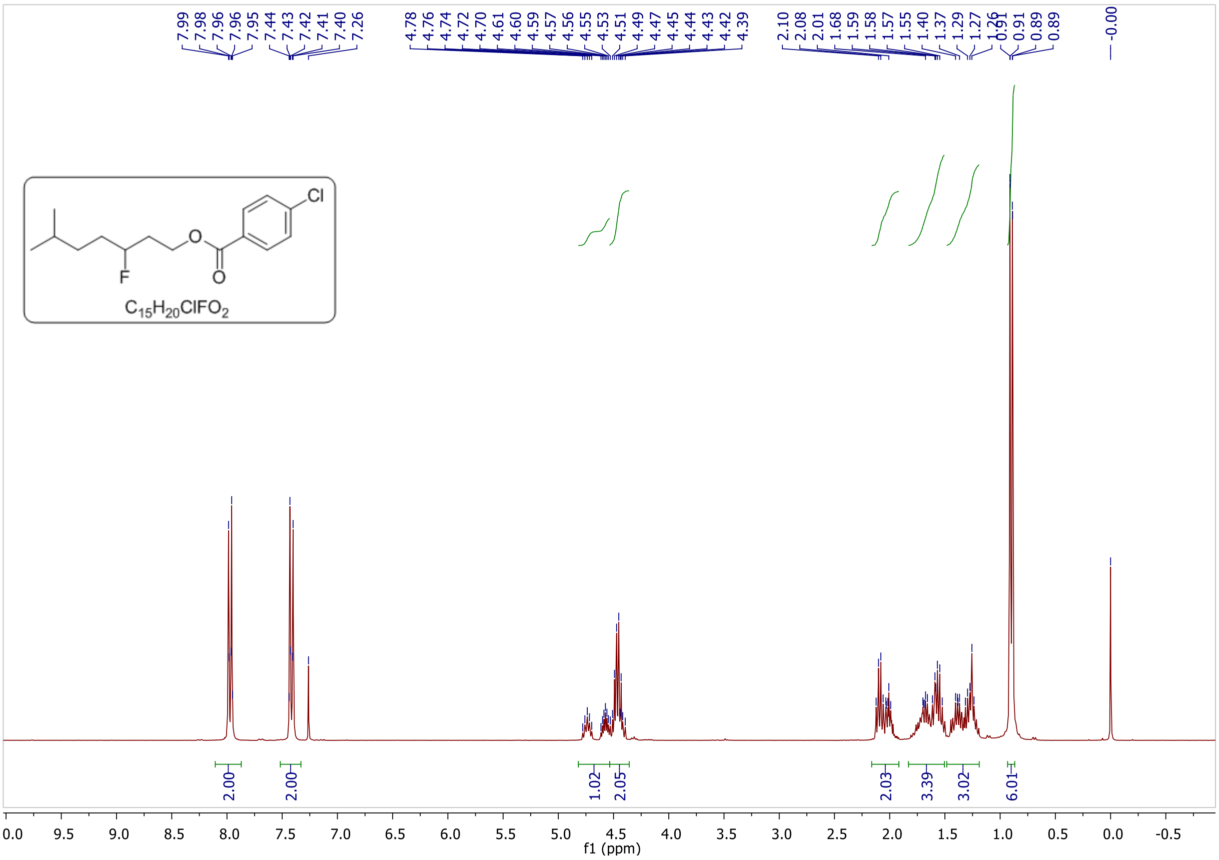

Supplementary Figure 184. ^{1}H-NMR (300 MHz) of **9a'**.

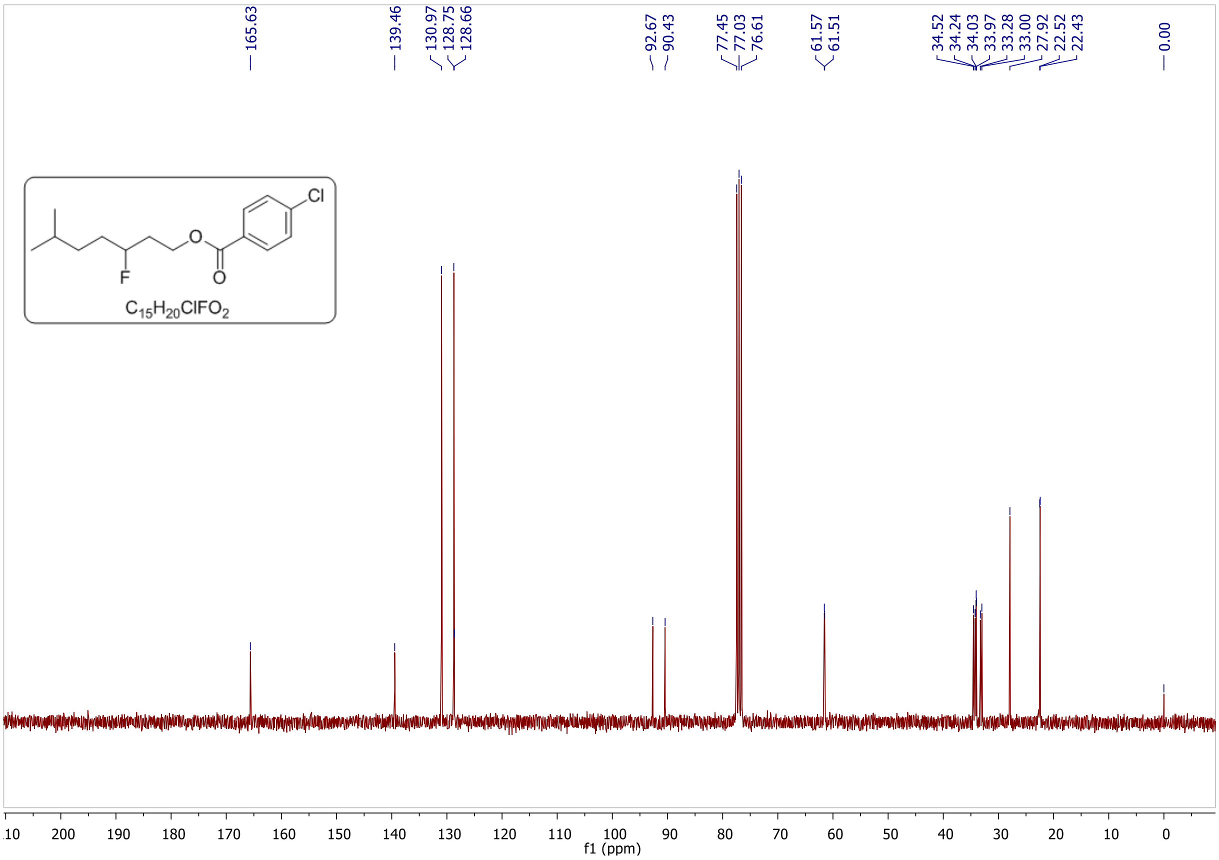

Supplementary Figure 185. ^{13}C-NMR (75 MHz) of **9a'**.

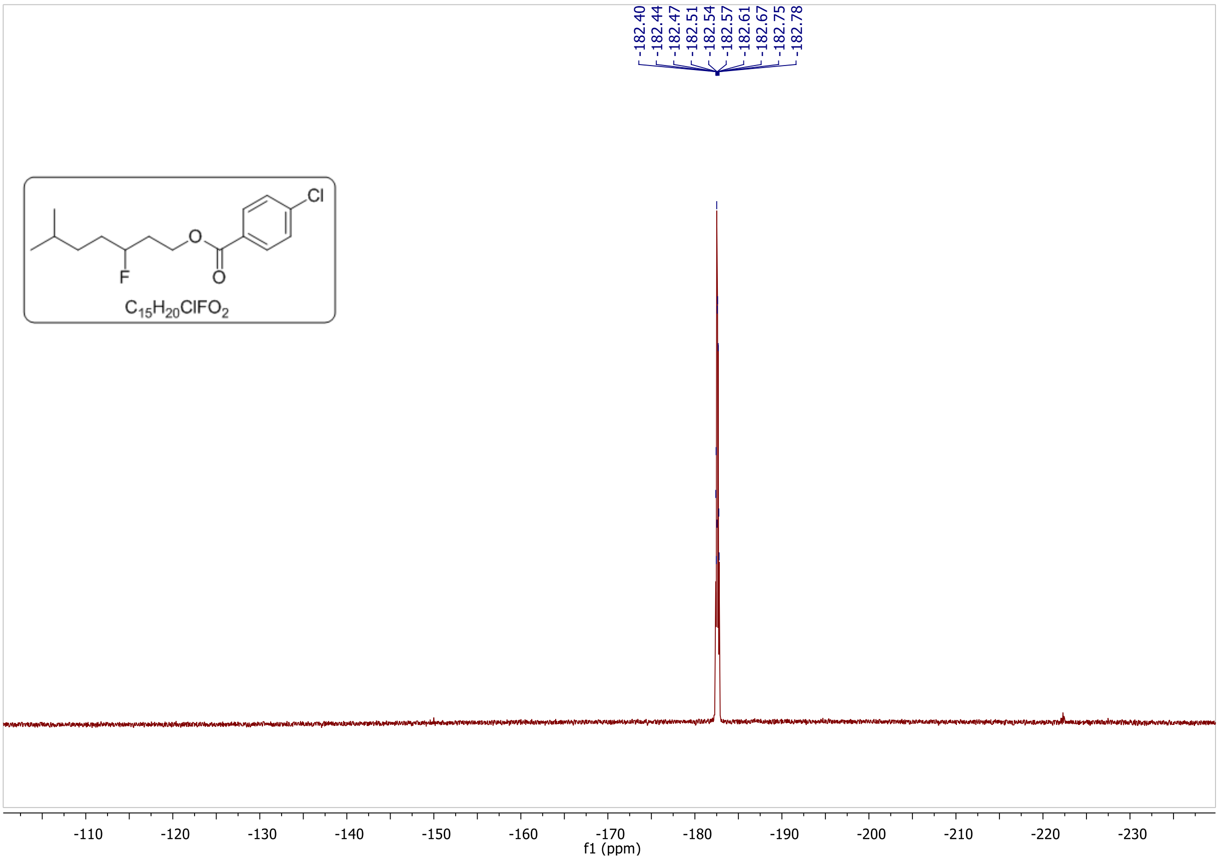

Supplementary Figure 186. ^{19}F-NMR (282 MHz) of **9a'**.

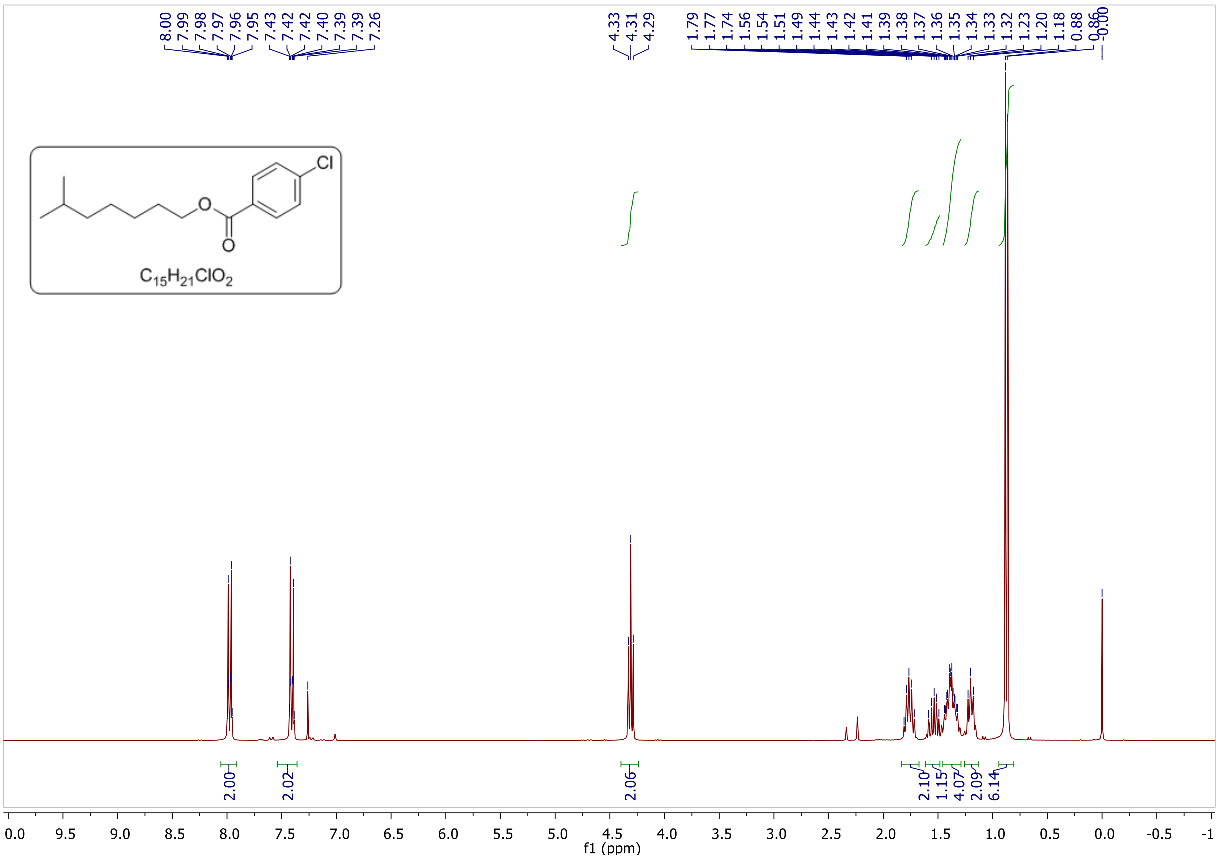

Supplementary Figure 187. ^{1}H-NMR (300 MHz) of **10a**.

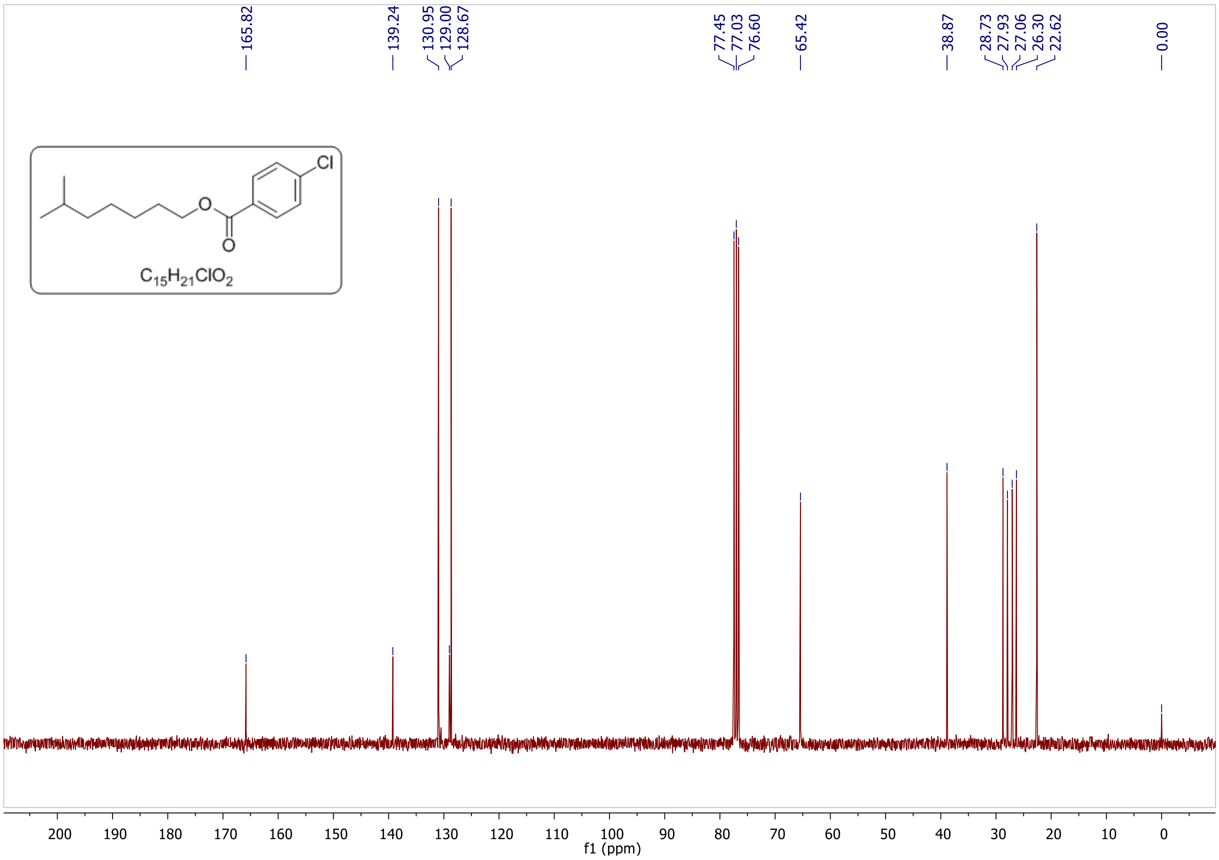

Supplementary Figure 188. ^{13}C-NMR (75 MHz) of **10a**.

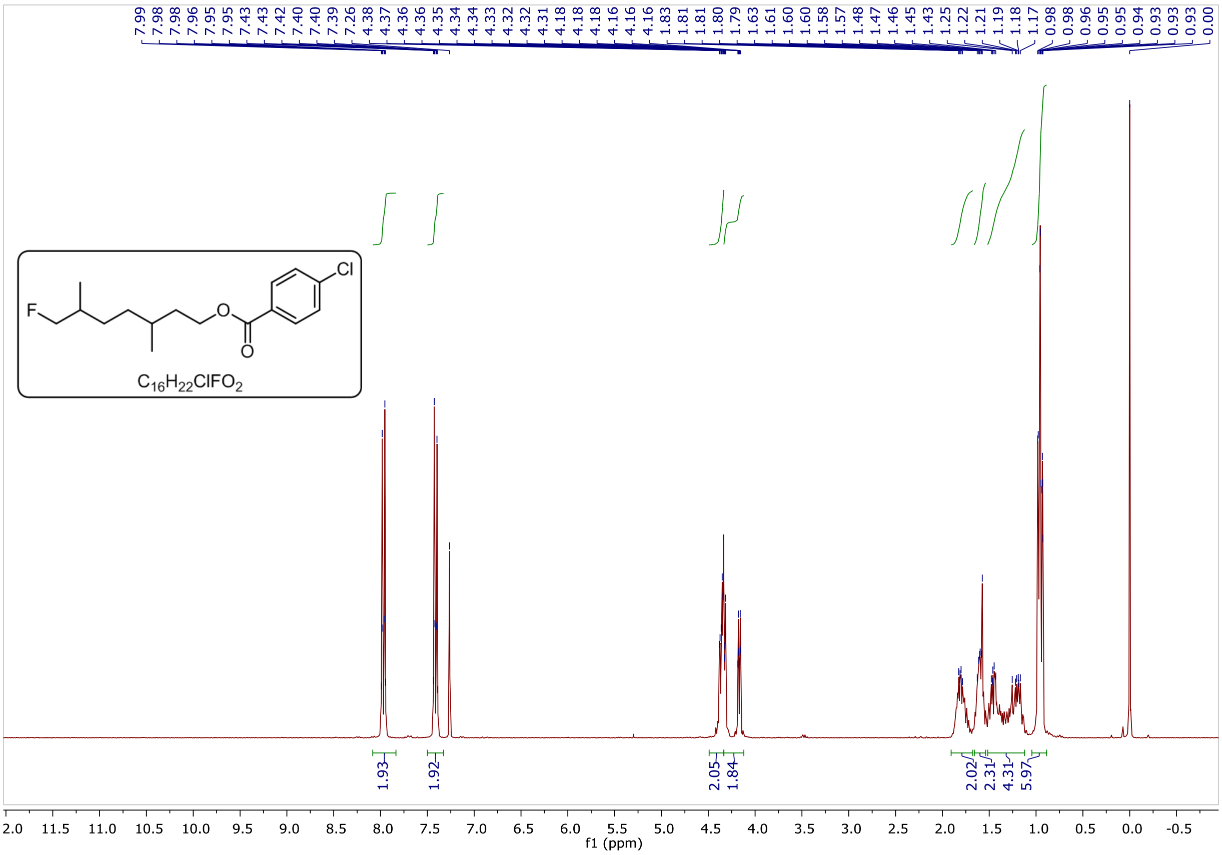

Supplementary Figure 189. ^{1}H-NMR (300 MHz) of **9b**.

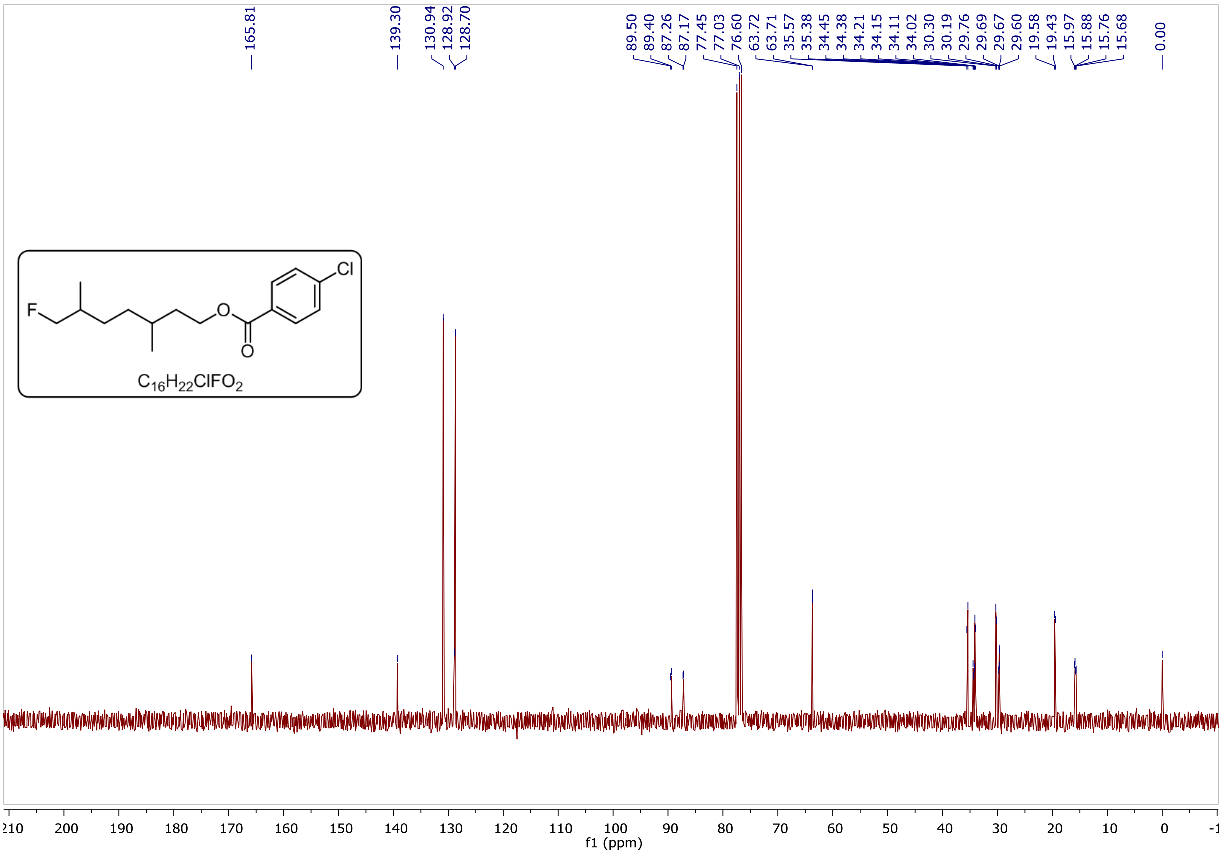

Supplementary Figure 190. ^{13}C-NMR (75 MHz) of **9b**.

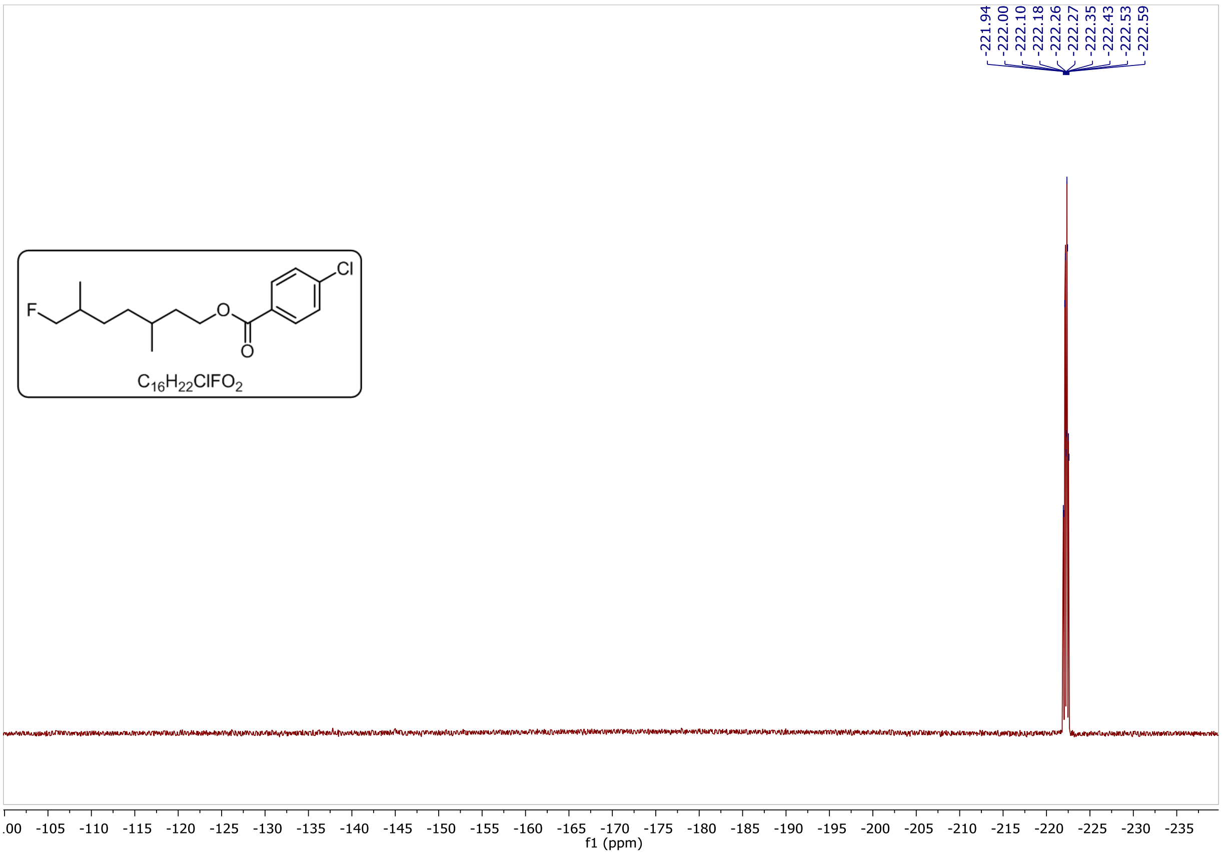

Supplementary Figure 191. ^{19}F-NMR (282 MHz) of **9b**.

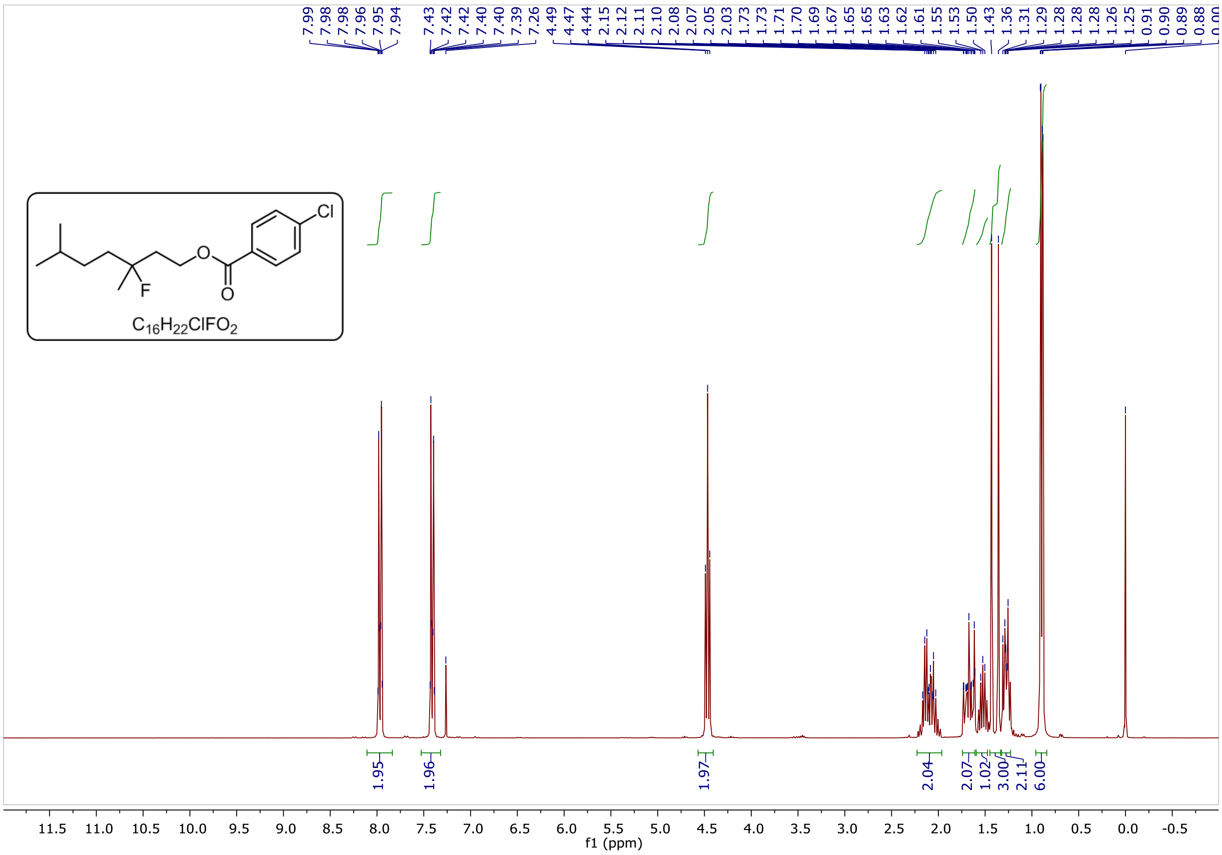

Supplementary Figure 192. ^{1}H-NMR (300 MHz) of **9b’**.

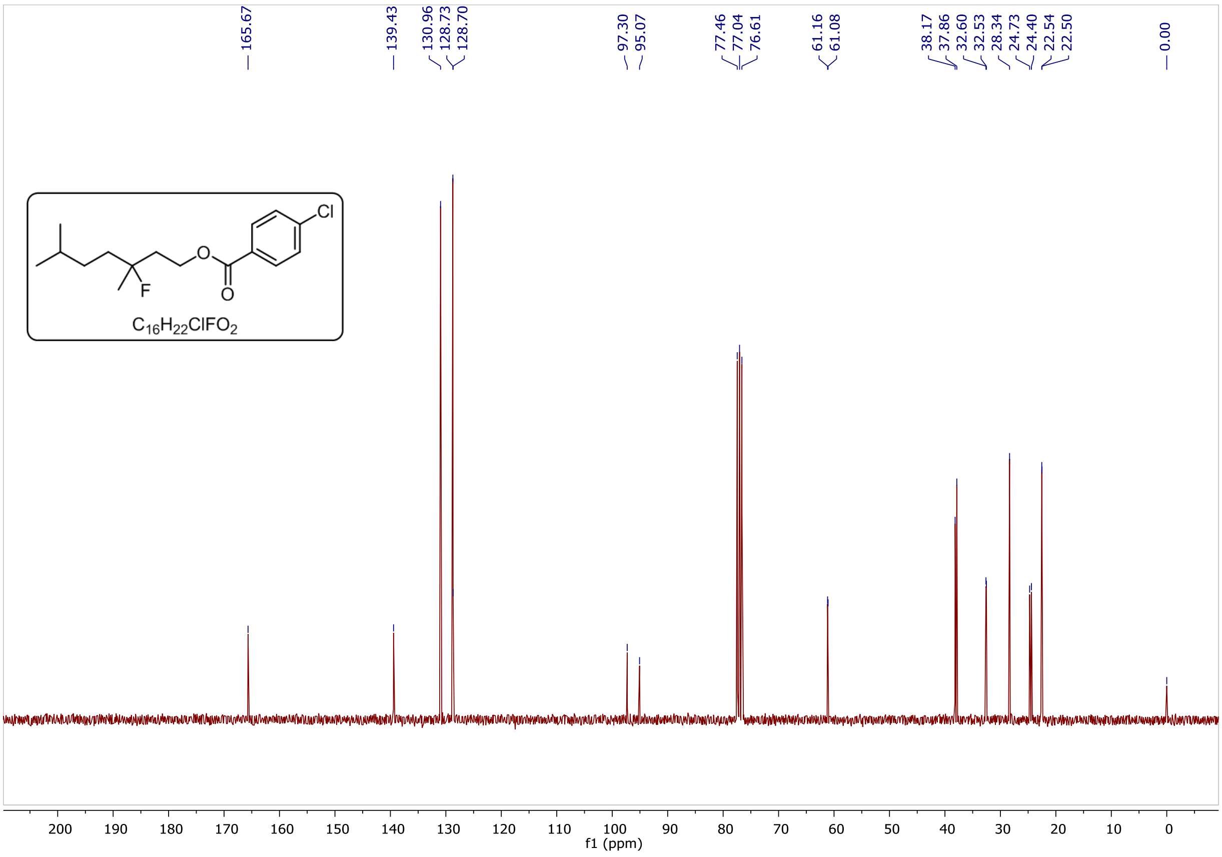

Supplementary Figure 193. ^{13}C-NMR (75 MHz) of **9b’**.

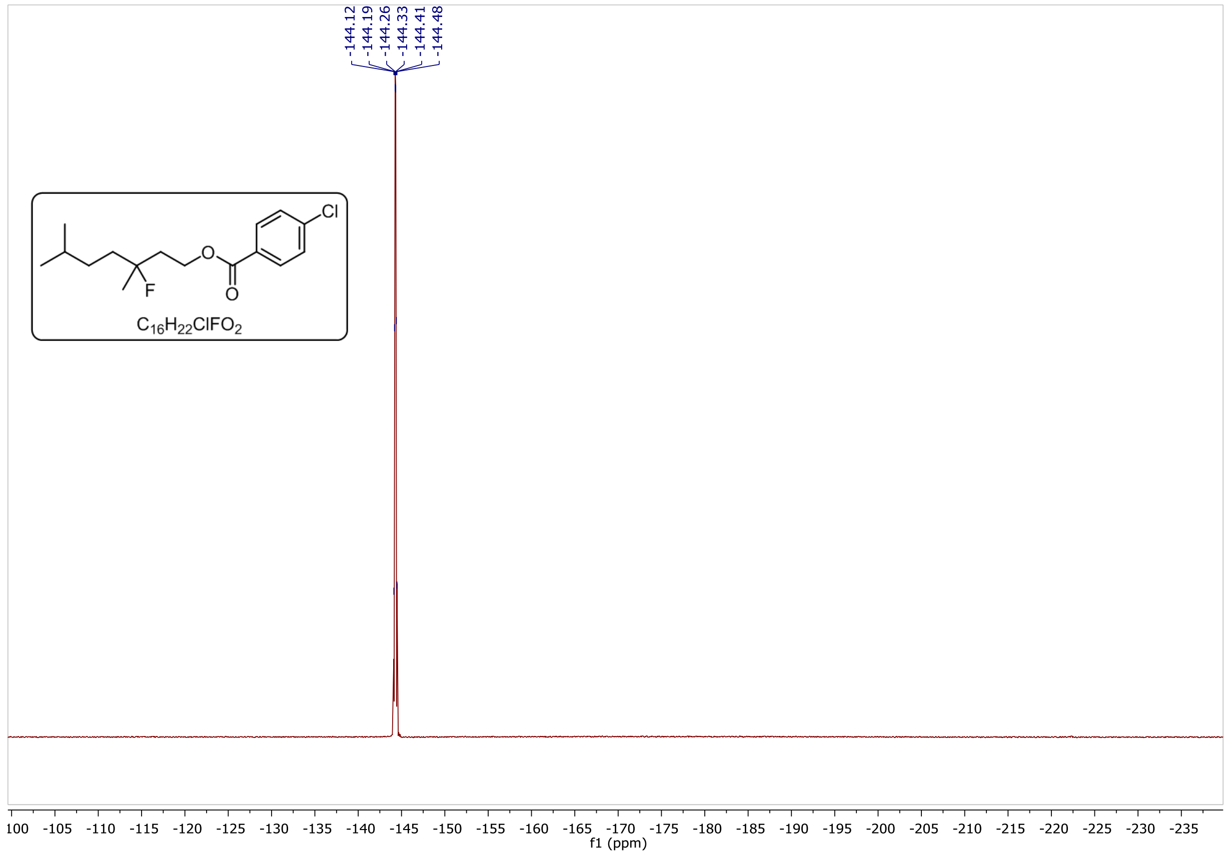

Supplementary Figure 194. ^{19}F-NMR (282 MHz) of **9b'**.

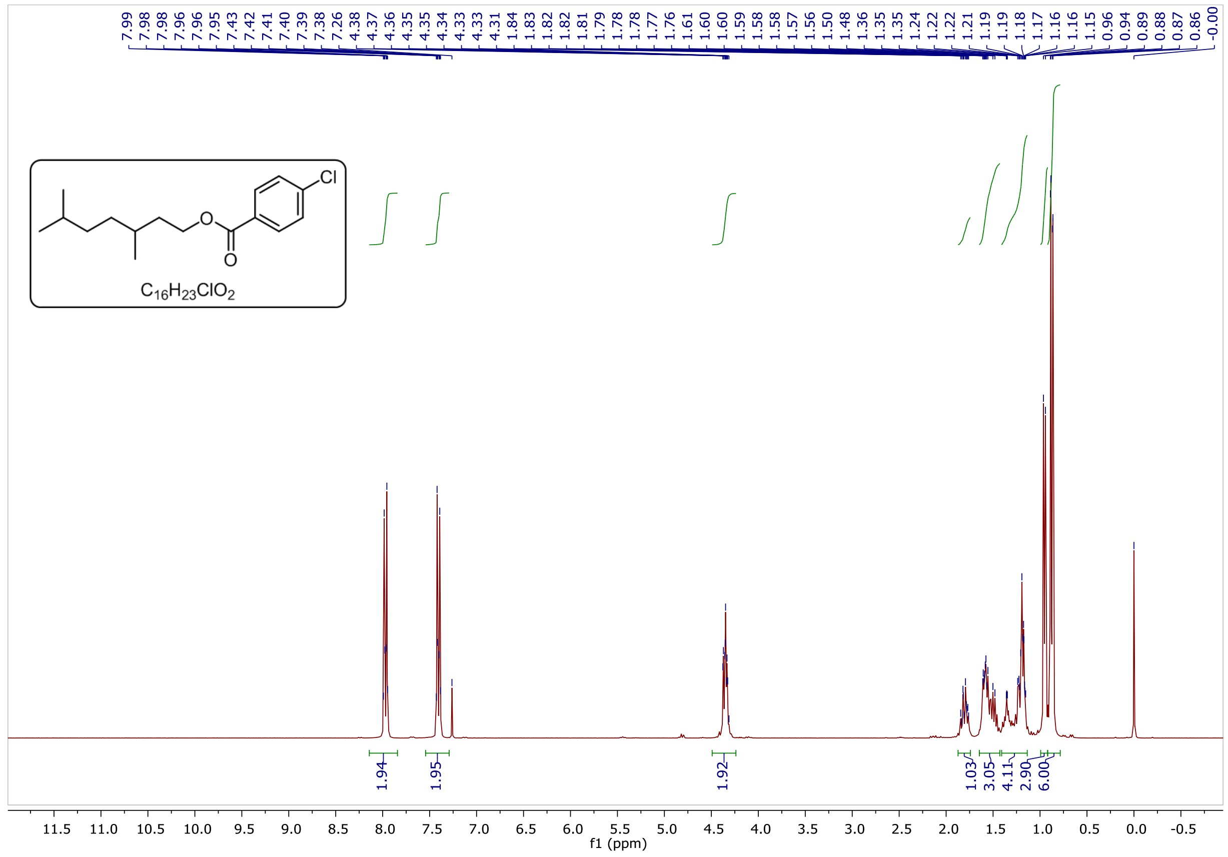

Supplementary Figure 195. ^{1}H-NMR (300 MHz) of **10b**.

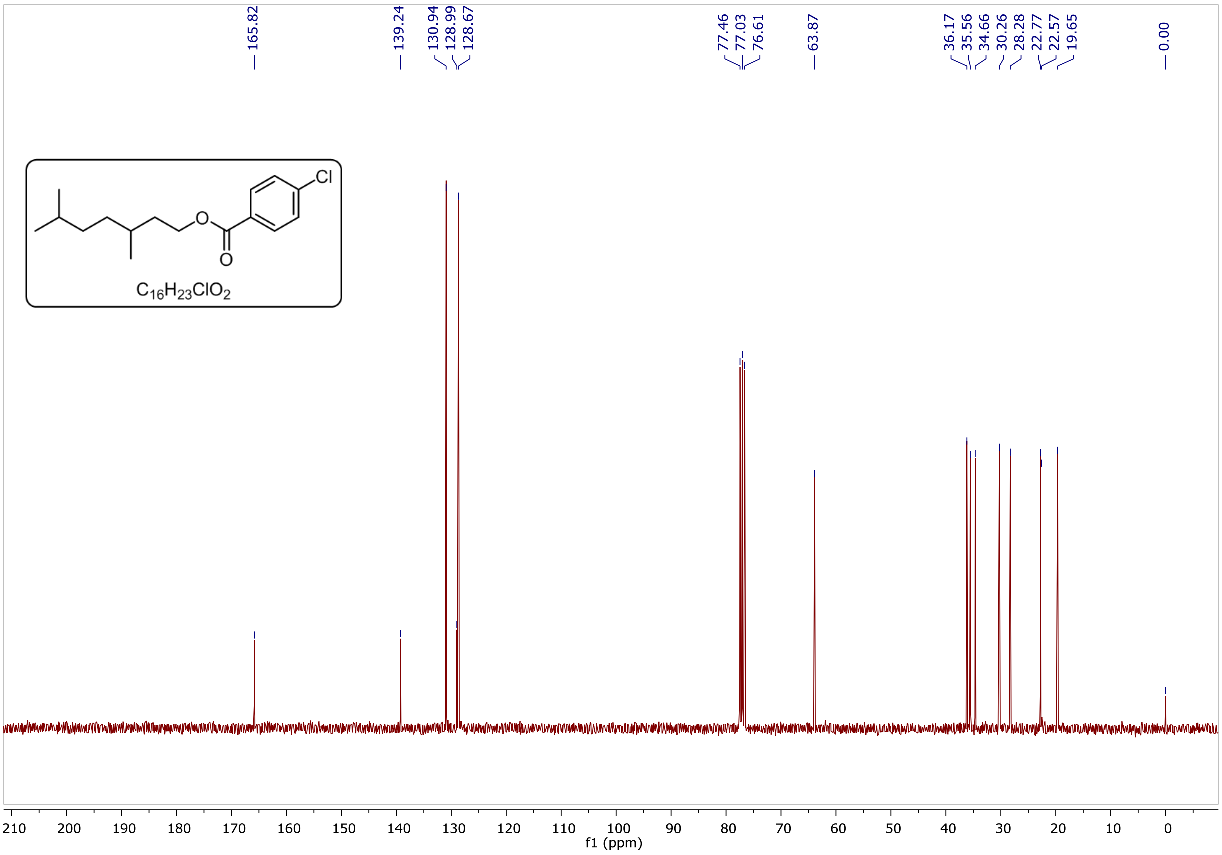

Supplementary Figure 196. ^{13}C-NMR (75 MHz) of **10b**.

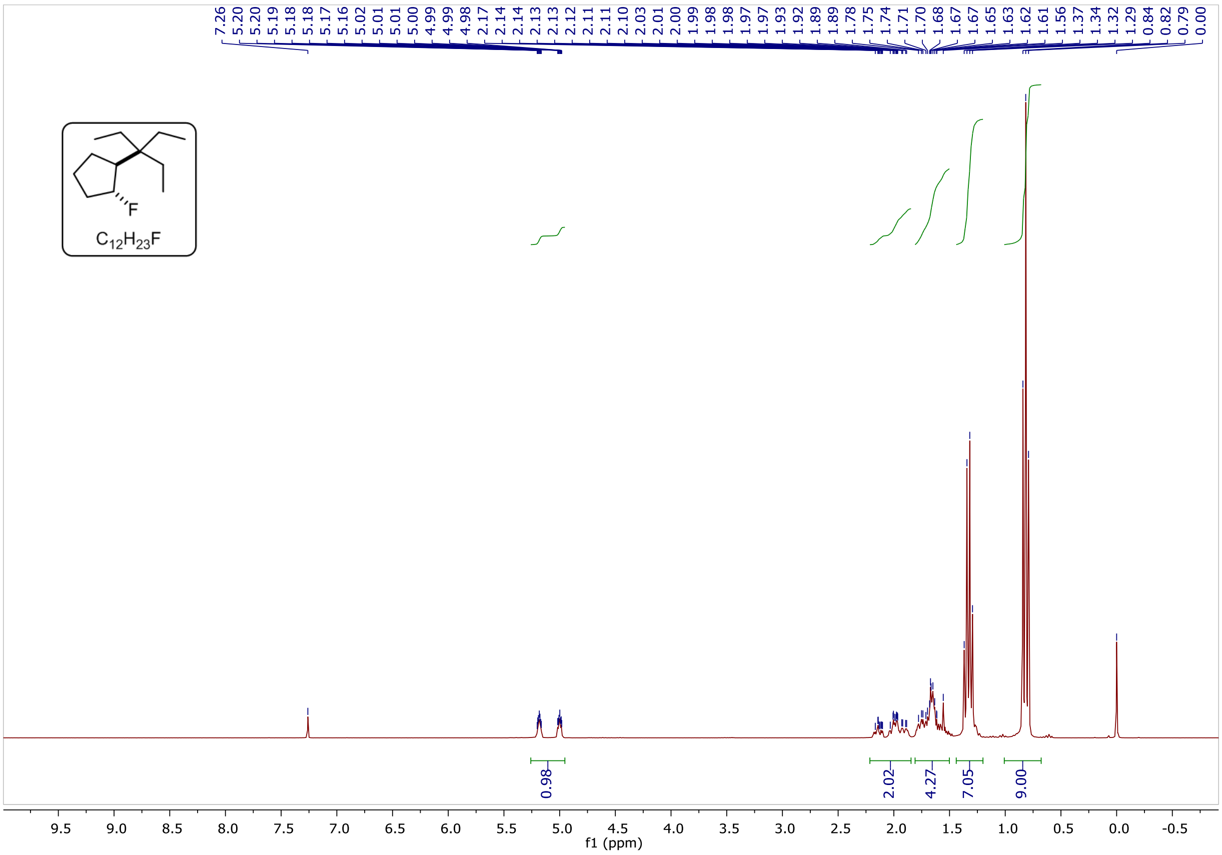

Supplementary Figure 197. ^{1}H-NMR (300 MHz) of **12**.

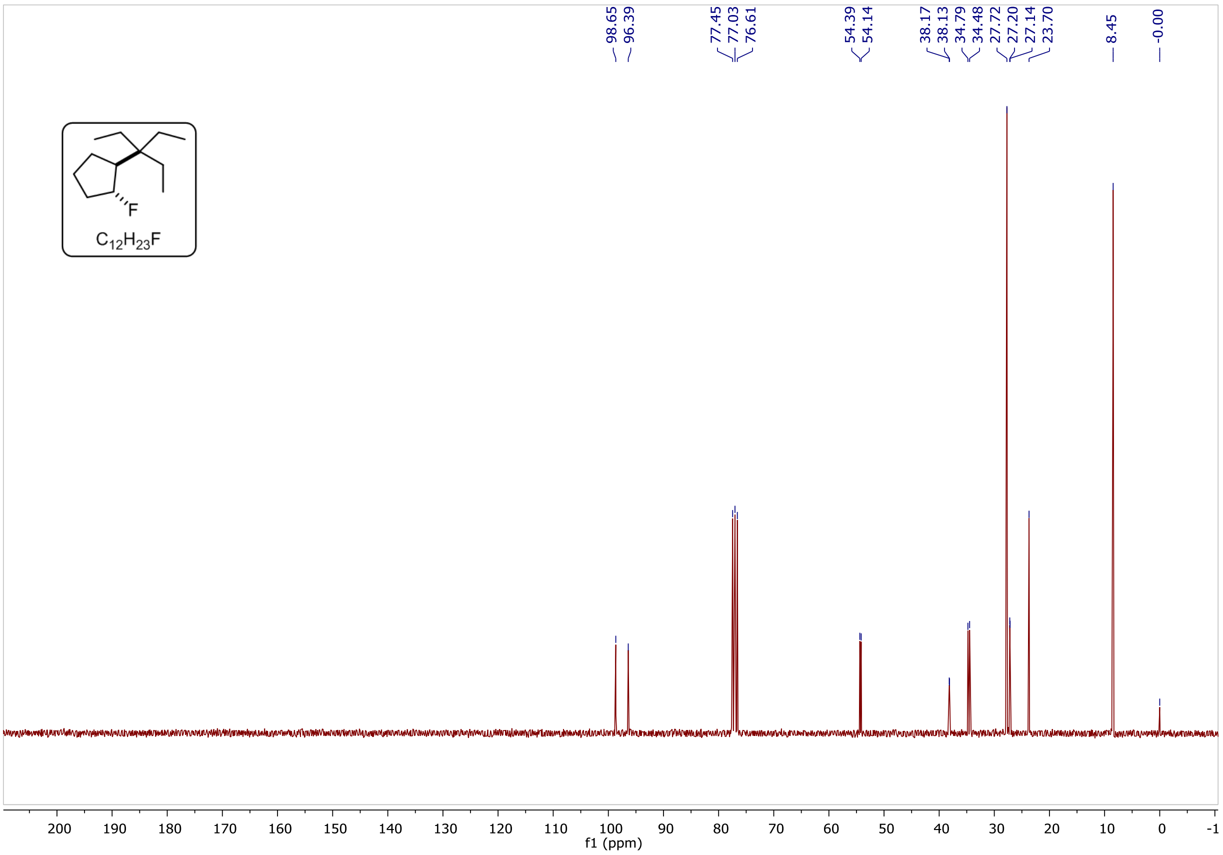

Supplementary Figure 198. ^{13}C-NMR (75 MHz) of **12**.

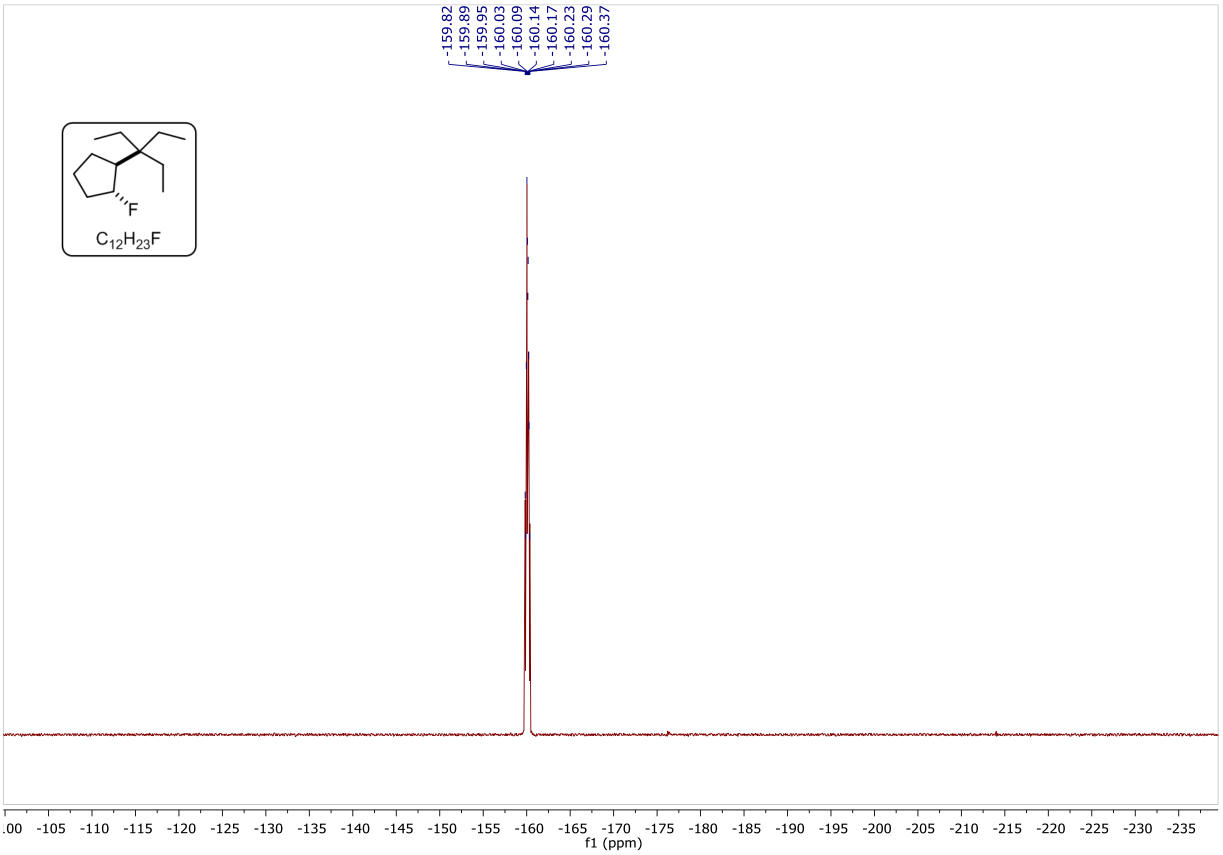

Supplementary Figure 199. ^{19}F-NMR (282 MHz) of **12**.

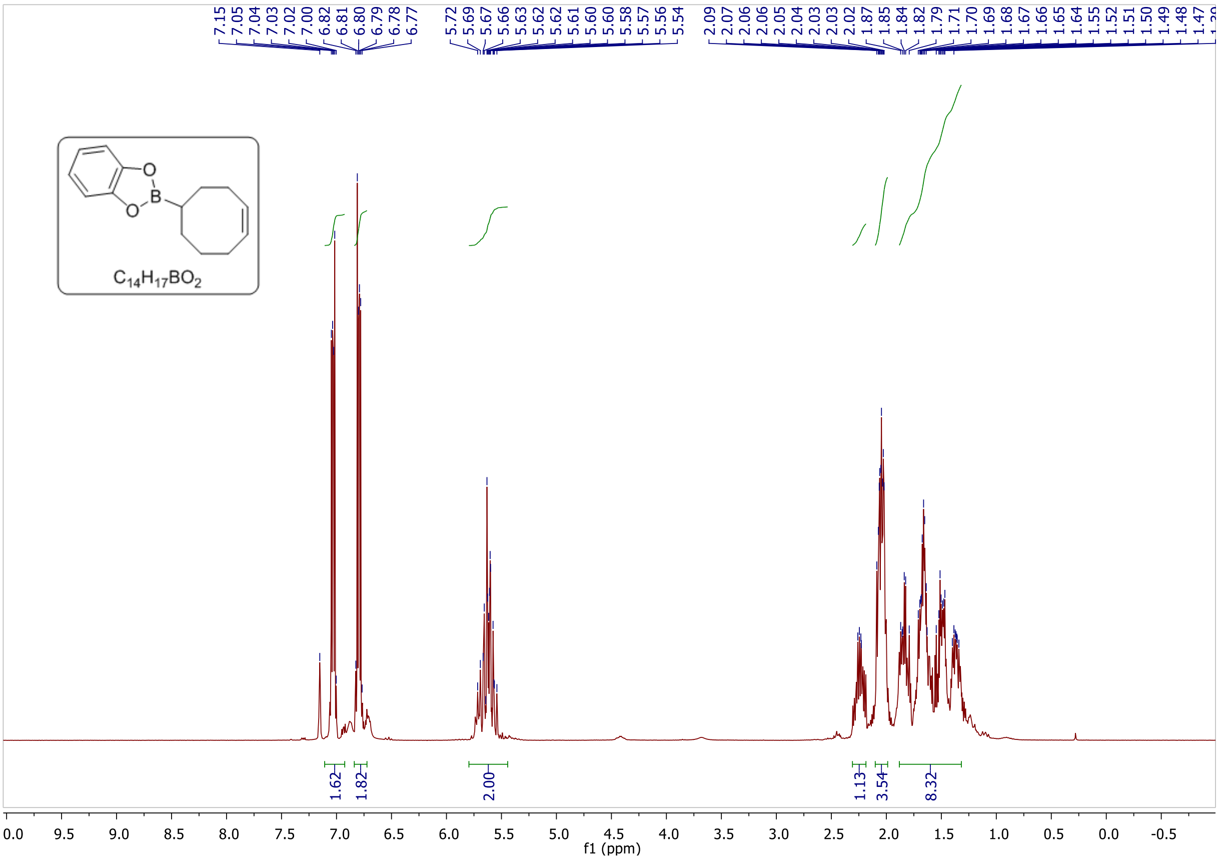

Supplementary Figure 200. ^{1}H-NMR (300 MHz) of **5**.

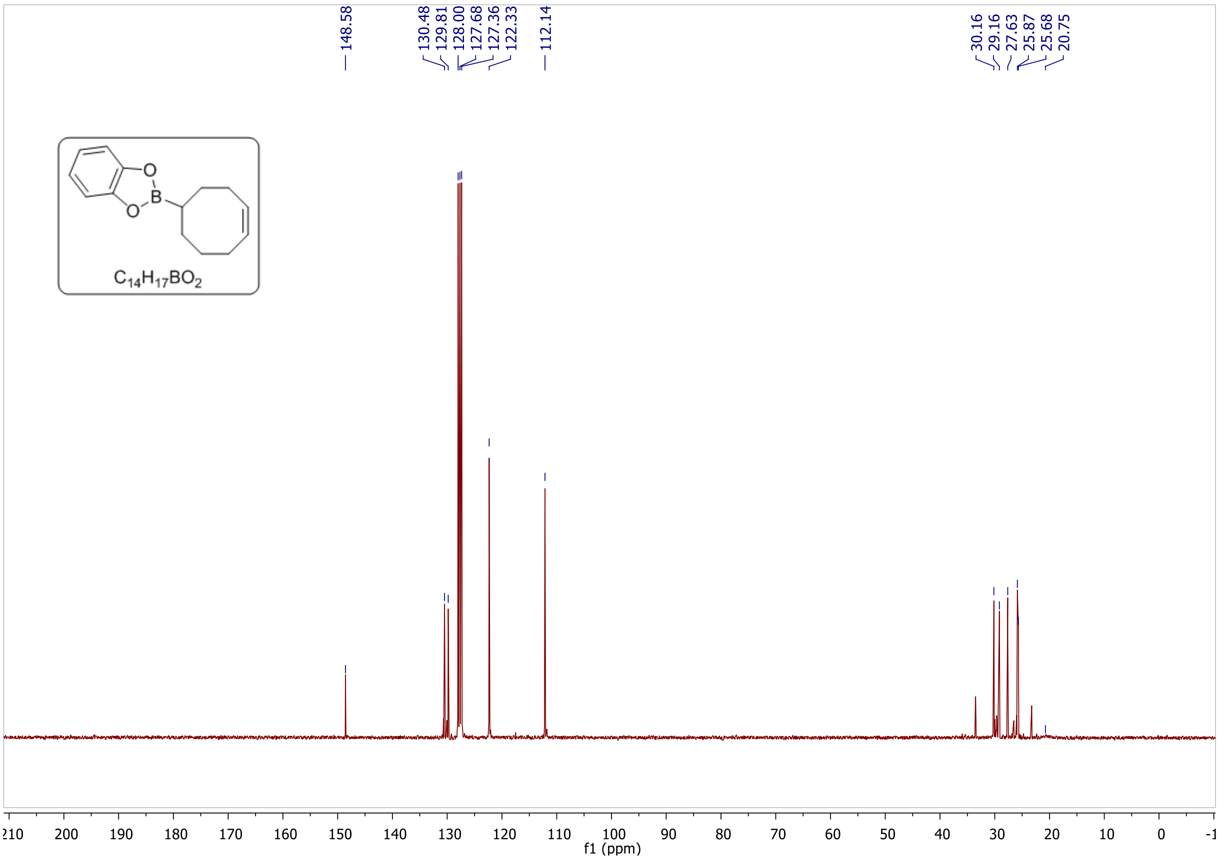

Supplementary Figure 201. ^{13}C-NMR (75 MHz) of **5**.

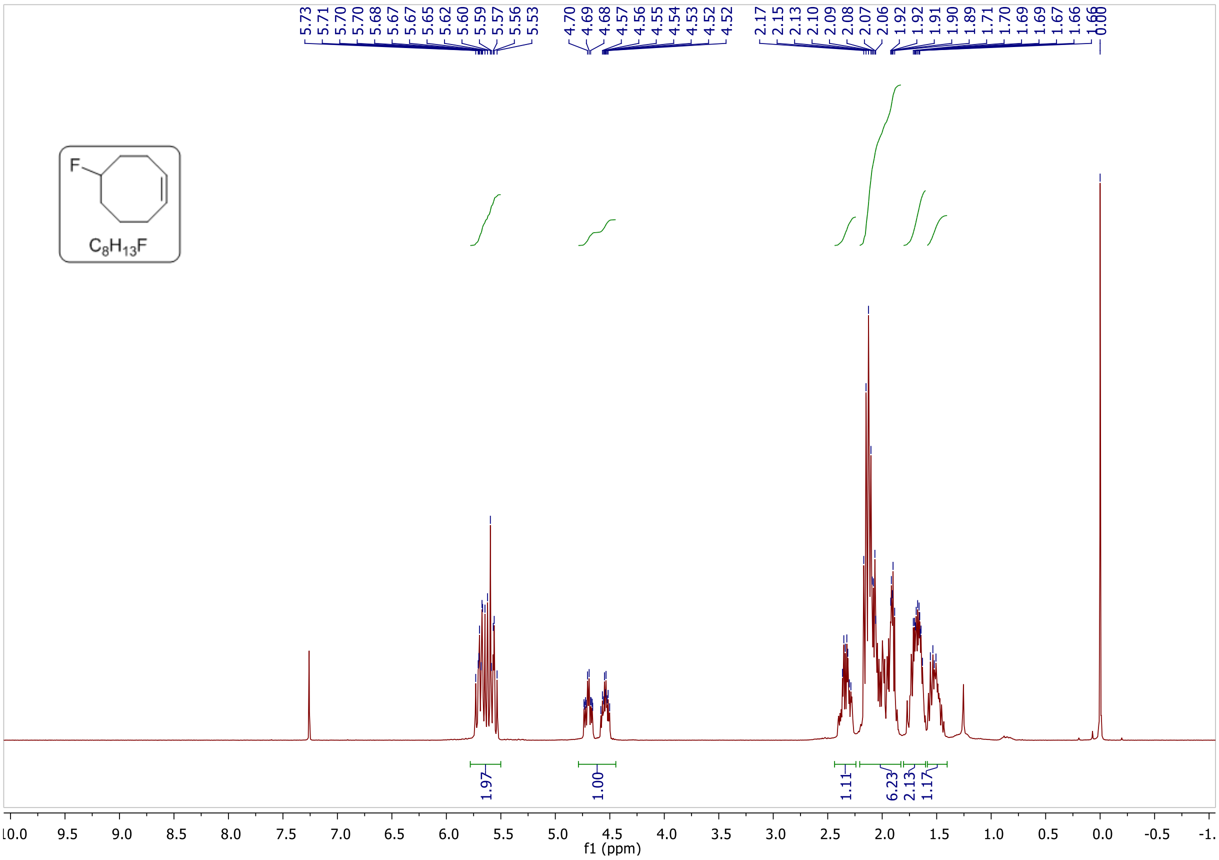

Supplementary Figure 202. ^{1}H-NMR (300 MHz) of **6**.

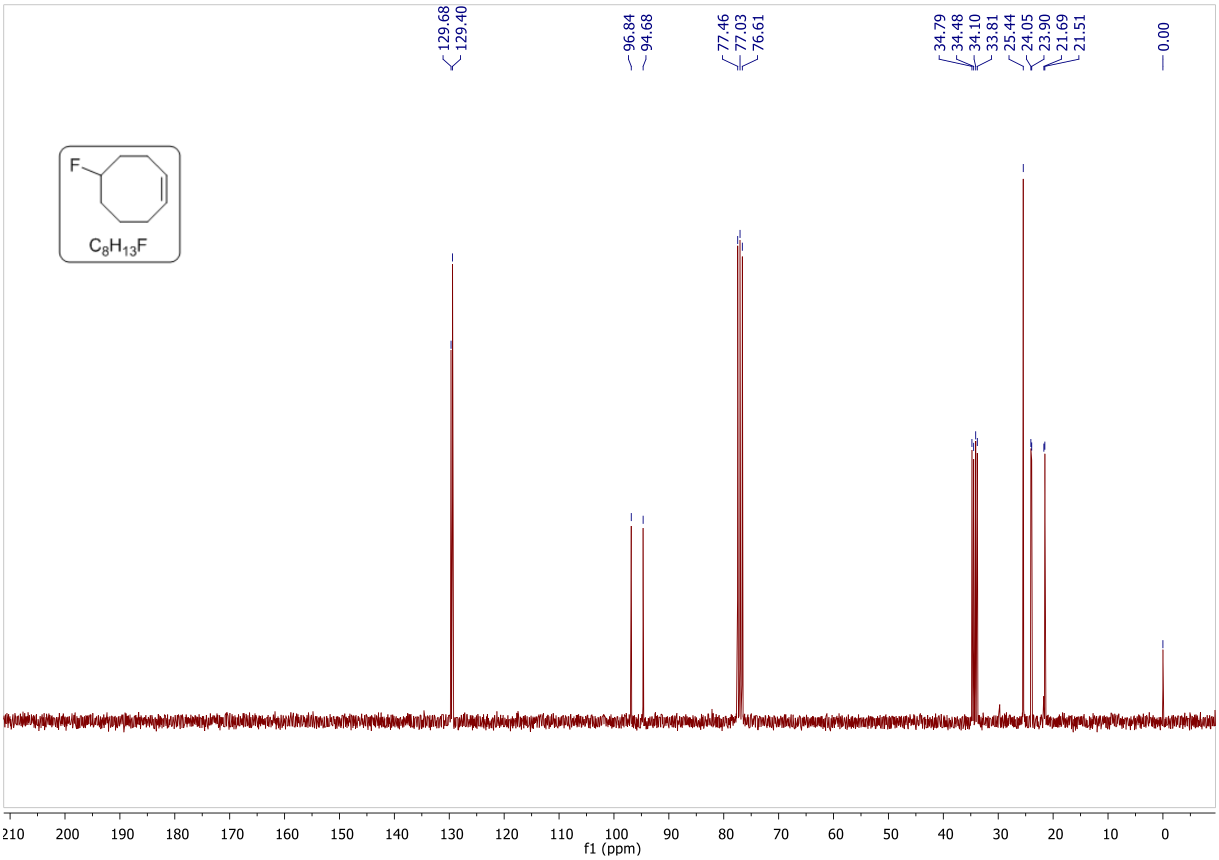

Supplementary Figure 203. ^{13}C-NMR (75 MHz) of **6**.

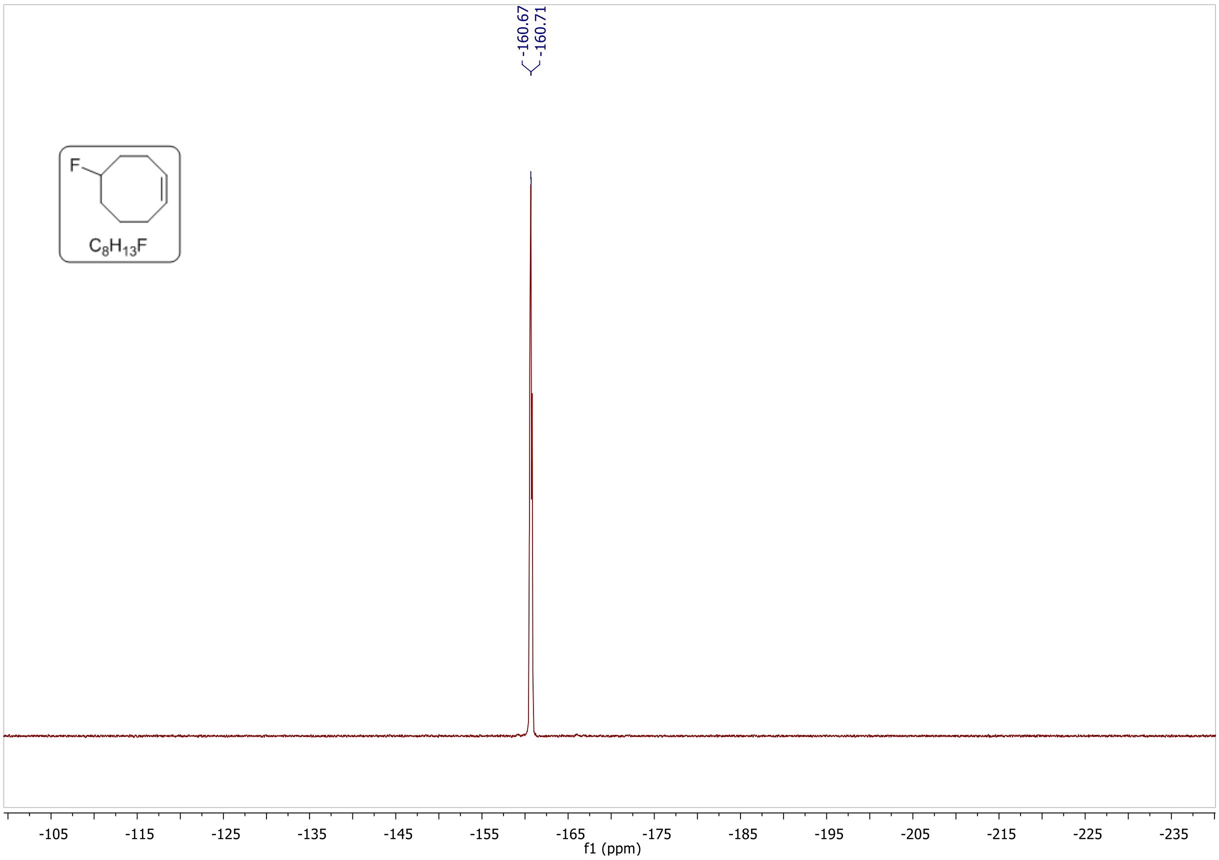

Supplementary Figure 204. ^{19}F-NMR (282 MHz) of **6**.

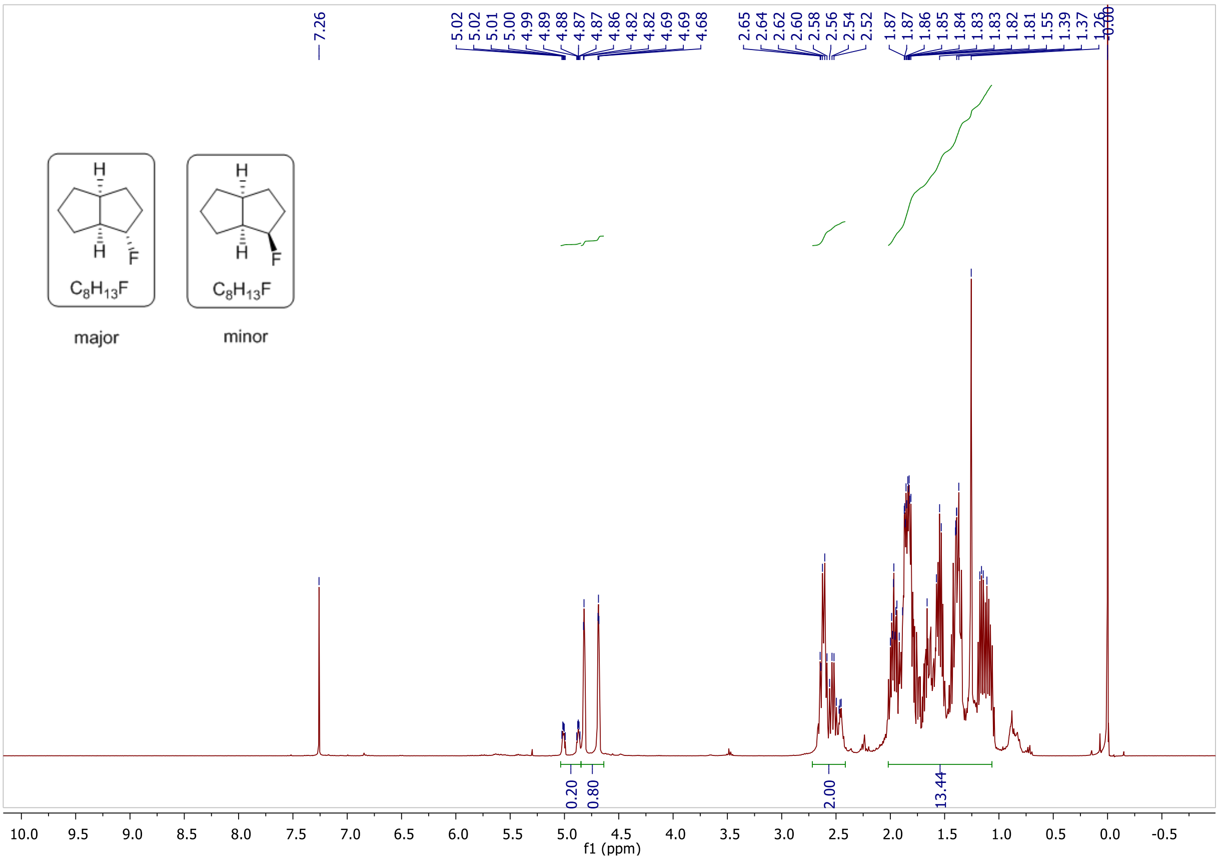

Supplementary Figure 205. ^{1}H-NMR (300 MHz) of **7**.

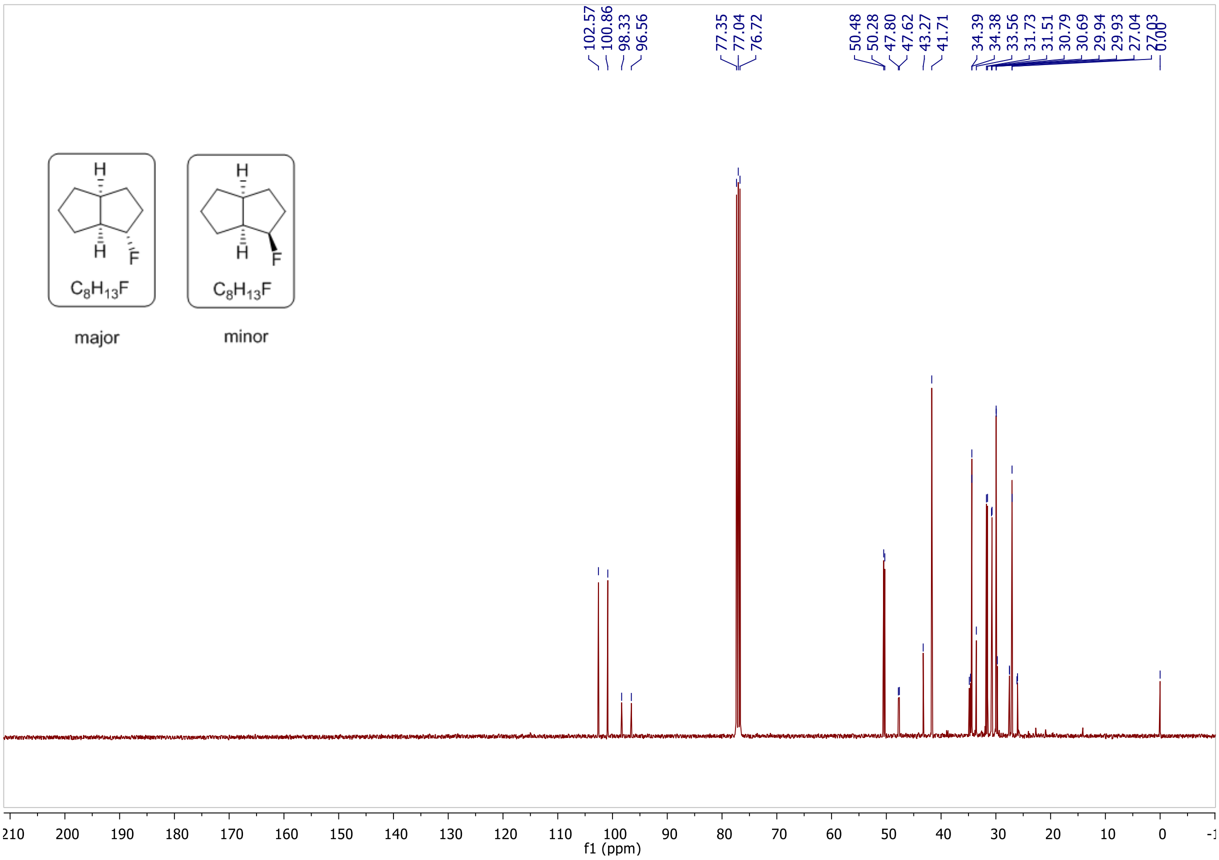

Supplementary Figure 206. ^{13}C-NMR (101 MHz) of **7**.

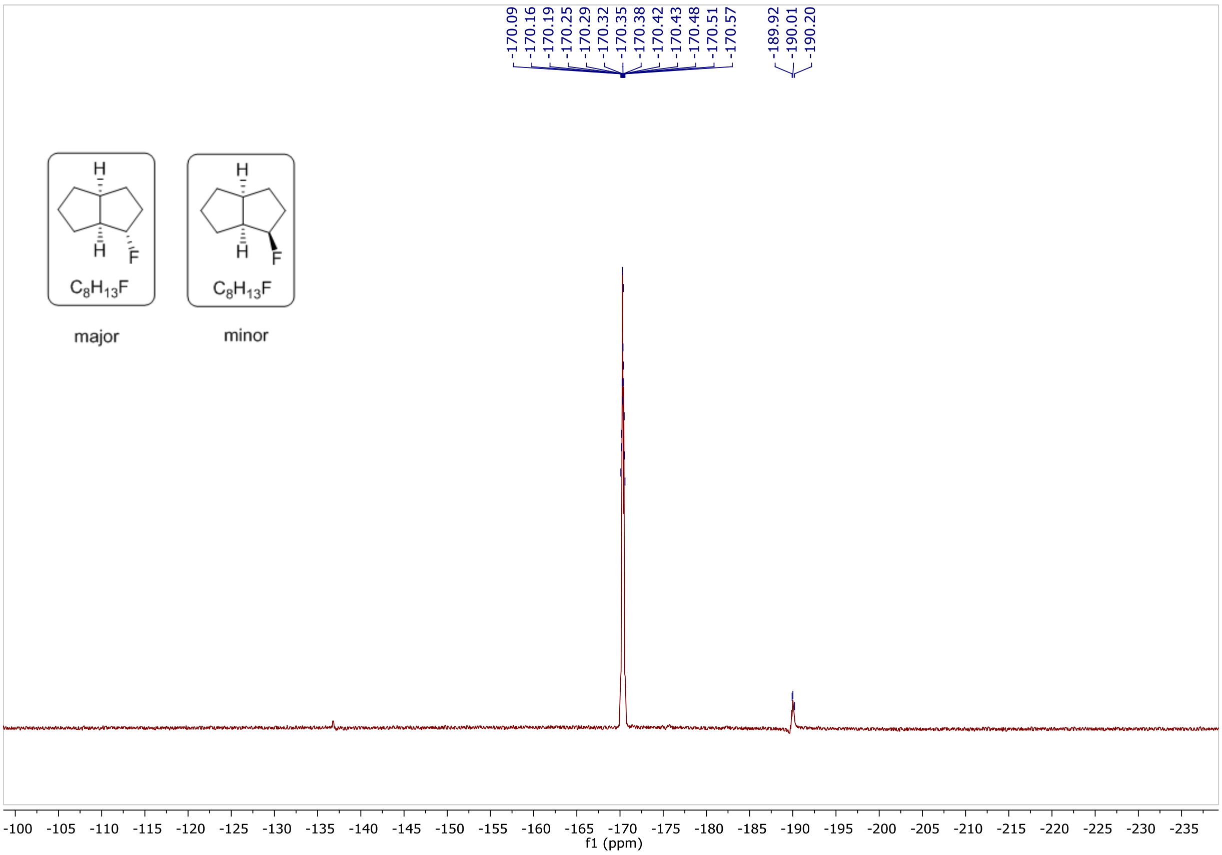

Supplementary Figure 207. ^{19}F-NMR (282 MHz) of **7**.

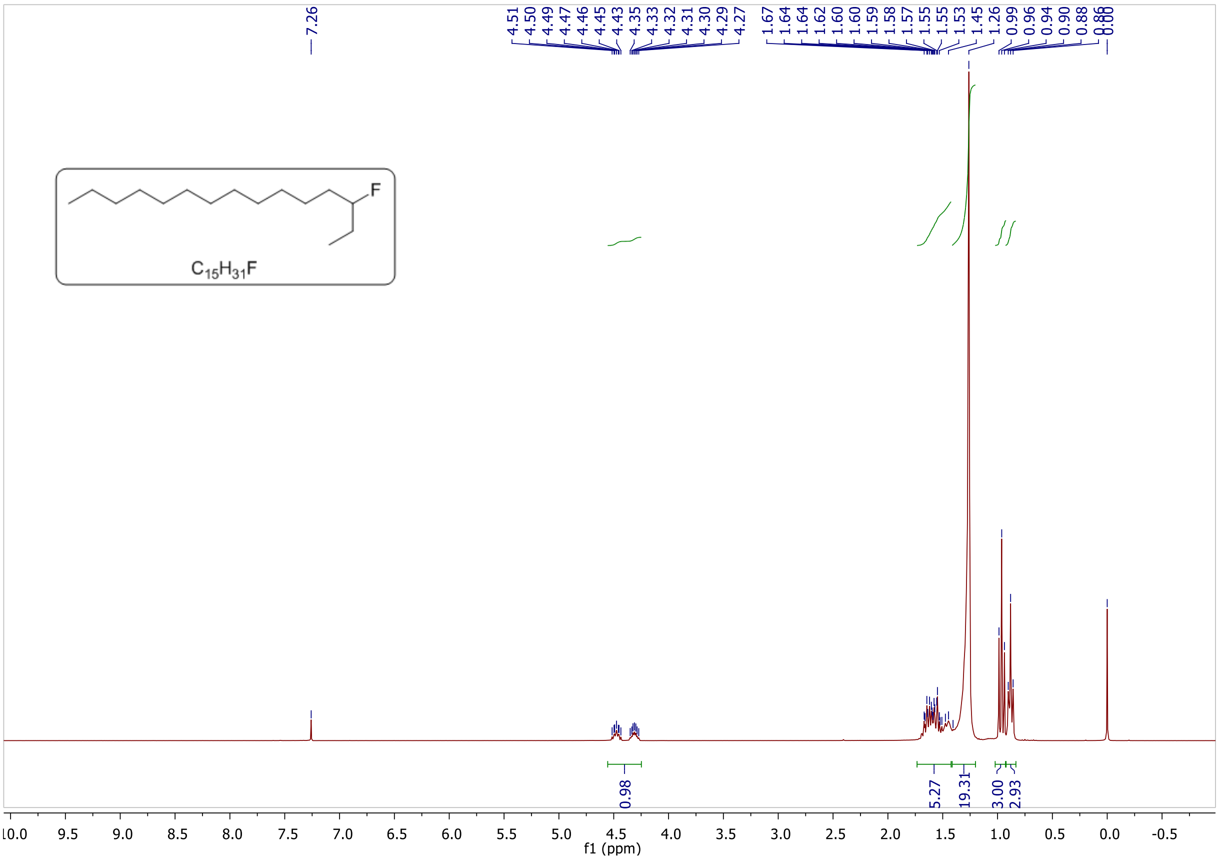

Supplementary Figure 208. ^{1}H-NMR (300 MHz) of **14a**.

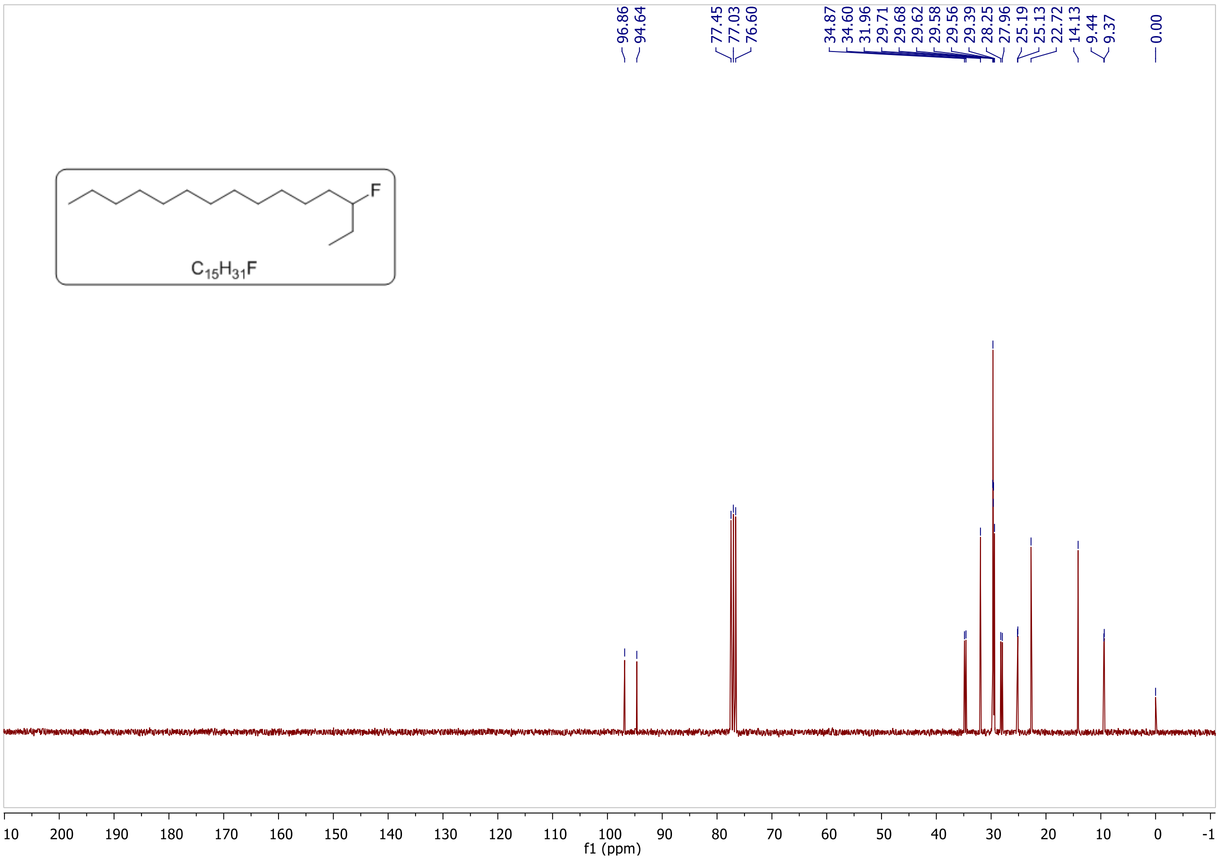

Supplementary Figure 209. ^{13}C-NMR (75 MHz) of **14a**.

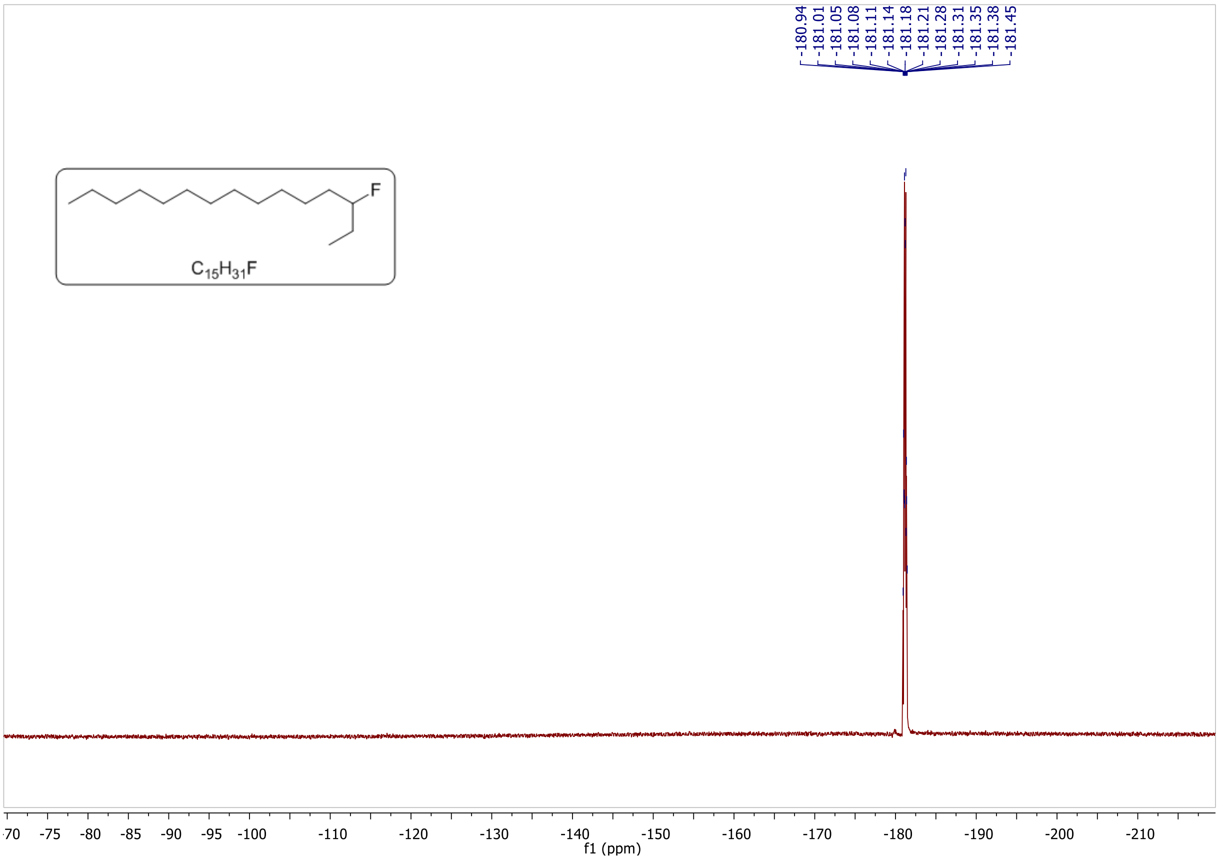

Supplementary Figure 210. ^{19}F-NMR (282 MHz) of **14a**.

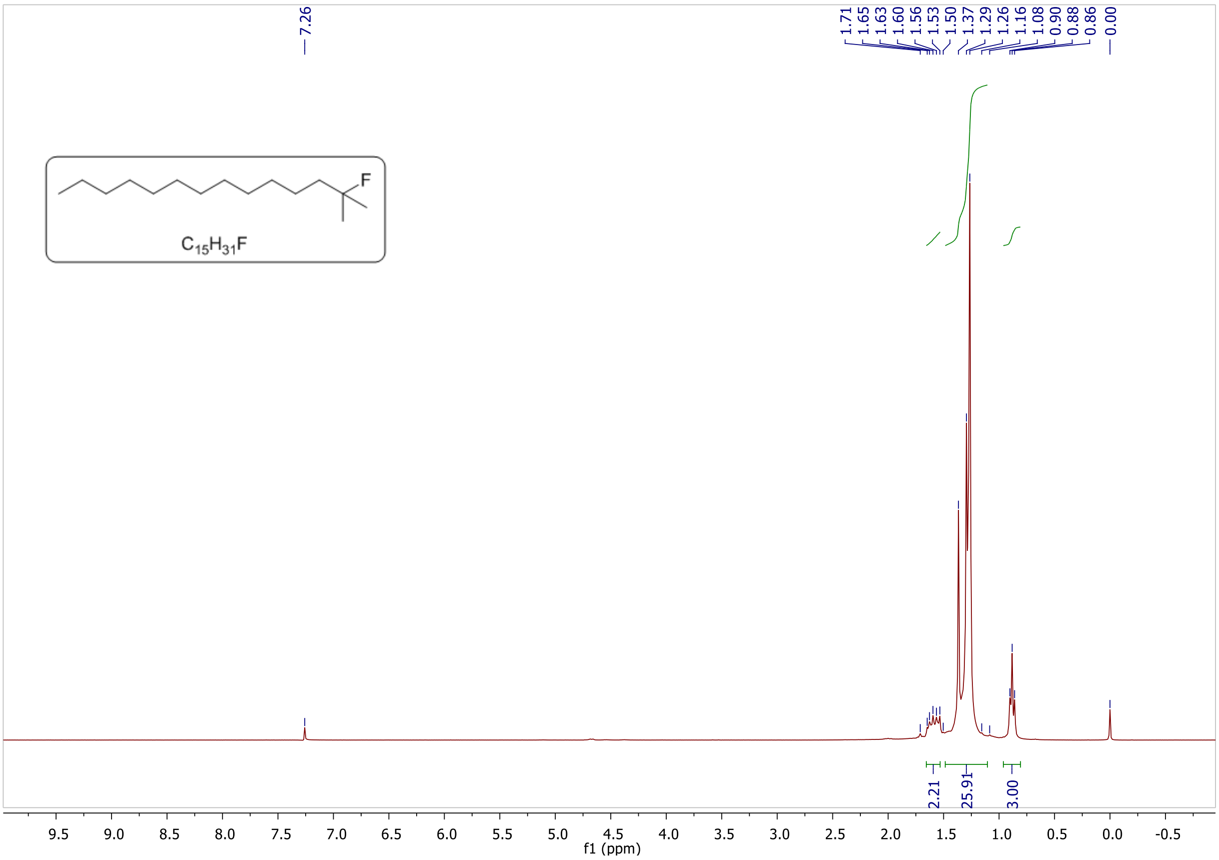

Supplementary Figure 211. ^{1}H-NMR (300 MHz) of **14b**.

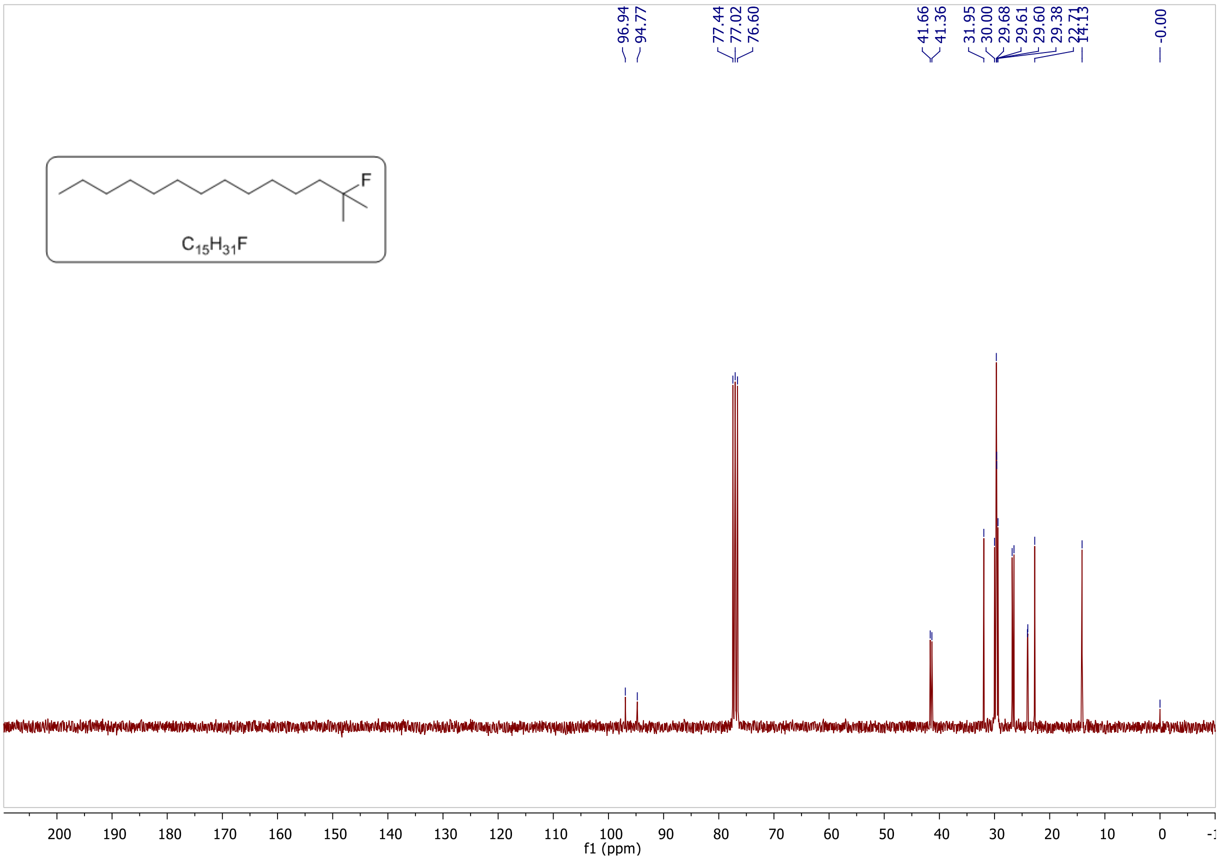

Supplementary Figure 212. ^{13}C-NMR (75 MHz) of **14b**.

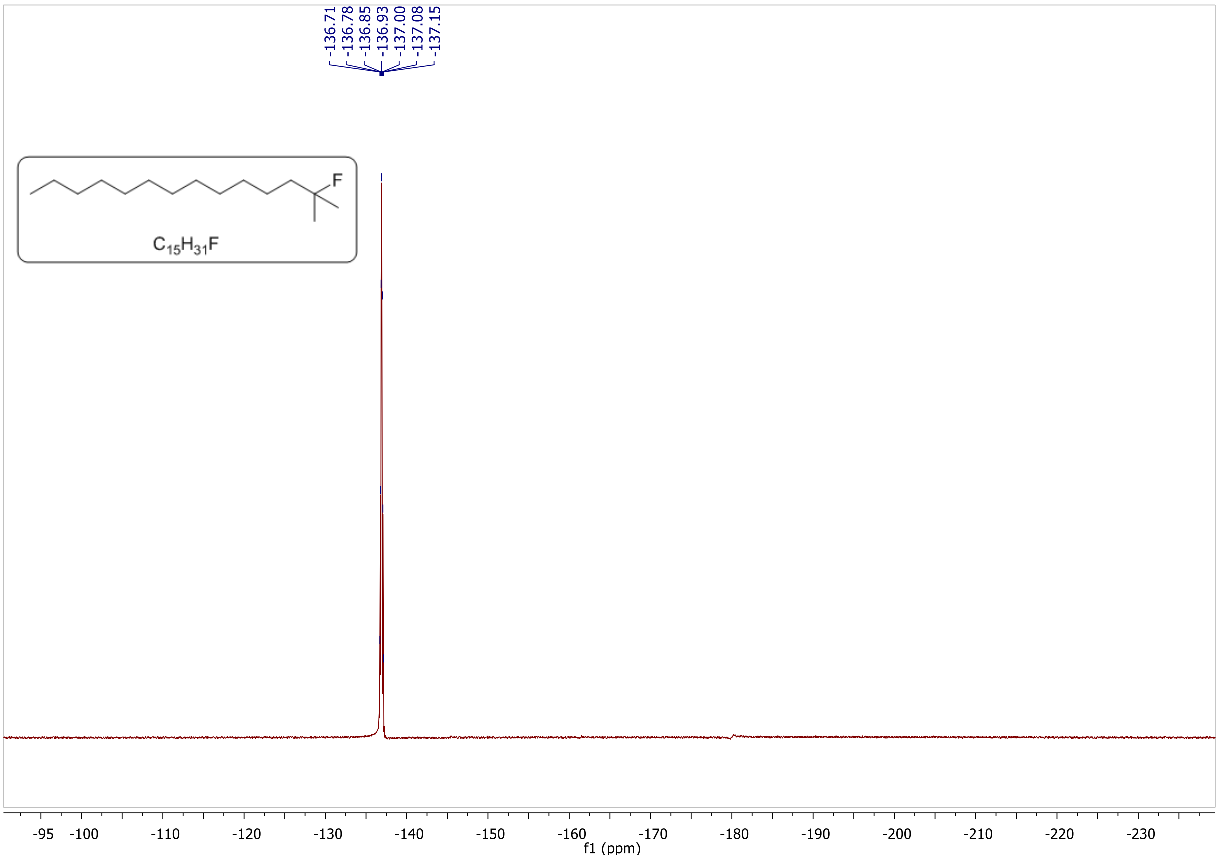

Supplementary Figure 213. ^{19}F-NMR (282 MHz) of **14b**.

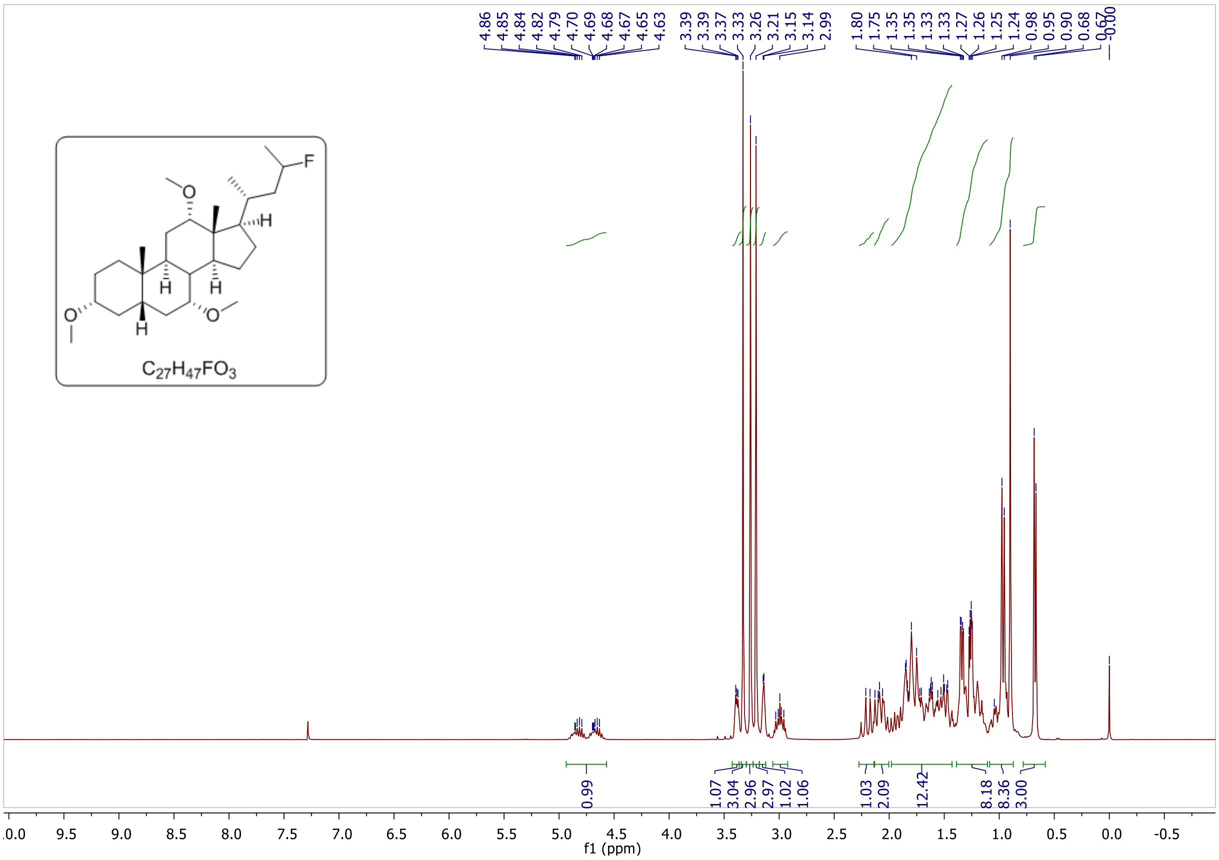

Supplementary Figure 214. ^{1}H-NMR (300 MHz) of **14c**.

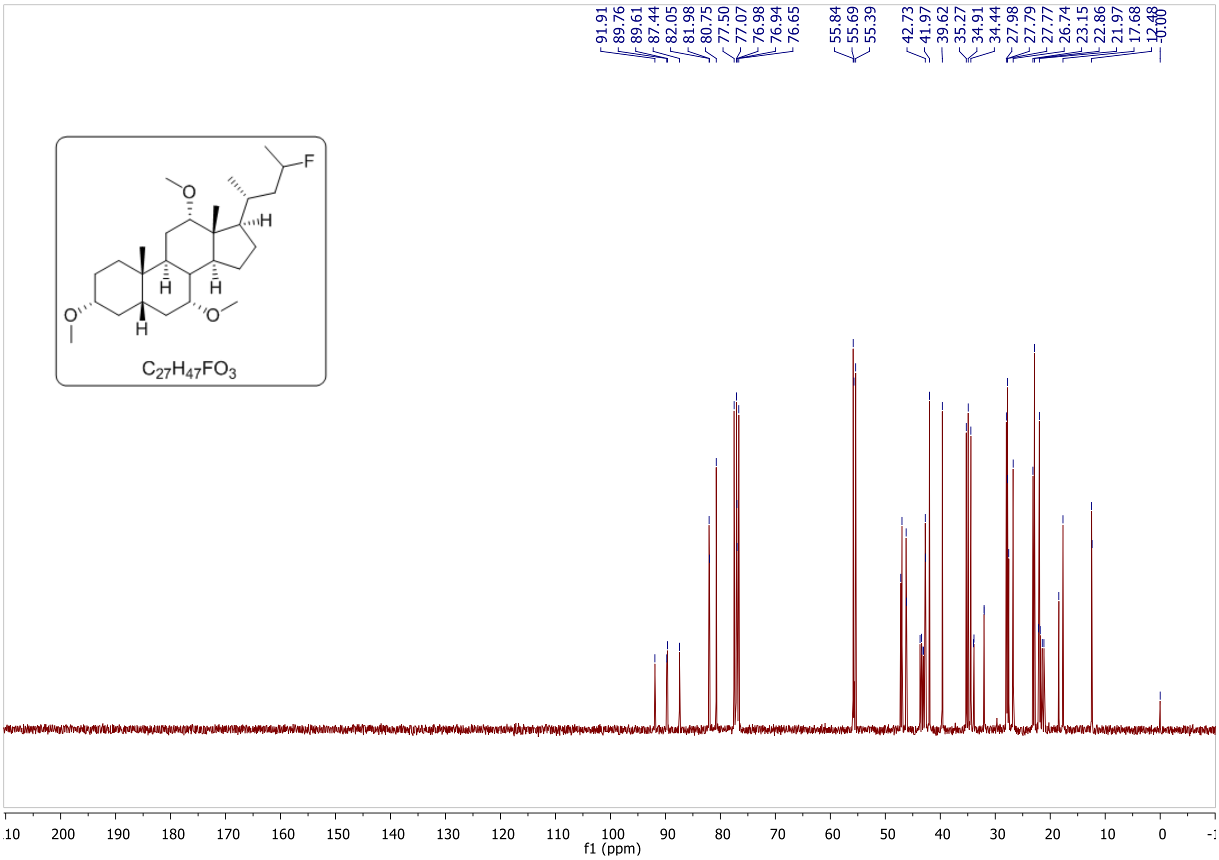

Supplementary Figure 215. ^{13}C-NMR (75 MHz) of **14c**.

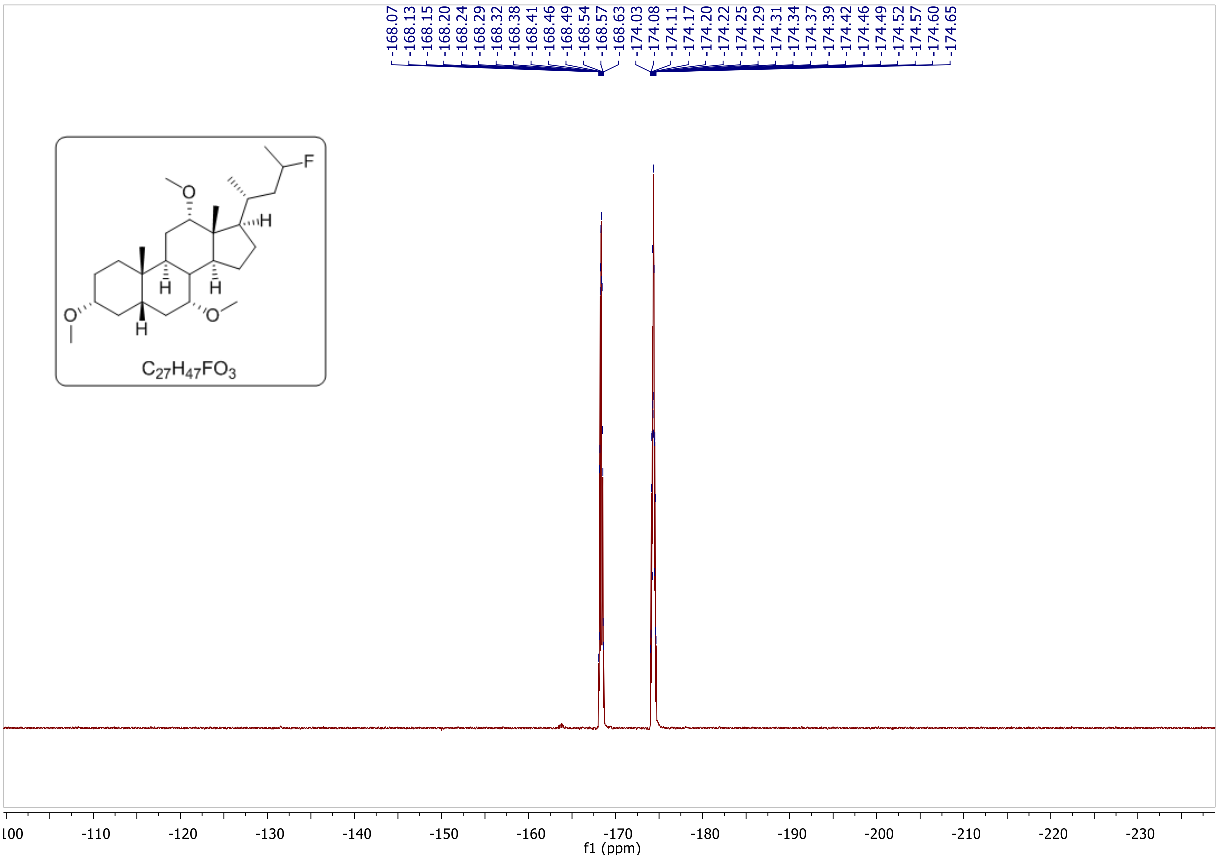

Supplementary Figure 216. ^{19}F-NMR (282 MHz) of **14c**.

Theoretical calculations

Methodology

As in previous studies on radical stabilities,[27-33] geometry optimizations of have been performed with a combination of the (U)B3LYP hybrid functional[34] and the 6-31G(d) basis set[35,36] in the gas phase. Thermochemical corrections (corr. ΔH & ΔG) to 298.15 K have been calculated at the same level of theory using the rigid rotor/harmonic oscillator model. Enthalpies (ΔH_{298}) and Gibbs energies (ΔG_{298}) at B3LYP/6-31G(d) level have been obtained through addition of corr. ΔG and corr. ΔH to ΔE_{tot} respectively. Improved single point total electronic energies (ΔE_{tot}) are obtained using the (RO)B2PLYP[37]/G3MP2Large and G3(MP2)-RAD scheme proposed by Radom et al..[38] Final enthalpies (ΔH_{298}) and Gibbs energies (ΔG_{298}) have been obtained through a combination of ΔE_{tot} calculated at (RO)B2PLYP/G3MP2Large and G3(MP2)-RAD with the thermochemical corrections (corr. ΔG and corr. ΔH) calculated at B3LYP/6-31G(d) level. A scaling factor of 0.9806 has been used for thermal correction to G3(MP2)-RAD scheme. Single point solvation energy (ΔG_{solv}) for DMF were calculated for gas phase optimized geometries using the SMD[39] continuum solvation model and subsequently added to gas phase energies to obtain solution phase energies that will be designated solution enthalpy ($\Delta H_{sol}=\Delta H_{298}+\Delta G_{solv}$) and solution free energies ($\Delta G_{sol}=\Delta G_{298}+\Delta G_{solv}$). In an alternative approached geometry optimization was carried out in the presence of the SMD continuum solvation model for DMF at (U)B3LYP/6-31G(d) level. The Gibbs energies calculated using the implicit DMF optimized geometry are designated as $\Delta G_{sol\text{-}opt}$.
Radical stabilization energies (RSE) for N-centered radicals generated by N-F homolytic bond cleavage in fluoroamines are measured with reference to fluoramine (H_2N-F) using the isodesmic fluorine exchange reaction shown in equ. 1a. R_2N-F bond dissociation energies (BDE) can then be derived from the calculated RSE values through the addition of the reference N-F BDE value in H_2N-F(286.6 kJ mol^{-1})[40] as expressed in equ. 1b.

$$R_2N{-}F \quad + \quad \bullet NH_2 \quad \xrightarrow{\mathbf{RSE}_{R_2N-F}} \quad R_2N\bullet \quad + \quad H_2N{-}F \qquad (1a)$$

$$\mathbf{BDE}_{R_2N-F} \quad = \quad RSE_{R_2N-F} \quad + \quad BDE^{Exp}_{H_2N-F} \qquad (1b)$$

Supplementary Figure 217. Radical stabilization energies of N-centered radicals.

In a completely analogous manner, N-H bond BDE values in the respective amines (R_2N-H) are calculated using NH_3 as a reference. BDE values for important reference molecules are listed in Supplementary Table 3.

Supplementary Table 3. Experimental and theoretical BDE values for reference molecules.

(Fluoro)Amine	BDE (kcal mol^{-1})	BDE (kJ mol^{-1})	Methods
F–NH_2	68.5	**286.6***	Derived from $\Delta_f H^0$ (NIST database)[40]
H–NH_2	107.57 ± 0.06	**450.08 ± 0.24***	Photolysis[41]
F–NH_2		291.7	W1w[42]
H–NH_2		450.3	W1w[42]

*Value used for reference molecule.

Potential energy surface (PES): Geometry optimizations for all the stationary points (minima, complexes and TS) along the PES have been performed at (U)B3LYP/6-31G(d) in the gas phase. Energy minima, complexes and TSs were confirmed by vibrational frequency calculation with 0, 0 and 1 imaginary frequencies, respectively. All stationary points were checked for wavefunction stability (stable=opt). The nature of transition states was further confirmed by IRC calculations [15 steps in both directions (reverse/forward) with stepsize=3] followed by geometry optimization to the minimum. In cases of very flat PES(s), manual displacement away from the TS(s) followed by geometry optimization was employed.
NBO charges were calculated using NBO6 module.[43] All calculations have been performed with Gaussian 09, revision D. 01.[44]

Structural Comparison (X-Ray vs QM)

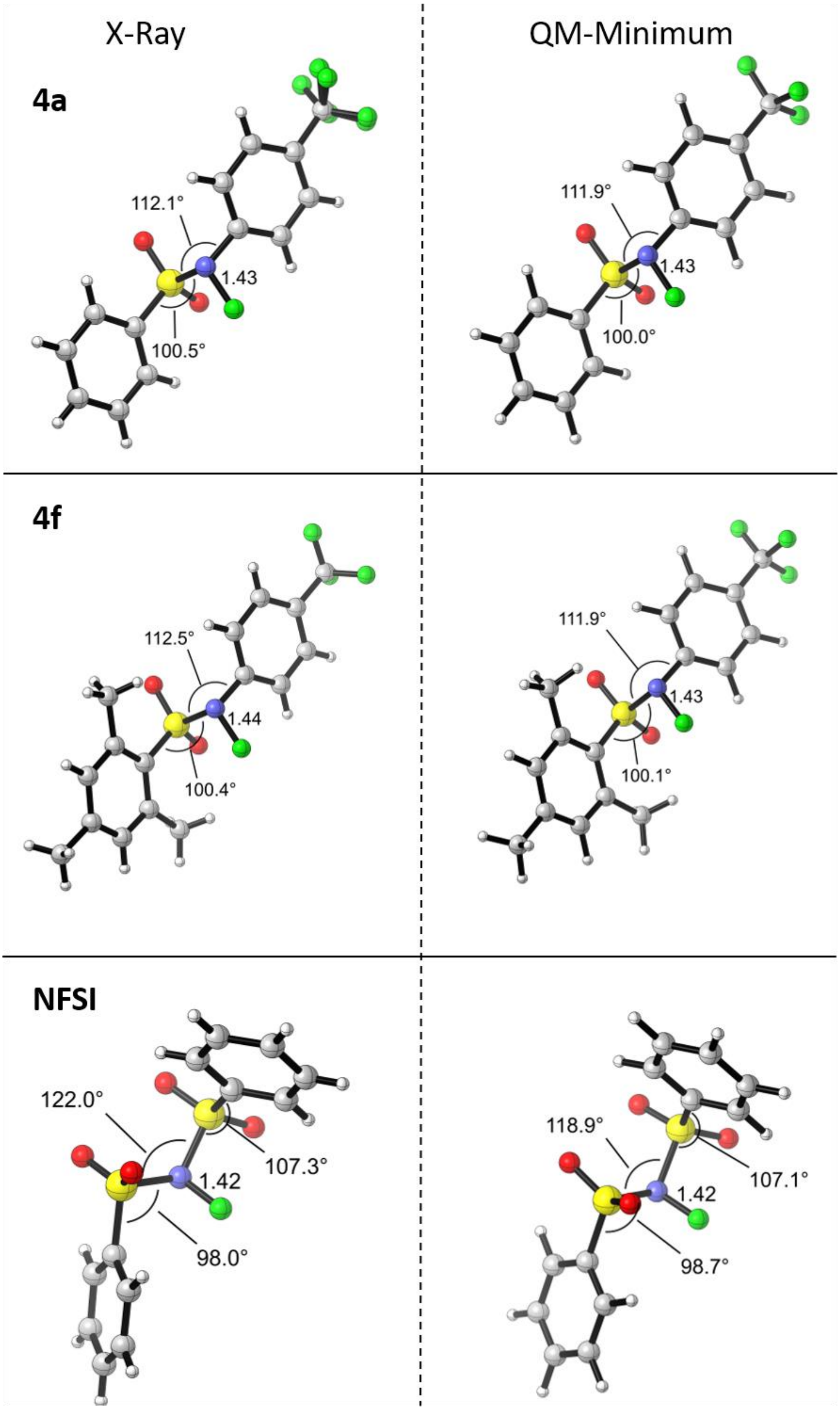

Supplementary Figure 218. Structural comparison between X-ray crystal structures and the corresponding gas phase QM minima obtained at the B3LYP/6-31G(d) level of theory.

Bond Strengths (F—NR$_2$)

(U)B3LYP/6-31G(d)

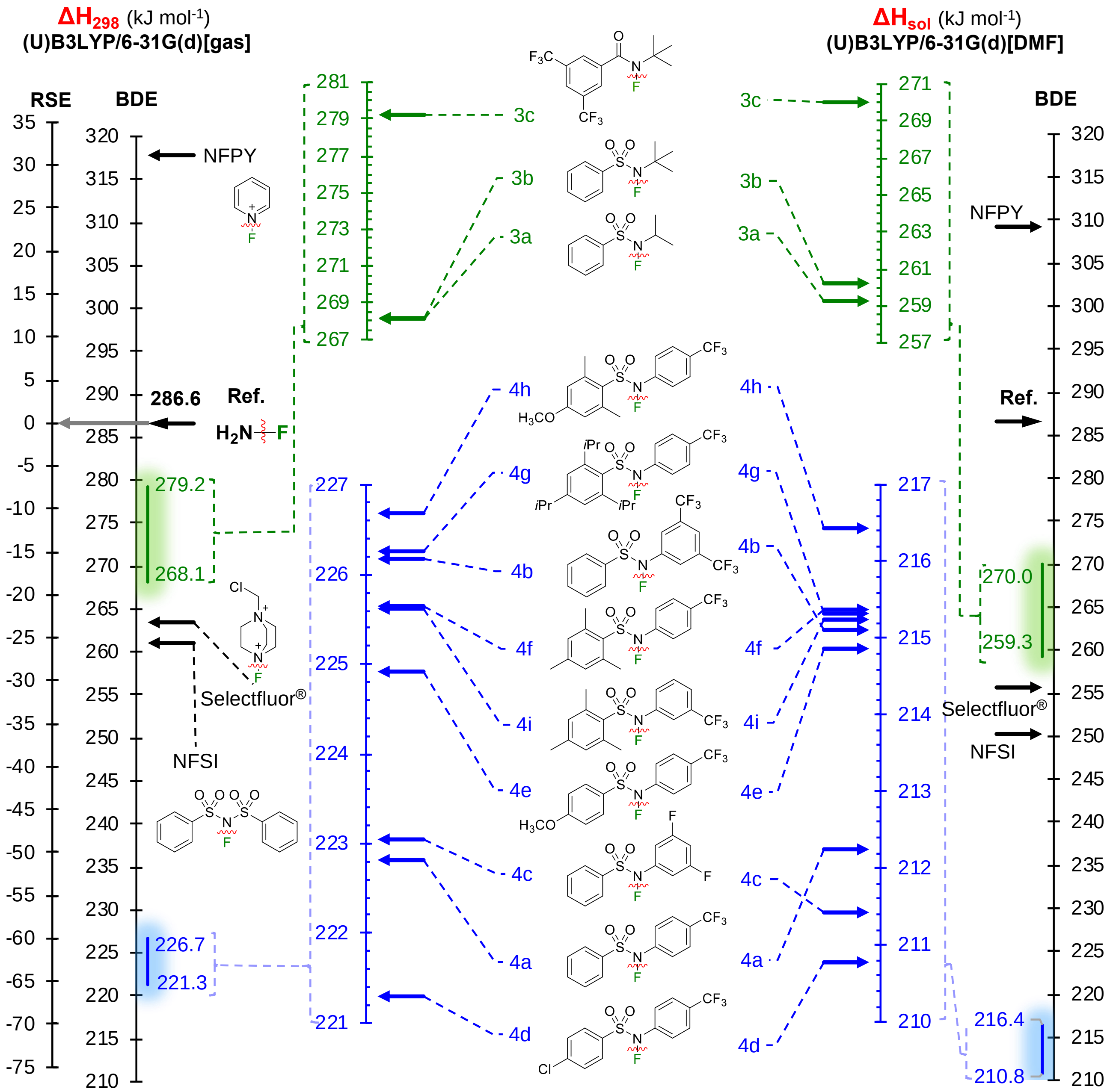

Supplementary Figure 219. Gas phase (ΔH_{298}) and solvation phase (DMF, $\Delta H_{sol}=\Delta H_{298}+\Delta G_{solv}$) **F—NR$_2$** bond dissociation energies (BDEs) and radical stabilization energies (RSEs) calculated at the (U)B3LYP/6-31G(d) level of theory. ΔG_{solv} (single point solvation energy) calculated at the SMD(DMF)/(U)B3LYP/6-31G(d)//(U)B3LYP/6-31G(d,p) level of theory.

(RO)B2PLYP/B2PLYP/G3MP2Large

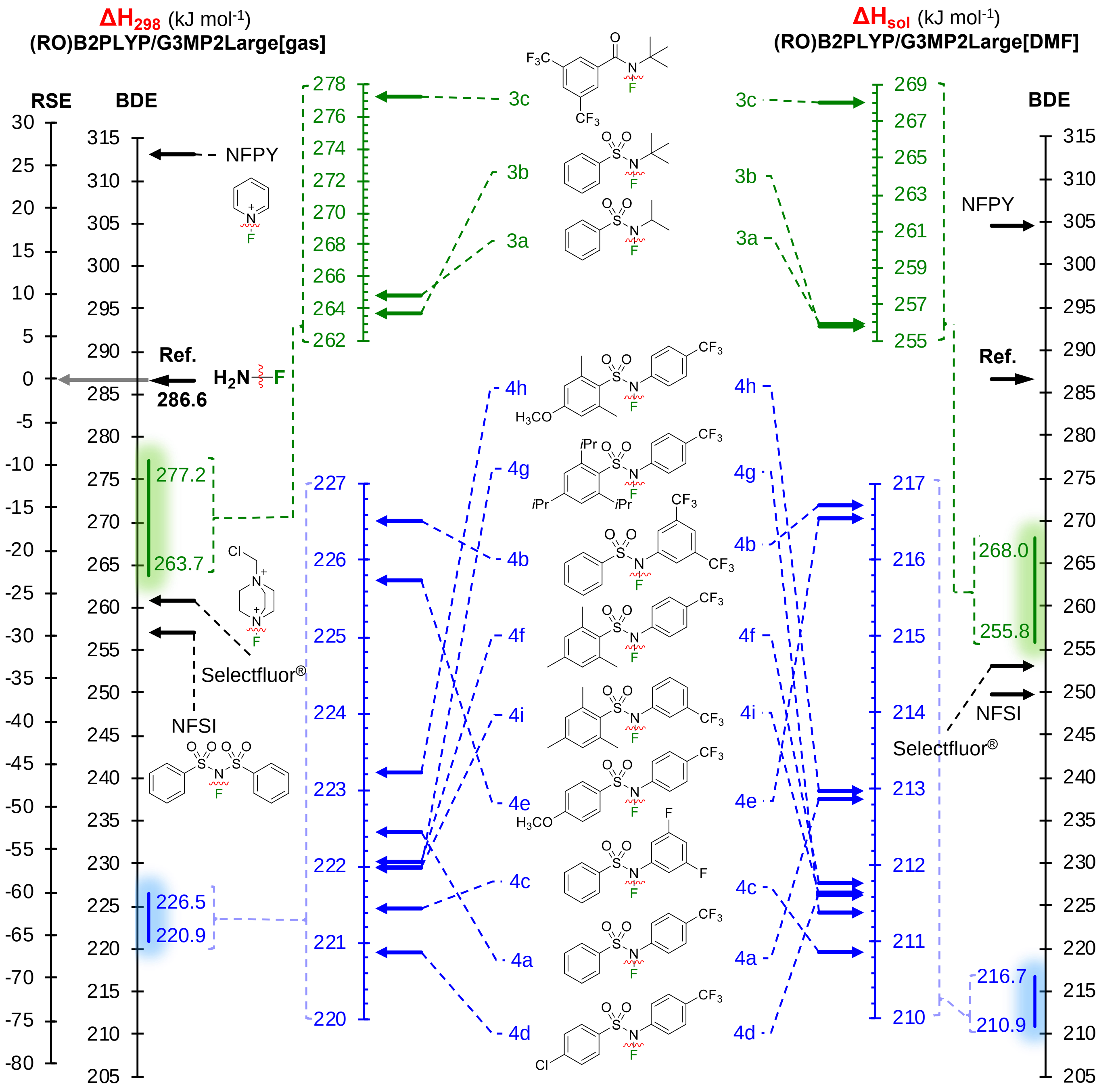

Supplementary Figure 220. Gas phase (ΔH_{298}) and solvation phase (DMF, $\Delta H_{sol}=\Delta H_{298}+\Delta G_{solv}$) **F—NR$_2$** bond dissociation energies (BDEs) and radical stabilization energies (RSEs) calculated at the (RO)B2PLYP/G3MP2Large//(U)B3LYP/6-31G(d). ΔG_{solv} (single point solvation energy) calculated at the SMD(DMF)/(U)B3LYP/6-31G(d)//(U)B3LYP/6-31G(d,p) level of theory.

G3(MP2)-RAD

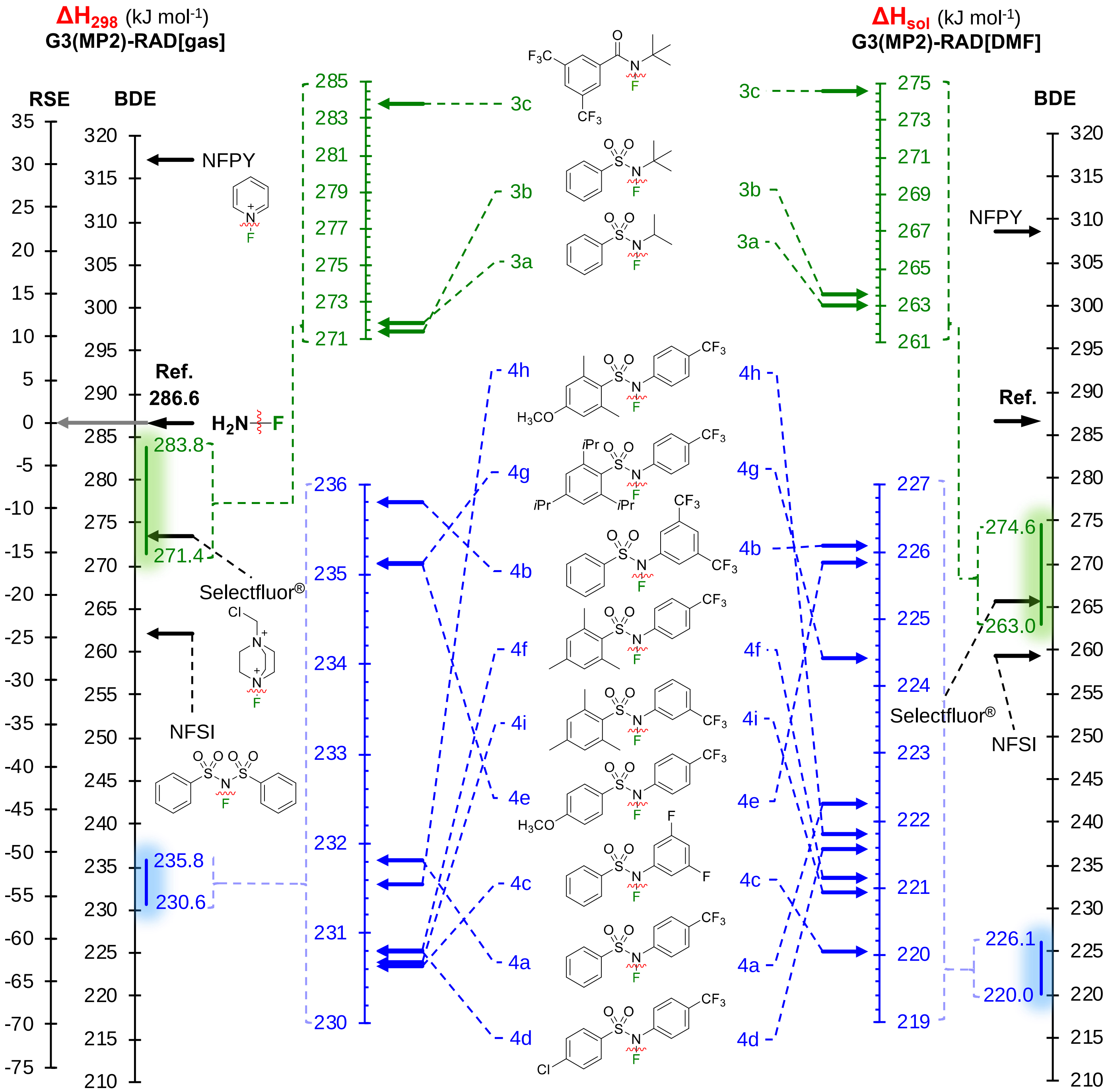

Supplementary Figure 221. Gas phase (ΔH_{298}) and solvation phase (DMF, $\Delta H_{sol}=\Delta H_{298}+\Delta G_{solv}$) **F—NR$_2$** bond dissociation energies (BDEs) and radical stabilization energies (RSEs) calculated at the G3(MP2)-RAD level of theory. ΔG_{solv} (single point solvation energy) calculated at the SMD(DMF)/(U)B3LYP/6-31G(d)//(U)B3LYP/6-31G(d,p) level of theory.

Supplementary Table 4. Gas phase (ΔH_{298}) **F—NR₂** bond dissociation energies (BDSs, in kJ mol^{-1}) for the systems listed in Supplementary Figure 219-221 at different levels of theory.

	(U)B3LYP/6-31G(d)[gas]		(RO)B2PLYP/G3MP2Large[gas]		G3(MP2)-RAD[gas]	
	**Best Conf.**	**Boltzmann Avg.**	**Best Conf.**	**Boltzmann Avg.**	**Best Conf.**	**Boltzmann Avg.**
	**ΔH_{298}**	**ΔH_{298}**	**ΔH_{298}**	**ΔH_{298}**	**ΔH_{298}**	**ΔH_{298}**
**NH_2—F**	**286.6**		**286.6**		**286.6**	
**3a**	268.1	267.8	264.8	264.2	271.9	271.0
**3b**	268.2	268.0	263.7	263.5	271.4	271.1
**3c**	278.8	278.8	276.6	276.6	283.8	283.8
**4a**	222.8	222.3	222.4	221.7	231.8	231.5
**4b**	226.2	225.4	226.5	225.9	235.8	235.4
**4c**	223.0	222.6	221.5	220.8	230.6	230.3
**4d**	221.3	220.8	220.9	220.2	230.8	230.4
**4e**	224.9	224.4	225.7	225.3	235.1	234.8
**4f**	225.6	225.2	222.1	221.3	230.8	230.1
**4g**	226.3	225.1	222.0	220.9	235.1	233.8
**4h**	226.7	226.3	223.2	222.9	231.5	231.8
**4i**	225.6	225.0	222.0	221.0	230.7	230.3
**NFSI**	261.0	261.1	257.1	256.4	262.1	261.1
**Selectfluor®**	263.5	263.5	260.8	260.8	273.5	273.5
**NFPY**	317.8	317.8	313.1	313.1	317.2	317.2

Supplementary Table 5. Gas phase **F—NR₂** radical stabilization energies (RSEs, in kJ mol^{-1}) for the systems listed in Supplementary Figure 219-221 at different levels of theory.

	(U)B3LYP/6-31G(d)[gas]						(RO)B2PLYP/G3MP2Large[gas]						G3(MP2)-RAD[gas]					
	**Best Conf.**			**Boltzmann Avg.**			**Best Conf.**			**Boltzmann Avg.**			**Best Conf.**			**Boltzmann Avg.**		
	**ΔE_{tot}**	**ΔH_{298}**	**ΔG_{298}**	**ΔE_{tot}**	**ΔH_{298}**	**ΔG_{298}**	**ΔE_{tot}**	**ΔH_{298}**	**ΔG_{298}**	**ΔE_{tot}**	**ΔH_{298}**	**ΔG_{298}**	**ΔE_{tot}**	**ΔH_{298}**	**ΔG_{298}**	**ΔE_{tot}**	**ΔH_{298}**	**ΔG_{298}**
**3a**	-27.8	**-18.5**	-28.9	-28.2	**-18.8**	-29.1	-31.1	**-21.8**	-32.2	-31.8	**-22.4**	-32.5	-23.8	**-14.7**	-25.1	-24.7	**-15.6**	-25.6
**3b**	-27.7	**-18.4**	-25.6	-27.8	**-18.6**	-25.2	-32.1	**-22.9**	-30.0	-32.4	**-23.1**	-29.7	-24.1	**-15.2**	-22.3	-24.6	**-15.5**	-22.0
**3c**	-15.7	**-7.8**	-20.8	-15.7	**-7.8**	-20.4	-17.9	**-10.0**	-23.0	-17.9	**-10.0**	-22.6	-10.4	**-2.8**	-12.0	-10.4	**-2.8**	-12.0
**4a**	-73.9	**-63.8**	-70.8	-74.2	**-64.3**	-71.5	-73.8	**-64.2**	-75.2	-74.5	**-64.9**	-75.6	-64.2	**-54.8**	-66.1	-64.5	**-55.1**	-67.0
**4b**	-70.1	**-60.4**	-70.6	-70.8	**-61.2**	-71.3	-69.5	**-60.1**	-72.8	-70.1	**-60.7**	-72.9	-60.0	**-50.8**	-63.3	-60.3	**-51.2**	-63.4
**4c**	-73.2	**-63.6**	-70.1	-73.5	**-64.0**	-70.8	-74.5	**-65.1**	-74.4	-75.1	**-65.8**	-74.9	-65.0	**-56.0**	-65.1	-65.3	**-56.3**	-65.7
**4d**	-75.5	**-65.3**	-71.9	-75.9	**-65.8**	-72.5	-75.3	**-65.7**	-76.2	-76.1	**-66.4**	-76.7	-65.2	**-55.8**	-67.3	-65.5	**-56.2**	-68.0
**4e**	-71.9	**-61.7**	-68.7	-72.1	**-62.2**	-69.7	-70.6	**-60.9**	-71.5	-71.0	**-61.3**	-71.9	-60.9	**-51.5**	-62.1	-61.2	**-51.8**	-62.9
**4f**	-71.1	**-61.0**	-66.2	-71.6	**-61.4**	-66.5	-74.7	**-64.5**	-69.8	-75.3	**-65.3**	-70.3	-65.0	**-55.8**	-63.5	-65.7	**-56.5**	-64.4
**4g**	-70.5	**-60.3**	-69.3	-71.4	**-61.5**	-69.6	-74.8	**-64.6**	-73.5	-75.5	**-65.7**	-74.3	-60.6	**-51.5**	-65.3	-61.9	**-52.8**	-65.8
**4h**	-69.9	**-59.9**	-68.5	-70.4	**-60.3**	-67.8	-73.3	**-63.4**	-71.6	-73.6	**-63.7**	-71.5	-64.1	**-55.1**	-65.6	-64.0	**-54.8**	-65.6
**4i**	-71.1	**-61.0**	-66.9	-71.8	**-61.6**	-66.9	-74.7	**-64.6**	-70.5	-75.5	**-65.6**	-71.1	-65.1	**-55.9**	-64.3	-65.5	**-56.3**	-65.1
**NFSI**	-35.5	**-25.6**	-31.4	-35.3	**-25.5**	-32.3	-39.4	**-29.5**	-35.3	-40.1	**-30.2**	-35.4	-34.1	**-24.5**	-30.2	-35.2	**-25.5**	-30.7
**Selectfluor®**	-29.8	**-23.1**	-31.3	-29.8	**-23.1**	-31.3	-32.5	**-25.8**	-33.9	-32.5	**-25.8**	-33.9	-19.6	**-13.1**	-21.2	-19.6	**-13.1**	-21.2
**NFPY**	27.4	**31.2**	23.5	27.4	**31.2**	23.5	22.7	**26.5**	18.8	22.7	**26.5**	18.8	26.9	**30.6**	22.9	26.9	**30.6**	22.9

Supplementary Table 6. Solvation phase (DMF, $\Delta H_{sol}=\Delta H_{298}+\Delta G_{solv}$) **F—NR$_2$** bond dissociation energies (BDEs, in kJ mol^{-1}) for the systems listed in Supplementary Table 4 at different levels of theory. ΔG_{solv} (single point solvation energy) calculated at the SMD(DMF)/(U)B3LYP/6-31G(d)//(U)B3LYP/6-31G(d,p) level of theory.

	**(U)B3LYP/6-31G(d)[DMF]**		**(RO)B2PLYP/G3MP2Large[DMF]**		**G3(MP2)-RAD[DMF]**	
	**Best Conf.**	**Boltzmann Avg.**	**Best Conf.**	**Boltzmann Avg.**	**Best Conf.**	**Boltzmann Avg.**
	**ΔH_{sol}**	**ΔH_{sol}**	**ΔH_{sol}**	**ΔH_{sol}**	**ΔH_{sol}**	**ΔH_{sol}**
**NH_2—F**	**286.6**		**286.6**		**286.6**	
**3a**	259.3	258.3	256.0	255.1	263.0	262.6
**3b**	260.2	259.7	255.8	255.2	263.6	263.2
**3c**	270.0	270.1	268.0	268.1	274.6	274.1
**4a**	212.2	212.1	212.9	212.3	222.3	222.0
**4b**	215.1	215.0	216.7	216.4	226.1	225.9
**4c**	211.4	211.4	210.9	210.3	220.0	219.8
**4d**	210.8	210.6	211.6	211.1	221.6	221.4
**4e**	214.9	214.7	216.5	216.2	225.8	225.7
**4f**	215.4	215.0	211.8	211.2	221.1	220.4
**4g**	215.3	214.5	211.4	210.5	224.4	223.5
**4h**	216.4	216.2	213.0	212.8	221.8	221.9
**4i**	215.2	214.6	211.6	210.8	220.9	220.5
**NFSI**	250.3	249.6	249.7	249.0	259.3	259.1
**Selectfluor®**	255.7	255.7	253.0	253.0	265.7	265.7
**NFPY**	309.2	309.2	304.5	304.5	308.6	308.6

Supplementary Table 7. Solvation phase (DMF) **F—NR$_2$** radical stabilization energies (RSEs, in kJ mol^{-1}) for the systems listed in Supplementary Table 5 at different levels of theory.

	**(U)B3LYP/6-31G(d)[DMF]**						**(RO)B2PLYP/G3MP2Large[DMF]**						**G3(MP2)-RAD[DMF]**					
	**Best Conf.**			**Boltzmann Avg.**			**Best Conf.**			**Boltzmann Avg.**			**Best Conf.**			**Boltzmann Avg.**		
	**ΔE_{sol}[a]**	**ΔH_{sol}[b]**	**ΔG_{sol}[c]**	**ΔE_{sol}**	**ΔH_{sol}**	**ΔG_{sol}**	**ΔE_{sol}**	**ΔH_{sol}**	**ΔG_{sol}**	**ΔE_{sol}**	**ΔH_{sol}**	**ΔG_{sol}**	**ΔE_{sol}**	**ΔH_{sol}**	**ΔG_{sol}**	**ΔE_{sol}**	**ΔH_{sol}**	**ΔG_{sol}**
**3a**	-36.7	**-27.3**	-37.7	-37.7	**-28.3**	-38.3	-40.0	**-30.6**	-41.0	-40.8	**-31.5**	-41.8	-32.5	**-23.6**	-33.9	-33.0	**-24.0**	-34.9
**3b**	-35.7	**-26.4**	-33.4	-36.1	**-26.9**	-33.3	-40.1	**-30.8**	-37.8	-40.6	**-31.4**	-37.9	-32.0	**-23.0**	-30.1	-32.3	**-23.4**	-30.3
**3c**	-24.4	**-16.6**	-25.7	-24.3	**-16.5**	-25.7	-26.4	**-18.6**	-27.9	-26.3	**-18.5**	-27.8	-19.6	**-12.0**	-21.2	-20.2	**-12.5**	-21.8
**4a**	-84.0	**-74.4**	-81.5	-84.3	**-74.5**	-82.3	-83.4	**-73.7**	-85.1	-83.9	**-74.3**	-85.6	-73.8	**-64.3**	-75.6	-74.0	**-64.6**	-76.5
**4b**	-80.9	**-71.5**	-81.9	-81.1	**-71.6**	-82.5	-79.3	**-69.9**	-82.2	-79.6	**-70.2**	-82.5	-69.6	**-60.5**	-72.7	-69.8	**-60.7**	-72.8
**4c**	-84.5	**-75.2**	-81.8	-84.7	**-75.2**	-82.5	-85.0	**-75.7**	-85.0	-85.6	**-76.3**	-85.6	-75.6	**-66.6**	-75.7	-75.8	**-66.8**	-76.2
**4d**	-85.5	**-75.8**	-82.8	-85.8	**-76.0**	-83.5	-84.6	**-75.0**	-86.4	-85.1	**-75.5**	-86.8	-74.4	**-65.0**	-76.4	-74.6	**-65.2**	-77.2
**4e**	-81.4	**-71.7**	-79.2	-81.7	**-71.9**	-80.4	-79.7	**-70.1**	-80.7	-80.0	**-70.4**	-81.4	-70.1	**-60.8**	-71.3	-70.3	**-60.9**	-71.9
**4f**	-81.3	**-71.2**	-76.3	-81.8	**-71.6**	-76.6	-84.9	**-74.8**	-79.9	-85.4	**-75.4**	-80.6	-74.7	**-65.5**	-73.6	-75.4	**-66.2**	-74.5
**4g**	-81.4	**-71.3**	-79.7	-82.0	**-72.1**	-80.1	-85.3	**-75.2**	-83.9	-85.9	**-76.1**	-84.9	-71.3	**-62.2**	-75.1	-72.3	**-63.1**	-75.7
**4h**	-80.1	**-70.2**	-78.4	-80.5	**-70.4**	-77.9	-83.6	**-73.6**	-81.5	-83.7	**-73.8**	-81.6	-74.0	**-64.8**	-75.5	-73.9	**-64.7**	-75.6
**4i**	-81.5	**-71.4**	-77.3	-82.3	**-72.0**	-77.3	-85.1	**-75.0**	-80.9	-85.7	**-75.8**	-81.5	-74.8	**-65.7**	-74.6	-75.2	**-66.1**	-75.3
**NFSI**	-46.3	**-36.3**	-43.8	-47.0	**-37.0**	-44.4	-46.8	**-36.9**	-42.7	-47.5	**-37.6**	-43.5	-36.9	**-27.3**	-37.6	-37.1	**-27.5**	-37.5
**Selectfluor®**	-37.6	**-30.9**	-39.1	-37.6	**-30.9**	-39.1	-40.3	**-33.6**	-41.7	-40.3	**-33.6**	-41.7	-27.4	**-20.9**	-29.0	-27.4	**-20.9**	-29.0
**NFPY**	18.8	**22.6**	14.9	18.8	**22.6**	14.9	14.1	**17.9**	10.2	14.1	**17.9**	10.2	18.3	**22.0**	14.4	18.3	**22.0**	14.4

[a]$\Delta E_{sol}=\Delta E_{tot}+\Delta G_{solv}$; [b]$\Delta H_{sol}=\Delta H_{298}+\Delta G_{solv}$; [c]$\Delta G_{sol}=\Delta G_{298}+\Delta G_{solv}$; ΔG_{solv} (single point solvation energy) calculated at the SMD(DMF)/(U)B3LYP/6-31G(d)//(U)B3LYP/6-31G(d,p) level of theory.

Bond Strengths (H—NR$_2$)

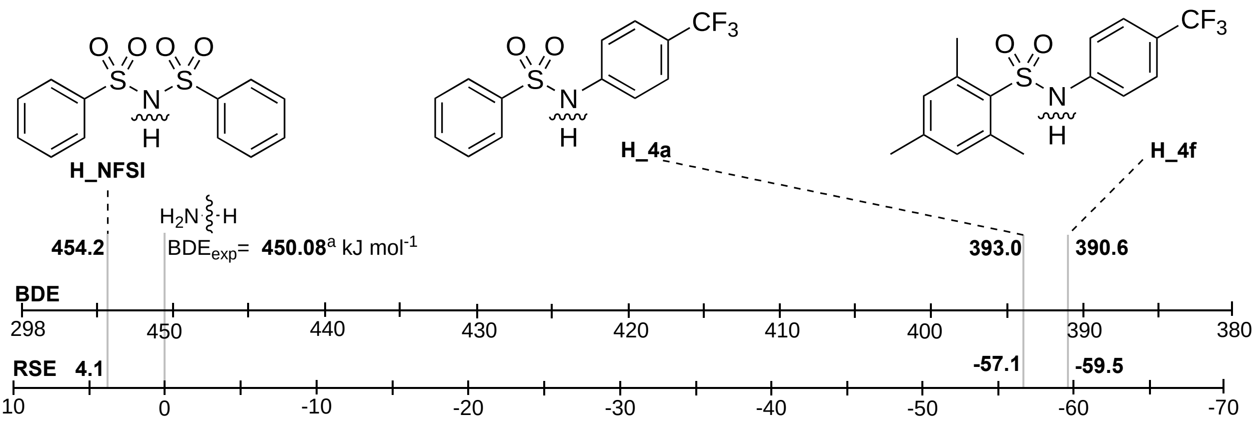

Supplementary Figure 222. Gas phase (ΔH_{298}) **H—NR$_2$** bond dissociation energies (BDEs, in kJ mol^{-1}) and radical stabilization energies (RSEs, in kJ mol^{-1}) relative to the NH_3/•NH_2 reference system calculated at the G3(MP2)-RAD level of theory. a) See Supplementary Table 3.

Supplementary Table 8. Radical stabilization energies (RSEs, in kJ mol^{-1}) relative to the NH_3/•NH_2 reference for the systems listed in Supplementary Figure 222 at different levels of theory.

System	(U)B3LYP/6-31G(d)[gas]			(RO)B2PLYP/G3MP2Large[gas]			G3(MP2)-RAD[gas]		
	ΔE_{tot}	ΔH_{298}	ΔG_{298}	ΔE_{tot}	ΔH_{298}	ΔG_{298}	ΔE_{tot}	ΔH_{298}	ΔG_{298}
**H_4a**									
Boltzmann Avg.	-79.59	-74.64	-82.05	-68.23	-63.30	-70.96	-61.95	**-57.12**	-64.75
Best Conf.	-79.01	-74.03	-81.01	-67.90	-62.92	-69.99	-61.64	-56.77	-63.88
**H_4f**									
Boltzmann Avg.	-83.53	-78.44	-83.02	-72.02	-67.15	-74.10	-64.49	**-59.47**	-66.74
Best Conf.	-82.57	-77.39	-82.07	-71.19	-66.30	-73.23	-63.81	-59.02	-65.94
**H_NFSI**									
Boltzmann Avg.	-14.72	-9.06	-15.17	-5.93	-0.26	-5.84	-1.40	**4.14**	-1.52
Best Conf.	-15.26	-9.60	-15.33	-6.22	-0.56	-6.28	-1.46	4.08	-1.63

QM Data (For Bond Strengths)

Supplementary Table 9. QM properties for gas phase optimized conformers of the system shown in Supplementary Figures 219–222 calculated at the (U)B3LYP\6-31G(d) level of theory. ΔG_{solv} (single point solvation energy) calculated at the SMD(DMF)/(U)B3LYP/6-31G(d)//(U)B3LYP/6-31G(d,p) level of theory.

System (filename)	ΔE_{tot} (hartree)	$HOMO_E$ (hartree)	$LUMO_E$ (hartree)	Low Frequency			corr. ZPE (hartree)	corr. ΔH (hartree)	corr. ΔG (hartree)	ΔG_{solv} (kcal mol^{-1})
**Reference**										
NH_2F (nh2f)	-155.7040027	-0.27212	0.04538	-24	-20	0	0.027606	0.031448	0.005360	-4.82
NH_3 (nh3)	-56.5479476	-0.25240	0.07859	-34	0	0	0.034532	0.038335	0.015453	-
•NH_2 (rnh2)	-55.8726187	-0.30937	0.07700	-20	-12	0	0.018974	0.022754	0.000637	-2.98
**NFSI**										
nfsi_4	-1714.9318638	-0.27444	-0.06430	-8	-1	0	0.208940	0.227945	0.159482	-15.63
nfsi_1	-1714.9318474	-0.27514	-0.06055	-5	-3	0	0.209089	0.227989	0.160868	-14.83
nfsi_10	-1714.9299267	-0.27668	-0.06484	-6	0	0	0.208934	0.227917	0.160124	-16.03
nfsi_11	-1714.9289975	-0.27833	-0.07027	-4	0	0	0.208792	0.227840	0.159516	-17.05
nfsi_3	-1714.9264726	-0.28486	-0.06431	-7	-5	0	0.209111	0.227966	0.161585	-17.37
r_nfsi_1	-1615.1139936	-0.27218	-0.05906	-10	-3	0	0.205007	0.223032	0.156332	-15.55
r_nfsi_10	-1615.1131011	-0.27896	-0.06136	-8	-6	0	0.204981	0.223035	0.155699	-16.93
**Selectfluor®**										
selectfluor	-943.9749746	-0.59847	-0.34799	-18	-11	-7	0.222362	0.233024	0.187349	-208.00
r_selectfluor	-844.1549544	-0.60024	-0.30836	-11	0	0	0.216986	0.226880	0.182071	-208.02
**NFPY**										
nfpy	-347.8003045	-0.49550	-0.26857	-7	0	0	0.093378	0.099474	0.064532	-59.79
r_nfpy	-247.9584997	-0.49386	-0.25979	-18	0	0	0.086625	0.092251	0.058348	-60.00
**3a**										
a3_2	-1053.2751670	-0.26450	-0.04617	-6	0	0	0.203644	0.218662	0.161486	-9.64
a3_5	-1053.2722811	-0.26463	-0.04571	-9	-6	0	0.203796	0.218677	0.162122	-9.51
a3_1	-1053.2712018	-0.26632	-0.04902	-10	0	0	0.203839	0.218726	0.162910	-10.91
a3_3	-1053.2691130	-0.26246	-0.05315	-9	0	0	0.203657	0.218632	0.161924	-10.95
a3_7	-1053.2685358	-0.26722	-0.05129	-9	-1	0	0.203800	0.218660	0.162660	-10.90
a3_4	-1053.2682956	-0.26677	-0.04885	0	0	0	0.204031	0.218779	0.163417	-10.83
a3_6	-1053.2661146	-0.26044	-0.05024	-9	0	0	0.203782	0.218665	0.162415	-10.41
r_a3_1	-953.4543702	-0.27070	-0.04012	0	0	0	0.199287	0.213522	0.156359	-9.92
r_a3_4	-953.4502484	-0.26813	-0.03820	-1	0	0	0.199490	0.213495	0.158317	-9.57
**3b**										
b3_4	-1092.5865879	-0.26360	-0.04515	-13	-5	0	0.231489	0.247765	0.188252	-9.55
b3_1	-1092.5822152	-0.26530	-0.04835	-3	0	0	0.231745	0.247892	0.189284	-10.85
b3_3	-1092.5809877	-0.25993	-0.05000	-14	-8	-4	0.231547	0.247776	0.189081	-10.52
r_b3_3	-992.7657576	-0.26728	-0.03758	0	0	0	0.227233	0.242605	0.184812	-9.61
r_b3_1	-992.7657154	-0.26740	-0.03764	-10	-5	0	0.227141	0.242564	0.184300	-9.57
**3c**										
c3_4	-1331.4351419	-0.26725	-0.07466	-5	0	0	0.241063	0.263119	0.188592	-5.40
c3_3	-1331.4254844	-0.26590	-0.07973	-6	0	0	0.241021	0.262953	0.188673	-7.84
r_c3_4	-1231.6095572	-0.27201	-0.07343	-3	-3	0	0.236176	0.257418	0.183401	-5.76

r_c3_3	-1231.6094375	-0.27204	-0.07384	-5	-3	0	0.236160	0.257401	0.183254	-5.75
**4a**										
a4_1	-1503.4152943	-0.25778	-0.06426	-8	-4	0	0.203749	0.222880	0.153317	-10.97
a4_4	-1503.4152321	-0.25757	-0.06426	-8	-4	0	0.203747	0.222885	0.153135	-10.98
a4_10	-1503.4150936	-0.25727	-0.06552	-8	-4	0	0.204120	0.223074	0.155228	-11.23
a4_5	-1503.4150586	-0.25737	-0.06559	-9	-4	-3	0.204080	0.223052	0.155002	-11.26
r_a4_1	-1403.6120663	-0.24575	-0.03880	-5	0	0	0.199969	0.218048	0.149649	-11.69
**4b**										
b4_2	-1840.4494647	-0.26829	-0.07066	-3	0	0	0.208426	0.231236	0.152584	-9.92
b4_1	-1840.4493760	-0.26814	-0.07056	-2	-1	0	0.208408	0.231238	0.152196	-9.94
b4_5	-1840.4490883	-0.26772	-0.07129	-6	-2	0	0.208634	0.231339	0.153549	-10.27
b4_3	-1840.4489566	-0.26805	-0.07186	-8	-1	0	0.208632	0.231358	0.153224	-10.40
b4_4	-1840.4489097	-0.26787	-0.07169	-7	-3	0	0.208616	0.231334	0.153371	-10.42
r_b4_1	-1740.6447822	-0.25565	-0.06383	-5	-3	0	0.204363	0.226229	0.147372	-10.81
r_b4_2	-1740.6447711	-0.25561	-0.06371	-5	-1	0	0.204366	0.226225	0.147680	-10.82
r_b4_3	-1740.6447868	-0.25578	-0.06438	-3	-1	0	0.204404	0.226248	0.147917	-10.79
**4c**										
c4_1	-1364.8424554	-0.25553	-0.06230	-6	-5	0	0.182494	0.199647	0.136133	-10.24
c4_2	-1364.8421870	-0.25529	-0.06356	-8	-1	0	0.182738	0.199773	0.137276	-10.51
r_c4_1	-1265.0389479	-0.24674	-0.03854	-1	0	0	0.178511	0.194623	0.132584	-11.20
**4d**										
d4_1	-1963.0093728	-0.26207	-0.07207	-4	-1	0	0.194031	0.214390	0.141457	-11.32
d4_3	-1963.0093208	-0.26198	-0.07208	-5	0	0	0.194034	0.214390	0.141512	-11.33
d4_5	-1963.0090918	-0.26160	-0.07358	-3	0	0	0.194441	0.214597	0.143610	-11.71
d4_4	-1963.0090359	-0.26180	-0.07368	-5	0	0	0.194420	0.214586	0.143494	-11.74
r_d4_1	-1863.2067350	-0.25016	-0.04954	-4	0	0	0.190262	0.209569	0.138086	-12.08
**4e**										
e4_1	-1617.9406273	-0.25117	-0.05673	-5	-3	0	0.236625	0.258327	0.183124	-12.10
e4_2	-1617.9405746	-0.25114	-0.05675	-5	-3	0	0.236633	0.258338	0.182984	-12.14
e4_7	-1617.9405421	-0.24988	-0.05800	-4	-1	0	0.236972	0.258507	0.184790	-12.43
e4_6	-1617.9404666	-0.24993	-0.05796	-5	-3	0	0.236935	0.258490	0.184547	-12.47
e4_4	-1617.9404659	-0.24914	-0.05722	0	0	0	0.237051	0.258558	0.184974	-12.48
e4_3	-1617.9404310	-0.24938	-0.05740	-1	0	0	0.237025	0.258541	0.184924	-12.52
r_e4_1	-1518.1365990	-0.23909	-0.03554	-4	0	0	0.232864	0.253503	0.179507	-12.81
r_e4_3	-1518.1366287	-0.23895	-0.03594	0	0	0	0.232902	0.253525	0.179660	-12.83
**4f**										
f4_11	-1621.3623737	-0.25383	-0.05916	-14	-6	-4	0.287572	0.311570	0.231651	-10.56
f4_10	-1621.3623138	-0.25376	-0.05916	-14	-6	-3	0.287577	0.311583	0.231448	-10.61
f4_1	-1621.3597198	-0.25338	-0.06307	-6	-1	0	0.288015	0.311816	0.233734	-10.68
f4_3	-1621.3597097	-0.25360	-0.06316	-6	0	0	0.288037	0.311825	0.233698	-10.71
r_f4_1	-1521.5580621	-0.24301	-0.03753	-9	0	0	0.283822	0.306733	0.228640	-11.18
**4g**										
g4_19	-1857.2342628	-0.25160	-0.05791	-4	-1	0	0.458759	0.490959	0.393269	-11.32
g4_7	-1857.2342025	-0.25153	-0.05799	-5	0	0	0.458759	0.490971	0.393047	-11.38
g4_11	-1857.2338017	-0.25245	-0.05912	-2	0	0	0.459058	0.491153	0.394178	-11.46
g4_9	-1857.2337812	-0.25224	-0.05911	-3	0	0	0.459026	0.491150	0.393956	-11.48
g4_12	-1857.2337353	-0.25240	-0.05913	-2	0	0	0.459064	0.491162	0.394110	-11.48

g4_10	-1857.2337251	-0.25217	-0.05909	-1	0	0	0.459034	0.491155	0.394006	-11.50
g4_1	-1857.2318335	-0.25286	-0.06068	-3	0	0	0.459211	0.491203	0.395097	-11.52
g4_3	-1857.2318267	-0.25321	-0.06056	-5	0	0	0.459236	0.491226	0.395420	-11.50
g4_2	-1857.2316834	-0.25303	-0.06068	-3	0	0	0.459187	0.491176	0.395589	-11.56
g4_5	-1857.2316566	-0.25318	-0.06055	-5	0	0	0.459179	0.491196	0.395398	-11.50
g4_13	-1857.2293743	-0.25485	-0.05754	-6	-3	0	0.458869	0.491015	0.393627	-11.99
g4_14	-1857.2293427	-0.25529	-0.05595	-7	-5	-3	0.458864	0.491000	0.393489	-12.02
r_g4_6	-1757.4296659	-0.24282	-0.03782	-7	-1	0	0.454937	0.486108	0.388770	-12.01
r_g4_7	-1757.4297268	-0.24281	-0.03784	-5	-2	0	0.454973	0.486139	0.389254	-12.00
r_g4_2	-1757.4297253	-0.24281	-0.03804	-6	-2	0	0.454961	0.486129	0.389419	-12.02
r_g4_9	-1757.4295361	-0.24338	-0.03725	-4	-1	0	0.454964	0.486117	0.389873	-12.22
r_g4_11	-1757.4295390	-0.24340	-0.03741	-1	0	0	0.455050	0.486158	0.390238	-12.20
**4h**										
h4_3	-1696.5685820	-0.24503	-0.05487	-12	-5	-3	0.292950	0.317627	0.236919	-11.43
h4_1	-1696.5685264	-0.24500	-0.05486	-12	-5	0	0.292958	0.317641	0.236717	-11.47
h4_4	-1696.5659036	-0.24418	-0.05887	-9	-4	0	0.293292	0.317819	0.238624	-11.50
h4_10	-1696.5659706	-0.24449	-0.05963	-3	0	0	0.293446	0.317907	0.239042	-11.42
h4_8	-1696.5658687	-0.24434	-0.05910	-9	-4	0	0.293270	0.317807	0.238411	-11.55
r_h4_9	-1596.7638243	-0.23343	-0.03550	-7	-6	0	0.289116	0.312773	0.232600	-12.00
r_h4_6	-1596.7638118	-0.23373	-0.03530	-8	-2	0	0.289040	0.312726	0.232909	-12.04
r_h4_1	-1596.7636644	-0.23272	-0.03581	-8	-3	0	0.289116	0.312757	0.233217	-12.02
r_h4_2	-1596.7636970	-0.23268	-0.03572	-4	-2	0	0.289151	0.312771	0.233649	-12.02
**4i**										
i4_3	-1621.3623737	-0.25383	-0.05916	-14	-6	-4	0.287572	0.311570	0.231650	-10.56
i4_1	-1621.3597198	-0.25338	-0.06307	-6	-1	0	0.288015	0.311816	0.233734	-10.68
i4_2	-1621.3597097	-0.25360	-0.06315	-6	0	0	0.288037	0.311825	0.233698	-10.71
r_i4_1	-1521.5580621	-0.24301	-0.03753	-9	0	0	0.283822	0.306733	0.228639	-11.18
r_i4_2	-1521.5580632	-0.24325	-0.03717	-7	0	0	0.283782	0.306723	0.228520	-11.20
**H_NFSI**										
AH_4	-1404.2573031	-0.24684	-0.05211	-5	0	0	0.213739	0.231732	0.165545	-
AH_2	-1404.2572899	-0.24608	-0.05200	-4	0	0	0.213736	0.231733	0.165596	-
AH_1	-1404.2572829	-0.24667	-0.05216	-3	0	0	0.213756	0.231754	0.165207	-
AH_7	-1404.2557348	-0.24096	-0.04953	-4	-4	0	0.213460	0.231585	0.164417	-
AH_8	-1404.2557275	-0.24072	-0.04957	-7	-4	0	0.213447	0.231577	0.164278	-
AH_5	-1404.2542400	-0.24263	-0.05170	-4	0	0	0.213583	0.231698	0.164340	-
**H_4a**										
BH_5	-1522.2019427	-0.24073	-0.04289	-1	0	0	0.297387	0.320341	0.243265	-
BH_6	-1522.2019280	-0.24056	-0.04289	0	0	0	0.297437	0.320371	0.243439	-
BH_2	-1522.2013436	-0.23990	-0.04519	-4	0	0	0.297589	0.320450	0.244233	-
BH_3	-1522.2013232	-0.23998	-0.04533	-2	0	0	0.297629	0.320470	0.244358	-
BH_10	-1522.2003805	-0.23915	-0.04561	-3	0	0	0.297303	0.320346	0.242307	-
BH_9	-1522.2003321	-0.23924	-0.04562	-4	0	0	0.297304	0.320356	0.241920	-
**H_4f**										
CH_1	-1615.7835103	-0.26851	-0.05156	-2	0	0	0.218443	0.236456	0.171172	-
CH_3	-1615.7792212	-0.27227	-0.05459	-5	0	0	0.218576	0.236455	0.170938	-

Supplementary Table 10. Values of individual calculation steps used in G3(MP2)-RAD schemes and (RO)B2PYLP/G3MP2Large performed over gas phase optimized geometries [at (U)B3LYP/6-31G(d) level] for the system listed in Supplementary Table 9.

System	(U)B3LYP/6-31G(d) (in hartree, scaled by 0.9806)			(RO)MP2/6-31G(d)	(RO)CCSD(T)/6-31G(d)	(RO)MP2/GTMP2large	(RO)B2PLYP/GTMP2large
(filename)	corr. ZPE	corr. ΔH	corr. ΔG	//(U)B3LYP/6-31G(d), (in hartree)			
**Reference**							
NH_2F (nh2f)	0.027070	0.030918	0.004823	-155.2928560	-155.3157397	-155.4878897	-155.6771412
NH_3 (nh3)	0.033862	0.037667	0.014782	-56.3541860	-56.3720855	-56.4480463	-56.5326182
$\bullet NH_2$ (rnh2)	0.018606	0.022386	0.000269	-55.6907831	-55.7115606	-55.7663229	-55.8517927
**NFSI**							
nfsi_4	0.204887	0.224171	0.155127	-1711.2044958	-1711.3855435	-1712.4332957	-1714.4447770
nfsi_1	0.205032	0.224214	0.156513	-1711.2040471	-1711.3854447	-1712.4313977	-1714.4432430
nfsi_10	0.204881	0.224144	0.155770	-1711.2020963	-1711.3831137	-1712.4286922	-1714.4408379
nfsi_11	0.204741	0.224069	0.155164	-1711.2009659	-1711.3818912	-1712.4273936	-1714.4400785
nfsi_3	0.205054	0.224192	0.157230	-1711.2017777	-1711.3819260	-1712.4331851	-1714.4410891
r_nfsi_1	0.201030	0.219319	0.152073	-1611.6019199	-1611.7888106	-1612.7167795	-1614.6344531
r_nfsi_10	0.201004	0.219322	0.151440	-1611.6006552	-1611.7871775	-1612.7127394	-1614.6315103
**Selectfluor®**							
selectfluor	0.218049	0.228888	0.182899	-941.8754849	-942.0223762	-942.5255475	-943.6208704
r_selectfluor	0.212776	0.222833	0.177740	-842.2730496	-842.4223106	-842.8069851	-843.8078890
**NFPY**							
nfpy	0.091566	0.097749	0.062674	-346.7725617	-346.8467826	-347.1351839	-347.6442141
r_nfpy	0.084944	0.090645	0.056630	-247.1542204	-247.2310701	-247.3986523	-247.8102347
**3a**							
a3_2	0.199693	0.214926	0.157313	-1050.7605638	-1050.9116666	-1051.6212927	-1052.9272544
a3_5	0.199843	0.214940	0.157949	-1050.7579230	-1050.9091024	-1051.6188118	-1052.9243595
a3_1	0.199884	0.214989	0.158736	-1050.7570411	-1050.9077561	-1051.6197126	-1052.9246339
a3_3	0.199706	0.214897	0.157751	-1050.7547473	-1050.9053304	-1051.6155251	-1052.9207690
a3_7	0.199846	0.214924	0.158487	-1050.7553104	-1050.9056470	-1051.6155468	-1052.9197673
a3_4	0.200073	0.215040	0.159242	-1050.7542667	-1050.9051136	-1051.6169819	-1052.9215259
a3_6	0.199829	0.214929	0.158242	-1050.7522089	-1050.9026940	-1051.6127938	-1052.9174102
r_a3_1	0.195421	0.209853	0.152286	-951.1599628	-951.3129665	-951.9047874	-953.1137502
r_a3_4	0.195620	0.209827	0.154244	-951.1558752	-951.3090289	-951.9007722	-953.1092612
**3b**							
b3_4	0.226998	0.243520	0.183514	-1089.9292437	-1090.0957458	-1090.8415181	-1092.2116587
b3_1	0.227249	0.243643	0.184543	-1089.9253188	-1090.0914774	-1090.8394620	-1092.2085887
r_b3_3	0.222825	0.238427	0.180174	-990.3279622	-990.4965367	-991.1249442	-992.3985413
r_b3_1	0.222735	0.238387	0.179663	-990.3279681	-990.4965474	-991.1249523	-992.3985184
**3c**							
c3_4	0.236387	0.258757	0.183555	-1327.8236922	-1328.0202366	-1329.2952407	-1331.0136421
c3_3	0.236345	0.258593	0.183638	-1327.8181106	-1328.0134683	-1329.2917504	-1331.0066058
r_c3_4	0.231594	0.253135	0.178474	-1228.2194158	-1228.4173655	-1229.5741402	-1231.1948548
r_c3_3	0.231579	0.253119	0.178328	-1228.2192410	-1228.4172179	-1229.5740470	-1231.1947995
**4a**							

a4_1	0.199797	0.219210	0.149061	-1499.8128940	-1499.9948658	-1501.1203827	-1502.9660463
a4_4	0.199795	0.219215	0.148878	-1499.8128106	-1499.9947838	-1501.1202889	-1502.9659769
a4_10	0.200160	0.219399	0.150968	-1499.8140520	-1499.9951643	-1501.1243612	-1502.9677692
a4_5	0.200121	0.219378	0.150743	-1499.8139969	-1499.9951165	-1501.1243073	-1502.9677160
r_a4_1	0.196090	0.214436	0.145485	-1400.2275750	-1400.4127345	-1401.4210765	-1403.1705230
**4b**							
b4_2	0.204383	0.227520	0.148165	-1836.0662801	-1836.2649289	-1837.7406016	-1839.9500207
b4_1	0.204365	0.227523	0.147776	-1836.0662301	-1836.2648757	-1837.7405146	-1839.9499227
b4_5	0.204587	0.227621	0.149128	-1836.0677724	-1836.2653196	-1837.7450899	-1839.9517266
b4_3	0.204585	0.227640	0.148802	-1836.0677183	-1836.2652649	-1837.7450100	-1839.9516078
b4_4	0.204569	0.227616	0.148949	-1836.0675379	-1836.2650998	-1837.7448753	-1839.9515603
r_b4_1	0.200398	0.222577	0.143051	-1736.4788938	-1736.6814343	-1738.0392584	-1740.1528414
r_b4_2	0.200401	0.222573	0.143358	-1736.4788742	-1736.6814128	-1738.0392560	-1740.1528442
r_b4_3	0.200439	0.222595	0.143594	-1736.4788917	-1736.6814437	-1738.0391998	-1740.1527936
**4c**							
c4_1	0.178954	0.196366	0.132328	-1361.5915636	-1361.7582001	-1362.7460617	-1364.4284643
c4_2	0.179193	0.196489	0.133469	-1361.5925515	-1361.7584171	-1362.7499068	-1364.4301307
r_c4_1	0.175048	0.191404	0.128877	-1262.0052826	-1262.1756322	-1263.0465128	-1264.6331406
**4d**							
d4_1	0.190266	0.210919	0.137366	-1958.8436719	-1959.0356433	-1960.2345743	-1962.4549088
d4_3	0.190270	0.210920	0.137420	-1958.8436043	-1959.0355784	-1960.2344856	-1962.4548377
d4_5	0.190669	0.211122	0.139514	-1958.8448128	-1959.0358986	-1960.2385786	-1962.4565706
d4_4	0.190648	0.211111	0.139398	-1958.8447523	-1959.0358446	-1960.2385219	-1962.4565175
r_d4_1	0.186571	0.206158	0.134089	-1859.2591652	-1859.4540378	-1860.5359406	-1862.6599211
**4e**							
e4_1	0.232034	0.254053	0.178179	-1614.0025933	-1614.2056431	-1615.4454166	-1617.4476434
e4_2	0.232042	0.254064	0.178039	-1614.0025170	-1614.2055709	-1615.4453191	-1617.4475766
e4_7	0.232374	0.254230	0.179842	-1614.0040271	-1614.2062057	-1615.4495701	-1617.4493954
e4_6	0.232339	0.254213	0.179599	-1614.0038964	-1614.2060886	-1615.4494793	-1617.4493368
e4_4	0.232452	0.254279	0.180024	-1614.0037313	-1614.2059585	-1615.4493954	-1617.4493501
e4_3	0.232426	0.254262	0.179975	-1614.0037408	-1614.2059639	-1615.4494057	-1617.4493234
r_e4_1	0.228347	0.249288	0.174656	-1514.4165916	-1514.6229270	-1515.7449392	-1517.6509186
r_e4_3	0.228384	0.249309	0.174809	-1514.4166112	-1514.6229386	-1515.7448993	-1517.6509019
**4f**							
f4_11	0.281993	0.306342	0.225671	-1617.3211635	-1617.5490185	-1618.7840982	-1620.8287562
f4_10	0.281998	0.306355	0.225468	-1617.3210826	-1617.5489439	-1618.7839985	-1620.8286894
f4_1	0.282428	0.306583	0.227751	-1617.3201304	-1617.5472124	-1618.7860268	-1620.8280881
f4_3	0.282449	0.306592	0.227715	-1617.3201645	-1617.5472319	-1618.7860860	-1620.8281049
r_f4_1	0.278316	0.301564	0.222756	-1517.7346498	-1517.9658223	-1519.0830611	-1521.0318463
**4g**							
g4_19	0.449860	0.482540	0.383808	-1852.3210593	-1852.6415380	-1854.0968321	-1856.5379101
g4_7	0.449859	0.482552	0.383585	-1852.3209828	-1852.6414668	-1854.0967405	-1856.5378505
g4_11	0.450152	0.482729	0.384713	-1852.3210776	-1852.6413244	-1854.0967003	-1856.5371189
g4_9	0.450121	0.482726	0.384491	-1852.3211134	-1852.6413531	-1854.0967214	-1856.5371069
g4_12	0.450158	0.482738	0.384644	-1852.3210086	-1852.6412614	-1854.0966118	-1856.5370522
g4_10	0.450129	0.482732	0.384541	-1852.3210438	-1852.6412856	-1854.0966393	-1856.5370478
g4_1	0.450303	0.482779	0.385631	-1852.3215531	-1852.6409779	-1854.1006235	-1856.5375850
g4_3	0.450327	0.482802	0.385953	-1852.3217041	-1852.6410628	-1854.1009382	-1856.5376498

g4_2	0.450279	0.482752	0.386124	-1852.3207933	-1852.6403254	-1854.1000201	-1856.5375023
g4_5	0.450271	0.482772	0.385932	-1852.3208628	-1852.6403562	-1854.1002462	-1856.5375560
g4_13	0.449967	0.482594	0.384165	-1852.3197150	-1852.6387844	-1854.0981877	-1856.5351034
g4_14	0.449962	0.482580	0.384027	-1852.3192122	-1852.6383061	-1854.0976097	-1856.5348857
r_g4_11	0.446222	0.477796	0.380870	-1752.7347907	-1753.0585214	-1754.3959843	-1756.7407201
r_g4_2	0.446135	0.477768	0.380052	-1752.7345414	-1753.0582573	-1754.3959093	-1756.7410377
r_g4_6	0.446112	0.477748	0.379402	-1752.7345220	-1753.0582364	-1754.3958746	-1756.7409526
r_g4_9	0.446138	0.477757	0.380506	-1752.7348279	-1753.0585566	-1754.3959875	-1756.7407195
**4h**							
h4_3	0.287266	0.312310	0.230822	-1692.3391209	-1692.5730427	-1693.8858185	-1696.0196385
h4_1	0.287274	0.312324	0.230620	-1692.3390383	-1692.5729655	-1693.8857184	-1696.0195731
h4_4	0.287602	0.312498	0.232524	-1692.3380447	-1692.5712266	-1693.8876056	-1696.0188713
h4_10	0.287753	0.312584	0.232940	-1692.3382057	-1692.5713360	-1693.8878026	-1696.0189606
h4_8	0.287580	0.312487	0.232312	-1692.3381070	-1692.5712682	-1693.8877085	-1696.0188995
r_h4_9	0.283508	0.307516	0.226598	-1592.7522658	-1592.9895162	-1594.1842240	-1596.2221109
r_h4_6	0.283433	0.307470	0.226908	-1592.7522989	-1592.9895730	-1594.1844062	-1596.2222203
r_h4_1	0.283507	0.307500	0.227216	-1592.7520879	-1592.9893013	-1594.1838315	-1596.2217967
r_h4_2	0.283542	0.307514	0.227648	-1592.7520986	-1592.9893067	-1594.1838223	-1596.2218030
**4i**							
i4_3	0.281993	0.306342	0.225672	-1617.3211636	-1617.5490187	-1618.7840982	-1620.8287562
i4_1	0.282428	0.306583	0.227750	-1617.3201305	-1617.5472125	-1618.7860269	-1620.8280881
i4_2	0.282449	0.306592	0.227715	-1617.3201647	-1617.5472321	-1618.7860863	-1620.8281051
r_i4_1	0.278316	0.301564	0.222754	-1517.7346498	-1517.9658223	-1519.0830610	-1521.0318444
r_i4_2	0.278276	0.301554	0.222635	-1517.7346468	-1517.9658290	-1519.0830917	-1521.0318638
**H_4a**							
AH_4	0.209592	0.227861	0.161118	-1400.8721343	-1401.0496437	-1402.0840927	-1403.8254871
AH_2	0.209590	0.227862	0.161168	-1400.8719922	-1401.0495180	-1402.0839944	-1403.8254587
AH_1	0.209609	0.227882	0.160779	-1400.8720634	-1401.0495806	-1402.0840139	-1403.8254344
AH_7	0.209319	0.227718	0.159992	-1400.8698520	-1401.0476682	-1402.0804518	-1403.8226966
AH_8	0.209306	0.227711	0.159853	-1400.8697871	-1401.0476077	-1402.0804570	-1403.8227290
AH_5	0.209439	0.227827	0.159913	-1400.8677382	-1401.0460541	-1402.0764256	-1403.8195473
**H_4f**							
BH_5	0.297387	0.320341	0.243265	-1518.3776630	-1518.6012327	-1519.7433228	-1521.6842599
BH_6	0.291667	0.314944	0.237290	-1518.3776060	-1518.6011776	-1519.7432890	-1521.6842434
BH_2	0.291816	0.315022	0.238082	-1518.3771393	-1518.6004691	-1519.7454451	-1521.6855570
BH_3	0.291855	0.315042	0.238208	-1518.3770890	-1518.6004297	-1519.7453968	-1521.6855215
BH_10	0.291535	0.314919	0.236159	-1518.3748028	-1518.5990088	-1519.7390399	-1521.6813163
BH_9	0.291536	0.314929	0.235772	-1518.3747861	-1518.5989958	-1519.7390148	-1521.6812824
**H_NFSI**							
CH_1	0.214205	0.232490	0.166652	-1612.2731784	-1612.4510669	-1613.4040713	-1615.3129100
CH_3	0.214336	0.232486	0.166419	-1612.2696472	-1612.4473036	-1613.3990325	-1615.3071863

Free Energy (ΔG) & Enthalpy (ΔH) Surfaces

Supplementary Table 11. Gas phase energies (in kJ mol^{-1}) for reactant complex (RC), transition state (TS) and product complex (PC) for the fluorine atom transfer process between ***iso*-Pr•** and **NFSI, NFSA's** (**4a** and **4f**) calculated at different levels of theory.

	(U)B3LYP/6-31G(d)[gas]						(RO)B2PLYP/G3MP2Large[gas]					
System	ΔG_{298}			ΔH_{298}			ΔG_{298}			ΔH_{298}		
Filename	RC	TS	PC	RC	TS	PC	RC	TS	PC	RC	TS	PC
**NFSI + iso-Pr• (Ref.)**												
nfsi_ipr_ts_15	33.6	60.8	-166.0	-3.9	**10.0**	-211.5	38.8	68.6	-166.7	1.3	**17.8**	-212.2
nfsi_ipr_ts_1	32.5	64.4	-169.5	**-5.0**	13.1	-214.1	39.2	72.2	-169.9	1.7	20.9	-214.5
nfsi_ipr_ts_32	38.8	**57.1**	-182.0	0.1	13.4	-223.6	45.3	66.5	-179.8	6.7	22.9	-221.4
nfsi_ipr_ts_23	35.8	61.7	-182.0	0.0	15.1	-223.6	41.6	70.2	-179.8	5.8	23.6	-221.4
nfsi_ipr_ts_30	33.7	61.5	-172.9	-4.9	15.6	-221.8	34.9	**66.1**	-173.1	-3.7	20.2	-221.9
nfsi_ipr_ts_20	31.4	62.3	-179.7	-4.5	15.9	**-223.7**	32.4	66.2	-182.8	-3.5	19.9	**-226.8**
nfsi_ipr_ts_34	36.0	62.0	**-183.2**	-0.2	16.0	-223.6	43.2	71.8	**-182.9**	7.1	25.8	-223.3
nfsi_ipr_ts_16	33.7	64.5	-173.0	-4.9	16.3	-221.8	34.9	68.3	-173.1	**-3.7**	20.1	-221.9
nfsi_ipr_ts_25	**26.5**	66.3	-174.0	-1.1	17.5	-221.2	**27.8**	69.3	-174.9	0.2	20.5	-222.1
nfsi_ipr_ts_40	47.6	77.8	-182.0	11.4	30.0	-223.6	45.1	76.8	-179.8	8.9	29.0	-221.4
nfsi_ipr_ts_47	48.6	81.8	-173.0	9.2	31.8	-221.8	46.1	81.1	-173.1	6.7	31.1	-221.9
Separate Product			**-202.5**			**-207.4**			**-211.5**			**-216.4**
**4a + *iso*-Pr• (Ref.)**												
a4_ipr_ts_16	31.6	**60.0**	-218.5	**-4.1**	**15.5**	-259.5	32.7	**64.6**	-222.7	1.0	**24.2**	-259.7
a4_ipr_ts_13	**27.6**	60.7	-219.4	-2.7	15.6	-259.3	31.5	65.7	-222.9	5.2	24.6	-258.7
a4_ipr_ts_15	31.4	62.5	-221.8	-3.6	16.7	-260.7	**31.1**	66.2	-225.0	0.2	24.4	-259.9
a4_ipr_ts_2	37.8	67.7	-219.4	-3.2	21.3	-259.3	33.9	68.3	-222.9	**-3.0**	26.0	-258.7
a4_ipr_ts_10	47.2	69.0	-221.5	9.7	21.8	**-265.1**	45.6	72.1	-224.6	12.1	29.0	-264.2
a4_ipr_ts_9	47.2	67.7	**-223.1**	9.4	21.9	-263.6	46.7	72.5	**-227.7**	13.0	30.7	-264.1
a4_ipr_ts_12	47.0	68.4	-221.0	9.7	21.9	-265.1	45.4	71.7	-224.2	12.1	29.2	**-264.2**
a4_ipr_ts_7	34.5	73.8	-210.7	-1.1	24.7	-256.6	31.9	73.2	-208.8	0.3	28.2	-250.7
a4_ipr_ts_1	35.4	72.6	-219.4	-2.6	24.8	-259.4	31.9	72.0	-223.6	-2.2	28.2	-259.6
Separate Product			**-242.0**			**-245.6**			**-251.4**			**-251.0**
**4f + *iso*-Pr• (Ref.)**												
f4_ipr_ts_6	**28.1**	**64.8**	-205.5	**-2.8**	**17.6**	-247.8	31.3	**69.0**	-210.6	**0.5**	**21.8**	-253.0
f4_ipr_ts_8	31.5	68.0	-209.7	-0.6	21.2	-249.9	32.7	71.4	-216.1	0.6	24.6	-256.3
f4_ipr_ts_9	29.2	69.4	-205.4	-0.4	21.4	-248.4	**30.4**	72.8	-210.7	0.8	24.7	-253.6
f4_ipr_ts_4	45.1	68.6	**-219.0**	8.6	23.0	-260.6	42.8	71.6	**-222.6**	6.3	26.1	-264.2
f4_ipr_ts_13	47.0	69.6	-217.4	8.6	23.1	**-261.9**	43.3	71.3	-219.7	5.0	24.7	**-264.2**
f4_ipr_ts_15	39.0	83.5	-217.5	5.0	32.9	-260.6	36.5	82.2	-221.1	2.5	31.6	-264.2
f4_ipr_ts_1	39.0	86.7	-217.5	6.8	37.2	-260.6	34.7	85.1	-221.1	2.6	35.6	-264.2
Separate Product			**-237.4**			**-242.8**			**-246.0**			**-251.4**

Supplementary Table 12. Solvation phase (DMF) energies (in kJ mol^{-1}) for reactant complex (RC), transition state (TS) and product complex (PC) for the fluorine atom transfer process between ***iso*-Pr•** and **NFSI, NFSA's** (**4a** and **4f**) calculated at different levels of theory.

System	(U)B3LYP/6-31G(d)[DMF]						(RO)B2PLYP/G3MP2Large[DMF]					
	$\Delta G_{sol}{}^{a}$			$\Delta H_{sol}{}^{b}$			ΔG_{sol}			ΔH_{sol}		
Filename	RC	TS	PC	RC	TS	PC	RC	TS	PC	RC	TS	PC
**NFSI + iso-Pr● (Ref.)**												
nfsi_ipr_ts_15	32.7	52.9	-172.8	-4.8	2.2	-218.3	38.0	60.8	-173.5	0.4	10.0	-219.0
nfsi_ipr_ts_1	32.7	55.7	-175.1	-4.8	4.3	-219.6	39.4	63.5	-175.5	2.0	12.1	-220.1
nfsi_ipr_ts_32	33.2	**46.6**	-189.0	-5.5	3.0	**-230.6**	39.7	56.1	-186.8	1.0	12.4	-228.5
nfsi_ipr_ts_23	32.6	50.2	-189.0	-3.2	3.7	-230.6	38.3	58.7	-186.8	2.5	12.2	-228.5
nfsi_ipr_ts_30	30.1	47.6	-179.1	**-8.5**	1.7	-228.0	31.4	**52.2**	-179.2	-7.2	6.3	-228.1
nfsi_ipr_ts_20	28.9	48.9	-183.2	-7.0	2.6	-227.2	29.9	52.9	-186.4	-6.0	6.5	**-230.4**
nfsi_ipr_ts_34	32.3	49.0	**-189.6**	-3.8	2.9	-229.9	39.6	58.8	**-189.3**	3.5	12.8	-229.6
nfsi_ipr_ts_16	30.1	49.1	-179.1	-8.5	**0.9**	-228.0	31.4	52.9	-179.2	**-7.2**	**4.7**	-228.1
nfsi_ipr_ts_25	**19.6**	52.8	-179.5	-8.0	4.0	-226.7	**20.9**	55.8	-180.3	-6.7	7.0	-227.5
nfsi_ipr_ts_40	33.8	57.7	-189.0	-2.3	9.9	-230.6	31.3	56.6	-186.8	-4.8	8.9	-228.5
nfsi_ipr_ts_47	39.5	60.6	-179.1	0.1	10.5	-228.0	37.0	59.9	-179.2	-2.3	9.8	-228.1
Separate Product			**-214.9**			**-218.1**			**-218.8**			**-223.6**
**4a + *iso*-Pr● (Ref.)**												
a4_ipr_ts_16	29.0	47.0	-227.0	-6.6	2.6	-267.9	30.9	52.4	-230.4	-0.5	12.2	-267.1
a4_ipr_ts_13	**20.9**	**46.3**	-227.6	**-9.3**	**1.2**	-267.4	**25.5**	**52.1**	-230.3	-0.5	11.2	-265.9
a4_ipr_ts_15	29.1	48.8	**-229.4**	-5.8	3.0	-268.2	29.6	53.3	-231.7	-1.1	11.8	-266.3
a4_ipr_ts_2	32.4	51.7	-227.6	-8.5	5.4	-267.4	29.3	53.1	-230.3	**-7.4**	**11.0**	-265.9
a4_ipr_ts_10	39.4	58.0	-227.0	2.0	10.9	-270.5	38.7	61.9	-229.3	5.4	19.1	-268.7
a4_ipr_ts_9	39.6	55.3	-228.6	1.9	9.6	-269.0	40.0	60.9	**-232.3**	6.5	19.3	-268.6
a4_ipr_ts_12	39.1	57.1	-226.6	1.9	10.8	**-270.5**	38.3	61.3	-228.9	5.3	19.1	**-268.7**
a4_ipr_ts_7	27.5	57.3	-222.5	-8.0	8.3	-268.3	25.7	57.6	-219.8	-5.6	12.8	-261.4
a4_ipr_ts_1	30.4	55.7	-227.5	-7.5	8.0	-267.4	27.7	56.0	-230.9	-6.1	12.5	-266.6
Separate Product			**-252.6**			**-256.1**			**-261.2**			**-260.5**
**4f + *iso*-Pr● (Ref.)**												
f4_ipr_ts_6	**21.4**	**51.5**	-220.2	**-9.7**	**4.1**	-262.8	24.7	**55.7**	-225.4	**-6.4**	**8.3**	-267.9
f4_ipr_ts_8	25.7	54.1	-221.7	-6.6	7.1	-262.1	26.9	57.4	-228.1	-5.4	10.5	-268.5
f4_ipr_ts_9	23.4	55.5	-220.4	-6.4	7.2	-263.6	**24.6**	58.8	-225.6	-5.2	10.6	-268.8
f4_ipr_ts_4	39.2	57.3	**-224.9**	2.5	11.5	-266.6	37.0	60.3	**-228.5**	0.3	14.6	**-270.2**
f4_ipr_ts_13	41.0	60.1	-222.8	2.5	13.4	**-267.5**	37.3	61.8	-225.1	-1.2	15.0	-269.9
f4_ipr_ts_15	31.8	67.3	-223.2	-2.4	16.5	-266.5	29.3	66.0	-226.8	-4.8	15.2	-270.1
f4_ipr_ts_1	32.5	69.6	-223.2	0.1	19.9	-266.5	28.2	68.0	-226.8	-4.1	18.3	-270.1
Separate Product			**-247.4**			**-253.0**			**-256.0**			**-261.6**

[a]$\Delta G_{sol}=\Delta G_{298}+\Delta G_{solv}$; [b]$\Delta H_{sol}=\Delta H_{298}+\Delta G_{solv}$; ΔG_{solv} (single point solvation energy) calculated at the SMD(DMF)/(U)B3LYP/6-31G(d)//(U)B3LYP/6-31G(d,p) level of theory.

Supplementary Table 13. Transition state analysis for spin-charge distribution and % of bond breaking and formation calculated at the (U)B3LYP/6-31G(d) level of theory. Results in DMF have been calculated at the SMD(DMF)/(U)B3LYP/6-31G(d)//(U)B3LYP/6-31G(d,p) level of theory. NBO charge/spin is calculated using the NBO6 module.

	Bond Breaking and Formation Analysis of TS									Spin and Charge on Fluorinating Reagents in TS								
										Charge					Spin			
	Bond Distance[a]		% Bond Elongation (Distance)		Wiberg Index (B_i)		% Evolution of Bond Order (%E_v)			Mulliken		NBO			Mulliken		NBO	
Molecule	N-F	C-F	N-F (TS-R)/R	C-F (TS-P)/P	N-F	C-F	N-F 100-(B_i^{TS}/B_i^R)	C-F 100-(B_i^{TS}/B_i^P)		Gas	DMF	Gas	DMF		Gas	DMF	Gas	DMF
**4a**																		
a4_ipr_ts_1	1.61	2.17	13.1%	54.4%	0.65	0.20	27.0%	75.3%		-0.18	-0.23	-0.20	-0.27		0.26	0.33	0.27	0.34
a4_ipr_ts_10	1.58	2.27	10.6%	61.7%	0.70	0.15	21.3%	82.0%		-0.14	-0.16	-0.16	-0.18		0.21	0.22	0.22	0.23
a4_ipr_ts_12	1.58	2.27	10.6%	61.7%	0.70	0.15	21.3%	82.0%		-0.14	-0.19	-0.16	-0.18		0.21	0.22	0.22	0.23
a4_ipr_ts_13	1.60	2.23	12.3%	59.0%	0.67	0.16	25.5%	80.3%		-0.14	-0.19	-0.16	-0.22		0.22	0.27	0.23	0.28
a4_ipr_ts_15	1.60	2.24	12.1%	59.9%	0.67	0.16	25.6%	80.7%		-0.14	-0.16	-0.16	-0.21		0.21	0.26	0.22	0.27
a4_ipr_ts_16	1.60	2.25	11.8%	59.9%	0.67	0.15	25.1%	81.1%		-0.13	-0.19	-0.16	-0.21		0.21	0.26	0.22	0.27
a4_ipr_ts_2	1.61	2.17	12.7%	54.6%	0.66	0.20	26.1%	76.2%		-0.17	-0.25	-0.19	-0.25		0.25	0.31	0.26	0.32
a4_ipr_ts_7	1.61	2.17	13.2%	54.3%	0.65	0.20	27.0%	75.2%		-0.18	-0.25	-0.20	-0.27		0.26	0.33	0.27	0.34
a4_ipr_ts_9	1.58	2.26	10.5%	61.2%	0.71	0.15	21.0%	82.0%		-0.14	-0.16	-0.16	-0.18		0.21	0.23	0.21	0.23
**4f**																		
f4_ipr_ts_1	1.63	2.18	13.8%	55.1%	0.64	0.20	28.5%	75.0%		-0.18	-0.25	-0.20	-0.27		0.21	0.23	0.21	0.23
f4_ipr_ts_13	1.58	2.25	10.9%	60.4%	0.70	0.16	21.8%	81.0%		-0.14	-0.21	-0.17	-0.18		0.21	0.23	0.21	0.23
f4_ipr_ts_15	1.62	2.18	13.5%	55.0%	0.64	0.20	28.0%	75.8%		-0.18	-0.21	-0.20	-0.26		0.21	0.23	0.21	0.23
f4_ipr_ts_4	1.58	2.24	10.8%	59.8%	0.70	0.16	21.6%	81.0%		-0.14	-0.16	-0.17	-0.19		0.21	0.23	0.21	0.23
f4_ipr_ts_6	1.61	2.23	13.0%	58.5%	0.65	0.17	26.7%	79.4%		-0.14	-0.24	-0.17	-0.23		0.21	0.23	0.21	0.23
f4_ipr_ts_8	1.61	2.22	13.0%	58.0%	0.65	0.18	26.9%	78.6%		-0.15	-0.16	-0.17	-0.24		0.21	0.23	0.21	0.23
f4_ipr_ts_9	1.61	2.22	13.0%	58.1%	0.65	0.17	26.9%	78.7%		-0.15	-0.21	-0.17	-0.24		0.21	0.23	0.21	0.23
**NFSI**																		
nfsi_ipr_ts_1	1.57	2.23	10.6%	58.5%	0.72	0.18	21.1%	78.6%		-0.17	-0.23	-0.19	-0.25		0.24	0.30	0.25	0.31
nfsi_ipr_ts_15	1.56	2.24	10.4%	59.2%	0.72	0.17	20.6%	79.5%		-0.16	-0.26	-0.18	-0.24		0.23	0.33	0.24	0.29
nfsi_ipr_ts_16	1.58	2.20	11.3%	56.5%	0.70	0.18	23.0%	77.8%		-0.16	-0.21	-0.18	-0.26		0.23	0.28	0.24	0.32
nfsi_ipr_ts_20	1.58	2.20	11.2%	56.7%	0.70	0.18	22.9%	77.9%		-0.16	-0.19	-0.18	-0.25		0.23	0.25	0.24	0.31
nfsi_ipr_ts_23	1.56	2.25	10.1%	59.9%	0.73	0.16	19.7%	80.1%		-0.15	-0.24	-0.18	-0.22		0.22	0.31	0.23	0.27
nfsi_ipr_ts_25	1.58	2.20	11.5%	56.5%	0.69	0.19	23.4%	77.3%		-0.17	-0.23	-0.19	-0.26		0.24	0.30	0.25	0.32
nfsi_ipr_ts_30	1.58	2.20	11.2%	56.6%	0.70	0.18	22.8%	77.9%		-0.16	-0.23	-0.18	-0.25		0.23	0.30	0.24	0.31
nfsi_ipr_ts_32	1.56	2.25	9.9%	60.3%	0.73	0.16	19.3%	80.6%		-0.15	-0.23	-0.17	-0.21		0.21	0.30	0.22	0.26
nfsi_ipr_ts_34	1.56	2.24	10.1%	59.3%	0.73	0.16	19.7%	80.0%		-0.16	-0.20	-0.18	-0.22		0.22	0.26	0.23	0.28
nfsi_ipr_ts_40	1.58	2.20	11.6%	56.6%	0.69	0.19	24.0%	77.1%		-0.18	-0.20	-0.20	-0.28		0.25	0.27	0.26	0.34
nfsi_ipr_ts_47	1.58	2.22	11.9%	57.7%	0.69	0.19	24.1%	77.1%		-0.17	-0.26	-0.20	-0.29		0.25	0.34	0.26	0.35
Reactant (**R**)															Product (**P**)			
N-F Bond:	Distance	B_i^R		N-F Bond:	Distance	B_i^R		N-F Bond:	Distance	B_i^R					C-F Bond:		Distance	B_i^P
**$4a_{Avg}$**	**1.43**	**0.89**		**$4f_{Avg}$**	**1.43**	**0.89**		**$NFSI_{Avg}$**	**1.42**	**0.91**						**iso-Pr**	**1.40**	**0.82**
a4_1	1.43	0.89		f4_1	1.43	0.89		nfsi_1	1.41	0.91								
a4_10	1.42	0.90		f4_10	1.43	0.89		nfsi_10	1.41	0.91								
a4_4	1.43	0.89		f4_11	1.43	0.89		nfsi_11	1.42	0.90								
a4_5	1.42	0.90		f4_3	1.43	0.89		nfsi_3	1.42	0.90								

Free Energy & Enthalpy Surfaces in Gas Phase at (U)B3LYP/6-31G(d)

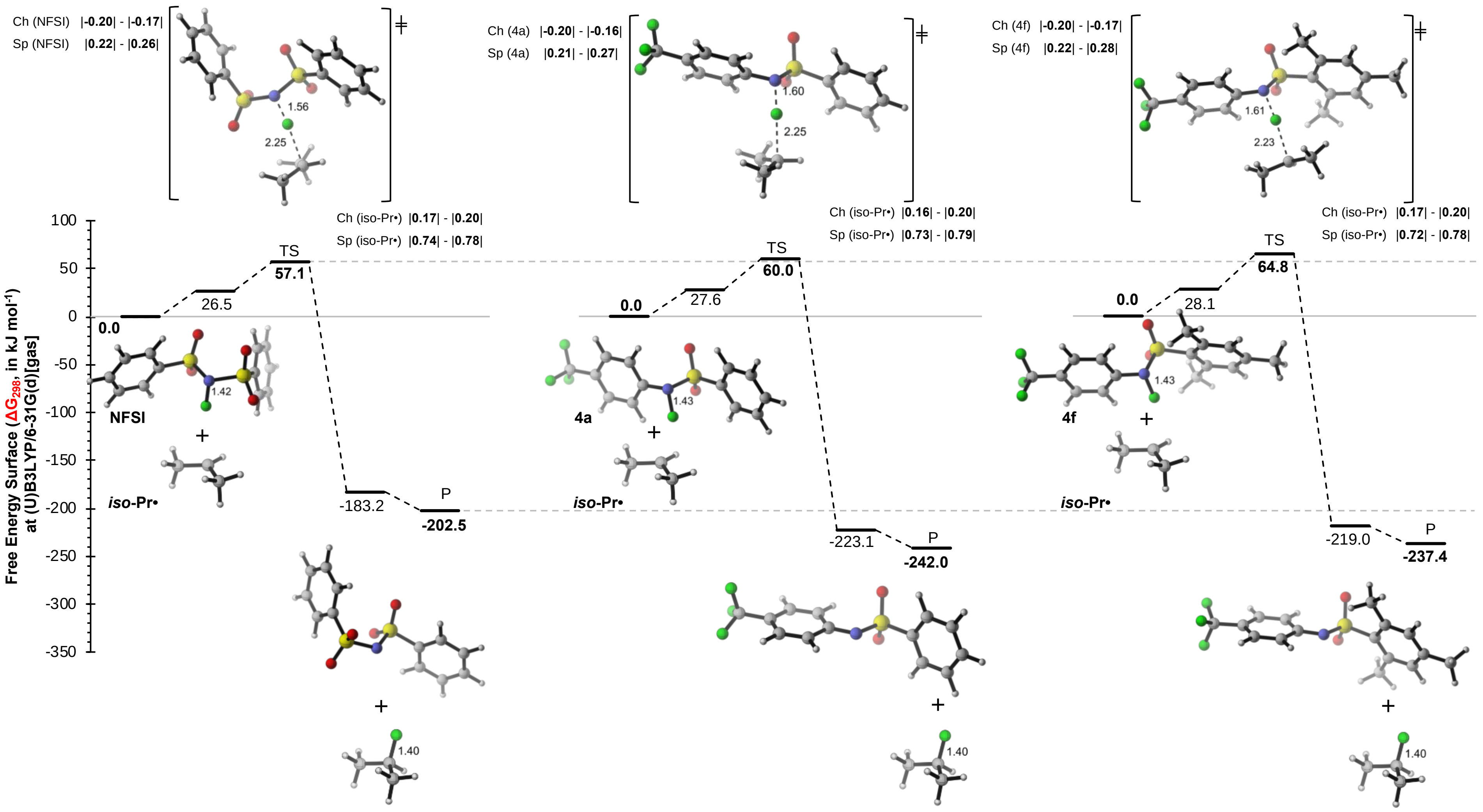

Supplementary Figure 223. Gas phase free energy (**ΔG_{298}**) surfaces (in kJ mol^{-1}) for the fluorine atom transfer process between isopropyl (secondary alkyl) radical (***iso*-Pr•**) and *N*-fluorobenzene-sulfonimide (**NFSI**) and *N*-fluoro-*N*-arylsulfonamides (**NFAS**) **4a** and **4f** calculated at the (U)B3LYP/6-31G(d) level of theory. NBO charge/spin is calculated using the NBO6 module at the (U)B3LYP/6-31G(d) level of theory.

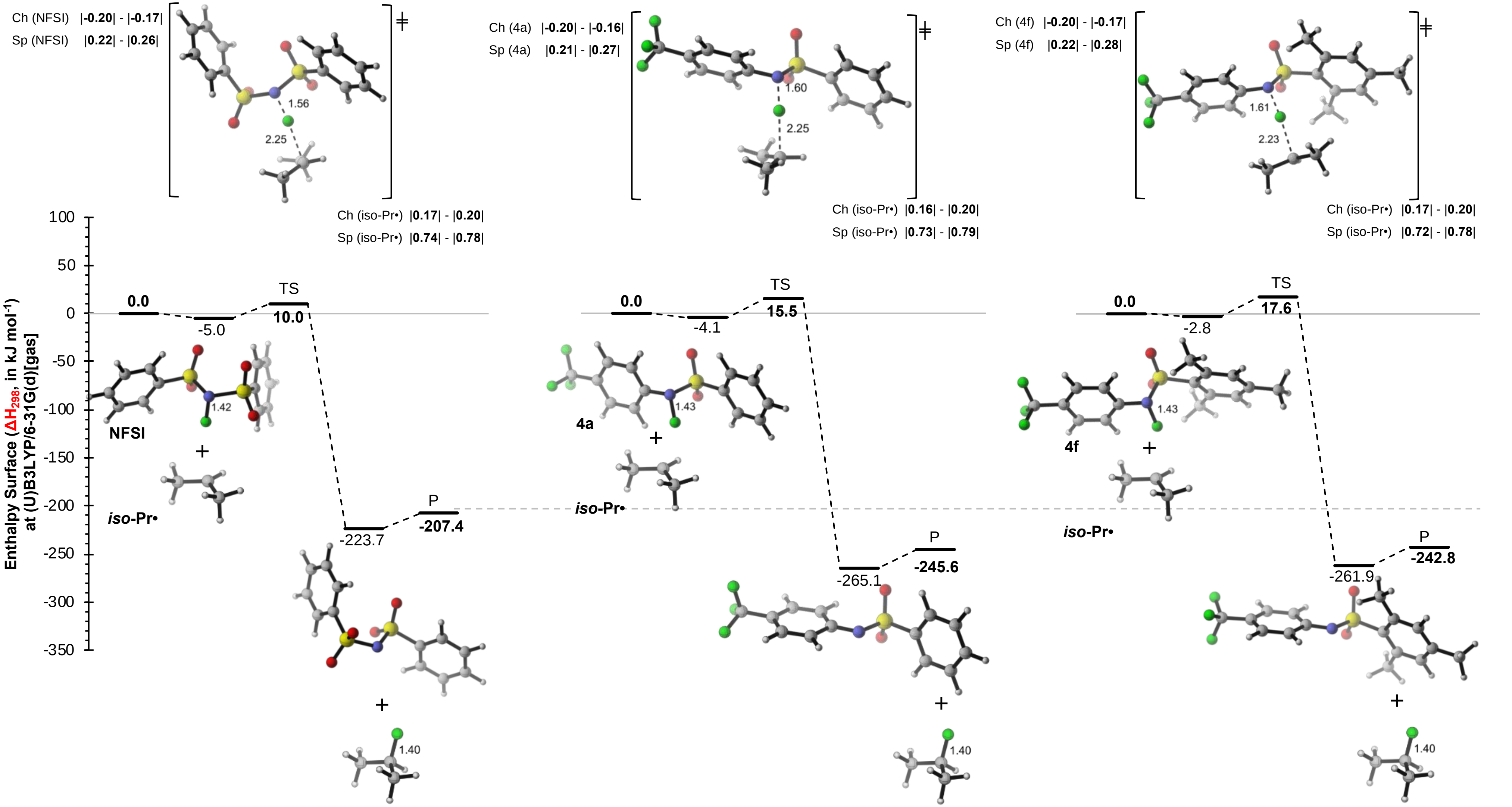

Supplementary Figure 224. Gas phase enthalpy (ΔH_{298}) surfaces (in kJ mol^{-1}) for the fluorine atom transfer process between isopropyl (secondary alkyl) radical (***iso*-Pr•**) and *N*-fluorobenzene-sulfonimide (**NFSI**) and *N*-fluoro-*N*-arylsulfonamides (**NFAS**) **4a** and **4f** calculated at the (U)B3LYP/6-31G(d) level of theory. NBO charge/spin is calculated using the NBO6 module at the (U)B3LYP/6-31G(d) level of theory.

Free Energy Surfaces in DMF Solution (Gas Phase Optimized)

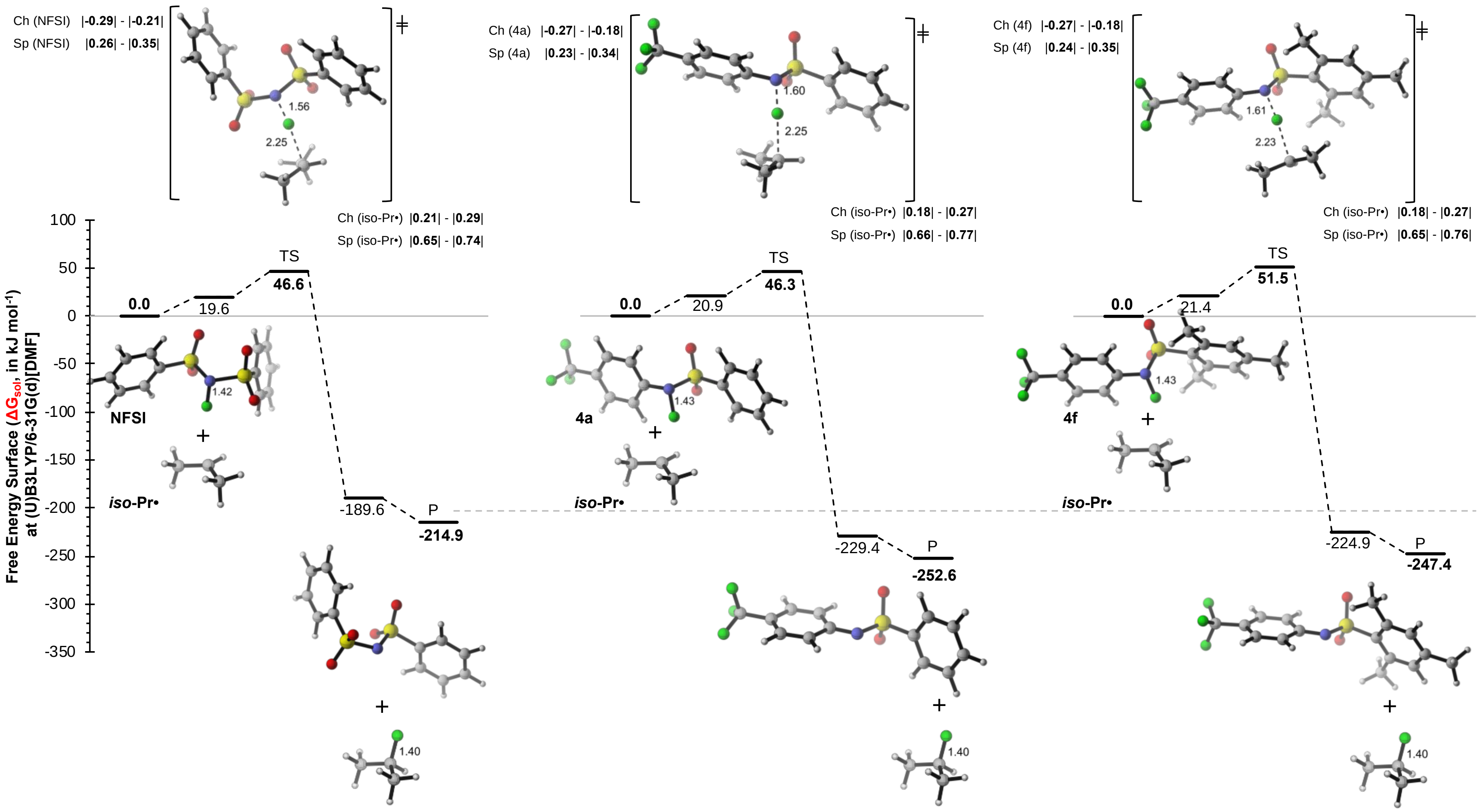

Supplementary Figure 225. Solution phase (DMF) free energy (ΔG_{sol}=ΔG_{298}+ΔG_{solv}) surfaces (in kJ mol^{-1}) for the fluorine atom transfer process between isopropyl (secondary alkyl) radical (***iso*-Pr•**) and *N*-fluorobenzene-sulfonimide (**NFSI**) and *N*-fluoro-*N*-arylsulfonamides (**NFAS**) **4a** and **4f** calculated at the (U)B3LYP/6-31G(d) level of theory. ΔG_{solv} (single point solvation energy) calculated at the SMD(DMF)/(U)B3LYP/6-31G(d)//(U)B3LYP/6-31G(d,p) level of theory. NBO charge/spin is calculated using the NBO6 module at the SMD(DMF)/(U)B3LYP/6-31G(d)//(U)B3LYP/6-31G(d) level of theory.

Charge and Spin Analysis (Gas Phase Optimized)

Lowest Energy TS Structure (ΔG_{298}) At (U)B3LYP/6-31G(d)

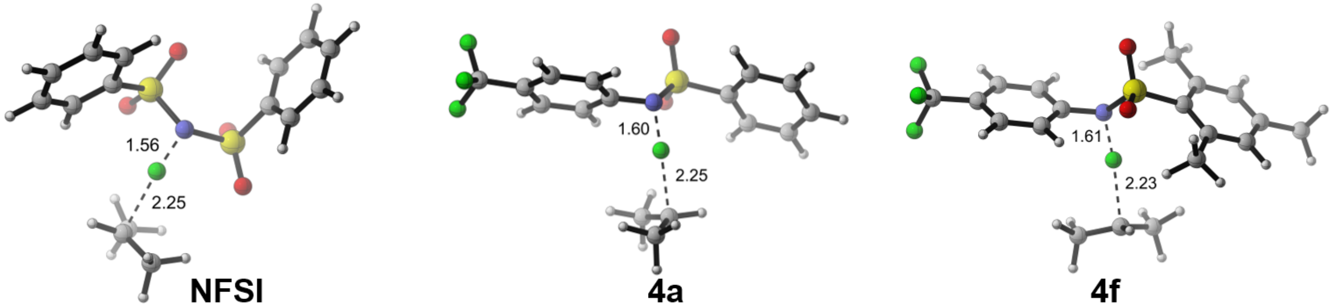

	NFSI	4a	4f
**N-F Bond**			
% Bond Elongation (Distance)[a]	10 - 12%	10 - 13%	11 - 14%
% Evolution of Bond Order ($\%E_v$)[b]	19 - 23%	21 - 27%	22 - 29%
**C-F Bond**			
% Bond Elongation (Distance)[c]	56 - 60%	54 - 62%	55 - 60%
% Evolution of Bond Order ($\%E_v$)[d]	77 - 81%	75 - 82%	75 - 81%
**Charge** on Fluorinating Reagents in TS			
Mulliken-Gas[e]	|-0.18| - |-0.15|	|-0.18| - |-0.13|	|-0.18| - |-0.14|
Mulliken-Solvent(DMF)[f]	|-0.26| - |-0.19|	|-0.25| - |-0.16|	|-0.25| - |-0.16|
NBO6-Gas[g]	|-0.20| - |-0.17|	|-0.20| - |-0.16|	|-0.20| - |-0.17|
NBO6-Solvent(DMF)[h]	|-0.29| - |-0.21|	|-0.27| - |-0.18|	|-0.27| - |-0.18|
**Spin** on Fluorinating Reagents in TS			
Mulliken-Gas	|0.21| - |0.25|	|0.21| - |0.26|	|0.21| - |0.27|
Mulliken-Solvent(DMF)	|0.25| - |0.34|	|0.22| - |0.33|	|0.23| - |0.34|
NBO6-Gas	|0.22| - |0.26|	|0.21| - |0.27|	|0.22| - |0.28|
NBO6-Solvent(DMF)	|0.26| - |0.35|	|0.23| - |0.34|	|0.24| - |0.35|

Supplementary Figure 226. TS analysis for spin-charge distribution and % of bond breaking and formation process for fluorine atom transfer. This analysis is conducted over gas phase optimized geometries at the (U)B3LYP/6-31G(d) level of theory. % Bond elongation is deviation of bond distance in TS to the avg. the bond distance of reactant[a]/product[c]. % Evolution of Bond Order ($\%E_v$) is the ratio of Wiberg Index (B_i) of TS to the avg. B_i of reactant[b]/product[d]. [e]Calcuated at (U)B3LYP/6-31G(d) level. [f]Calculated at SMD(DMF)/(U)B3LYP/6-31G(d)//(U)B3LYP/6-31G(d) level. NBO charge/spin is calculated using NBO-6 module at (U)B3LYP/6-31G(d)[g] and SMD(DMF)/(U)B3LYP/6-31G(d)//(U)B3LYP/6-31G(d)[h] levels. All the properties are provided as a range that is calculated over conformational space, see Supplementary Table 11 for more details.

Free Energy & Enthalpy Surfaces at (RO)B2PLYP/G3MP2Large (Gas Phase Optimized)

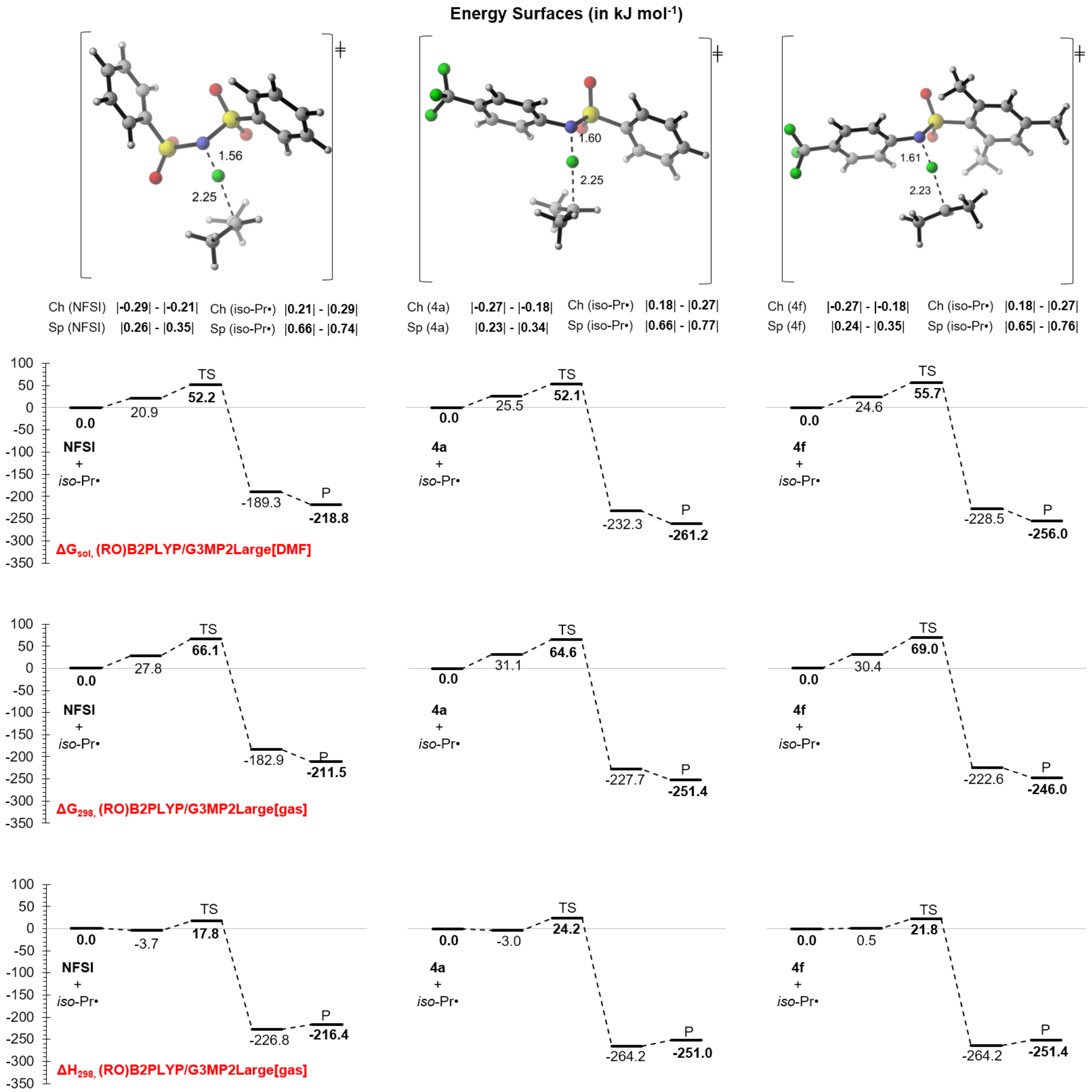

Supplementary Figure 227. Free energy and enthalpy surfaces (in kJ mol^{-1}) for the fluorine atom transfer process between isopropyl (secondary alkyl) radical (***iso*-Pr•**) and *N*-fluorobenzene-sulfonimide (**NFSI**) and *N*-fluoro-*N*-arylsulfonamides (**NFAS**) **4a** and **4f** calculated at the (RO)B2PLYP/G3MP2Large level of theory. $\Delta G_{sol}=\Delta G_{298}+\Delta G_{solv}$, where ΔG_{solv} is (single point solvation energy) calculated at the SMD(DMF)/(U)B3LYP/6-31G(d)//(U)B3LYP/6-31G(d,p) level of theory. NBO charge/spin is calculated using the NBO6 module at the SMD(DMF)/(U)B3LYP/6-31G(d)//(U)B3LYP/6-31G(d) level of theory.

Free Energy & Enthalpy Surfaces in DMF Solution (Solution Phase Optimized)

Supplementary Table 14. Solvation phase optimized (DMF,opt) energies (in kJ mol^{-1}) for reactant complex (RC), transition state (TS) and product complex (PC) for the fluorine atom transfer process between ***iso*-Pr•** and **NFSI, NFSA's** calculated at different levels of theory.

	(U)B3LYP/6-31G(d)[DMF,opt]						(RO)B2PLYP/G3MP2Large[DMF,opt]					
System	$\Delta G_{sol\text{-}opt}$			$\Delta H_{sol\text{-}opt}$			$\Delta G_{sol\text{-}opt}$[a]			$\Delta H_{sol\text{-}opt}$[b]		
Filename	RC	TS	PC	RC	TS	PC	RC	TS	PC	RC	TS	PC
**NFSI + iso-Pr● (Ref.)**												
nfsi_ipr_ts_25	29.4	**44.3**	-184.4	-9.1	1.4	**-228.5**	29.3	**46.1**	-185.1	-8.2	4.2	-228.2
nfsi_ipr_ts_32	26.8	47.1	**-186.2**	-5.4	1.5	-225.2	30.8	53.6	**-190.7**	-0.4	9.0	**-228.7**
nfsi_ipr_ts_16	27.2	47.2	-184.4	-9.1	1.1	-228.5	27.4	48.4	-185.1	-7.9	**3.3**	-228.2
nfsi_ipr_ts_30	29.4	47.2	-184.4	-9.1	1.1	-228.5	29.3	48.4	-185.1	-8.2	3.3	-228.2
nfsi_ipr_ts_20	**26.7**	49.1	-184.7	**-9.1**	2.0	-226.4	**26.3**	50.5	-185.7	**-8.5**	4.3	-226.3
nfsi_ipr_ts_34	28.6	49.3	-183.4	-5.7	1.6	-226.8	33.5	55.8	-182.1	0.2	9.2	-224.6
nfsi_ipr_ts_15	29.8	50.1	-175.1	-6.1	**1.0**	-220.4	32.4	54.9	-178.1	-2.5	6.9	-222.4
nfsi_ipr_ts_23	27.0	50.4	-185.4	-5.2	1.7	-227.0	30.1	57.0	-184.7	-1.1	9.2	-225.3
nfsi_ipr_ts_1	29.6	51.8	-174.4	-5.4	3.1	-221.3	31.5	56.3	-176.4	-2.5	8.6	-222.3
Separate Product			**-216.8**			**-217.8**			**-221.5**			**-225.7**
**4a + *iso*-Pr● (Ref.)**												
a4_ipr_ts_13	26.4	**45.3**	**-226.9**	-7.2	2.6	-263.9	28.4	51.7	**-232.1**	-2.5	11.7	-266.3
a4_ipr_ts_16	30.1	45.5	-225.9	-8.4	3.2	-267.1	32.7	**51.3**	-228.9	-3.0	11.8	**-267.2**
a4_ipr_ts_15	**26.0**	46.6	-224.4	-8.3	**2.5**	-263.4	31.2	52.5	-229.5	-0.4	11.1	-265.8
a4_ipr_ts_10	34.3	49.4	-222.1	-0.6	8.6	-261.3	39.1	54.5	-226.1	7.0	16.5	-262.4
a4_ipr_ts_2	30.2	50.6	-225.0	**-8.7**	5.9	-267.2	28.3	52.0	-226.5	-7.7	**10.1**	-265.9
a4_ipr_ts_12	33.2	52.3	-221.8	1.9	9.4	-261.9	34.6	56.5	-226.2	6.1	16.4	-263.5
a4_ipr_ts_1	30.7	53.4	-212.8	-7.2	7.5	-256.2	27.1	54.4	-221.7	**-8.1**	11.3	-262.2
a4_ipr_ts_9	36.4	54.1	-221.1	-0.6	8.8	-261.0	41.2	59.0	-225.0	7.0	16.5	-262.1
a4_ipr_ts_7	27.9	55.2	-222.9	-6.3	7.9	**-268.3**	**24.2**	56.2	-219.5	-7.2	11.7	-262.2
Separate Product			**-253.1**			**-254.9**			**-260.8**			**-259.7**
**4f + *iso*-Pr● (Ref.)**												
f4_ipr_ts_6	**26.6**	**53.1**	-220.2	**-8.7**	**4.3**	-264.1	**27.4**	**56.7**	-226.8	-7.9	**7.9**	**-270.7**
f4_ipr_ts_9		54.7	-224.3		7.2	-263.6		57.2	-229.7		9.8	-269.1
f4_ipr_ts_8	30.9	55.0	**-225.2**	-7.8	7.3	-263.6	29.8	57.6	**-231.6**	**-8.8**	9.8	-270.0
f4_ipr_ts_4	32.2	57.2	-224.5	-0.6	10.3	**-268.5**	32.6	58.9	-225.8	-0.2	11.9	-269.8
f4_ipr_ts_15	33.8	66.7	-217.7	-2.2	17.0	-259.8	30.4	64.3	-222.3	-5.6	14.6	-264.4
Separate Product			**-251.5**			**-253.1**			**-260.6**			**-262.2**

[a]$\Delta G_{sol\text{-}opt}=\Delta E_{tot}$+corr. $\Delta G+\Delta G_{solv}$; [b]$\Delta H_{sol\text{-}opt}=\Delta E_{tot}$+corr. $\Delta H+\Delta G_{solv}$; corr. ΔG, corr. ΔH, and ΔG_{solv} (single point solvation energy) were calculated at SMD(DMF)/(U)B3LYP/6-31G(d) level. ΔE_{tot} was calculated at (RO)B2PLYP/G3MP2Large//SMD(DMF)/(U)B3LYP/6-31G(d) level

Supplementary Table 15. Transition state analysis for spin-charge distribution and % of bond breaking and formation calculated at the SMD(DMF)/(U)B3LYP/6-31G(d) level of theory. NBO charge/spin is calculated using the NBO6 module.

	Bond Breaking and Formation Analysis of TS								Spin and Charge on Fluorinating Reagents in TS			
	Bond Distance[a]		% Bond Elongation (Distance)		Wiberg Index (B_i)		% Evolution of Bond Order (%E_v)		Charge		Spin	
Molecule	N-F	C-F	N-F (TS-R)/R	C-F (TS-P)/P	N-F	C-F	N-F 100-(B_i^{TS}/B_i^R)	C-F 100-(B_i^{TS}/B_i^P)	Mulliken	NBO	Mulliken	NBO
**4a**												
a4_ipr_ts_1	1.59	2.28	11.2%	60.8%	0.68	0.16	23.4%	80.0%	-0.17	-0.19	0.23	0.24
a4_ipr_ts_10	1.57	2.33	9.9%	64.0%	0.71	0.13	19.9%	83.6%	-0.14	-0.15	0.19	0.20
a4_ipr_ts_12	1.58	2.33	10.1%	64.2%	0.71	0.13	20.2%	83.5%	-0.14	-0.16	0.19	0.20
a4_ipr_ts_13	1.58	2.32	10.6%	63.3%	0.69	0.14	22.2%	82.8%	-0.14	-0.16	0.20	0.21
a4_ipr_ts_15	1.58	2.32	10.7%	63.6%	0.69	0.14	22.3%	83.0%	-0.14	-0.16	0.20	0.20
a4_ipr_ts_16	1.58	2.32	10.7%	63.3%	0.69	0.14	22.4%	82.9%	-0.14	-0.16	0.20	0.21
a4_ipr_ts_2	1.59	2.28	11.1%	60.9%	0.68	0.16	23.1%	80.5%	-0.16	-0.18	0.22	0.23
a4_ipr_ts_7	1.59	2.28	11.2%	60.9%	0.68	0.16	23.5%	80.0%	-0.17	-0.19	0.23	0.24
a4_ipr_ts_9	1.57	2.33	10.0%	63.7%	0.71	0.13	20.0%	83.5%	-0.14	-0.16	0.19	0.20
**4f**												
f4_ipr_ts_15	1.60	2.28	11.8%	60.6%	0.67	0.16	24.7%	79.6%	-0.18	-0.19	0.24	0.24
f4_ipr_ts_4	1.58	2.31	10.3%	63.0%	0.70	0.14	20.6%	82.9%	-0.14	-0.16	0.20	0.20
f4_ipr_ts_6	1.60	2.32	11.4%	63.0%	0.68	0.14	23.5%	82.2%	-0.15	-0.17	0.21	0.21
f4_ipr_ts_8	1.60	2.32	11.7%	63.3%	0.67	0.15	24.2%	81.6%	-0.16	-0.17	0.21	0.22
f4_ipr_ts_9	1.60	2.32	11.7%	63.2%	0.67	0.15	24.2%	81.5%	-0.16	-0.17	0.21	0.22
**NFSI**												
nfsi_ipr_ts_1	1.55	2.34	8.9%	64.5%	0.74	0.14	17.6%	82.4%	-0.16	-0.17	0.20	0.21
nfsi_ipr_ts_15	1.55	2.34	8.7%	64.7%	0.75	0.14	17.3%	82.9%	-0.15	-0.17	0.20	0.20
nfsi_ipr_ts_16	1.56	2.33	9.6%	64.3%	0.73	0.14	19.2%	82.3%	-0.15	-0.17	0.20	0.21
nfsi_ipr_ts_20	1.56	2.33	9.6%	63.7%	0.73	0.14	19.4%	81.9%	-0.16	-0.17	0.20	0.21
nfsi_ipr_ts_23	1.55	2.33	8.8%	64.2%	0.75	0.14	17.2%	82.8%	-0.15	-0.16	0.20	0.20
nfsi_ipr_ts_25	1.56	2.33	9.6%	63.9%	0.73	0.14	19.3%	82.1%	-0.15	-0.17	0.20	0.21
nfsi_ipr_ts_30	1.56	2.33	9.6%	64.3%	0.73	0.14	19.2%	82.3%	-0.15	-0.17	0.20	0.21
nfsi_ipr_ts_32	1.55	2.33	8.8%	64.1%	0.75	0.14	17.2%	82.7%	-0.15	-0.17	0.20	0.20
nfsi_ipr_ts_34	1.55	2.33	8.9%	64.2%	0.75	0.14	17.3%	82.7%	-0.15	-0.17	0.20	0.20

Reactant (**R**)

N-F Bond:	Distance	B_i^R	N-F Bond:	Distance	B_i^R	N-F Bond:	Distance	B_i^R
**$4a_{Avg}$**	**1.43**	**0.89**	**$4f_{Avg}$**	**1.43**	**0.88**	**$NFSI_{Avg}$**	**1.42**	**0.90**
a4_1	1.43	0.88	f4_1	1.43	0.88	nfsi_1	1.42	0.91
a4_10	1.43	0.89	f4_10	1.43	0.88	nfsi_10	1.42	0.91
a4_4	1.43	0.88	f4_11	1.44	0.88	nfsi_11	1.43	0.90
a4_5	1.43	0.89	f4_3	1.43	0.89	nfsi_3	1.42	0.90
						nfsi_4	1.42	0.90

Product (**P**)

C-F Bond:	Distance	B_i^P
**iso-Pr**	**1.42**	**0.80**

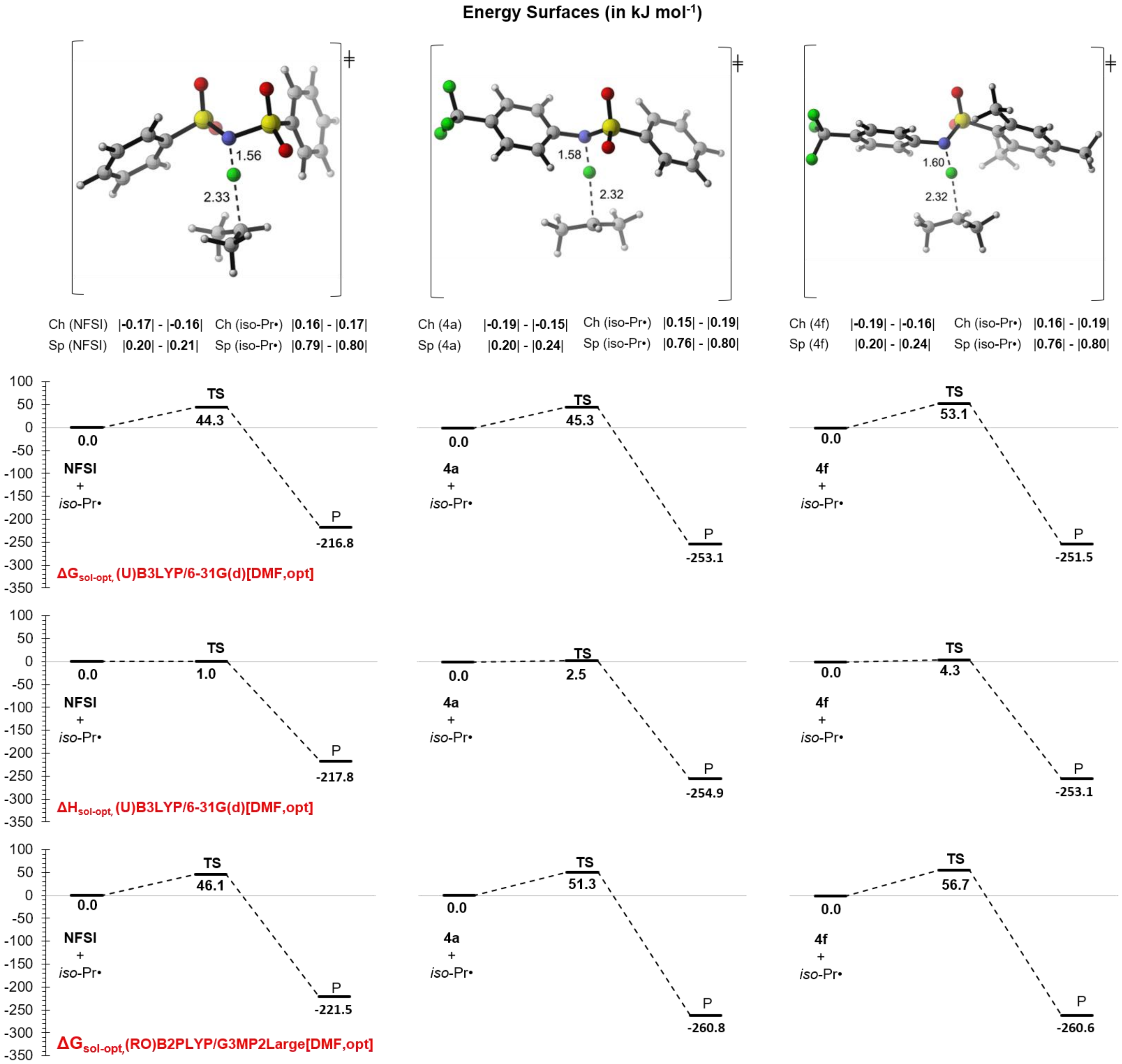

Supplementary Figure 228. Solvation phase optimized (DMF,opt) free energy (**$\Delta G_{sol\text{-}opt}$**) and enthalpy (**$\Delta H_{sol\text{-}opt}$**) surfaces (in kJ mol^{-1}) for the fluorine atom transfer process between isopropyl (secondary alkyl) radical (***iso*-Pr•**) and *N*-fluorobenzene-sulfonimide (**NFSI**) and *N*-fluoro-*N*-arylsulfonamides (**NFAS**) **4a** and **4f** calculated at the SMD(DMF)/(U)B3LYP/6-31G(d) level of theory. Free energy surfaces are re-evaluated at the (RO)B2PLYP/G3MP2Large level of theory. **$\Delta G_{sol\text{-}opt}$** at (RO)B2PLYP/G3MP2Large(DMF,opt] is obtained by adding ΔG_{solv} at SMD(DMF)/(U)B3LYP/6-31G(d) level to ΔG_{298} at (RO)B2PLYP/G3MP2Large //SMD(DMF)/(U)B3LYP/6-31G(d) level. NBO charge/spin is calculated using the NBO6 module at the SMD(DMF)/(U)B3LYP/6-31G(d) level of theory.

Charge and Spin Analysis (Solution Phase Optimized)

Lowest Energy ($\Delta G_{sol\text{-}opt}$) TS Structure At (U)B3LYP/6-31G(d)/SMD(DMF)

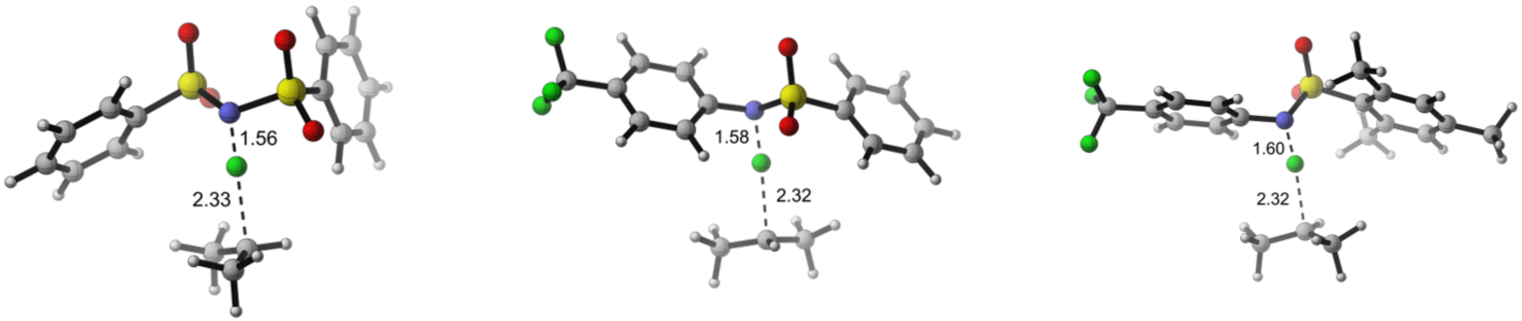

	NFSI	4a	4f
**N-F Bond**			
% Bond Elongation (Distance)[a]	9 - 10%	10 - 11%	10 - 12%
% Evolution of Bond Order (%E_v)[b]	17 - 19%	20 - 23%	21 - 25%
**C-F Bond**			
% Bond Elongation (Distance)[c]	64 - 65%	61 - 64%	61 - 63%
% Evolution of Bond Order (%E_v)[d]	82 - 83%	80 - 84%	80 - 83%
**Charge** on Fluorinating Reagents in TS			
Mulliken-Solvent(DMF)	|-0.16| - |-0.15|	|-0.17| - |-0.14|	|-0.18| - |-0.14|
NBO6-Solvent(DMF)	|-0.17| - |-0.16|	|-0.19| - |-0.15|	|-0.19| - |-0.16|
**Spin** on Fluorinating Reagents in TS			
Mulliken-Solvent(DMF)	|0.19| - |0.20|	|0.19| - |0.23|	|0.20| - |0.24|
NBO6-Solvent(DMF)	|0.20| - |0.21|	|0.20| - |0.24|	|0.20| - |0.24|

Supplementary Figure 229. TS analysis for spin-charge distribution and % of bond breaking and formation process for fluorine atom transfer. This analysis is conducted over implicit solvation optimized geometries at the SMD(DMF)/(U)B3LYP/6-31G(d) level of theory. % Bond elongation is deviation of bond distance in TS to the avg. the bond distance of reactant[a]/product[c]. % Evolution of Bond Order (%E_v) is the ratio of Wiberg Index (B_i) of TS to the avg. B_i of reactant[b]/product[d]. All the properties are provided as a range that is calculated over conformational space, see Supplementary Table 15 for more details.

(U)B3LYP/6-31G(d) - Energy Surfaces with Conformational Details (Gas)

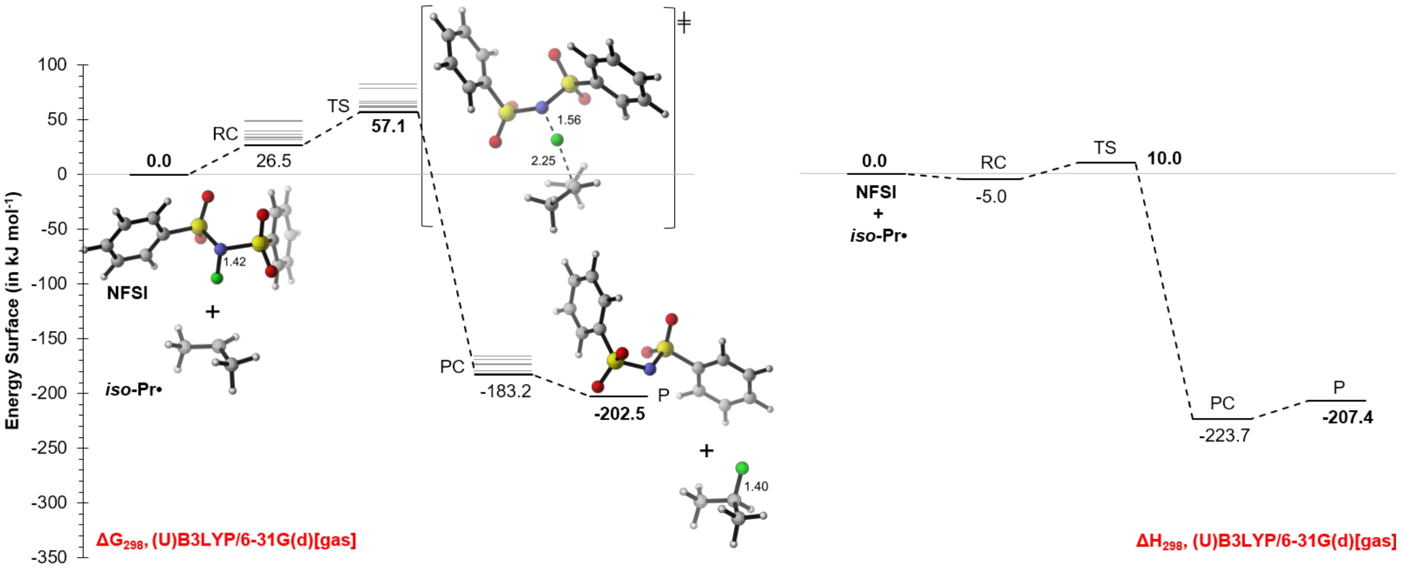

Supplementary Figure 230. Gas phase free energy (ΔG_{298}) and enthalpy (ΔH_{298}) surfaces (in kJ mol^{-1}) for the fluorine atom transfer process between **NFSI** and ***iso*-Pr•** calculated at (U)B3LYP/6-31G(d) level. Faded bars are used to show the conformational space screened for each point along PES.

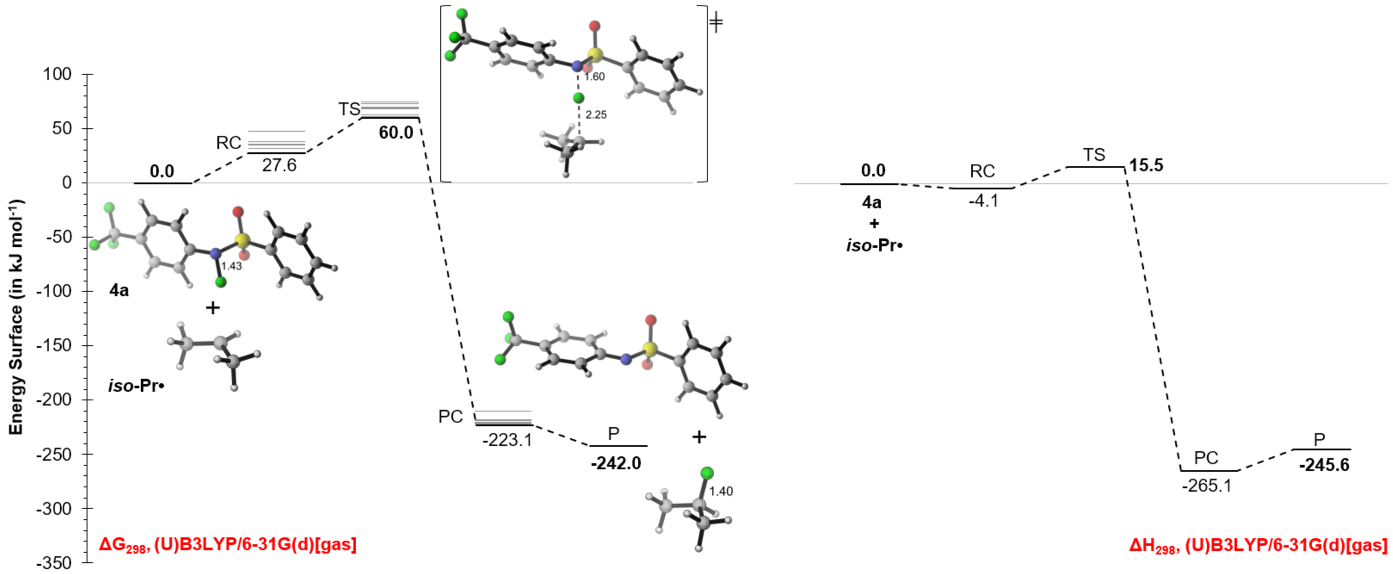

Supplementary Figure 231. Gas phase free energy (ΔG_{298}) and enthalpy (ΔH_{298}) surfaces (in kJ mol^{-1}) for fluorine atom transfer process between **4a** and ***iso*-Pr•** calculated at (U)B3LYP/6-31G(d) level.

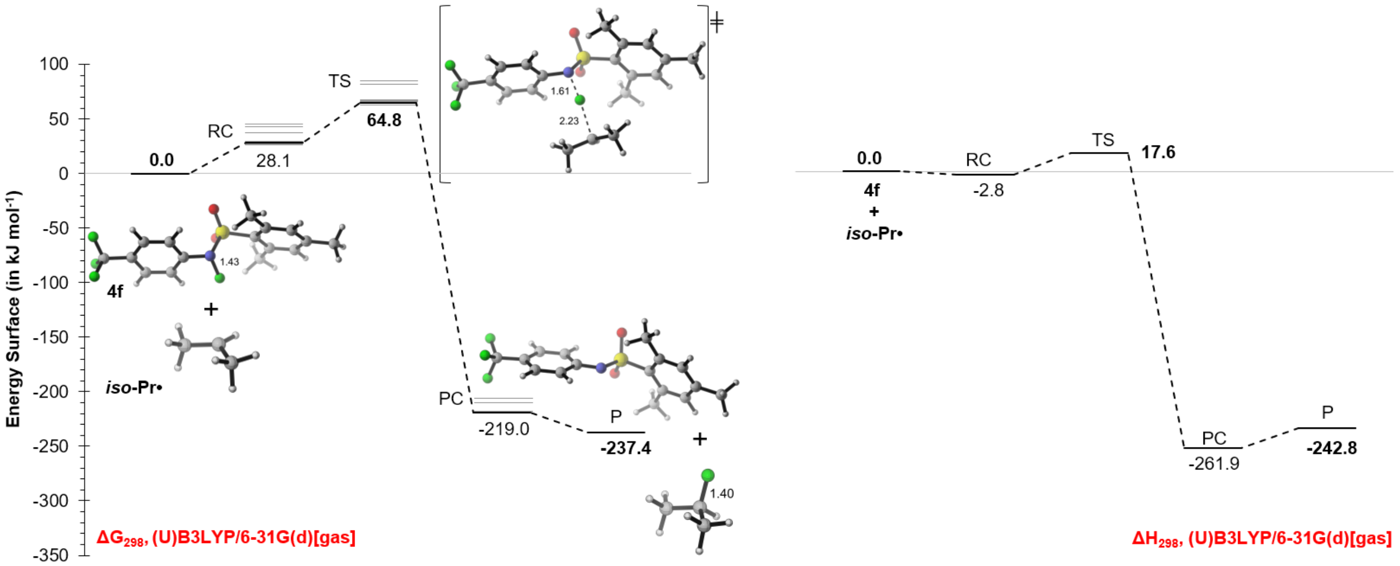

Supplementary Figure 232. Gas phase free energy (ΔG_{298}) and enthalpy (ΔH_{298}) surfaces (in kJ mol^{-1}) for fluorine atom transfer process between **4f** with ***iso*-Pr•** calculated at (U)B3LYP/6-31G(d) level.

(U)B3LYP/6-31G(d) - Energy Surfaces with Conformational Details (DMF)

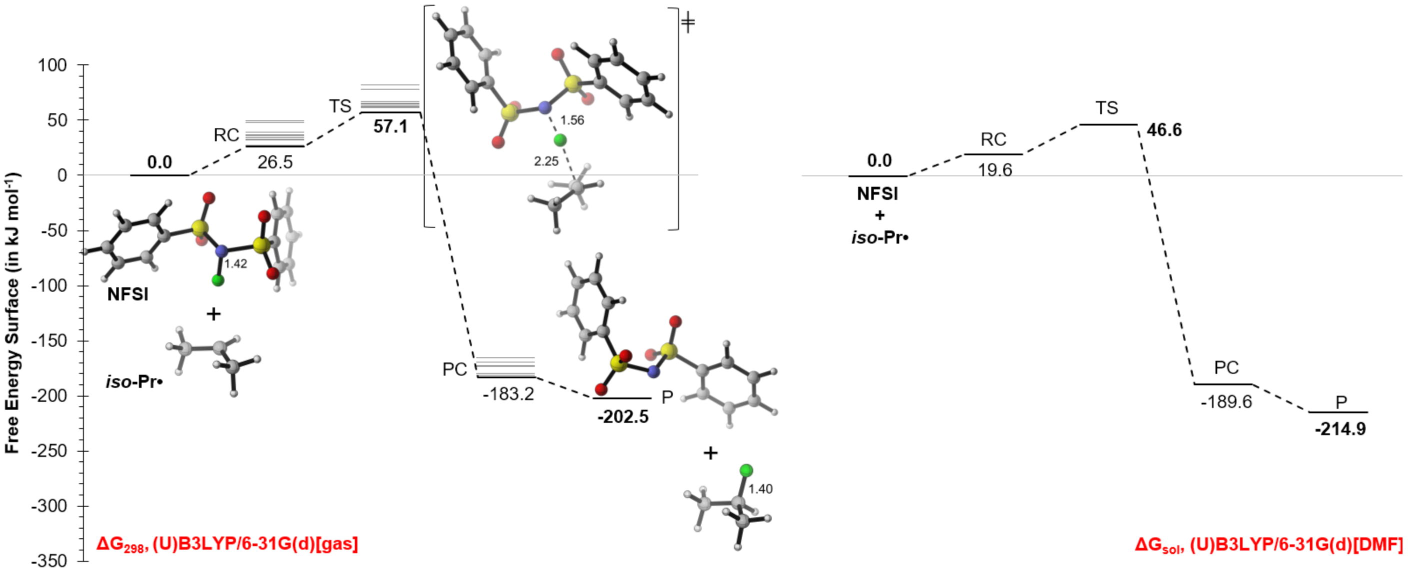

Supplementary Figure 233. Comparison of gas phase (**ΔG_{298}**) and solvation phase (**ΔG_{sol}**=ΔG_{298}+ΔG_{solv}) free energy surfaces (in kJ mol^{-1}) for the fluorine atom transfer process between **NFSI** and ***iso*-Pr•** calculated at (U)B3LYP/6-31G(d) level. ΔG_{solv} (single point solvation energy) calculated at SMD(DMF)/(U)B3LYP/6-31G(d)//(U)B3LYP/6-31G(d,p) level.

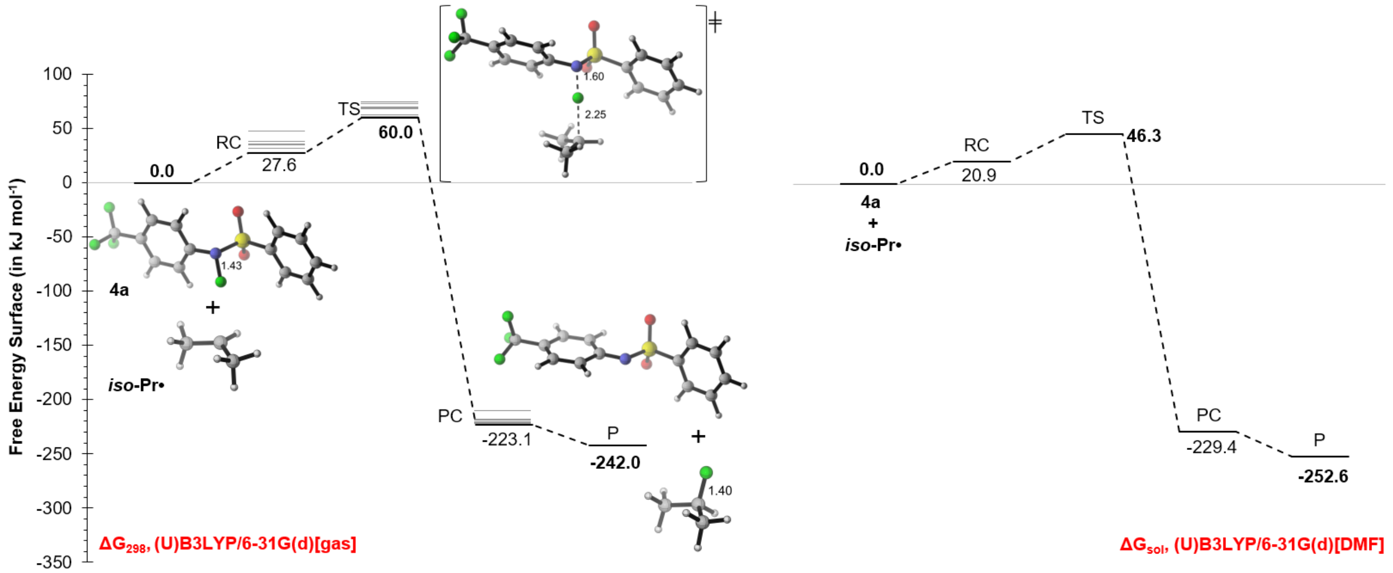

Supplementary Figure 234. Comparison of gas phase (**ΔG_{298}**) and solvation phase (**ΔG_{sol}**=ΔG_{298}+ΔG_{solv}) free energy surfaces (in kJ mol^{-1}) for the fluorine atom transfer process between **4a** and ***iso*-Pr•** calculated at (U)B3LYP/6-31G(d) level.

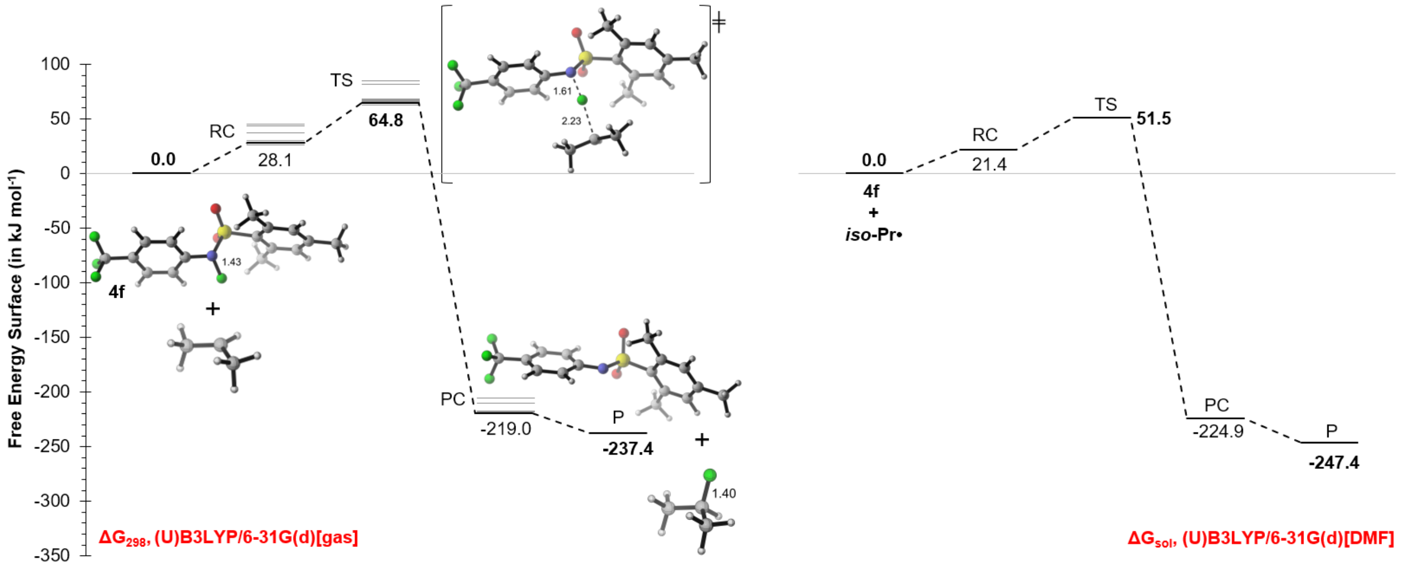

Supplementary Figure 235. Comparison of gas phase (**ΔG_{298}**) and solvation phase (**ΔG_{sol}**=ΔG_{298}+ΔG_{solv}) free energy surfaces (in kJ mol^{-1}) for the fluorine atom transfer process between **4f** with ***iso*-Pr•** calculated at (U)B3LYP/6-31G(d) level.

(U)B3LYP/6-31G(d) - Energy Surfaces with Conformational Details (DMF,opt)

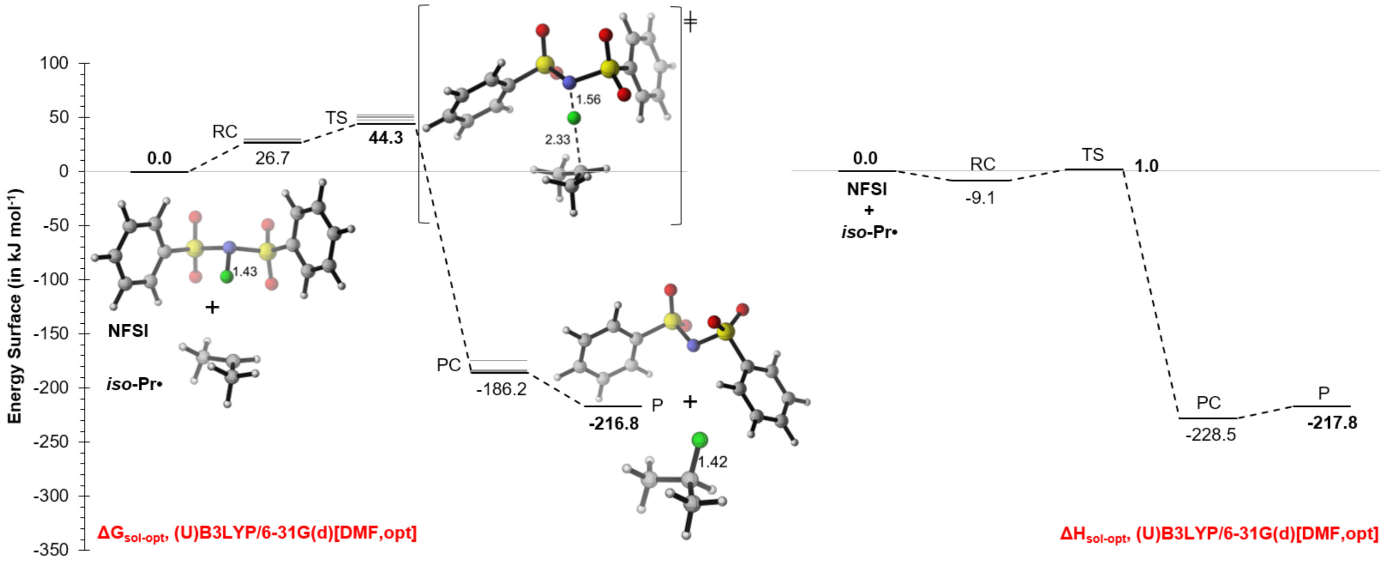

Supplementary Figure 236. Solvation phase optimized (DMF,opt) free energy ($\Delta G_{sol\text{-}opt}$) and enthalpy ($\Delta H_{sol\text{-}opt}$) surfaces (in kJ mol^{-1}) for the fluorine atom transfer process between **NFSI** and ***iso*-Pr•** calculated at (U)B3LYP/6-31G(d) level. Faded bars are used to show the conformational space screened for each point along PES.

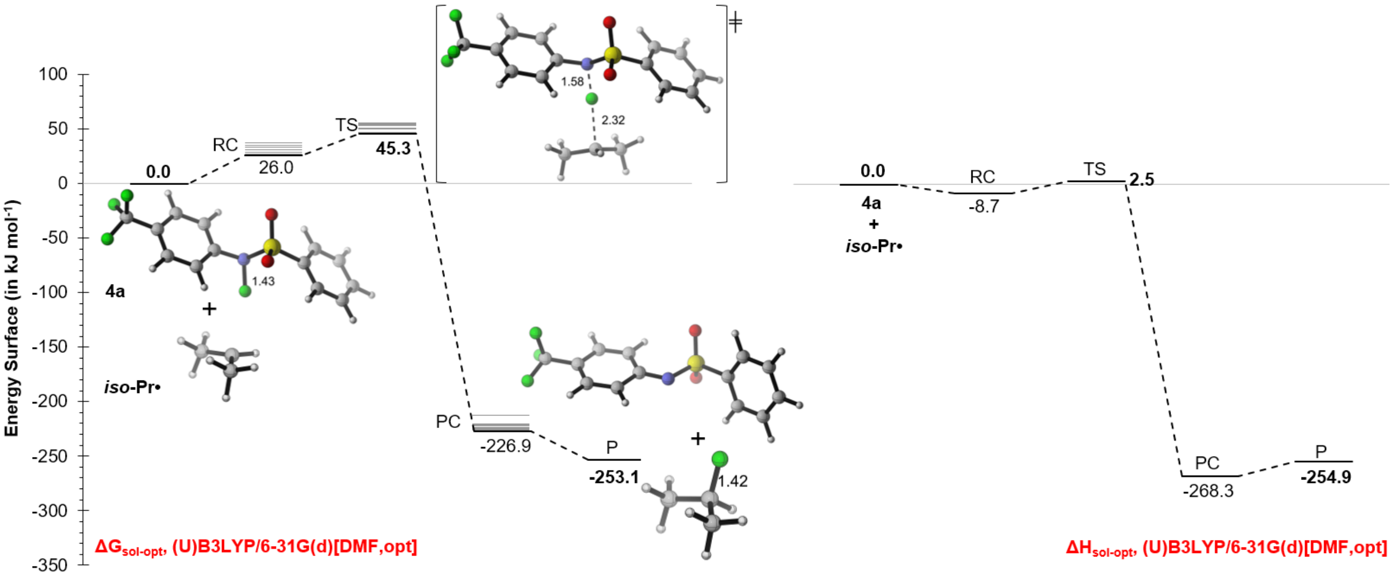

Supplementary Figure 237. Solvation phase optimized (DMF,opt) free energy ($\Delta G_{sol\text{-}opt}$) and enthalpy ($\Delta H_{sol\text{-}opt}$) surfaces (in kJ mol^{-1}) for fluorine atom transfer process between **4a** and ***iso*-Pr•** calculated at (U)B3LYP/6-31G(d) level.

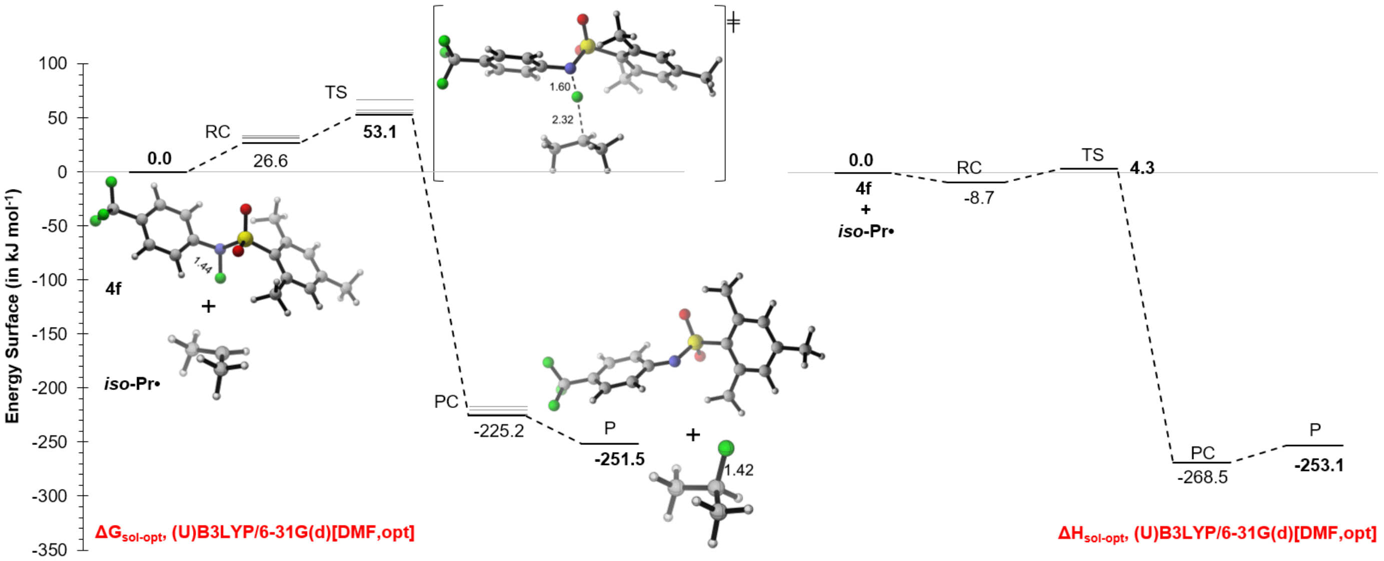

Supplementary Figure 238. Solvation phase optimized (DMF,opt) free energy ($\Delta G_{sol\text{-}opt}$) and enthalpy ($\Delta H_{sol\text{-}opt}$) surfaces (in kJ mol^{-1}) for fluorine atom transfer process between **4f** with ***iso*-Pr•** calculated at (U)B3LYP/6-31G(d) level.

QM Data (For Free Energy & Enthalpy Surfaces)

Supplementary Table 16. QM properties for gas phase optimized conformers calculated at different levels of theory.

	(U)B3LYP/6-31G(d)[gas]								(RO)B2PLYP/GTMP2large[gas]
System (filename)	ΔE_{tot} (hartree)	Low Frequency			corr. ZPE (hartree)	corr. ΔH (hartree)	corr. ΔG (hartree)	ΔG_{solv} (kcal mol^{-1})	ΔE_{tot} (hartree)
**Reference**									
**iso-Pr-F** (Fip_1)	-218.3780028	-15	0	0	0.096609	0.102594	0.069393	-2.85	-218.2828508
**iso-Pr•** (r_ipc_1)	-118.4781528	-33	-13	0	0.088606	0.094688	0.061409	-1.03	-118.3871285
**NFSI + iso-Pr•**									
**TS**									
nfsi_ipr_ts_15	-1833.4070197	-489	-6	-4	0.298455	0.323449	0.241038	-16.64	-1832.8259271
nfsi_ipr_ts_1	-1833.4059545	-495	-5	0	0.298617	0.323553	0.241366	-16.86	-1832.8248736
nfsi_ipr_ts_32	-1833.4055505	-475	-6	-3	0.298045	0.323285	0.238175	-17.27	-1832.8238478
nfsi_ipr_ts_23	-1833.4049743	-478	-5	0	0.298218	0.323359	0.239354	-17.51	-1832.8236301
nfsi_ipr_ts_30	-1833.4049180	-525	-6	-5	0.298338	0.323469	0.239226	-18.09	-1832.8250515
nfsi_ipr_ts_20	-1833.4047642	-522	-6	-3	0.298256	0.323439	0.239351	-17.95	-1832.8251445
nfsi_ipr_ts_34	-1833.4047340	-482	-3	0	0.298291	0.323430	0.239224	-17.88	-1832.8228765
nfsi_ipr_ts_16	-1833.4046443	-525	-5	0	0.298340	0.323464	0.240086	-18.45	-1832.8250893
nfsi_ipr_ts_25	-1833.4042767	-529	-6	-3	0.298552	0.323561	0.240393	-17.99	-1832.8250269
nfsi_ipr_ts_40	-1833.3994147	-535	-11	-7	0.298438	0.323461	0.239923	-19.58	-1832.8216906
nfsi_ipr_ts_47	-1833.3988020	-528	-8	-5	0.298514	0.323515	0.240844	-19.84	-1832.8209536
**RC**									
nfsi_ipr_ts_1_ircf_o	-1833.4141176	-6	-2	0	0.298811	0.324835	0.237355	-14.71	-1832.8334495
nfsi_ipr_ts_16_ircf_o	-1833.4140381	-2	0	0	0.298718	0.324775	0.237730	-15.62	-1832.8354394
nfsi_ipr_ts_30_ircf_o	-1833.4140381	-2	0	0	0.298718	0.324775	0.237730	-15.62	-1832.8354393
nfsi_ipr_ts_20_ircf_o	-1833.4138737	-5	-3	0	0.298667	0.324775	0.236713	-15.36	-1832.8353849
nfsi_ipr_ts_15_ircf_o	-1833.4136866	-5	0	0	0.298727	0.324811	0.237359	-14.97	-1832.8335871
nfsi_ipr_ts_25_ircf_o	-1833.4124153	-4	0	0	0.298223	0.324625	0.233378	-16.42	-1832.8338043
nfsi_ipr_ts_34_ircf_o	-1833.4121903	-10	-4	-1	0.298646	0.324744	0.236759	-15.63	-1832.8313085
nfsi_ipr_ts_23_ircf_o	-1833.4121075	-8	-5	-1	0.298587	0.324731	0.236625	-15.54	-1832.8318116
nfsi_ipr_ts_32_ircf_o	-1833.4120813	-5	0	0	0.298681	0.324747	0.237733	-16.11	-1832.8314781
nfsi_ipr_ts_47_ircf_o	-1833.4087187	-1	0	0	0.298888	0.324856	0.238118	-16.94	-1832.8315586
nfsi_ipr_ts_40_ircf_o	-1833.4078438	0	0	0	0.298729	0.324815	0.236847	-18.06	-1832.8306801
**PC**									
nfsi_ipr_ts_20_ircr_o	-1833.5004316	-2	0	0	0.302718	0.327849	0.242856	-15.61	-1832.9235142
nfsi_ipr_ts_23_ircr_o	-1833.5003992	-5	0	0	0.302688	0.327859	0.241965	-16.45	-1832.9214629
nfsi_ipr_ts_32_ircr_o	-1833.5003992	-5	0	0	0.302688	0.327859	0.241965	-16.45	-1832.9214629
nfsi_ipr_ts_40_ircr_o	-1833.5003992	-5	0	0	0.302688	0.327859	0.241965	-16.45	-1832.9214629
nfsi_ipr_ts_34_ircr_o	-1833.5003950	-2	0	0	0.302586	0.327857	0.241490	-16.29	-1832.9221672
nfsi_ipr_ts_16_ircr_o	-1833.4998081	0	0	0	0.302934	0.327935	0.244809	-16.24	-1832.9217431
nfsi_ipr_ts_30_ircr_o	-1833.4998081	0	0	0	0.302935	0.327935	0.244810	-16.24	-1832.9217432
nfsi_ipr_ts_47_ircr_o	-1833.4998081	0	0	0	0.302934	0.327935	0.244809	-16.24	-1832.9217431

nfsi_ipr_ts_25_ircr_o	-1833.4995788	-5	-2	0	0.302887	0.327926	0.244162	-16.06	-1832.9217995
nfsi_ipr_ts_1_ircr_o	-1833.4968514	-4	0	0	0.302875	0.327918	0.243162	-16.09	-1832.9189071
nfsi_ipr_ts_15_ircr_o	-1833.4958257	-5	0	0	0.302833	0.327868	0.243455	-16.37	-1832.9179787
**4a<br>+<br>iso-Pr●**									
**TS**									
a4_ipr_ts_16	-1621.8883110	-455	-7	-4	0.292937	0.318354	0.232330	-13.23	-1621.3462901
a4_ipr_ts_13	-1621.8882725	-472	-4	-2	0.292916	0.318328	0.232563	-13.57	-1621.3461040
a4_ipr_ts_15	-1621.8879046	-457	-4	-3	0.293006	0.318380	0.232876	-13.40	-1621.3462051
a4_ipr_ts_2	-1621.8861807	-545	-9	-1	0.293126	0.318429	0.233127	-13.95	-1621.3456680
a4_ipr_ts_10	-1621.8858199	-425	-3	0	0.292927	0.318258	0.233257	-12.75	-1621.3443495
a4_ipr_ts_9	-1621.8857922	-422	-6	-3	0.292847	0.318266	0.232749	-13.09	-1621.3437098
a4_ipr_ts_12	-1621.8857857	-424	-4	0	0.292910	0.318263	0.232989	-12.80	-1621.3442601
a4_ipr_ts_7	-1621.8849816	-554	-10	-3	0.293352	0.318521	0.234244	-14.06	-1621.3449103
a4_ipr_ts_1	-1621.8849380	-553	-10	-7	0.293273	0.318486	0.233732	-14.14	-1621.3448771
**RC**									
a4_ipr_ts_16_ircr_o	-1621.8972041	-5	-3	0	0.293511	0.319760	0.230400	-10.74	-1621.3565240
a4_ipr_ts_15_ircr_o	-1621.8969870	-6	-4	-2	0.293482	0.319751	0.230116	-10.67	-1621.3568288
a4_ipr_ts_2_ircr_o	-1621.8969791	-4	-3	0	0.293818	0.319888	0.232541	-11.42	-1621.3581768
a4_ipr_ts_1_ircr_o	-1621.8967296	-9	-3	0	0.293670	0.319845	0.231377	-11.31	-1621.3578027
a4_ipr_ts_13_ircr_o	-1621.8966084	-3	0	0	0.293334	0.319711	0.228296	-11.73	-1621.3548739
a4_ipr_ts_7_ircr_o	-1621.8961284	0	0	0	0.293596	0.319829	0.230432	-11.79	-1621.3568493
a4_ipr_ts_9_ircr_o	-1621.8921240	-4	0	0	0.293651	0.319810	0.231243	-11.93	-1621.3520105
a4_ipr_ts_12_ircr_o	-1621.8919585	-4	0	0	0.293547	0.319772	0.231013	-12.01	-1621.3522818
a4_ipr_ts_10_ircr_o	-1621.8919510	-3	0	0	0.293532	0.319764	0.231099	-11.98	-1621.3522758
**PC**									
a4_ipr_ts_12_ircf_o	-1621.9997241	0	0	0	0.297763	0.322888	0.236710	-11.45	-1621.4606654
a4_ipr_ts_10_ircf_o	-1621.9997212	0	0	0	0.297752	0.322883	0.236533	-11.44	-1621.4606553
a4_ipr_ts_9_ircf_o	-1621.9991112	-4	0	0	0.297549	0.322822	0.235285	-11.43	-1621.4605666
a4_ipr_ts_15_ircf_o	-1621.9980744	-6	-5	0	0.297653	0.322887	0.234740	-11.92	-1621.4590076
a4_ipr_ts_16_ircf_o	-1621.9975697	-5	0	0	0.297596	0.322861	0.235525	-12.15	-1621.4589204
a4_ipr_ts_13_ircf_o	-1621.9975428	-4	0	0	0.297663	0.322918	0.235144	-12.09	-1621.4586049
a4_ipr_ts_2_ircf_o	-1621.9975428	-4	0	0	0.297663	0.322918	0.235145	-12.09	-1621.4586049
a4_ipr_ts_1_ircf_o	-1621.9975321	-5	0	0	0.297544	0.322838	0.235118	-12.05	-1621.4588473
a4_ipr_ts_7_ircf_o	-1621.9966595	-3	-2	0	0.297983	0.323050	0.237573	-12.95	-1621.4556573
**4a<br>+<br>iso-Pr●**									
**TS**									
f4_ipr_ts_6	-1739.8347600	-495	-7	0	0.377016	0.407193	0.311835	-12.93	-1739.2085060
f4_ipr_ts_8	-1739.8333042	-502	-3	0	0.376928	0.407128	0.311611	-13.08	-1739.2073791
f4_ipr_ts_9	-1739.8332664	-501	-2	0	0.376947	0.407130	0.312095	-13.08	-1739.2073360
f4_ipr_ts_4	-1739.8324752	-447	-9	0	0.376705	0.406975	0.310986	-12.45	-1739.2066603
f4_ipr_ts_13	-1739.8324363	-450	-5	-3	0.376738	0.406957	0.311355	-12.02	-1739.2071641
f4_ipr_ts_15	-1739.8291656	-557	-7	0	0.377447	0.407422	0.313360	-13.62	-1739.2050092

f4_ipr_ts_1	-1739.8275078	-562	-8	-2	0.377413	0.407413	0.312917	-13.83	-1739.2034833
**RC**									
f4_ipr_ts_6_ircr_o	-1739.8438163	-2	0	0	0.377260	0.408467	0.306895	-11.34	-1739.2179213
f4_ipr_ts_8_ircr_o	-1739.8430031	-5	-1	0	0.377320	0.408498	0.307397	-11.14	-1739.2178872
f4_ipr_ts_9_ircf_o	-1739.8429465	-7	0	0	0.377296	0.408511	0.306452	-11.13	-1739.2178423
f4_ipr_ts_15_ircr_o	-1739.8408814	-8	0	0	0.377373	0.408510	0.308112	-11.46	-1739.2171689
f4_ipr_ts_1_ircf_o	-1739.8402058	-5	-3	0	0.377398	0.408541	0.307433	-11.30	-1739.2171765
f4_ipr_ts_4_ircr_o	-1739.8394897	-2	-1	0	0.377372	0.408480	0.309041	-11.15	-1739.2156884
f4_ipr_ts_13_ircr_o	-1739.8394538	-6	-2	0	0.377464	0.408477	0.309738	-11.18	-1739.2162066
**PC**									
f4_ipr_ts_13_ircf_o	-1739.9456011	-2	0	0	0.381533	0.411567	0.315192	-11.04	-1739.3218384
f4_ipr_ts_1_ircr_o	-1739.9450187	-6	0	0	0.381343	0.411475	0.314577	-11.11	-1739.3217324
f4_ipr_ts_15_ircf_o	-1739.9450187	-6	0	0	0.381343	0.411475	0.314577	-11.11	-1739.3217325
f4_ipr_ts_4_ircf_o	-1739.9449956	-5	-1	0	0.381302	0.411468	0.313956	-11.14	-1739.3217089
f4_ipr_ts_8_ircf_o	-1739.9409229	-5	0	0	0.381319	0.411473	0.313443	-12.61	-1739.3187105
f4_ipr_ts_9_ircr_o	-1739.9403437	-10	-1	0	0.381455	0.411459	0.314486	-13.33	-1739.3176809
f4_ipr_ts_6_ircf_o	-1739.9402512	-4	-2	0	0.381576	0.411589	0.314384	-13.27	-1739.3175614

Supplementary Table 17. QM properties for solvation phase optimized (DMF,opt) conformers calculated at different levels of theory.

	SMD(DMF)/(U)B3LYP/6-31G(d)[DMF,opt]								(RO)B2PLYP/GTMP2large[DMF,opt]
System (filename)	ΔE_{tot} (hartree)	Low Frequency			corr. ZPE (hartree)	corr. ΔH (hartree)	corr. ΔG (hartree)	ΔG_{solv} (kcal mol^{-1})	ΔE_{tot} (hartree)
**Reference**									
**iso-Pr-F** (Fip_1)	-218.3826405	-33	0	0	0.096301	0.102290	0.069081	-3.01	-218.2828046
**iso-Pr●** (r_ipc_1)	-118.4798327	-51	-42	0	0.088420	0.094398	0.061560	-1.06	-118.3871151
**NFSI + iso-Pr●**									
**R**									
nfsi_1	-1714.9565976	-3	0	0	0.208813	0.227731	0.160946	-16.15	-1714.4418479
nfsi_10	-1714.9565046	-7	0	0	0.208685	0.227713	0.159951	-17.42	-1714.4391742
nfsi_11	-1714.9568385	-8	-1	0	0.208499	0.227605	0.159090	-17.99	-1714.4386866
nfsi_3	-1714.9555606	-8	0	0	0.208852	0.227715	0.161554	-19.10	-1714.4392459
nfsi_4	-1714.9575240	-5	-3	0	0.208667	0.227673	0.160018	-16.64	-1714.4429867
**TS**									
nfsi_ipr_ts_25	-1833.4346015	-573	-15	0	0.297685	0.322880	0.238472	-19.10	-1832.8242006
nfsi_ipr_ts_32	-1833.4344286	-546	-10	-8	0.297504	0.322726	0.239374	-19.38	-1832.8217678
nfsi_ipr_ts_16	-1833.4347109	-566	-9	-5	0.297735	0.322856	0.239678	-19.26	-1832.8242492
nfsi_ipr_ts_30	-1833.4347109	-566	-9	-5	0.297736	0.322856	0.239678	-19.26	-1832.8242492
nfsi_ipr_ts_20	-1833.4343735	-575	-7	-4	0.297718	0.322850	0.240091	-19.14	-1832.8240777
nfsi_ipr_ts_34	-1833.4344331	-547	-6	0	0.297686	0.322785	0.240189	-19.48	-1832.8216030
nfsi_ipr_ts_15	-1833.4346103	-548	-4	-3	0.297665	0.322728	0.240678	-18.10	-1832.8246263
nfsi_ipr_ts_23	-1833.4344246	-544	-6	0	0.297749	0.322795	0.240633	-19.45	-1832.8216524
nfsi_ipr_ts_1	-1833.4339682	-554	-7	0	0.297791	0.322867	0.240689	-18.20	-1832.8239416
**RC**									
nfsi_ipr_ts_25_ircf_o	-1833.4400074	-6	0	0	0.298415	0.324292	0.238200	-17.17	-1832.8333907
nfsi_ipr_ts_30_ircf_o	-1833.4400074	-6	0	0	0.298415	0.324292	0.238199	-17.17	-1832.8333908
nfsi_ipr_ts_16_ircf_o	-1833.4399657	-6	0	0	0.298249	0.324250	0.237334	-17.24	-1832.8331342
nfsi_ipr_ts_20_ircf_o	-1833.4398889	-8	-4	0	0.298072	0.324150	0.237050	-17.10	-1832.8334976
nfsi_ipr_ts_15_ircf_o	-1833.4389317	-11	0	0	0.298290	0.324323	0.237265	-16.82	-1832.8318454
nfsi_ipr_ts_34_ircf_o	-1833.4385889	0	0	0	0.297992	0.324165	0.236475	-18.37	-1832.8281577
nfsi_ipr_ts_1_ircf_o	-1833.4385701	0	0	0	0.298125	0.324246	0.236833	-16.79	-1832.8317861
nfsi_ipr_ts_32_ircf_o	-1833.4384636	-7	0	0	0.297999	0.324158	0.235667	-18.26	-1832.8285487
nfsi_ipr_ts_23_ircf_o	-1833.4383246	-8	0	0	0.297777	0.324062	0.235608	-18.75	-1832.8279687
**PC**									
nfsi_ipr_ts_16_ircr_o	-1833.5266161	-8	-4	0	0.302235	0.327304	0.243375	-17.53	-1832.9196584
nfsi_ipr_ts_25_ircr_o	-1833.5266161	-8	-4	0	0.302235	0.327304	0.243374	-17.53	-1832.9196586
nfsi_ipr_ts_30_ircr_o	-1833.5266161	-8	-4	0	0.302235	0.327304	0.243375	-17.53	-1832.9196585
nfsi_ipr_ts_23_ircr_o	-1833.5260913	0	0	0	0.302215	0.327361	0.242471	-19.18	-1832.9159499
nfsi_ipr_ts_34_ircr_o	-1833.5259812	0	0	0	0.302232	0.327326	0.243128	-18.88	-1832.9161295
nfsi_ipr_ts_20_ircr_o	-1833.5258814	-10	-1	0	0.302208	0.327377	0.242520	-18.26	-1832.9178437
nfsi_ipr_ts_32_ircr_o	-1833.5251574	0	0	0	0.301844	0.327117	0.241224	-20.48	-1832.9149277
nfsi_ipr_ts_1_ircr_o	-1833.5240081	0	0	0	0.302601	0.327442	0.244582	-18.18	-1832.9165142

nfsi_ipr_ts_15_ircr_o	-1833.5234905	-8	0	0	0.302320	0.327279	0.243800	-18.59	-1832.9157344
**P**									
r_nfsi_1	-1615.1392295	-20	-13	-6	0.204607	0.221875	0.156998	-16.14	-1614.6331659
r_nfsi_10	-1615.1406591	-8	0	0	0.204694	0.222781	0.155624	-17.69	-1614.6300007
**4a + iso-Pr●**									
**R**									
a4_1	-1503.4331568	0	0	0	0.203507	0.222571	0.154110	-11.48	-1502.9653785
a4_10	-1503.4336625	-7	0	0	0.203639	0.222613	0.155080	-12.08	-1502.9665925
a4_4	-1503.4330958	-6	0	0	0.203402	0.222516	0.153438	-11.48	-1502.9653160
a4_5	-1503.4336761	-5	0	0	0.203646	0.222605	0.155084	-12.08	-1502.9666148
**TS**									
a4_ipr_ts_13	-1621.9100239	-516	-6	0	0.292074	0.317533	0.232381	-13.81	-1621.3457033
a4_ipr_ts_16	-1621.9098877	-516	-12	-5	0.292208	0.317625	0.232311	-13.81	-1621.3457538
a4_ipr_ts_15	-1621.9100340	-511	-4	0	0.292064	0.317502	0.232888	-13.77	-1621.3459624
a4_ipr_ts_10	-1621.9076661	-496	-11	-6	0.291945	0.317441	0.231565	-14.26	-1621.3430763
a4_ipr_ts_2	-1621.9089485	-566	-18	-7	0.292426	0.317716	0.233315	-15.00	-1621.3446017
a4_ipr_ts_12	-1621.9074222	-494	-5	0	0.292160	0.317519	0.232433	-14.33	-1621.3430746
a4_ipr_ts_1	-1621.9082624	-569	-12	-1	0.292374	0.317629	0.233682	-14.98	-1621.3440986
a4_ipr_ts_9	-1621.9076720	-499	-2	0	0.292238	0.317545	0.233366	-14.32	-1621.3430937
a4_ipr_ts_7	-1621.9082349	-570	-14	-9	0.292598	0.317753	0.234352	-14.99	-1621.3440715
**RC**									
a4_ipr_ts_2_ircr_o	-1621.9160834	-7	0	0	0.293318	0.319287	0.232666	-12.66	-1621.3567087
a4_ipr_ts_15_ircf_o	-1621.9158318	0	0	0	0.293027	0.319167	0.230841	-12.32	-1621.3543400
a4_ipr_ts_16_ircr_o	-1621.9157037	0	0	0	0.292933	0.319009	0.232244	-12.35	-1621.3551153
a4_ipr_ts_1_ircr_o	-1621.9155293	-5	0	0	0.293272	0.319284	0.232324	-12.84	-1621.3565616
a4_ipr_ts_13_ircf_o	-1621.9151583	0	0	0	0.292624	0.318911	0.230307	-12.59	-1621.3544337
a4_ipr_ts_7_ircr_o	-1621.9149186	0	0	0	0.292849	0.319045	0.230643	-13.74	-1621.3545283
a4_ipr_ts_9_ircr_o	-1621.9128066	-4	0	0	0.293069	0.319105	0.231774	-13.65	-1621.3493227
a4_ipr_ts_10_ircr_o	-1621.9127334	-6	0	0	0.292841	0.319019	0.230884	-13.69	-1621.3491922
a4_ipr_ts_12_ircr_o	-1621.9115640	0	0	0	0.292398	0.318792	0.229301	-13.73	-1621.3492556
**PC**									
a4_ipr_ts_7_ircf_o	-1622.0182334	0	0	0	0.297575	0.322540	0.238430	-14.96	-1621.4532181
a4_ipr_ts_2_ircf_o	-1622.0176068	-8	0	0	0.297261	0.322348	0.236996	-13.54	-1621.4566832
a4_ipr_ts_16_ircf_o	-1622.0174744	0	0	0	0.297104	0.322266	0.236525	-14.29	-1621.4559275
a4_ipr_ts_13_ircr_o	-1622.0160075	-6	-4	0	0.296723	0.322023	0.234671	-15.03	-1621.4541428
a4_ipr_ts_15_ircr_o	-1622.0159467	-8	0	0	0.296959	0.322119	0.235578	-14.65	-1621.4546525
a4_ipr_ts_12_ircf_o	-1622.0154275	-5	0	0	0.297038	0.322199	0.236032	-13.63	-1621.4554771
a4_ipr_ts_10_ircf_o	-1622.0150480	-7	-4	0	0.296827	0.322037	0.235529	-14.29	-1621.4538680
a4_ipr_ts_9_ircf_o	-1622.0149589	-5	0	0	0.296868	0.322043	0.235819	-14.63	-1621.4532221
a4_ipr_ts_1_ircf_o	-1622.0131250	-2	0	0	0.296945	0.322064	0.237143	-17.02	-1621.4494498
**P**									
r_a4_1	-1403.6309148	-17	-7	0	0.199597	0.217689	0.150136	-12.06	-1403.1697487
**4a + iso-Pr●**									
**R**									

f4_1	-1621.3772910	0	0	0	0.287471	0.311145	0.234443	-11.38	-1620.8271825
f4_10	-1621.3795400	-9	-5	0	0.287044	0.310925	0.232393	-11.01	-1620.8280611
f4_11	-1621.3795371	-12	-8	0	0.286957	0.310887	0.232125	-10.99	-1620.8280948
f4_3	-1621.3773136	0	0	0	0.287423	0.311129	0.234104	-11.39	-1620.8272297
**TS**									
f4_ipr_ts_6	-1739.8555835	-534	-4	0	0.376154	0.406138	0.313136	-13.22	-1739.2081783
f4_ipr_ts_9	-1739.8543224	-540	-7	-1	0.376030	0.406011	0.312473	-13.35	-1739.2071277
f4_ipr_ts_8	-1739.8543183	-538	-7	-3	0.376051	0.406023	0.312616	-13.35	-1739.2071229
f4_ipr_ts_4	-1739.8531584	-510	0	0	0.375953	0.406022	0.312286	-13.61	-1739.2058914
f4_ipr_ts_15	-1739.8511041	-588	-7	-2	0.376692	0.406505	0.313826	-14.22	-1739.2043811
**RC**									
f4_ipr_ts_6_ircr_o	-1739.8618620	0	0	0	0.376452	0.407493	0.309332	-11.94	-1739.2175682
f4_ipr_ts_8_ircr_o	-1739.8613389	-8	0	0	0.376392	0.407318	0.310432	-12.27	-1739.2172272
f4_ipr_ts_15_ircr_o	-1739.8595327	-3	0	0	0.376739	0.407619	0.309744	-12.30	-1739.2162727
f4_ipr_ts_4_ircr_o	-1739.8587225	-8	-3	0	0.376355	0.407424	0.308311	-12.80	-1739.2132029
**PC**									
f4_ipr_ts_4_ircf_o	-1739.9640611	-5	0	0	0.380827	0.410719	0.315873	-12.67	-1739.3194123
f4_ipr_ts_6_ircf_o	-1739.9621157	-3	0	0	0.380482	0.410469	0.315575	-14.80	-1739.3161002
f4_ipr_ts_8_ircf_o	-1739.9620085	-7	-4	0	0.380443	0.410542	0.313557	-14.61	-1739.3161948
f4_ipr_ts_9_ircr_o	-1739.9619996	-4	0	0	0.380388	0.410522	0.313913	-14.67	-1739.3157436
f4_ipr_ts_15_ircf_o	-1739.9606078	-6	0	0	0.380571	0.410584	0.315040	-14.99	-1739.3135257
**P**									
r_f4_1	-1521.5762673	-15	-8	0	0.283267	0.306127	0.228333	-11.65	-1521.0312274

References

1 Kapat, A., König, A., Montermini, F. & Renaud, P. A radical procedure for the anti-Markovnikov hydroazidation of alkenes. *J. Am. Chem. Soc.* **133**, 13890-13893, doi:10.1021/ja2054989 (2011).
2 Bartlett, P. D. & Rüchardt, C. Peresters. IV. Substituent effects upon the concerted decomposition of *t*-butyl phenylperacetates. *J. Am. Chem. Soc.* **82**, 1756-1762, doi:10.1021/ja01492a054 (1960).
3 Brown, H. C. *et al.* Organoboranes. 27. Exploration of synthetic procedures for the preparation of monoisopinocampheylborane. *J. Org. Chem.* **47**, 5069-5074, doi:10.1021/jo00147a005 (1982).
4 Brown, H. C. & Singaram, B. Hydroboration. 68. Chiral synthesis via organoboranes. 1. A simple procedure to achieve products of essentially 100% optical purity in hydroboration of alkenes with monoisopinocampheylborane. Synthesis of boronic esters and derived products of very high enantiomeric purities. *J. Am. Chem. Soc.* **106**, 1797-1800, doi:10.1021/ja00318a040 (1984).
5 Brown, C. H. *Organic synthesis via boranes*. (John Wiley&Sons, Inc., 1975).
6 Schunk, S. *et al.* Pyrimidine and triazine sulfonamide derivates as b1 bradykinin receptor (b1r) inhibitors for treating pain WO 2010046109 (2010).
7 Binisti, C. *et al.* Structure–activity relationships in platelet-activating factor (PAF). 11-From PAF-antagonism to phospholipase A2 inhibition: syntheses and structure–activity relationships in 1-arylsulfamido-2-alkylpiperazines. *Eur. J. Med. Chem.* **36**, 809-828, doi:10.1016/S0223-5234(01)01274-0 (2001).
8 Rono, L. J., Yayla, H. G., Wang, D. Y., Armstrong, M. F. & Knowles, R. R. Enantioselective photoredox catalysis enabled by proton-coupled electron transfer: development of an asymmetric aza-pinacol cyclization. *J. Am. Chem. Soc.* **135**, 17735-17738, doi:10.1021/ja4100595 (2013).
9 Chen, B.-S. *et al.* Diastereoselective synthesis and bioactivity of long-chain anti-2-amino-3-alkanols. *Eur. J. Med. Chem.* **46**, 5480-5486, doi:10.1016/j.ejmech.2011.09.010 (2011).
10 Meyer, D. & Renaud, P. Enantioselective hydroazidation of trisubstituted non-activated alkenes. *Angew. Chem. Int. Ed.* **56**, 10858-10861, doi:10.1002/anie.201703340 (2017).
11 Duan, X.-F., Zeng, J., Lü, J.-W. & Zhang, Z.-B. Insights into the general and efficient cross McMurry reactions between ketones. *J. Org. Chem.* **71**, 9873-9876, doi:10.1021/jo061644d (2006).
12 Maher, M. J., Schibur, H. J. & Bates, F. S. When convergent syntheses of graft block copolymers diverge: the treachery of chemical images. *J. Polym. Sci., Part A: Polym. Chem.* **55**, 3097-3104, doi:10.1002/pola.28660 (2017).
13 Catti, L. & Tiefenbacher, K. Intramolecular hydroalkoxylation catalyzed inside a self-assembled cavity of an enzyme-like host structure. *Chem. Commun.* **51**, 892-894, doi:10.1039/C4CC08211G (2015).
14 Roman, M. *et al.* Supramolecular balance: using cooperativity to amplify weak interactions. *J. Am. Chem. Soc.* **132**, 16818-16824, doi:10.1021/ja105717u (2010).
15 Nösel, P. *et al.* Oxidative gold catalysis meets photochemistry – Synthesis of benzo[a]fluorenones from diynes. *Adv. Synth. Catal.* **356**, 3755-3760, doi:10.1002/adsc.201400969 (2014).
16 Rueda-Becerril, M. *et al.* Fluorine transfer to alkyl radicals. *J. Am. Chem. Soc.* **134**, 4026-4029, doi:10.1021/ja211679v (2012).
17 Kaufmann, D., Bialer, M., Shimshoni, J. A., Devor, M. & Yagen, B. Synthesis and evaluation of antiallodynic and anticonvulsant activity of novel amide and urea derivatives of valproic acid analogues. *J. Med. Chem.* **52**, 7236-7248, doi:10.1021/jm901229s (2009).

18 Wang, Y., Zheng, Z. & Zhang, L. Intramolecular insertions into unactivated C(sp3)–H bonds by oxidatively generated β-diketone-α-gold carbenes: synthesis of cyclopentanones. *J. Am. Chem. Soc.* **137**, 5316-5319, doi:10.1021/jacs.5b02280 (2015).
19 Tanner, D. D., Yabuuchi, H. & Lutzer, H. Kinetic studies on the mechanism of the decomposition of a number of β-substituted *tert*-butyl α, α-dimethylperpropionates. *Can. J. Chem.* **55**, 612-618, doi:10.1139/v77-087 (1977).
20 Taylor, D. M. & Meier, G. P. A facile transfer fluorination approach to the synthesis of *N*-fluoro sulfonamides. *Tetrahedron Lett.* **41**, 3291-3294, doi:10.1016/S0040-4039(00)00525-6 (2000).
21 Niu, B. *et al.* Synthesis of nitromethyl-substituted oxindole derivatives via a desulfonylation cascade. *Synlett* **26**, 635-638, doi:10.1055/s-0034-1378947 (2015).
22 Macchi, P., Burgi, H.-B., Chimpri, A. S., Hauser, J. & Gal, Z. Low-energy contamination of Mo microsource X-ray radiation: analysis and solution of the problem. *J. Appl. Crystallogr.* **44**, 763-771, doi:10.1107/S0021889811016232 (2011).
23 Sheldrick, G. SHELXT - Integrated space-group and crystal-structure determination. *Acta Crystal. Sect. A* **71**, 3-8, doi:10.1107/S2053273314026370 (2015).
24 CrysAlisPRO v. 1.171.34.44 (Oxford Diffraction, Yarnton, UK, 2010).
25 Villa, G., Povie, G. & Renaud, P. Radical chain reduction of alkylboron compounds with catechols. *J. Am. Chem. Soc.* **133**, 5913-5920, doi:10.1021/ja110224d (2011).
26 Yin, F., Wang, Z., Li, Z. & Li, C. Silver-catalyzed decarboxylative fluorination of aliphatic carboxylic acids in aqueous solution. *J. Am. Chem. Soc.* **134**, 10401-10404, doi:10.1021/ja3048255 (2012).
27 Zipse, H. in *Radicals in Synthesis I - Method and Mechanisms* Vol. 263 *Topics in Current Chemistry* (ed Andreas Gansäuer) 163-189 (Springer, 2006).
28 Hioe, J. & Zipse, H. Radical stability and its role in synthesis and catalysis. *Org. Biomol. Chem.* **8**, 3609-3617, doi:10.1039/C004166A (2010).
29 Hioe, J. & Zipse, H. Radicals in enzymatic catalysis - a thermodynamic perspective. *Faraday Discuss.* **145**, 301-313, doi:10.1039/B907121K (2010).
30 Hioe, J., Savasci, G., Brand, H. & Zipse, H. The stability of Cα peptide radicals: why glycyl radical enzymes? *Chem. Eur. J.* **17**, 3781-3789, doi:10.1002/chem.201002620 (2011).
31 Hioe, J. & Zipse, H. Hydrogen transfer in SAM-mediated enzymatic radical reactions. *Chem. Eur. J.* **18**, 16463-16472, doi:10.1002/chem.201202869 (2012).
32 Hioe, J., Mosch, M., Smith, D. M. & Zipse, H. Dissociation energies of Cα–H bonds in amino acids – A re-examination. *RSC Adv.* **3**, 12403-12408, doi:10.1039/C3RA42115E (2013).
33 Šakić, D. & Zipse, H. Radical stability as a guideline in C–H amination reactions. *Adv. Synth. Catal.* **358**, 3983-3991 (2016).
34 Becke, A. D. Density-functional thermochemistry. III. The role of exact exchange. *J. Chem. Phys.* **98**, 5648-5652, doi:10.1063/1.464913 (1993).
35 Ditchfield, R., Hehre, W. J. & Pople, J. A. Self-consistent molecular-orbital methods. IX. An extended Gaussian-type basis for molecular-orbital studies of organic molecules. *J. Chem. Phys.* **54**, 724-728, doi:10.1063/1.1674902 (1971).
36 Krishnan, R., Binkley, J. S., Seeger, R. & Pople, J. A. Self-consistent molecular orbital methods. XX. A basis set for correlated wave functions. *J. Chem. Phys.* **72**, 650-654, doi:10.1063/1.438955 (1980).
37 Graham, D. C., Menon, A. S., Goerigk, L., Grimme, S. & Radom, L. Optimization and basis-set dependence of a restricted-open-shell form of B2-PLYP double-hybrid density functional theory. *J. Phys. Chem. A* **113**, 9861-9873, doi:10.1021/jp9042864 (2009).
38 Henry, D. J., Sullivan, M. B. & Radom, L. G3-RAD and G3X-RAD: Modified Gaussian-3 (G3) and Gaussian-3X (G3X) procedures for radical thermochemistry. *J. Chem. Phys.* **118**, 4849-4860, doi:10.1063/1.1544731 (2003).
39 Marenich, A. V., Cramer, C. J. & Truhlar, D. G. Universal solvation model based on solute electron density and on a continuum model of the solvent defined by the bulk

dielectric constant and atomic surface tensions. *J. Phys. Chem. B* **113**, 6378-6396, doi:10.1021/jp810292n (2009).
40 Luo, Y.-R. *Comprehensive handbook of chemical bond energies*. (CRC press, 2007).
41 Mordaunt, D. H., Ashfold, M. N. R. & Dixon, R. N. Photodissociation dynamics of Ã state ammonia molecules. I. State dependent μ-v correlations in the $NH_2(ND_2)$ products. *J. Chem. Phys.* **104**, 6460-6471, doi:10.1063/1.471367 (1996).
42 Chan, B. & Radom, L. BDE261: A comprehensive set of high-level theoretical bond dissociation enthalpies. *J. Phys. Chem. A* **116**, 4975-4986, doi:10.1021/jp302542z (2012).
43 Glendening, E. D., Landis, C. R. & Weinhold, F. NBO 6.0: Natural bond orbital analysis program. *J. Computat. Chem.* **34**, 1429-1437, doi:10.1002/jcc.23266 (2013).
44 Gaussian 09, Revision D.01 (Gaussian, Inc., Wallingford CT, 2013).
