# Supplementary material for: A third generation of radical fluorinating agents based on N-fluoro-N-arylsulfonamides
Source: Nat Commun. 2018 Nov 20;9:4888. doi: 10.1038/s41467-018-07196-9 (PMC6244228; doi:10.1038/s41467-018-07196-9)
Supplement: Supplementary file 3 — Description of Additional Supplementary Files [file 41467_2018_7196_MOESM3_ESM.pdf]

**Description of Additional Supplementary Files**

File Name: Supplementary Data 1
Description: Coordinates for supplementary table 9

File Name: Supplementary Data 2
Description: Coordinates for supplementary table 16

File Name: Supplementary Data 3
Description: Coordinates for supplementary table 17
